# Mapping the landscape of synthetic lethal interactions in liver cancer

**DOI:** 10.7150/thno.63416

**Published:** 2021-08-26

**Authors:** Chen Yang, Yuchen Guo, Ruolan Qian, Yiwen Huang, Linmeng Zhang, Jun Wang, Xiaowen Huang, Zhicheng Liu, Wenxin Qin, Cun Wang, Huimin Chen, Xuhui Ma, Dayong Zhang

**Affiliations:** 1 Department of Clinical Medicine, School of Medicine, Zhejiang University City College, Hangzhou, China.; 2 State Key Laboratory of Oncogenes and Related Genes, Shanghai Cancer Institute, Renji Hospital, Shanghai Jiao Tong University School of Medicine, Shanghai, China.; 3 Department of Clinical Medicine, Fujian Medical University, Fuzhou, China.; 4 Division of Gastroenterology and Hepatology, Key Laboratory of Gastroenterology and Hepatology, Renji Hospital, School of Medicine, Shanghai Jiao Tong University, Shanghai, China.; 5 Hepatic Surgery Center, Tongji Hospital, Tongji Medical College, Huazhong University of Science and Technology, Wuhan, China.

**Keywords:** liver cancer, synthetic lethality, precision medicine, TP53, PLK1

## Abstract

Almost all the current therapies against liver cancer are based on the “one size fits all” principle and offer only limited survival benefit. Fortunately, synthetic lethality (SL) may provide an alternate route towards individualized therapy in liver cancer. The concept that simultaneous losses of two genes are lethal to a cell while a single loss is non-lethal can be utilized to selectively eliminate tumors with genetic aberrations.

**Methods:** To infer liver cancer-specific SL interactions, we propose a computational pipeline termed SiLi (statistical inference-based synthetic lethality identification) that incorporates five inference procedures. Based on large-scale sequencing datasets, SiLi analysis was performed to identify SL interactions in liver cancer.

**Results:** By SiLi analysis, a total of 272 SL pairs were discerned, which included 209 unique target candidates. Among these, polo-like kinase 1 (*PLK1*) was considered to have considerable therapeutic potential. Further computational and experimental validation of the SL pair *TP53-PLK1* demonstrated that inhibition of PLK1 could be a novel therapeutic strategy specifically targeting those patients with *TP53*-mutant liver tumors.

**Conclusions:** In this study, we report a comprehensive analysis of synthetic lethal interactions of liver cancer. Our findings may open new possibilities for patient-tailored therapeutic interventions in liver cancer.

## Introduction

Recent rapid advances in individualized therapy have revolutionized cancer prognosis, making cancers previously considered to be lethal, such as breast cancer and lung cancer, manageable and even curable 1. However, it remains challenging to realize individualized therapy for liver cancer. Almost all the approved therapies against liver cancer lack corresponding biomarkers for predicting efficacy and can only yield marginal survival benefit 2, 3. Considering that the global burden of liver cancer has been increasing year over year, novel therapeutics are urgently required to counter this global health threat.

By sequencing large-scale clinical samples, the roles of many mutated genes in the occurrence and development of liver cancer have been elucidated 2. In many cancer types, genetically targeted therapies have been successfully applied to target mutated gene products. However, unlike *EGFR* 4 or *ALK* 5 mutations in lung cancer, most liver cancer mutations are undruggable with conventional approaches. For example, *TP53* mutation, the most common genetic change accounting for ~30% of all liver cancer cases, is currently difficult to target therapeutically for the immaturity of p53-targeted agonists 6. Fortunately, an appealing concept called synthetic lethality (SL) provides a promising approach to target mutated genes previously considered to be undruggable 7. The original concept of SL reflects relationships between two genes in which their simultaneous disruption causes cell death whereas functional loss of only one of the genes does not affect cell viability 7. By harnessing this concept, selective elimination of cells with mutations in tumor suppressor genes (TSGs), such as *TP53*, can be realized using conventional chemical inhibitors. A classic example of the application of this concept is PARP inhibitors used for the treatment of tumors with *BRCA* mutations, which currently represent the only SL-based therapeutics approved by the United States Food and Drug Administration (FDA) 8. Many expanded SL concepts have also been developed, such as synthetic dosage lethality 7, 9, metabolic SL 10, collateral SL 11, and conditional SL 7, offering additional possibilities for the development of SL-based therapies.

To date, many experimental and computational approaches have been adopted to screen potential SL interactions. Experimental SL screening approaches can be broadly classified into two categories: model organism-based and human cell-based screening. Model organism-based screens mainly depend on synthetic genetic array analysis 12 and diploid synthetic lethality analysis with microarrays 13, while human cell-based screens usually utilize RNA interference (RNAi) and clustered regularly interspaced short palindromic repeats (CRISPR) technologies 14, 15. As alternatives to experimental methods, emerging computational approaches, such as DAISY 16, MiSL 17, ISLE 18, and DiscoverSL 19, can offer convenient and low-cost SL prediction. However, most established approaches utilize pan-cancer data for SL inference and do not focus on a specific cancer type. Accordingly, detailed SL interactions in liver cancer remain under-explored. To fill this gap, we propose a new approach tailored to liver cancer, termed statistical inference-based synthetic lethality identification (SiLi). Based on this approach, we determined the landscape of SL interactions in liver cancer, which might provide new opportunities for highly specific therapeutic strategies.

## Methods

### Clinical Data

Seven clinical cohorts of liver cancer, including TCGA-LIHC 20, LINC-JP 21, LIRI-JP 22, LICA-FR 23, KOREAN 24, LICA-CN, and CHCC-HBV 25, were included in this study, which comprised 1,825 patients from multiple geographically different origins. Of these, TCGA-LIHC, LIRI-JP, LICA-FR, and CHCC-HBV cohorts had both RNA sequencing (RNAseq) and whole genome/exome sequencing (WGS/WES) data available, while LINC-JP, KOREAN, and LICA-CN cohorts only contained WGS/WES data. Among the four cohorts with available transcriptome data, TCGA-LIHC, LIRI-JP, and LICA-FR cohorts provided raw counts of gene expression, while CHCC-HBV cohort provided fragments per kilobase per million reads (FPKM) normalized data. For consistency, both raw counts and FPKM were transformed into transcripts per kilobase million (TPM) values that were more comparable between samples. As for the mutation data, in this study, we focused on the single nucleotide variants (SNVs) and small insertions/deletions (indels). Copy number variants (CNVs) profiles were not considered due to the data limitations. Non-functional mutations, including silent mutations (synonymous mutations) and mutations in intronic or intergenic regions, were first excluded, and only cases with functional mutations were considered as gene mutation events. Samples without functional mutations or fewer than 10 mutations in gene panels were considered as outliers and discarded from downstream analyses. Genes with mutation frequencies less than 2.5% were also excluded. Loss-of-function (LOF) or inactivating mutations were defined as any truncating mutation (frameshift and nonsense mutations), the resulting proteins of which were usually nonfunctional. Duplicated mutations in each sample were merged to keep only one record. For survival analysis, three cohorts (TCGA-LIHC, LIRI-JP, and CHCC-HBV) provided follow-up information. The survival data of TCGA-LIHC cohort were achieved from the TCGA Pan-Cancer Clinical Data Resource (TCGA-CDR) 26, while the survival data of LIRI-JP and CHCC-HBV cohorts were obtained from supplementary files of reference 22, 25.

### Cancer Cell Line Data

Expression profiles of human cancer cell lines were obtained from the Broad Institute Cancer Cell Line Encyclopedia (CCLE) project (based on RNAseq) 27 and the Wellcome Sanger Institute Genomics of Drug Sensitivity in Cancer (GDSC) project (based on microarray) 28. Drug response data were achieved from the Cancer Therapeutics Response Portal (CTRPv.2.0, released October 2015) 29, GDSC1&2 (released October 2019) 28, and PRISM Repurposing dataset (19Q4, released December 2019) 30, respectively. CTRP had the sensitivity data of 481 compounds across 835 cell lines, GDSC contained the sensitivity data of 396 compounds across 773 cell lines, while PRISM included the sensitivity data of 1448 compounds across 482 cell lines. All the three datasets provided area under the dose-response curve (AUC) values as a measure of drug sensitivity, where lower AUC values indicate increased sensitivity to treatments. Notably, cell lines in CTRP and PRISM were obtained from the CCLE project, and molecular data in CCLE were thus used for CTRP and PRISM analyses. For gene dependency data, genome-wide gene dependency scores, including CERES scores from CRISPR knockout screens and DEMETER scores for RNAi screens, were downloaded from the Cancer Dependency Map (DepMap) portal 31, 32. Lower CERES or DEMETER scores indicate that corresponding genes are more likely to be essential in cell growth and survival.

### Driver Genes Detection

*DriverNet* algorithm was utilized to discern potential driver genes of liver cancer 33. By exploiting influence graph, this algorithm integrates genome and transcriptome data to evaluate the driver mutation probability. The input files of *DriverNet* include an influence graph, a mutation matrix, and a corresponding gene expression matrix. In this study, the influence graph was constructed using an updated protein functional interaction network (Version 2019, Reactome) 34. The output of *DriverNet* is the significance of imported genes. Genes with adjusted *P* < 0.05 were deemed significant. To determine whether our prediction was reliable, we compiled a comprehensive list of liver cancer-associated driver genes from 14 previously published studies and compared our prediction with previous results. Since we only focused on tumor suppressor genes (TSGs) among these driver genes, 20/20 rule was adopted to further filter out oncogenes 35. Briefly, we defined those genes containing >20% LOF mutations as potential tumor suppressors, which were used to construct subsequent TSG-target network.

### Network-Based Stratification

Based on the mutation profiles of driver genes, network-based stratification (NBS) was conducted to identify classifications of liver cancer 36. This algorithm is currently implemented in Python package called *pyNBS* (based on Python 2.7.2), which runs much faster than its original MATLAB-based version 37. The input data of NBS analysis include a high-quality cancer reference network (CRN) provided by the new NBS study 37 and a mutation matrix of driver genes. The resulting data contain the clustering information and corresponding consensus matrix. The number of clusters *k* was varied from 2 to 5. To evaluate the robustness of classifications, we further calculated the cophenetic correlation coefficient based on the resultant consensus matrix using the *NMF* R package. The *k* value that resulted in the maximum cophenetic correlation coefficient was considered as the optimal number of clusters. Through conducting nearest template prediction (NTP) analyses (Gene Pattern modules), we compared our results with previously published results, including classifications by Boyault *et al.* (G1-G6) 38, Chiang *et al.* (Five subclasses) 39, Hoshida *et al.* (S1-S3) 40, Désert *et al.* (Four subclasses) 41, and Yang *et al.* (C1-C3) 42.

### Collection of Drug Targets

Currently, not all human proteins are druggable and only less than 20% of them can be targeted using traditional small molecule agents 43. Therefore, to identify genes with potential therapeutic implications, we only focused on druggable targets (DTs) and used them to construct TSG-DT network. Target information was derived from two sources, namely the Drug Repurposing Hub 44 and DrugBank 45. The Drug Repurposing Hub contains 6125 compounds with corresponding 2249 target genes, while DrugBank compiles 5514 compounds and 2724 target genes. The target information in DrugBank was extracted from the fulldatabase file (XML format) using *XML* and *dbparser* packages. After removing duplication, a total of 3194 druggable genes were identified. Notably, for establishing the TSG-associated SL network, target genes whose mechanism of action (MOA) was denoted as an agonist were excluded.

### Mutual Exclusivity Analysis

The analysis was performed on the basis of 1825 patients with available genomic data using *discover* R package. Gene pairs with adjusted *P* < 0.15 or nominal *P* < 0.05 were considered significant. Mutual exclusivity-based SL network was visualized using Cytoscape software (version 3.7.1) 46. The annotation of TSG and target genes were also presented in the network graph.

### Functional Similarity Analysis

Functional similarity (FS) scores between gene pairs were calculated based on the semantic similarities in molecular function (MF) and cellular component (CC) aspect of the gene ontology (GO) terms, which can take both function and location of genes into account 47. The FS score for a gene pair is given as:


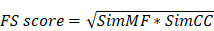


Semantic similarities in MF (SimMF) and CC (SimCC) were measured based on the GO topological structure through using *GOSemSim* package 48. Gene pairs with FS scores >0.5 were considered to have high functional interactions.

### Rank Aggregation Analysis

Rank aggregation analysis was conducted to obtain a robust ranking of resultant SL pairs based on the ranking results from multiple sources. An order statistics-based method proposed by Stuart *et al.* was utilized to perform this analysis 49. The output scores of this method were probabilities. We then defined the rank aggregation score (RAS) as follows:


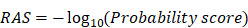


The resulting RAS was then used to determine a final ranking of candidate SL pairs. A higher RAS indicated a more concordant high ranking.

### Predicting Drug Sensitivity in Clinical Samples

Either CCLE or GDSC projects only measured the drug response data in less than 20 liver cancer cell lines, the limited number of which might diminish statistical power and interfere with meaningful conclusions of downstream analyses. Previous studies have demonstrated that drug response in clinical samples can be estimated using data from *in vitro* cell line experiments 50, 51. Therefore, we intended to perform drug response prediction based on the actual drug sensitivity and molecular data, which could to some extent tackle the problem of limited cell line number. Herein, the ridge regression model, which was considered an efficient and effective method in the previous report, was utilized to conduct transcriptome data-based drug response prediction 52. Based on the expression and drug response data of solid cell lines from CCLE and GDSC projects (excluding hematopoietic and lymphoid tissue-derived cell lines), this predictive model was trained with a satisfied predictive accuracy evaluated by default 10-fold cross-validation and applied to the clinical samples to achieve the estimated drug response values. The drugs were mapped to their targets for constructing the TSG-DT-drug network.

### Enrichment Analysis

To functionally describe the gene set of interest, we conducted the hypergeometric test using the *clusterProfiler* package based on the hallmark definitions (h.all.v7.0.symbols) downloaded from the Molecular Signatures Database (MSigDB) 53. The resulting *P* values from the hypergeometric tests were adjusted for multiple comparison testing and adjusted *P* < 0.05 were considered significant. The adjusted *P* values were then transformed to -log10(*P*) and visualized as bar plots.

### Human Cell Lines and Compounds

The human HCC cell lines, HEP3B217, HUH7, HEPG2, SNU398, SNU878, HUH6, SKHEP1 and PLCPRF5, were provided by Erasmus University (Rotterdam, Netherlands). MHCC97H were provided by the Liver Cancer Institute of Zhongshan Hospital (Shanghai, China). These cells were maintained in Dulbecco's modified Eagle's medium (DMEM) (Gibco, Carlsbad, CA) supplemented with 10% fetal bovine serum (FBS) (Gibco) and 1% penicillin/streptomycin (BasalMedia), incubated at 37 °C in humidified atmosphere with 5% CO2. Mycoplasma contamination was excluded via a PCR-based method. The information of *TP53* mutation status of cell lines used in this study was achieved from our previous publication 54. The identities of all the cell lines were confirmed by short tandem repeat (STR) profiling. Volasertib (S2235) and GSK461364 (S2193) were purchased from Selleck Chemicals and dissolved in dimethyl sulfoxide (DMSO) using a storage concentration of 10 mM.

### Cell Proliferation Assays

For long-term cell proliferation assay, cells were seeded into six-well plates (2-3×10^4^ cells per well) and agents or vehicle control was added after 24 hours. Cells were treated with agents as indicated for 10-14 days during which the culture media were replaced every three days. Afterwards, cells were stained with 1% crystal violet for 30 minutes and rinsed with tap water. Pictures were taken using ImageScanner^TM^ III (GE Healthcare) at 300 dpi resolution. In order to obtain the quantitative results of long-term cell proliferation assays, crystal violet was solubilized using 33% glacial acetic acid for 20 min and the absorbance was measured at 590 nm. For short-term assays, cells were seeded into 96-well plates (2-3×10^3^ cells per well) and were treated with agents for 72 hours. Then, cell viability was measured using CellTiter-Blue (CTB) assay (Promega) according to the manufacturer's recommendations. Experiments were performed in triplicate.

### Cell Apoptosis Assays

To visualize Caspase 3/7 activity, IncuCyte Caspase-3/7 green apoptosis assay reagent was added to the culture medium and images of phase and fluorescence were captured in IncuCyte ZOOM system (Essen Bioscience). Cell apoptosis was analyzed based on green fluorescent staining of apoptotic cells.

### Statistical Analysis

Unless stated otherwise, all the computational analyses and graphical visualization were performed in R statistical software (v.3.6.0, R Core Team, R Foundation for Statistical Computing, Vienna, Austria). Comparison of a continuous variable in two groups was performed using either parametric test (Student's t-test) if the variable was normally distributed or nonparametric test (Wilcoxon rank-sum test). Similarly, correlation between two continuous variables was measured by either Pearson's r correlation or Spearman's rank-order correlation. Contingency table variables were analyzed by Fisher's exact tests. The hazard ratio (HR) was estimated using a Cox regression model by R package *survival*. Survival curve was carried out using Kaplan-Meier methods and the log-rank (Mantel-Cox) test was used to determine the statistical significance of differences. The Benjamini-Hochberg method was applied for multiple testing correction. A two-tailed *P* < 0.05 was considered statistically significant unless indicated otherwise.

## Results

### The SiLi Pipeline

**Figure 1:**
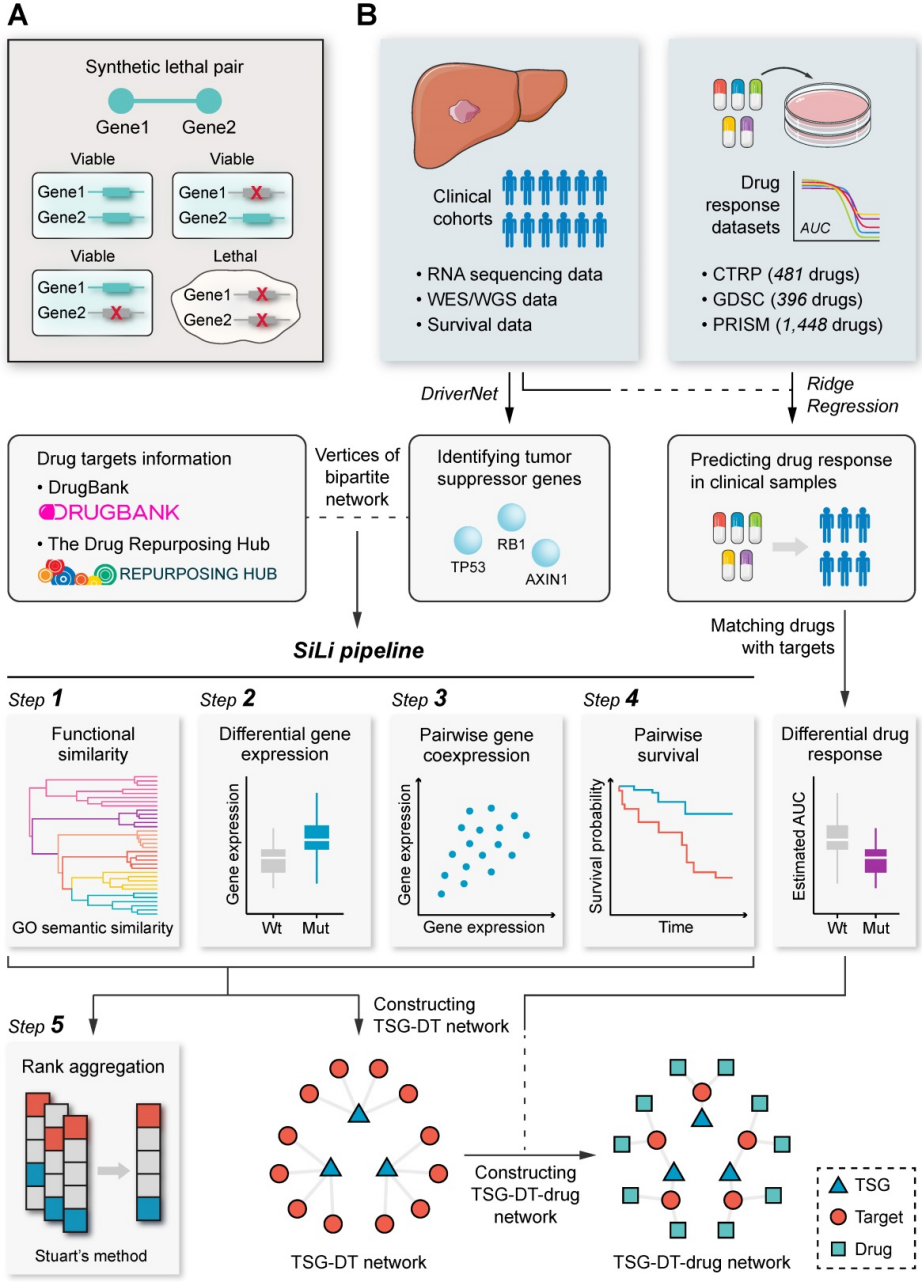
** Study overview.** (**A**) Schematic representation of the concept of synthetic lethality. (**B**) Flow chart of the SiLi pipeline developed for inferring synthetic lethal interaction in liver cancer.

SiLi is a computational pipeline for statistically inferring candidate SL interactions using high-throughput clinical data sets, drawing on experiences from several previous approaches such as DAISY 16, MiSL 17, ISLE 18, and DiscoverSL 19. This computational pipeline includes five inference steps: (1) functional similarity analysis, (2) differential gene expression analysis, (3) pairwise gene coexpression analysis, (4) pairwise survival analysis, and (5) rank aggregation analysis. The detailed procedures are described as follows:

- The strategy for functional similarity analysis is based on the notion that genes with SL interactions tend to engage in closely related biological processes and accordingly their location in the GO topological structure should be close (**Figure 1A**) 16. We defined FS score as the geometric mean of semantic similarities between MF and CC. Gene pairs with FS scores ≤ 0.5 were considered to have low functional similarity and were filtered out from the list of candidate SL pairs.
- The strategy for differential gene expression analysis is motivated by the assumption that if a certain gene loses its function due to mutation, tumors may increase the expression of its SL partners as a compensatory mechanism (**Figure 1A**). This hypothesis was adopted in multiple earlier studies to search for potential SL interactions 17, 55. We conducted differential expression analysis using Wilcoxon rank-sum test between samples with and without functional TSG mutations, and only genes with significantly higher expression in mutated samples were deemed potential SL partners of corresponding TSGs.
- The strategy for pairwise gene coexpression analysis is based on the analogous notion of functional similarity, that is, SL pairs tend to have a similar function and hence are more likely to be coexpressed (**Figure 1A**) 16. Gene pairs with significant correlation (Spearman's correlation coefficient > 0.15 and adjusted *P* < 0.05) were considered to be potential SL pairs.
- The strategy for pairwise survival analysis assumes that co-inactivation of paired genes would reduce tumor fitness and consequently patients with co-inactive SL pairs are more likely to exhibit significantly better outcomes than patients without co-inactivation (**Figure 1A**) 18, 56. Due to data limitations, we mainly used expression data to define inactive/active gene status, and a gene was considered to be inactive in a sample if its gene expression was below the median across all samples. Gene pairs that could lead to significantly better prognosis for patients with co-inactivation than patients without co-inactivation were retained for subsequent analysis (*P* < 0.05).
- Only gene pairs that passed all four procedures described above were taken as candidate SL pairs and utilized for constructing the TSG-DT network. Rank aggregation analysis using Stuart's method was then conducted to rank the resultant SL candidates 49.

A schematic diagram of the procedures of SiLi and the overall study design is presented in **Figure 1B**.

### A Brief Illustration and Validation of SiLi

**Figure 2:**
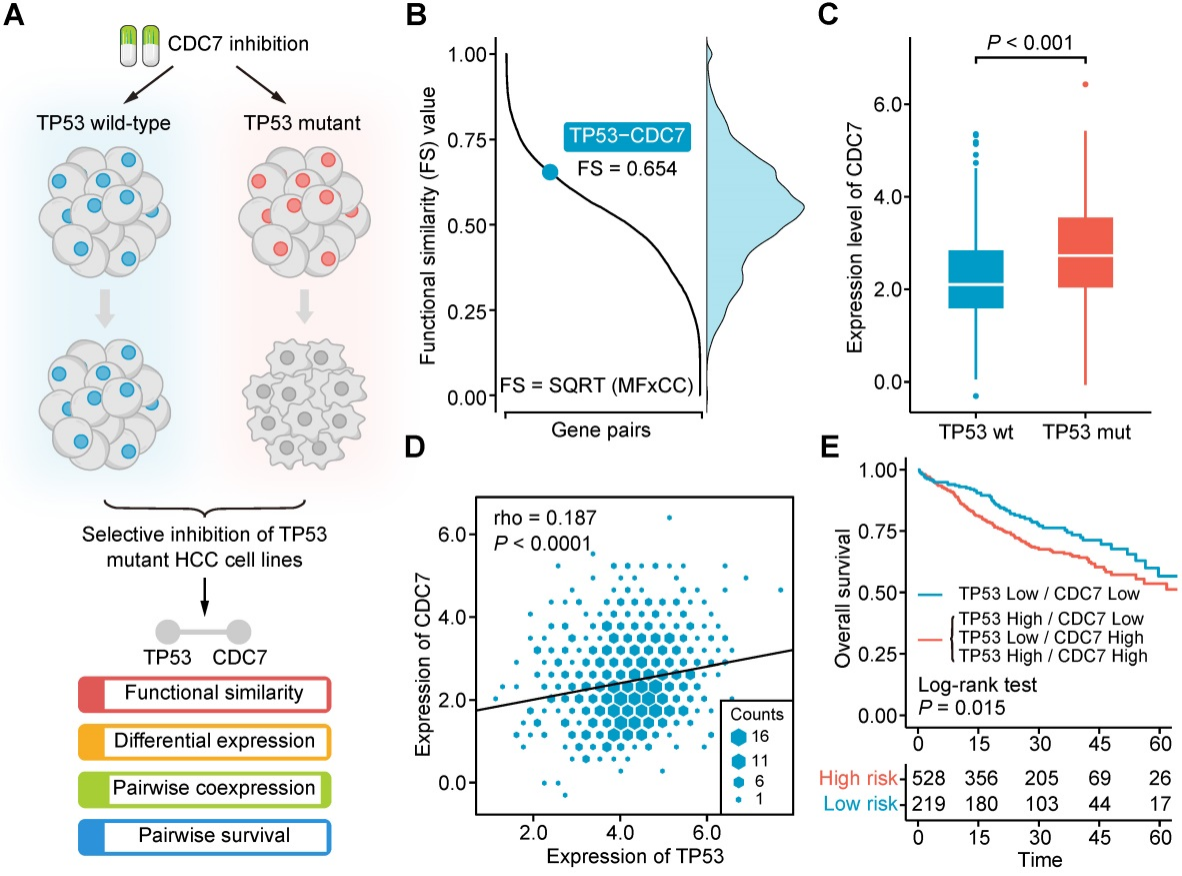
** Computational validation of the synthetic lethal interaction between TP53 and CDC7.** (**A**) Schematic diagram of the experimental finding of *TP53-CDC7* interaction. (**B**) The distribution of functional similarity scores among different gene pairs and the illustration of the functional similarity score of *TP53-CDC7* gene pair. (**C**) Differential gene expression of *CDC7* between *TP53*-mutant and *TP53*-wild-type samples. Statistical significance of expression difference was determined using Wilcoxon rank-sum test. (**D**) Correlation of the gene expression between *TP53* and *CDC7*. Spearman correlation coefficient is indicated on the top left of the plots. (**E**) Survival difference between the groups with and without co-inactivation of *TP53* and *CDC7*.

In our recent work, we found that CDC7 inhibitors could selectively suppress the growth and proliferation of *TP53*-mutant liver cancer cell lines, suggesting that an SL interaction may exist between *TP53* and *CDC7* in liver cancer (**Figure 2A**) 54. By exploiting this finding, we intend to briefly exemplify the practical application of SiLi as well as examine whether the theoretical assumptions used for the development of SiLi hold in this real SL interaction. Functional similarity analysis gave an FS score of 0.654 for the *TP53-CDC7* pair, higher than the threshold (0.5) we set (**Figure 2B**). Differential analysis of *CDC7* expression between *TP53*-mutant and *TP53* wild-type samples suggested a significant difference between the two groups, with higher *CDC7* expression in the *TP53*-mutant samples than the *TP53*-wild-type samples (*P* < 0.001) (**Figure 2C**). Coexpression analysis indicated that *TP53* and *CDC7* were significantly positively correlated (*P* < 0.0001), and the Spearman's correlation coefficient value (0.187) was also higher than our threshold (0.15) (**Figure 2D**). In the pairwise survival analysis, we observed that patients with co-inactivation of *TP53* and *CDC7* had significantly better survival outcomes than patients without co-inactivation (*P* = 0.015) (**Figure 2E**). Generally, these analysis results not only display the procedures of SiLi intuitively but also demonstrate the rationality of the SiLi pipeline design to some extent.

### Determination of Driver Genes in Liver Cancer

**Figure 3:**
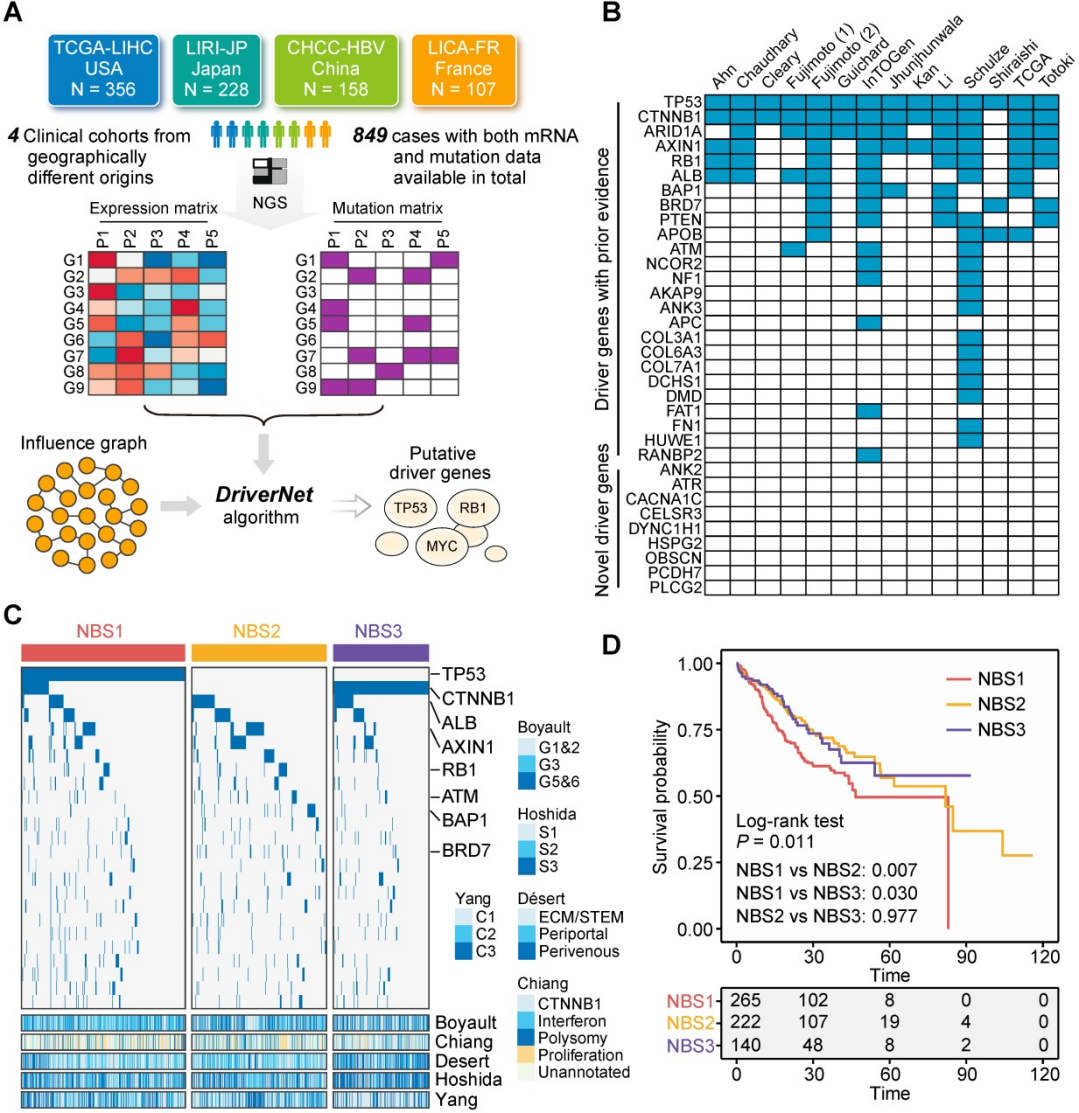
** Identification of driver genes in liver cancer.** (**A**) Summary of included clinical cohorts for *DriverNet* analysis. (**B**) Comparison between driver genes identified in this study and that reported by previous studies. (**C**) The mutation distribution of subclasses identified by network-based stratification. Statistical significance of difference was determined using Fisher's exact tests. Only statistically significant genes were labelled in the figure. Previously reported transcriptome-based molecular classifications were presented on the bottom of the plot. (**D**) Survival difference between three NBS subclasses. The statistical significance was determined by log-rank (Mantel-Cox) test.

Not all gene aberrations are related to tumorigenesis and tumor progression; some aberrations occur randomly, which are termed 'passengers' 35. Only a small number of aberrations, called 'drivers', possess the ability to confer selective advantages to tumor cells 35. Therefore, therapeutic strategies that target driver genes are more likely to have clinical significance. Currently, a plethora of algorithms based on various statistical principles have been developed to discern candidate driver genes 57. Of these, mutation frequency-based algorithms, such as *MuSiC* and *MutSigCV*, are the most commonly used approaches, and have been adopted by the majority of large-scale clinical studies focused on liver cancer 20, 23, 24. To identify candidate drivers, we applied a network-based method, *DriverNet*, to the currently most comprehensive metadata set of liver cancer, which includes 849 patients with liver cancer from four clinical cohorts with both expression and mutation data available (**Figure 3A**) 33. Batch effects were removed to ensure comparability between different cohorts (**Figure S1A**). This analysis yielded 34 genes with *q* < 0.05. Of these, 25 genes (73.5%) have been reported by at least one previous study as driver candidates, which demonstrates the reliability of our prediction (**Figure 3B** and**Table S1**). These 25 genes were considered robust drivers of liver cancer and used for subsequent analysis.

To demonstrate the clinical implications of these driver genes, we next classified their mutation profiles using the NBS algorithm 36. According to their cophenetic correlation coefficients, patients were assigned to three subclasses (**Figure S1B** and** S1C**). Each subclass had distinguishing mutation features. NBS1 and NBS2 exhibited a higher proportion of TSG mutations (the identification of TSGs is presented below), including *TP53*, *AXIN1*, *RB1*, *BAP1*, and *BRD7*, while NBS3 was characterized by a high mutation frequency of *CTNNB1* and low mutation frequency of TSGs (**Figure 3C**). Based on this result, NBS1 and NBS2 were defined as TSG-enriched subclasses. NBS classification was also associated with previously reported transcriptome-based classification. Taking the subclass NBS3 as an example, it was linked to Désert's perivenous (*P* < 0.001) 41 and Hoshida's S3 (*P* < 0.001) 40 subclasses. In addition, survival analysis indicated that there was a significant difference in survival outcome among the three NBS subclasses, and NBS1 exhibited a worse prognosis than either NBS2 (*P* = 0.007) or NBS3 (*P* = 0.030) (**Figure 3D**). In summary, the above results provide insight into driver gene-based clinical categorization of patients with liver cancer and hold the potential to guide further investigations of individualized therapies.

### Selection of Tumor Suppressors and Druggable Genes

Driver genes can be further classified into two classes: TSGs and oncogenes. Most alterations in TSGs cause loss of gene functions, while alterations in oncogenes tend to be gain-of-function mutations 35. The functional differences between these two driver gene classes could lead to distinct strategies for inferring their SL partners. Herein, motivated by the 20/20 rule, we defined those drivers containing more than 20% LOF mutations as potential TSGs 35. According to this principle, 14 of the 25 genes were deemed TSGs, which were then examined manually to guarantee a credible result (**Figure S2A**). We further investigated the mutation distribution of TSGs and oncogenes. The proportion of cases with at least one TSG mutation (70.67%) was higher than the proportion of cases with at least one oncogene mutation (48.41%), suggesting that therapeutics focused on TSGs might cover more patients with liver cancer than therapeutics focused on oncogenes (**Figure S2B**). However, most of current molecular targeted therapies exert their function through inhibiting a hyperactivated oncogene rather than restoring an inactivated TSG 6. Fortunately, SL offers an alternative means to indirectly target TSGs. Based on these considerations, we subsequently focused on interrogating SL partners of the 14 identified TSGs.

Another critical problem is that not all identified SL partners of TSGs can be druggable if SL inference is performed in a genome-wide level 43. Therefore, to ensure that all candidate genes can be targeted using conventional chemical agents, we compiled a list of 3194 druggable genes and limited the SL candidates to these genes. Accordingly, the SL network is a bipartite network, constructed on the basis of 14 TSGs and 3194 druggable genes.

### Inference of TSG-DT interactions

Based on the concept that simultaneous mutation of two genes in an SL pair influences cellular processes and causes cell death, mutual exclusivity analysis can be adopted to discover potential SL interactions 58-60. For conducting this analysis, we collected seven clinical cohorts with available WES/WGS data, comprising 1825 patients with liver cancer (**Figure S3A**). With the threshold for significance set to adjusted *P* < 0.15, only 18 mutually exclusive gene pairs were identified. If we further limited these gene pairs to TSG-DT interactions, only 4 gene pairs were left (**Figure S3B**). To identify more mutually exclusive pairs, we relaxed the threshold to nominal *P* < 0.05. Under this condition, a total of 325 mutually exclusive pairs and 67 TSG-DT pairs were identified (**Figure S3C**), which we believe is still limited for further screening. Although mutual exclusivity analysis has been adopted by multiple studies to infer candidate SL interactions, this analysis might not be a suitable option in liver cancer, given the relatively sparse mutation profiles of this cancer type 19, 59, 61. In summary, these results support the rationality of excluding mutual exclusivity analysis from the SiLi pipeline.

**Figure 4:**
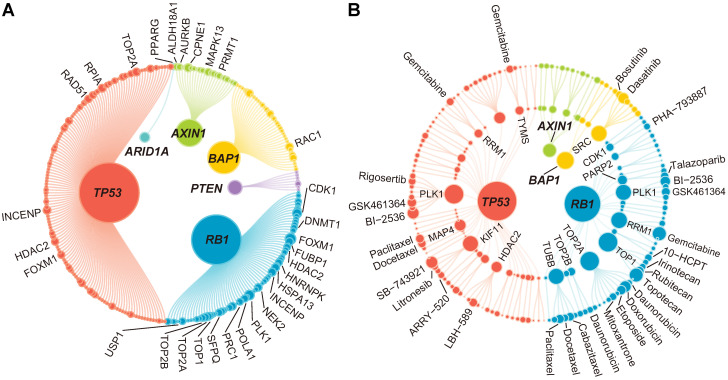
** The results of the identification of SL interactions.** (**A**) A bipartite network of 272 TSG-DT interactions. The node size is proportional to the node degree. Only top 30 TSG-DT pairs with highest RAS scores are marked on the plot. The detailed information is presented in Table S2. (**B**) A bipartite network of 165 TSG-DT-drug interactions. For nodes in the inner layers, the node size is proportional to the node degree. For nodes in the outer layer, the node size is proportional to the fold change values of differential response analysis. Only top 30 TSG-DT-drug pairs with highest fold change values are marked on the plot. The detailed information is presented in Table S2.

Taking the 14 TSGs and 3194 druggable genes identified above as the basis, the first four steps of SiLi, including functional similarity analysis, differential gene expression analysis, pairwise gene coexpression analysis, and pairwise survival analysis, were conducted to infer TSG-DT pairs with potential SL interactions. A total of 272 TSG-DT pairs (including 209 unique targets) passed the four screening steps and were considered SL candidates for liver cancer (**Table S2**). These SL candidates are visualized in a bipartite network graph in **Figure 4A**.

Subsequently, in order to obtain a ranking of the 272 TSG-DT pairs, rank aggregation analysis was performed to integrate the SiLi results. Briefly, the TSG-DT pairs were firstly ranked based on their FS scores (functional similarity), fold change values (differential expression), correlation coefficients (pairwise coexpression), and hazard ratios (pairwise survival). Then, Stuart's method was applied to integrate all these rankings and calculate the RAS of each TSG-DT pair (**Figure S4A** and**Table S3**) 49. We considered that TSG-DT pairs with high RASs were more likely to have SL interactions.

The SL candidates in this study were compared to those inferred in other studies to evaluate our prediction results. Due to the deficiency of studies focused on investigating liver cancer-specific SL pairs, we compared our results with those from pan-cancer or other tumor-associated studies, including studies by Wang *et al.* 55, Kranthi *et al.* 62, Ye *et al.* 59, and Jerby-Arnon *et al.* 16. The comparison results suggest that the overall consistency between different predictions is quite poor. Only 3.68% (10 pairs) of our prediction overlapped with other results (**Figure S4B**). We suppose that the different inference procedures, as well as input data, might be the major contributors of the discrepancy.

### Extension of TSG-DT interactions to TSG-DT-drug interactions

By leveraging pharmacogenomic data from CTRP 29, GDSC 28, and PRISM 30 datasets and integrating drug information into the TSG-DT network, we intended to further construct a TSG-DT-drug network, which might possess relatively direct and practical clinical implications. Regardless of duplication, a total of 1986 unique drugs were included in this study. Estimated drug response in clinical samples could be obtained by constructing ridge regression model based on actual drug sensitivity and expression data from cell lines. The reliability of this approach has been validated computationally and experimentally in our previous publication 51. Next, according to target annotation, 1986 drugs were mapped to 209 unique targets in the TSG-DT network, which retained 381 DT-associated drugs. To further connect TSGs with these DT-associated drugs, differential drug response analysis was conducted between samples with and without TSG mutations. Only drugs with significantly lower estimated AUC values in mutated samples were considered to be SL-associated drugs. This analysis yielded 165 TSG-drug pairs (without removing duplication from different datasets) and 62 TSG-DT pairs (**Table S2**). These pairs are visualized in a TSG-DT-drug network graph in **Figure 4B**. Considering that the 1986 drugs could only cover 96 of 209 (45.9%) targets inferred by SiLi, this step was taken as an extension rather than a further screening to avoid potential bias.

### Characterization of Target Genes in the TSG-DT Network

**Figure 5:**
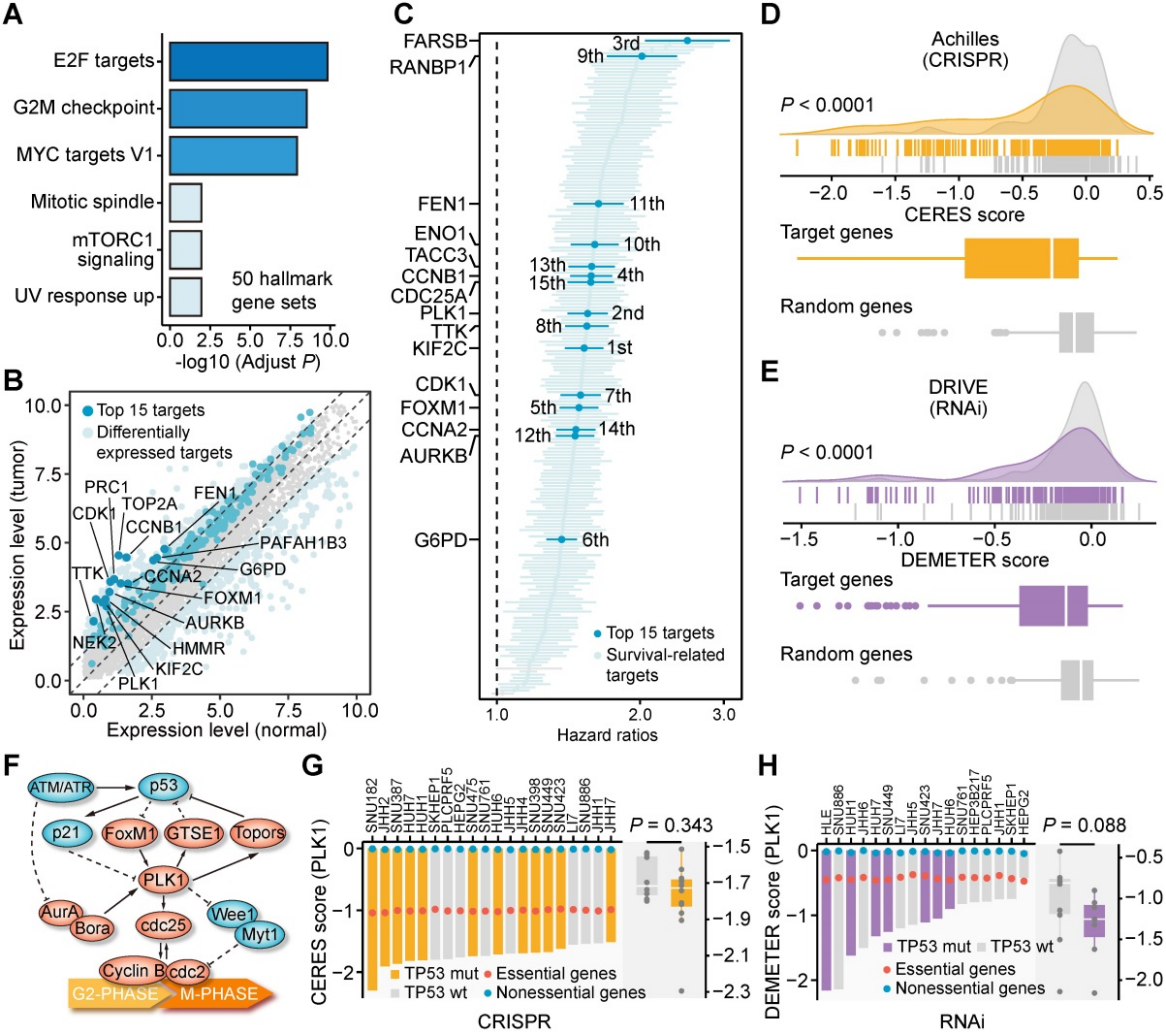
** Clinical and biological characteristics of resulting 209 unique targets.** (**A**) The results of enrichment analysis based on 209 target genes using 50 hallmark gene sets. (**B**) Differential expressed genes between tumor tissues and normal tissues. The 209 targets were marked on the plot. (**C**) The survival association of 209 target genes determined by cox proportional hazards regression analysis. (**D**) The difference of CRISPR-based gene dependency scores between target set and random set. (**E**) The difference of RNAi-based gene dependency scores between target set and random set. Statistical significance of expression difference was determined using Wilcoxon rank-sum test. (**F**) Schematic plot of the biological relationship between *TP53* and *PLK1*. (**G**) The CRISPR-based gene dependency scores of *PLK1* across 20 liver cancer cell lines. (**H**) The RNAi-based dependency scores of *PLK1* across 17 liver cancer cell lines.

As mentioned above, there are 209 unique target genes in the 272 TSG-DT pairs. To evaluate the clinical and biological significance of these genes, we carried out comprehensive analyses using clinical data from the metadata set and gene dependency data from CRISPR and RNAi screens. The biological processes related to the 209 targets were first characterized by enrichment analysis. Most of the significantly enriched processes, such as E2F targets, G2M checkpoint, and MYC targets V1, were associated with cell proliferation, and were generally consistent with the function of their SL partners (**Figure 5A**). Next, the expression differences of these genes between tumor and normal tissues were investigated. Differential genes were identified by adjusted *P* < 0.01 and |log_2_(fold change)| > 1. As expected, all the differential target genes exhibited higher expression levels in tumor tissues than normal tissues (**Figure 5B** and**Table S4**). Cox proportional hazards regression analysis was also performed to reveal their association with survival outcome. The result suggests that all the significant genes (207 of 209) are associated with unfavorable prognosis (hazard ratio > 1) (**Figure 5C** and**Table S5**). These findings demonstrate that the clinical phenotypes of these 209 genes are compatible with their roles as potential targets for inhibitors.

Furthermore, the dependency of the target genes across liver cancer cell lines was also explored. We first generated a random gene set of the same size (209) and then compared the dependency scores of the target set and the random set. The target set had significantly lower dependency scores than the random set using either CRISPR-based data (**Figure 5D**) or RNAi-based data for comparison (**Figure 5E**), which means that liver cancer cell lines are more likely to depend on these 209 genes for their survival and growth. The dependency rankings of the 209 genes were also calculated (**Figure S5A** and** S5B**,**Table S6**). Overall, the above analysis results systematically depict the characteristics of the 209 genes and preliminarily unveil their potential as novel therapeutic targets for treating liver cancer.

### Identification of Novel Therapeutics for *TP53*-mutant Liver Cancer

The rankings of the 209 target genes varied greatly when different analysis results were used for ranking. Among the targets, we noticed that polo-like kinase 1 (*PLK1*) was a relatively top-ranked target in multiple analyses (differential expression analysis: 11^th^; survival analysis: 2^nd^; CRISPR-based dependency: 14^th^; RNAi-based dependency: 7^th^). Also, two* PLK1*-engaged gene pairs, *RB1-PLK1* and *TP53-PLK1*, are highly ranked among the 272 TSG-DT pairs (*RB1-PLK1*: 4^th^; *TP53-PLK1*: 47^th^) (**Figure S4A**). Accordingly, the target *PLK1* was selected for further validation of its therapeutic potential in liver cancer. Given that the low mutation frequency of *RB1* (4.83%) (**Figure S2B**) is neither conducive to subsequent experimental validation (Hep3B is the only *RB1*-mutated liver cancer cell line recorded in CCLE) nor favorable for its potential clinical application, we selected the *TP53-PLK1* for subsequent validation.

**Figure 6:**
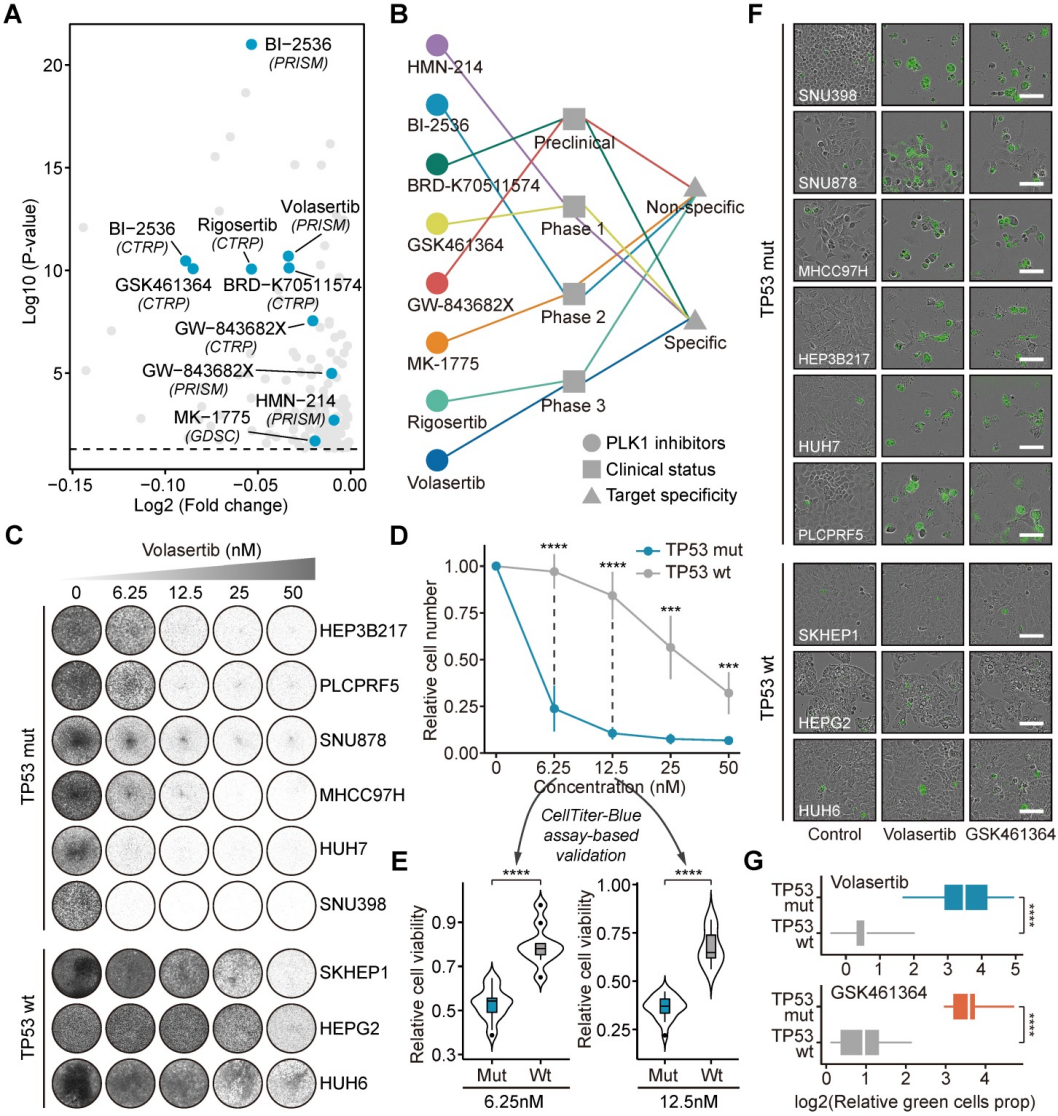
** Investigations of the roles of PLK1 inhibition as a novel therapeutic strategy of liver cancer.** (**A**) The results of differential drug response analyses. The PLK1 inhibitors with significant response differences between *TP53*-mutant and wild-type groups were labeled on the plot. (**B**) The information of clinical status and target specificity of included PLK1 inhibitors. (**C**) Long-term cell proliferation assays were conducted based on six *TP53*-mutant cell lines (SNU398, SNU878, MHCC97H, HEP3B217, HUH7, and PLCPRF5) and three *TP53*-wild-type cell lines (SKHEP1, HEPG2, and HUH6) treated with volasertib using gradient concentrations (6.25nM, 12.5nM, 25nM, and 50nM). (**D**) Quantitative results of long-term cell proliferation assays of volasertib. The point represents the mean value and the error bar indicates the standard deviation. (**E**) Comparison of CellTiter-Blue assay-based cell viability between *TP53*-mutant and *TP53*-wild-type cell lines treated with volasertib using two different concentrations (6.25nM and 12.5nM). (**F**) Representative Incucyte images of caspase-3/7 green assays of liver cancer cell lines treated with volasertib and GSK461364 (scale bars: 100 µm). Based on the results from cell proliferation assays, 12.5nM was chosen for volasertib treatment while 6.25nM was chosen for GSK461364 treatment. (**G**) Quantitative comparison of caspase-3/7 green assays between *TP53*-mutant and *TP53*-wild-type cell lines. Statistical significance of difference was determined using Student t-test (***P < 0.001, ****P < 0.0001).

*TP53* and *PLK1* collectively participate in the cell cycle process (**Figure 5F**) 63. To delineate the relationship between *TP53* and *PLK1* in liver cancer, the dependency scores of *PLK1* across *TP53*-mutant and *TP53*-wild-type liver cancer cell lines were analyzed (**Figure 5G** and **5H)**. Cell lines with *TP53* mutations showed a trend toward lower *PLK1* dependency scores, albeit not statistically significant. Many small molecule inhibitors targeting PLK1 are currently available. The results of *in silico* analysis suggested that *TP53* mutation could lead to higher sensitivity of tumors to treatment with multiple PLK1 inhibitors (**Figure 6A**). The current clinical status and target specificity of these PLK1 inhibitors were presented in **Figure 6B** 44. Among them, volasertib was selected for further experimental validation for its late-phase clinical status and high target specificity. Long-term cell proliferation assays were first performed using nine liver cancer cell lines (**Figure 6C**). The quantitative results showed that, under the condition of different treatment concentrations, volasertib all exhibited a stronger antitumor activity on *TP53*-mutant cell lines than on *TP53*-wild-type cell lines (**Figure 6D**). We further conducted short-term CTB-based viability assays to validate this result (**Figure S6**). It could be observed that the cell viability of *TP53*-mutant cell lines were significantly lower than that of *TP53*-wild-type cell lines, consistent with the results from long-term assays (**Figure 6E**). Aside from volasertib, another PLK1 inhibitor named GSK461364 was also used to perform additional validation. Similarly, the treatments of GSK461364 had preferential growth inhibitory activity on *TP53*-mutant cells than *TP53*-wild-type cells as well, which further demonstrated the reliability of our findings (**Figure S7A-C** and **Figure S8**).

Concurrently, to explore the effects of PLK1 inhibitors on the apoptosis pathway, we performed caspase 3/7 assays, in which apoptosis activation is proportional to the intensity of green fluorescence (**Figure 6F**). *TP53*-mutant cell lines exhibited stronger green fluorescence than *TP53*-wild-type cell lines, indicating that PLK1 inhibitors might have a stronger ability to induce apoptosis upon *TP53* mutation (**Figure S9 and Figure 6G**). Overall, the consistent results from *in silico* prediction and *in vitro* experiments demonstrate the potential of targeting PLK1 for treating liver cancer.

## Discussion

Current therapeutic strategies against liver cancer still follow the principle of “one size fits all” with low overall response rates 3. Due to the absence of FDA-approved agents that can specifically target genetic aberrations, individualized treatment of liver cancer has lagged far behind other cancers 3. Recently, the selective FGFR4 inhibitor fisogatinib has presented some promising results in phase I/II clinical trials and brought new therapeutic opportunities to liver cancer 64. However, considering that the target population of fisogatinib is limited to patients with either genomic amplification or upregulated expression of *FGF19*, this therapy can only cover <30% of all liver cancer cases 64. According to our finding, >70% of liver cancer patients harbored at least one TSG mutation. Nevertheless, effective therapies targeting patients with TSG mutations remain largely unexplored 3. Aiming to fill this knowledge gap, we proposed the SiLi pipeline and thereby determined 272 TSG-DT pairs with potential SL interactions in liver cancer. Further, we extended these TSG-DT interactions to TSG-DT-drug interactions that had more direct clinical significance. Theoretically, the vast majority of patients with liver cancer can be covered by SL-based therapeutic strategies and our findings could provide novel insights into personalized liver cancer treatment.

There are two main types of computational approaches to inferring SL interactions: statistical approaches and machine learning approaches 65. Statistical approaches, such as the previously reported DAISY 16 and ISLE 18 and our SiLi, use biological hypothesis-based statistical tests to predict SL pairs, which are relatively convenient and flexible. In comparison, machine learning approaches require prior knowledge of SL interactions for training prediction models. Taking DiscoverSL as an example, this approach applies a random forest classifier to determine whether SL pairs of interest are meaningful 19. Before the prediction, the random forest model needs first to be trained on validated positive/negative SL pairs using statistical tests-based features as independent variables. New SL interactions can then be predicted based on the trained random forest model 19. Evidently, the development of machine learning approaches relies on a sufficient number of previously validated SL pairs. In the absence of training data, adopting statistical approaches should be a preferable option.

The specific procedures adopted by various statistical approaches also vary substantially. Many established approaches integrate analyses of functional genetic screening data into their pipelines 16, 60. This procedure was not adopted in SiLi since the insufficient number of cell lines with available gene dependency data could limit the statistical power and thus affect the final prediction results. Another common inference procedure adopted by many statistical approaches is mutual exclusivity analysis 19, 59, 61. This procedure, according to our findings, is also not applicable to liver cancer due to the sparsity of the mutation profiles. In general, among the numerous approaches developed to date, SiLi is the only one tailored specifically to liver cancer data. Notably, SiLi can also be applied to other cancers that have similar data characteristics as liver cancer.

Among the 272 identified TSG-DT gene pairs, *TP53-PLK1* exhibits several advantageous biological and clinical properties. Thus, it was selected to be further investigated for its therapeutic implication. The relationship between p53 and PLK1 has been described in a previous study 63. Specifically, both p53 and PLK1 engage in the cell cycle process (Figure 5F). The roles of p53 that induce cell cycle arrest rival the functions of PLK1 that promote cell cycle progression 63. Expression of *PLK1* is indirectly repressed by p53 through multiple processes, and suppression of *PLK1* through p53 is a crucial mechanism that cells use to prevent abnormal overcoming of cell cycle arrest 63. Mutation or loss of *TP53* can lead to upregulation of *PLK1* expression, thereby promoting a hyperproliferative phenotype 63. Among patients with primary breast cancer, patients with *TP53*-mutant tumors expressing *PLK1* have been found to exhibit a poorer survival outcome than patients having either *PLK1* expression or *TP53* mutation alone 66. Since intact p53 may reduce the sensitivity of tumor cells to selective PLK1 inhibition by suppressing the basal level of PLK1, cells with *TP53* mutations may have a greater susceptibility to PLK1 inhibition, which was reported in a prior study 67. Although a very recent study presented a preliminary finding that *TP53*-mutant Huh7 cells were more sensitive to PLK1 inhibitors than *TP53*-wild-type HepG2 cells, systematic analyses have not yet been conducted to characterize *PLK1* as a specific target and PLK1 inhibitors as selective therapeutics of *TP53*-mutant liver cancer 68. In this study, utilizing public data from clinical cohorts and functional genetic screens, we comprehensively investigated the potential therapeutic role of *PLK1* in liver cancer. The significant clinical implications and the high cell dependencies both suggest that PLK1 can be a promising therapeutic target in liver cancer. Comparisons of the estimated drug response of *TP53*-mutant and *TP53*-wild-type samples to several PLK1 inhibitors showed that PLK1 inhibitors tended to have lower estimated AUC values (higher sensitivity) in the *TP53*-mutant group than those in the *TP53*-wild-type group. Considering that the estimated drug response cannot represent the actual case, we further conducted *in vitro* experiments across nine liver cancer cell lines to validate the *in silico* results. As expected, the experimental results showed a good agreement with the computational results, collectively demonstrating that inhibition of PLK1 has the potential to be a novel and selective anti-liver cancer strategy.

This study has several limitations. First, the identification of liver cancer-specific SL interactions relied on only three types of data, namely expression data, SNVs/indels-based mutation data, and survival data, without the involvement of CNVs profiles due to the data limitation. Since CNVs are also closely associated with the gain/loss of function of corresponding genes, the exclusion of CNV data might affect the accuracy of the SL prediction. Generally, currently available resources are insufficient for carrying out multiple omics-based analyses in a large-scale metadata set of liver cancer. With more and more high-throughput sequencing data becoming available, the results of this work should be validated and extended in an appropriate large-scale metadata set in the future. Second, an insufficient number of liver cancer-specific SL interactions with experimental evidence limited our capacity to assess the accuracy of the SL prediction. Comparisons between our results and several previous non-specific predictions presented poor concordance, suggesting that further validation would be necessary to ensure the reliability of our results. Finally, we only conducted *in vitro* experiments to investigate the therapeutic potential of PLK1 inhibitors. Performing more comprehensive *in vivo* validations using cell line derived xenograft or patient derived xenograft tumor models might give more convincing results. These limitations notwithstanding, this study has several strengths, including the individual data pooled study design that contributed a large number of patients with liver cancer. Owing to the large sample size, we were able to explore the SL partners of TSGs with low mutation frequency.

Overall, the present study proposed a new approach for SL identification and presents the most comprehensive landscape of TSG-associated SL interactions in liver cancer. Additionally, a novel therapeutic strategy targeting *PLK1* was also demonstrated to have significant potential in the field of personalized liver cancer treatment.

## Abbreviations

AUC: area under the dose-response curve
CC: cellular component
CCLE: cancer cell line encyclopedia
CNVs: copy number variants
CRISPR: clustered regularly interspaced short palindromic repeats
CRN: cancer reference network
CTRP: cancer therapeutics response portal
DepMap: Dependency Map
DTs: druggable targets
FDA: United States Food and Drug Administration
FPKM: fragments per kilobase per million reads
FS: functional similarity
HR: hazard ratio
Indels: insertions/deletions
GDSC: genomics of drug sensitivity in cancer
GO: gene ontology
LOF: loss-of-function
MF: molecular function
MOA: mechanism of action
NBS: network-based stratification
NTP: nearest template prediction
RAS: rank aggregation score
RNAi: RNA interference
RNAseq: RNA sequencing
SiLi: statistical inference-based synthetic lethality identification
SL: synthetic lethality
SNVs: single nucleotide variants
TCGA-CDR: TCGA Pan-Cancer Clinical Data Resource
TPM: transcripts per kilobase million
TSGs: tumor suppressor genes
WGS: whole genome sequencing
WES: whole exome sequencing
