# Supplementary material for: Mapping the landscape of synthetic lethal interactions in liver cancer
Source: Theranostics. 2021 Aug 26;11(18):9038–53. doi: 10.7150/thno.63416 (PMC8419043; doi:10.7150/thno.63416)
Supplement: Supplementary file 1 — Supplementary figures and tables. [file thnov11p9038s1.pdf]

Supplementary Information

Yang et al. "Mapping the landscape of synthetic lethal interactions in liver cancer"

The PDF file includes:

Supplementary Figure 1. Removing batch effect and classification of clinical samples based on mutation data.

Supplementary Figure 2. Identification of tumor suppressor genes.

Supplementary Figure 3. Mutation exclusivity analysis.

Supplementary Figure 4. Candidate synthetic lethality (SL) interactions based on SiLi prediction.

Supplementary Figure 5. The gene dependency of candidate targets in liver cancer.

Supplementary Figure 6. The results of CellTiter-Blue (CTB) cell viability assays of cells treated with volasertib.

Supplementary Figure 7. Drug sensitivity of *TP53*-mutant and *TP53*-wild-type cell lines treated with GSK461364.

Supplementary Figure 8. The results of CellTiter-Blue (CTB) cell viability assays of cells treated with GSK461364.

Supplementary Figure 9. The results of caspase-3/7 green assays of cells treated with volasertib and GSK461364.

Supplemental Table 1. Detailed information of previous studies providing driver gene lists.

Supplemental Table 2. TSG-DT and TSG-DT-drug pairs.

Supplemental Table 3. Ranking of 272 TSG-DT pairs.

Supplemental Table 4. Results of differential expression analysis.

Supplemental Table 5. Results of cox regression analysis.

Supplemental Table 6. Gene dependency of target genes.

Supplementary Figures

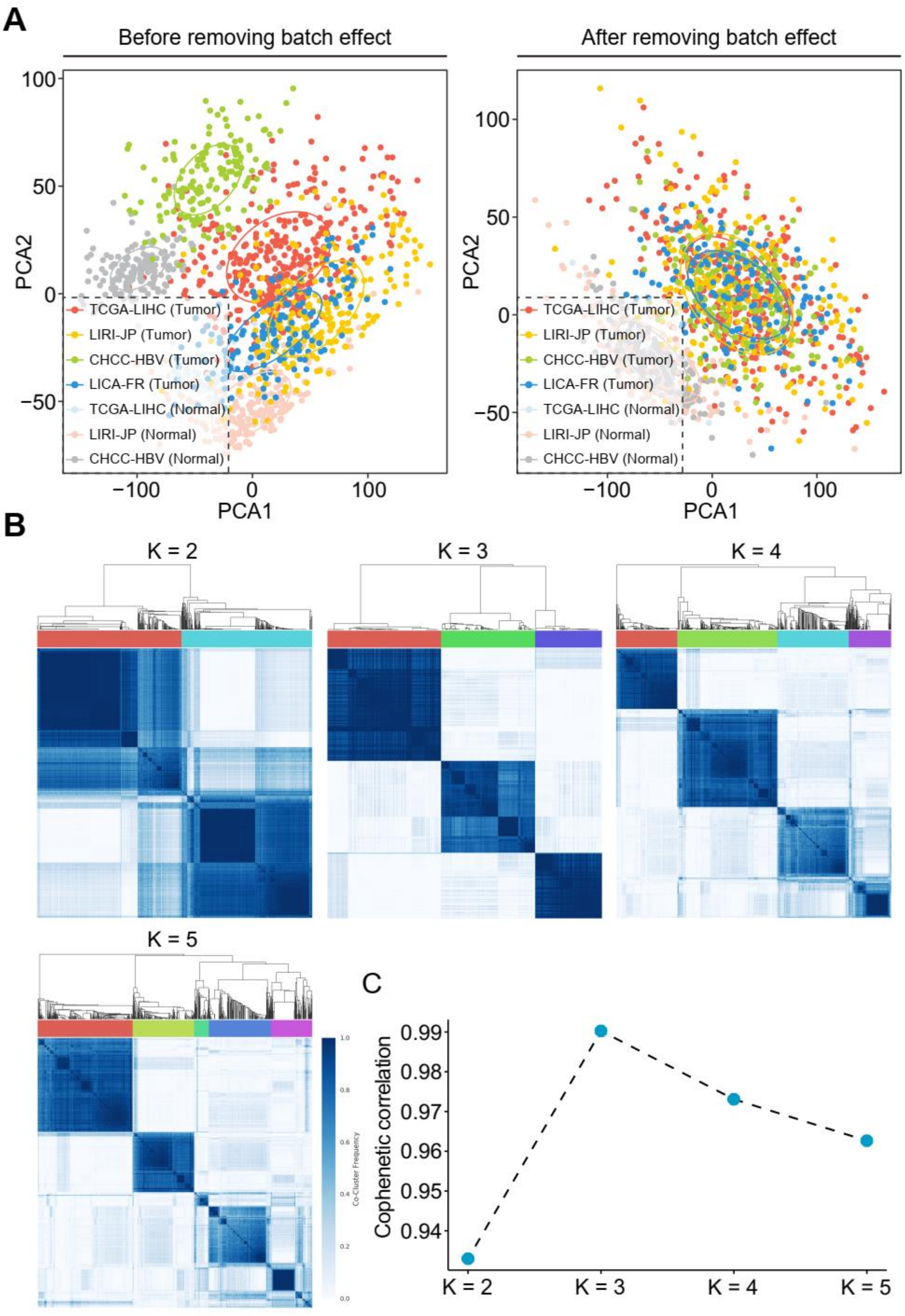

Supplementary Figure 1. (A) Removing batch effect. Scatter plot of principal component analysis (PCA) of four clinical cohorts (including TCGA-LIHC, LIRI-JP, CHCC-HBV, and LICA-FR) before (upper) and after (lower) batch effect correction. (B) Consensus matrix of network-based stratification (NBS) based on mutation data for k=2-5. (C) Cophenetic correlation coefficient under corresponding k values.

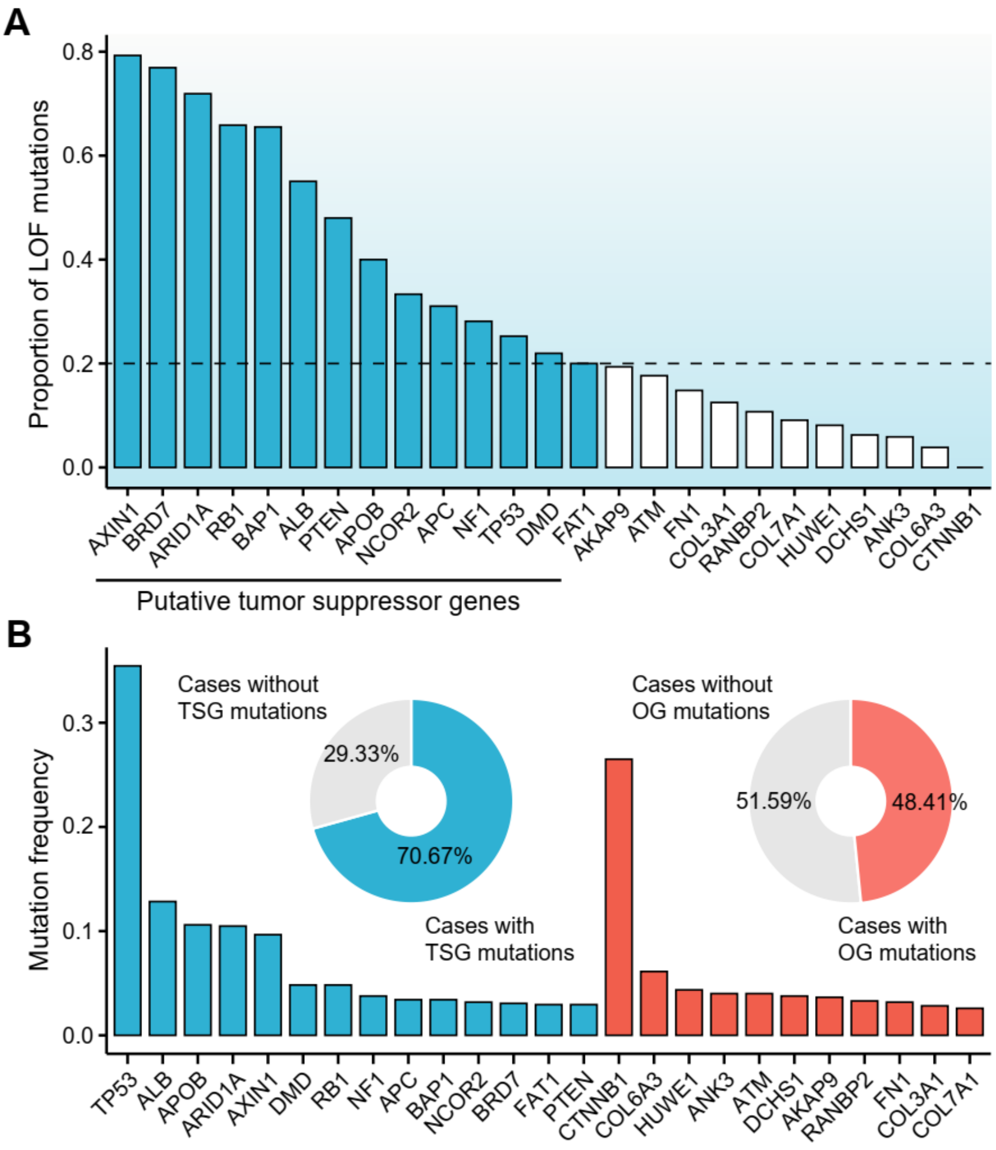

Supplementary Figure 2. Identification of tumor suppressor genes. (A) The proportion of loss-of-function (LOF) mutations of each driver gene. Genes with > 20% LOF mutations were considered as candidate tumor suppressor genes (TSGs). (B) Mutation frequency (bar chart) and the proportion of liver cancer cases (pie charts) with at least one TSG (left) or oncogene (OG) (right) mutation.

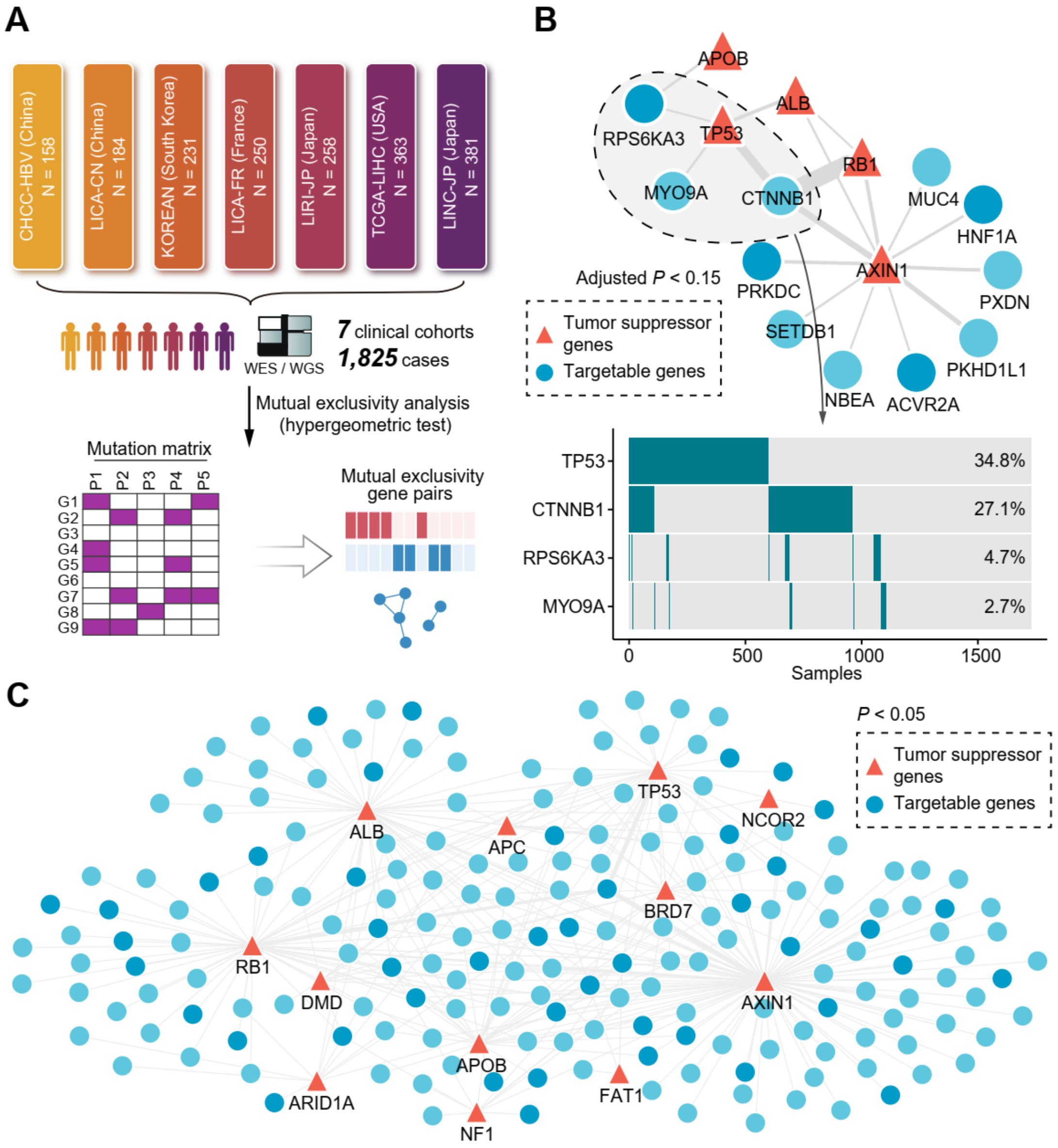

Supplementary Figure 3. Mutation exclusivity analysis. (A) Summary of included clinical cohorts for mutation exclusivity analysis. (B) Illustration of potential TSG-target interactions. Only interactions with adjust $P < 0.15$ were included. (C) Network of TSG-target interactions based on a relaxed significance threshold of $P < 0.05$.

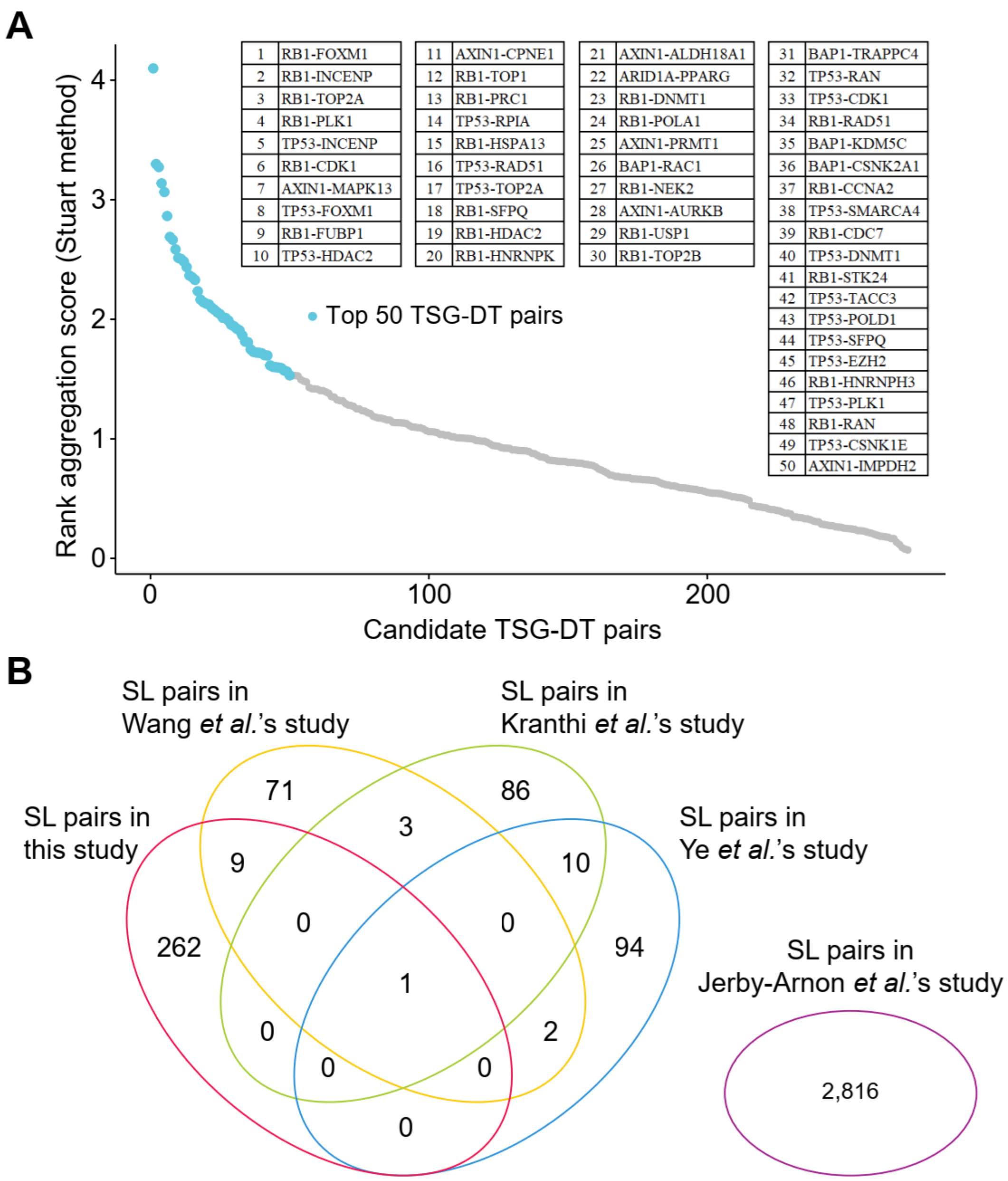

Supplementary Figure 4. Candidate synthetic lethality (SL) interactions based on SiLi prediction. (A) The ranking results of 272 candidate TSG-target pairs based on Stuart method. Top 50 pairs were listed in the table. (B) Comparison of the 272 SL pairs in the present study with SL pairs in other studies, including Wang et al.'s study [1], Kranthi et al.'s study [2], Ye et al.'s study [3], and Jerby-Arnon et al.'s study [4].

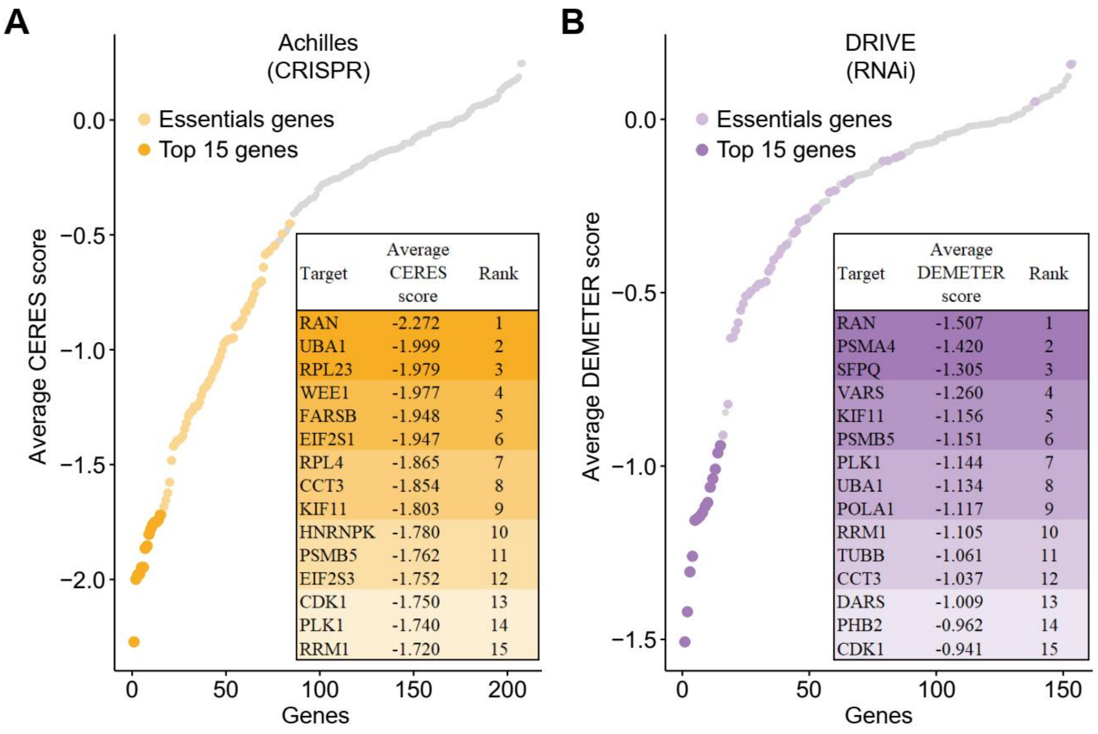

Target	Average CERES score	Rank
RAN	-2.272	1
UBA1	-1.999	2
RPL23	-1.979	3
WEE1	-1.977	4
FARSB	-1.948	5
EIF2S1	-1.947	6
RPL4	-1.865	7
CCT3	-1.854	8
KIF11	-1.803	9
HNRNPK	-1.780	10
PSMB5	-1.762	11
EIF2S3	-1.752	12
CDK1	-1.750	13
PLK1	-1.740	14
RRM1	-1.720	15

Target	Average DEMETER score	Rank
RAN	-1.507	1
PSMA4	-1.420	2
SFPQ	-1.305	3
VARS	-1.260	4
KIF11	-1.156	5
PSMB5	-1.151	6
PLK1	-1.144	7
UBA1	-1.134	8
POLA1	-1.117	9
RRM1	-1.105	10
TUBB	-1.061	11
CCT3	-1.037	12
DARS	-1.009	13
PHB2	-0.962	14
CDK1	-0.941	15

Supplementary Figure 5. The gene dependency of candidate targets in liver cancer. (A) Distribution of average CERES score (from CRISPR screens) of 209 candidate targets. (B) Distribution of average DEMETER score (from RNAi screens) of candidate targets. Note that a lower CERES or DEMETER score of a certain gene indicates a higher likelihood that this gene is essential in cell growth and survival. Top 15 targets were presented in corresponding tables.

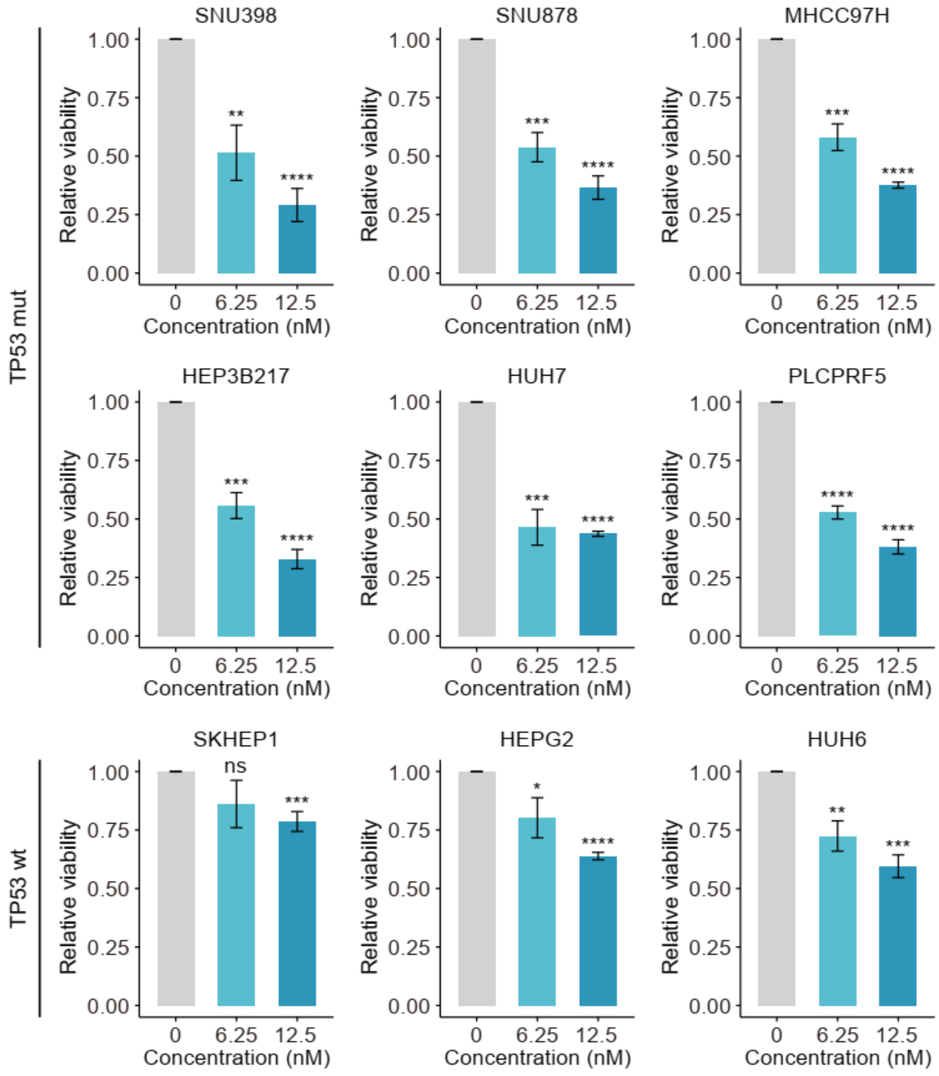

Supplementary Figure 6. The results of CellTiter-Blue (CTB) cell viability assays of cells treated with volasertib. A total of nine liver cancer cell lines, including six *TP53*-mutant cell lines (SNU398, SNU878, MHCC97H, HEP3B217, HUH7, and PLCPRF5) and three *TP53*-wild-type cell lines (SKHEP1, HEPG2, and HUH6) were treated with volasertib (6.25nM and 12.5nM) and the cell viability was measured by CTB assays. Statistical significance of difference was determined using Student t-test (ns represents no significance, *$P < 0.05$, **$P < 0.01$, ***$P < 0.001$, ****$P < 0.0001$).

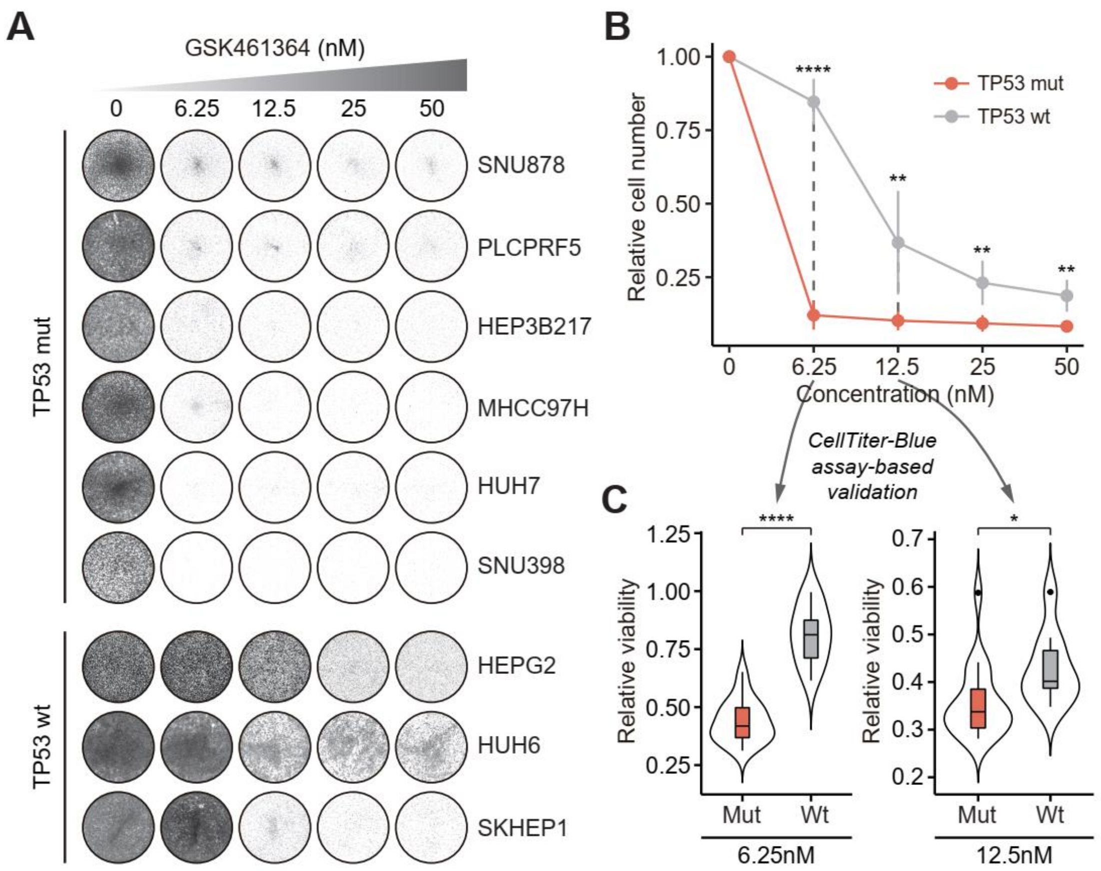

Supplementary Figure 7. Drug sensitivity of *TP53*-mutant and *TP53*-wild-type cell lines treated with GSK461364. (A) Long-term cell proliferation assays were conducted based on six *TP53*-mutant cell lines (SNU398, SNU878, MHCC97H, HEP3B217, HUH7, and PLCPRF5) and three *TP53*-wild-type cell lines (SKHEP1, HEPG2, and HUH6) treated with GSK461364 using gradient concentrations (6.25nM, 12.5nM, 25nM, and 50nM). (B) Quantitative results of long-term cell proliferation assays of GSK461364. The point represents the mean value and the error bar indicates the standard deviation. (C) Comparison of CellTiter-Blue assay-based cell viability between *TP53*-mutant and *TP53*-wild-type cell lines treated with GSK461364 using two different concentrations (6.25nM and 12.5nM). Statistical significance of difference was determined using Student t-test (*$P < 0.05$, **$P < 0.01$, ****$P < 0.0001$).

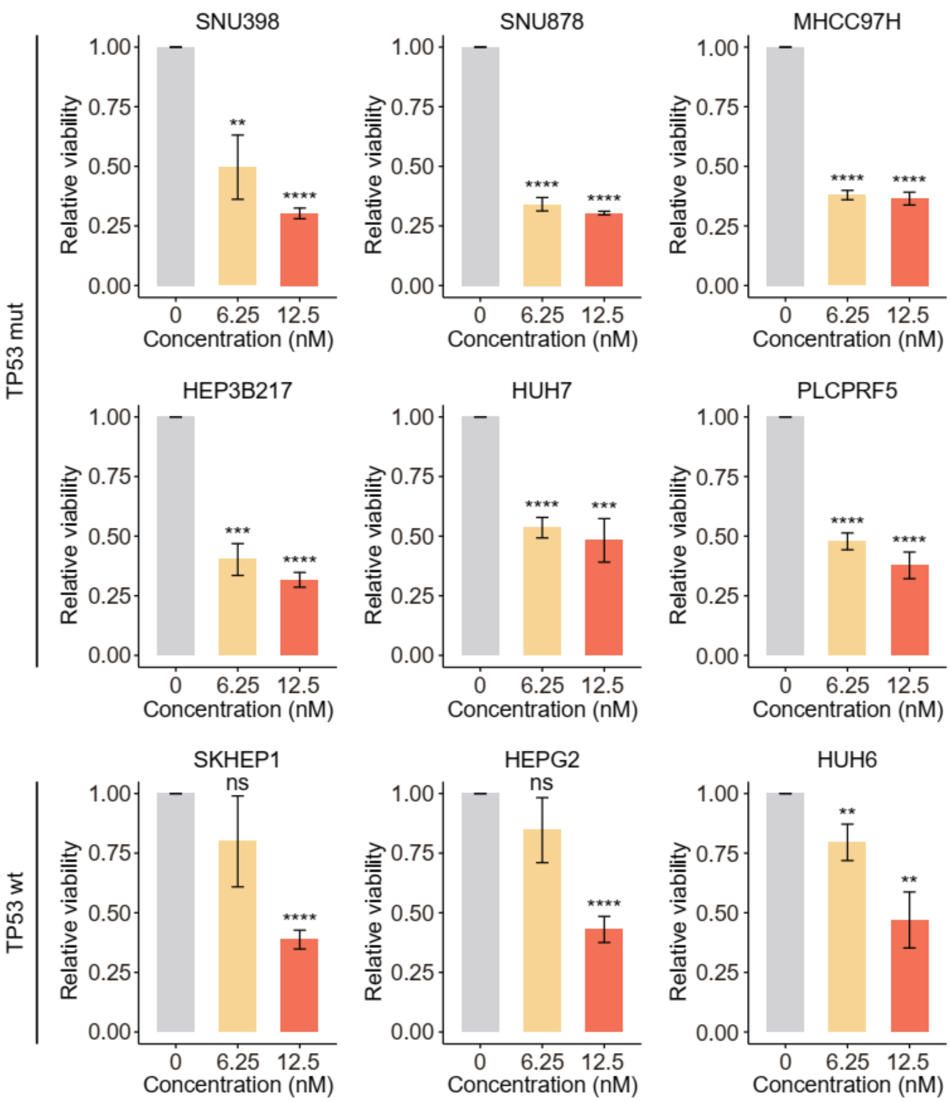

Supplementary Figure 8. The results of CellTiter-Blue (CTB) cell viability assays of cells treated with GSK461364. A total of nine liver cancer cell lines, including six *TP53*-mutant cell lines (SNU398, SNU878, MHCC97H, HEP3B217, HUH7, and PLCPRF5) and three *TP53*-wild-type cell lines (SKHEP1, HEPG2, and HUH6) were treated with GSK461364 (6.25nM and 12.5nM) and the cell viability was measured by CTB assays. Statistical significance of difference was determined using Student t-test (ns represents no significance, $^{**}P < 0.01$, $^{***}P < 0.001$, $^{****}P < 0.0001$).

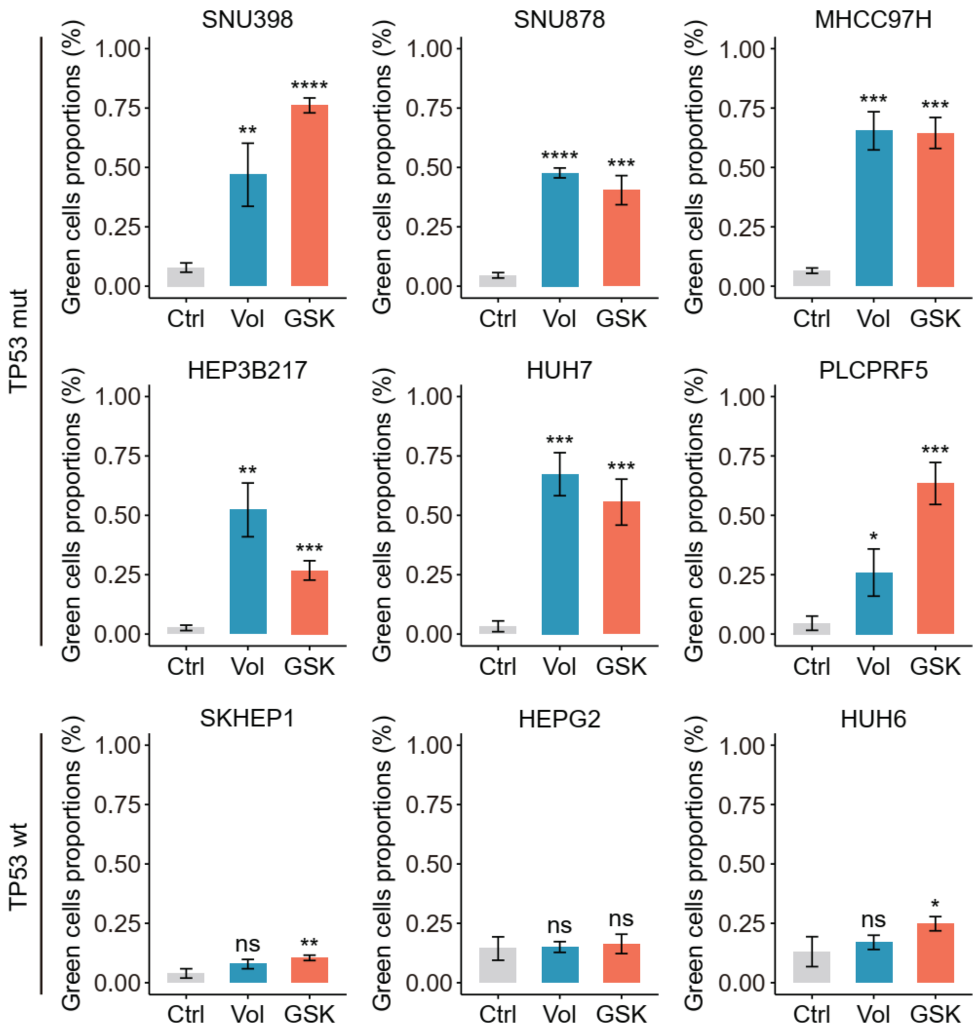

Supplementary Figure 9. The results of caspase-3/7 green assays of cells treated with volasertib and GSK461364. A total of nine liver cancer cell lines, including six *TP53*-mutant cell lines (SNU398, SNU878, MHCC97H, HEP3B217, HUH7, and PLCPRF5) and three *TP53*-wild-type cell lines (SKHEP1, HEPG2, and HUH6) were used to conduct caspase-3/7 green assays. Based on the results from cell proliferation assays, 12.5nM was chosen for volasertib treatment while 6.25nM was chosen for GSK461364 treatment. Statistical significance of difference was determined using Student t-test (ns represents no significance, *$P < 0.05$, **$P < 0.01$, ***$P < 0.001$, ****$P < 0.0001$).

References

1. Wang X, Simon R. Identification of potential synthetic lethal genes to p53 using a computational biology approach. BMC Med Genomics. 2013; 6: 30.
2. Kranthi T, Rao SB, Manimaran P. Identification of synthetic lethal pairs in biological systems through network information centrality. Mol Biosyst. 2013; 9: 2163-7.
3. Ye H, Zhang X, Chen Y, Liu Q, Wei J. Ranking novel cancer driving synthetic lethal gene pairs using TCGA data. Oncotarget. 2016; 7: 55352-67.
4. Jerby-Arnon L, Pfetzer N, Waldman YY, McGarry L, James D, Shanks E, et al. Predicting cancer-specific vulnerability via data-driven detection of synthetic lethality. Cell. 2014; 158: 1199-209.

Supplemental Table 1. Detailed information of previous studies providing driver gene lists

Publication	Employed algorithm	Gene number	PMID
Ahn	MutSigCV	9	24798001
Chaudhary	MutSigCV/OncodriveFM	10	30242023
Cleary	MutSig	13	23728943
Fujimoto_1	Statistical and functional analyses	15	22634756
Fujimoto_2	Statistical and functional analyses	37	27064257
Guichard	Statistical analyses	16	22561517
InTOGen	IntOGen pipeline	75	24037244
Jhunjhunwala	Statistical analyses	9	25159915
Kan	Statistical analyses	11	23788652
Li	MutSigCV/MutSigCL/ MutSigFN	43	29556353
Schulze	MutSigCV	161	25822088
Shiraishi	Statistical analyses	15	25526364
TCGA	MutSigCV	26	28622513
Totoki	Aggregated somatic alteration method /MutSigCV/Inactivation bias method	30	25362482

Supplemental Table 2. TSG-DT and TSG-DT-drug pairs

TSG-DT pairs				TSG-DT-drug pairs				
TSG gene	Target gene	TSG-DT		TSG gene	Target gene	Drug	TSG-DT-drug	Dataset
ARID1A	PPARG	ARID1A-PPARG		AXIN1	AURKB	barasertib	AXIN1-AURKB-barasertib	CTRP
AXIN1	ALDH18A1	AXIN1-ALDH18A1		AXIN1	CDK7	PHA-793887	AXIN1-CDK7-PHA-793887	CTRP
AXIN1	PRMT1	AXIN1-PRMT1		AXIN1	FGFR2	AZD4547	AXIN1-FGFR2-AZD4547	CTRP
AXIN1	RPL23	AXIN1-RPL23		AXIN1	FGFR2	erdafitinib	AXIN1-FGFR2-erdafitinib	PRISM
AXIN1	AURKB	AXIN1-AURKB		AXIN1	FGFR2	ponatinib	AXIN1-FGFR2-ponatinib	PRISM
AXIN1	IMPDH2	AXIN1-IMPDH2		AXIN1	FGFR2	dovitinib	AXIN1-FGFR2-dovitinib	PRISM
AXIN1	VEGFB	AXIN1-VEGFB		AXIN1	FGFR3	AZD4547	AXIN1-FGFR3-AZD4547	CTRP
AXIN1	CPNE1	AXIN1-CPNE1		AXIN1	FGFR3	erdafitinib	AXIN1-FGFR3-erdafitinib	PRISM
AXIN1	CDK7	AXIN1-CDK7		AXIN1	FGFR3	ponatinib	AXIN1-FGFR3-ponatinib	PRISM
AXIN1	RPS19	AXIN1-RPS19		AXIN1	FGFR3	dovitinib	AXIN1-FGFR3-dovitinib	PRISM
AXIN1	DNMT3A	AXIN1-DNMT3A		AXIN1	IMPDH2	AVN-944	AXIN1-IMPDH2-AVN-944	PRISM
AXIN1	GNAS	AXIN1-GNAS		AXIN1	WEE1	MK-1775	AXIN1-WEE1-MK-1775	CTRP
AXIN1	MAPK13	AXIN1-MAPK13		AXIN1	WEE1	MK-1775	AXIN1-WEE1-MK-1775	PRISM
AXIN1	P2RX4	AXIN1-P2RX4		BAP1	GSK3A	CHIR-99021	BAP1-GSK3A-CHIR-99021	GDSC
AXIN1	CHEK2	AXIN1-CHEK2		BAP1	GSK3A	indirubin	BAP1-GSK3A-indirubin	PRISM
AXIN1	PIK3R2	AXIN1-PIK3R2		BAP1	RAC1	NSC23766	BAP1-RAC1-NSC23766	CTRP
AXIN1	RPL15	AXIN1-RPL15		BAP1	RAC1	EHT-1864	BAP1-RAC1-EHT-1864	GDSC
AXIN1	PDE9A	AXIN1-PDE9A		BAP1	SRC	dasatinib	BAP1-SRC-dasatinib	CTRP
AXIN1	WEE1	AXIN1-WEE1		BAP1	SRC	saracatinib	BAP1-SRC-saracatinib	CTRP
AXIN1	MAP3K11	AXIN1-MAP3K11		BAP1	SRC	Ponatinib	BAP1-SRC-Ponatinib	GDSC
AXIN1	SNAP25	AXIN1-SNAP25		BAP1	SRC	Dasatinib	BAP1-SRC-Dasatinib	GDSC
AXIN1	FGFR3	AXIN1-FGFR3		BAP1	SRC	vandetanib	BAP1-SRC-vandetanib	PRISM
AXIN1	FGFR2	AXIN1-FGFR2		BAP1	SRC	bosutinib	BAP1-SRC-bosutinib	PRISM
BAP1	RAC1	BAP1-RAC1		BAP1	SRC	KX2-391	BAP1-SRC-KX2-391	PRISM
BAP1	NUDT1	BAP1-NUDT1		RB1	CDC25B	NSC95397	RB1-CDC25B-NSC95397	CTRP
BAP1	TYRO3	BAP1-TYRO3		RB1	CDK1	isopropylolomou	B1-CDK1-N9-isopropylolomouci	CTRP
BAP1	PRMT1	BAP1-PRMT1		RB1	CDK1	PHA-793887	RB1-CDK1-PHA-793887	CTRP

BAP1	CAPNS1	BAP1-CAPNS1		RB1	DNMT1	decitabine	RB1-DNMT1-decitabine	CTRP
BAP1	TRAPPC4	BAP1-TRAPPC4		RB1	DNMT1	zebularine	RB1-DNMT1-zebularine	CTRP
BAP1	PPP2R1A	BAP1-PPP2R1A		RB1	DNMT1	RG-108	RB1-DNMT1-RG-108	CTRP
BAP1	ARPC4	BAP1-ARPC4		RB1	DNMT1	azacitidine	RB1-DNMT1-azacitidine	CTRP
BAP1	GARS	BAP1-GARS		RB1	HDAC2	BRD-K11533227	RB1-HDAC2-BRD-K11533227	CTRP
BAP1	GSK3A	BAP1-GSK3A		RB1	HDAC2	ACY-1215	RB1-HDAC2-ACY-1215	PRISM
BAP1	YWHAQ	BAP1-YWHAQ		RB1	HDAC2	scriptaid	RB1-HDAC2-scriptaid	PRISM
BAP1	SMARCA4	BAP1-SMARCA4		RB1	MAPK3	CID-5458317	RB1-MAPK3-CID-5458317	PRISM
BAP1	PHB2	BAP1-PHB2		RB1	PARP2	olaparib	RB1-PARP2-olaparib	CTRP
BAP1	CSNK2A1	BAP1-CSNK2A1		RB1	PARP2	Talazoparib	RB1-PARP2-Talazoparib	GDSC
BAP1	PTGES3	BAP1-PTGES3		RB1	PARP2	olaparib	RB1-PARP2-olaparib	PRISM
BAP1	LCMT1	BAP1-LCMT1		RB1	PARP2	talazoparib	RB1-PARP2-talazoparib	PRISM
BAP1	KDM5C	BAP1-KDM5C		RB1	PLK1	GW-843682X	RB1-PLK1-GW-843682X	CTRP
BAP1	YWHAB	BAP1-YWHAB		RB1	PLK1	BRD-K70511574	RB1-PLK1-BRD-K70511574	CTRP
BAP1	YWHAH	BAP1-YWHAH		RB1	PLK1	BI-2536	RB1-PLK1-BI-2536	CTRP
BAP1	LARS	BAP1-LARS		RB1	PLK1	GSK461364	RB1-PLK1-GSK461364	CTRP
BAP1	IGF2R	BAP1-IGF2R		RB1	PLK1	BI-2536	RB1-PLK1-BI-2536	PRISM
BAP1	ARPC2	BAP1-ARPC2		RB1	PLK1	HMN-214	RB1-PLK1-HMN-214	PRISM
BAP1	SRC	BAP1-SRC		RB1	POLA1	clofarabine	RB1-POLA1-clofarabine	PRISM
BAP1	GPI	BAP1-GPI		RB1	PSMA4	carfilzomib	RB1-PSMA4-carfilzomib	PRISM
BAP1	DNM2	BAP1-DNM2		RB1	RAD51	B02	RB1-RAD51-B02	CTRP
BAP1	CSNK1E	BAP1-CSNK1E		RB1	RRM1	ciclopirox	RB1-RRM1-ciclopirox	CTRP
BAP1	ODC1	BAP1-ODC1		RB1	RRM1	gemcitabine	RB1-RRM1-gemcitabine	CTRP
BAP1	GLTP	BAP1-GLTP		RB1	RRM1	gemcitabine	RB1-RRM1-gemcitabine	PRISM
BAP1	DAGLB	BAP1-DAGLB		RB1	RRM1	clofarabine	RB1-RRM1-clofarabine	PRISM
BAP1	UBE2D2	BAP1-UBE2D2		RB1	RRM1	hydroxyurea	RB1-RRM1-hydroxyurea	PRISM
BAP1	PAK2	BAP1-PAK2		RB1	RRM1	triapine	RB1-RRM1-triapine	PRISM
BAP1	MARK2	BAP1-MARK2		RB1	SPHK1	SKI-II	RB1-SPHK1-SKI-II	CTRP
BAP1	EIF2S3	BAP1-EIF2S3		RB1	SPHK1	idronoxil	RB1-SPHK1-idronoxil	PRISM
BAP1	ACP1	BAP1-ACP1		RB1	SPHK1	SKI-II	RB1-SPHK1-SKI-II	PRISM
BAP1	DNMT3A	BAP1-DNMT3A		RB1	TOP1	topotecan	RB1-TOP1-topotecan	CTRP

BAP1	LIMK1	BAP1-LIMK1		RB1	TOP1	rubitecan	RB1-TOP1-rubitecan	PRISM
BAP1	KDM6A	BAP1-KDM6A		RB1	TOP1	topotecan	RB1-TOP1-topotecan	PRISM
PTEN	RHOA	PTEN-RHOA		RB1	TOP1	hydroxycamptoth	B1-TOP1-10-hydroxycamptothec	PRISM
PTEN	HMGB1	PTEN-HMGB1		RB1	TOP1	irinotecan	RB1-TOP1-irinotecan	PRISM
PTEN	MAP4K5	PTEN-MAP4K5		RB1	TOP1	genz-644282	RB1-TOP1-genz-644282	PRISM
PTEN	ARF6	PTEN-ARF6		RB1	TOP2A	etoposide	RB1-TOP2A-etoposide	CTRP
PTEN	CAPN1	PTEN-CAPN1		RB1	TOP2A	doxorubicin	RB1-TOP2A-doxorubicin	CTRP
PTEN	CASK	PTEN-CASK		RB1	TOP2A	mitoxantrone	RB1-TOP2A-mitoxantrone	PRISM
PTEN	PSEN1	PTEN-PSEN1		RB1	TOP2A	doxorubicin	RB1-TOP2A-doxorubicin	PRISM
RB1	CDK1	RB1-CDK1		RB1	TOP2A	etoposide	RB1-TOP2A-etoposide	PRISM
RB1	PLK1	RB1-PLK1		RB1	TOP2A	norfloxacin	RB1-TOP2A-norfloxacin	PRISM
RB1	CLIC1	RB1-CLIC1		RB1	TOP2A	daunorubicin	RB1-TOP2A-daunorubicin	PRISM
RB1	FOXM1	RB1-FOXM1		RB1	TOP2A	sparfloxacin	RB1-TOP2A-sparfloxacin	PRISM
RB1	KDM1A	RB1-KDM1A		RB1	TOP2A	amsacrine	RB1-TOP2A-amsacrine	PRISM
RB1	NEK2	RB1-NEK2		RB1	TOP2A	valrubicin	RB1-TOP2A-valrubicin	PRISM
RB1	TOP2A	RB1-TOP2A		RB1	TOP2A	trovafloxacin	RB1-TOP2A-trovafloxacin	PRISM
RB1	CIT	RB1-CIT		RB1	TOP2A	oposide-phospha	RB1-TOP2A-etoposide-phosphat	PRISM
RB1	PRC1	RB1-PRC1		RB1	TOP2B	etoposide	RB1-TOP2B-etoposide	PRISM
RB1	CCNA2	RB1-CCNA2		RB1	TOP2B	daunorubicin	RB1-TOP2B-daunorubicin	PRISM
RB1	RAD51	RB1-RAD51		RB1	TUBA1C	vinorelbine	RB1-TUBA1C-vinorelbine	PRISM
RB1	PLK4	RB1-PLK4		RB1	TUBB	ABT-751	RB1-TUBB-ABT-751	PRISM
RB1	HDAC2	RB1-HDAC2		RB1	TUBB	paclitaxel	RB1-TUBB-paclitaxel	PRISM
RB1	MAPKAPK5	RB1-MAPKAPK5		RB1	TUBB	docetaxel	RB1-TUBB-docetaxel	PRISM
RB1	APEX1	RB1-APEX1		RB1	TUBB	vinorelbine	RB1-TUBB-vinorelbine	PRISM
RB1	RAN	RB1-RAN		RB1	TUBB	cabazitaxel	RB1-TUBB-cabazitaxel	PRISM
RB1	TTK	RB1-TTK		RB1	USP13	spautin-1	RB1-USP13-spautin-1	CTRP
RB1	KIF18A	RB1-KIF18A		TP53	ADA	vidarabine	TP53-ADA-vidarabine	PRISM
RB1	CDC42	RB1-CDC42		TP53	ADA	pentostatin	TP53-ADA-pentostatin	PRISM
RB1	VDAC1	RB1-VDAC1		TP53	ADA	fludarabine	TP53-ADA-fludarabine	PRISM
RB1	USP13	RB1-USP13		TP53	CDK1	AT-7519	TP53-CDK1-AT-7519	GDSC
RB1	HNRNPL	RB1-HNRNPL		TP53	CDK16	SNS-032	TP53-CDK16-SNS-032	CTRP

RB1	CENPE	RB1-CENPE		TP53	CHEK1	AZD7762	TP53-CHEK1-AZD7762	CTRP
RB1	TUBA1C	RB1-TUBA1C		TP53	CHEK1	Wee1 Inhibitor	TP53-CHEK1-Wee1 Inhibitor	GDSC
RB1	CDC25B	RB1-CDC25B		TP53	DNMT1	zebularine	TP53-DNMT1-zebularine	CTRP
RB1	YWHAQ	RB1-YWHAQ		TP53	EZH2	BRD1835	TP53-EZH2-BRD1835	CTRP
RB1	YWHAB	RB1-YWHAB		TP53	FKBP1A	tacrolimus	TP53-FKBP1A-tacrolimus	PRISM
RB1	UBE2D2	RB1-UBE2D2		TP53	FKBP1A	sirolimus	TP53-FKBP1A-sirolimus	PRISM
RB1	PTGES3	RB1-PTGES3		TP53	HDAC2	LBH-589	TP53-HDAC2-LBH-589	CTRP
RB1	HMMR	RB1-HMMR		TP53	HDAC2	apicidin	TP53-HDAC2-apicidin	CTRP
RB1	MAPK3	RB1-MAPK3		TP53	HDAC2	entinostat	TP53-HDAC2-entinostat	CTRP
RB1	PARP2	RB1-PARP2		TP53	HDAC2	Merck60	TP53-HDAC2-Merck60	CTRP
RB1	DNMT1	RB1-DNMT1		TP53	HDAC2	BRD-K11533227	TP53-HDAC2-BRD-K11533227	CTRP
RB1	ENO1	RB1-ENO1		TP53	HDAC2	scriptaid	TP53-HDAC2-scriptaid	PRISM
RB1	PSMA4	RB1-PSMA4		TP53	HSP90AA1	tanespimycin	TP53-HSP90AA1-tanespimycin	CTRP
RB1	SFPQ	RB1-SFPQ		TP53	HSP90AA1	SNX-2112	TP53-HSP90AA1-SNX-2112	CTRP
RB1	USP1	RB1-USP1		TP53	HSP90AA1	tanespimycin	TP53-HSP90AA1-tanespimycin	PRISM
RB1	YWHAH	RB1-YWHAH		TP53	IMPDH1	mercaptopurine	TP53-IMPDH1-mercaptopurine	PRISM
RB1	SF3A3	RB1-SF3A3		TP53	IMPDH1	azathioprine	TP53-IMPDH1-azathioprine	PRISM
RB1	UBE2N	RB1-UBE2N		TP53	IRAK1	WZ3105	TP53-IRAK1-WZ3105	GDSC
RB1	TOP2B	RB1-TOP2B		TP53	KIF11	SB-743921	TP53-KIF11-SB-743921	CTRP
RB1	INCENP	RB1-INCENP		TP53	KIF11	ARRY-520	TP53-KIF11-ARRY-520	GDSC
RB1	G6PD	RB1-G6PD		TP53	KIF11	litronesib	TP53-KIF11-litronesib	PRISM
RB1	SPHK1	RB1-SPHK1		TP53	KIF11	ispinesib	TP53-KIF11-ispinesib	PRISM
RB1	IL12A	RB1-IL12A		TP53	M6PR	adaprev	TP53-M6PR-adaprev	PRISM
RB1	ALDOA	RB1-ALDOA		TP53	MAP2K2	MEK1-2-inhibito	TP53-MAP2K2-MEK1-2-inhibito	PRISM
RB1	DDX39B	RB1-DDX39B		TP53	MAP3K9	GDC-0980	TP53-MAP3K9-GDC-0980	PRISM
RB1	PPP5C	RB1-PPP5C		TP53	MAP4	docetaxel	TP53-MAP4-docetaxel	PRISM
RB1	HNRNPH1	RB1-HNRNPH1		TP53	MAP4	paclitaxel	TP53-MAP4-paclitaxel	PRISM
RB1	PHB2	RB1-PHB2		TP53	MAPK1	C6-ceramide	TP53-MAPK1-C6-ceramide	CTRP
RB1	POLA1	RB1-POLA1		TP53	MAPK3	CID-5458317	TP53-MAPK3-CID-5458317	PRISM
RB1	PKN1	RB1-PKN1		TP53	MMP14	marimastat	TP53-MMP14-marimastat	PRISM
RB1	STK24	RB1-STK24		TP53	PKN1	RKI-1447	TP53-PKN1-RKI-1447	PRISM

RB1	TYRO3	RB1-TYRO3		TP53	PLK1	BI-2536	TP53-PLK1-BI-2536	CTRP
RB1	RRM1	RB1-RRM1		TP53	PLK1	BRD-K70511574	TP53-PLK1-BRD-K70511574	CTRP
RB1	SMARCA4	RB1-SMARCA4		TP53	PLK1	GSK461364	TP53-PLK1-GSK461364	CTRP
RB1	FUBP1	RB1-FUBP1		TP53	PLK1	rigosertib	TP53-PLK1-rigosertib	CTRP
RB1	TOP1	RB1-TOP1		TP53	PLK1	GW-843682X	TP53-PLK1-GW-843682X	CTRP
RB1	RHEB	RB1-RHEB		TP53	PLK1	MK-1775	TP53-PLK1-MK-1775	GDSC
RB1	ARPC3	RB1-ARPC3		TP53	PLK1	BI-2536	TP53-PLK1-BI-2536	PRISM
RB1	HNRNPD	RB1-HNRNPD		TP53	PLK1	volasertib	TP53-PLK1-volasertib	PRISM
RB1	CDC7	RB1-CDC7		TP53	PLK1	GW-843682X	TP53-PLK1-GW-843682X	PRISM
RB1	HNRNPK	RB1-HNRNPK		TP53	PLK1	HMN-214	TP53-PLK1-HMN-214	PRISM
RB1	HNRNPH3	RB1-HNRNPH3		TP53	POLA1	clofarabine	TP53-POLA1-clofarabine	PRISM
RB1	EED	RB1-EED		TP53	POLD1	clofarabine	TP53-POLD1-clofarabine	PRISM
RB1	HNRNPA2B1	RB1-HNRNPA2B1		TP53	PPAT	mercaptopurine	TP53-PPAT-mercaptopurine	PRISM
RB1	TUBB	RB1-TUBB		TP53	PPAT	azathioprine	TP53-PPAT-azathioprine	PRISM
RB1	MTAP	RB1-MTAP		TP53	PRKCD	tamoxifen	TP53-PRKCD-tamoxifen	PRISM
RB1	IRAK1	RB1-IRAK1		TP53	PSMB5	MLN2238	TP53-PSMB5-MLN2238	CTRP
RB1	HSPA13	RB1-HSPA13		TP53	PSMB5	bortezomib	TP53-PSMB5-bortezomib	CTRP
RB1	EIF2S1	RB1-EIF2S1		TP53	PSMB5	carfilzomib	TP53-PSMB5-carfilzomib	PRISM
TP53	CDK1	TP53-CDK1		TP53	RAC1	NSC23766	TP53-RAC1-NSC23766	CTRP
TP53	RAC1	TP53-RAC1		TP53	RAD51	B02	TP53-RAD51-B02	CTRP
TP53	CCT3	TP53-CCT3		TP53	ROCK1	GSK429286A	TP53-ROCK1-GSK429286A	GDSC
TP53	PPAT	TP53-PPAT		TP53	ROCK1	Y-27632	TP53-ROCK1-Y-27632	PRISM
TP53	RAN	TP53-RAN		TP53	ROCK1	GSK429286A	TP53-ROCK1-GSK429286A	PRISM
TP53	RPIA	TP53-RPIA		TP53	ROCK1	RKI-1447	TP53-ROCK1-RKI-1447	PRISM
TP53	HM13	TP53-HM13		TP53	RRM1	gemcitabine	TP53-RRM1-gemcitabine	PRISM
TP53	RAD51	TP53-RAD51		TP53	RRM1	hydroxyurea	TP53-RRM1-hydroxyurea	PRISM
TP53	KIF2C	TP53-KIF2C		TP53	RRM1	fludarabine	TP53-RRM1-fludarabine	PRISM
TP53	UBA1	TP53-UBA1		TP53	RRM1	clofarabine	TP53-RRM1-clofarabine	PRISM
TP53	HDAC2	TP53-HDAC2		TP53	TACC3	KHS101	TP53-TACC3-KHS101	CTRP
TP53	PLK1	TP53-PLK1		TP53	TOP2A	trovafloxacin	TP53-TOP2A-trovafloxacin	PRISM
TP53	TYRO3	TP53-TYRO3		TP53	TOP2A	sparfloxacin	TP53-TOP2A-sparfloxacin	PRISM

TP53	RANBP1	TP53-RANBP1		TP53	TOP2A	mitoxantrone	TP53-TOP2A-mitoxantrone	PRISM
TP53	FOXM1	TP53-FOXM1		TP53	TOP2A	daunorubicin	TP53-TOP2A-daunorubicin	PRISM
TP53	EED	TP53-EED		TP53	TOP2A	etoposide	TP53-TOP2A-etoposide	PRISM
TP53	PTGES3	TP53-PTGES3		TP53	TOP2A	oposide-phospha	P53-TOP2A-etoposide-phosphat	PRISM
TP53	CIT	TP53-CIT		TP53	TOP2A	podophyllotoxin	TP53-TOP2A-podophyllotoxin	PRISM
TP53	PLK4	TP53-PLK4		TP53	TOP2A	amsacrine	TP53-TOP2A-amsacrine	PRISM
TP53	YWHAH	TP53-YWHAH		TP53	TOP2A	doxorubicin	TP53-TOP2A-doxorubicin	PRISM
TP53	CCNA2	TP53-CCNA2		TP53	TYMS	gemcitabine	TP53-TYMS-gemcitabine	PRISM
TP53	RPL4	TP53-RPL4		TP53	TYMS	capecitabine	TP53-TYMS-capecitabine	PRISM
TP53	TOP2A	TP53-TOP2A		TP53	TYMS	raltitrexed	TP53-TYMS-raltitrexed	PRISM
TP53	INCENP	TP53-INCENP		TP53	USP1	ML323	TP53-USP1-ML323	GDSC
TP53	IMPDH1	TP53-IMPDH1		TP53	USP1	NSC-632839	TP53-USP1-NSC-632839	PRISM
TP53	EZH2	TP53-EZH2		TP53	USP14	IU1	TP53-USP14-IU1	CTRP
TP53	PSMB5	TP53-PSMB5		TP53	USP14	WP1130	TP53-USP14-WP1130	CTRP
TP53	FARSA	TP53-FARSA						
TP53	NEK2	TP53-NEK2						
TP53	USP1	TP53-USP1						
TP53	YWHAQ	TP53-YWHAQ						
TP53	HSPD1	TP53-HSPD1						
TP53	RPSA	TP53-RPSA						
TP53	NPM1	TP53-NPM1						
TP53	RIOK1	TP53-RIOK1						
TP53	HSP90AA1	TP53-HSP90AA1						
TP53	APEX1	TP53-APEX1						
TP53	TTK	TP53-TTK						
TP53	ASNA1	TP53-ASNA1						
TP53	DNMT1	TP53-DNMT1						
TP53	POLD1	TP53-POLD1						
TP53	FARSB	TP53-FARSB						
TP53	KIF11	TP53-KIF11						
TP53	CDC7	TP53-CDC7						

TP53	HSPB1	TP53-HSPB1						
TP53	RALA	TP53-RALA						
TP53	EIF2S1	TP53-EIF2S1						
TP53	BRD9	TP53-BRD9						
TP53	PLCB3	TP53-PLCB3						
TP53	KDM3A	TP53-KDM3A						
TP53	CSNK1G1	TP53-CSNK1G1						
TP53	SLC25A6	TP53-SLC25A6						
TP53	MTAP	TP53-MTAP						
TP53	MAPKAPK5	TP53-MAPKAPK5						
TP53	M6PR	TP53-M6PR						
TP53	PKN1	TP53-PKN1						
TP53	IRAK1	TP53-IRAK1						
TP53	CDC42	TP53-CDC42						
TP53	KIF18A	TP53-KIF18A						
TP53	GYG1	TP53-GYG1						
TP53	HNRNPL	TP53-HNRNPL						
TP53	USP14	TP53-USP14						
TP53	EIF3F	TP53-EIF3F						
TP53	MAP3K9	TP53-MAP3K9						
TP53	PRMT1	TP53-PRMT1						
TP53	HDAC11	TP53-HDAC11						
TP53	KDM1A	TP53-KDM1A						
TP53	ROCK1	TP53-ROCK1						
TP53	TACC3	TP53-TACC3						
TP53	CSNK1E	TP53-CSNK1E						
TP53	CCNB1	TP53-CCNB1						
TP53	FEN1	TP53-FEN1						
TP53	ADA	TP53-ADA						
TP53	CREB1	TP53-CREB1						
TP53	GARS	TP53-GARS						

TP53	GLB1	TP53-GLB1							
TP53	DCK	TP53-DCK							
TP53	MAP4	TP53-MAP4							
TP53	SFPQ	TP53-SFPQ							
TP53	CDK5R1	TP53-CDK5R1							
TP53	CDC25A	TP53-CDC25A							
TP53	TYMS	TP53-TYMS							
TP53	UBE2N	TP53-UBE2N							
TP53	DARS2	TP53-DARS2							
TP53	EGLN3	TP53-EGLN3							
TP53	CAD	TP53-CAD							
TP53	PAFAH1B3	TP53-PAFAH1B3							
TP53	STK39	TP53-STK39							
TP53	EHMT2	TP53-EHMT2							
TP53	PHB2	TP53-PHB2							
TP53	HSPH1	TP53-HSPH1							
TP53	VARS	TP53-VARS							
TP53	TPM3	TP53-TPM3							
TP53	TFRC	TP53-TFRC							
TP53	ARPC2	TP53-ARPC2							
TP53	MAP2K2	TP53-MAP2K2							
TP53	STIP1	TP53-STIP1							
TP53	PRC1	TP53-PRC1							
TP53	CLIC1	TP53-CLIC1							
TP53	MAPK3	TP53-MAPK3							
TP53	CPNE1	TP53-CPNE1							
TP53	PI4KA	TP53-PI4KA							
TP53	POLA1	TP53-POLA1							
TP53	PRKCD	TP53-PRKCD							
TP53	MAPK1	TP53-MAPK1							
TP53	HNRNPA2B1	TP53-HNRNPA2B1							

TP53	RRM1	TP53-RRM1						
TP53	RPL23	TP53-RPL23						
TP53	PAK2	TP53-PAK2						
TP53	ITPA	TP53-ITPA						
TP53	PPIH	TP53-PPIH						
TP53	APAF1	TP53-APAF1						
TP53	SMARCA4	TP53-SMARCA4						
TP53	FKBP1A	TP53-FKBP1A						
TP53	EWSR1	TP53-EWSR1						
TP53	CDC25B	TP53-CDC25B						
TP53	SLC2A1	TP53-SLC2A1						
TP53	CALM2	TP53-CALM2						
TP53	MAT2A	TP53-MAT2A						
TP53	MMP14	TP53-MMP14						
TP53	BRCC3	TP53-BRCC3						
TP53	HP1BP3	TP53-HP1BP3						
TP53	HSP90AB1	TP53-HSP90AB1						
TP53	MAP3K4	TP53-MAP3K4						
TP53	MYL6B	TP53-MYL6B						
TP53	DARS	TP53-DARS						
TP53	PRKAG1	TP53-PRKAG1						
TP53	UBE2D2	TP53-UBE2D2						
TP53	CHEK1	TP53-CHEK1						
TP53	ARPC4	TP53-ARPC4						
TP53	APLP2	TP53-APLP2						
TP53	LARS	TP53-LARS						
TP53	CA9	TP53-CA9						
TP53	CDK16	TP53-CDK16						

Supplemental Table 3. Ranking of 272 TSG-DT pairs

TSG-DT pairs	Rank_cor	Rank_DE	Rank_FS	Rank_cox	RAS
RB1-FOXM1	259	5	3	10	4.099
RB1-INCENP	20	40	2	127	3.299
RB1-TOP2A	225	1	46	17	3.273
RB1-PLK1	257	4	105	4	3.137
TP53-INCENP	112	73	1	86	3.064
RB1-CDK1	256	6	219	2	2.864
AXIN1-MAPK13	110	52	6	33	2.689
TP53-FOXM1	161	21	4	64	2.665
RB1-FUBP1	4	119	13	184	2.585
TP53-HDAC2	25	109	12	57	2.513
AXIN1-CPNE1	35	142	29	19	2.504
RB1-TOP1	1	175	63	186	2.483
RB1-PRC1	205	3	87	26	2.437
TP53-RPIA	26	143	24	43	2.366
RB1-HSPA13	13	65	9	271	2.351
TP53-RAD51	221	37	5	53	2.329
TP53-TOP2A	266	13	10	84	2.235
RB1-SFPQ	42	89	11	115	2.164
RB1-HDAC2	21	118	64	35	2.144
RB1-HNRNPK	2	219	45	203	2.131
AXIN1-ALDH18A1	125	173	68	3	2.124
ARID1A-PPARG	198	136	27	5	2.097
RB1-DNMT1	134	10	36	107	2.079
RB1-POLA1	54	14	53	166	2.059
AXIN1-PRMT1	37	218	80	6	2.042
BAP1-RAC1	220	139	246	1	2.013
RB1-NEK2	246	7	175	16	2.011
AXIN1-AURKB	43	128	130	11	1.989
RB1-USP1	87	9	125	120	1.954
RB1-TOP2B	3	209	82	126	1.941
BAP1-TRAPPC4	245	80	8	32	1.921
TP53-RAN	60	148	30	42	1.908
TP53-CDK1	219	30	114	8	1.866
RB1-RAD51	260	47	39	30	1.813
BAP1-KDM5C	15	124	101	91	1.808
BAP1-CSNK2A1	88	102	73	85	1.748
RB1-CCNA2	235	20	79	29	1.727
TP53-SMARCA4	6	256	28	243	1.721
RB1-CDC7	251	2	134	201	1.720
TP53-DNMT1	62	122	23	118	1.716
RB1-STK24	5	112	183	170	1.699
TP53-TACC3	177	32	15	177	1.698
TP53-POLD1	111	75	33	119	1.614
TP53-SFPQ	73	156	7	195	1.602
TP53-EZH2	192	53	16	90	1.599

RB1-HNRNPH3	14	199	31	204	1.596
TP53-PLK1	176	26	75	59	1.591
RB1-RAN	93	57	136	41	1.572
TP53-CSNK1E	7	216	57	178	1.563
AXIN1-IMPDH2	150	163	74	13	1.531
TP53-UBA1	39	242	60	56	1.529
RB1-SF3A3	119	64	55	123	1.529
RB1-KDM1A	113	178	62	15	1.526
BAP1-TYRO3	253	22	261	20	1.496
TP53-HNRNPL	9	232	50	158	1.483
RB1-CLIC1	143	55	271	7	1.476
TP53-PRMT1	16	161	81	163	1.429
TP53-CSNK1G1	31	258	19	138	1.419
AXIN1-PDE9A	269	25	42	81	1.418
RB1-PLK4	115	49	169	34	1.415
RB1-YWHAQ	22	61	260	75	1.404
TP53-CREB1	38	231	14	185	1.403
AXIN1-DNMT3A	57	174	118	25	1.392
RB1-YWHAB	10	206	172	76	1.382
RB1-CENPE	155	28	149	68	1.353
RB1-HNRNPH1	28	130	76	154	1.349
PTEN-MAP4K5	59	84	88	164	1.326
RB1-RRM1	146	11	188	176	1.320
TP53-KDM1A	145	78	21	169	1.319
RB1-PARP2	195	70	26	103	1.297
TP53-NPM1	66	240	17	109	1.283
RB1-HNRNPA2B1	8	149	98	259	1.279
RB1-HNRNPL	33	184	106	67	1.271
RB1-CDC42	12	187	262	49	1.251
TP53-NEK2	250	24	59	96	1.250
RB1-KIF18A	147	31	204	47	1.236
AXIN1-PIK3R2	224	205	18	48	1.231
BAP1-NUDT1	186	87	185	18	1.219
TP53-EWSR1	11	263	52	245	1.212
TP53-KIF2C	249	15	170	55	1.189
RB1-TTK	215	16	255	45	1.180
AXIN1-RPS19	272	151	41	22	1.174
TP53-PTGES3	84	181	96	69	1.173
AXIN1-VEGFB	159	66	250	14	1.166
RB1-EED	69	93	49	216	1.158
BAP1-CSNK1E	49	79	133	161	1.157
TP53-CCNA2	237	56	84	80	1.140
RB1-UBE2N	19	195	147	125	1.137
RB1-HMMR	226	8	264	97	1.136
TP53-POLA1	67	141	20	231	1.134
AXIN1-P2RX4	77	157	168	40	1.132
RB1-CDC25B	271	29	92	72	1.127
RB1-CIT	211	45	244	23	1.111

AXIN1-GNAS	162	168	128	31	1.100
TP53-CCNB1	234	18	65	179	1.097
TP53-RAC1	121	228	189	12	1.093
TP53-EED	91	249	58	65	1.092
RB1-DDX39B	140	121	70	150	1.084
AXIN1-RPL23	262	223	108	9	1.070
RB1-APEX1	120	176	129	39	1.061
TP53-APEX1	40	253	56	113	1.060
TP53-STIP1	78	81	93	225	1.058
TP53-UBE2N	53	212	37	202	1.052
BAP1-SMARCA4	108	69	217	66	1.037
AXIN1-FGFR2	231	17	54	266	1.037
RB1-TUBA1C	160	96	150	70	1.030
RB1-YWHAH	24	105	234	122	1.030
TP53-TYRO3	118	72	203	61	1.019
RB1-HNRNPD	55	132	69	198	1.016
TP53-YWHAQ	90	159	141	104	1.009
BAP1-PRMT1	204	91	156	27	1.009
TP53-PRKCD	63	120	47	232	1.007
AXIN1-CHEK2	152	170	117	44	1.005
TP53-RIOK1	32	234	109	111	1.004
TP53-RPL4	76	254	91	82	1.002
RB1-SPHK1	203	12	243	136	0.997
AXIN1-FGFR3	139	34	78	214	0.986
RB1-SMARCA4	130	194	25	181	0.983
TP53-CCT3	127	197	184	24	0.981
TP53-HNRNPA2B1	41	257	43	234	0.980
BAP1-DNMT3A	79	33	124	237	0.970
TP53-PLK4	169	107	138	74	0.957
TP53-KDM3A	70	244	34	134	0.945
BAP1-MARK2	17	83	251	213	0.941
TP53-PKN1	72	214	44	145	0.937
AXIN1-WEE1	171	169	94	94	0.927
TP53-TTK	227	19	195	116	0.922
AXIN1-CDK7	157	230	140	21	0.922
BAP1-ARPC4	92	192	151	46	0.921
TP53-YWHAH	47	207	148	79	0.907
AXIN1-SNAP25	207	36	83	175	0.907
TP53-RANBP1	174	113	154	63	0.904
RB1-MAPK3	124	137	171	102	0.901
BAP1-GSK3A	44	180	248	54	0.901
RB1-MAPKAPK5	64	239	167	36	0.900
BAP1-YWHAQ	187	106	139	58	0.894
RB1-PTGES3	71	193	153	83	0.879
TP53-RPSA	173	154	107	106	0.876
BAP1-PPP2R1A	144	85	232	37	0.862
TP53-EHMT2	52	165	77	211	0.849
BAP1-LARS	122	138	180	100	0.847

TP53-KIF18A	184	51	163	148	0.840
TP53-FARSB	131	126	190	121	0.823
TP53-CDC7	228	63	86	128	0.820
BAP1-CAPNS1	210	160	206	28	0.818
TP53-PLCB3	56	210	113	133	0.814
BAP1-SRC	190	44	191	143	0.812
TP53-PRC1	196	23	158	226	0.811
BAP1-YWHAH	223	60	127	98	0.810
RB1-USP13	209	104	137	60	0.803
RB1-EIF2S1	23	71	236	272	0.803
TP53-MAPKAPK5	95	185	126	142	0.803
PTEN-PSEN1	34	92	197	223	0.798
TP53-FARSA	18	271	241	93	0.797
BAP1-GLTP	175	166	66	180	0.795
BAP1-LIMK1	61	58	222	241	0.788
RB1-VDAC1	94	140	228	50	0.786
BAP1-IGF2R	83	111	254	101	0.781
TP53-CPNE1	30	183	112	229	0.777
TP53-FEN1	236	50	72	182	0.765
TP53-IRAK1	180	144	161	146	0.747
PTEN-ARF6	80	74	223	168	0.739
TP53-VARS	102	267	22	218	0.727
TP53-TYMS	123	68	160	200	0.715
AXIN1-MAP3K11	105	225	85	137	0.698
TP53-HSP90AB1	82	196	38	253	0.694
BAP1-ODC1	188	90	174	172	0.686
PTEN-RHOA	167	158	201	62	0.685
TP53-IMPDH1	137	82	253	88	0.679
TP53-USP1	206	97	181	99	0.678
RB1-PPP5C	98	110	210	152	0.676
AXIN1-RPL15	258	247	40	73	0.675
TP53-HSP90AA1	170	264	32	112	0.664
RB1-UBE2D2	81	215	227	78	0.664
RB1-ALDOA	244	27	212	149	0.663
RB1-G6PD	168	46	224	135	0.663
TP53-GYG1	45	189	214	156	0.659
TP53-CHEK1	191	59	71	260	0.658
TP53-CIT	264	77	157	71	0.656
BAP1-LCMT1	208	182	103	89	0.653
TP53-ITPA	153	177	35	239	0.651
BAP1-YWHAB	247	147	67	95	0.646
TP53-LARS	27	268	100	264	0.634
TP53-GARS	158	190	176	187	0.625
TP53-MAPK3	103	179	61	228	0.618
BAP1-PTGES3	194	204	120	87	0.614
TP53-HDAC11	185	114	194	165	0.607
TP53-MAPK1	104	198	51	233	0.607
PTEN-HMGB1	197	98	192	151	0.602

TP53-CLIC1	74	94	211	227	0.593
BAP1-DNM2	85	101	239	157	0.590
RB1-TYRO3	230	38	226	171	0.590
TP53-KIF11	148	48	269	124	0.585
TP53-CDC25A	240	43	121	199	0.581
TP53-MAP4	46	221	216	194	0.579
TP53-HSPD1	243	200	48	105	0.577
TP53-ARPC2	48	203	202	222	0.570
TP53-EGLN3	232	62	102	206	0.568
TP53-CDC42	202	115	187	147	0.561
PTEN-CAPN1	172	76	215	174	0.552
TP53-STK39	182	129	122	210	0.549
TP53-CDC25B	214	39	135	246	0.546
BAP1-GARS	248	100	208	52	0.545
TP53-HM13	193	222	218	51	0.543
TP53-MAP3K4	29	237	237	254	0.541
TP53-CAD	58	233	116	208	0.534
BAP1-PAK2	109	164	182	207	0.529
TP53-PPAT	117	243	257	38	0.524
BAP1-PHB2	242	99	196	77	0.516
BAP1-DAGLB	129	86	220	189	0.514
TP53-FKBP1A	50	146	186	244	0.509
TP53-BRD9	128	236	155	132	0.507
PTEN-CASK	138	54	240	191	0.503
RB1-ENO1	270	42	209	110	0.493
TP53-CDK16	36	127	235	267	0.485
TP53-TPM3	51	153	245	220	0.442
TP53-RRM1	165	134	165	235	0.439
TP53-MMP14	65	152	179	250	0.433
TP53-PAFAH1B3	154	41	268	209	0.430
TP53-APAF1	96	213	111	242	0.426
BAP1-EIF2S3	166	150	199	217	0.423
TP53-CA9	254	35	225	265	0.413
TP53-ADA	189	171	221	183	0.409
TP53-HSPH1	149	226	97	215	0.408
TP53-HP1BP3	181	125	90	252	0.402
TP53-CALM2	68	238	123	248	0.397
TP53-DARS2	222	186	119	205	0.388
TP53-PAK2	86	208	143	238	0.379
TP53-HSPB1	241	202	104	129	0.378
TP53-USP14	151	135	265	159	0.372
TP53-PPIH	212	95	145	240	0.347
TP53-ASNA1	229	252	99	117	0.346
TP53-EIF2S1	163	265	164	131	0.342
TP53-M6PR	116	188	247	144	0.340
RB1-PKN1	267	117	152	167	0.332
TP53-RALA	141	167	270	130	0.331
TP53-TFRC	218	108	231	221	0.324

TP53-ARPC4	101	191	132	262	0.314
BAP1-ACP1	217	155	229	219	0.308
TP53-RPL23	142	248	89	236	0.306
RB1-TUBB	183	88	177	268	0.287
TP53-UBE2D2	97	266	115	258	0.283
TP53-MAP3K9	178	261	178	162	0.280
TP53-BRCC3	126	260	95	251	0.275
TP53-PRKAG1	133	220	110	257	0.274
BAP1-UBE2D2	238	162	213	190	0.265
TP53-CDK5R1	132	262	142	196	0.263
TP53-SLC2A1	233	67	256	247	0.261
RB1-MTAP	107	145	193	269	0.254
BAP1-ARPC2	216	131	266	108	0.251
RB1-ARPC3	164	224	238	197	0.248
TP53-SLC25A6	100	269	205	139	0.246
RB1-PSMA4	252	217	159	114	0.242
TP53-MTAP	213	245	198	140	0.233
TP53-PSMB5	268	246	131	92	0.232
BAP1-KDM6A	199	116	166	261	0.230
TP53-EIF3F	89	251	258	160	0.223
RB1-RHEB	99	211	259	188	0.217
TP53-GLB1	201	255	173	192	0.210
TP53-PI4KA	75	241	263	230	0.202
TP53-PHB2	114	272	146	212	0.194
TP53-MAT2A	136	201	233	249	0.186
TP53-DARS	106	229	200	256	0.185
TP53-ROCK1	179	250	242	173	0.181
TP53-MAP2K2	135	270	144	224	0.177
RB1-IL12A	200	172	272	141	0.167
BAP1-GPI	261	103	267	153	0.165
TP53-MYL6B	255	133	252	255	0.135
TP53-DCK	156	259	249	193	0.116
RB1-PHB2	265	235	207	155	0.090
RB1-IRAK1	239	123	230	270	0.078
TP53-APLP2	263	227	162	263	0.071

Supplemental Table 4. Results of differential expression analysis

Gene	Mean expression in normal	Mean expression in tumor	logFC	P.Value	adj.P.Val
A1BG	8.490	7.114	-1.376	0.000	0.000
A1CF	6.066	5.799	-0.267	0.000	0.000
A2M	10.597	9.169	-1.428	0.000	0.000
A4GALT	1.616	2.189	0.574	0.000	0.000
A4GNT	0.314	0.339	0.025	0.566	0.581
AAAS	4.229	4.933	0.704	0.000	0.000
AACS	1.172	2.112	0.940	0.000	0.000
AADAC	8.818	8.220	-0.598	0.000	0.000
AADAT	4.812	2.828	-1.984	0.000	0.000
AAGAB	3.502	4.473	0.971	0.000	0.000
AAK1	1.836	2.298	0.462	0.000	0.000
AAMP	5.949	6.463	0.514	0.000	0.000
AANAT	0.102	0.184	0.082	0.000	0.000
AARS	5.720	6.432	0.712	0.000	0.000
AARS2	2.809	3.902	1.093	0.000	0.000
AARSD1	2.747	3.538	0.791	0.000	0.000
AASDH	2.360	2.609	0.249	0.000	0.000
AASDHPPT	3.609	4.021	0.412	0.000	0.000
AASS	4.914	4.057	-0.857	0.000	0.000
AATK	1.062	1.110	0.048	0.348	0.364
ABAT	6.843	5.804	-1.039	0.000	0.000
ABCA1	4.448	4.316	-0.133	0.007	0.008
ABCA10	2.293	1.539	-0.754	0.000	0.000
ABCA2	4.010	4.282	0.271	0.000	0.000
ABCA3	0.984	1.543	0.559	0.000	0.000
ABCA4	0.647	1.001	0.354	0.000	0.000
ABCA5	3.659	3.991	0.332	0.000	0.000
ABCA6	4.755	4.034	-0.721	0.000	0.000
ABCA7	2.180	2.706	0.525	0.000	0.000
ABCA8	4.143	2.816	-1.327	0.000	0.000
ABCA9	2.968	2.037	-0.931	0.000	0.000
ABCB1	4.673	4.661	-0.012	0.883	0.887
ABCB10	3.534	4.368	0.834	0.000	0.000
ABCB11	4.160	3.623	-0.537	0.000	0.000
ABCB4	6.214	5.213	-1.001	0.000	0.000
ABCB6	2.954	3.727	0.774	0.000	0.000
ABCB7	3.932	4.294	0.362	0.000	0.000
ABCB8	3.189	4.047	0.858	0.000	0.000
ABCB9	0.467	0.880	0.414	0.000	0.000
ABCC1	2.102	2.511	0.409	0.000	0.000
ABCC10	1.860	2.931	1.070	0.000	0.000
ABCC11	1.193	1.237	0.044	0.284	0.298
ABCC2	6.000	5.908	-0.092	0.226	0.239
ABCC3	5.533	5.663	0.131	0.052	0.057
ABCC4	1.201	2.286	1.085	0.000	0.000
ABCC5	1.720	2.719	0.999	0.000	0.000
ABCC6	5.607	5.533	-0.075	0.186	0.198

ABCC8	0.137	0.289	0.153	0.000	0.000
ABCC9	3.427	2.803	-0.623	0.000	0.000
ABCD1	2.951	4.221	1.271	0.000	0.000
ABCD2	0.286	0.301	0.015	0.479	0.495
ABCD3	5.726	5.567	-0.159	0.000	0.000
ABCD4	3.857	4.008	0.152	0.000	0.000
ABCE1	4.011	4.346	0.335	0.000	0.000
ABCF1	4.928	5.810	0.883	0.000	0.000
ABCF2	3.190	4.344	1.154	0.000	0.000
ABCF3	4.605	5.136	0.531	0.000	0.000
ABCG1	2.192	2.815	0.624	0.000	0.000
ABCG2	4.021	3.268	-0.752	0.000	0.000
ABCG5	5.704	5.078	-0.626	0.000	0.000
ABCG8	5.662	5.264	-0.399	0.000	0.000
ABHD1	1.442	1.467	0.024	0.657	0.669
ABHD10	4.636	4.919	0.283	0.000	0.000
ABHD11	3.688	4.489	0.801	0.000	0.000
ABHD12	4.630	5.741	1.111	0.000	0.000
ABHD12B	0.232	0.443	0.211	0.000	0.000
ABHD13	3.069	3.222	0.153	0.000	0.000
ABHD14A	3.727	4.308	0.581	0.000	0.000
ABHD14A-AC	2.971	3.001	0.030	0.318	0.333
ABHD14B	6.642	6.442	-0.200	0.000	0.000
ABHD15	4.470	4.062	-0.408	0.000	0.000
ABHD16A	3.128	3.924	0.796	0.000	0.000
ABHD2	5.942	5.618	-0.324	0.000	0.000
ABHD3	4.253	5.003	0.750	0.000	0.000
ABHD4	3.615	4.754	1.139	0.000	0.000
ABHD5	3.670	3.875	0.204	0.000	0.000
ABHD6	5.012	4.528	-0.484	0.000	0.000
ABHD8	3.911	4.507	0.596	0.000	0.000
ABI1	4.085	4.897	0.812	0.000	0.000
ABI2	1.720	2.234	0.515	0.000	0.000
ABI3	3.012	2.964	-0.048	0.347	0.363
ABI3BP	1.767	1.152	-0.615	0.000	0.000
ABL1	3.284	4.096	0.812	0.000	0.000
ABL2	1.824	2.655	0.831	0.000	0.000
ABLIM1	3.918	4.305	0.387	0.000	0.000
ABLIM2	0.956	1.590	0.635	0.000	0.000
ABLIM3	5.229	4.484	-0.745	0.000	0.000
ABO	0.187	0.272	0.085	0.002	0.002
ABR	2.201	2.297	0.096	0.105	0.113
ABT1	4.044	4.871	0.827	0.000	0.000
ABTB1	3.418	3.837	0.419	0.000	0.000
ABTB2	3.160	3.442	0.281	0.000	0.000
ACAA1	7.804	6.675	-1.128	0.000	0.000
ACAA2	8.332	7.130	-1.202	0.000	0.000
ACACB	4.651	4.012	-0.638	0.000	0.000
ACAD10	3.885	4.076	0.191	0.000	0.000
ACAD8	3.706	3.794	0.088	0.003	0.003
ACAD9	4.331	4.967	0.636	0.000	0.000
ACADL	3.945	2.530	-1.415	0.000	0.000

ACADM	6.612	5.974	-0.638	0.000	0.000
ACADS	7.186	5.965	-1.221	0.000	0.000
ACADSB	7.296	6.231	-1.065	0.000	0.000
ACADVL	8.986	8.391	-0.596	0.000	0.000
ACAN	0.048	0.502	0.454	0.000	0.000
ACAP1	2.554	2.145	-0.409	0.000	0.000
ACAP2	2.598	3.142	0.543	0.000	0.000
ACAP3	3.331	3.778	0.447	0.000	0.000
ACAT1	7.958	7.095	-0.863	0.000	0.000
ACAT2	5.587	5.594	0.007	0.880	0.884
ACBD3	4.294	5.271	0.976	0.000	0.000
ACBD4	5.578	5.044	-0.533	0.000	0.000
ACBD5	5.093	5.278	0.185	0.000	0.000
ACBD7	0.315	0.796	0.481	0.000	0.000
ACCS	3.283	3.226	-0.057	0.300	0.315
ACD	2.592	3.413	0.821	0.000	0.000
ACE2	1.945	1.640	-0.305	0.001	0.001
ACER1	0.420	0.186	-0.234	0.000	0.000
ACER2	1.233	1.183	-0.050	0.203	0.216
ACER3	1.793	2.300	0.507	0.000	0.000
ACHE	1.928	1.849	-0.078	0.302	0.317
ACIN1	4.491	5.183	0.692	0.000	0.000
ACLY	4.133	5.608	1.475	0.000	0.000
ACMSD	6.270	5.333	-0.937	0.000	0.000
ACO1	5.806	5.605	-0.202	0.000	0.000
ACO2	5.590	6.109	0.518	0.000	0.000
ACOT1	3.979	3.715	-0.265	0.000	0.000
ACOT11	0.775	0.684	-0.091	0.014	0.016
ACOT12	6.111	4.856	-1.255	0.000	0.000
ACOT13	5.613	5.568	-0.046	0.200	0.212
ACOT2	5.506	5.164	-0.342	0.000	0.000
ACOT4	3.584	3.604	0.020	0.634	0.647
ACOT6	1.360	1.138	-0.222	0.000	0.000
ACOT7	3.610	4.273	0.663	0.000	0.000
ACOT9	3.193	3.881	0.688	0.000	0.000
ACOX1	6.336	5.962	-0.375	0.000	0.000
ACOX2	7.017	6.139	-0.878	0.000	0.000
ACOX3	3.857	3.997	0.140	0.000	0.000
ACP1	5.452	6.052	0.600	0.000	0.000
ACP2	5.544	5.904	0.360	0.000	0.000
ACP5	4.727	4.889	0.162	0.050	0.055
ACP6	2.171	2.990	0.819	0.000	0.000
ACPP	0.196	0.257	0.061	0.021	0.023
ACR	0.613	0.688	0.075	0.012	0.013
ACRBP	1.169	1.433	0.264	0.000	0.000
ACSF2	4.933	5.276	0.342	0.000	0.000
ACSF3	2.834	2.763	-0.071	0.024	0.027
ACSL1	8.933	7.501	-1.432	0.000	0.000
ACSL3	4.410	5.197	0.788	0.000	0.000
ACSL4	3.024	5.905	2.881	0.000	0.000
ACSL5	6.575	5.903	-0.673	0.000	0.000
ACSL6	1.052	1.710	0.658	0.000	0.000

ACSM1	3.016	4.155	1.139	0.000	0.000
ACSM2A	7.162	5.796	-1.366	0.000	0.000
ACSM2B	6.962	6.128	-0.835	0.000	0.000
ACSM3	5.315	3.597	-1.718	0.000	0.000
ACSM5	6.711	5.050	-1.661	0.000	0.000
ACSS1	2.021	2.636	0.614	0.000	0.000
ACSS2	5.356	5.633	0.277	0.000	0.000
ACSS3	3.548	3.702	0.154	0.034	0.037
ACTA1	0.260	0.575	0.315	0.000	0.000
ACTA2	5.357	6.248	0.891	0.000	0.000
ACTB	10.387	10.919	0.532	0.000	0.000
ACTG1	9.436	9.960	0.523	0.000	0.000
ACTG2	0.823	2.081	1.258	0.000	0.000
ACTL6A	3.593	4.599	1.006	0.000	0.000
ACTL6B	0.075	0.194	0.118	0.000	0.000
ACTN1	4.721	5.172	0.451	0.000	0.000
ACTN2	0.410	1.288	0.878	0.000	0.000
ACTN4	6.173	7.135	0.961	0.000	0.000
ACTR10	4.377	4.996	0.618	0.000	0.000
ACTR1A	4.780	5.527	0.746	0.000	0.000
ACTR1B	4.988	5.624	0.636	0.000	0.000
ACTR2	5.729	6.488	0.758	0.000	0.000
ACTR3	4.079	4.896	0.817	0.000	0.000
ACTR3B	1.808	2.342	0.534	0.000	0.000
ACTR3C	2.004	1.978	-0.026	0.436	0.452
ACTR5	2.576	3.429	0.853	0.000	0.000
ACTR6	3.247	4.037	0.790	0.000	0.000
ACTR8	2.799	3.438	0.638	0.000	0.000
ACVR1	3.686	4.142	0.456	0.000	0.000
ACVR1B	3.958	4.465	0.506	0.000	0.000
ACVR1C	0.959	0.710	-0.250	0.000	0.000
ACVR2A	2.505	2.608	0.103	0.001	0.002
ACVR2B	1.445	1.646	0.201	0.000	0.000
ACVRL1	2.289	2.897	0.608	0.000	0.000
ACY1	5.796	5.478	-0.317	0.000	0.000
ACY3	5.179	4.536	-0.643	0.000	0.000
ACYP2	3.288	3.068	-0.220	0.000	0.000
ADA	2.173	3.029	0.857	0.000	0.000
ADAL	1.911	2.260	0.349	0.000	0.000
ADAM10	3.006	3.837	0.830	0.000	0.000
ADAM11	0.379	0.559	0.180	0.000	0.000
ADAM12	0.246	0.576	0.329	0.000	0.000
ADAM15	3.845	5.242	1.397	0.000	0.000
ADAM17	2.185	2.781	0.597	0.000	0.000
ADAM19	2.395	2.473	0.078	0.105	0.113
ADAM20	0.129	0.128	-0.002	0.822	0.829
ADAM21	0.150	0.282	0.132	0.000	0.000
ADAM22	0.205	0.509	0.304	0.000	0.000
ADAM23	0.244	0.822	0.578	0.000	0.000
ADAM28	0.932	0.966	0.035	0.500	0.515
ADAM33	0.668	0.698	0.031	0.462	0.478
ADAM8	2.331	2.432	0.101	0.064	0.069

ADAM9	2.830	3.938	1.108	0.000	0.000
ADAMDEC1	0.563	1.358	0.795	0.000	0.000
ADAMTS1	4.091	3.258	-0.833	0.000	0.000
ADAMTS10	2.611	2.921	0.311	0.000	0.000
ADAMTS12	0.712	0.839	0.127	0.001	0.002
ADAMTS13	4.338	2.258	-2.080	0.000	0.000
ADAMTS14	0.386	0.831	0.445	0.000	0.000
ADAMTS15	0.737	0.757	0.020	0.605	0.619
ADAMTS16	0.209	0.771	0.562	0.000	0.000
ADAMTS17	1.682	1.969	0.287	0.000	0.000
ADAMTS2	3.274	2.428	-0.845	0.000	0.000
ADAMTS3	0.304	0.424	0.120	0.000	0.000
ADAMTS4	2.123	2.167	0.044	0.487	0.502
ADAMTS5	1.142	1.265	0.123	0.002	0.003
ADAMTS6	0.293	0.670	0.377	0.000	0.000
ADAMTS7	0.798	1.335	0.537	0.000	0.000
ADAMTS8	0.296	0.244	-0.052	0.002	0.002
ADAMTS9	1.624	2.388	0.763	0.000	0.000
ADAMTSL1	0.616	0.733	0.118	0.000	0.000
ADAMTSL2	4.614	2.837	-1.777	0.000	0.000
ADAMTSL3	2.507	2.006	-0.501	0.000	0.000
ADAMTSL4	3.940	4.184	0.244	0.000	0.000
ADAMTSL5	1.481	1.957	0.476	0.000	0.000
ADAP1	1.715	1.572	-0.143	0.028	0.031
ADAP2	2.982	2.799	-0.183	0.000	0.001
ADAR	5.379	6.456	1.077	0.000	0.000
ADARB1	1.910	2.075	0.165	0.000	0.000
ADAT1	2.665	3.180	0.515	0.000	0.000
ADAT2	1.731	2.176	0.445	0.000	0.000
ADAT3	1.557	1.845	0.288	0.000	0.000
ADCK1	2.022	2.627	0.605	0.000	0.000
ADCK2	3.365	4.520	1.156	0.000	0.000
ADCK5	3.104	4.053	0.949	0.000	0.000
ADCY1	2.538	1.423	-1.115	0.000	0.000
ADCY10	1.828	1.826	-0.002	0.972	0.973
ADCY3	2.089	2.530	0.441	0.000	0.000
ADCY4	1.547	1.897	0.350	0.000	0.000
ADCY5	1.512	1.305	-0.207	0.000	0.000
ADCY6	2.779	3.814	1.035	0.000	0.000
ADCY7	1.751	1.813	0.062	0.208	0.220
ADCY9	2.829	3.431	0.602	0.000	0.000
ADCYAP1	0.313	0.237	-0.076	0.004	0.004
ADCYAP1R1	0.192	0.218	0.026	0.193	0.205
ADD1	4.873	5.444	0.570	0.000	0.000
ADD3	3.755	3.926	0.171	0.006	0.006
ADH1A	10.090	7.941	-2.149	0.000	0.000
ADH1B	10.548	8.195	-2.353	0.000	0.000
ADH1C	10.166	7.860	-2.306	0.000	0.000
ADH4	11.003	7.691	-3.312	0.000	0.000
ADH5	6.681	6.844	0.163	0.000	0.000
ADH6	7.757	6.401	-1.356	0.000	0.000
ADH7	0.244	0.251	0.007	0.769	0.779

ADHFE1	5.231	4.775	-0.455	0.000	0.000
ADI1	8.111	7.516	-0.595	0.000	0.000
ADIPOR1	5.926	6.887	0.962	0.000	0.000
ADIPOR2	5.737	5.776	0.040	0.305	0.320
ADK	5.755	5.215	-0.540	0.000	0.000
ADM	4.273	4.078	-0.196	0.006	0.007
ADM2	1.258	2.851	1.593	0.000	0.000
ADNP	3.798	4.677	0.878	0.000	0.000
ADNP2	2.579	3.130	0.551	0.000	0.000
ADO	3.078	3.475	0.397	0.000	0.000
ADORA1	0.654	0.951	0.296	0.000	0.000
ADORA2B	0.683	1.025	0.342	0.000	0.000
ADORA3	1.876	1.417	-0.459	0.000	0.000
ADPGK	3.868	4.357	0.488	0.000	0.000
ADPRH	1.735	2.126	0.390	0.000	0.000
ADPRHL1	1.269	1.891	0.622	0.000	0.000
ADPRHL2	4.357	5.088	0.731	0.000	0.000
ADRA1A	3.532	1.458	-2.074	0.000	0.000
ADRA1B	3.268	2.231	-1.036	0.000	0.000
ADRA1D	0.183	0.405	0.222	0.000	0.000
ADRA2A	1.121	1.098	-0.023	0.714	0.726
ADRA2C	0.995	1.816	0.821	0.000	0.000
ADRB1	0.786	0.454	-0.332	0.000	0.000
ADRB2	2.838	2.451	-0.387	0.000	0.000
ADSL	3.742	4.774	1.032	0.000	0.000
ADSS	3.872	4.795	0.923	0.000	0.000
ADSSL1	3.348	3.036	-0.312	0.000	0.000
AEBP1	5.017	4.279	-0.739	0.000	0.000
AEBP2	2.718	3.079	0.361	0.000	0.000
AEN	3.020	3.604	0.584	0.000	0.000
AES	6.705	6.947	0.242	0.000	0.000
AFAP1	1.164	1.738	0.574	0.000	0.000
AFAP1L1	2.170	2.827	0.657	0.000	0.000
AFAP1L2	1.263	1.303	0.040	0.316	0.331
AFF1	3.773	3.680	-0.093	0.024	0.026
AFF3	1.211	1.721	0.509	0.000	0.000
AFF4	4.414	4.531	0.118	0.001	0.002
AFG3L2	4.434	4.885	0.451	0.000	0.000
AFM	9.036	6.772	-2.264	0.000	0.000
AFMID	6.097	6.336	0.239	0.000	0.000
AFP	2.731	4.455	1.724	0.000	0.000
AFTPH	3.820	4.343	0.523	0.000	0.000
AGA	3.870	4.357	0.487	0.000	0.000
AGAP1	1.394	2.055	0.661	0.000	0.000
AGAP2	1.069	1.353	0.284	0.000	0.000
AGAP3	3.772	4.456	0.684	0.000	0.000
AGAP5	0.963	1.206	0.243	0.000	0.000
AGAP6	2.806	3.057	0.251	0.000	0.000
AGBL2	1.579	1.671	0.093	0.085	0.092
AGBL3	0.800	1.406	0.607	0.000	0.000
AGBL4	0.373	0.117	-0.256	0.000	0.000
AGBL5	2.926	3.961	1.035	0.000	0.000

AGER	2.489	2.647	0.158	0.004	0.005
AGFG1	3.442	4.355	0.913	0.000	0.000
AGFG2	5.014	5.553	0.539	0.000	0.000
AGGF1	3.417	4.154	0.737	0.000	0.000
AGK	2.778	3.487	0.709	0.000	0.000
AGL	4.281	3.651	-0.630	0.000	0.000
AGMAT	6.221	5.861	-0.360	0.000	0.000
AGMO	5.353	5.036	-0.317	0.000	0.000
AGPAT2	7.612	6.978	-0.634	0.000	0.000
AGPAT3	4.453	5.067	0.614	0.000	0.000
AGPAT4	0.906	1.566	0.661	0.000	0.000
AGPAT5	3.179	3.733	0.554	0.000	0.000
AGPS	3.498	4.110	0.611	0.000	0.000
AGR2	0.656	1.475	0.819	0.000	0.000
AGRN	4.322	5.177	0.854	0.000	0.000
AGRP	0.213	0.408	0.196	0.000	0.000
AGT	10.540	10.922	0.382	0.000	0.000
AGTPBP1	2.144	2.625	0.481	0.000	0.000
AGTR1	5.639	4.695	-0.943	0.000	0.000
AGTRAP	3.651	4.502	0.851	0.000	0.000
AGXT	10.153	8.687	-1.466	0.000	0.000
AGXT2	5.361	3.995	-1.366	0.000	0.000
AHCTF1	3.043	4.025	0.982	0.000	0.000
AHCY	6.751	7.357	0.605	0.000	0.000
AHCYL1	5.349	5.797	0.449	0.000	0.000
AHCYL2	2.417	2.895	0.477	0.000	0.000
AHDC1	2.781	2.655	-0.126	0.003	0.003
AHI1	2.299	2.302	0.003	0.950	0.953
AHNAK	5.533	5.997	0.464	0.000	0.000
AHNAK2	0.311	0.716	0.405	0.000	0.000
AHR	4.786	4.970	0.184	0.001	0.001
AHRR	0.374	0.681	0.307	0.000	0.000
AHSA1	5.365	6.266	0.901	0.000	0.000
AHSG	11.702	10.720	-0.983	0.000	0.000
AIDA	3.712	4.762	1.050	0.000	0.000
AIF1	5.386	5.114	-0.272	0.000	0.000
AIF1L	1.356	2.088	0.732	0.000	0.000
AIFM1	6.134	6.332	0.198	0.000	0.000
AIFM2	3.309	4.452	1.143	0.000	0.000
AIFM3	0.664	1.231	0.567	0.000	0.000
AIG1	6.247	5.977	-0.269	0.000	0.000
AIM2	1.123	0.942	-0.181	0.001	0.001
AIMP1	4.606	5.288	0.683	0.000	0.000
AIMP2	4.695	5.499	0.804	0.000	0.000
AIP	5.601	6.136	0.534	0.000	0.000
AK1	4.230	4.657	0.427	0.000	0.000
AK2	5.813	6.093	0.280	0.000	0.000
AK3	6.415	6.220	-0.194	0.000	0.000
AK4	5.205	5.358	0.154	0.007	0.008
AK7	0.637	0.852	0.215	0.000	0.000
AK8	0.242	0.625	0.383	0.000	0.000
AKAP1	5.063	5.651	0.588	0.000	0.000

AKAP10	2.766	2.994	0.229	0.000	0.000
AKAP11	2.897	3.046	0.149	0.000	0.000
AKAP12	3.278	2.930	-0.348	0.000	0.000
AKAP13	3.804	3.968	0.163	0.000	0.000
AKAP2	2.355	2.242	-0.113	0.000	0.000
AKAP3	0.601	0.487	-0.113	0.000	0.000
AKAP5	0.723	0.907	0.183	0.000	0.000
AKAP6	0.755	0.799	0.044	0.062	0.068
AKAP7	3.508	3.310	-0.198	0.000	0.000
AKAP8	3.557	4.094	0.537	0.000	0.000
AKAP8L	4.294	4.966	0.672	0.000	0.000
AKAP9	3.971	3.936	-0.035	0.459	0.475
AKIP1	3.108	4.333	1.225	0.000	0.000
AKIRIN1	5.085	5.282	0.198	0.000	0.000
AKIRIN2	5.097	5.579	0.481	0.000	0.000
AKNA	2.946	2.777	-0.170	0.001	0.001
AKR1A1	7.293	7.425	0.132	0.000	0.000
AKR1B1	3.487	3.878	0.391	0.000	0.000
AKR1B10	3.732	7.691	3.960	0.000	0.000
AKR1B15	0.306	1.528	1.222	0.000	0.000
AKR1C1	6.701	7.116	0.416	0.000	0.000
AKR1C2	5.451	6.543	1.093	0.000	0.000
AKR1C3	5.824	7.777	1.953	0.000	0.000
AKR1C4	8.097	7.451	-0.646	0.000	0.000
AKR1D1	6.782	4.531	-2.251	0.000	0.000
AKR1E2	0.631	0.693	0.061	0.108	0.116
AKR7A2	5.613	6.055	0.442	0.000	0.000
AKR7A3	7.740	5.562	-2.178	0.000	0.000
AKT1	4.908	4.998	0.090	0.013	0.014
AKT1S1	4.082	4.985	0.903	0.000	0.000
AKT2	4.552	4.874	0.322	0.000	0.000
AKT3	1.774	1.900	0.126	0.019	0.021
AKTIP	3.756	3.787	0.032	0.368	0.384
ALAD	6.680	6.506	-0.173	0.001	0.001
ALAS1	8.237	7.605	-0.633	0.000	0.000
ALAS2	0.314	0.214	-0.100	0.000	0.000
ALB	16.617	14.657	-1.960	0.000	0.000
ALCAM	4.627	5.428	0.801	0.000	0.000
ALDH16A1	3.892	4.407	0.514	0.000	0.000
ALDH18A1	4.078	5.157	1.079	0.000	0.000
ALDH1A1	9.570	9.783	0.213	0.003	0.003
ALDH1A2	1.537	1.557	0.019	0.712	0.723
ALDH1A3	1.758	1.273	-0.486	0.000	0.000
ALDH1B1	6.980	6.146	-0.834	0.000	0.000
ALDH1L1	6.623	5.861	-0.762	0.000	0.000
ALDH1L2	0.406	0.563	0.158	0.000	0.000
ALDH2	8.936	7.617	-1.320	0.000	0.000
ALDH3A1	1.134	2.865	1.731	0.000	0.000
ALDH3A2	6.758	6.942	0.184	0.000	0.000
ALDH3B1	1.964	2.584	0.621	0.000	0.000
ALDH4A1	7.622	7.193	-0.429	0.000	0.000
ALDH5A1	5.979	5.689	-0.290	0.000	0.000

ALDH6A1	6.789	5.443	-1.347	0.000	0.000
ALDH7A1	6.164	6.082	-0.081	0.071	0.077
ALDH8A1	7.178	5.521	-1.657	0.000	0.000
ALDH9A1	6.917	6.724	-0.193	0.000	0.000
ALDOA	6.296	7.490	1.193	0.000	0.000
ALDOB	12.638	10.537	-2.101	0.000	0.000
ALDOC	4.929	5.146	0.217	0.009	0.010
ALG1	3.332	4.245	0.913	0.000	0.000
ALG10	1.046	1.437	0.391	0.000	0.000
ALG10B	1.051	1.347	0.296	0.000	0.000
ALG11	1.443	1.742	0.299	0.000	0.000
ALG12	3.004	3.873	0.869	0.000	0.000
ALG13	3.291	3.785	0.494	0.000	0.000
ALG14	1.983	2.514	0.532	0.000	0.000
ALG1L	1.099	3.563	2.464	0.000	0.000
ALG1L2	0.135	0.168	0.033	0.005	0.006
ALG2	4.873	5.269	0.396	0.000	0.000
ALG3	4.682	5.802	1.119	0.000	0.000
ALG5	5.054	5.514	0.460	0.000	0.000
ALG6	2.581	3.445	0.864	0.000	0.000
ALG8	4.570	5.458	0.888	0.000	0.000
ALKBH1	2.731	3.072	0.341	0.000	0.000
ALKBH2	3.959	4.879	0.921	0.000	0.000
ALKBH3	3.565	3.755	0.190	0.000	0.000
ALKBH4	2.885	3.644	0.760	0.000	0.000
ALKBH5	5.432	5.760	0.328	0.000	0.000
ALKBH6	2.030	2.865	0.835	0.000	0.000
ALKBH7	6.492	6.466	-0.026	0.479	0.495
ALKBH8	2.022	2.252	0.231	0.000	0.000
ALLC	1.362	0.547	-0.815	0.000	0.000
ALMS1	1.584	2.422	0.839	0.000	0.000
ALOX15	0.156	0.303	0.147	0.000	0.000
ALOX15B	0.378	0.925	0.546	0.000	0.000
ALOX5	2.482	2.329	-0.153	0.049	0.053
ALOX5AP	2.643	2.505	-0.138	0.073	0.080
ALPK1	1.855	1.929	0.074	0.067	0.072
ALPK2	1.574	2.134	0.560	0.000	0.000
ALPK3	1.242	2.149	0.907	0.000	0.000
ALPL	5.584	4.089	-1.495	0.000	0.000
ALS2	3.090	3.442	0.352	0.000	0.000
ALS2CL	2.694	3.615	0.921	0.000	0.000
ALS2CR12	0.490	0.531	0.041	0.049	0.053
ALX3	0.341	0.916	0.575	0.000	0.000
AMACR	4.742	4.789	0.047	0.535	0.550
AMBP	12.994	12.236	-0.758	0.000	0.000
AMBRA1	2.721	3.503	0.782	0.000	0.000
AMD1	3.901	4.299	0.398	0.000	0.000
AMDHD1	6.828	5.740	-1.088	0.000	0.000
AMDHD2	3.201	4.188	0.987	0.000	0.000
AMFR	5.787	5.992	0.205	0.000	0.000
AMH	0.304	0.807	0.502	0.000	0.000
AMHR2	0.627	0.122	-0.505	0.000	0.000

AMIGO1	1.340	1.726	0.385	0.000	0.000
AMIGO2	2.223	2.120	-0.103	0.103	0.111
AMIGO3	1.039	1.591	0.552	0.000	0.000
AMMECR1	1.792	2.404	0.612	0.000	0.000
AMMECR1L	3.249	3.756	0.507	0.000	0.000
AMN	4.091	3.310	-0.780	0.000	0.000
AMOT	1.322	1.359	0.037	0.463	0.479
AMOTL1	2.592	2.749	0.157	0.019	0.022
AMOTL2	3.745	3.355	-0.389	0.000	0.000
AMPD1	0.523	0.228	-0.295	0.000	0.000
AMPD2	4.166	4.688	0.522	0.000	0.000
AMPD3	1.182	1.502	0.321	0.000	0.000
AMPH	0.327	0.296	-0.031	0.214	0.227
AMT	5.128	4.991	-0.137	0.002	0.003
AMY2B	2.788	2.741	-0.047	0.401	0.417
AMZ1	0.251	0.313	0.061	0.029	0.032
AMZ2	4.060	4.802	0.743	0.000	0.000
ANAPC10	2.617	2.895	0.277	0.000	0.000
ANAPC11	5.335	6.338	1.003	0.000	0.000
ANAPC13	5.405	5.850	0.445	0.000	0.000
ANAPC16	5.712	5.893	0.181	0.000	0.000
ANAPC2	4.067	4.595	0.528	0.000	0.000
ANAPC4	2.739	3.509	0.771	0.000	0.000
ANAPC5	4.163	4.939	0.776	0.000	0.000
ANAPC7	3.323	4.461	1.138	0.000	0.000
ANG	10.178	8.765	-1.412	0.000	0.000
ANGEL1	2.989	3.488	0.500	0.000	0.000
ANGEL2	2.740	3.611	0.871	0.000	0.000
ANGPT1	0.790	1.407	0.617	0.000	0.000
ANGPT2	1.079	1.914	0.835	0.000	0.000
ANGPTL1	3.266	1.864	-1.402	0.000	0.000
ANGPTL2	3.279	3.166	-0.114	0.072	0.078
ANGPTL3	8.613	7.634	-0.980	0.000	0.000
ANGPTL4	6.773	5.972	-0.801	0.000	0.000
ANGPTL6	4.693	3.125	-1.568	0.000	0.000
ANK2	0.724	0.656	-0.068	0.044	0.049
ANK3	1.801	1.130	-0.671	0.000	0.000
ANKAR	0.899	1.314	0.416	0.000	0.000
ANKDD1A	1.102	1.351	0.249	0.000	0.000
ANKFY1	2.924	3.050	0.127	0.001	0.001
ANKH	4.323	4.670	0.347	0.000	0.000
ANKHD1	3.050	3.547	0.497	0.000	0.000
ANKHD1-EIF4	2.239	2.622	0.383	0.000	0.000
ANKIB1	2.917	3.624	0.708	0.000	0.000
ANKK1	0.314	0.323	0.009	0.658	0.670
ANKLE1	0.350	0.573	0.222	0.000	0.000
ANKLE2	2.756	3.413	0.657	0.000	0.000
ANKMY1	1.411	2.028	0.617	0.000	0.000
ANKMY2	3.297	3.868	0.572	0.000	0.000
ANKRA2	3.576	4.137	0.561	0.000	0.000
ANKRD1	1.135	1.633	0.498	0.000	0.000
ANKRD10	4.925	5.336	0.410	0.000	0.000

ANKRD11	3.156	3.521	0.366	0.000	0.000
ANKRD12	3.651	3.747	0.096	0.028	0.031
ANKRD13A	3.414	4.049	0.636	0.000	0.000
ANKRD13B	0.709	1.451	0.742	0.000	0.000
ANKRD13C	2.832	3.311	0.479	0.000	0.000
ANKRD13D	2.659	3.320	0.661	0.000	0.000
ANKRD16	2.059	2.818	0.758	0.000	0.000
ANKRD17	4.021	4.166	0.145	0.001	0.001
ANKRD2	0.253	0.476	0.223	0.000	0.000
ANKRD22	1.231	1.577	0.347	0.000	0.000
ANKRD23	0.849	1.257	0.408	0.000	0.000
ANKRD24	2.738	2.158	-0.580	0.000	0.000
ANKRD26	1.705	2.099	0.394	0.000	0.000
ANKRD27	1.855	3.059	1.204	0.000	0.000
ANKRD28	3.120	3.547	0.427	0.000	0.000
ANKRD29	1.204	2.355	1.151	0.000	0.000
ANKRD31	0.133	0.192	0.059	0.000	0.000
ANKRD33B	0.492	0.536	0.044	0.173	0.184
ANKRD35	1.094	1.367	0.273	0.000	0.000
ANKRD36	0.783	1.071	0.288	0.000	0.000
ANKRD36B	0.684	0.876	0.192	0.000	0.000
ANKRD37	3.277	2.935	-0.343	0.000	0.000
ANKRD39	2.176	3.127	0.951	0.000	0.000
ANKRD40	3.693	4.360	0.668	0.000	0.000
ANKRD42	1.470	1.628	0.158	0.000	0.000
ANKRD44	1.690	1.767	0.077	0.052	0.057
ANKRD45	0.154	0.445	0.291	0.000	0.000
ANKRD46	3.869	3.967	0.098	0.015	0.016
ANKRD49	2.717	3.422	0.706	0.000	0.000
ANKRD50	2.119	2.528	0.409	0.000	0.000
ANKRD52	2.383	3.520	1.137	0.000	0.000
ANKRD53	0.660	0.588	-0.072	0.019	0.022
ANKRD54	3.085	3.895	0.809	0.000	0.000
ANKRD55	1.415	0.529	-0.886	0.000	0.000
ANKRD6	1.926	2.367	0.442	0.000	0.000
ANKRD9	2.516	2.920	0.404	0.000	0.000
ANKS1A	3.202	3.168	-0.034	0.392	0.408
ANKS1B	0.540	0.989	0.449	0.000	0.000
ANKS3	2.085	2.740	0.654	0.000	0.000
ANKS4B	3.901	3.428	-0.473	0.000	0.000
ANKS6	1.501	2.355	0.854	0.000	0.000
ANKZF1	3.489	4.384	0.895	0.000	0.000
ANLN	0.560	2.831	2.271	0.000	0.000
ANO1	4.814	4.097	-0.717	0.000	0.000
ANO10	2.496	3.701	1.205	0.000	0.000
ANO2	0.181	0.472	0.291	0.000	0.000
ANO3	0.504	0.441	-0.064	0.079	0.086
ANO4	0.077	0.269	0.192	0.000	0.000
ANO5	1.477	1.637	0.160	0.003	0.003
ANO6	4.501	5.156	0.656	0.000	0.000
ANO7	0.740	1.126	0.386	0.000	0.000
ANO8	2.227	2.891	0.665	0.000	0.000

ANO9	1.364	1.530	0.166	0.019	0.021
ANP32A	4.746	5.586	0.840	0.000	0.000
ANP32B	6.357	6.938	0.582	0.000	0.000
ANP32E	4.413	5.361	0.948	0.000	0.000
ANPEP	7.874	7.843	-0.031	0.592	0.606
ANTXR1	2.294	2.446	0.153	0.039	0.043
ANTXR2	3.573	2.956	-0.617	0.000	0.000
ANXA1	4.174	4.323	0.149	0.027	0.030
ANXA10	5.778	3.628	-2.150	0.000	0.000
ANXA11	4.888	5.688	0.800	0.000	0.000
ANXA13	3.437	3.226	-0.211	0.041	0.046
ANXA2	4.937	6.711	1.774	0.000	0.000
ANXA3	1.699	0.979	-0.721	0.000	0.000
ANXA4	5.543	5.804	0.261	0.000	0.000
ANXA5	6.496	7.082	0.587	0.000	0.000
ANXA6	6.389	6.503	0.114	0.016	0.018
ANXA7	6.378	6.906	0.529	0.000	0.000
ANXA9	4.807	5.486	0.678	0.000	0.000
AOAH	2.672	2.302	-0.370	0.000	0.000
AOC2	1.355	1.494	0.139	0.000	0.000
AOC3	3.531	3.418	-0.113	0.015	0.017
AOX1	8.674	7.334	-1.340	0.000	0.000
AP1AR	3.937	4.093	0.155	0.000	0.000
AP1B1	4.438	5.147	0.709	0.000	0.000
AP1G1	4.075	4.204	0.129	0.000	0.000
AP1G2	3.280	3.593	0.313	0.000	0.000
AP1M1	3.683	4.378	0.694	0.000	0.000
AP1M2	1.648	2.517	0.869	0.000	0.000
AP1S1	5.170	6.370	1.199	0.000	0.000
AP1S2	2.430	2.759	0.329	0.000	0.000
AP1S3	0.809	1.431	0.622	0.000	0.000
AP2A1	4.460	5.257	0.797	0.000	0.000
AP2A2	4.141	4.567	0.425	0.000	0.000
AP2B1	4.373	5.160	0.787	0.000	0.000
AP2M1	6.120	6.921	0.801	0.000	0.000
AP3B1	3.039	4.174	1.134	0.000	0.000
AP3D1	4.663	5.494	0.831	0.000	0.000
AP3M1	3.587	4.300	0.713	0.000	0.000
AP3M2	1.685	2.538	0.853	0.000	0.000
AP3S1	4.824	5.648	0.824	0.000	0.000
AP3S2	3.174	3.579	0.406	0.000	0.000
AP4B1	2.567	3.112	0.546	0.000	0.000
AP4E1	1.763	2.360	0.597	0.000	0.000
AP4M1	1.964	2.909	0.945	0.000	0.000
AP4S1	1.351	1.412	0.062	0.010	0.011
APAF1	1.802	2.482	0.680	0.000	0.000
APBA1	2.476	2.087	-0.388	0.000	0.000
APBA2	0.789	0.759	-0.030	0.486	0.501
APBA3	2.759	3.389	0.630	0.000	0.000
APBB1	2.263	2.907	0.643	0.000	0.000
APBB1IP	3.893	3.681	-0.212	0.002	0.002
APBB2	2.853	3.192	0.339	0.000	0.000

APBB3	3.614	4.258	0.644	0.000	0.000
APC	2.229	2.592	0.363	0.000	0.000
APCDD1	1.634	1.198	-0.436	0.000	0.000
APCS	11.295	10.104	-1.191	0.000	0.000
APEH	5.495	6.003	0.508	0.000	0.000
APEX1	6.453	7.352	0.899	0.000	0.000
APH1A	6.283	7.364	1.082	0.000	0.000
APH1B	1.739	2.382	0.643	0.000	0.000
API5	4.942	5.360	0.418	0.000	0.000
APIP	3.003	4.049	1.046	0.000	0.000
APLF	0.824	1.215	0.391	0.000	0.000
APLN	0.680	2.355	1.674	0.000	0.000
APLNR	3.388	3.254	-0.134	0.079	0.086
APLP1	0.491	1.239	0.748	0.000	0.000
APLP2	7.282	7.712	0.430	0.000	0.000
APOA1	14.285	12.485	-1.799	0.000	0.000
APOA2	14.140	14.262	0.123	0.262	0.276
APOA4	4.592	3.174	-1.418	0.000	0.000
APOA5	8.961	6.861	-2.100	0.000	0.000
APOB	9.587	9.289	-0.299	0.000	0.000
APOBEC2	0.434	0.566	0.132	0.000	0.000
APOBEC3A	0.735	0.643	-0.092	0.009	0.011
APOBEC3B	0.922	2.290	1.368	0.000	0.000
APOBEC3C	2.687	2.451	-0.236	0.000	0.000
APOBEC3D	1.421	1.540	0.119	0.022	0.025
APOBEC3F	1.841	2.030	0.189	0.000	0.000
APOBEC3G	1.989	2.006	0.017	0.755	0.765
APOBEC3H	1.018	1.204	0.186	0.000	0.000
APOBR	2.264	2.262	-0.001	0.975	0.976
APOC1	13.474	12.870	-0.604	0.000	0.000
APOC2	9.310	9.272	-0.038	0.621	0.635
APOC3	14.028	12.511	-1.516	0.000	0.000
APOC4	6.190	5.120	-1.070	0.000	0.000
APOC4-APOC	7.275	6.668	-0.607	0.000	0.000
APOD	1.238	1.470	0.232	0.001	0.001
APOE	12.685	12.909	0.224	0.000	0.000
APOF	8.404	5.132	-3.272	0.000	0.000
APOH	13.258	12.331	-0.927	0.000	0.000
APOL1	7.320	6.782	-0.538	0.000	0.000
APOL2	5.310	5.768	0.459	0.000	0.000
APOL3	4.593	4.643	0.050	0.470	0.485
APOL4	1.305	1.665	0.360	0.000	0.000
APOL5	0.615	0.892	0.277	0.000	0.000
APOLD1	2.201	3.109	0.908	0.000	0.000
APOM	8.476	8.098	-0.378	0.000	0.000
APOO	4.047	4.774	0.727	0.000	0.000
APOOL	2.778	3.373	0.595	0.000	0.000
APP	7.042	7.210	0.168	0.008	0.009
APPBP2	2.667	3.123	0.456	0.000	0.000
APPL1	3.052	3.747	0.696	0.000	0.000
APPL2	3.375	4.043	0.667	0.000	0.000
APRT	6.328	6.832	0.505	0.000	0.000

APTX	3.745	4.344	0.599	0.000	0.000
AQP11	3.510	3.636	0.126	0.014	0.015
AQP3	6.166	5.107	-1.060	0.000	0.000
AQP4	0.832	0.579	-0.253	0.000	0.000
AQP5	0.124	0.271	0.147	0.000	0.000
AQP6	0.271	0.528	0.257	0.000	0.000
AQP7	3.646	2.641	-1.005	0.000	0.000
AQP8	0.473	1.462	0.988	0.000	0.000
AQP9	8.246	7.097	-1.149	0.000	0.000
AQR	2.293	2.856	0.563	0.000	0.000
AR	3.478	2.720	-0.758	0.000	0.000
ARAF	5.509	5.742	0.233	0.000	0.000
ARAP1	4.683	5.192	0.509	0.000	0.000
ARAP2	2.288	1.947	-0.341	0.000	0.000
ARAP3	2.161	2.458	0.297	0.000	0.000
ARC	0.349	0.344	-0.006	0.829	0.836
ARCN1	5.813	6.221	0.407	0.000	0.000
AREG	0.817	0.655	-0.162	0.001	0.002
ARF1	7.523	8.523	1.001	0.000	0.000
ARF3	4.815	5.586	0.771	0.000	0.000
ARF4	6.783	7.407	0.624	0.000	0.000
ARF5	6.491	7.196	0.705	0.000	0.000
ARF6	5.434	5.847	0.413	0.000	0.000
ARFGAP1	4.294	5.168	0.874	0.000	0.000
ARFGAP2	5.188	5.586	0.398	0.000	0.000
ARFGAP3	4.881	5.418	0.538	0.000	0.000
ARFGEF1	3.183	4.000	0.817	0.000	0.000
ARFGEF2	3.467	4.427	0.961	0.000	0.000
ARFIP1	3.545	3.943	0.398	0.000	0.000
ARFIP2	4.099	4.788	0.689	0.000	0.000
ARFRP1	3.397	4.249	0.852	0.000	0.000
ARG1	9.314	8.100	-1.214	0.000	0.000
ARG2	2.418	3.007	0.589	0.000	0.000
ARGLU1	4.930	5.293	0.363	0.000	0.000
ARHGAP1	4.277	4.919	0.642	0.000	0.000
ARHGAP10	2.143	1.398	-0.745	0.000	0.000
ARHGAP11A	0.748	2.322	1.574	0.000	0.000
ARHGAP11B	0.364	0.719	0.355	0.000	0.000
ARHGAP12	3.850	4.475	0.625	0.000	0.000
ARHGAP15	2.146	1.688	-0.458	0.000	0.000
ARHGAP17	3.011	3.571	0.560	0.000	0.000
ARHGAP18	2.277	3.009	0.732	0.000	0.000
ARHGAP19	1.679	2.438	0.759	0.000	0.000
ARHGAP20	0.840	0.662	-0.179	0.000	0.000
ARHGAP21	3.199	3.684	0.485	0.000	0.000
ARHGAP22	0.532	1.002	0.471	0.000	0.000
ARHGAP24	2.073	1.997	-0.077	0.088	0.095
ARHGAP25	2.653	2.490	-0.163	0.001	0.001
ARHGAP26	1.833	2.211	0.378	0.000	0.000
ARHGAP27	2.225	2.530	0.305	0.000	0.000
ARHGAP28	0.206	0.483	0.277	0.000	0.000
ARHGAP29	3.267	3.387	0.119	0.009	0.010

ARHGAP30	2.928	2.763	-0.166	0.005	0.006
ARHGAP31	1.618	1.769	0.152	0.000	0.000
ARHGAP32	2.037	2.194	0.158	0.000	0.000
ARHGAP33	1.548	2.426	0.877	0.000	0.000
ARHGAP35	4.122	4.640	0.518	0.000	0.000
ARHGAP39	0.910	1.772	0.861	0.000	0.000
ARHGAP4	3.617	4.040	0.424	0.000	0.000
ARHGAP42	2.237	2.115	-0.122	0.004	0.005
ARHGAP44	0.756	0.964	0.208	0.000	0.000
ARHGAP5	3.430	3.917	0.487	0.000	0.000
ARHGAP6	0.590	0.808	0.219	0.000	0.000
ARHGAP8	0.610	0.576	-0.033	0.377	0.393
ARHGAP9	2.742	2.329	-0.413	0.000	0.000
ARHGDIA	6.960	7.496	0.536	0.000	0.000
ARHGDIB	6.146	6.246	0.100	0.086	0.093
ARHGEF1	4.401	4.850	0.449	0.000	0.000
ARHGEF10	1.268	1.298	0.031	0.461	0.477
ARHGEF10L	5.432	5.451	0.019	0.708	0.719
ARHGEF11	3.001	4.265	1.264	0.000	0.000
ARHGEF12	4.244	4.375	0.131	0.001	0.001
ARHGEF15	1.883	2.419	0.536	0.000	0.000
ARHGEF16	2.254	2.644	0.389	0.000	0.000
ARHGEF17	2.345	2.619	0.274	0.000	0.000
ARHGEF18	2.448	3.201	0.753	0.000	0.000
ARHGEF19	1.280	1.781	0.501	0.000	0.000
ARHGEF2	2.581	3.553	0.972	0.000	0.000
ARHGEF25	1.911	1.854	-0.057	0.324	0.339
ARHGEF26	4.365	3.598	-0.768	0.000	0.000
ARHGEF3	2.068	2.735	0.667	0.000	0.000
ARHGEF33	0.180	0.181	0.001	0.928	0.931
ARHGEF35	1.870	2.393	0.524	0.000	0.000
ARHGEF37	1.361	2.201	0.840	0.000	0.000
ARHGEF38	0.241	0.429	0.187	0.000	0.000
ARHGEF4	0.363	0.312	-0.050	0.090	0.098
ARHGEF40	4.453	4.897	0.445	0.000	0.000
ARHGEF5	2.353	2.891	0.539	0.000	0.000
ARHGEF6	2.440	2.458	0.018	0.718	0.729
ARHGEF7	3.331	3.663	0.333	0.000	0.000
ARHGEF9	1.965	2.508	0.544	0.000	0.000
ARID1A	3.644	4.218	0.574	0.000	0.000
ARID1B	2.396	2.814	0.418	0.000	0.000
ARID2	1.950	2.503	0.553	0.000	0.000
ARID3A	1.161	2.344	1.183	0.000	0.000
ARID3B	1.416	2.033	0.617	0.000	0.000
ARID3C	2.949	2.000	-0.949	0.000	0.000
ARID4A	3.766	3.558	-0.208	0.000	0.000
ARID4B	3.073	3.737	0.664	0.000	0.000
ARID5A	4.223	4.222	-0.001	0.987	0.987
ARID5B	2.559	2.706	0.147	0.003	0.003
ARIH1	2.639	3.187	0.548	0.000	0.000
ARIH2	3.920	4.652	0.732	0.000	0.000
ARL1	4.831	5.494	0.662	0.000	0.000

ARL10	0.610	0.665	0.055	0.036	0.040
ARL11	0.759	0.754	-0.005	0.886	0.890
ARL13B	2.110	2.467	0.357	0.000	0.000
ARL14	1.328	1.166	-0.162	0.053	0.058
ARL15	2.742	3.575	0.832	0.000	0.000
ARL16	4.160	4.875	0.715	0.000	0.000
ARL17A	0.382	0.819	0.437	0.000	0.000
ARL17B	0.668	0.847	0.180	0.000	0.000
ARL2	3.497	4.602	1.105	0.000	0.000
ARL2BP	3.590	4.349	0.759	0.000	0.000
ARL3	2.735	3.289	0.554	0.000	0.000
ARL4A	2.966	3.968	1.002	0.000	0.000
ARL4C	3.896	3.757	-0.139	0.047	0.052
ARL4D	5.342	5.383	0.040	0.541	0.555
ARL5A	3.573	4.008	0.434	0.000	0.000
ARL5B	3.762	3.812	0.050	0.336	0.351
ARL6	1.264	1.584	0.319	0.000	0.000
ARL6IP1	7.298	7.345	0.047	0.223	0.236
ARL6IP4	4.414	4.872	0.458	0.000	0.000
ARL6IP5	5.600	6.023	0.423	0.000	0.000
ARL6IP6	2.118	2.906	0.788	0.000	0.000
ARL8A	5.302	5.880	0.579	0.000	0.000
ARL8B	4.873	5.643	0.770	0.000	0.000
ARL9	0.225	0.300	0.075	0.002	0.002
ARMC1	3.892	5.057	1.165	0.000	0.000
ARMC10	3.771	4.265	0.494	0.000	0.000
ARMC2	0.695	1.139	0.444	0.000	0.000
ARMC5	3.407	3.235	-0.172	0.000	0.000
ARMC6	5.074	4.738	-0.336	0.000	0.000
ARMC7	3.034	3.798	0.764	0.000	0.000
ARMC8	2.612	3.187	0.575	0.000	0.000
ARMC9	0.516	1.081	0.565	0.000	0.000
ARMCX1	2.338	2.443	0.105	0.065	0.071
ARMCX2	1.811	1.755	-0.056	0.316	0.331
ARMCX3	4.153	3.545	-0.608	0.000	0.000
ARMCX4	1.160	1.315	0.155	0.000	0.000
ARMCX5	2.182	2.693	0.510	0.000	0.000
ARMCX6	2.732	2.594	-0.138	0.031	0.034
ARMS2	0.171	0.223	0.052	0.001	0.001
ARNT	4.290	4.955	0.664	0.000	0.000
ARNT2	0.743	1.057	0.314	0.000	0.000
ARNTL	2.955	3.018	0.063	0.195	0.207
ARNTL2	1.454	1.847	0.393	0.000	0.000
ARPC1A	5.739	6.895	1.155	0.000	0.000
ARPC1B	5.244	6.363	1.119	0.000	0.000
ARPC2	5.339	6.138	0.799	0.000	0.000
ARPC3	6.214	7.112	0.898	0.000	0.000
ARPC4	5.440	6.166	0.726	0.000	0.000
ARPC4-TTLL3	1.908	2.295	0.387	0.000	0.000
ARPC5	4.723	5.939	1.216	0.000	0.000
ARPC5L	4.352	4.874	0.523	0.000	0.000
ARPP19	4.322	5.170	0.848	0.000	0.000

ARPP21	0.501	0.394	-0.107	0.002	0.002
ARRB1	3.125	2.978	-0.147	0.008	0.009
ARRB2	4.440	4.883	0.443	0.000	0.000
ARRDC1	4.361	4.613	0.252	0.000	0.000
ARRDC2	4.232	4.869	0.638	0.000	0.000
ARRDC3	5.212	5.295	0.083	0.151	0.161
ARRDC4	4.175	3.825	-0.350	0.000	0.000
ARRDC5	0.391	0.369	-0.022	0.322	0.337
ARSA	5.240	5.857	0.617	0.000	0.000
ARSB	2.445	2.878	0.432	0.000	0.000
ARSD	4.667	4.508	-0.159	0.001	0.001
ARSE	6.109	6.077	-0.032	0.692	0.704
ARSF	1.157	0.921	-0.236	0.001	0.001
ARSG	1.028	1.445	0.417	0.000	0.000
ARSI	0.537	0.712	0.175	0.000	0.000
ARSJ	1.474	1.301	-0.173	0.001	0.001
ARSK	1.878	2.384	0.506	0.000	0.000
ART3	0.197	0.213	0.016	0.532	0.546
ART4	3.335	2.764	-0.571	0.000	0.000
ART5	0.367	0.398	0.031	0.414	0.430
ARTN	0.348	0.739	0.391	0.000	0.000
ARV1	4.112	5.013	0.902	0.000	0.000
ARVCF	3.348	3.181	-0.166	0.000	0.000
AS3MT	3.511	3.841	0.330	0.000	0.000
ASAH1	5.183	5.593	0.411	0.000	0.000
ASAH2	0.599	0.853	0.254	0.000	0.000
ASAH2B	1.284	1.364	0.080	0.000	0.000
ASAP1	2.576	3.550	0.974	0.000	0.000
ASAP2	1.316	2.107	0.792	0.000	0.000
ASAP3	2.623	3.571	0.948	0.000	0.000
ASB1	2.935	3.258	0.323	0.000	0.000
ASB12	0.177	0.192	0.015	0.267	0.281
ASB13	5.371	5.270	-0.100	0.037	0.041
ASB14	0.648	0.857	0.209	0.000	0.000
ASB16	0.616	1.349	0.732	0.000	0.000
ASB2	0.773	0.747	-0.026	0.483	0.499
ASB4	0.752	0.883	0.130	0.009	0.010
ASB6	2.889	3.612	0.723	0.000	0.000
ASB7	2.415	2.882	0.467	0.000	0.000
ASB8	3.936	4.387	0.451	0.000	0.000
ASB9	3.174	3.323	0.149	0.024	0.027
ASCC1	2.846	3.238	0.393	0.000	0.000
ASCC2	4.860	5.414	0.554	0.000	0.000
ASCC3	2.756	3.530	0.774	0.000	0.000
ASCL1	0.602	0.703	0.102	0.153	0.163
ASCL2	1.085	0.855	-0.230	0.000	0.000
ASF1A	3.445	4.215	0.770	0.000	0.000
ASF1B	1.131	3.171	2.040	0.000	0.000
ASGR1	8.806	8.168	-0.638	0.000	0.000
ASGR2	9.305	8.579	-0.726	0.000	0.000
ASH1L	2.702	3.491	0.788	0.000	0.000
ASH2L	3.404	3.743	0.339	0.000	0.000

ASIP	0.930	0.856	-0.075	0.038	0.042
ASL	7.630	7.026	-0.604	0.000	0.000
ASMTL	2.215	2.205	-0.010	0.651	0.664
ASNA1	5.122	6.224	1.102	0.000	0.000
ASNS	1.873	2.750	0.877	0.000	0.000
ASNSD1	4.928	5.420	0.491	0.000	0.000
ASPA	2.188	1.108	-1.080	0.000	0.000
ASPDH	7.078	4.904	-2.174	0.000	0.000
ASPG	5.339	2.629	-2.711	0.000	0.000
ASPH	3.653	4.946	1.293	0.000	0.000
ASPHD1	1.081	1.901	0.820	0.000	0.000
ASPHD2	0.648	0.868	0.220	0.000	0.000
ASPM	0.550	2.734	2.184	0.000	0.000
ASPN	4.484	3.648	-0.835	0.000	0.000
ASPRV1	1.110	1.078	-0.032	0.255	0.268
ASPSCR1	3.663	4.567	0.903	0.000	0.000
ASRGL1	1.248	2.494	1.245	0.000	0.000
ASS1	10.767	8.762	-2.006	0.000	0.000
ASTE1	2.298	2.653	0.356	0.000	0.000
ASTN2	0.944	1.354	0.410	0.000	0.000
ASXL1	3.917	4.608	0.690	0.000	0.000
ASXL2	1.864	2.390	0.527	0.000	0.000
ASXL3	0.259	0.192	-0.066	0.000	0.000
ATAD1	4.012	4.337	0.326	0.000	0.000
ATAD2	2.715	4.450	1.735	0.000	0.000
ATAD2B	1.883	2.262	0.379	0.000	0.000
ATAD3A	3.651	4.256	0.606	0.000	0.000
ATAD3B	2.492	3.114	0.622	0.000	0.000
ATAD3C	1.819	1.172	-0.647	0.000	0.000
ATAD5	0.687	1.428	0.741	0.000	0.000
ATAT1	1.934	2.796	0.862	0.000	0.000
ATE1	2.939	3.444	0.505	0.000	0.000
ATF1	3.610	4.117	0.506	0.000	0.000
ATF2	3.320	4.017	0.698	0.000	0.000
ATF3	5.457	4.638	-0.818	0.000	0.000
ATF4	7.749	8.203	0.454	0.000	0.000
ATF5	8.833	7.416	-1.417	0.000	0.000
ATF6	3.798	4.548	0.749	0.000	0.000
ATF6B	4.831	5.900	1.069	0.000	0.000
ATF7	2.693	3.279	0.586	0.000	0.000
ATF7IP	2.465	2.881	0.416	0.000	0.000
ATF7IP2	3.760	3.970	0.210	0.000	0.000
ATG10	1.633	2.347	0.714	0.000	0.000
ATG12	3.484	4.143	0.659	0.000	0.000
ATG13	4.087	4.923	0.836	0.000	0.000
ATG14	2.824	3.471	0.647	0.000	0.000
ATG16L1	3.300	3.771	0.471	0.000	0.000
ATG2A	4.258	4.025	-0.234	0.000	0.000
ATG2B	2.936	3.010	0.074	0.056	0.061
ATG3	3.867	4.526	0.659	0.000	0.000
ATG4A	3.720	4.149	0.429	0.000	0.000
ATG4B	4.110	4.671	0.561	0.000	0.000

ATG4C	2.893	3.396	0.503	0.000	0.000
ATG4D	3.571	4.037	0.467	0.000	0.000
ATG5	3.989	4.273	0.284	0.000	0.000
ATG7	2.474	3.299	0.826	0.000	0.000
ATG9A	4.009	4.764	0.754	0.000	0.000
ATG9B	0.699	0.786	0.087	0.007	0.008
ATIC	4.898	5.946	1.048	0.000	0.000
ATL1	0.645	0.827	0.182	0.000	0.000
ATL2	4.495	5.145	0.650	0.000	0.000
ATL3	3.018	3.795	0.778	0.000	0.000
ATM	2.425	2.756	0.331	0.000	0.000
ATMIN	3.616	3.877	0.261	0.000	0.000
ATN1	4.425	5.097	0.672	0.000	0.000
ATOH7	1.041	0.656	-0.385	0.000	0.000
ATOH8	3.910	2.169	-1.741	0.000	0.000
ATOX1	5.622	6.490	0.867	0.000	0.000
ATP10A	0.698	0.909	0.210	0.000	0.000
ATP10D	1.914	2.132	0.218	0.000	0.000
ATP11A	2.557	3.245	0.688	0.000	0.000
ATP11B	3.086	3.482	0.395	0.000	0.000
ATP11C	4.058	3.571	-0.487	0.000	0.000
ATP13A1	3.794	4.677	0.883	0.000	0.000
ATP13A2	2.556	3.396	0.841	0.000	0.000
ATP13A3	5.086	5.385	0.299	0.000	0.000
ATP13A4	0.572	0.242	-0.330	0.000	0.000
ATP1A1	6.246	7.348	1.102	0.000	0.000
ATP1A2	0.566	0.891	0.325	0.000	0.000
ATP1B1	6.809	7.973	1.164	0.000	0.000
ATP1B2	1.794	1.504	-0.290	0.000	0.000
ATP1B3	3.959	5.007	1.049	0.000	0.000
ATP2A1	0.656	0.998	0.342	0.000	0.000
ATP2A2	4.534	5.578	1.045	0.000	0.000
ATP2A3	2.551	2.348	-0.203	0.002	0.003
ATP2B1	3.159	3.668	0.509	0.000	0.000
ATP2B2	3.755	4.085	0.331	0.000	0.000
ATP2B4	3.526	4.282	0.755	0.000	0.000
ATP2C1	3.501	4.375	0.874	0.000	0.000
ATP2C2	0.362	0.261	-0.101	0.004	0.005
ATP6AP1	4.982	6.281	1.299	0.000	0.000
ATP6AP1L	0.963	1.303	0.341	0.000	0.000
ATP6AP2	5.968	6.766	0.798	0.000	0.000
ATP6V0A1	3.885	4.669	0.784	0.000	0.000
ATP6V0A2	1.736	2.217	0.481	0.000	0.000
ATP6V0B	5.924	6.756	0.832	0.000	0.000
ATP6V0C	5.527	6.132	0.605	0.000	0.000
ATP6V0D1	4.266	4.957	0.690	0.000	0.000
ATP6V0D2	0.112	0.827	0.715	0.000	0.000
ATP6V0E1	7.011	7.819	0.808	0.000	0.000
ATP6V0E2	5.342	5.923	0.581	0.000	0.000
ATP6V1A	4.496	5.199	0.704	0.000	0.000
ATP6V1B1	0.275	0.659	0.384	0.000	0.000
ATP6V1B2	4.178	4.497	0.319	0.000	0.000

ATP6V1C1	3.832	5.317	1.486	0.000	0.000
ATP6V1C2	2.565	3.189	0.624	0.000	0.000
ATP6V1D	4.337	5.043	0.705	0.000	0.000
ATP6V1E1	5.291	6.315	1.024	0.000	0.000
ATP6V1E2	0.852	1.439	0.587	0.000	0.000
ATP6V1F	6.327	7.519	1.193	0.000	0.000
ATP6V1G1	6.961	7.445	0.484	0.000	0.000
ATP6V1G2	0.586	0.768	0.182	0.000	0.000
ATP6V1G2-DI	2.885	3.221	0.336	0.000	0.000
ATP6V1H	3.562	4.494	0.932	0.000	0.000
ATP7A	1.339	1.774	0.435	0.000	0.000
ATP7B	3.178	3.264	0.086	0.078	0.085
ATP8A1	1.340	1.507	0.168	0.000	0.000
ATP8B1	3.030	4.040	1.010	0.000	0.000
ATP8B2	2.051	2.714	0.663	0.000	0.000
ATP8B3	0.557	0.967	0.410	0.000	0.000
ATP8B4	1.444	1.207	-0.237	0.000	0.000
ATP9A	2.974	3.900	0.926	0.000	0.000
ATP9B	1.791	2.312	0.521	0.000	0.000
ATPAF1	5.052	5.157	0.105	0.000	0.001
ATPAF2	3.162	3.433	0.271	0.000	0.000
ATR	2.501	3.165	0.664	0.000	0.000
ATRIP	1.189	1.862	0.673	0.000	0.000
ATRN	4.643	5.560	0.917	0.000	0.000
ATRNL1	0.849	0.801	-0.048	0.367	0.383
ATRX	2.709	3.136	0.427	0.000	0.000
ATXN1	2.340	2.750	0.409	0.000	0.000
ATXN10	5.206	5.824	0.617	0.000	0.000
ATXN1L	2.660	3.179	0.519	0.000	0.000
ATXN2	2.865	3.499	0.634	0.000	0.000
ATXN2L	4.548	5.341	0.793	0.000	0.000
ATXN3	1.405	1.696	0.291	0.000	0.000
ATXN7	2.740	2.935	0.195	0.000	0.000
ATXN7L1	1.807	2.089	0.282	0.000	0.000
ATXN7L2	1.360	2.029	0.669	0.000	0.000
ATXN7L3	3.745	4.778	1.033	0.000	0.000
ATXN7L3B	4.142	4.893	0.751	0.000	0.000
AUH	4.382	4.593	0.212	0.000	0.000
AUP1	6.705	7.403	0.698	0.000	0.000
AURKA	1.642	4.128	2.486	0.000	0.000
AURKAIP1	6.936	7.202	0.266	0.000	0.000
AURKB	0.952	3.212	2.261	0.000	0.000
AURKC	1.461	1.368	-0.094	0.009	0.010
AUTS2	3.253	2.889	-0.364	0.000	0.000
AVEN	3.828	4.219	0.391	0.000	0.000
AVIL	1.955	2.059	0.104	0.075	0.082
AVL9	1.954	2.817	0.863	0.000	0.000
AVPI1	5.488	5.007	-0.481	0.000	0.000
AVPR1A	2.343	1.358	-0.985	0.000	0.000
AVPR2	0.177	0.332	0.155	0.000	0.000
AXDND1	0.050	0.245	0.195	0.000	0.000
AXIN1	2.665	3.307	0.642	0.000	0.000

AXIN2	1.445	2.127	0.682	0.000	0.000
AXL	3.224	2.579	-0.646	0.000	0.000
AZGP1	10.736	9.140	-1.596	0.000	0.000
AZIN1	4.847	5.834	0.987	0.000	0.000
AZU1	0.190	0.164	-0.026	0.089	0.096
B2M	11.115	11.196	0.081	0.122	0.131
B3GALNT1	0.914	1.670	0.757	0.000	0.000
B3GALNT2	1.900	2.956	1.056	0.000	0.000
B3GALT2	0.584	0.602	0.018	0.709	0.720
B3GALT4	1.569	1.807	0.238	0.000	0.000
B3GALT6	3.522	4.241	0.719	0.000	0.000
B3GAT1	1.717	0.655	-1.062	0.000	0.000
B3GAT2	0.494	0.683	0.189	0.000	0.000
B3GAT3	4.689	5.584	0.895	0.000	0.000
B3GNT2	3.699	4.155	0.456	0.000	0.000
B3GNT3	1.839	2.649	0.810	0.000	0.000
B3GNT4	0.248	0.373	0.125	0.000	0.000
B3GNT5	1.115	2.216	1.101	0.000	0.000
B3GNT7	1.419	1.294	-0.124	0.033	0.036
B3GNT8	0.943	1.109	0.166	0.001	0.001
B3GNT9	2.272	2.417	0.146	0.004	0.005
B3GNTL1	1.152	2.013	0.861	0.000	0.000
B4GALNT1	0.307	1.417	1.110	0.000	0.000
B4GALNT3	0.585	0.606	0.020	0.667	0.680
B4GALNT4	0.414	0.790	0.376	0.000	0.000
B4GALT1	5.998	6.210	0.212	0.000	0.000
B4GALT2	4.244	4.866	0.622	0.000	0.000
B4GALT3	3.815	4.933	1.118	0.000	0.000
B4GALT4	2.901	3.476	0.574	0.000	0.000
B4GALT5	4.112	4.568	0.456	0.000	0.000
B4GALT6	1.061	1.696	0.636	0.000	0.000
B4GALT7	3.234	4.202	0.968	0.000	0.000
B9D1	2.173	3.099	0.926	0.000	0.000
B9D2	2.248	2.580	0.332	0.000	0.000
BAALC	1.421	1.463	0.042	0.515	0.530
BAAT	8.977	8.482	-0.495	0.000	0.000
BABAM1	4.777	5.560	0.783	0.000	0.000
BACE1	3.937	4.224	0.287	0.000	0.000
BACE2	2.150	2.371	0.221	0.014	0.016
BACH1	3.513	3.885	0.372	0.000	0.000
BACH2	1.232	0.919	-0.313	0.000	0.000
BAG1	4.481	4.752	0.271	0.000	0.000
BAG2	2.250	3.344	1.093	0.000	0.000
BAG3	4.462	5.062	0.600	0.000	0.000
BAG4	3.254	3.494	0.241	0.000	0.000
BAG5	3.234	3.740	0.506	0.000	0.000
BAG6	6.370	7.147	0.777	0.000	0.000
BAHD1	3.330	3.743	0.413	0.000	0.000
BAIAP2	4.385	4.225	-0.160	0.001	0.001
BAIAP2L1	4.323	5.176	0.853	0.000	0.000
BAIAP2L2	1.698	3.362	1.664	0.000	0.000
BAIAP3	2.725	2.780	0.055	0.264	0.278

BAK1	2.981	4.183	1.202	0.000	0.000
BAMBI	4.308	4.878	0.569	0.000	0.000
BANK1	0.597	0.520	-0.076	0.051	0.056
BANP	2.122	2.654	0.532	0.000	0.000
BAP1	4.628	5.475	0.846	0.000	0.000
BARD1	0.759	1.399	0.639	0.000	0.000
BARX1	0.222	0.548	0.326	0.000	0.000
BASP1	3.625	2.840	-0.784	0.000	0.000
BATF	2.499	2.906	0.408	0.000	0.000
BATF2	2.456	2.477	0.021	0.698	0.710
BATF3	1.346	1.283	-0.064	0.126	0.135
BAX	4.746	5.803	1.057	0.000	0.000
BAZ1A	3.294	3.666	0.373	0.000	0.000
BAZ1B	4.079	4.851	0.772	0.000	0.000
BAZ2A	3.674	4.245	0.571	0.000	0.000
BAZ2B	2.543	2.467	-0.076	0.045	0.049
BBC3	2.320	3.203	0.883	0.000	0.000
BBIP1	2.728	3.043	0.316	0.000	0.000
BBOX1	4.854	2.899	-1.955	0.000	0.000
BBS1	1.528	1.908	0.380	0.000	0.000
BBS10	2.717	3.280	0.563	0.000	0.000
BBS12	0.801	1.039	0.238	0.000	0.000
BBS2	2.790	3.218	0.427	0.000	0.000
BBS4	2.899	3.607	0.707	0.000	0.000
BBS5	1.670	1.621	-0.048	0.180	0.192
BBS7	1.362	1.902	0.539	0.000	0.000
BBS9	1.739	2.100	0.362	0.000	0.000
BBX	2.690	3.164	0.474	0.000	0.000
BCAM	4.925	6.088	1.163	0.000	0.000
BCAN	0.199	0.904	0.704	0.000	0.000
BCAP29	3.563	4.307	0.744	0.000	0.000
BCAP31	6.808	8.070	1.261	0.000	0.000
BCAR1	3.791	4.122	0.331	0.000	0.000
BCAR3	3.097	3.462	0.365	0.000	0.000
BCAS1	0.235	0.882	0.647	0.000	0.000
BCAS2	5.378	5.847	0.469	0.000	0.000
BCAS3	2.087	2.676	0.589	0.000	0.000
BCAS4	0.518	1.301	0.783	0.000	0.000
BCAT1	0.695	1.490	0.795	0.000	0.000
BCCIP	4.649	5.087	0.438	0.000	0.000
BCHE	6.437	4.533	-1.904	0.000	0.000
BCKDHA	4.068	3.930	-0.139	0.000	0.001
BCKDHB	5.016	4.183	-0.833	0.000	0.000
BCKDK	5.278	5.358	0.080	0.015	0.017
BCL10	3.062	3.533	0.472	0.000	0.000
BCL11A	0.356	0.529	0.173	0.000	0.000
BCL11B	0.893	0.828	-0.066	0.093	0.101
BCL2	1.516	1.629	0.113	0.016	0.018
BCL2A1	2.459	2.365	-0.094	0.231	0.244
BCL2L1	5.797	6.412	0.615	0.000	0.000
BCL2L10	2.894	2.731	-0.163	0.129	0.139
BCL2L11	3.060	3.690	0.630	0.000	0.000

BCL2L12	3.672	4.708	1.036	0.000	0.000
BCL2L13	3.969	4.369	0.400	0.000	0.000
BCL2L14	0.575	0.799	0.224	0.000	0.000
BCL2L15	0.268	0.509	0.241	0.000	0.000
BCL2L2	3.342	3.929	0.587	0.000	0.000
BCL2L2-PABP	2.812	3.234	0.422	0.000	0.000
BCL3	5.748	5.557	-0.191	0.002	0.002
BCL6	4.657	4.689	0.032	0.540	0.555
BCL6B	2.025	2.862	0.837	0.000	0.000
BCL7A	2.179	2.964	0.785	0.000	0.000
BCL7B	4.462	5.054	0.592	0.000	0.000
BCL9	1.839	3.190	1.351	0.000	0.000
BCL9L	2.272	3.022	0.749	0.000	0.000
BCLAF1	4.225	4.611	0.386	0.000	0.000
BCO2	4.590	1.996	-2.594	0.000	0.000
BCOR	2.719	3.510	0.791	0.000	0.000
BCORL1	1.610	2.313	0.703	0.000	0.000
BCR	2.945	3.736	0.791	0.000	0.000
BCS1L	4.167	4.782	0.615	0.000	0.000
BDH1	6.352	5.710	-0.642	0.000	0.000
BDH2	5.574	4.395	-1.179	0.000	0.000
BDKRB1	0.313	0.662	0.350	0.000	0.000
BDKRB2	0.982	1.319	0.337	0.000	0.000
BDNF	0.157	0.259	0.102	0.000	0.000
BDP1	2.517	2.926	0.409	0.000	0.000
BEAN1	0.319	0.342	0.023	0.369	0.385
BECN1	3.870	4.682	0.812	0.000	0.000
BEGAIN	0.384	0.391	0.007	0.826	0.833
BEND3	0.698	1.416	0.717	0.000	0.000
BEND5	0.701	0.833	0.132	0.002	0.002
BEND7	1.935	2.457	0.522	0.000	0.000
BEST1	0.872	1.288	0.416	0.000	0.000
BEST4	0.446	0.822	0.376	0.000	0.000
BET1	3.458	4.031	0.573	0.000	0.000
BET1L	4.581	5.263	0.682	0.000	0.000
BEX1	2.421	1.491	-0.930	0.000	0.000
BEX2	1.692	2.809	1.117	0.000	0.000
BEX4	3.569	3.623	0.054	0.434	0.450
BEX5	1.922	2.030	0.108	0.114	0.122
BFAR	4.052	4.742	0.690	0.000	0.000
BFSP1	0.420	1.100	0.679	0.000	0.000
BFSP2	0.099	0.415	0.316	0.000	0.000
BGLAP	1.277	1.897	0.619	0.000	0.000
BGN	8.103	6.693	-1.410	0.000	0.000
BHLHA15	1.275	1.856	0.581	0.000	0.000
BHLHB9	1.130	1.536	0.405	0.000	0.000
BHLHE22	0.863	0.564	-0.299	0.000	0.000
BHLHE40	6.663	6.362	-0.300	0.000	0.000
BHLHE41	1.293	1.599	0.306	0.000	0.000
BHMT	8.920	6.851	-2.069	0.000	0.000
BHMT2	8.003	7.589	-0.414	0.000	0.000
BICC1	1.844	2.124	0.280	0.001	0.002

BICD1	0.462	0.988	0.526	0.000	0.000
BICD2	2.850	3.324	0.474	0.000	0.000
BID	4.294	5.124	0.829	0.000	0.000
BIK	0.949	1.702	0.753	0.000	0.000
BIN1	3.746	4.537	0.792	0.000	0.000
BIN2	2.607	2.278	-0.329	0.000	0.000
BIN3	2.714	2.887	0.174	0.000	0.000
BIRC2	4.879	5.095	0.216	0.000	0.000
BIRC3	3.411	4.164	0.753	0.000	0.000
BIRC5	1.091	3.666	2.575	0.000	0.000
BIRC6	3.377	3.747	0.370	0.000	0.000
BIRC7	0.138	0.558	0.420	0.000	0.000
BIVM	3.555	3.821	0.266	0.000	0.000
BLCAP	4.723	4.944	0.221	0.000	0.000
BLK	0.974	0.462	-0.512	0.000	0.000
BLM	0.461	1.622	1.161	0.000	0.000
BLMH	3.140	3.620	0.480	0.000	0.000
BLNK	4.523	4.191	-0.332	0.000	0.000
BLOC1S1	6.012	6.562	0.550	0.000	0.000
BLOC1S2	4.480	4.984	0.505	0.000	0.000
BLOC1S3	2.580	3.819	1.239	0.000	0.000
BLVRA	3.563	4.897	1.334	0.000	0.000
BLVRB	7.650	7.758	0.108	0.011	0.013
BLZF1	2.796	3.836	1.041	0.000	0.000
BMF	2.193	3.015	0.822	0.000	0.000
BMI1	4.245	5.186	0.940	0.000	0.000
BMP1	3.943	3.787	-0.156	0.001	0.002
BMP2	2.646	3.189	0.543	0.000	0.000
BMP4	1.873	2.920	1.048	0.000	0.000
BMP5	1.278	0.416	-0.862	0.000	0.000
BMP6	2.202	2.222	0.021	0.680	0.693
BMP8A	1.203	1.280	0.077	0.027	0.030
BMP8B	0.677	1.144	0.468	0.000	0.000
BMPER	2.136	0.408	-1.728	0.000	0.000
BMPR1A	1.616	2.200	0.584	0.000	0.000
BMPR1B	0.434	0.263	-0.171	0.000	0.000
BMPR2	3.152	3.344	0.191	0.000	0.000
BMS1	3.115	3.664	0.549	0.000	0.000
BMX	0.923	0.653	-0.270	0.000	0.000
BNC2	0.265	0.326	0.061	0.010	0.012
BNIP1	2.988	3.521	0.532	0.000	0.000
BNIP2	4.050	4.184	0.134	0.000	0.000
BNIP3	6.163	6.274	0.111	0.006	0.007
BNIP3L	4.564	4.576	0.012	0.808	0.816
BNIPL	0.502	0.993	0.492	0.000	0.000
BOC	0.609	0.827	0.218	0.000	0.000
BOD1	4.587	5.532	0.944	0.000	0.000
BOK	6.560	6.147	-0.414	0.000	0.000
BOLA1	4.206	5.221	1.015	0.000	0.000
BOLA2	0.624	0.972	0.349	0.000	0.000
BOLA2B	1.322	1.810	0.488	0.000	0.000
BOLA3	3.141	4.255	1.113	0.000	0.000

BPGM	3.110	4.243	1.133	0.000	0.000
BPHL	5.118	5.211	0.092	0.033	0.037
BPI	0.421	0.308	-0.113	0.000	0.000
BPNT1	4.038	4.997	0.958	0.000	0.000
BPTF	2.911	3.533	0.622	0.000	0.000
BRAF	1.973	2.434	0.461	0.000	0.000
BRAP	2.987	3.624	0.637	0.000	0.000
BRAT1	3.629	4.569	0.940	0.000	0.000
BRCA1	1.072	2.040	0.969	0.000	0.000
BRCA2	0.538	0.974	0.436	0.000	0.000
BRCC3	3.160	3.811	0.651	0.000	0.000
BRD1	3.390	3.696	0.305	0.000	0.000
BRD2	5.863	6.355	0.491	0.000	0.000
BRD3	2.770	3.564	0.794	0.000	0.000
BRD4	3.856	4.128	0.272	0.000	0.000
BRD7	3.223	3.824	0.602	0.000	0.000
BRD8	3.461	4.285	0.824	0.000	0.000
BRD9	2.692	3.516	0.823	0.000	0.000
BRF1	1.871	2.320	0.450	0.000	0.000
BRF2	2.209	2.907	0.697	0.000	0.000
BRI3	5.842	6.504	0.663	0.000	0.000
BRI3BP	1.898	2.675	0.777	0.000	0.000
BRIP1	0.775	1.682	0.907	0.000	0.000
BRIX1	3.024	4.011	0.987	0.000	0.000
BRK1	6.974	7.554	0.580	0.000	0.000
BRMS1	4.393	5.458	1.065	0.000	0.000
BRMS1L	2.162	2.448	0.286	0.000	0.000
BROX	2.966	4.005	1.038	0.000	0.000
BRPF1	2.404	3.041	0.636	0.000	0.000
BRPF3	3.312	4.328	1.015	0.000	0.000
BRSK1	0.619	1.268	0.649	0.000	0.000
BRSK2	0.137	0.389	0.252	0.000	0.000
BRWD1	2.515	2.882	0.367	0.000	0.000
BRWD3	1.650	2.180	0.530	0.000	0.000
BSCL2	3.204	3.787	0.583	0.000	0.000
BSDC1	5.114	5.220	0.106	0.000	0.001
BSG	6.670	7.951	1.282	0.000	0.000
BSN	0.789	0.869	0.080	0.028	0.031
BSPRY	1.268	1.693	0.425	0.000	0.000
BST1	2.052	1.790	-0.262	0.000	0.000
BST2	8.213	8.045	-0.169	0.034	0.037
BTAF1	3.409	3.909	0.500	0.000	0.000
BTBD1	4.330	4.763	0.433	0.000	0.000
BTBD10	2.765	3.527	0.761	0.000	0.000
BTBD11	0.656	0.770	0.114	0.001	0.001
BTBD16	1.942	1.706	-0.236	0.000	0.000
BTBD19	2.034	1.701	-0.334	0.000	0.000
BTBD2	4.172	4.896	0.724	0.000	0.000
BTBD3	2.441	3.241	0.800	0.000	0.000
BTBD6	4.876	4.932	0.056	0.214	0.227
BTBD7	1.939	2.467	0.528	0.000	0.000
BTBD8	0.347	0.479	0.132	0.000	0.000

BTBD9	1.559	1.984	0.425	0.000	0.000
BTC	0.652	0.891	0.239	0.000	0.000
BTD	5.197	5.236	0.039	0.364	0.380
BTF3	7.654	8.264	0.609	0.000	0.000
BTF3L4	3.593	4.090	0.496	0.000	0.000
BTG1	6.452	6.514	0.062	0.168	0.179
BTG2	4.827	4.845	0.017	0.800	0.809
BTG3	3.176	4.249	1.073	0.000	0.000
BTK	1.855	1.722	-0.133	0.008	0.009
BTLA	0.525	0.499	-0.025	0.420	0.436
BTN2A1	3.278	3.950	0.672	0.000	0.000
BTN2A2	2.022	2.824	0.802	0.000	0.000
BTN3A1	3.579	4.195	0.616	0.000	0.000
BTN3A2	3.797	4.148	0.351	0.000	0.000
BTN3A3	3.308	3.530	0.221	0.000	0.000
BTNL8	0.414	1.078	0.664	0.000	0.000
BTNL9	2.196	2.613	0.417	0.000	0.000
BTRC	2.556	2.897	0.341	0.000	0.000
BUB1	0.475	2.320	1.845	0.000	0.000
BUB1B	0.546	2.402	1.855	0.000	0.000
BUB3	3.642	4.695	1.053	0.000	0.000
BUD13	3.407	4.176	0.769	0.000	0.000
BUD31	4.958	5.900	0.942	0.000	0.000
BVES	0.309	0.392	0.083	0.000	0.000
BYSL	3.329	4.399	1.070	0.000	0.000
BZW1	5.646	5.762	0.116	0.001	0.002
BZW2	3.478	4.566	1.088	0.000	0.000
C10orf105	0.368	0.335	-0.034	0.026	0.029
C10orf55	0.424	0.658	0.234	0.000	0.000
C10orf62	0.306	0.401	0.095	0.000	0.000
C10orf88	2.120	2.895	0.776	0.000	0.000
C11orf1	3.655	3.386	-0.270	0.000	0.000
C11orf21	1.049	0.836	-0.213	0.000	0.000
C11orf24	5.559	5.436	-0.124	0.001	0.001
C11orf45	0.479	0.851	0.372	0.000	0.000
C11orf49	1.649	2.393	0.744	0.000	0.000
C11orf52	2.300	2.490	0.190	0.000	0.000
C11orf54	5.880	5.373	-0.508	0.000	0.000
C11orf58	5.009	5.566	0.557	0.000	0.000
C11orf65	0.687	0.600	-0.086	0.000	0.000
C11orf68	4.383	5.277	0.895	0.000	0.000
C11orf74	3.487	4.272	0.784	0.000	0.000
C11orf80	1.648	2.619	0.971	0.000	0.000
C11orf86	0.235	0.275	0.039	0.205	0.218
C11orf91	0.391	0.494	0.103	0.000	0.000
C11orf94	0.419	0.453	0.033	0.157	0.168
C11orf95	2.718	3.249	0.531	0.000	0.000
C11orf96	4.991	3.621	-1.371	0.000	0.000
C12orf10	4.510	5.229	0.719	0.000	0.000
C12orf29	2.876	3.119	0.243	0.000	0.000
C12orf4	2.201	2.890	0.689	0.000	0.000
C12orf43	2.343	2.841	0.498	0.000	0.000

C12orf45	3.407	4.119	0.712	0.000	0.000
C12orf49	1.875	2.816	0.942	0.000	0.000
C12orf60	1.368	1.483	0.115	0.000	0.001
C12orf65	2.507	3.158	0.651	0.000	0.000
C12orf66	2.049	2.184	0.135	0.000	0.000
C12orf71	0.155	0.162	0.007	0.555	0.569
C12orf73	2.166	3.171	1.005	0.000	0.000
C12orf74	0.142	0.202	0.061	0.000	0.000
C12orf75	2.019	3.399	1.380	0.000	0.000
C12orf76	1.965	2.526	0.561	0.000	0.000
C14orf119	4.051	4.775	0.725	0.000	0.000
C14orf180	1.634	0.270	-1.364	0.000	0.000
C14orf28	2.180	2.287	0.107	0.000	0.000
C14orf93	1.996	2.885	0.889	0.000	0.000
C15orf38-AP3S	2.486	2.836	0.349	0.000	0.000
C15orf39	2.086	3.117	1.031	0.000	0.000
C15orf40	1.795	2.345	0.550	0.000	0.000
C15orf41	1.320	2.250	0.929	0.000	0.000
C15orf48	1.525	2.823	1.298	0.000	0.000
C15orf61	1.741	2.479	0.738	0.000	0.000
C15orf62	1.556	1.801	0.246	0.000	0.000
C16orf45	3.586	3.352	-0.234	0.000	0.000
C16orf46	1.247	1.105	-0.142	0.000	0.000
C16orf54	1.464	1.186	-0.278	0.000	0.000
C16orf58	5.425	5.720	0.296	0.000	0.000
C16orf70	4.358	4.365	0.007	0.858	0.864
C16orf71	0.361	0.545	0.184	0.000	0.000
C16orf72	2.851	3.091	0.240	0.000	0.000
C16orf74	0.832	0.968	0.136	0.002	0.003
C16orf86	2.365	2.220	-0.145	0.000	0.000
C16orf87	2.533	2.601	0.068	0.047	0.052
C16orf89	0.531	0.491	-0.040	0.310	0.325
C16orf91	3.489	4.276	0.786	0.000	0.000
C16orf95	1.360	1.451	0.091	0.001	0.001
C17orf107	2.123	2.040	-0.083	0.047	0.052
C17orf47	0.331	0.345	0.014	0.321	0.337
C17orf49	2.906	3.580	0.674	0.000	0.000
C17orf51	0.820	0.992	0.173	0.000	0.000
C17orf53	0.769	1.836	1.067	0.000	0.000
C17orf58	3.421	4.627	1.206	0.000	0.000
C17orf67	1.491	1.395	-0.096	0.063	0.068
C17orf75	2.510	3.341	0.831	0.000	0.000
C17orf80	2.670	3.535	0.864	0.000	0.000
C17orf97	1.409	1.488	0.079	0.101	0.109
C18orf21	3.333	4.148	0.816	0.000	0.000
C18orf25	2.201	2.702	0.501	0.000	0.000
C18orf32	3.502	4.058	0.556	0.000	0.000
C18orf54	0.457	0.911	0.454	0.000	0.000
C19orf12	4.482	4.714	0.231	0.000	0.000
C19orf18	1.400	1.387	-0.013	0.790	0.798
C19orf24	5.413	5.979	0.566	0.000	0.000
C19orf25	3.073	3.957	0.884	0.000	0.000

C19orf33	1.484	1.569	0.084	0.350	0.366
C19orf38	1.859	1.665	-0.194	0.000	0.000
C19orf44	1.970	2.539	0.569	0.000	0.000
C19orf47	2.175	2.890	0.715	0.000	0.000
C19orf48	3.825	5.224	1.399	0.000	0.000
C19orf53	6.476	7.292	0.816	0.000	0.000
C19orf54	2.837	3.416	0.579	0.000	0.000
C19orf57	0.892	1.490	0.598	0.000	0.000
C19orf66	6.208	5.807	-0.401	0.000	0.000
C19orf70	5.440	5.960	0.520	0.000	0.000
C19orf71	2.137	2.119	-0.018	0.741	0.751
C19orf73	1.885	2.300	0.416	0.000	0.000
C1D	3.823	4.144	0.322	0.000	0.000
C1GALT1	3.022	3.628	0.606	0.000	0.000
C1GALT1C1	4.680	5.333	0.653	0.000	0.000
C1orf100	0.074	0.201	0.127	0.000	0.000
C1orf105	0.445	0.824	0.380	0.000	0.000
C1orf109	2.281	3.061	0.780	0.000	0.000
C1orf112	1.316	2.474	1.158	0.000	0.000
C1orf115	6.154	6.510	0.356	0.000	0.000
C1orf116	0.896	1.084	0.187	0.010	0.012
C1orf122	4.786	5.213	0.427	0.000	0.000
C1orf123	4.191	4.568	0.377	0.000	0.000
C1orf127	0.303	0.396	0.093	0.000	0.000
C1orf131	2.615	3.586	0.971	0.000	0.000
C1orf159	1.883	2.458	0.575	0.000	0.000
C1orf162	4.049	3.214	-0.836	0.000	0.000
C1orf174	3.179	3.730	0.551	0.000	0.000
C1orf189	0.284	0.354	0.070	0.000	0.000
C1orf198	3.401	4.715	1.314	0.000	0.000
C1orf21	3.298	3.065	-0.233	0.000	0.000
C1orf210	3.035	2.995	-0.040	0.630	0.644
C1orf216	2.162	3.059	0.896	0.000	0.000
C1orf226	2.626	3.504	0.878	0.000	0.000
C1orf35	2.825	4.083	1.259	0.000	0.000
C1orf43	7.178	8.224	1.046	0.000	0.000
C1orf50	3.500	3.499	-0.001	0.959	0.961
C1orf52	3.121	3.652	0.532	0.000	0.000
C1orf53	4.102	4.583	0.481	0.000	0.000
C1orf54	3.370	3.490	0.120	0.018	0.021
C1orf56	3.312	4.193	0.881	0.000	0.000
C1orf74	0.955	1.759	0.805	0.000	0.000
C1QA	7.855	7.318	-0.537	0.000	0.000
C1QB	7.658	7.027	-0.631	0.000	0.000
C1QBP	5.992	6.334	0.341	0.000	0.000
C1QC	7.518	6.982	-0.536	0.000	0.000
C1QL1	1.013	2.631	1.618	0.000	0.000
C1QL4	0.090	0.419	0.329	0.000	0.000
C1QTNF1	4.639	2.989	-1.650	0.000	0.000
C1QTNF2	0.590	0.621	0.031	0.333	0.349
C1QTNF3	1.966	3.088	1.122	0.000	0.000
C1QTNF4	0.462	0.477	0.015	0.552	0.566

C1QTNF5	2.096	2.312	0.216	0.000	0.000
C1QTNF6	2.431	3.080	0.649	0.000	0.000
C1QTNF7	0.877	0.469	-0.408	0.000	0.000
C1QTNF9	0.339	0.284	-0.055	0.001	0.001
C1QTNF9B	0.187	0.452	0.265	0.000	0.000
C1R	10.260	8.770	-1.490	0.000	0.000
C1RL	6.813	5.935	-0.877	0.000	0.000
C1S	10.298	9.400	-0.899	0.000	0.000
C2	7.697	7.982	0.284	0.000	0.000
C20orf144	0.053	0.216	0.163	0.000	0.000
C20orf194	1.717	2.190	0.473	0.000	0.000
C20orf202	0.298	0.551	0.253	0.000	0.000
C20orf27	3.555	4.785	1.230	0.000	0.000
C20orf96	2.386	3.404	1.018	0.000	0.000
C21orf58	0.646	1.781	1.135	0.000	0.000
C21orf62	0.390	0.120	-0.270	0.000	0.000
C21orf91	2.457	2.079	-0.379	0.000	0.000
C22orf15	0.738	0.775	0.037	0.116	0.124
C22orf23	0.606	1.062	0.456	0.000	0.000
C22orf39	2.437	3.027	0.590	0.000	0.000
C22orf46	2.285	3.026	0.741	0.000	0.000
C2CD2	2.459	2.787	0.328	0.000	0.000
C2CD2L	2.257	2.456	0.199	0.000	0.000
C2CD3	1.407	1.970	0.563	0.000	0.000
C2CD4A	0.861	1.383	0.522	0.000	0.000
C2CD4B	1.854	1.108	-0.747	0.000	0.000
C2CD4C	0.535	0.472	-0.062	0.051	0.056
C2orf15	1.016	1.662	0.646	0.000	0.000
C2orf16	0.927	1.055	0.128	0.000	0.000
C2orf40	1.229	0.526	-0.703	0.000	0.000
C2orf42	3.025	3.175	0.150	0.000	0.000
C2orf49	2.224	2.827	0.603	0.000	0.000
C2orf66	0.130	0.215	0.085	0.000	0.000
C2orf68	3.590	4.477	0.887	0.000	0.000
C2orf69	2.801	3.396	0.595	0.000	0.000
C2orf72	6.418	6.646	0.228	0.000	0.000
C2orf74	3.010	3.269	0.259	0.000	0.000
C2orf76	2.513	3.314	0.801	0.000	0.000
C2orf81	0.688	0.865	0.177	0.000	0.000
C2orf88	1.678	1.320	-0.358	0.000	0.000
C3	12.351	11.570	-0.781	0.000	0.000
C3AR1	3.033	3.091	0.058	0.374	0.390
C3orf14	1.188	1.533	0.345	0.000	0.000
C3orf18	1.954	2.762	0.809	0.000	0.000
C3orf20	0.132	0.136	0.003	0.745	0.755
C3orf33	1.767	2.533	0.765	0.000	0.000
C3orf36	0.204	0.349	0.145	0.000	0.000
C3orf38	3.006	3.515	0.509	0.000	0.000
C3orf52	0.392	0.533	0.140	0.001	0.001
C3orf62	1.814	2.582	0.768	0.000	0.000
C3orf67	0.556	1.010	0.454	0.000	0.000
C3orf70	0.440	0.677	0.237	0.000	0.000

C4A	7.528	7.082	-0.446	0.000	0.000
C4B	7.673	7.197	-0.476	0.000	0.000
C4BPA	10.040	9.313	-0.728	0.000	0.000
C4BPB	7.858	7.626	-0.231	0.001	0.001
C4orf19	4.390	4.139	-0.251	0.000	0.000
C4orf3	6.274	6.137	-0.138	0.000	0.000
C4orf33	2.050	1.949	-0.101	0.007	0.008
C4orf36	0.787	0.808	0.020	0.312	0.328
C4orf46	1.172	2.034	0.862	0.000	0.000
C4orf47	0.507	0.715	0.208	0.000	0.000
C4orf48	2.139	3.049	0.910	0.000	0.000
C5	7.900	7.548	-0.352	0.000	0.000
C5AR1	3.260	3.001	-0.259	0.000	0.000
C5orf15	5.595	6.117	0.522	0.000	0.000
C5orf22	3.027	3.910	0.882	0.000	0.000
C5orf24	4.271	4.815	0.544	0.000	0.000
C5orf30	0.933	1.822	0.890	0.000	0.000
C5orf34	0.533	1.535	1.001	0.000	0.000
C5orf46	0.171	1.041	0.870	0.000	0.000
C5orf49	1.278	1.014	-0.264	0.000	0.000
C5orf51	2.937	3.835	0.898	0.000	0.000
C5orf56	1.999	2.126	0.127	0.001	0.001
C5orf58	0.102	0.816	0.714	0.000	0.000
C5orf63	0.980	1.319	0.339	0.000	0.000
C6	8.331	6.304	-2.027	0.000	0.000
C6orf106	5.788	6.716	0.927	0.000	0.000
C6orf120	4.608	4.957	0.349	0.000	0.000
C6orf132	0.589	0.869	0.279	0.000	0.000
C6orf136	3.968	4.819	0.851	0.000	0.000
C6orf141	1.406	1.023	-0.383	0.000	0.000
C6orf163	0.461	0.652	0.191	0.000	0.000
C6orf201	1.080	1.141	0.061	0.025	0.028
C6orf203	4.123	4.484	0.361	0.000	0.000
C6orf223	0.418	1.004	0.586	0.000	0.000
C6orf226	3.781	4.503	0.722	0.000	0.000
C6orf47	3.518	4.499	0.981	0.000	0.000
C6orf48	5.338	6.653	1.315	0.000	0.000
C6orf52	0.690	1.004	0.314	0.000	0.000
C6orf62	5.727	6.612	0.885	0.000	0.000
C7	6.734	3.846	-2.888	0.000	0.000
C7orf25	1.186	1.962	0.776	0.000	0.000
C7orf26	3.888	4.458	0.570	0.000	0.000
C7orf31	1.096	1.667	0.571	0.000	0.000
C7orf43	3.386	4.038	0.652	0.000	0.000
C7orf50	3.990	5.130	1.139	0.000	0.000
C7orf61	0.383	0.571	0.188	0.000	0.000
C8A	8.982	7.095	-1.886	0.000	0.000
C8B	8.940	7.526	-1.414	0.000	0.000
C8G	8.877	8.656	-0.221	0.001	0.001
C8orf33	3.768	5.334	1.565	0.000	0.000
C8orf37	1.326	1.857	0.531	0.000	0.000
C8orf44	1.386	2.195	0.810	0.000	0.000

C8orf44-SGK3	1.452	1.660	0.207	0.000	0.000
C8orf48	0.447	0.561	0.114	0.000	0.000
C8orf58	1.905	1.894	-0.011	0.772	0.781
C8orf59	4.643	5.805	1.162	0.000	0.000
C8orf76	2.942	4.192	1.250	0.000	0.000
C8orf82	5.155	5.540	0.385	0.000	0.000
C9	9.332	5.620	-3.712	0.000	0.000
C9orf116	1.167	1.573	0.406	0.000	0.000
C9orf152	0.610	1.314	0.704	0.000	0.000
C9orf153	0.112	0.136	0.024	0.007	0.008
C9orf16	4.734	5.770	1.037	0.000	0.000
C9orf24	0.468	0.755	0.287	0.000	0.000
C9orf3	3.870	4.064	0.194	0.000	0.000
C9orf40	1.885	2.864	0.979	0.000	0.000
C9orf43	1.179	1.190	0.011	0.701	0.712
C9orf47	0.285	0.288	0.003	0.867	0.872
C9orf50	0.113	0.168	0.055	0.000	0.000
C9orf64	3.922	4.245	0.324	0.000	0.000
C9orf66	0.628	0.430	-0.198	0.000	0.000
C9orf72	3.007	2.694	-0.312	0.000	0.000
C9orf78	5.029	5.144	0.115	0.000	0.000
C9orf85	1.987	2.402	0.416	0.000	0.000
CA11	1.916	2.141	0.226	0.000	0.000
CA12	0.582	1.887	1.305	0.000	0.000
CA13	1.739	1.635	-0.104	0.020	0.022
CA14	2.119	2.051	-0.069	0.360	0.376
CA2	6.895	5.708	-1.187	0.000	0.000
CA3	1.150	0.852	-0.298	0.000	0.000
CA4	0.334	0.729	0.395	0.000	0.000
CA5A	3.500	2.772	-0.728	0.000	0.000
CA5B	0.913	1.353	0.440	0.000	0.000
CA8	0.098	0.287	0.189	0.000	0.000
CA9	1.658	1.618	-0.040	0.731	0.742
CAB39	4.368	4.968	0.600	0.000	0.000
CAB39L	1.855	1.977	0.122	0.000	0.000
CABIN1	3.116	3.892	0.776	0.000	0.000
CABLES1	2.583	2.929	0.346	0.000	0.000
CABLES2	1.714	2.699	0.985	0.000	0.000
CABP1	0.122	0.212	0.091	0.000	0.000
CABP4	1.143	0.789	-0.353	0.000	0.000
CABP7	0.885	1.194	0.310	0.000	0.000
CABYR	0.562	1.761	1.199	0.000	0.000
CACHD1	1.333	1.715	0.382	0.000	0.000
CACNA1C	0.417	0.719	0.301	0.000	0.000
CACNA1D	0.972	1.392	0.420	0.000	0.000
CACNA1H	2.719	2.175	-0.544	0.000	0.000
CACNA2D1	0.580	0.506	-0.074	0.013	0.015
CACNA2D2	0.502	0.553	0.051	0.065	0.071
CACNA2D3	0.279	0.448	0.169	0.000	0.000
CACNA2D4	0.754	0.930	0.176	0.000	0.000
CACNB1	0.581	0.862	0.282	0.000	0.000
CACNB2	1.649	1.423	-0.226	0.000	0.000

CACNB3	0.822	1.337	0.515	0.000	0.000
CACNB4	0.116	0.361	0.244	0.000	0.000
CACNG4	0.212	0.998	0.786	0.000	0.000
CACYBP	4.130	5.468	1.338	0.000	0.000
CAD	2.867	3.933	1.066	0.000	0.000
CADM1	4.090	4.652	0.562	0.000	0.000
CADM2	0.235	0.261	0.026	0.334	0.350
CADM3	0.569	0.417	-0.152	0.000	0.000
CADM4	2.717	3.104	0.386	0.000	0.000
CADPS2	2.695	3.376	0.681	0.000	0.000
CALCA	0.638	0.820	0.182	0.007	0.008
CALCB	0.077	0.201	0.125	0.000	0.000
CALCOCO1	4.032	4.515	0.483	0.000	0.000
CALCOCO2	5.094	5.419	0.325	0.000	0.000
CALCRL	2.469	3.211	0.741	0.000	0.000
CALD1	6.360	6.521	0.161	0.000	0.000
CALHM2	2.143	2.444	0.300	0.000	0.000
CALM1	6.388	6.436	0.048	0.109	0.117
CALM2	6.730	7.449	0.719	0.000	0.000
CALM3	6.346	7.182	0.837	0.000	0.000
CALML3	0.477	0.295	-0.182	0.000	0.000
CALML4	2.774	3.216	0.442	0.000	0.000
CALML6	0.779	0.967	0.187	0.000	0.000
CALR	9.184	10.122	0.938	0.000	0.000
CALR3	0.132	0.231	0.099	0.000	0.000
CALU	4.862	5.994	1.131	0.000	0.000
CALY	0.167	0.345	0.178	0.000	0.000
CAMK1D	2.157	2.383	0.225	0.000	0.000
CAMK1G	0.344	0.414	0.070	0.030	0.033
CAMK2A	0.218	0.332	0.114	0.001	0.001
CAMK2B	2.325	0.806	-1.519	0.000	0.000
CAMK2D	3.669	3.803	0.134	0.000	0.000
CAMK2G	2.838	3.587	0.749	0.000	0.000
CAMK2N1	6.079	6.349	0.270	0.000	0.000
CAMK2N2	0.879	2.045	1.166	0.000	0.000
CAMK4	0.520	0.319	-0.200	0.000	0.000
CAMKK1	0.739	0.888	0.149	0.000	0.000
CAMKK2	4.046	4.436	0.389	0.000	0.000
CAMKMT	1.509	2.020	0.511	0.000	0.000
CAMLG	4.497	5.328	0.831	0.000	0.000
CAMSAP1	2.217	2.761	0.544	0.000	0.000
CAMTA1	3.551	3.759	0.208	0.000	0.000
CAMTA2	3.745	3.789	0.044	0.288	0.303
CAND1	3.557	4.140	0.583	0.000	0.000
CAND2	1.059	0.727	-0.332	0.000	0.000
CANT1	3.552	4.799	1.248	0.000	0.000
CANX	7.350	8.492	1.142	0.000	0.000
CAP1	6.041	6.767	0.726	0.000	0.000
CAP2	2.107	4.431	2.324	0.000	0.000
CAPG	2.960	4.737	1.777	0.000	0.000
CAPN1	4.733	5.558	0.824	0.000	0.000
CAPN10	2.160	2.864	0.704	0.000	0.000

CAPN11	0.218	0.647	0.429	0.000	0.000
CAPN12	2.955	3.866	0.911	0.000	0.000
CAPN13	0.249	0.449	0.200	0.000	0.000
CAPN2	4.104	5.136	1.032	0.000	0.000
CAPN3	2.593	2.272	-0.321	0.000	0.000
CAPN5	4.673	4.797	0.124	0.035	0.039
CAPN6	0.877	0.781	-0.096	0.225	0.238
CAPN7	3.580	4.038	0.459	0.000	0.000
CAPN8	0.337	0.813	0.476	0.000	0.000
CAPN9	0.184	0.379	0.195	0.000	0.000
CAPNS1	6.463	7.444	0.981	0.000	0.000
CAPRIN1	4.748	5.720	0.973	0.000	0.000
CAPRIN2	2.574	2.944	0.370	0.000	0.000
CAPS	2.904	3.451	0.547	0.000	0.000
CAPS2	0.797	0.763	-0.033	0.171	0.182
CAPZA1	5.218	5.948	0.730	0.000	0.000
CAPZA2	4.406	5.163	0.757	0.000	0.000
CAPZA3	0.653	0.507	-0.147	0.000	0.000
CARD10	3.700	4.266	0.566	0.000	0.000
CARD11	1.716	1.559	-0.157	0.007	0.008
CARD14	0.423	0.575	0.152	0.000	0.000
CARD16	3.415	3.464	0.049	0.373	0.389
CARD17	0.339	0.459	0.120	0.000	0.000
CARD6	2.215	2.327	0.112	0.031	0.035
CARD8	2.695	3.159	0.464	0.000	0.000
CARD9	1.005	1.046	0.041	0.224	0.237
CARHSP1	5.656	5.827	0.171	0.000	0.000
CARM1	3.707	4.525	0.817	0.000	0.000
CARNS1	0.626	0.815	0.189	0.000	0.000
CARS	3.533	3.853	0.319	0.000	0.000
CARS2	3.515	3.897	0.382	0.000	0.000
CASC1	0.238	0.226	-0.012	0.504	0.519
CASC3	3.958	4.952	0.993	0.000	0.000
CASC4	4.620	5.015	0.395	0.000	0.000
CASD1	2.829	3.439	0.610	0.000	0.000
CASK	2.501	3.596	1.095	0.000	0.000
CASKIN1	0.881	1.309	0.428	0.000	0.000
CASKIN2	3.635	3.819	0.184	0.000	0.000
CASP1	3.233	3.176	-0.057	0.335	0.351
CASP10	2.422	2.703	0.281	0.000	0.000
CASP2	2.091	3.020	0.928	0.000	0.000
CASP3	4.101	4.798	0.698	0.000	0.000
CASP4	4.149	4.284	0.135	0.007	0.008
CASP5	0.316	0.391	0.075	0.004	0.005
CASP6	4.119	4.371	0.251	0.000	0.000
CASP7	3.925	4.125	0.200	0.000	0.000
CASP8	2.835	3.523	0.688	0.000	0.000
CASP8AP2	1.942	2.495	0.553	0.000	0.000
CASP9	2.812	3.080	0.267	0.000	0.000
CASQ1	0.288	0.410	0.122	0.000	0.000
CASQ2	0.563	1.025	0.462	0.000	0.000
CASR	0.523	0.317	-0.205	0.000	0.000

CASS4	0.962	0.825	-0.137	0.000	0.000
CAST	4.713	5.099	0.386	0.000	0.000
CASZ1	1.116	1.278	0.163	0.000	0.000
CAT	8.969	7.940	-1.029	0.000	0.000
CATSPER1	0.133	0.314	0.180	0.000	0.000
CATSPER2	0.659	1.037	0.378	0.000	0.000
CATSPER3	1.072	1.286	0.215	0.000	0.000
CATSPERB	0.259	0.378	0.119	0.000	0.000
CATSPERG	0.629	0.632	0.003	0.899	0.903
CAV1	3.747	4.464	0.717	0.000	0.000
CAV2	4.081	4.330	0.249	0.001	0.001
CBFA2T2	2.147	3.135	0.988	0.000	0.000
CBFA2T3	2.061	1.112	-0.948	0.000	0.000
CBFB	3.666	4.090	0.423	0.000	0.000
CBL	1.715	2.074	0.359	0.000	0.000
CBLB	2.345	2.573	0.228	0.000	0.000
CBLC	4.114	4.235	0.121	0.074	0.080
CBLL1	2.734	3.586	0.852	0.000	0.000
CBLN1	0.690	1.397	0.707	0.000	0.000
CBLN3	2.079	1.764	-0.314	0.000	0.000
CBLN4	0.648	0.635	-0.013	0.817	0.825
CBR1	7.484	7.814	0.330	0.000	0.000
CBR3	1.127	2.060	0.933	0.000	0.000
CBR4	4.677	3.988	-0.689	0.000	0.000
CBS	5.958	5.012	-0.945	0.000	0.000
CBWD1	1.731	2.105	0.375	0.000	0.000
CBWD5	1.635	1.638	0.004	0.874	0.879
CBX1	3.664	4.959	1.294	0.000	0.000
CBX2	0.558	1.654	1.095	0.000	0.000
CBX3	5.335	6.296	0.961	0.000	0.000
CBX4	3.851	5.009	1.158	0.000	0.000
CBX5	2.656	3.361	0.705	0.000	0.000
CBX6	1.819	2.370	0.551	0.000	0.000
CBX7	3.213	3.565	0.352	0.000	0.000
CBX8	1.978	3.181	1.203	0.000	0.000
CBY1	3.813	4.444	0.631	0.000	0.000
CBY3	0.239	0.438	0.199	0.000	0.000
CC2D1A	4.030	4.586	0.556	0.000	0.000
CC2D1B	3.190	4.035	0.845	0.000	0.000
CC2D2A	1.221	1.503	0.283	0.000	0.000
CC2D2B	0.264	0.341	0.077	0.000	0.000
CCAR1	4.250	4.913	0.662	0.000	0.000
CCBE1	1.992	0.501	-1.490	0.000	0.000
CCDC102A	1.616	2.031	0.416	0.000	0.000
CCDC102B	0.929	1.549	0.620	0.000	0.000
CCDC103	0.181	0.323	0.142	0.000	0.000
CCDC106	3.983	3.906	-0.078	0.065	0.071
CCDC110	0.389	0.717	0.329	0.000	0.000
CCDC112	1.303	2.039	0.736	0.000	0.000
CCDC113	0.795	1.081	0.286	0.000	0.000
CCDC114	0.206	0.375	0.168	0.000	0.000
CCDC115	3.626	4.032	0.406	0.000	0.000

CCDC116	0.156	0.209	0.053	0.000	0.000
CCDC117	3.017	3.842	0.824	0.000	0.000
CCDC12	4.157	4.685	0.528	0.000	0.000
CCDC120	2.281	2.676	0.395	0.000	0.000
CCDC121	1.378	1.905	0.527	0.000	0.000
CCDC122	0.896	1.235	0.339	0.000	0.000
CCDC124	5.446	6.363	0.918	0.000	0.000
CCDC125	3.026	3.429	0.403	0.000	0.000
CCDC126	2.506	2.920	0.414	0.000	0.000
CCDC127	1.668	2.223	0.555	0.000	0.000
CCDC13	0.495	0.657	0.162	0.000	0.000
CCDC130	3.909	4.556	0.647	0.000	0.000
CCDC134	1.719	2.517	0.798	0.000	0.000
CCDC136	0.251	0.272	0.021	0.272	0.286
CCDC137	3.308	4.539	1.232	0.000	0.000
CCDC138	0.765	1.530	0.765	0.000	0.000
CCDC14	2.698	3.474	0.776	0.000	0.000
CCDC141	0.207	0.166	-0.041	0.009	0.010
CCDC142	1.834	2.743	0.909	0.000	0.000
CCDC146	2.467	2.373	-0.094	0.071	0.077
CCDC148	0.372	0.561	0.189	0.000	0.000
CCDC149	1.684	1.968	0.284	0.000	0.000
CCDC15	0.951	1.551	0.600	0.000	0.000
CCDC150	1.510	1.669	0.158	0.000	0.000
CCDC151	0.737	0.692	-0.045	0.096	0.104
CCDC152	6.665	5.730	-0.935	0.000	0.000
CCDC153	0.628	0.764	0.136	0.000	0.000
CCDC154	0.773	1.117	0.344	0.000	0.000
CCDC157	0.728	0.916	0.188	0.000	0.000
CCDC158	0.817	0.745	-0.073	0.013	0.014
CCDC159	3.564	4.198	0.634	0.000	0.000
CCDC167	5.379	6.483	1.103	0.000	0.000
CCDC17	1.367	1.445	0.078	0.057	0.062
CCDC18	1.691	1.950	0.259	0.000	0.000
CCDC22	3.641	4.351	0.710	0.000	0.000
CCDC24	1.992	2.695	0.703	0.000	0.000
CCDC25	4.962	4.582	-0.380	0.000	0.000
CCDC28A	5.286	5.269	-0.017	0.608	0.622
CCDC28B	1.282	2.494	1.212	0.000	0.000
CCDC3	4.084	2.993	-1.091	0.000	0.000
CCDC30	0.345	0.432	0.087	0.000	0.000
CCDC34	1.843	3.470	1.627	0.000	0.000
CCDC36	0.176	0.260	0.083	0.000	0.000
CCDC38	0.928	0.632	-0.296	0.000	0.000
CCDC40	0.808	1.246	0.439	0.000	0.000
CCDC42	0.199	0.124	-0.075	0.000	0.000
CCDC43	2.889	3.643	0.754	0.000	0.000
CCDC47	5.548	6.332	0.784	0.000	0.000
CCDC50	4.611	4.937	0.325	0.000	0.000
CCDC51	3.139	3.965	0.827	0.000	0.000
CCDC57	3.049	3.133	0.084	0.064	0.069
CCDC58	4.545	5.103	0.558	0.000	0.000

CCDC59	3.560	4.178	0.618	0.000	0.000
CCDC6	3.026	3.678	0.652	0.000	0.000
CCDC61	2.880	3.782	0.903	0.000	0.000
CCDC65	0.796	1.017	0.221	0.000	0.000
CCDC66	1.651	2.218	0.567	0.000	0.000
CCDC68	2.705	2.375	-0.331	0.000	0.000
CCDC69	3.974	3.887	-0.087	0.092	0.100
CCDC7	0.568	0.854	0.286	0.000	0.000
CCDC71	3.666	4.289	0.623	0.000	0.000
CCDC73	1.090	1.341	0.251	0.000	0.000
CCDC74A	0.763	0.932	0.169	0.000	0.000
CCDC74B	0.179	0.251	0.072	0.002	0.002
CCDC77	1.675	2.619	0.943	0.000	0.000
CCDC78	0.322	0.688	0.366	0.000	0.000
CCDC8	0.967	0.777	-0.190	0.000	0.000
CCDC80	1.564	2.230	0.666	0.000	0.000
CCDC81	0.226	0.405	0.179	0.000	0.000
CCDC82	3.030	3.378	0.347	0.000	0.000
CCDC84	2.896	3.320	0.424	0.000	0.000
CCDC85A	0.184	0.322	0.139	0.000	0.000
CCDC85B	4.332	4.733	0.402	0.000	0.000
CCDC85C	1.947	2.654	0.707	0.000	0.000
CCDC86	3.581	4.695	1.115	0.000	0.000
CCDC87	0.365	0.519	0.154	0.000	0.000
CCDC88A	2.354	3.069	0.715	0.000	0.000
CCDC88B	2.511	2.569	0.058	0.301	0.316
CCDC88C	1.976	1.856	-0.120	0.017	0.019
CCDC89	0.287	0.244	-0.043	0.009	0.010
CCDC9	3.698	4.198	0.500	0.000	0.000
CCDC90B	3.943	4.020	0.077	0.007	0.008
CCDC91	2.833	3.280	0.447	0.000	0.000
CCDC92	3.532	3.776	0.244	0.000	0.000
CCDC93	2.553	3.554	1.001	0.000	0.000
CCDC96	0.845	1.162	0.317	0.000	0.000
CCDC97	3.016	4.116	1.099	0.000	0.000
CCHCR1	2.694	4.074	1.380	0.000	0.000
CCL11	0.626	0.736	0.110	0.032	0.035
CCL13	0.526	1.105	0.579	0.000	0.000
CCL17	0.806	1.012	0.206	0.000	0.000
CCL19	5.344	3.203	-2.141	0.000	0.000
CCL2	5.202	4.080	-1.121	0.000	0.000
CCL20	2.959	5.160	2.201	0.000	0.000
CCL21	6.108	4.019	-2.089	0.000	0.000
CCL22	0.709	0.646	-0.063	0.109	0.117
CCL24	1.386	1.364	-0.023	0.719	0.730
CCL25	0.645	2.085	1.440	0.000	0.000
CCL26	0.332	0.833	0.501	0.000	0.000
CCL28	1.154	1.573	0.419	0.000	0.000
CCL8	1.481	1.774	0.293	0.000	0.000
CCM2	4.560	4.708	0.148	0.000	0.000
CCNA2	1.630	3.513	1.883	0.000	0.000
CCNB1	1.575	4.466	2.891	0.000	0.000

CCNB1IP1	4.821	4.759	-0.061	0.235	0.248
CCNB2	0.752	3.220	2.468	0.000	0.000
CCNB3	0.313	0.545	0.233	0.000	0.000
CCNC	4.511	4.991	0.480	0.000	0.000
CCND1	6.130	5.768	-0.362	0.000	0.000
CCND2	1.718	2.055	0.337	0.000	0.000
CCND3	3.479	3.823	0.343	0.000	0.000
CCNDBP1	3.440	4.016	0.576	0.000	0.000
CCNE1	0.757	2.283	1.526	0.000	0.000
CCNE2	0.671	2.020	1.349	0.000	0.000
CCNF	0.758	2.062	1.304	0.000	0.000
CCNG1	6.222	6.824	0.602	0.000	0.000
CCNG2	2.059	2.760	0.700	0.000	0.000
CCNH	3.883	3.984	0.102	0.000	0.000
CCNI	5.998	5.892	-0.106	0.023	0.025
CCNI2	0.440	0.699	0.260	0.000	0.000
CCNJ	1.537	1.772	0.235	0.000	0.000
CCNJL	0.416	0.683	0.267	0.000	0.000
CCNK	2.343	2.938	0.594	0.000	0.000
CCNL1	5.120	5.057	-0.063	0.175	0.186
CCNL2	5.410	5.895	0.485	0.000	0.000
CCNO	0.400	1.264	0.864	0.000	0.000
CCNT1	2.192	2.495	0.303	0.000	0.000
CCNT2	3.332	3.627	0.295	0.000	0.000
CCNY	4.605	5.247	0.642	0.000	0.000
CCNYL1	3.137	3.651	0.514	0.000	0.000
CCP110	1.593	1.907	0.314	0.000	0.000
CCPG1	3.981	4.033	0.052	0.150	0.160
CCR1	3.016	2.490	-0.526	0.000	0.000
CCR10	0.526	0.739	0.214	0.000	0.000
CCR2	1.392	1.232	-0.160	0.002	0.003
CCR4	0.688	0.578	-0.110	0.004	0.004
CCR5	1.823	1.760	-0.063	0.304	0.319
CCR6	0.428	0.706	0.278	0.000	0.000
CCR7	1.514	1.268	-0.245	0.000	0.000
CCR8	0.229	0.436	0.208	0.000	0.000
CCRL2	1.323	1.600	0.277	0.000	0.000
CCS	6.092	5.978	-0.114	0.001	0.001
CCT2	5.237	6.189	0.952	0.000	0.000
CCT3	6.199	7.863	1.664	0.000	0.000
CCT4	5.824	6.850	1.027	0.000	0.000
CCT5	5.034	6.256	1.222	0.000	0.000
CCT6A	5.503	6.871	1.368	0.000	0.000
CCT6B	1.604	1.226	-0.378	0.000	0.000
CCT7	6.104	6.978	0.874	0.000	0.000
CCT8	5.747	6.630	0.883	0.000	0.000
CD101	0.858	0.996	0.139	0.000	0.000
CD109	0.814	2.051	1.237	0.000	0.000
CD14	9.112	8.028	-1.083	0.000	0.000
CD151	6.324	7.179	0.855	0.000	0.000
CD160	1.228	0.712	-0.515	0.000	0.000
CD163	5.026	3.917	-1.109	0.000	0.000

CD163L1	1.626	2.234	0.609	0.000	0.000
CD164	6.412	6.612	0.200	0.000	0.000
CD177	0.207	0.433	0.226	0.000	0.000
CD180	1.307	1.117	-0.189	0.000	0.000
CD19	0.867	0.743	-0.124	0.015	0.017
CD1A	0.212	0.310	0.098	0.001	0.002
CD1B	0.315	0.508	0.193	0.000	0.000
CD1C	2.182	1.698	-0.484	0.000	0.000
CD1D	4.028	2.586	-1.442	0.000	0.000
CD1E	1.047	0.872	-0.175	0.000	0.000
CD2	3.251	3.172	-0.079	0.323	0.339
CD200	1.139	2.071	0.932	0.000	0.000
CD200R1	1.042	0.828	-0.214	0.000	0.000
CD207	0.891	0.584	-0.306	0.000	0.000
CD209	1.778	1.752	-0.026	0.629	0.642
CD22	1.075	0.946	-0.129	0.006	0.007
CD226	0.678	0.419	-0.260	0.000	0.000
CD244	1.837	1.047	-0.789	0.000	0.000
CD247	2.264	1.860	-0.404	0.000	0.000
CD248	2.258	3.095	0.837	0.000	0.000
CD27	2.391	2.348	-0.043	0.551	0.565
CD274	1.767	1.511	-0.256	0.000	0.000
CD276	4.519	4.845	0.326	0.000	0.000
CD28	1.075	1.308	0.233	0.000	0.000
CD2AP	3.621	4.445	0.824	0.000	0.000
CD2BP2	4.284	5.175	0.890	0.000	0.000
CD300A	3.383	2.688	-0.695	0.000	0.000
CD300C	1.327	1.170	-0.158	0.000	0.001
CD300E	1.264	0.920	-0.343	0.000	0.000
CD300LB	0.721	0.807	0.086	0.050	0.055
CD300LF	1.373	1.682	0.309	0.000	0.000
CD300LG	0.660	0.501	-0.159	0.000	0.000
CD320	3.886	4.790	0.905	0.000	0.000
CD33	1.517	1.350	-0.167	0.000	0.000
CD34	1.701	3.600	1.899	0.000	0.000
CD36	3.532	3.978	0.446	0.000	0.000
CD37	2.733	2.738	0.005	0.934	0.937
CD38	1.438	1.258	-0.180	0.002	0.002
CD3D	3.294	3.293	-0.001	0.990	0.990
CD3E	3.304	2.910	-0.394	0.000	0.000
CD3G	1.063	1.018	-0.046	0.338	0.353
CD4	5.763	4.439	-1.325	0.000	0.000
CD40	4.074	4.130	0.055	0.360	0.376
CD40LG	1.092	1.085	-0.006	0.892	0.896
CD44	3.365	3.557	0.192	0.022	0.024
CD46	5.603	6.532	0.929	0.000	0.000
CD47	4.165	4.643	0.478	0.000	0.000
CD48	3.265	2.854	-0.411	0.000	0.000
CD5	1.697	1.600	-0.097	0.103	0.111
CD52	4.746	4.718	-0.028	0.745	0.755
CD53	4.717	4.506	-0.211	0.004	0.005
CD55	3.671	3.901	0.230	0.001	0.001

CD58	2.960	3.861	0.901	0.000	0.000
CD5L	5.603	2.614	-2.990	0.000	0.000
CD6	1.770	1.591	-0.179	0.002	0.002
CD63	8.359	9.126	0.767	0.000	0.000
CD68	4.069	4.111	0.042	0.427	0.443
CD69	2.856	1.971	-0.885	0.000	0.000
CD7	2.465	2.573	0.109	0.143	0.153
CD70	0.408	0.582	0.174	0.000	0.000
CD72	2.117	2.103	-0.014	0.804	0.812
CD74	9.415	9.622	0.208	0.007	0.008
CD79A	2.439	1.706	-0.733	0.000	0.000
CD79B	2.273	2.152	-0.120	0.020	0.023
CD80	0.809	0.824	0.016	0.680	0.692
CD81	8.045	7.331	-0.715	0.000	0.000
CD82	4.622	4.520	-0.101	0.048	0.052
CD83	3.092	3.149	0.057	0.319	0.335
CD84	1.814	1.773	-0.041	0.462	0.478
CD86	2.524	2.411	-0.112	0.061	0.067
CD8A	2.810	2.207	-0.603	0.000	0.000
CD8B	1.777	1.488	-0.289	0.000	0.000
CD9	5.053	5.396	0.343	0.000	0.000
CD93	3.085	3.770	0.685	0.000	0.000
CD96	1.628	1.510	-0.118	0.031	0.034
CD99L2	4.325	5.100	0.775	0.000	0.000
CDA	5.984	4.518	-1.466	0.000	0.000
CDADC1	2.789	2.919	0.130	0.000	0.000
CDAN1	2.553	3.278	0.725	0.000	0.000
CDC123	4.788	5.791	1.003	0.000	0.000
CDC14A	1.158	1.597	0.439	0.000	0.000
CDC14B	3.630	3.353	-0.277	0.000	0.000
CDC16	4.847	5.340	0.493	0.000	0.000
CDC20	1.041	4.035	2.993	0.000	0.000
CDC23	3.292	4.372	1.080	0.000	0.000
CDC25A	0.624	1.774	1.150	0.000	0.000
CDC25B	3.829	4.960	1.131	0.000	0.000
CDC25C	0.356	2.221	1.865	0.000	0.000
CDC27	3.622	4.388	0.767	0.000	0.000
CDC34	6.295	6.557	0.263	0.000	0.000
CDC37	6.144	6.878	0.735	0.000	0.000
CDC37L1	4.988	4.176	-0.812	0.000	0.000
CDC40	3.067	3.285	0.218	0.000	0.000
CDC42	6.176	6.838	0.662	0.000	0.000
CDC42BPA	3.258	3.774	0.516	0.000	0.000
CDC42BPB	4.289	4.828	0.539	0.000	0.000
CDC42BPG	1.169	1.292	0.123	0.032	0.035
CDC42EP1	6.115	6.656	0.541	0.000	0.000
CDC42EP2	2.535	2.673	0.138	0.003	0.003
CDC42EP3	1.699	1.773	0.074	0.095	0.102
CDC42EP4	4.155	4.924	0.769	0.000	0.000
CDC42EP5	2.377	2.669	0.292	0.000	0.000
CDC42SE1	4.563	5.419	0.857	0.000	0.000
CDC42SE2	3.814	4.433	0.618	0.000	0.000

CDC45	0.790	2.638	1.848	0.000	0.000
CDC5L	3.714	4.450	0.737	0.000	0.000
CDC6	0.886	2.826	1.941	0.000	0.000
CDC7	1.301	2.460	1.159	0.000	0.000
CDC73	3.287	4.143	0.857	0.000	0.000
CDCA2	0.300	1.489	1.189	0.000	0.000
CDCA3	0.501	2.320	1.820	0.000	0.000
CDCA4	1.583	2.926	1.343	0.000	0.000
CDCA5	0.784	3.049	2.265	0.000	0.000
CDCA7	0.382	1.605	1.223	0.000	0.000
CDCA7L	2.017	2.843	0.825	0.000	0.000
CDCA8	0.942	3.104	2.163	0.000	0.000
CDCP1	0.865	0.989	0.124	0.041	0.045
CDH1	5.977	5.728	-0.249	0.000	0.000
CDH11	0.737	1.178	0.441	0.000	0.000
CDH13	0.611	1.796	1.184	0.000	0.000
CDH15	0.692	0.682	-0.009	0.873	0.878
CDH16	0.619	0.768	0.149	0.010	0.011
CDH19	1.329	0.542	-0.787	0.000	0.000
CDH2	4.998	5.400	0.402	0.000	0.000
CDH23	1.761	1.499	-0.262	0.000	0.000
CDH24	0.882	1.937	1.055	0.000	0.000
CDH26	0.235	0.241	0.006	0.768	0.778
CDH3	0.135	0.271	0.136	0.000	0.000
CDH4	0.174	0.181	0.007	0.706	0.717
CDH5	3.653	3.834	0.181	0.001	0.001
CDH6	1.051	1.284	0.232	0.000	0.000
CDHR1	0.382	0.228	-0.154	0.000	0.000
CDHR2	5.012	2.343	-2.670	0.000	0.000
CDHR3	1.227	1.852	0.625	0.000	0.000
CDHR5	6.851	6.513	-0.337	0.000	0.000
CDIPT	5.699	6.349	0.650	0.000	0.000
CDK1	0.984	3.583	2.599	0.000	0.000
CDK10	4.850	5.077	0.227	0.000	0.000
CDK11A	2.866	3.188	0.322	0.000	0.000
CDK11B	3.818	4.288	0.470	0.000	0.000
CDK12	2.886	3.487	0.601	0.000	0.000
CDK13	3.533	4.032	0.498	0.000	0.000
CDK14	2.838	3.455	0.617	0.000	0.000
CDK16	3.628	4.743	1.115	0.000	0.000
CDK17	3.065	3.358	0.293	0.000	0.000
CDK18	3.046	3.443	0.397	0.000	0.000
CDK19	2.232	2.828	0.596	0.000	0.000
CDK2	3.328	4.033	0.705	0.000	0.000
CDK20	1.487	1.721	0.234	0.000	0.000
CDK2AP1	4.188	4.925	0.737	0.000	0.000
CDK2AP2	5.870	6.549	0.679	0.000	0.000
CDK3	2.878	2.972	0.094	0.026	0.029
CDK5	2.766	4.031	1.266	0.000	0.000
CDK5R1	0.744	1.368	0.624	0.000	0.000
CDK5RAP1	3.455	4.303	0.848	0.000	0.000
CDK5RAP2	3.513	4.467	0.955	0.000	0.000

CDK5RAP3	5.484	6.361	0.877	0.000	0.000
CDK6	2.402	2.918	0.516	0.000	0.000
CDK7	3.365	4.353	0.988	0.000	0.000
CDK8	2.762	3.270	0.508	0.000	0.000
CDK9	5.588	5.564	-0.024	0.460	0.476
CDKAL1	2.128	3.018	0.890	0.000	0.000
CDKL1	0.519	0.773	0.254	0.000	0.000
CDKL3	0.670	1.026	0.356	0.000	0.000
CDKL5	0.495	0.619	0.124	0.000	0.000
CDKN1A	6.656	6.395	-0.262	0.000	0.000
CDKN1B	5.121	5.490	0.369	0.000	0.000
CDKN1C	2.654	2.229	-0.425	0.000	0.000
CDKN2A	1.213	3.375	2.163	0.000	0.000
CDKN2AIP	3.619	3.724	0.105	0.003	0.003
CDKN2AIPNL	3.604	4.892	1.288	0.000	0.000
CDKN2B	1.569	2.748	1.179	0.000	0.000
CDKN2C	1.859	3.721	1.862	0.000	0.000
CDKN2D	2.249	2.859	0.609	0.000	0.000
CDKN3	0.950	3.873	2.923	0.000	0.000
CDNF	2.801	2.243	-0.558	0.000	0.000
CDO1	7.966	7.097	-0.869	0.000	0.000
CDON	0.954	1.357	0.402	0.000	0.000
CDR2	3.775	4.229	0.454	0.000	0.000
CDR2L	1.658	1.945	0.287	0.000	0.000
CDRT1	0.087	0.508	0.421	0.000	0.000
CDRT4	1.425	1.426	0.000	0.996	0.996
CDS1	1.039	1.497	0.458	0.000	0.000
CDS2	3.354	4.029	0.675	0.000	0.000
CDT1	0.902	2.869	1.966	0.000	0.000
CDV3	5.794	6.400	0.606	0.000	0.000
CDX1	0.173	0.257	0.084	0.001	0.001
CDYL	2.307	3.101	0.794	0.000	0.000
CDYL2	1.049	1.112	0.063	0.075	0.082
CEACAM1	4.991	5.105	0.114	0.035	0.038
CEACAM16	0.167	0.218	0.051	0.002	0.003
CEACAM19	2.445	2.734	0.289	0.000	0.000
CEACAM21	1.193	1.062	-0.132	0.003	0.004
CEACAM3	0.367	0.187	-0.180	0.000	0.000
CEACAM4	0.805	0.562	-0.242	0.000	0.000
CEBPA	5.710	6.836	1.126	0.000	0.000
CEBPB	7.238	7.314	0.076	0.082	0.089
CEBPD	7.342	6.966	-0.376	0.000	0.000
CEBPG	4.888	5.839	0.950	0.000	0.000
CECR2	1.473	1.827	0.355	0.000	0.000
CEL	0.497	0.988	0.491	0.000	0.000
CELA2B	0.143	0.167	0.024	0.037	0.040
CELF1	4.517	4.939	0.422	0.000	0.000
CELF2	1.829	1.642	-0.187	0.000	0.000
CELF4	0.128	0.309	0.182	0.000	0.000
CELF5	0.270	0.621	0.351	0.000	0.000
CELF6	1.277	1.691	0.414	0.000	0.000
CELSR1	1.650	2.102	0.452	0.000	0.000

CELSR2	1.001	1.643	0.642	0.000	0.000
CELSR3	0.304	1.609	1.305	0.000	0.000
CEMP1	1.737	2.418	0.681	0.000	0.000
CEND1	0.203	0.426	0.224	0.000	0.000
CENPA	0.382	2.362	1.980	0.000	0.000
CENPB	5.074	5.985	0.911	0.000	0.000
CENPE	0.391	1.623	1.232	0.000	0.000
CENPF	0.496	2.951	2.455	0.000	0.000
CENPH	1.235	2.762	1.527	0.000	0.000
CENPI	0.313	1.243	0.930	0.000	0.000
CENPJ	1.292	2.243	0.952	0.000	0.000
CENPK	0.610	1.968	1.358	0.000	0.000
CENPL	0.655	2.003	1.348	0.000	0.000
CENPM	0.906	3.070	2.164	0.000	0.000
CENPN	2.159	3.139	0.980	0.000	0.000
CENPO	1.555	2.556	1.001	0.000	0.000
CENPP	0.880	1.311	0.431	0.000	0.000
CENPQ	1.971	3.251	1.280	0.000	0.000
CENPT	3.652	4.128	0.476	0.000	0.000
CENPV	4.713	5.174	0.461	0.000	0.000
CENPW	1.935	4.208	2.273	0.000	0.000
CEP104	2.906	3.301	0.394	0.000	0.000
CEP112	1.025	1.559	0.534	0.000	0.000
CEP120	2.751	2.991	0.240	0.000	0.000
CEP128	0.535	0.985	0.450	0.000	0.000
CEP135	0.996	1.327	0.331	0.000	0.000
CEP152	0.715	1.428	0.713	0.000	0.000
CEP164	2.487	2.875	0.388	0.000	0.000
CEP170	2.128	2.766	0.638	0.000	0.000
CEP19	0.892	1.387	0.495	0.000	0.000
CEP192	2.262	2.799	0.537	0.000	0.000
CEP250	1.592	2.646	1.054	0.000	0.000
CEP290	1.748	2.388	0.640	0.000	0.000
CEP350	2.502	3.386	0.884	0.000	0.000
CEP44	2.008	2.490	0.482	0.000	0.000
CEP55	0.541	2.052	1.511	0.000	0.000
CEP57	4.191	4.550	0.359	0.000	0.000
CEP57L1	1.768	2.132	0.364	0.000	0.000
CEP63	2.480	2.948	0.469	0.000	0.000
CEP68	1.822	2.611	0.789	0.000	0.000
CEP70	3.466	3.833	0.367	0.000	0.000
CEP72	1.387	2.450	1.063	0.000	0.000
CEP76	1.468	1.943	0.475	0.000	0.000
CEP78	1.393	1.916	0.523	0.000	0.000
CEP85	2.008	2.907	0.898	0.000	0.000
CEP89	1.265	2.023	0.759	0.000	0.000
CEP95	2.994	3.620	0.626	0.000	0.000
CEP97	0.856	1.316	0.460	0.000	0.000
CEPT1	3.490	3.553	0.064	0.070	0.076
CERCAM	1.527	1.912	0.384	0.000	0.000
CERK	4.219	4.912	0.693	0.000	0.000
CES1	10.856	10.255	-0.601	0.000	0.000

CES2	7.831	7.391	-0.440	0.000	0.000
CES3	4.437	3.869	-0.568	0.000	0.000
CES4A	3.239	2.234	-1.004	0.000	0.000
CES5A	1.715	1.084	-0.631	0.000	0.000
CETN2	4.362	5.475	1.113	0.000	0.000
CETN3	2.863	3.504	0.641	0.000	0.000
CETP	4.611	2.418	-2.193	0.000	0.000
CFD	4.040	3.258	-0.781	0.000	0.000
CFDP1	4.917	5.277	0.359	0.000	0.000
CFH	8.997	8.734	-0.262	0.000	0.000
CFHR1	9.327	8.828	-0.499	0.000	0.000
CFHR2	8.209	7.642	-0.568	0.000	0.000
CFHR3	7.344	5.138	-2.206	0.000	0.000
CFHR4	5.305	4.105	-1.200	0.000	0.000
CFHR5	5.973	5.912	-0.061	0.637	0.650
CFI	8.679	7.751	-0.929	0.000	0.000
CFL1	6.903	7.991	1.088	0.000	0.000
CFL2	5.370	5.044	-0.326	0.000	0.000
CFP	4.444	1.792	-2.652	0.000	0.000
CFTR	1.993	0.766	-1.226	0.000	0.000
CGGBP1	4.464	4.964	0.500	0.000	0.000
CGN	5.086	5.349	0.263	0.000	0.000
CGNL1	4.400	4.572	0.172	0.003	0.004
CGREF1	3.436	4.076	0.640	0.000	0.000
CGRRF1	2.262	2.808	0.546	0.000	0.000
CH25H	2.335	1.460	-0.875	0.000	0.000
CHAC1	1.939	2.376	0.437	0.000	0.000
CHAC2	2.317	2.705	0.388	0.000	0.000
CHAD	4.410	4.270	-0.141	0.065	0.071
CHADL	1.838	2.252	0.414	0.000	0.000
CHAF1A	1.968	3.460	1.492	0.000	0.000
CHAF1B	0.767	1.843	1.076	0.000	0.000
CHCHD1	5.205	5.855	0.650	0.000	0.000
CHCHD10	7.779	7.646	-0.133	0.007	0.008
CHCHD2	7.944	8.697	0.753	0.000	0.000
CHCHD3	4.100	5.036	0.936	0.000	0.000
CHCHD4	3.839	4.601	0.762	0.000	0.000
CHCHD5	3.760	4.261	0.501	0.000	0.000
CHCHD6	2.340	3.065	0.725	0.000	0.000
CHCHD7	3.300	4.070	0.769	0.000	0.000
CHD1	3.491	3.604	0.114	0.003	0.004
CHD1L	4.609	5.813	1.204	0.000	0.000
CHD2	3.745	3.902	0.157	0.000	0.000
CHD3	2.720	3.402	0.682	0.000	0.000
CHD4	5.156	5.924	0.768	0.000	0.000
CHD6	2.844	3.050	0.206	0.000	0.000
CHD7	1.956	2.630	0.674	0.000	0.000
CHD8	3.157	3.881	0.724	0.000	0.000
CHD9	2.854	2.741	-0.113	0.007	0.008
CHDH	4.688	4.787	0.099	0.038	0.042
CHEK1	0.945	2.165	1.219	0.000	0.000
CHEK2	2.040	3.249	1.209	0.000	0.000

CHERP	3.943	4.566	0.623	0.000	0.000
CHFR	1.863	2.374	0.512	0.000	0.000
CHI3L1	6.942	6.303	-0.639	0.000	0.000
CHI3L2	0.557	0.680	0.123	0.007	0.008
CHIC1	1.256	1.264	0.009	0.862	0.867
CHIC2	4.280	4.078	-0.202	0.000	0.000
CHID1	4.890	5.504	0.614	0.000	0.000
CHIT1	0.248	0.751	0.504	0.000	0.000
CHKA	4.587	5.357	0.770	0.000	0.000
CHKB	3.433	3.905	0.472	0.000	0.000
CHKB-CPT1B	2.135	2.424	0.289	0.000	0.000
CHL1	0.273	0.478	0.205	0.000	0.000
CHM	2.274	2.795	0.521	0.000	0.000
CHML	1.539	2.780	1.241	0.000	0.000
CHMP1A	4.907	5.506	0.599	0.000	0.000
CHMP1B	4.831	5.390	0.559	0.000	0.000
CHMP2A	6.657	7.408	0.751	0.000	0.000
CHMP2B	4.449	5.031	0.582	0.000	0.000
CHMP4A	3.459	3.718	0.259	0.000	0.000
CHMP4C	2.991	3.989	0.997	0.000	0.000
CHMP5	5.376	5.954	0.578	0.000	0.000
CHMP6	4.363	5.043	0.681	0.000	0.000
CHMP7	3.721	4.015	0.294	0.000	0.000
CHN1	0.948	1.618	0.669	0.000	0.000
CHN2	3.465	3.460	-0.004	0.924	0.928
CHODL	0.122	0.468	0.346	0.000	0.000
CHORDC1	2.508	3.150	0.642	0.000	0.000
CHPF	5.474	5.842	0.368	0.000	0.000
CHPF2	3.938	4.715	0.777	0.000	0.000
CHPT1	5.505	6.037	0.532	0.000	0.000
CHRAC1	3.516	4.480	0.964	0.000	0.000
CHRD	5.065	4.542	-0.524	0.000	0.000
CHRDL1	0.948	0.658	-0.290	0.000	0.000
CHRDL2	2.142	2.614	0.472	0.000	0.000
CHRM3	0.289	0.715	0.425	0.000	0.000
CHRNA10	0.738	0.777	0.039	0.146	0.156
CHRNA4	2.894	1.325	-1.568	0.000	0.000
CHRNA5	0.150	0.564	0.413	0.000	0.000
CHRNB1	2.821	2.976	0.155	0.000	0.000
CHRNE	2.181	2.003	-0.178	0.000	0.000
CHST1	1.099	1.706	0.607	0.000	0.000
CHST10	0.751	1.342	0.592	0.000	0.000
CHST11	1.770	2.305	0.534	0.000	0.000
CHST12	1.337	1.781	0.445	0.000	0.000
CHST13	4.969	5.680	0.711	0.000	0.000
CHST14	2.731	3.414	0.683	0.000	0.000
CHST15	3.286	3.670	0.384	0.000	0.000
CHST2	1.332	1.665	0.333	0.000	0.000
CHST3	1.277	1.464	0.187	0.001	0.001
CHST4	2.893	0.814	-2.079	0.000	0.000
CHST7	3.446	2.589	-0.857	0.000	0.000
CHST8	0.258	0.208	-0.050	0.138	0.148

CHST9	1.906	1.522	-0.384	0.000	0.000
CHSY1	2.909	3.048	0.139	0.009	0.010
CHSY3	1.074	1.055	-0.019	0.619	0.633
CHTF18	1.920	3.016	1.096	0.000	0.000
CHTOP	4.415	5.367	0.953	0.000	0.000
CHUK	3.000	3.512	0.512	0.000	0.000
CHURC1	4.219	4.435	0.216	0.000	0.000
CHURC1-FNT	1.678	1.829	0.151	0.000	0.000
CIAO1	5.258	5.794	0.537	0.000	0.000
CIAPIN1	3.895	4.618	0.724	0.000	0.000
CIB1	7.193	7.524	0.331	0.000	0.000
CIB2	0.920	1.835	0.915	0.000	0.000
CIC	3.606	4.266	0.660	0.000	0.000
CIDEC	1.249	1.421	0.172	0.026	0.029
CIITA	1.552	1.747	0.195	0.000	0.000
CILP	1.139	0.685	-0.454	0.000	0.000
CILP2	0.432	0.512	0.080	0.041	0.045
CINP	3.394	3.914	0.520	0.000	0.000
CIR1	4.408	4.912	0.504	0.000	0.000
CIRBP	5.897	6.224	0.327	0.000	0.000
CISD1	4.519	5.045	0.526	0.000	0.000
CISD2	5.291	5.692	0.401	0.000	0.000
CISH	4.056	3.825	-0.230	0.000	0.000
CIT	0.996	1.492	0.497	0.000	0.000
CITED1	0.167	0.347	0.181	0.000	0.000
CITED2	5.392	5.140	-0.253	0.000	0.000
CITED4	5.390	5.545	0.155	0.021	0.023
CIZ1	3.878	4.799	0.921	0.000	0.000
CKAP2	1.659	3.277	1.618	0.000	0.000
CKAP2L	0.409	1.812	1.402	0.000	0.000
CKAP4	4.628	6.088	1.460	0.000	0.000
CKAP5	3.931	4.628	0.697	0.000	0.000
CKB	3.867	4.534	0.668	0.000	0.000
CKLF	3.328	4.548	1.219	0.000	0.000
CKLF-CMTM1	1.042	1.564	0.522	0.000	0.000
CKM	0.131	0.218	0.088	0.000	0.000
CKMT2	0.978	1.019	0.041	0.489	0.504
CKS2	4.756	6.346	1.590	0.000	0.000
CLASP1	3.053	3.398	0.346	0.000	0.000
CLASP2	2.171	2.719	0.549	0.000	0.000
CLASRP	4.301	4.808	0.507	0.000	0.000
CLCF1	2.223	2.569	0.346	0.000	0.000
CLCN1	0.097	0.389	0.292	0.000	0.000
CLCN2	1.723	2.516	0.793	0.000	0.000
CLCN3	3.249	3.586	0.337	0.000	0.000
CLCN4	1.816	2.327	0.511	0.000	0.000
CLCN5	3.253	3.329	0.076	0.075	0.082
CLCN6	2.638	2.971	0.333	0.000	0.000
CLCN7	3.967	4.997	1.031	0.000	0.000
CLCNKA	0.221	0.788	0.567	0.000	0.000
CLDN1	7.597	7.605	0.008	0.880	0.884
CLDN10	2.550	0.810	-1.741	0.000	0.000

CLDN11	1.392	1.254	-0.138	0.019	0.021
CLDN12	4.480	4.905	0.425	0.000	0.000
CLDN14	4.251	3.933	-0.318	0.000	0.000
CLDN15	3.321	4.976	1.655	0.000	0.000
CLDN16	0.986	1.285	0.300	0.000	0.000
CLDN19	0.159	0.548	0.389	0.000	0.000
CLDN2	5.046	4.152	-0.893	0.000	0.000
CLDN23	2.927	3.286	0.359	0.000	0.000
CLDN3	5.320	5.460	0.141	0.080	0.087
CLDN4	2.407	2.927	0.519	0.000	0.000
CLDN5	3.394	3.465	0.071	0.240	0.253
CLDN6	0.150	0.261	0.111	0.003	0.003
CLDN7	4.422	5.112	0.690	0.000	0.000
CLDN9	1.733	1.977	0.245	0.000	0.000
CLDND1	4.224	4.569	0.346	0.000	0.000
CLEC10A	2.989	2.583	-0.406	0.000	0.000
CLEC11A	3.548	3.011	-0.537	0.000	0.000
CLEC12A	1.745	1.049	-0.696	0.000	0.000
CLEC12B	0.218	0.147	-0.071	0.000	0.000
CLEC14A	3.314	4.016	0.701	0.000	0.000
CLEC16A	2.215	2.851	0.636	0.000	0.000
CLEC17A	0.342	0.225	-0.117	0.000	0.000
CLEC18B	0.203	0.309	0.105	0.000	0.000
CLEC1A	1.236	1.258	0.022	0.536	0.550
CLEC1B	3.692	0.454	-3.237	0.000	0.000
CLEC2B	3.259	2.920	-0.339	0.000	0.000
CLEC2D	1.878	2.152	0.274	0.000	0.000
CLEC3B	5.043	4.069	-0.974	0.000	0.000
CLEC4A	1.977	2.099	0.121	0.007	0.008
CLEC4D	0.224	0.177	-0.047	0.012	0.014
CLEC4E	1.251	1.077	-0.175	0.001	0.002
CLEC4F	0.916	0.652	-0.264	0.000	0.000
CLEC4G	5.856	1.051	-4.805	0.000	0.000
CLEC4M	4.492	0.502	-3.989	0.000	0.000
CLEC5A	0.484	0.675	0.192	0.000	0.000
CLEC7A	2.131	1.851	-0.281	0.000	0.000
CLEC9A	0.677	0.400	-0.276	0.000	0.000
CLECL1	0.898	0.788	-0.110	0.004	0.004
CLGN	1.269	2.960	1.691	0.000	0.000
CLIC1	6.474	7.758	1.283	0.000	0.000
CLIC2	2.946	2.866	-0.080	0.154	0.164
CLIC3	1.108	1.300	0.191	0.001	0.001
CLIC4	5.004	5.329	0.326	0.000	0.000
CLIC5	0.482	0.795	0.313	0.000	0.000
CLIC6	1.522	0.708	-0.814	0.000	0.000
CLINT1	5.358	5.517	0.159	0.000	0.000
CLIP1	3.448	3.931	0.483	0.000	0.000
CLIP2	1.804	2.470	0.666	0.000	0.000
CLIP3	1.675	2.004	0.329	0.000	0.000
CLIP4	2.052	2.426	0.373	0.000	0.000
CLK1	5.795	5.810	0.015	0.745	0.755
CLK2	4.054	5.170	1.115	0.000	0.000

CLK3	2.741	3.181	0.439	0.000	0.000
CLK4	3.085	3.141	0.056	0.116	0.124
CLMN	3.488	3.662	0.174	0.000	0.000
CLMP	0.533	0.443	-0.090	0.013	0.014
CLN3	3.197	4.616	1.418	0.000	0.000
CLN5	3.720	3.823	0.103	0.001	0.002
CLN6	3.594	4.794	1.200	0.000	0.000
CLN8	3.046	2.690	-0.356	0.000	0.000
CLNS1A	4.865	5.659	0.794	0.000	0.000
CLOCK	1.988	2.332	0.344	0.000	0.000
CLP1	2.854	3.509	0.656	0.000	0.000
CLPB	3.487	3.754	0.267	0.000	0.000
CLPP	5.446	5.661	0.215	0.000	0.000
CLPTM1	5.776	6.579	0.802	0.000	0.000
CLPTM1L	4.945	5.859	0.914	0.000	0.000
CLPX	4.934	4.907	-0.027	0.406	0.422
CLRN3	5.459	2.964	-2.495	0.000	0.000
CLSPN	0.254	1.481	1.227	0.000	0.000
CLSTN1	3.678	4.550	0.872	0.000	0.000
CLSTN2	0.510	0.365	-0.145	0.000	0.000
CLSTN3	4.540	4.973	0.433	0.000	0.000
CLTA	6.089	7.130	1.041	0.000	0.000
CLTB	5.579	6.194	0.615	0.000	0.000
CLTC	5.582	6.513	0.930	0.000	0.000
CLTCL1	1.602	2.477	0.875	0.000	0.000
CLU	11.030	10.580	-0.450	0.000	0.000
CLUAP1	1.829	2.342	0.513	0.000	0.000
CLUL1	0.084	0.404	0.321	0.000	0.000
CLVS1	0.888	1.479	0.592	0.000	0.000
CLYBL	4.445	3.847	-0.598	0.000	0.000
CMA1	0.633	0.460	-0.173	0.000	0.000
CMAS	5.440	5.877	0.437	0.000	0.000
CMBL	7.335	6.633	-0.701	0.000	0.000
CMC1	2.945	3.455	0.510	0.000	0.000
CMIP	2.643	3.112	0.469	0.000	0.000
CMKLR1	2.159	2.076	-0.083	0.123	0.132
CMPK1	6.844	6.987	0.144	0.000	0.000
CMPK2	2.286	2.443	0.157	0.011	0.013
CMTM1	0.464	0.777	0.313	0.000	0.000
CMTM2	0.580	0.477	-0.102	0.001	0.002
CMTM3	3.106	3.698	0.592	0.000	0.000
CMTM4	1.524	2.387	0.864	0.000	0.000
CMTM6	5.889	5.744	-0.144	0.000	0.001
CMTM7	2.541	2.997	0.456	0.000	0.000
CMTM8	5.186	5.650	0.464	0.000	0.000
CMYA5	1.523	1.137	-0.386	0.000	0.000
CNBP	8.490	8.475	-0.015	0.545	0.559
CNDP1	3.826	1.097	-2.729	0.000	0.000
CNDP2	5.842	5.908	0.066	0.047	0.051
CNFN	0.696	1.488	0.792	0.000	0.000
CNGA1	3.981	2.890	-1.091	0.000	0.000
CNGA4	0.160	0.215	0.055	0.000	0.000

CNIH2	0.470	0.798	0.328	0.000	0.000
CNIH3	0.643	0.991	0.348	0.000	0.000
CNIH4	3.256	4.682	1.426	0.000	0.000
CNKSR1	1.705	0.991	-0.714	0.000	0.000
CNKSR2	0.702	1.274	0.572	0.000	0.000
CNKSR3	2.939	2.791	-0.148	0.000	0.000
CNN1	1.665	1.978	0.314	0.000	0.000
CNN2	5.124	5.290	0.165	0.002	0.002
CNN3	6.318	7.023	0.705	0.000	0.000
CNNM1	0.565	1.219	0.653	0.000	0.000
CNNM2	1.347	1.815	0.468	0.000	0.000
CNNM3	4.319	4.828	0.509	0.000	0.000
CNNM4	1.992	2.675	0.683	0.000	0.000
CNOT1	4.274	4.877	0.603	0.000	0.000
CNOT10	3.248	4.051	0.803	0.000	0.000
CNOT2	3.261	3.749	0.488	0.000	0.000
CNOT3	3.191	4.041	0.850	0.000	0.000
CNOT4	2.493	3.100	0.606	0.000	0.000
CNOT6	2.353	3.205	0.851	0.000	0.000
CNOT6L	3.123	3.192	0.069	0.063	0.069
CNOT7	3.552	3.841	0.289	0.000	0.000
CNOT8	4.086	4.690	0.604	0.000	0.000
CNP	4.361	5.228	0.867	0.000	0.000
CNPPD1	5.021	5.760	0.739	0.000	0.000
CNPY2	4.887	5.830	0.942	0.000	0.000
CNPY3	6.058	6.454	0.396	0.000	0.000
CNPY4	2.376	3.181	0.806	0.000	0.000
CNR1	0.206	0.578	0.372	0.000	0.000
CNRIP1	1.980	1.863	-0.117	0.005	0.006
CNST	3.774	4.163	0.388	0.000	0.000
CNTD1	1.567	2.146	0.579	0.000	0.000
CNTD2	1.165	2.013	0.848	0.000	0.000
CNTF	0.619	0.779	0.160	0.000	0.000
CNTFR	3.050	1.780	-1.270	0.000	0.000
CNTLN	2.304	2.400	0.096	0.011	0.012
CNTN1	0.437	0.333	-0.104	0.035	0.039
CNTN3	1.847	0.834	-1.012	0.000	0.000
CNTN4	1.047	0.618	-0.429	0.000	0.000
CNTNAP1	1.007	1.594	0.587	0.000	0.000
CNTNAP2	1.462	1.712	0.250	0.000	0.000
CNTNAP3	0.320	0.480	0.160	0.000	0.000
CNTNAP3B	0.467	0.654	0.186	0.000	0.000
CNTRL	2.013	2.387	0.374	0.000	0.000
CNTROB	2.880	3.457	0.577	0.000	0.000
COASY	5.216	5.884	0.668	0.000	0.000
COBL	2.994	3.182	0.188	0.000	0.000
COBLL1	4.391	3.944	-0.448	0.000	0.000
COCH	0.450	2.187	1.737	0.000	0.000
COG1	3.548	4.166	0.618	0.000	0.000
COG2	2.154	3.308	1.154	0.000	0.000
COG3	4.282	4.583	0.301	0.000	0.000
COG4	4.260	4.930	0.669	0.000	0.000

COG5	2.557	3.365	0.808	0.000	0.000
COG6	2.329	2.636	0.307	0.000	0.000
COG7	3.169	3.747	0.578	0.000	0.000
COG8	2.789	3.114	0.324	0.000	0.000
COIL	3.396	4.222	0.826	0.000	0.000
COL10A1	0.523	0.788	0.266	0.000	0.000
COL11A2	0.769	0.768	-0.001	0.989	0.990
COL12A1	1.615	1.942	0.327	0.000	0.000
COL13A1	0.434	0.575	0.141	0.000	0.000
COL14A1	2.872	2.382	-0.490	0.000	0.000
COL15A1	1.062	2.957	1.894	0.000	0.000
COL16A1	1.787	1.925	0.138	0.073	0.079
COL18A1	8.302	8.045	-0.257	0.000	0.000
COL1A1	4.315	5.496	1.181	0.000	0.000
COL1A2	4.107	5.052	0.944	0.000	0.000
COL21A1	0.714	1.379	0.665	0.000	0.000
COL22A1	0.174	0.821	0.646	0.000	0.000
COL23A1	0.837	0.763	-0.075	0.049	0.054
COL24A1	0.107	0.562	0.454	0.000	0.000
COL25A1	0.368	0.120	-0.249	0.000	0.000
COL27A1	3.587	3.749	0.162	0.014	0.016
COL28A1	0.420	0.398	-0.022	0.574	0.589
COL3A1	5.666	5.851	0.184	0.080	0.086
COL4A1	3.841	5.774	1.933	0.000	0.000
COL4A2	4.061	5.765	1.704	0.000	0.000
COL4A3	0.768	0.670	-0.098	0.027	0.030
COL4A3BP	3.407	3.600	0.193	0.000	0.000
COL4A4	1.224	1.101	-0.122	0.013	0.015
COL4A5	1.058	1.564	0.506	0.000	0.000
COL5A1	2.577	3.035	0.459	0.000	0.000
COL5A2	2.879	4.095	1.216	0.000	0.000
COL5A3	2.690	3.877	1.187	0.000	0.000
COL6A1	5.950	5.701	-0.249	0.000	0.001
COL6A2	5.685	5.502	-0.183	0.018	0.020
COL6A3	2.717	3.161	0.444	0.000	0.000
COL6A6	0.527	0.116	-0.411	0.000	0.000
COL7A1	1.144	2.265	1.121	0.000	0.000
COL8A1	0.600	1.202	0.602	0.000	0.000
COL8A2	0.995	1.017	0.022	0.700	0.712
COL9A1	0.069	0.369	0.300	0.000	0.000
COL9A2	1.162	1.573	0.411	0.000	0.000
COL9A3	1.938	1.840	-0.099	0.140	0.150
COLEC10	5.006	1.639	-3.367	0.000	0.000
COLEC11	6.469	4.781	-1.688	0.000	0.000
COLEC12	0.348	0.981	0.632	0.000	0.000
COLQ	1.473	1.807	0.334	0.000	0.000
COMMD1	4.335	4.673	0.337	0.000	0.000
COMMD10	3.594	4.371	0.777	0.000	0.000
COMMD2	2.926	3.973	1.046	0.000	0.000
COMMD3	4.156	4.978	0.823	0.000	0.000
COMMD3-BM	2.526	3.154	0.628	0.000	0.000
COMMD4	4.222	5.369	1.147	0.000	0.000

COMMD5	3.557	4.762	1.205	0.000	0.000
COMMD6	5.512	5.815	0.302	0.000	0.000
COMMD7	4.666	5.474	0.808	0.000	0.000
COMMD8	3.308	4.087	0.779	0.000	0.000
COMMD9	3.492	4.222	0.730	0.000	0.000
COMP	0.340	1.042	0.702	0.000	0.000
COMT	6.447	5.978	-0.469	0.000	0.000
COMTD1	4.192	4.535	0.343	0.000	0.000
COPA	5.485	6.662	1.177	0.000	0.000
COPB1	5.333	6.063	0.730	0.000	0.000
COPB2	4.880	5.603	0.723	0.000	0.000
COPE	5.856	6.852	0.996	0.000	0.000
COPG2	2.547	3.642	1.095	0.000	0.000
COPS2	4.004	4.565	0.561	0.000	0.000
COPS3	4.541	5.084	0.543	0.000	0.000
COPS4	3.784	4.164	0.380	0.000	0.000
COPS5	4.033	5.039	1.006	0.000	0.000
COPS6	5.542	6.538	0.996	0.000	0.000
COPS7A	5.019	5.704	0.685	0.000	0.000
COPS7B	3.159	3.929	0.770	0.000	0.000
COPS8	3.741	4.488	0.746	0.000	0.000
COPZ1	5.787	6.712	0.925	0.000	0.000
COPZ2	4.789	4.613	-0.176	0.001	0.001
COQ10A	3.889	4.412	0.523	0.000	0.000
COQ10B	4.483	4.839	0.356	0.000	0.000
COQ2	3.226	3.365	0.139	0.000	0.000
COQ3	3.708	4.078	0.370	0.000	0.000
COQ4	4.686	4.776	0.090	0.001	0.001
COQ5	5.140	5.482	0.342	0.000	0.000
COQ7	3.077	3.535	0.459	0.000	0.000
CORIN	0.073	0.400	0.327	0.000	0.000
CORO1A	3.891	3.729	-0.162	0.023	0.026
CORO1B	4.511	5.555	1.044	0.000	0.000
CORO1C	4.581	5.000	0.419	0.000	0.000
CORO2A	1.679	2.357	0.677	0.000	0.000
CORO2B	0.922	0.841	-0.081	0.052	0.057
CORO6	0.600	0.778	0.178	0.000	0.000
CORO7	2.226	2.635	0.409	0.000	0.000
CORT	0.631	0.715	0.085	0.001	0.001
COTL1	4.269	4.048	-0.220	0.001	0.001
COX10	3.003	3.314	0.311	0.000	0.000
COX11	4.214	4.799	0.585	0.000	0.000
COX15	4.273	4.750	0.476	0.000	0.000
COX16	4.835	5.189	0.354	0.000	0.000
COX17	5.509	5.864	0.355	0.000	0.000
COX18	3.084	3.028	-0.056	0.051	0.056
COX19	2.184	2.932	0.748	0.000	0.000
COX4I1	7.615	7.830	0.214	0.000	0.000
COX4I2	1.006	2.541	1.535	0.000	0.000
COX5A	7.215	7.855	0.641	0.000	0.000
COX5B	7.919	8.295	0.376	0.000	0.000
COX6A1	7.999	8.564	0.565	0.000	0.000

COX6A2	3.304	2.056	-1.248	0.000	0.000
COX6B1	8.328	9.180	0.852	0.000	0.000
COX6C	7.227	8.153	0.926	0.000	0.000
COX7A1	3.305	2.895	-0.409	0.000	0.000
COX7A2	6.940	7.554	0.614	0.000	0.000
COX7A2L	5.075	5.736	0.661	0.000	0.000
COX7B	6.267	6.698	0.431	0.000	0.000
COX7C	8.172	8.645	0.473	0.000	0.000
COX8A	8.396	9.087	0.691	0.000	0.000
CP	9.537	8.308	-1.229	0.000	0.000
CPA3	1.355	1.273	-0.082	0.250	0.264
CPA4	0.098	0.276	0.178	0.000	0.000
CPAMD8	0.816	0.515	-0.301	0.000	0.000
CPB1	0.189	0.187	-0.001	0.950	0.952
CPB2	9.699	8.995	-0.704	0.000	0.000
CPD	3.912	5.244	1.332	0.000	0.000
CPE	4.188	5.170	0.981	0.000	0.000
CPEB2	3.292	3.504	0.212	0.000	0.000
CPEB3	3.249	2.169	-1.080	0.000	0.000
CPEB4	4.209	4.151	-0.058	0.241	0.255
CPLX1	1.983	3.087	1.104	0.000	0.000
CPLX2	0.253	1.823	1.570	0.000	0.000
CPM	3.939	4.165	0.226	0.000	0.000
CPN1	6.056	5.422	-0.634	0.000	0.000
CPN2	7.562	6.794	-0.769	0.000	0.000
CPNE1	5.157	6.112	0.955	0.000	0.000
CPNE2	2.518	2.995	0.478	0.000	0.000
CPNE3	4.374	5.211	0.836	0.000	0.000
CPNE5	0.955	1.291	0.336	0.000	0.000
CPNE6	0.234	0.152	-0.083	0.000	0.001
CPNE7	0.833	1.433	0.600	0.000	0.000
CPNE8	3.281	3.150	-0.131	0.004	0.004
CPNE9	0.102	0.205	0.102	0.000	0.000
CPO	0.593	0.469	-0.124	0.000	0.000
CPOX	4.172	4.237	0.064	0.072	0.079
CPPED1	3.840	4.774	0.934	0.000	0.000
CPS1	9.155	7.450	-1.706	0.000	0.000
CPSF1	4.821	5.726	0.905	0.000	0.000
CPSF2	2.563	3.268	0.705	0.000	0.000
CPSF3	3.615	4.647	1.032	0.000	0.000
CPSF4	3.041	4.238	1.197	0.000	0.000
CPSF4L	0.488	0.761	0.273	0.000	0.000
CPSF6	3.513	4.505	0.993	0.000	0.000
CPSF7	4.781	5.209	0.428	0.000	0.000
CPT1A	5.985	5.909	-0.076	0.151	0.161
CPT1B	2.417	2.702	0.285	0.000	0.000
CPT1C	0.722	1.080	0.358	0.000	0.000
CPT2	5.948	5.342	-0.606	0.000	0.000
CPVL	4.100	4.374	0.273	0.001	0.001
CPXM1	0.681	1.645	0.963	0.000	0.000
CPXM2	1.789	1.516	-0.273	0.000	0.000
CPZ	1.004	0.703	-0.300	0.000	0.000

CR1	0.904	0.515	-0.389	0.000	0.000
CR1L	0.339	0.228	-0.111	0.000	0.000
CR2	0.398	0.329	-0.070	0.111	0.120
CRABP2	1.127	1.542	0.415	0.000	0.000
CRADD	3.751	3.775	0.023	0.491	0.506
CRAT	6.401	6.316	-0.086	0.018	0.020
CRB3	3.836	4.339	0.502	0.000	0.000
CRBN	3.827	4.022	0.195	0.000	0.000
CRCP	4.419	4.902	0.482	0.000	0.000
CREB1	2.494	3.052	0.558	0.000	0.000
CREB3	4.718	5.420	0.702	0.000	0.000
CREB3L1	0.844	1.470	0.627	0.000	0.000
CREB3L2	3.119	4.120	1.000	0.000	0.000
CREB3L3	7.461	7.074	-0.387	0.000	0.000
CREB3L4	3.071	4.119	1.048	0.000	0.000
CREB5	0.928	1.082	0.154	0.000	0.000
CREBBP	3.282	3.593	0.311	0.000	0.000
CREBL2	5.111	5.104	-0.006	0.865	0.870
CREBZF	4.226	4.718	0.491	0.000	0.000
CREG1	7.517	7.897	0.380	0.000	0.000
CRELD1	4.402	5.038	0.636	0.000	0.000
CRELD2	4.000	5.049	1.049	0.000	0.000
CREM	4.035	4.089	0.053	0.161	0.172
CRHBP	5.377	1.624	-3.753	0.000	0.000
CRIM1	3.576	3.728	0.152	0.029	0.032
CRIP1	2.580	2.847	0.267	0.000	0.000
CRIP2	4.713	4.902	0.189	0.001	0.001
CRIP3	1.963	3.285	1.322	0.000	0.000
CRIPAK	2.434	3.003	0.570	0.000	0.000
CRIPT	3.379	4.042	0.662	0.000	0.000
CRISPLD1	0.712	0.787	0.074	0.058	0.063
CRISPLD2	3.138	2.293	-0.845	0.000	0.000
CRK	4.856	4.916	0.061	0.082	0.089
CRKL	4.312	5.099	0.788	0.000	0.000
CRLF1	0.559	1.095	0.536	0.000	0.000
CRLF3	2.201	2.865	0.664	0.000	0.000
CRLS1	5.819	6.223	0.404	0.000	0.000
CRMP1	1.144	1.419	0.274	0.000	0.000
CRNKL1	3.595	4.422	0.827	0.000	0.000
CROCC	1.966	2.575	0.609	0.000	0.000
CROT	4.023	4.430	0.408	0.000	0.000
CRP	9.089	8.158	-0.931	0.000	0.000
CRTAC1	0.570	0.386	-0.185	0.000	0.000
CRTAM	0.990	0.764	-0.227	0.000	0.000
CRTAP	4.714	5.713	0.999	0.000	0.000
CRTC1	2.213	2.732	0.519	0.000	0.000
CRTC2	4.472	5.409	0.937	0.000	0.000
CRTC3	2.526	2.939	0.413	0.000	0.000
CRY1	3.297	3.529	0.231	0.000	0.000
CRY2	4.806	5.064	0.258	0.000	0.000
CRYAA	3.352	2.641	-0.711	0.000	0.000
CRYAB	2.907	2.905	-0.002	0.985	0.986

CRYBA1	0.134	0.170	0.036	0.003	0.003
CRYBB1	0.746	0.806	0.060	0.094	0.101
CRYBB2	0.324	0.500	0.176	0.000	0.000
CRYGS	1.816	2.285	0.469	0.000	0.000
CRYL1	7.482	7.028	-0.455	0.000	0.000
CRYM	3.116	3.079	-0.036	0.562	0.577
CRYZ	6.754	6.778	0.024	0.624	0.638
CRYZL1	2.654	3.052	0.398	0.000	0.000
CS	4.492	5.482	0.990	0.000	0.000
CSAD	5.410	4.785	-0.625	0.000	0.000
CSAG1	0.253	1.435	1.182	0.000	0.000
CSDC2	0.397	0.322	-0.076	0.019	0.021
CSDE1	6.736	7.274	0.538	0.000	0.000
CSE1L	4.434	5.631	1.197	0.000	0.000
CSF1	3.297	3.493	0.197	0.001	0.001
CSF1R	4.106	3.684	-0.423	0.000	0.000
CSF2RA	0.657	0.636	-0.021	0.400	0.417
CSF2RB	1.810	1.772	-0.038	0.497	0.512
CSF3R	2.467	1.907	-0.560	0.000	0.000
CSGALNACT1	1.740	1.982	0.241	0.000	0.000
CSGALNACT2	3.362	3.854	0.493	0.000	0.000
CSK	4.722	5.092	0.369	0.000	0.000
CSNK1A1	4.015	4.499	0.484	0.000	0.000
CSNK1D	4.366	5.260	0.894	0.000	0.000
CSNK1E	3.746	4.767	1.020	0.000	0.000
CSNK1G1	1.552	2.242	0.690	0.000	0.000
CSNK1G2	4.415	5.044	0.630	0.000	0.000
CSNK1G3	3.419	3.784	0.365	0.000	0.000
CSNK2A1	3.620	4.503	0.883	0.000	0.000
CSNK2A2	3.386	3.849	0.464	0.000	0.000
CSNK2B	5.364	6.312	0.948	0.000	0.000
CSPG4	0.931	1.949	1.018	0.000	0.000
CSPG5	0.191	0.876	0.685	0.000	0.000
CSPP1	2.459	3.264	0.805	0.000	0.000
CSRNP1	5.674	4.338	-1.336	0.000	0.000
CSRNP2	2.739	3.295	0.557	0.000	0.000
CSRNP3	0.341	0.360	0.019	0.413	0.429
CSRP1	5.046	5.011	-0.035	0.516	0.530
CSRP2	3.239	4.012	0.774	0.000	0.000
CST1	0.176	1.484	1.308	0.000	0.000
CST2	0.315	0.828	0.514	0.000	0.000
CST3	8.032	8.142	0.110	0.008	0.009
CST7	3.894	3.129	-0.764	0.000	0.000
CSTA	3.612	4.520	0.909	0.000	0.000
CSTB	5.496	6.886	1.390	0.000	0.000
CSTF1	3.486	4.064	0.579	0.000	0.000
CSTF2	2.419	3.626	1.207	0.000	0.000
CSTF2T	3.528	3.988	0.459	0.000	0.000
CSTF3	3.209	3.962	0.753	0.000	0.000
CTAGE4	0.197	0.320	0.123	0.000	0.000
CTBP1	4.958	5.623	0.665	0.000	0.000
CTBP2	1.781	1.798	0.017	0.761	0.771

CTBS	3.942	3.661	-0.281	0.000	0.000
CTC1	2.609	2.882	0.273	0.000	0.000
CTCF	4.001	4.520	0.519	0.000	0.000
CTDNEP1	5.012	5.458	0.446	0.000	0.000
CTDP1	2.612	3.232	0.620	0.000	0.000
CTDSP1	5.912	6.295	0.383	0.000	0.000
CTDSP2	5.145	5.825	0.680	0.000	0.000
CTDSPL	3.798	4.259	0.461	0.000	0.000
CTDSPL2	2.179	2.803	0.624	0.000	0.000
CTF1	1.422	1.000	-0.422	0.000	0.000
CTGF	5.717	5.724	0.006	0.941	0.944
CTH	6.662	5.295	-1.367	0.000	0.000
CTHRC1	0.915	2.895	1.979	0.000	0.000
CTIF	3.969	4.098	0.129	0.002	0.002
CTLA4	1.092	1.457	0.364	0.000	0.000
CTNNA1	5.394	6.430	1.036	0.000	0.000
CTNNA3	0.657	0.329	-0.329	0.000	0.000
CTNNAL1	4.194	4.608	0.415	0.000	0.000
CTNNB1	5.729	6.591	0.861	0.000	0.000
CTNNBIP1	3.497	4.188	0.690	0.000	0.000
CTNNBL1	4.811	5.320	0.510	0.000	0.000
CTNND1	5.552	5.871	0.320	0.000	0.000
CTNND2	0.652	1.258	0.606	0.000	0.000
CTNS	3.441	3.954	0.513	0.000	0.000
CTPS2	2.749	3.470	0.721	0.000	0.000
CTR9	4.345	5.024	0.680	0.000	0.000
CTRL	0.579	0.647	0.068	0.001	0.002
CTSA	5.832	7.267	1.435	0.000	0.000
CTSB	8.711	8.617	-0.094	0.046	0.051
CTSC	3.782	4.609	0.827	0.000	0.000
CTSD	9.245	9.996	0.752	0.000	0.000
CTSE	0.517	0.747	0.230	0.003	0.004
CTSF	6.298	6.470	0.173	0.001	0.001
CTSG	1.172	0.798	-0.373	0.000	0.000
CTSH	5.816	6.455	0.639	0.000	0.000
CTSK	3.317	3.916	0.599	0.000	0.000
CTSO	5.907	5.475	-0.432	0.000	0.000
CTSS	5.843	6.038	0.196	0.006	0.007
CTSW	2.693	2.205	-0.488	0.000	0.000
CTSZ	8.365	8.560	0.195	0.000	0.000
CTTN	4.846	5.837	0.991	0.000	0.000
CTTNBP2	1.090	0.786	-0.304	0.000	0.000
CTTNBP2NL	1.811	2.202	0.391	0.000	0.000
CTU1	2.028	2.845	0.817	0.000	0.000
CTU2	2.886	3.381	0.495	0.000	0.000
CTXN1	0.361	0.926	0.565	0.000	0.000
CUBN	0.244	0.280	0.036	0.055	0.060
CUEDC1	2.184	3.154	0.970	0.000	0.000
CUEDC2	5.367	5.983	0.616	0.000	0.000
CUL1	5.203	5.769	0.566	0.000	0.000
CUL2	3.458	4.230	0.772	0.000	0.000
CUL3	3.914	4.281	0.368	0.000	0.000

CUL4A	4.406	4.930	0.523	0.000	0.000
CUL4B	3.781	4.644	0.863	0.000	0.000
CUL5	3.318	3.725	0.407	0.000	0.000
CUL7	3.137	4.297	1.160	0.000	0.000
CUL9	2.564	3.481	0.917	0.000	0.000
CUTA	6.934	7.962	1.028	0.000	0.000
CUTC	4.471	4.683	0.213	0.000	0.000
CUX1	3.111	3.624	0.512	0.000	0.000
CUX2	3.910	3.217	-0.692	0.000	0.000
CUZD1	0.252	0.509	0.258	0.000	0.000
CWC15	5.049	5.529	0.480	0.000	0.000
CWC22	3.712	4.090	0.378	0.000	0.000
CWC27	2.824	3.720	0.895	0.000	0.000
CWF19L1	2.964	3.762	0.798	0.000	0.000
CWF19L2	3.179	3.425	0.246	0.000	0.000
CX3CL1	3.766	3.651	-0.115	0.037	0.041
CX3CR1	1.161	1.035	-0.126	0.002	0.002
CXADR	4.100	4.409	0.309	0.000	0.000
CXCL1	3.078	2.621	-0.457	0.000	0.000
CXCL10	4.781	5.422	0.641	0.000	0.000
CXCL11	1.595	2.241	0.646	0.000	0.000
CXCL12	6.125	3.609	-2.516	0.000	0.000
CXCL13	1.821	1.983	0.162	0.145	0.155
CXCL14	5.310	1.392	-3.918	0.000	0.000
CXCL16	5.199	5.526	0.327	0.000	0.000
CXCL17	0.185	1.014	0.830	0.000	0.000
CXCL2	7.736	5.574	-2.162	0.000	0.000
CXCL3	1.211	1.077	-0.134	0.026	0.029
CXCL5	0.787	1.221	0.434	0.000	0.000
CXCL6	3.080	1.887	-1.193	0.000	0.000
CXCL9	4.239	4.224	-0.015	0.892	0.896
CXCR1	0.806	0.442	-0.364	0.000	0.000
CXCR2	0.789	0.442	-0.347	0.000	0.000
CXCR3	1.304	1.488	0.184	0.002	0.002
CXCR4	5.002	4.969	-0.033	0.683	0.695
CXCR5	0.481	0.426	-0.055	0.050	0.055
CXCR6	1.843	1.522	-0.320	0.000	0.000
CXorf21	1.428	1.301	-0.127	0.006	0.007
CXorf38	2.562	2.984	0.423	0.000	0.000
CXorf40A	2.409	2.976	0.567	0.000	0.000
CXorf40B	3.563	4.316	0.752	0.000	0.000
CXorf56	3.221	3.990	0.768	0.000	0.000
CXorf58	0.126	0.162	0.036	0.000	0.000
CXorf65	0.702	0.717	0.015	0.704	0.716
CXorf66	0.362	0.233	-0.129	0.000	0.000
CXXC1	4.320	4.926	0.606	0.000	0.000
CXXC4	0.570	0.630	0.060	0.055	0.060
CXXC5	5.308	5.688	0.380	0.000	0.000
CYB561	3.187	3.834	0.647	0.000	0.000
CYB561D1	1.323	2.006	0.682	0.000	0.000
CYB561D2	3.981	4.816	0.835	0.000	0.000
CYB5A	8.042	7.548	-0.493	0.000	0.000

CYB5B	4.128	4.671	0.543	0.000	0.000
CYB5D1	2.406	2.342	-0.063	0.041	0.045
CYB5D2	4.935	4.436	-0.499	0.000	0.000
CYB5R1	4.425	5.565	1.140	0.000	0.000
CYB5R2	1.362	1.238	-0.124	0.001	0.001
CYB5R3	6.141	6.700	0.559	0.000	0.000
CYB5R4	1.734	2.277	0.543	0.000	0.000
CYB5RL	0.897	1.535	0.638	0.000	0.000
CYBA	5.315	5.330	0.015	0.857	0.863
CYBB	3.638	3.382	-0.256	0.001	0.001
CYBRD1	3.893	3.425	-0.468	0.000	0.000
CYC1	6.906	7.924	1.018	0.000	0.000
CYCS	5.623	6.425	0.802	0.000	0.000
CYFIP2	4.283	3.501	-0.783	0.000	0.000
CYGB	3.931	3.383	-0.548	0.000	0.000
CYHR1	3.304	4.386	1.082	0.000	0.000
CYLD	3.100	3.214	0.114	0.001	0.001
CYP11A1	2.164	2.162	-0.002	0.979	0.980
CYP17A1	1.777	3.114	1.337	0.000	0.000
CYP1A1	3.988	2.538	-1.450	0.000	0.000
CYP1A2	7.480	2.660	-4.820	0.000	0.000
CYP1B1	2.553	2.872	0.319	0.000	0.000
CYP20A1	2.520	2.882	0.362	0.000	0.000
CYP21A2	2.990	3.172	0.182	0.015	0.017
CYP26A1	3.289	0.983	-2.306	0.000	0.000
CYP26B1	0.621	1.003	0.382	0.000	0.000
CYP27A1	8.727	8.378	-0.349	0.000	0.000
CYP27B1	0.568	1.139	0.571	0.000	0.000
CYP27C1	0.210	0.543	0.333	0.000	0.000
CYP2A13	0.760	0.970	0.209	0.005	0.006
CYP2A6	9.729	6.364	-3.365	0.000	0.000
CYP2A7	6.459	3.072	-3.386	0.000	0.000
CYP2B6	8.455	5.062	-3.393	0.000	0.000
CYP2C18	6.303	5.291	-1.012	0.000	0.000
CYP2C19	3.222	1.870	-1.352	0.000	0.000
CYP2C8	10.351	7.015	-3.336	0.000	0.000
CYP2C9	9.977	7.502	-2.475	0.000	0.000
CYP2D6	8.213	7.428	-0.784	0.000	0.000
CYP2E1	10.641	7.469	-3.172	0.000	0.000
CYP2J2	6.722	5.692	-1.030	0.000	0.000
CYP2R1	2.473	3.442	0.968	0.000	0.000
CYP2S1	1.336	1.504	0.168	0.009	0.011
CYP2U1	2.180	2.108	-0.072	0.068	0.074
CYP2W1	0.176	0.478	0.303	0.000	0.000
CYP39A1	5.431	2.924	-2.507	0.000	0.000
CYP3A4	10.608	6.447	-4.161	0.000	0.000
CYP3A43	4.081	2.384	-1.697	0.000	0.000
CYP3A5	7.388	6.978	-0.410	0.000	0.000
CYP3A7	6.122	5.322	-0.799	0.000	0.000
CYP46A1	0.472	0.416	-0.056	0.003	0.003
CYP4A11	9.124	6.701	-2.423	0.000	0.000
CYP4A22	6.841	4.501	-2.340	0.000	0.000

CYP4F11	5.545	5.432	-0.113	0.131	0.141
CYP4F12	5.062	4.281	-0.781	0.000	0.000
CYP4F2	7.054	5.345	-1.709	0.000	0.000
CYP4F22	2.626	2.988	0.361	0.000	0.000
CYP4F3	6.522	5.979	-0.543	0.000	0.000
CYP4V2	5.716	4.779	-0.937	0.000	0.000
CYP4X1	2.640	2.213	-0.427	0.000	0.000
CYP4Z1	0.837	0.514	-0.323	0.000	0.000
CYP51A1	4.129	4.813	0.683	0.000	0.000
CYP7A1	3.752	4.755	1.003	0.000	0.000
CYP7B1	3.366	3.176	-0.190	0.003	0.003
CYP8B1	7.696	5.371	-2.324	0.000	0.000
CYR61	6.687	5.189	-1.498	0.000	0.000
CYS1	1.730	0.791	-0.939	0.000	0.000
CYSLTR1	0.664	0.718	0.054	0.046	0.050
CYSLTR2	0.408	0.506	0.098	0.001	0.001
CYTH1	4.160	4.774	0.615	0.000	0.000
CYTH2	3.213	4.151	0.938	0.000	0.000
CYTH3	1.946	2.675	0.729	0.000	0.000
CYTH4	2.393	2.497	0.104	0.054	0.059
CYTIP	2.798	2.228	-0.569	0.000	0.000
CYTL1	0.411	0.746	0.334	0.000	0.000
CYYR1	2.382	2.921	0.539	0.000	0.000
D2HGDH	4.314	4.371	0.057	0.239	0.252
DAAM1	2.739	2.843	0.104	0.030	0.033
DAAM2	2.157	2.146	-0.012	0.826	0.834
DAB1	1.203	1.043	-0.160	0.001	0.001
DAB2	3.662	4.129	0.467	0.000	0.000
DAB2IP	2.882	3.609	0.726	0.000	0.000
DACT1	1.752	1.420	-0.332	0.000	0.000
DACT2	1.759	1.730	-0.030	0.719	0.730
DACT3	1.234	0.956	-0.278	0.000	0.000
DAD1	7.602	8.519	0.917	0.000	0.000
DAG1	4.769	5.542	0.773	0.000	0.000
DAGLA	0.919	1.641	0.722	0.000	0.000
DAGLB	2.894	3.439	0.546	0.000	0.000
DALRD3	3.733	4.341	0.608	0.000	0.000
DAND5	0.082	0.279	0.196	0.000	0.000
DAO	5.542	4.599	-0.944	0.000	0.000
DAP	6.341	7.037	0.696	0.000	0.000
DAP3	5.170	6.458	1.288	0.000	0.000
DAPK1	4.318	4.096	-0.222	0.000	0.000
DAPK2	0.972	2.022	1.050	0.000	0.000
DAPK3	4.089	4.859	0.770	0.000	0.000
DAPP1	1.256	1.086	-0.170	0.000	0.000
DARS	4.853	5.451	0.598	0.000	0.000
DARS2	3.144	4.485	1.341	0.000	0.000
DAXX	4.148	5.256	1.108	0.000	0.000
DAZAP1	4.251	5.004	0.753	0.000	0.000
DAZAP2	6.243	6.634	0.390	0.000	0.000
DBF4	1.602	2.670	1.068	0.000	0.000
DBF4B	0.859	1.813	0.954	0.000	0.000

DBH	4.166	2.208	-1.958	0.000	0.000
DBI	7.776	8.070	0.294	0.000	0.000
DBN1	2.128	3.345	1.218	0.000	0.000
DBNDD1	2.010	3.670	1.660	0.000	0.000
DBNDD2	1.665	2.498	0.833	0.000	0.000
DBNL	3.776	4.527	0.751	0.000	0.000
DBP	2.581	3.431	0.850	0.000	0.000
DBR1	2.538	3.341	0.803	0.000	0.000
DBT	3.488	3.182	-0.307	0.000	0.000
DCAF10	3.258	3.748	0.491	0.000	0.000
DCAF11	6.157	5.905	-0.252	0.000	0.000
DCAF12	3.890	4.611	0.721	0.000	0.000
DCAF13	2.632	3.953	1.320	0.000	0.000
DCAF15	3.531	4.223	0.692	0.000	0.000
DCAF16	2.471	3.338	0.867	0.000	0.000
DCAF17	1.932	2.494	0.562	0.000	0.000
DCAF4	1.924	2.743	0.820	0.000	0.000
DCAF4L1	0.326	0.613	0.287	0.000	0.000
DCAF5	3.988	4.246	0.258	0.000	0.000
DCAF6	5.337	5.792	0.455	0.000	0.000
DCAF7	3.796	4.745	0.949	0.000	0.000
DCAKD	3.959	4.580	0.620	0.000	0.000
DCBLD1	2.302	2.475	0.173	0.000	0.000
DCBLD2	2.649	2.897	0.249	0.000	0.000
DCDC1	0.338	0.348	0.010	0.667	0.680
DCDC2	2.444	3.186	0.742	0.000	0.000
DCDC2B	0.572	0.634	0.062	0.001	0.001
DCHS1	1.591	1.611	0.020	0.624	0.638
DCK	2.614	3.616	1.001	0.000	0.000
DCLK1	0.166	0.329	0.164	0.000	0.000
DCLK2	1.018	1.126	0.107	0.006	0.006
DCLRE1A	2.533	3.071	0.538	0.000	0.000
DCLRE1B	1.375	2.240	0.865	0.000	0.000
DCLRE1C	1.305	1.985	0.680	0.000	0.000
DCN	6.239	3.419	-2.820	0.000	0.000
DCP1B	2.429	2.856	0.427	0.000	0.000
DCP2	2.468	3.145	0.678	0.000	0.000
DCPS	4.779	5.047	0.268	0.000	0.000
DCST2	1.189	1.662	0.472	0.000	0.000
DCT	0.197	0.160	-0.037	0.010	0.011
DCTD	5.254	5.309	0.055	0.111	0.119
DCTN1	3.973	4.770	0.797	0.000	0.000
DCTN2	4.632	5.799	1.167	0.000	0.000
DCTN3	4.401	5.045	0.644	0.000	0.000
DCTN4	3.925	4.754	0.829	0.000	0.000
DCTN5	2.500	3.104	0.604	0.000	0.000
DCTN6	4.382	4.534	0.152	0.000	0.000
DCTPP1	4.217	5.226	1.008	0.000	0.000
DCUN1D1	3.228	3.565	0.338	0.000	0.000
DCUN1D2	2.536	3.015	0.479	0.000	0.000
DCUN1D3	2.852	2.409	-0.443	0.000	0.000
DCUN1D4	4.019	4.365	0.346	0.000	0.000

DCUN1D5	3.754	4.560	0.806	0.000	0.000
DCXR	10.308	9.072	-1.236	0.000	0.000
DDA1	3.693	4.537	0.844	0.000	0.000
DDAH1	5.606	5.540	-0.067	0.180	0.192
DDAH2	5.036	5.905	0.868	0.000	0.000
DDB1	4.641	5.539	0.898	0.000	0.000
DDB2	4.065	3.914	-0.151	0.001	0.001
DDC	4.827	4.852	0.025	0.757	0.767
DDHD1	1.138	1.646	0.508	0.000	0.000
DDHD2	2.261	2.739	0.478	0.000	0.000
DDI2	3.056	3.180	0.124	0.003	0.003
DDIT3	5.356	6.192	0.836	0.000	0.000
DDIT4	5.469	6.123	0.653	0.000	0.000
DDIT4L	0.326	0.559	0.233	0.000	0.000
DDO	3.598	3.630	0.032	0.485	0.501
DDOST	6.320	7.451	1.130	0.000	0.000
DDR1	2.345	2.976	0.631	0.000	0.000
DDRGK1	6.264	6.848	0.584	0.000	0.000
DDTL	4.698	4.586	-0.112	0.067	0.073
DDX1	5.215	5.990	0.775	0.000	0.000
DDX10	2.447	3.244	0.798	0.000	0.000
DDX11	1.476	2.606	1.130	0.000	0.000
DDX17	6.192	6.733	0.542	0.000	0.000
DDX18	4.216	4.665	0.449	0.000	0.000
DDX19A	3.292	3.610	0.318	0.000	0.000
DDX19B	3.639	3.360	-0.279	0.000	0.000
DDX20	2.068	2.776	0.708	0.000	0.000
DDX21	4.537	4.967	0.429	0.000	0.000
DDX23	4.637	5.360	0.723	0.000	0.000
DDX24	4.995	5.214	0.219	0.000	0.000
DDX27	3.804	4.677	0.873	0.000	0.000
DDX28	3.389	3.729	0.339	0.000	0.000
DDX31	2.258	2.846	0.588	0.000	0.000
DDX39A	4.133	5.500	1.367	0.000	0.000
DDX39B	5.168	5.764	0.596	0.000	0.000
DDX3X	5.694	5.851	0.158	0.000	0.000
DDX3Y	3.177	3.170	-0.007	0.955	0.957
DDX41	4.583	5.593	1.009	0.000	0.000
DDX42	4.479	5.193	0.714	0.000	0.000
DDX43	0.578	0.631	0.052	0.224	0.237
DDX46	3.733	4.310	0.577	0.000	0.000
DDX47	2.560	2.856	0.297	0.000	0.000
DDX49	4.465	5.603	1.138	0.000	0.000
DDX5	7.130	7.513	0.383	0.000	0.000
DDX50	3.937	4.550	0.613	0.000	0.000
DDX51	2.826	3.491	0.665	0.000	0.000
DDX54	4.631	5.361	0.730	0.000	0.000
DDX55	3.019	3.716	0.698	0.000	0.000
DDX56	5.056	5.753	0.697	0.000	0.000
DDX58	3.296	3.576	0.280	0.000	0.000
DDX59	3.105	4.021	0.916	0.000	0.000
DDX6	3.508	3.921	0.413	0.000	0.000

DDX60	3.034	3.194	0.159	0.009	0.010
DDX60L	2.531	2.391	-0.140	0.001	0.001
DEAF1	2.883	3.806	0.923	0.000	0.000
DECR1	7.748	7.488	-0.260	0.000	0.000
DECR2	5.824	6.032	0.208	0.000	0.000
DEDD	4.308	5.291	0.983	0.000	0.000
DEDD2	4.131	4.758	0.628	0.000	0.000
DEF6	2.962	2.986	0.025	0.695	0.707
DEF8	3.095	3.785	0.690	0.000	0.000
DEFB1	8.097	7.069	-1.028	0.000	0.000
DEFB132	0.442	0.995	0.554	0.000	0.000
DEGS1	4.546	5.545	0.999	0.000	0.000
DEGS2	0.512	0.722	0.210	0.000	0.000
DEK	5.056	5.984	0.928	0.000	0.000
DENND1A	2.696	3.231	0.535	0.000	0.000
DENND1B	1.921	2.464	0.544	0.000	0.000
DENND1C	3.052	3.236	0.184	0.000	0.000
DENND2A	1.778	1.781	0.003	0.952	0.954
DENND2C	1.588	1.975	0.387	0.000	0.000
DENND2D	3.164	3.050	-0.114	0.044	0.049
DENND3	1.690	1.767	0.076	0.089	0.096
DENND4A	3.263	3.306	0.043	0.278	0.293
DENND4B	3.434	4.406	0.972	0.000	0.000
DENND4C	3.048	3.344	0.296	0.000	0.000
DENND5A	3.174	4.067	0.893	0.000	0.000
DENND5B	2.780	3.034	0.254	0.000	0.000
DENR	4.341	5.216	0.875	0.000	0.000
DEPDC1	0.294	1.846	1.552	0.000	0.000
DEPDC1B	0.619	2.262	1.642	0.000	0.000
DEPDC4	0.531	0.816	0.285	0.000	0.000
DEPDC5	2.740	3.068	0.327	0.000	0.000
DEPDC7	5.315	4.291	-1.024	0.000	0.000
DEPTOR	3.886	4.697	0.811	0.000	0.000
DERA	5.461	5.452	-0.010	0.780	0.789
DERL1	4.922	5.666	0.744	0.000	0.000
DERL2	4.188	4.527	0.340	0.000	0.000
DERL3	2.015	2.453	0.438	0.000	0.000
DES	1.491	0.719	-0.772	0.000	0.000
DET1	1.843	2.456	0.613	0.000	0.000
DFFA	2.876	3.493	0.617	0.000	0.000
DFFB	1.668	1.938	0.270	0.000	0.000
DGAT1	5.180	5.791	0.611	0.000	0.000
DGAT2	5.852	5.455	-0.397	0.000	0.000
DGCR2	4.248	5.281	1.033	0.000	0.000
DGCR6	3.049	2.882	-0.167	0.002	0.002
DGCR8	3.173	3.617	0.443	0.000	0.000
DGKA	1.905	1.945	0.041	0.463	0.478
DGKD	2.496	3.029	0.534	0.000	0.000
DGKE	1.451	1.402	-0.049	0.219	0.232
DGKG	0.557	0.701	0.144	0.000	0.000
DGKQ	2.607	3.423	0.816	0.000	0.000
DGKZ	2.799	3.509	0.710	0.000	0.000

DGUOK	5.065	5.680	0.615	0.000	0.000
DHCR24	8.710	9.288	0.579	0.000	0.000
DHCR7	5.877	6.534	0.657	0.000	0.000
DHDDS	3.338	4.011	0.672	0.000	0.000
DHDH	0.470	0.974	0.504	0.000	0.000
DHFR	4.053	4.452	0.399	0.000	0.000
DHH	0.224	0.361	0.137	0.000	0.000
DHODH	4.985	3.912	-1.073	0.000	0.000
DHPS	4.990	5.563	0.573	0.000	0.000
DHRS1	6.050	5.236	-0.814	0.000	0.000
DHRS12	4.034	3.662	-0.372	0.000	0.000
DHRS13	1.952	3.001	1.049	0.000	0.000
DHRS2	2.558	3.280	0.722	0.000	0.000
DHRS3	7.475	7.555	0.080	0.018	0.020
DHRS4	5.426	5.197	-0.228	0.000	0.000
DHRS4L2	5.142	4.891	-0.251	0.000	0.000
DHRS7	5.796	6.546	0.750	0.000	0.000
DHRS7B	3.359	3.680	0.321	0.000	0.000
DHRS9	1.043	1.244	0.201	0.000	0.000
DHTKD1	6.388	6.016	-0.372	0.000	0.000
DHX15	4.239	4.842	0.603	0.000	0.000
DHX16	4.372	5.175	0.803	0.000	0.000
DHX29	3.713	4.428	0.715	0.000	0.000
DHX30	4.021	4.726	0.705	0.000	0.000
DHX32	3.589	3.700	0.111	0.028	0.031
DHX33	2.676	3.089	0.414	0.000	0.000
DHX34	2.807	3.903	1.095	0.000	0.000
DHX35	2.288	3.004	0.715	0.000	0.000
DHX36	3.051	3.480	0.429	0.000	0.000
DHX37	2.285	3.182	0.897	0.000	0.000
DHX38	4.049	4.501	0.452	0.000	0.000
DHX40	2.887	3.525	0.637	0.000	0.000
DHX57	1.876	2.958	1.082	0.000	0.000
DHX58	3.911	3.836	-0.075	0.116	0.124
DHX8	2.931	3.690	0.759	0.000	0.000
DHX9	4.998	5.868	0.870	0.000	0.000
DIABLO	2.879	3.490	0.611	0.000	0.000
DIAPH1	5.894	6.302	0.408	0.000	0.000
DIAPH2	2.366	2.665	0.299	0.000	0.000
DIAPH3	0.255	1.125	0.870	0.000	0.000
DICER1	3.391	3.467	0.076	0.063	0.068
DIDO1	2.814	3.518	0.704	0.000	0.000
DIO1	6.614	6.072	-0.542	0.000	0.000
DIO2	0.113	0.688	0.575	0.000	0.000
DIO3	0.513	0.427	-0.086	0.031	0.034
DIP2A	2.292	2.742	0.450	0.000	0.000
DIP2B	3.083	3.612	0.529	0.000	0.000
DIP2C	2.909	2.852	-0.057	0.263	0.277
DIRAS1	0.187	0.565	0.377	0.000	0.000
DIRAS2	0.477	0.597	0.120	0.011	0.013
DIRAS3	2.831	1.146	-1.685	0.000	0.000
DIRC2	2.532	3.114	0.581	0.000	0.000

DIRC3	0.120	0.232	0.112	0.000	0.000
DIS3	2.922	3.249	0.327	0.000	0.000
DIS3L	3.295	3.719	0.424	0.000	0.000
DIS3L2	2.253	2.856	0.603	0.000	0.000
DISC1	0.610	0.881	0.271	0.000	0.000
DISP1	2.400	2.837	0.437	0.000	0.000
DISP2	0.231	0.632	0.400	0.000	0.000
DIXDC1	2.201	2.172	-0.029	0.478	0.494
DKC1	3.997	5.009	1.012	0.000	0.000
DKK1	0.398	2.081	1.683	0.000	0.000
DKK2	0.232	0.637	0.405	0.000	0.000
DKK3	3.085	3.363	0.278	0.001	0.001
DKK4	0.410	1.351	0.942	0.000	0.000
DKKL1	0.175	0.485	0.310	0.000	0.000
DLAT	3.451	4.370	0.918	0.000	0.000
DLC1	3.650	3.189	-0.462	0.000	0.000
DLD	5.208	5.620	0.412	0.000	0.000
DLEC1	0.894	0.562	-0.332	0.000	0.000
DLEU1	1.246	1.454	0.207	0.000	0.000
DLEU7	0.211	0.229	0.019	0.240	0.254
DLG1	3.410	3.915	0.505	0.000	0.000
DLG2	0.721	0.435	-0.286	0.000	0.000
DLG3	1.370	1.650	0.279	0.000	0.000
DLG4	2.396	2.279	-0.117	0.005	0.006
DLG5	1.635	3.015	1.380	0.000	0.000
DLGAP3	0.296	0.393	0.096	0.000	0.000
DLGAP4	3.631	4.388	0.756	0.000	0.000
DLGAP5	0.537	2.410	1.873	0.000	0.000
DLK1	0.387	1.321	0.934	0.000	0.000
DLK2	0.417	1.098	0.681	0.000	0.000
DLL1	2.522	2.228	-0.294	0.000	0.000
DLL4	1.839	3.018	1.179	0.000	0.000
DLST	5.517	5.831	0.314	0.000	0.000
DMAP1	3.837	4.388	0.551	0.000	0.000
DMC1	0.147	0.592	0.446	0.000	0.000
DMD	3.451	2.950	-0.502	0.000	0.000
DMGDH	6.337	5.030	-1.307	0.000	0.000
DMKN	1.136	1.586	0.450	0.000	0.000
DMPK	2.723	3.270	0.546	0.000	0.000
DMRTA1	2.784	2.287	-0.497	0.000	0.000
DMTF1	3.120	3.796	0.677	0.000	0.000
DMWD	2.356	3.216	0.859	0.000	0.000
DMXL1	2.737	3.030	0.293	0.000	0.000
DMXL2	2.505	3.127	0.622	0.000	0.000
DNA2	1.476	2.181	0.705	0.000	0.000
DNAAF1	0.425	0.542	0.117	0.005	0.005
DNAAF2	3.106	3.740	0.634	0.000	0.000
DNAH1	2.456	2.797	0.341	0.000	0.000
DNAH11	0.605	1.072	0.466	0.000	0.000
DNAH12	0.063	0.419	0.357	0.000	0.000
DNAH14	0.777	1.522	0.745	0.000	0.000
DNAH17	0.307	0.590	0.283	0.000	0.000

DNAH2	0.267	0.215	-0.052	0.003	0.003
DNAH5	0.522	0.721	0.199	0.000	0.000
DNAH6	0.974	0.873	-0.100	0.014	0.016
DNAI1	0.186	0.271	0.085	0.000	0.000
DNAJA1	7.342	7.690	0.348	0.000	0.000
DNAJA2	4.897	5.181	0.283	0.000	0.000
DNAJA3	4.734	4.964	0.230	0.000	0.000
DNAJA4	1.452	1.733	0.280	0.000	0.000
DNAJB1	6.299	6.686	0.387	0.000	0.000
DNAJB11	4.821	6.099	1.277	0.000	0.000
DNAJB12	4.026	4.480	0.453	0.000	0.000
DNAJB13	0.106	0.268	0.162	0.000	0.000
DNAJB14	1.961	2.179	0.217	0.000	0.000
DNAJB2	4.637	5.396	0.759	0.000	0.000
DNAJB4	3.591	3.683	0.092	0.088	0.095
DNAJB5	1.422	2.006	0.584	0.000	0.000
DNAJB6	3.105	3.983	0.878	0.000	0.000
DNAJB9	6.054	6.220	0.166	0.000	0.000
DNAJC1	5.156	5.635	0.479	0.000	0.000
DNAJC10	2.811	3.484	0.674	0.000	0.000
DNAJC11	3.867	4.280	0.413	0.000	0.000
DNAJC12	4.969	3.564	-1.404	0.000	0.000
DNAJC13	2.805	3.572	0.767	0.000	0.000
DNAJC15	3.643	3.601	-0.042	0.318	0.334
DNAJC16	3.352	3.297	-0.055	0.068	0.074
DNAJC17	3.117	3.366	0.249	0.000	0.000
DNAJC18	0.842	1.193	0.351	0.000	0.000
DNAJC19	4.676	4.896	0.220	0.000	0.000
DNAJC2	3.330	4.252	0.923	0.000	0.000
DNAJC21	3.579	4.223	0.644	0.000	0.000
DNAJC22	4.478	4.468	-0.010	0.834	0.841
DNAJC24	1.773	2.274	0.501	0.000	0.000
DNAJC25	5.236	4.739	-0.497	0.000	0.000
DNAJC25-GN(	2.110	2.331	0.221	0.000	0.000
DNAJC27	1.407	1.510	0.103	0.000	0.000
DNAJC28	1.398	1.497	0.099	0.000	0.000
DNAJC30	3.742	4.148	0.407	0.000	0.000
DNAJC4	4.736	4.878	0.142	0.003	0.004
DNAJC5	3.791	4.820	1.029	0.000	0.000
DNAJC5B	0.540	0.669	0.129	0.000	0.000
DNAJC6	0.418	1.593	1.175	0.000	0.000
DNAJC7	4.107	4.776	0.669	0.000	0.000
DNAJC8	5.349	6.065	0.717	0.000	0.000
DNAL1	1.348	1.827	0.480	0.000	0.000
DNAL4	2.659	3.557	0.898	0.000	0.000
DNALI1	3.810	2.980	-0.830	0.000	0.000
DNASE1	0.988	1.399	0.411	0.000	0.000
DNASE1L2	0.667	1.116	0.450	0.000	0.000
DNASE1L3	6.172	3.482	-2.690	0.000	0.000
DNASE2	5.126	5.752	0.626	0.000	0.000
DNASE2B	0.316	0.548	0.233	0.000	0.000
DNHD1	1.901	1.823	-0.078	0.101	0.109

DNLZ	1.614	2.082	0.468	0.000	0.000
DNM1	1.221	1.427	0.206	0.001	0.001
DNM1L	3.280	4.173	0.893	0.000	0.000
DNM2	4.080	4.754	0.673	0.000	0.000
DNM3	0.258	0.727	0.469	0.000	0.000
DNMBP	3.102	3.079	-0.023	0.553	0.567
DNMT1	2.985	3.991	1.006	0.000	0.000
DNMT3A	1.745	2.874	1.129	0.000	0.000
DNMT3B	0.744	1.657	0.914	0.000	0.000
DNMT3L	1.906	1.218	-0.688	0.000	0.000
DNPEP	5.060	5.577	0.518	0.000	0.000
DNTTIP1	4.124	4.920	0.796	0.000	0.000
DNTTIP2	3.919	4.512	0.593	0.000	0.000
DOC2B	0.418	0.567	0.150	0.000	0.000
DOCK1	3.024	3.439	0.415	0.000	0.000
DOCK10	1.906	1.717	-0.189	0.000	0.000
DOCK11	1.598	1.781	0.183	0.000	0.000
DOCK2	1.922	1.717	-0.205	0.000	0.000
DOCK3	0.166	0.348	0.182	0.000	0.000
DOCK4	2.610	3.146	0.536	0.000	0.000
DOCK5	2.563	2.475	-0.088	0.071	0.077
DOCK6	2.776	3.550	0.774	0.000	0.000
DOCK7	2.323	3.035	0.713	0.000	0.000
DOCK8	2.235	1.864	-0.370	0.000	0.000
DOCK9	2.255	2.863	0.608	0.000	0.000
DOHH	3.102	3.813	0.711	0.000	0.000
DOK1	2.046	2.250	0.204	0.000	0.000
DOK2	3.376	3.027	-0.349	0.000	0.000
DOK3	1.708	1.926	0.218	0.000	0.000
DOK4	3.925	4.540	0.615	0.000	0.000
DOK5	0.741	1.106	0.366	0.000	0.000
DOK6	0.439	0.470	0.031	0.376	0.392
DOK7	0.835	1.185	0.350	0.000	0.000
DOLK	3.574	4.537	0.963	0.000	0.000
DOLPP1	4.136	4.755	0.618	0.000	0.000
DONSON	2.902	3.943	1.041	0.000	0.000
DOT1L	2.767	3.221	0.454	0.000	0.000
DPAGT1	3.783	4.668	0.885	0.000	0.000
DPCD	2.531	3.464	0.933	0.000	0.000
DPEP1	0.641	0.998	0.357	0.000	0.000
DPEP2	1.831	1.457	-0.374	0.000	0.000
DPEP3	0.312	0.211	-0.102	0.000	0.000
DPF2	2.783	3.640	0.857	0.000	0.000
DPF3	1.445	0.656	-0.789	0.000	0.000
DPH1	3.814	3.800	-0.014	0.709	0.721
DPH2	3.291	4.381	1.090	0.000	0.000
DPH3	3.841	4.590	0.749	0.000	0.000
DPM1	5.554	6.181	0.627	0.000	0.000
DPM2	4.353	5.171	0.817	0.000	0.000
DPM3	6.435	7.299	0.864	0.000	0.000
DPP3	3.645	4.583	0.938	0.000	0.000
DPP4	4.691	5.395	0.704	0.000	0.000

DPP7	6.246	6.268	0.022	0.699	0.711
DPP8	2.060	2.613	0.553	0.000	0.000
DPP9	4.565	4.955	0.389	0.000	0.000
DPPA4	0.218	0.144	-0.074	0.000	0.000
DPT	4.915	2.006	-2.909	0.000	0.000
DPY19L1	3.350	3.734	0.384	0.000	0.000
DPY19L2	0.245	0.405	0.160	0.000	0.000
DPY19L3	2.120	2.583	0.462	0.000	0.000
DPY19L4	3.169	3.836	0.668	0.000	0.000
DPY30	4.776	5.589	0.813	0.000	0.000
DPYD	4.589	4.409	-0.180	0.002	0.002
DPYS	7.964	6.905	-1.059	0.000	0.000
DPYSL2	3.530	3.695	0.165	0.003	0.003
DPYSL3	2.309	2.489	0.180	0.009	0.010
DPYSL4	0.240	0.494	0.254	0.000	0.000
DQX1	0.069	0.959	0.890	0.000	0.000
DR1	2.830	3.562	0.733	0.000	0.000
DRAM1	2.690	3.443	0.753	0.000	0.000
DRAM2	4.216	5.053	0.836	0.000	0.000
DRAP1	5.990	6.918	0.928	0.000	0.000
DRD1	0.718	0.579	-0.139	0.003	0.004
DRD4	0.941	1.281	0.340	0.000	0.000
DRG1	4.989	5.878	0.889	0.000	0.000
DRG2	3.223	3.750	0.527	0.000	0.000
DROSHA	3.149	4.218	1.069	0.000	0.000
DSC2	1.768	2.252	0.483	0.000	0.000
DSCAML1	0.428	0.326	-0.102	0.001	0.002
DSCC1	1.013	2.500	1.486	0.000	0.000
DSE	1.932	1.449	-0.483	0.000	0.000
DSEL	1.653	1.392	-0.261	0.000	0.000
DSG1	2.398	1.990	-0.408	0.000	0.000
DSG2	3.242	3.719	0.478	0.000	0.000
DSN1	2.914	4.272	1.357	0.000	0.000
DSP	4.822	5.679	0.858	0.000	0.000
DST	4.153	4.228	0.075	0.110	0.119
DSTN	6.332	6.916	0.584	0.000	0.000
DSTYK	1.606	2.473	0.867	0.000	0.000
DTD1	2.786	3.696	0.909	0.000	0.000
DTHD1	0.404	0.241	-0.163	0.000	0.000
DTL	0.820	2.707	1.887	0.000	0.000
DTNA	1.151	2.516	1.366	0.000	0.000
DTNB	1.919	2.561	0.642	0.000	0.000
DTNBP1	2.556	3.447	0.891	0.000	0.000
DTWD1	1.829	2.261	0.433	0.000	0.000
DTWD2	2.087	2.381	0.295	0.000	0.000
DTX1	4.351	2.730	-1.621	0.000	0.000
DTX2	2.594	3.261	0.668	0.000	0.000
DTX3	2.300	2.497	0.197	0.002	0.003
DTX3L	4.691	5.263	0.572	0.000	0.000
DTX4	3.604	3.619	0.015	0.831	0.838
DTYMK	3.280	4.870	1.590	0.000	0.000
DUOX1	0.303	0.977	0.674	0.000	0.000

DUOX2	0.407	0.932	0.525	0.000	0.000
DUOXA1	0.082	0.301	0.219	0.000	0.000
DUOXA2	0.282	1.019	0.736	0.000	0.000
DUS1L	5.529	6.364	0.835	0.000	0.000
DUS3L	3.666	4.085	0.419	0.000	0.000
DUS4L	1.575	2.464	0.889	0.000	0.000
DUSP1	8.727	7.662	-1.064	0.000	0.000
DUSP10	4.947	4.710	-0.237	0.000	0.000
DUSP11	3.244	3.717	0.473	0.000	0.000
DUSP12	3.219	4.385	1.166	0.000	0.000
DUSP15	0.346	0.677	0.331	0.000	0.000
DUSP16	4.746	4.720	-0.026	0.584	0.599
DUSP18	0.722	1.194	0.472	0.000	0.000
DUSP19	0.921	1.152	0.232	0.000	0.000
DUSP2	3.175	2.462	-0.713	0.000	0.000
DUSP22	3.306	3.970	0.664	0.000	0.000
DUSP23	6.761	7.826	1.064	0.000	0.000
DUSP26	0.294	0.462	0.168	0.000	0.000
DUSP28	1.366	1.981	0.615	0.000	0.000
DUSP3	5.645	6.288	0.643	0.000	0.000
DUSP4	1.063	1.280	0.217	0.000	0.000
DUSP5	4.824	3.876	-0.948	0.000	0.000
DUSP6	5.900	5.152	-0.748	0.000	0.000
DUSP7	1.968	2.757	0.790	0.000	0.000
DUSP8	2.022	2.780	0.759	0.000	0.000
DUSP9	0.663	3.190	2.527	0.000	0.000
DUT	4.455	5.438	0.983	0.000	0.000
DVL1	5.047	5.460	0.413	0.000	0.000
DVL2	2.613	3.690	1.076	0.000	0.000
DVL3	4.308	5.163	0.855	0.000	0.000
DYDC2	0.236	1.239	1.002	0.000	0.000
DYM	2.843	3.652	0.809	0.000	0.000
DYNC1H1	4.456	5.384	0.928	0.000	0.000
DYNC1I1	0.400	1.348	0.949	0.000	0.000
DYNC1I2	4.289	4.853	0.564	0.000	0.000
DYNC1LI1	3.128	3.828	0.701	0.000	0.000
DYNC1LI2	3.728	4.068	0.339	0.000	0.000
DYNC2H1	0.916	0.892	-0.023	0.502	0.517
DYNC2LI1	2.420	3.124	0.704	0.000	0.000
DYNLL1	6.373	7.410	1.037	0.000	0.000
DYNLL2	4.662	4.870	0.208	0.000	0.000
DYNLRB1	5.689	6.738	1.049	0.000	0.000
DYNLRB2	0.522	0.445	-0.077	0.003	0.003
DYNLT1	5.094	5.662	0.568	0.000	0.000
DYNLT3	4.502	5.037	0.535	0.000	0.000
DYRK1A	3.317	3.688	0.371	0.000	0.000
DYRK1B	2.649	3.438	0.789	0.000	0.000
DYRK2	1.864	2.624	0.759	0.000	0.000
DYRK3	1.520	1.866	0.345	0.000	0.000
DYRK4	2.398	2.698	0.301	0.000	0.000
DYSF	3.519	3.751	0.232	0.000	0.000
DZIP1	1.295	1.150	-0.145	0.004	0.005

DZIP1L	0.833	1.032	0.199	0.000	0.000
DZIP3	1.858	2.624	0.766	0.000	0.000
E2F1	1.336	3.924	2.588	0.000	0.000
E2F2	0.361	1.166	0.806	0.000	0.000
E2F3	2.183	3.204	1.021	0.000	0.000
E2F4	4.136	4.843	0.707	0.000	0.000
E2F5	1.248	2.247	0.999	0.000	0.000
E2F6	2.431	3.163	0.732	0.000	0.000
E2F7	0.216	0.995	0.779	0.000	0.000
E2F8	0.394	1.731	1.338	0.000	0.000
E4F1	3.013	3.784	0.770	0.000	0.000
EAF1	3.596	4.042	0.446	0.000	0.000
EAF2	1.678	2.137	0.459	0.000	0.000
EAPP	5.018	5.391	0.374	0.000	0.000
EARS2	3.614	4.036	0.421	0.000	0.000
EBAG9	4.239	4.844	0.606	0.000	0.000
EBF1	0.607	1.317	0.711	0.000	0.000
EBF2	0.146	0.475	0.329	0.000	0.000
EBF3	0.444	0.798	0.354	0.000	0.000
EBF4	3.015	2.454	-0.561	0.000	0.000
EBI3	2.609	2.303	-0.305	0.000	0.000
EBLN2	1.202	1.369	0.167	0.000	0.000
EBNA1BP2	5.380	5.756	0.376	0.000	0.000
EBP	7.525	7.723	0.198	0.000	0.000
EBPL	6.643	6.619	-0.025	0.570	0.584
ECD	3.459	4.234	0.775	0.000	0.000
ECE1	5.052	5.100	0.047	0.214	0.227
ECE2	2.205	2.980	0.775	0.000	0.000
ECEL1	0.244	0.505	0.260	0.000	0.000
ECH1	8.210	8.149	-0.061	0.108	0.117
ECHDC1	4.854	4.829	-0.025	0.581	0.596
ECHDC2	6.619	5.933	-0.686	0.000	0.000
ECHDC3	6.218	5.754	-0.464	0.000	0.000
ECHS1	9.968	9.171	-0.797	0.000	0.000
ECI1	6.567	6.642	0.075	0.029	0.033
ECI2	6.823	6.838	0.015	0.712	0.723
ECM1	5.084	2.632	-2.452	0.000	0.000
ECM2	4.457	3.761	-0.696	0.000	0.000
ECSCR	2.819	3.440	0.621	0.000	0.000
ECSIT	5.227	5.546	0.319	0.000	0.000
ECT2	1.161	3.157	1.996	0.000	0.000
ECT2L	0.161	0.151	-0.010	0.165	0.176
EDA	1.016	1.287	0.271	0.000	0.000
EDA2R	0.932	1.104	0.171	0.001	0.001
EDAR	0.718	0.668	-0.049	0.217	0.230
EDARADD	0.381	1.086	0.705	0.000	0.000
EDC3	2.981	3.886	0.905	0.000	0.000
EDC4	3.572	3.975	0.403	0.000	0.000
EDEM1	5.015	4.867	-0.148	0.000	0.000
EDEM2	4.939	5.750	0.811	0.000	0.000
EDEM3	3.971	4.758	0.787	0.000	0.000
EDF1	8.835	9.209	0.375	0.000	0.000

EDIL3	0.649	1.487	0.838	0.000	0.000
EDN1	2.332	2.084	-0.249	0.000	0.000
EDN2	1.054	0.573	-0.481	0.000	0.000
EDNRA	1.456	1.754	0.297	0.000	0.000
EDNRB	4.495	3.519	-0.976	0.000	0.000
EEA1	2.663	3.179	0.516	0.000	0.000
EED	2.268	3.064	0.796	0.000	0.000
EEF1A2	1.779	3.731	1.952	0.000	0.000
EEF1B2	7.635	8.247	0.612	0.000	0.000
EEF1D	6.035	6.827	0.792	0.000	0.000
EEF1G	4.867	5.466	0.599	0.000	0.000
EEF2	9.427	9.762	0.335	0.000	0.000
EEF2K	2.691	3.352	0.660	0.000	0.000
EEFSEC	4.300	4.759	0.460	0.000	0.000
EEPD1	3.626	4.135	0.508	0.000	0.000
EFCAB1	0.359	0.239	-0.120	0.000	0.000
EFCAB10	0.629	0.927	0.298	0.000	0.000
EFCAB11	1.047	1.536	0.490	0.000	0.000
EFCAB2	1.635	2.354	0.719	0.000	0.000
EFCAB6	0.434	0.360	-0.073	0.000	0.000
EFCAB7	0.962	1.537	0.575	0.000	0.000
EFEMP1	3.403	3.466	0.063	0.591	0.605
EFEMP2	2.969	2.867	-0.102	0.082	0.089
EFHB	0.171	0.252	0.081	0.000	0.000
EFHC1	1.887	2.305	0.418	0.000	0.000
EFHD1	3.786	3.086	-0.700	0.000	0.000
EFHD2	5.079	5.663	0.584	0.000	0.000
EFNA1	7.115	8.202	1.087	0.000	0.000
EFNA2	2.827	3.195	0.368	0.000	0.000
EFNA4	2.506	4.175	1.669	0.000	0.000
EFNA5	0.598	0.763	0.165	0.006	0.006
EFNB1	3.520	3.991	0.472	0.000	0.000
EFNB2	2.525	3.178	0.653	0.000	0.000
EFNB3	1.153	0.546	-0.607	0.000	0.000
EFR3A	4.560	5.303	0.743	0.000	0.000
EFR3B	0.182	0.247	0.064	0.000	0.000
EFS	1.083	0.859	-0.224	0.000	0.000
EFTUD2	3.595	4.630	1.035	0.000	0.000
EGFL6	0.108	0.475	0.367	0.000	0.000
EGFL7	4.937	5.287	0.350	0.000	0.000
EGFL8	2.041	2.412	0.371	0.000	0.000
EGFLAM	1.181	1.397	0.216	0.000	0.000
EGFR	4.040	4.077	0.037	0.515	0.530
EGLN1	4.409	5.174	0.765	0.000	0.000
EGLN3	1.076	1.896	0.820	0.000	0.000
EGR1	7.565	5.366	-2.199	0.000	0.000
EGR2	2.558	1.528	-1.030	0.000	0.000
EGR3	1.451	0.894	-0.557	0.000	0.000
EHBP1	4.433	4.481	0.048	0.237	0.250
EHBP1L1	3.955	4.338	0.383	0.000	0.000
EHD1	3.753	4.121	0.369	0.000	0.000
EHD2	3.209	4.006	0.797	0.000	0.000

EHD3	2.917	1.887	-1.030	0.000	0.000
EHD4	3.100	4.012	0.911	0.000	0.000
EHF	1.659	2.402	0.744	0.000	0.000
EHHADH	7.526	6.461	-1.065	0.000	0.000
EHMT1	3.104	3.424	0.320	0.000	0.000
EHMT2	3.026	4.544	1.518	0.000	0.000
EI24	6.782	6.813	0.032	0.341	0.356
EID1	6.404	6.748	0.343	0.000	0.000
EID2	3.304	3.919	0.615	0.000	0.000
EID2B	0.850	1.526	0.676	0.000	0.000
EID3	1.050	1.406	0.356	0.000	0.000
EIF1	8.318	8.818	0.500	0.000	0.000
EIF1AD	3.439	4.263	0.824	0.000	0.000
EIF1AX	5.092	5.568	0.476	0.000	0.000
EIF1AY	2.847	2.815	-0.032	0.778	0.788
EIF1B	5.443	5.786	0.343	0.000	0.000
EIF2A	5.126	5.859	0.732	0.000	0.000
EIF2AK1	5.557	6.441	0.885	0.000	0.000
EIF2AK2	2.451	3.013	0.562	0.000	0.000
EIF2AK3	3.013	3.341	0.328	0.000	0.000
EIF2AK4	3.983	4.037	0.054	0.122	0.131
EIF2B1	4.312	5.183	0.871	0.000	0.000
EIF2B2	3.034	3.830	0.796	0.000	0.000
EIF2B3	3.830	4.245	0.415	0.000	0.000
EIF2B4	3.896	4.670	0.774	0.000	0.000
EIF2B5	4.129	4.763	0.634	0.000	0.000
EIF2D	3.510	4.659	1.149	0.000	0.000
EIF2S1	4.354	4.949	0.596	0.000	0.000
EIF2S2	5.503	6.317	0.814	0.000	0.000
EIF2S3	5.688	6.611	0.922	0.000	0.000
EIF3A	5.544	6.179	0.634	0.000	0.000
EIF3B	4.970	6.024	1.054	0.000	0.000
EIF3D	5.686	6.689	1.003	0.000	0.000
EIF3E	6.292	7.259	0.967	0.000	0.000
EIF3F	4.641	5.312	0.671	0.000	0.000
EIF3G	6.098	6.787	0.689	0.000	0.000
EIF3H	5.312	6.365	1.053	0.000	0.000
EIF3I	7.012	7.644	0.632	0.000	0.000
EIF3J	5.325	5.807	0.482	0.000	0.000
EIF3K	6.492	7.344	0.852	0.000	0.000
EIF3L	6.187	6.702	0.515	0.000	0.000
EIF3M	5.168	5.929	0.761	0.000	0.000
EIF4A1	4.534	4.942	0.409	0.000	0.000
EIF4A2	6.633	7.239	0.606	0.000	0.000
EIF4A3	4.304	5.275	0.971	0.000	0.000
EIF4B	6.323	6.851	0.528	0.000	0.000
EIF4E	2.525	2.869	0.344	0.000	0.000
EIF4E2	4.337	4.856	0.519	0.000	0.000
EIF4E3	2.210	1.819	-0.391	0.000	0.000
EIF4EBP1	5.583	6.213	0.630	0.000	0.000
EIF4EBP2	5.507	6.120	0.614	0.000	0.000
EIF4EBP3	4.831	5.120	0.290	0.000	0.000

EIF4ENIF1	2.590	3.330	0.740	0.000	0.000
EIF4G1	6.793	7.201	0.407	0.000	0.000
EIF4G2	6.626	7.352	0.726	0.000	0.000
EIF4G3	3.370	4.071	0.701	0.000	0.000
EIF4H	6.674	7.242	0.568	0.000	0.000
EIF5	6.377	6.266	-0.112	0.000	0.001
EIF5A	7.578	7.688	0.110	0.001	0.001
EIF5A2	1.062	1.893	0.831	0.000	0.000
EIF5B	4.635	5.339	0.704	0.000	0.000
EIF6	6.969	7.814	0.845	0.000	0.000
ELAC1	2.329	2.589	0.260	0.000	0.000
ELAC2	4.343	4.632	0.289	0.000	0.000
ELANE	0.263	0.247	-0.016	0.506	0.521
ELAVL1	3.815	4.652	0.836	0.000	0.000
ELF1	4.236	4.322	0.086	0.083	0.090
ELF2	2.969	3.112	0.143	0.000	0.000
ELF3	4.565	5.197	0.633	0.000	0.000
ELF4	1.793	2.078	0.285	0.000	0.000
ELFN1	3.521	2.774	-0.746	0.000	0.000
ELFN2	0.387	1.347	0.960	0.000	0.000
ELK1	4.006	4.526	0.521	0.000	0.000
ELK3	2.597	3.120	0.523	0.000	0.000
ELK4	3.132	3.856	0.725	0.000	0.000
ELL	3.139	3.337	0.198	0.000	0.000
ELL2	5.462	5.493	0.031	0.523	0.537
ELL3	0.973	1.589	0.616	0.000	0.000
ELMO1	2.987	3.559	0.572	0.000	0.000
ELMO2	3.106	3.647	0.540	0.000	0.000
ELMO3	3.059	2.931	-0.128	0.061	0.066
ELMOD2	3.091	3.602	0.511	0.000	0.000
ELMOD3	2.558	3.180	0.622	0.000	0.000
ELN	3.160	2.773	-0.387	0.000	0.000
ELOF1	4.412	4.742	0.330	0.000	0.000
ELOVL1	4.680	5.940	1.260	0.000	0.000
ELOVL2	4.427	5.611	1.184	0.000	0.000
ELOVL3	0.159	0.489	0.330	0.000	0.000
ELOVL4	0.201	0.527	0.326	0.000	0.000
ELOVL5	5.496	6.371	0.876	0.000	0.000
ELOVL6	4.207	3.974	-0.233	0.000	0.000
ELOVL7	1.272	2.042	0.770	0.000	0.000
ELP2	4.256	4.383	0.127	0.000	0.000
ELP3	3.640	3.950	0.310	0.000	0.000
ELP4	3.034	3.606	0.572	0.000	0.000
EMB	2.079	1.955	-0.124	0.052	0.057
EMCN	2.074	2.702	0.628	0.000	0.000
EMD	5.216	6.011	0.795	0.000	0.000
EME1	1.218	2.484	1.266	0.000	0.000
EME2	1.311	1.716	0.405	0.000	0.000
EMG1	3.307	3.984	0.677	0.000	0.000
EMID1	1.848	2.255	0.407	0.000	0.000
EMILIN1	4.638	3.599	-1.039	0.000	0.000
EMILIN2	1.360	2.108	0.747	0.000	0.000

EMILIN3	1.249	1.257	0.008	0.827	0.834
EML1	1.556	1.532	-0.025	0.530	0.545
EML2	2.430	3.226	0.796	0.000	0.000
EML3	4.233	4.498	0.264	0.000	0.000
EML4	4.796	5.403	0.608	0.000	0.000
EML5	0.569	0.717	0.148	0.000	0.000
EML6	0.375	1.171	0.796	0.000	0.000
EMP1	3.236	3.359	0.123	0.101	0.108
EMP2	4.549	4.760	0.211	0.000	0.000
EMP3	4.236	4.125	-0.111	0.088	0.095
EMX1	0.105	0.786	0.680	0.000	0.000
ENAH	2.239	4.101	1.863	0.000	0.000
ENAM	0.467	0.696	0.229	0.000	0.000
ENC1	3.963	3.742	-0.222	0.000	0.000
ENDOD1	2.740	2.508	-0.232	0.000	0.000
ENDOG	5.372	5.280	-0.092	0.027	0.030
ENDOU	0.183	0.162	-0.021	0.065	0.071
ENDOV	2.464	2.608	0.144	0.000	0.000
ENG	5.769	5.319	-0.450	0.000	0.000
ENGASE	3.540	4.034	0.494	0.000	0.000
ENHO	3.722	3.885	0.163	0.104	0.112
ENKUR	0.269	0.392	0.123	0.000	0.000
ENO1	8.028	8.961	0.933	0.000	0.000
ENO2	1.882	1.977	0.095	0.193	0.205
ENO3	5.519	3.980	-1.539	0.000	0.000
ENOPH1	4.217	5.136	0.918	0.000	0.000
ENOSF1	4.174	4.440	0.266	0.000	0.000
ENOX1	0.344	0.684	0.340	0.000	0.000
ENOX2	2.152	2.849	0.697	0.000	0.000
ENPEP	4.317	3.730	-0.588	0.000	0.000
ENPP1	4.749	4.461	-0.289	0.000	0.000
ENPP2	3.551	4.288	0.738	0.000	0.000
ENPP3	1.239	1.646	0.407	0.000	0.000
ENPP4	2.683	3.463	0.780	0.000	0.000
ENPP5	0.957	1.144	0.187	0.009	0.010
ENPP6	0.139	0.461	0.322	0.000	0.000
ENPP7	3.210	3.289	0.079	0.364	0.380
ENSA	5.487	6.454	0.967	0.000	0.000
ENTPD1	1.927	2.653	0.727	0.000	0.000
ENTPD2	1.141	2.002	0.861	0.000	0.000
ENTPD3	0.162	0.296	0.134	0.000	0.000
ENTPD4	2.712	3.035	0.323	0.000	0.000
ENTPD5	5.046	4.972	-0.074	0.245	0.259
ENTPD6	4.418	5.470	1.052	0.000	0.000
ENTPD7	2.750	3.149	0.399	0.000	0.000
ENTPD8	3.615	2.827	-0.788	0.000	0.000
ENY2	4.840	5.678	0.838	0.000	0.000
EOMES	1.380	0.903	-0.477	0.000	0.000
EP300	3.354	3.902	0.548	0.000	0.000
EP400	2.089	2.722	0.632	0.000	0.000
EPAS1	6.320	6.151	-0.169	0.000	0.000
EPB41	3.798	4.213	0.415	0.000	0.000

EPB41L1	2.445	2.975	0.530	0.000	0.000
EPB41L2	3.221	3.513	0.292	0.000	0.000
EPB41L3	1.721	1.506	-0.215	0.000	0.000
EPB41L4A	1.706	1.298	-0.408	0.000	0.000
EPB41L4B	4.975	4.108	-0.867	0.000	0.000
EPB41L5	3.878	3.945	0.067	0.098	0.106
EPC1	2.470	2.858	0.388	0.000	0.000
EPC2	2.677	3.117	0.440	0.000	0.000
EPCAM	3.266	2.900	-0.366	0.018	0.020
EPDR1	3.189	4.066	0.877	0.000	0.000
EPG5	2.204	2.649	0.444	0.000	0.000
EPHA1	3.526	3.979	0.453	0.000	0.000
EPHA2	4.665	3.515	-1.149	0.000	0.000
EPHA3	1.991	1.454	-0.537	0.000	0.000
EPHA4	0.770	1.004	0.234	0.000	0.000
EPHB1	0.815	0.408	-0.407	0.000	0.000
EPHB2	0.522	1.294	0.772	0.000	0.000
EPHB3	0.968	0.972	0.004	0.942	0.945
EPHB4	4.212	5.014	0.802	0.000	0.000
EPHB6	1.929	1.633	-0.296	0.000	0.000
EPHX1	10.070	10.619	0.548	0.000	0.000
EPHX2	7.198	5.943	-1.255	0.000	0.000
EPHX3	0.415	0.490	0.076	0.037	0.041
EPHX4	0.091	0.479	0.388	0.000	0.000
EPM2A	2.201	1.948	-0.253	0.000	0.000
EPM2AIP1	3.151	3.495	0.344	0.000	0.000
EPN1	4.628	5.083	0.455	0.000	0.000
EPN2	2.394	2.779	0.385	0.000	0.000
EPN3	0.223	0.341	0.118	0.002	0.002
EPO	2.388	1.459	-0.929	0.000	0.000
EPOR	3.473	3.182	-0.292	0.000	0.000
EPRS	4.532	5.844	1.311	0.000	0.000
EPS15	3.942	4.257	0.315	0.000	0.000
EPS15L1	2.387	3.154	0.767	0.000	0.000
EPS8	4.132	4.538	0.406	0.000	0.000
EPS8L1	1.514	1.114	-0.399	0.000	0.000
EPS8L2	5.886	6.093	0.208	0.000	0.000
EPS8L3	0.603	2.968	2.365	0.000	0.000
EPSTI1	2.754	2.606	-0.147	0.035	0.039
ERAL1	5.029	5.887	0.858	0.000	0.000
ERAP1	4.073	4.667	0.594	0.000	0.000
ERAP2	3.001	3.369	0.368	0.000	0.000
ERAS	0.397	0.392	-0.005	0.834	0.841
ERBB2	4.227	4.301	0.074	0.251	0.265
ERBB3	5.288	6.256	0.968	0.000	0.000
ERC1	2.255	2.618	0.363	0.000	0.000
ERCC1	3.858	4.528	0.669	0.000	0.000
ERCC2	2.744	3.696	0.952	0.000	0.000
ERCC3	3.246	3.963	0.717	0.000	0.000
ERCC4	1.599	1.820	0.221	0.000	0.000
ERCC5	4.203	4.353	0.151	0.000	0.000
ERCC6	1.214	1.437	0.223	0.000	0.000

ERCC6L	0.258	1.099	0.841	0.000	0.000
ERCC8	1.563	2.193	0.630	0.000	0.000
ERF	5.051	5.621	0.571	0.000	0.000
ERG	2.238	1.996	-0.242	0.000	0.000
ERGIC1	5.540	5.686	0.146	0.000	0.000
ERGIC2	3.988	4.443	0.454	0.000	0.000
ERGIC3	5.953	6.770	0.817	0.000	0.000
ERH	6.192	6.941	0.749	0.000	0.000
ERI1	1.923	2.458	0.535	0.000	0.000
ERI2	3.292	3.254	-0.038	0.300	0.316
ERI3	4.593	5.483	0.890	0.000	0.000
ERICH1	2.024	2.237	0.213	0.000	0.000
ERLEC1	5.078	6.013	0.934	0.000	0.000
ERLIN1	5.282	4.836	-0.446	0.000	0.000
ERLIN2	3.911	4.023	0.112	0.006	0.007
ERMAP	3.269	3.377	0.109	0.002	0.003
ERMN	0.454	0.377	-0.077	0.002	0.002
ERMP1	2.705	3.772	1.068	0.000	0.000
ERN1	3.039	3.009	-0.030	0.543	0.558
ERP27	0.725	1.049	0.323	0.000	0.000
ERP29	6.952	7.235	0.283	0.000	0.000
ERP44	5.013	5.441	0.428	0.000	0.000
ERRFI1	8.190	7.192	-0.998	0.000	0.000
ERV3-1	1.451	2.130	0.679	0.000	0.000
ERVFRD-1	0.231	0.102	-0.129	0.000	0.000
ESAM	3.546	4.278	0.732	0.000	0.000
ESCO1	2.807	3.440	0.634	0.000	0.000
ESCO2	0.283	1.041	0.758	0.000	0.000
ESD	6.178	6.331	0.153	0.000	0.000
ESF1	3.736	4.533	0.798	0.000	0.000
ESM1	0.675	3.259	2.584	0.000	0.000
ESPL1	1.151	2.194	1.043	0.000	0.000
ESPN	5.567	5.408	-0.159	0.012	0.013
ESPNL	0.757	0.949	0.192	0.000	0.000
ESR1	2.753	1.152	-1.601	0.000	0.000
ESRP1	0.999	0.903	-0.096	0.287	0.302
ESRP2	4.852	4.634	-0.218	0.000	0.000
ESRRA	5.385	6.016	0.631	0.000	0.000
ESRRG	0.662	0.347	-0.315	0.000	0.000
ESYT1	5.210	5.962	0.752	0.000	0.000
ESYT2	4.169	4.662	0.493	0.000	0.000
ESYT3	0.148	0.466	0.317	0.000	0.000
ETAA1	2.692	3.371	0.679	0.000	0.000
ETF1	5.071	5.624	0.553	0.000	0.000
ETFA	7.010	6.999	-0.012	0.729	0.740
ETFB	7.010	7.134	0.124	0.002	0.002
ETFDH	6.298	5.122	-1.175	0.000	0.000
ETHE1	5.236	5.256	0.020	0.611	0.625
ETNK2	6.316	5.795	-0.521	0.000	0.000
ETS1	4.188	4.058	-0.131	0.022	0.024
ETS2	7.250	6.432	-0.819	0.000	0.000
ETV1	1.356	1.758	0.402	0.000	0.000

ETV2	1.112	1.558	0.446	0.000	0.000
ETV3	1.831	2.484	0.652	0.000	0.000
ETV4	1.460	2.948	1.489	0.000	0.000
ETV5	2.495	2.992	0.497	0.000	0.000
ETV7	1.745	2.002	0.257	0.000	0.000
EVC	2.023	1.711	-0.312	0.000	0.000
EVC2	0.908	0.952	0.044	0.358	0.374
EVI2A	2.577	2.593	0.016	0.796	0.805
EVI2B	3.589	3.201	-0.388	0.000	0.000
EVI5	2.868	3.002	0.134	0.000	0.000
EVI5L	1.823	2.564	0.740	0.000	0.000
EVL	4.040	3.966	-0.074	0.183	0.195
EVPL	0.266	0.765	0.500	0.000	0.000
EVPLL	0.777	0.783	0.006	0.892	0.896
EWSR1	5.162	5.756	0.594	0.000	0.000
EXD2	2.370	2.827	0.457	0.000	0.000
EXD3	2.243	2.356	0.113	0.004	0.004
EXO1	0.429	2.050	1.621	0.000	0.000
EXOC1	3.035	3.478	0.443	0.000	0.000
EXOC2	3.095	3.945	0.850	0.000	0.000
EXOC3	3.623	4.293	0.670	0.000	0.000
EXOC3L1	1.964	2.300	0.336	0.000	0.000
EXOC3L2	2.588	3.124	0.536	0.000	0.000
EXOC3L4	5.384	4.502	-0.882	0.000	0.000
EXOC4	3.111	4.006	0.896	0.000	0.000
EXOC5	2.725	3.155	0.430	0.000	0.000
EXOC6	2.754	3.517	0.763	0.000	0.000
EXOC6B	1.301	1.741	0.440	0.000	0.000
EXOC7	3.810	4.605	0.795	0.000	0.000
EXOC8	2.569	3.316	0.747	0.000	0.000
EXOG	1.468	2.181	0.713	0.000	0.000
EXOSC1	3.428	4.280	0.852	0.000	0.000
EXOSC10	3.837	4.487	0.650	0.000	0.000
EXOSC2	3.128	3.755	0.628	0.000	0.000
EXOSC3	3.173	3.948	0.775	0.000	0.000
EXOSC4	4.679	5.783	1.105	0.000	0.000
EXOSC5	4.185	5.233	1.048	0.000	0.000
EXOSC6	3.150	3.213	0.064	0.041	0.045
EXOSC7	3.449	4.032	0.583	0.000	0.000
EXOSC8	3.545	4.126	0.581	0.000	0.000
EXOSC9	3.044	3.800	0.756	0.000	0.000
EXPH5	2.002	1.175	-0.827	0.000	0.000
EXT1	4.427	5.028	0.601	0.000	0.000
EXT2	4.032	4.715	0.683	0.000	0.000
EXTL2	2.758	3.191	0.433	0.000	0.000
EXTL3	1.957	2.730	0.773	0.000	0.000
EYA2	1.201	0.675	-0.526	0.000	0.000
EYA3	2.376	2.896	0.520	0.000	0.000
EYS	0.383	0.540	0.158	0.000	0.000
EZH1	2.915	3.638	0.723	0.000	0.000
EZH2	1.648	3.391	1.743	0.000	0.000
EZR	5.744	5.692	-0.052	0.381	0.397

F10	7.370	7.356	-0.013	0.843	0.850
F11	7.182	5.699	-1.482	0.000	0.000
F12	8.906	7.980	-0.926	0.000	0.000
F13A1	1.529	2.409	0.879	0.000	0.000
F13B	6.743	6.368	-0.375	0.000	0.000
F2	10.346	9.704	-0.643	0.000	0.000
F2R	3.739	3.808	0.070	0.313	0.328
F2RL1	3.138	3.277	0.139	0.123	0.132
F2RL2	1.116	0.999	-0.117	0.023	0.026
F2RL3	0.678	1.296	0.618	0.000	0.000
F3	2.527	1.823	-0.704	0.000	0.000
F5	7.500	7.756	0.257	0.000	0.000
F7	6.872	6.408	-0.464	0.000	0.000
F8	2.700	2.298	-0.402	0.000	0.000
F9	8.516	6.277	-2.239	0.000	0.000
FA2H	0.454	0.497	0.043	0.435	0.451
FAAH	5.405	4.888	-0.517	0.000	0.000
FAAH2	3.751	3.407	-0.344	0.000	0.000
FABP1	10.933	9.351	-1.581	0.000	0.000
FABP3	3.372	3.292	-0.080	0.272	0.287
FABP4	2.673	3.511	0.838	0.000	0.000
FABP5	2.228	3.926	1.698	0.000	0.000
FADS1	3.153	4.384	1.231	0.000	0.000
FADS2	3.431	4.698	1.268	0.000	0.000
FADS3	3.045	3.725	0.681	0.000	0.000
FADS6	2.028	1.797	-0.230	0.008	0.009
FAF1	3.432	4.255	0.823	0.000	0.000
FAF2	3.826	4.595	0.769	0.000	0.000
FAH	6.727	6.074	-0.653	0.000	0.000
FAHD1	5.502	5.720	0.218	0.000	0.000
FAHD2A	4.811	4.105	-0.707	0.000	0.000
FAHD2B	1.805	2.024	0.219	0.001	0.001
FAIM	2.027	2.971	0.945	0.000	0.000
FAM102A	4.855	4.736	-0.118	0.052	0.057
FAM102B	1.728	2.028	0.301	0.000	0.000
FAM104B	2.910	3.386	0.476	0.000	0.000
FAM107A	1.990	1.806	-0.184	0.000	0.000
FAM107B	5.519	5.520	0.001	0.986	0.987
FAM110A	1.897	2.772	0.875	0.000	0.000
FAM110B	2.142	2.566	0.424	0.000	0.000
FAM110C	4.052	3.157	-0.895	0.000	0.000
FAM111A	3.196	3.865	0.669	0.000	0.000
FAM111B	0.919	2.592	1.673	0.000	0.000
FAM114A1	4.674	5.169	0.495	0.000	0.000
FAM114A2	2.669	3.267	0.598	0.000	0.000
FAM117A	3.035	3.267	0.233	0.000	0.000
FAM117B	1.525	1.934	0.409	0.000	0.000
FAM118A	2.704	3.265	0.561	0.000	0.000
FAM118B	2.224	2.955	0.731	0.000	0.000
FAM120A	5.188	5.790	0.602	0.000	0.000
FAM120AOS	3.724	4.493	0.769	0.000	0.000
FAM120B	3.009	3.305	0.296	0.000	0.000

FAM120C	1.521	1.876	0.356	0.000	0.000
FAM122A	3.173	3.213	0.040	0.194	0.206
FAM122B	2.964	3.922	0.958	0.000	0.000
FAM122C	0.777	1.015	0.238	0.000	0.000
FAM124A	0.595	0.597	0.002	0.936	0.939
FAM124B	0.710	1.196	0.486	0.000	0.000
FAM126A	1.772	2.169	0.397	0.000	0.000
FAM126B	2.224	2.318	0.093	0.005	0.005
FAM129A	1.574	1.913	0.339	0.000	0.000
FAM129B	4.209	4.532	0.323	0.000	0.000
FAM129C	0.554	0.293	-0.261	0.000	0.000
FAM131A	1.862	2.512	0.649	0.000	0.000
FAM131B	0.152	0.266	0.115	0.000	0.000
FAM131C	0.257	0.459	0.201	0.000	0.000
FAM133A	0.179	1.250	1.071	0.000	0.000
FAM133B	3.124	3.431	0.307	0.000	0.000
FAM135A	1.540	2.147	0.608	0.000	0.000
FAM135B	0.139	0.239	0.100	0.000	0.000
FAM136A	4.692	5.440	0.748	0.000	0.000
FAM13A	3.790	2.468	-1.322	0.000	0.000
FAM13B	2.512	2.912	0.399	0.000	0.000
FAM13C	0.925	1.495	0.570	0.000	0.000
FAM149A	3.841	3.019	-0.822	0.000	0.000
FAM149B1	2.970	3.506	0.536	0.000	0.000
FAM151A	2.875	1.705	-1.169	0.000	0.000
FAM151B	0.702	1.002	0.300	0.000	0.000
FAM155A	0.140	0.245	0.105	0.000	0.000
FAM155B	0.263	0.799	0.537	0.000	0.000
FAM160A1	0.496	0.416	-0.080	0.002	0.003
FAM160A2	4.546	4.738	0.193	0.000	0.000
FAM160B1	3.658	3.957	0.299	0.000	0.000
FAM160B2	3.208	3.557	0.349	0.000	0.000
FAM161A	0.968	1.520	0.551	0.000	0.000
FAM161B	1.318	1.711	0.393	0.000	0.000
FAM162A	6.171	6.078	-0.094	0.012	0.013
FAM162B	0.877	1.491	0.613	0.000	0.000
FAM163A	0.249	0.186	-0.064	0.003	0.004
FAM163B	2.359	0.830	-1.529	0.000	0.000
FAM166A	1.498	1.822	0.323	0.000	0.000
FAM166B	0.910	0.977	0.067	0.040	0.044
FAM167A	0.479	0.507	0.028	0.507	0.522
FAM167B	3.925	3.343	-0.582	0.000	0.000
FAM168A	2.400	3.034	0.634	0.000	0.000
FAM168B	4.266	5.284	1.017	0.000	0.000
FAM169A	1.519	2.222	0.703	0.000	0.000
FAM169B	0.756	0.543	-0.213	0.000	0.000
FAM170A	0.274	0.236	-0.038	0.045	0.049
FAM171A1	3.858	3.740	-0.118	0.183	0.195
FAM171A2	0.277	0.930	0.653	0.000	0.000
FAM171B	0.465	0.752	0.286	0.000	0.000
FAM172A	2.282	2.872	0.590	0.000	0.000
FAM173A	4.050	4.535	0.485	0.000	0.000

FAM173B	3.096	3.860	0.764	0.000	0.000
FAM174A	4.402	4.900	0.499	0.000	0.000
FAM174B	2.028	2.336	0.308	0.000	0.000
FAM177A1	4.156	4.556	0.400	0.000	0.000
FAM177B	0.395	0.444	0.048	0.110	0.118
FAM180A	2.711	0.993	-1.717	0.000	0.000
FAM182B	0.133	0.266	0.133	0.000	0.000
FAM183A	0.272	0.642	0.370	0.000	0.000
FAM184A	1.824	2.162	0.337	0.000	0.000
FAM185A	2.456	2.816	0.360	0.000	0.000
FAM186B	0.467	0.580	0.113	0.000	0.000
FAM189A2	0.659	0.612	-0.047	0.133	0.142
FAM189B	2.355	4.149	1.794	0.000	0.000
FAM192A	4.474	5.133	0.659	0.000	0.000
FAM193A	2.882	3.513	0.631	0.000	0.000
FAM193B	4.138	4.530	0.392	0.000	0.000
FAM198A	2.150	1.219	-0.932	0.000	0.000
FAM198B	2.090	2.796	0.706	0.000	0.000
FAM199X	3.037	3.859	0.822	0.000	0.000
FAM19A2	0.203	0.298	0.095	0.000	0.000
FAM19A5	1.771	1.240	-0.531	0.000	0.000
FAM200A	1.887	2.770	0.883	0.000	0.000
FAM200B	3.750	4.108	0.358	0.000	0.000
FAM204A	2.518	2.999	0.481	0.000	0.000
FAM20A	5.124	5.330	0.206	0.000	0.000
FAM20B	3.559	4.688	1.129	0.000	0.000
FAM20C	5.227	5.404	0.177	0.000	0.000
FAM24B	0.547	1.501	0.954	0.000	0.000
FAM32A	6.018	6.530	0.512	0.000	0.000
FAM3A	4.550	5.198	0.647	0.000	0.000
FAM3B	1.762	2.523	0.761	0.000	0.000
FAM3C	4.643	4.929	0.286	0.000	0.000
FAM3D	0.316	0.290	-0.026	0.481	0.497
FAM43A	2.538	3.041	0.503	0.000	0.000
FAM43B	0.670	0.505	-0.165	0.000	0.000
FAM45A	2.903	3.697	0.794	0.000	0.000
FAM47E-STBI	4.202	4.245	0.043	0.370	0.386
FAM49A	2.235	2.273	0.038	0.477	0.493
FAM49B	2.860	4.099	1.239	0.000	0.000
FAM50A	5.182	6.411	1.229	0.000	0.000
FAM50B	3.776	3.907	0.130	0.057	0.062
FAM53A	0.796	1.409	0.614	0.000	0.000
FAM53B	2.209	2.894	0.684	0.000	0.000
FAM53C	2.763	3.517	0.754	0.000	0.000
FAM57A	1.720	2.287	0.567	0.000	0.000
FAM57B	0.038	0.286	0.248	0.000	0.000
FAM71E1	2.008	2.342	0.335	0.000	0.000
FAM71F2	0.366	0.589	0.224	0.000	0.000
FAM72A	0.388	0.928	0.541	0.000	0.000
FAM72B	0.327	0.907	0.581	0.000	0.000
FAM72D	0.110	1.116	1.006	0.000	0.000
FAM76A	2.312	2.698	0.386	0.000	0.000

FAM76B	3.040	3.252	0.211	0.000	0.000
FAM78A	1.618	1.766	0.148	0.001	0.001
FAM78B	0.548	1.127	0.579	0.000	0.000
FAM81A	0.203	0.701	0.498	0.000	0.000
FAM83D	1.555	3.759	2.204	0.000	0.000
FAM83E	0.420	0.258	-0.162	0.000	0.000
FAM83F	0.991	0.250	-0.741	0.000	0.000
FAM83G	2.909	3.257	0.348	0.000	0.000
FAM83H	3.667	5.199	1.532	0.000	0.000
FAM84A	0.879	0.840	-0.039	0.376	0.391
FAM84B	3.393	4.146	0.752	0.000	0.000
FAM86B1	0.430	0.419	-0.010	0.597	0.611
FAM89A	2.907	3.208	0.302	0.000	0.000
FAM89B	4.266	4.885	0.619	0.000	0.000
FAM8A1	5.635	5.899	0.264	0.000	0.000
FAM90A1	0.141	0.341	0.201	0.000	0.000
FAM91A1	3.781	4.693	0.912	0.000	0.000
FAM98A	3.797	4.398	0.602	0.000	0.000
FAM98B	2.137	2.672	0.535	0.000	0.000
FAM98C	2.908	3.584	0.676	0.000	0.000
FAM9B	1.082	0.551	-0.531	0.000	0.000
FAN1	2.891	3.510	0.619	0.000	0.000
FANCA	2.078	2.915	0.837	0.000	0.000
FANCB	0.209	0.698	0.489	0.000	0.000
FANCC	3.119	2.636	-0.483	0.000	0.000
FANCD2	0.848	2.210	1.362	0.000	0.000
FANCE	1.131	2.168	1.037	0.000	0.000
FANCF	2.359	3.264	0.905	0.000	0.000
FANCG	2.177	3.463	1.286	0.000	0.000
FANCI	1.570	2.987	1.417	0.000	0.000
FANCL	3.913	4.345	0.433	0.000	0.000
FANCM	0.891	1.459	0.568	0.000	0.000
FANK1	0.290	0.352	0.061	0.020	0.022
FAP	0.513	1.394	0.880	0.000	0.000
FAR1	1.988	2.130	0.142	0.006	0.006
FAR2	1.030	1.307	0.276	0.000	0.000
FARP1	3.113	3.500	0.387	0.000	0.000
FARP2	2.468	2.313	-0.155	0.000	0.000
FARS2	3.438	4.092	0.654	0.000	0.000
FARSA	4.959	5.693	0.734	0.000	0.000
FARSB	3.959	4.961	1.002	0.000	0.000
FAS	3.824	3.383	-0.441	0.000	0.000
FASLG	1.120	0.974	-0.146	0.001	0.001
FASN	6.198	7.551	1.353	0.000	0.000
FASTK	5.032	6.087	1.055	0.000	0.000
FASTKD1	3.411	3.954	0.543	0.000	0.000
FASTKD2	3.527	3.908	0.381	0.000	0.000
FASTKD3	2.586	3.340	0.754	0.000	0.000
FASTKD5	3.407	4.014	0.607	0.000	0.000
FAT1	3.400	4.615	1.215	0.000	0.000
FAT4	1.367	1.095	-0.272	0.000	0.000
FATE1	0.184	0.758	0.574	0.000	0.000

FAU	8.364	8.829	0.464	0.000	0.000
FBF1	1.099	1.979	0.881	0.000	0.000
FBL	5.827	6.991	1.164	0.000	0.000
FBLIM1	2.651	3.741	1.090	0.000	0.000
FBLL1	0.889	1.403	0.514	0.000	0.000
FBLN1	2.649	3.254	0.604	0.000	0.000
FBLN2	2.743	2.698	-0.045	0.599	0.613
FBLN5	4.409	3.372	-1.038	0.000	0.000
FBLN7	1.854	2.655	0.802	0.000	0.000
FBN1	1.868	2.532	0.663	0.000	0.000
FBN3	0.108	0.647	0.539	0.000	0.000
FBP1	10.002	7.696	-2.306	0.000	0.000
FBP2	0.403	0.487	0.085	0.000	0.000
FBRS	3.734	4.359	0.625	0.000	0.000
FBRSL1	3.519	4.093	0.573	0.000	0.000
FBXL12	3.502	3.982	0.480	0.000	0.000
FBXL13	0.171	0.326	0.155	0.000	0.000
FBXL14	2.328	2.679	0.351	0.000	0.000
FBXL15	3.543	4.025	0.482	0.000	0.000
FBXL16	0.500	0.981	0.481	0.000	0.000
FBXL17	2.624	3.052	0.428	0.000	0.000
FBXL18	0.928	2.114	1.186	0.000	0.000
FBXL19	1.883	2.716	0.833	0.000	0.000
FBXL2	0.434	0.634	0.200	0.000	0.000
FBXL20	1.714	2.310	0.596	0.000	0.000
FBXL22	0.224	0.459	0.236	0.000	0.000
FBXL3	3.730	3.737	0.007	0.856	0.862
FBXL4	2.592	2.928	0.336	0.000	0.000
FBXL5	5.200	5.604	0.403	0.000	0.000
FBXL6	3.581	4.601	1.020	0.000	0.000
FBXL7	1.457	1.995	0.539	0.000	0.000
FBXL8	1.925	2.444	0.519	0.000	0.000
FBXO10	1.727	2.271	0.543	0.000	0.000
FBXO15	0.478	0.402	-0.076	0.000	0.000
FBXO16	1.033	1.108	0.075	0.028	0.031
FBXO17	4.467	4.702	0.235	0.000	0.000
FBXO2	4.389	4.605	0.216	0.017	0.019
FBXO21	3.747	3.574	-0.173	0.000	0.000
FBXO22	2.896	3.498	0.602	0.000	0.000
FBXO24	0.703	0.923	0.220	0.000	0.000
FBXO25	3.211	3.488	0.278	0.000	0.000
FBXO27	2.362	3.376	1.014	0.000	0.000
FBXO28	3.589	3.897	0.308	0.000	0.000
FBXO3	3.099	3.338	0.239	0.000	0.000
FBXO30	1.709	2.264	0.555	0.000	0.000
FBXO31	4.346	4.684	0.339	0.000	0.000
FBXO32	1.281	2.441	1.160	0.000	0.000
FBXO33	2.922	3.190	0.268	0.000	0.000
FBXO34	3.097	3.416	0.319	0.000	0.000
FBXO36	0.991	1.342	0.352	0.000	0.000
FBXO38	3.237	3.769	0.532	0.000	0.000
FBXO39	0.373	0.532	0.158	0.000	0.000

FBXO4	2.929	3.556	0.627	0.000	0.000
FBXO41	0.633	0.962	0.329	0.000	0.000
FBXO42	2.478	2.886	0.409	0.000	0.000
FBXO43	0.231	1.269	1.037	0.000	0.000
FBXO44	3.714	4.463	0.748	0.000	0.000
FBXO45	2.137	3.137	1.000	0.000	0.000
FBXO46	2.989	3.805	0.816	0.000	0.000
FBXO5	1.607	2.224	0.617	0.000	0.000
FBXO6	3.996	4.676	0.680	0.000	0.000
FBXO7	5.196	5.573	0.377	0.000	0.000
FBXO8	4.549	4.395	-0.154	0.000	0.000
FBXO9	3.891	4.653	0.762	0.000	0.000
FBXW10	0.334	0.939	0.605	0.000	0.000
FBXW11	3.703	4.424	0.721	0.000	0.000
FBXW2	3.524	4.122	0.598	0.000	0.000
FBXW4	4.019	4.562	0.542	0.000	0.000
FBXW5	6.505	6.867	0.361	0.000	0.000
FBXW7	2.604	2.660	0.056	0.095	0.103
FBXW8	1.987	2.754	0.767	0.000	0.000
FBXW9	2.182	2.939	0.757	0.000	0.000
FCAMR	2.116	1.814	-0.302	0.001	0.001
FCAR	0.463	0.247	-0.217	0.000	0.000
FCER1A	2.032	1.502	-0.529	0.000	0.000
FCER1G	5.995	5.820	-0.175	0.020	0.022
FCER2	0.790	0.483	-0.307	0.000	0.000
FCF1	3.432	3.969	0.536	0.000	0.000
FCGR1A	1.241	1.583	0.342	0.000	0.000
FCGR1B	0.559	0.554	-0.005	0.866	0.872
FCGR2A	2.473	3.123	0.650	0.000	0.000
FCGR2B	3.189	1.582	-1.607	0.000	0.000
FCGR3A	5.369	4.664	-0.706	0.000	0.000
FCGR3B	1.413	0.936	-0.478	0.000	0.000
FCGRT	7.728	7.394	-0.335	0.000	0.000
FCHO1	1.415	1.113	-0.301	0.000	0.000
FCHO2	3.348	3.787	0.439	0.000	0.000
FCHSD1	1.795	2.047	0.252	0.000	0.000
FCHSD2	3.548	3.600	0.052	0.178	0.190
FCN1	1.778	1.402	-0.376	0.000	0.000
FCN2	6.162	1.553	-4.609	871157617	0.000
FCN3	6.887	2.607	-4.280	0.000	0.000
FCRL1	0.547	0.323	-0.224	0.000	0.000
FCRL2	0.482	0.293	-0.189	0.000	0.000
FCRL3	0.715	0.488	-0.226	0.000	0.000
FCRL5	0.698	0.417	-0.281	0.000	0.000
FCRL6	1.419	0.934	-0.485	0.000	0.000
FCRLA	0.645	0.688	0.043	0.381	0.397
FCRLB	0.759	1.001	0.242	0.000	0.000
FDFT1	5.312	5.934	0.622	0.000	0.000
FDX1	5.427	5.324	-0.103	0.003	0.004
FDXACB1	1.183	1.594	0.412	0.000	0.000
FDXR	3.784	4.168	0.384	0.000	0.000
FECH	3.774	4.165	0.391	0.000	0.000

FEM1B	3.088	3.561	0.473	0.000	0.000
FEM1C	3.635	3.839	0.204	0.000	0.000
FEN1	2.965	4.774	1.809	0.000	0.000
FER	0.928	1.308	0.381	0.000	0.000
FERMT1	0.587	1.445	0.858	0.000	0.000
FERMT2	4.958	4.563	-0.395	0.000	0.000
FERMT3	3.667	3.697	0.030	0.622	0.636
FES	3.959	3.751	-0.208	0.002	0.003
FETUB	7.208	5.481	-1.727	0.000	0.000
FEZ1	2.572	1.690	-0.883	0.000	0.000
FEZ2	3.068	3.520	0.453	0.000	0.000
FFAR2	0.884	0.652	-0.232	0.000	0.000
FFAR3	0.179	0.238	0.060	0.010	0.011
FGA	13.135	11.911	-1.224	0.000	0.000
FGB	13.342	11.857	-1.485	0.000	0.000
FGD1	1.271	2.287	1.017	0.000	0.000
FGD2	2.023	1.638	-0.385	0.000	0.000
FGD3	1.985	1.841	-0.144	0.006	0.007
FGD4	3.004	2.743	-0.262	0.000	0.000
FGD5	1.814	2.154	0.340	0.000	0.000
FGD6	1.691	2.314	0.624	0.000	0.000
FGF1	0.640	0.732	0.092	0.023	0.025
FGF11	0.282	0.397	0.115	0.000	0.000
FGF12	0.393	1.097	0.704	0.000	0.000
FGF13	0.431	1.005	0.574	0.000	0.000
FGF14	0.509	0.754	0.245	0.000	0.000
FGF17	0.246	0.489	0.243	0.000	0.000
FGF18	0.289	0.461	0.172	0.000	0.000
FGF19	0.633	1.154	0.521	0.000	0.000
FGF2	1.584	1.684	0.100	0.080	0.087
FGF21	3.610	4.710	1.100	0.000	0.000
FGF22	0.141	0.228	0.086	0.000	0.000
FGF7	0.679	0.660	-0.019	0.677	0.690
FGFBP2	1.063	0.855	-0.208	0.000	0.000
FGFBP3	0.738	1.106	0.368	0.000	0.000
FGFR1	2.207	1.858	-0.349	0.000	0.000
FGFR1OP	2.211	2.372	0.161	0.000	0.000
FGFR1OP2	3.510	3.579	0.069	0.083	0.090
FGFR2	4.371	3.490	-0.881	0.000	0.000
FGFR3	4.900	5.101	0.200	0.005	0.006
FGFR4	5.369	6.428	1.059	0.000	0.000
FGFRL1	4.664	5.014	0.351	0.000	0.000
FGG	12.667	11.451	-1.216	0.000	0.000
FGGY	4.372	4.842	0.470	0.000	0.000
FGL1	10.104	9.275	-0.829	0.000	0.000
FGL2	4.256	3.585	-0.671	0.000	0.000
FGR	2.816	2.436	-0.380	0.000	0.000
FH	7.429	7.832	0.403	0.000	0.000
FHAD1	0.085	0.416	0.330	0.000	0.000
FHDC1	0.363	0.701	0.338	0.000	0.000
FHIT	1.793	2.585	0.792	0.000	0.000
FHL1	3.870	3.241	-0.629	0.000	0.000

FHL2	2.767	2.305	-0.462	0.000	0.000
FHL3	2.881	3.696	0.815	0.000	0.000
FHL5	0.373	0.523	0.151	0.000	0.000
FHOD1	2.357	2.679	0.322	0.000	0.000
FHOD3	0.725	0.910	0.184	0.001	0.001
FIBIN	1.587	1.640	0.053	0.461	0.477
FIBP	4.100	5.129	1.029	0.000	0.000
FICD	1.628	2.346	0.718	0.000	0.000
FIG4	2.595	3.202	0.606	0.000	0.000
FIGN	1.226	1.150	-0.076	0.073	0.079
FIGNL1	1.524	2.666	1.142	0.000	0.000
FILIP1	1.164	1.229	0.065	0.107	0.115
FILIP1L	3.209	2.796	-0.413	0.000	0.000
FIP1L1	3.067	3.688	0.621	0.000	0.000
FIS1	7.116	7.679	0.563	0.000	0.000
FITM1	3.197	1.833	-1.364	0.000	0.000
FITM2	1.855	2.574	0.719	0.000	0.000
FIZ1	2.087	2.754	0.667	0.000	0.000
FJX1	1.749	2.015	0.265	0.000	0.000
FKBP10	2.884	3.587	0.703	0.000	0.000
FKBP11	3.760	4.853	1.093	0.000	0.000
FKBP14	1.563	2.216	0.653	0.000	0.000
FKBP15	2.896	3.357	0.461	0.000	0.000
FKBP1A	6.009	7.052	1.043	0.000	0.000
FKBP1B	2.201	2.849	0.647	0.000	0.000
FKBP2	7.124	7.394	0.270	0.000	0.000
FKBP3	5.439	6.113	0.674	0.000	0.000
FKBP4	5.447	6.093	0.646	0.000	0.000
FKBP5	4.677	5.052	0.375	0.000	0.000
FKBP7	2.701	2.858	0.157	0.000	0.000
FKBP8	6.890	7.680	0.790	0.000	0.000
FKBPL	2.637	3.655	1.018	0.000	0.000
FKRP	2.641	2.886	0.245	0.000	0.000
FKTN	1.825	2.464	0.639	0.000	0.000
FLAD1	3.919	5.341	1.422	0.000	0.000
FLCN	2.970	3.529	0.559	0.000	0.000
FLI1	2.315	2.335	0.019	0.669	0.682
FLII	5.077	5.611	0.534	0.000	0.000
FLNA	5.067	5.598	0.531	0.000	0.000
FLNB	4.979	5.631	0.653	0.000	0.000
FLNC	0.738	1.494	0.756	0.000	0.000
FLOT1	6.133	7.052	0.919	0.000	0.000
FLOT2	5.468	6.227	0.759	0.000	0.000
FLRT1	0.306	0.523	0.217	0.000	0.000
FLRT2	0.845	0.592	-0.253	0.000	0.000
FLRT3	2.824	2.687	-0.136	0.049	0.053
FLT1	2.084	2.707	0.624	0.000	0.000
FLT3	0.693	0.468	-0.224	0.000	0.000
FLT3LG	1.762	1.957	0.196	0.000	0.000
FLT4	2.353	2.411	0.057	0.205	0.217
FLVCR1	1.536	3.136	1.600	0.000	0.000
FLVCR2	3.337	3.393	0.056	0.254	0.268

FLYWCH1	2.660	3.336	0.676	0.000	0.000
FLYWCH2	3.447	4.428	0.981	0.000	0.000
FMN1	0.295	0.488	0.193	0.000	0.000
FMNL1	2.596	2.593	-0.002	0.968	0.970
FMNL2	2.538	2.869	0.331	0.000	0.000
FMNL3	1.688	2.403	0.715	0.000	0.000
FMO1	0.692	1.485	0.793	0.000	0.000
FMO2	1.808	1.155	-0.653	0.000	0.000
FMO3	8.559	7.473	-1.086	0.000	0.000
FMO4	4.896	4.572	-0.324	0.000	0.000
FMO5	6.779	6.619	-0.160	0.033	0.036
FMOD	3.169	2.730	-0.439	0.000	0.000
FMR1	3.925	4.627	0.702	0.000	0.000
FN1	9.325	9.702	0.377	0.000	0.000
FN3K	5.138	5.592	0.454	0.000	0.000
FN3KRP	4.380	5.295	0.914	0.000	0.000
FNBP1	3.725	4.107	0.382	0.000	0.000
FNBP1L	3.109	4.100	0.992	0.000	0.000
FNBP4	3.836	4.350	0.514	0.000	0.000
FNDC1	0.790	1.076	0.285	0.000	0.000
FNDC3A	4.908	4.773	-0.135	0.003	0.004
FNDC3B	3.704	4.412	0.708	0.000	0.000
FNDC4	6.251	5.283	-0.968	0.000	0.000
FNDC5	4.302	3.144	-1.158	0.000	0.000
FNIP1	2.951	3.554	0.602	0.000	0.000
FNIP2	3.511	3.178	-0.333	0.000	0.000
FNTA	3.613	4.087	0.474	0.000	0.000
FNTB	2.405	3.125	0.721	0.000	0.000
FOLH1	3.872	2.992	-0.880	0.000	0.000
FOLR1	0.514	0.877	0.363	0.000	0.000
FOLR2	4.735	4.384	-0.351	0.000	0.000
FOPNL	4.437	4.970	0.533	0.000	0.000
FOS	7.800	5.214	-2.586	0.000	0.000
FOSB	4.939	2.341	-2.599	0.000	0.000
FOSL1	1.780	1.290	-0.490	0.000	0.000
FOSL2	3.810	3.963	0.153	0.014	0.015
FOXA1	4.179	4.628	0.449	0.000	0.000
FOXA2	5.029	5.398	0.369	0.000	0.000
FOXA3	5.699	5.412	-0.287	0.000	0.000
FOXC1	1.252	1.633	0.381	0.000	0.000
FOXC2	0.272	0.479	0.207	0.000	0.000
FOXD2	0.523	1.261	0.737	0.000	0.000
FOXD4	0.172	0.447	0.274	0.000	0.000
FOXF1	1.769	1.214	-0.555	0.000	0.000
FOXF2	0.245	0.553	0.309	0.000	0.000
FOXH1	1.077	1.440	0.363	0.000	0.000
FOXJ1	0.382	0.850	0.469	0.000	0.000
FOXJ2	2.371	2.814	0.443	0.000	0.000
FOXJ3	3.426	3.753	0.327	0.000	0.000
FOXK1	1.703	2.565	0.862	0.000	0.000
FOXK2	2.774	3.714	0.940	0.000	0.000
FOXL1	0.312	0.563	0.251	0.000	0.000

FOXM1	1.365	3.522	2.157	0.000	0.000
FOXN2	3.214	3.910	0.697	0.000	0.000
FOXN3	3.745	4.137	0.391	0.000	0.000
FOXN4	0.465	1.339	0.874	0.000	0.000
FOXO1	4.219	3.591	-0.628	0.000	0.000
FOXO3	3.474	3.815	0.341	0.000	0.000
FOXO4	2.865	3.500	0.634	0.000	0.000
FOXP1	2.938	3.655	0.717	0.000	0.000
FOXP2	0.950	0.614	-0.337	0.000	0.000
FOXP3	1.893	2.064	0.171	0.021	0.023
FOXP4	3.867	4.751	0.884	0.000	0.000
FOXQ1	1.619	2.522	0.902	0.000	0.000
FOXRED1	4.537	4.760	0.223	0.000	0.000
FOXRED2	2.739	3.926	1.187	0.000	0.000
FOXS1	0.799	1.978	1.180	0.000	0.000
FPGS	5.294	5.352	0.058	0.079	0.085
FPGT	2.755	3.439	0.684	0.000	0.000
FPGT-TNNI3K	0.297	0.362	0.065	0.000	0.000
FPR1	2.426	1.633	-0.793	0.000	0.000
FPR2	0.744	0.344	-0.401	0.000	0.000
FPR3	3.321	3.100	-0.220	0.001	0.002
FRA10AC1	2.898	3.401	0.503	0.000	0.000
FRAS1	0.576	0.894	0.318	0.000	0.000
FRAT1	3.182	3.780	0.598	0.000	0.000
FRAT2	3.773	4.509	0.736	0.000	0.000
FREM1	0.536	0.384	-0.152	0.000	0.000
FREM2	1.473	0.442	-1.030	0.000	0.000
FRG1	4.596	4.889	0.293	0.000	0.000
FRMD1	0.187	0.299	0.112	0.000	0.000
FRMD3	0.672	1.016	0.345	0.000	0.000
FRMD4A	2.724	2.742	0.018	0.721	0.732
FRMD4B	3.504	3.004	-0.500	0.000	0.000
FRMD6	2.272	2.092	-0.180	0.001	0.001
FRMD8	3.299	4.217	0.918	0.000	0.000
FRMPD1	0.692	1.067	0.374	0.000	0.000
FRRS1	3.119	3.577	0.458	0.000	0.000
FRS2	2.689	3.275	0.586	0.000	0.000
FRS3	1.739	2.510	0.771	0.000	0.000
FRY	2.253	2.602	0.349	0.000	0.000
FRYL	2.212	2.609	0.397	0.000	0.000
FRZB	2.086	2.635	0.549	0.000	0.000
FSCN1	3.626	4.099	0.473	0.000	0.000
FSCN2	0.183	0.294	0.111	0.000	0.000
FSCN3	0.109	0.109	0.000	0.969	0.971
FSD1	0.598	0.419	-0.178	0.000	0.000
FSD1L	0.360	0.893	0.533	0.000	0.000
FSIP1	0.235	0.446	0.212	0.000	0.000
FSIP2	0.106	0.258	0.152	0.000	0.000
FST	5.870	5.941	0.071	0.431	0.448
FSTL1	3.938	3.956	0.017	0.802	0.810
FSTL3	3.838	3.991	0.153	0.083	0.090
FSTL4	0.127	0.664	0.537	0.000	0.000

FTCD	8.905	7.271	-1.634	0.000	0.000
FTH1	9.917	10.862	0.945	0.000	0.000
FTL	13.527	14.427	0.901	0.000	0.000
FTO	2.200	2.604	0.404	0.000	0.000
FTSJ1	3.528	4.354	0.825	0.000	0.000
FTSJ3	3.902	4.900	0.998	0.000	0.000
FUBP1	3.886	4.661	0.774	0.000	0.000
FUBP3	4.418	4.841	0.423	0.000	0.000
FUCA1	6.371	6.455	0.084	0.016	0.018
FUCA2	5.363	6.255	0.892	0.000	0.000
FUK	2.743	3.246	0.503	0.000	0.000
FUNDC1	3.246	4.042	0.796	0.000	0.000
FUNDC2	3.626	4.271	0.645	0.000	0.000
FURIN	7.253	7.317	0.064	0.290	0.305
FUS	5.127	5.914	0.788	0.000	0.000
FUT1	0.891	1.415	0.524	0.000	0.000
FUT10	1.242	1.408	0.166	0.000	0.000
FUT11	2.014	2.311	0.297	0.000	0.000
FUT2	0.280	1.263	0.983	0.000	0.000
FUT3	0.890	0.701	-0.189	0.000	0.001
FUT4	1.498	1.691	0.193	0.001	0.001
FUT5	0.221	0.242	0.021	0.148	0.158
FUT6	2.665	2.176	-0.489	0.000	0.000
FUT7	0.371	0.378	0.007	0.783	0.792
FUT8	1.434	1.629	0.195	0.000	0.000
FUZ	2.981	3.060	0.079	0.128	0.137
FXN	3.789	3.274	-0.515	0.000	0.000
FXR1	4.059	4.861	0.803	0.000	0.000
FXR2	3.984	4.306	0.322	0.000	0.000
FXYD1	6.740	4.541	-2.199	0.000	0.000
FXYD2	2.911	2.167	-0.744	0.000	0.000
FXYD3	0.567	1.341	0.774	0.000	0.000
FXYD5	4.388	4.624	0.236	0.000	0.000
FXYD6	3.517	2.938	-0.578	0.000	0.000
FXYD7	0.525	0.342	-0.183	0.000	0.000
FYCO1	2.943	3.543	0.600	0.000	0.000
FYN	4.113	3.448	-0.665	0.000	0.000
FYTTD1	3.717	4.040	0.323	0.000	0.000
FZD1	1.973	1.844	-0.129	0.023	0.025
FZD2	0.708	1.072	0.365	0.000	0.000
FZD3	0.553	0.786	0.233	0.000	0.000
FZD4	3.525	3.746	0.221	0.000	0.000
FZD5	4.032	4.545	0.513	0.000	0.000
FZD6	2.013	3.008	0.995	0.000	0.000
FZD7	1.311	1.505	0.195	0.001	0.001
FZD8	1.867	1.553	-0.313	0.000	0.000
FZD9	0.267	0.496	0.229	0.000	0.000
FZR1	3.818	4.314	0.496	0.000	0.000
G0S2	7.643	6.858	-0.785	0.000	0.000
G2E3	1.442	2.060	0.618	0.000	0.000
G3BP1	4.133	4.830	0.697	0.000	0.000
G3BP2	4.507	4.726	0.219	0.000	0.000

G6PC	8.206	7.297	-0.908	0.000	0.000
G6PC3	3.073	4.013	0.941	0.000	0.000
G6PD	2.557	4.365	1.808	0.000	0.000
GAA	5.664	6.295	0.631	0.000	0.000
GAB1	2.148	2.289	0.141	0.000	0.000
GAB2	2.101	2.963	0.863	0.000	0.000
GAB3	1.517	1.461	-0.057	0.189	0.200
GABARAPL1	6.594	5.710	-0.884	0.000	0.000
GABARAPL2	5.499	5.869	0.369	0.000	0.000
GABBR1	1.862	1.658	-0.203	0.001	0.001
GABBR2	0.280	0.624	0.344	0.000	0.000
GABPA	3.426	3.775	0.348	0.000	0.000
GABPB1	2.134	2.681	0.547	0.000	0.000
GABPB2	1.402	2.004	0.602	0.000	0.000
GABRB3	1.070	0.557	-0.513	0.000	0.000
GABRD	0.478	2.129	1.650	0.000	0.000
GABRE	1.952	3.187	1.235	0.000	0.000
GABRP	1.459	0.529	-0.930	0.000	0.000
GABRR2	0.340	0.405	0.065	0.003	0.003
GADD45A	6.927	6.209	-0.717	0.000	0.000
GADD45B	8.044	6.675	-1.369	0.000	0.000
GADD45G	7.372	6.214	-1.157	0.000	0.000
GADD45GIP1	5.617	6.300	0.683	0.000	0.000
GAK	3.999	4.526	0.527	0.000	0.000
GAL3ST1	1.795	2.696	0.901	0.000	0.000
GAL3ST2	0.397	0.604	0.207	0.000	0.000
GAL3ST4	0.896	1.411	0.515	0.000	0.000
GALC	2.560	2.676	0.116	0.036	0.039
GALE	5.211	5.725	0.514	0.000	0.000
GALK1	5.853	6.524	0.671	0.000	0.000
GALK2	2.929	3.580	0.651	0.000	0.000
GALM	5.726	5.852	0.126	0.001	0.001
GALNS	2.647	2.715	0.068	0.072	0.079
GALNT1	4.654	5.432	0.777	0.000	0.000
GALNT10	1.780	2.772	0.992	0.000	0.000
GALNT11	3.070	3.850	0.780	0.000	0.000
GALNT12	0.665	0.929	0.264	0.000	0.000
GALNT14	0.748	0.535	-0.213	0.000	0.000
GALNT2	5.234	5.700	0.467	0.000	0.000
GALNT3	1.520	1.258	-0.262	0.000	0.000
GALNT4	1.370	1.507	0.137	0.000	0.000
GALNT6	0.839	0.983	0.144	0.000	0.000
GALNT7	1.093	1.280	0.187	0.000	0.000
GALP	0.438	0.479	0.041	0.232	0.245
GALR2	0.134	0.317	0.182	0.000	0.000
GALR3	0.362	0.655	0.293	0.000	0.000
GAMT	8.874	8.295	-0.579	0.000	0.000
GAN	0.597	0.714	0.117	0.000	0.000
GANAB	6.908	7.623	0.715	0.000	0.000
GANC	2.251	2.467	0.216	0.000	0.000
GAP43	0.122	0.383	0.261	0.000	0.000
GAPT	0.780	0.625	-0.154	0.000	0.000

GAPVD1	2.591	2.944	0.353	0.000	0.000
GAR1	3.501	4.090	0.590	0.000	0.000
GARNL3	1.220	1.233	0.012	0.748	0.758
GARS	4.872	6.040	1.168	0.000	0.000
GART	3.625	4.298	0.673	0.000	0.000
GAS1	1.847	1.134	-0.713	0.000	0.000
GAS2	3.910	3.864	-0.046	0.403	0.419
GAS2L1	3.009	3.780	0.771	0.000	0.000
GAS2L3	0.880	1.699	0.819	0.000	0.000
GAS6	4.838	4.462	-0.376	0.000	0.000
GAS7	1.131	1.394	0.263	0.000	0.000
GAS8	2.586	3.278	0.691	0.000	0.000
GATA1	0.119	0.155	0.036	0.011	0.012
GATA2	1.106	1.317	0.211	0.000	0.000
GATA3	1.399	1.044	-0.356	0.000	0.000
GATA4	4.149	4.203	0.054	0.272	0.286
GATA5	0.801	0.483	-0.318	0.000	0.000
GATA6	3.665	3.390	-0.274	0.000	0.000
GATAD1	3.850	4.647	0.797	0.000	0.000
GATAD2A	3.828	4.356	0.527	0.000	0.000
GATAD2B	2.833	3.566	0.733	0.000	0.000
GATC	3.663	4.338	0.675	0.000	0.000
GATM	9.050	8.477	-0.573	0.000	0.000
GBA	4.182	5.915	1.733	0.000	0.000
GBA2	4.597	5.355	0.758	0.000	0.000
GBE1	5.017	5.014	-0.002	0.961	0.963
GBF1	3.977	4.676	0.700	0.000	0.000
GBGT1	1.075	1.381	0.307	0.000	0.000
GBP1	5.628	5.004	-0.624	0.000	0.000
GBP2	4.180	5.398	1.218	0.000	0.000
GBP3	3.741	3.882	0.141	0.040	0.045
GBP4	3.687	3.699	0.012	0.846	0.852
GBP5	2.410	2.218	-0.192	0.015	0.017
GBP6	0.201	0.217	0.016	0.449	0.465
GBP7	5.565	4.218	-1.348	0.000	0.000
GC	11.794	11.006	-0.789	0.000	0.000
GCA	3.136	3.399	0.264	0.000	0.000
GCAT	5.546	5.365	-0.181	0.002	0.003
GCC1	3.040	3.642	0.601	0.000	0.000
GCC2	2.860	3.067	0.208	0.000	0.000
GCDH	6.040	5.093	-0.947	0.000	0.000
GCGR	6.148	3.515	-2.633	0.000	0.000
GCH1	5.990	4.845	-1.145	0.000	0.000
GCHFR	6.173	5.733	-0.440	0.000	0.000
GCK	1.272	1.033	-0.239	0.015	0.017
GCKR	6.565	5.340	-1.225	0.000	0.000
GCLC	5.275	5.281	0.006	0.895	0.899
GCLM	4.434	4.723	0.288	0.000	0.000
GCNT1	1.068	1.124	0.056	0.226	0.239
GCNT2	2.389	2.501	0.112	0.012	0.013
GCNT3	0.376	1.399	1.024	0.000	0.000
GCNT4	1.105	1.427	0.321	0.000	0.000

GCSH	5.336	4.980	-0.356	0.000	0.000
GDA	4.756	3.887	-0.869	0.000	0.000
GDAP1	1.324	2.333	1.009	0.000	0.000
GDAP2	1.597	2.038	0.441	0.000	0.000
GDE1	4.124	4.649	0.525	0.000	0.000
GDF11	0.888	1.263	0.375	0.000	0.000
GDF15	5.698	5.981	0.283	0.003	0.003
GDF6	0.484	0.286	-0.198	0.000	0.000
GDF7	1.246	1.061	-0.185	0.000	0.000
GDF9	0.446	0.726	0.280	0.000	0.000
GDI1	4.861	5.749	0.888	0.000	0.000
GDI2	6.725	7.172	0.447	0.000	0.000
GDNF	0.839	0.876	0.036	0.562	0.577
GDPD1	0.827	1.771	0.944	0.000	0.000
GDPD3	1.469	1.979	0.510	0.000	0.000
GDPD4	0.809	0.677	-0.132	0.002	0.002
GDPD5	1.764	2.095	0.331	0.000	0.000
GEM	3.564	2.923	-0.641	0.000	0.000
GEMIN4	2.507	2.705	0.198	0.000	0.000
GEMIN5	2.565	3.187	0.622	0.000	0.000
GEMIN6	2.608	3.419	0.811	0.000	0.000
GEMIN7	3.608	4.586	0.978	0.000	0.000
GEMIN8	3.343	3.917	0.574	0.000	0.000
GEN1	1.997	2.293	0.297	0.000	0.000
GET4	3.355	3.713	0.358	0.000	0.000
GFAP	0.264	0.328	0.064	0.036	0.039
GFER	4.388	4.821	0.433	0.000	0.000
GFI1	1.124	1.249	0.124	0.012	0.013
GFM1	3.986	4.478	0.492	0.000	0.000
GFM2	4.364	4.912	0.548	0.000	0.000
GFOD1	1.702	1.631	-0.071	0.034	0.038
GFOD2	3.485	3.280	-0.205	0.000	0.000
GFPT1	3.758	4.537	0.779	0.000	0.000
GFPT2	1.223	1.079	-0.144	0.029	0.032
GFRA1	3.437	2.544	-0.893	0.000	0.000
GFRA2	1.178	0.950	-0.228	0.000	0.000
GFRA3	0.211	0.341	0.130	0.005	0.006
GGA1	3.578	4.251	0.673	0.000	0.000
GGA2	3.755	4.487	0.732	0.000	0.000
GGA3	3.021	3.853	0.832	0.000	0.000
GGCT	4.824	5.813	0.988	0.000	0.000
GGCX	5.732	6.016	0.284	0.000	0.000
GGH	6.159	7.284	1.124	0.000	0.000
GGN	0.114	0.328	0.214	0.000	0.000
GGPS1	3.456	4.395	0.939	0.000	0.000
GGT1	4.793	4.250	-0.543	0.000	0.000
GGT5	4.584	2.965	-1.618	0.000	0.000
GGT6	0.921	0.495	-0.426	0.000	0.000
GGT7	3.620	4.062	0.442	0.000	0.000
GHDC	3.784	4.499	0.715	0.000	0.000
GHITM	7.742	7.770	0.029	0.391	0.407
GHR	5.868	3.895	-1.973	0.000	0.000

GHRHR	0.153	0.875	0.721	0.000	0.000
GHRL	0.660	0.704	0.044	0.101	0.109
GIGYF1	3.837	4.685	0.848	0.000	0.000
GIGYF2	2.894	3.430	0.537	0.000	0.000
GIMAP1	2.103	1.986	-0.117	0.007	0.007
GIMAP2	3.312	3.520	0.207	0.000	0.000
GIMAP4	4.699	4.448	-0.251	0.000	0.000
GIMAP5	2.805	2.640	-0.165	0.000	0.000
GIMAP6	3.084	2.978	-0.105	0.054	0.059
GIMAP7	4.490	4.291	-0.198	0.001	0.001
GIMAP8	3.076	2.838	-0.238	0.000	0.000
GIN1	1.968	2.248	0.280	0.000	0.000
GINS1	0.872	2.739	1.867	0.000	0.000
GINS2	1.338	2.633	1.295	0.000	0.000
GINS3	0.829	1.741	0.911	0.000	0.000
GINS4	0.630	1.753	1.123	0.000	0.000
GIPC1	4.418	5.537	1.118	0.000	0.000
GIPC2	3.544	2.926	-0.618	0.000	0.000
GIPC3	1.403	1.691	0.288	0.000	0.000
GIPR	0.504	0.620	0.116	0.008	0.009
GIT1	2.903	3.939	1.035	0.000	0.000
GJA1	2.930	3.785	0.854	0.000	0.000
GJA4	3.724	3.964	0.239	0.000	0.000
GJB1	7.912	8.215	0.303	0.000	0.000
GJB2	4.984	4.492	-0.492	0.000	0.000
GJB3	0.937	0.559	-0.379	0.000	0.000
GJB5	0.244	0.379	0.136	0.000	0.000
GJC1	0.464	1.476	1.012	0.000	0.000
GJC2	0.755	0.739	-0.016	0.649	0.662
GJC3	1.894	1.267	-0.627	0.000	0.000
GJD3	1.669	2.122	0.453	0.000	0.000
GK	4.926	4.377	-0.550	0.000	0.000
GK5	1.406	2.138	0.733	0.000	0.000
GKAP1	2.822	3.239	0.417	0.000	0.000
GLA	3.623	4.946	1.323	0.000	0.000
GLB1	4.614	5.506	0.893	0.000	0.000
GLB1L	1.989	2.764	0.775	0.000	0.000
GLB1L2	0.372	0.434	0.062	0.089	0.096
GLCCI1	1.970	2.339	0.368	0.000	0.000
GLCE	3.345	3.871	0.526	0.000	0.000
GLDC	5.487	4.907	-0.580	0.000	0.000
GLDN	0.244	1.009	0.765	0.000	0.000
GLE1	3.897	4.505	0.608	0.000	0.000
GLG1	3.811	4.396	0.585	0.000	0.000
GLI1	0.503	0.937	0.434	0.000	0.000
GLI2	0.494	0.476	-0.018	0.604	0.618
GLI3	0.395	0.453	0.058	0.047	0.052
GLI4	2.619	3.870	1.251	0.000	0.000
GLIPR1	2.460	2.748	0.288	0.000	0.000
GLIPR1L2	0.516	0.546	0.030	0.204	0.217
GLIPR2	2.570	2.783	0.213	0.000	0.000
GLIS2	1.970	2.616	0.646	0.000	0.000

GLIS3	1.573	1.535	-0.038	0.490	0.505
GLMN	1.935	2.749	0.814	0.000	0.000
GLO1	6.447	7.067	0.620	0.000	0.000
GLOD4	4.735	4.699	-0.036	0.249	0.263
GLOD5	2.697	1.726	-0.971	0.000	0.000
GLP2R	0.355	0.159	-0.196	0.000	0.000
GLRB	0.939	1.159	0.219	0.001	0.001
GLRX	5.329	4.925	-0.404	0.000	0.000
GLRX2	4.025	4.869	0.844	0.000	0.000
GLRX3	3.850	4.723	0.872	0.000	0.000
GLRX5	5.778	6.117	0.338	0.000	0.000
GLS	2.638	3.587	0.949	0.000	0.000
GLS2	5.741	3.169	-2.572	0.000	0.000
GLT1D1	3.959	3.406	-0.553	0.000	0.000
GLT8D1	5.066	5.561	0.495	0.000	0.000
GLT8D2	1.570	1.336	-0.235	0.000	0.000
GLTP	3.736	4.460	0.725	0.000	0.000
GLTPD2	6.412	5.528	-0.884	0.000	0.000
GLUD1	8.602	8.118	-0.484	0.000	0.000
GLUD2	2.602	3.403	0.801	0.000	0.000
GLUL	7.510	8.831	1.321	0.000	0.000
GLYAT	7.340	4.645	-2.695	0.000	0.000
GLYATL1	7.054	4.907	-2.148	0.000	0.000
GLYATL3	0.396	0.262	-0.134	0.000	0.000
GLYCTK	7.262	6.929	-0.333	0.000	0.000
GLYR1	4.538	5.023	0.485	0.000	0.000
GM2A	3.980	5.221	1.242	0.000	0.000
GMCL1	2.713	3.409	0.695	0.000	0.000
GMDS	3.283	4.087	0.804	0.000	0.000
GMEB1	1.684	2.210	0.526	0.000	0.000
GMEB2	2.650	3.522	0.871	0.000	0.000
GMFB	3.271	4.273	1.001	0.000	0.000
GMFG	4.888	4.636	-0.252	0.000	0.000
GMIP	2.578	2.951	0.373	0.000	0.000
GMNN	3.418	5.556	2.138	0.000	0.000
GMPPA	4.397	5.172	0.775	0.000	0.000
GMPPB	2.334	3.097	0.762	0.000	0.000
GMPR	2.960	2.525	-0.436	0.000	0.000
GMPR2	4.785	5.369	0.584	0.000	0.000
GMPS	2.950	4.117	1.167	0.000	0.000
GNA11	3.383	3.845	0.463	0.000	0.000
GNA12	2.981	4.122	1.142	0.000	0.000
GNA13	4.224	4.830	0.605	0.000	0.000
GNA14	2.530	1.550	-0.980	0.000	0.000
GNA15	1.655	1.803	0.148	0.005	0.005
GNAI1	3.586	4.439	0.854	0.000	0.000
GNAI2	5.942	6.286	0.344	0.000	0.000
GNAI3	2.843	3.389	0.546	0.000	0.000
GNAL	0.585	1.228	0.643	0.000	0.000
GNAO1	2.555	1.285	-1.271	0.000	0.000
GNAQ	3.267	3.681	0.414	0.000	0.000
GNAS	6.757	7.308	0.551	0.000	0.000

GNAT1	0.252	0.526	0.274	0.000	0.000
GNAT2	0.356	0.363	0.008	0.592	0.606
GNAZ	0.762	2.515	1.754	0.000	0.000
GNB1	5.685	6.491	0.806	0.000	0.000
GNB1L	1.008	1.817	0.810	0.000	0.000
GNB2	6.465	7.139	0.674	0.000	0.000
GNB3	0.387	0.532	0.146	0.000	0.000
GNB4	2.027	1.974	-0.053	0.268	0.282
GNB5	2.331	3.299	0.968	0.000	0.000
GNE	5.644	4.857	-0.786	0.000	0.000
GNG11	3.896	3.979	0.083	0.094	0.102
GNG12	4.718	4.791	0.073	0.136	0.146
GNG2	2.352	2.140	-0.212	0.000	0.000
GNG3	0.675	0.890	0.214	0.000	0.000
GNG4	0.181	1.338	1.157	0.000	0.000
GNG5	6.908	7.517	0.609	0.000	0.000
GNG7	2.329	2.341	0.012	0.810	0.818
GNGT2	1.496	1.394	-0.101	0.012	0.013
GNL1	3.840	4.469	0.629	0.000	0.000
GNL2	4.172	4.928	0.756	0.000	0.000
GNL3	5.189	5.999	0.810	0.000	0.000
GNL3L	2.663	3.341	0.678	0.000	0.000
GNLY	2.015	1.855	-0.159	0.004	0.004
GNMT	8.094	5.986	-2.108	0.000	0.000
GNPAT	4.753	6.055	1.302	0.000	0.000
GNPDA1	3.371	4.453	1.082	0.000	0.000
GNPDA2	1.189	1.648	0.459	0.000	0.000
GNPNAT1	5.419	5.163	-0.256	0.000	0.000
GNPTAB	2.479	3.218	0.739	0.000	0.000
GNPTG	5.658	6.143	0.485	0.000	0.000
GNRH1	1.226	1.516	0.290	0.000	0.000
GNRH2	1.017	0.462	-0.555	0.000	0.000
GNS	5.023	6.144	1.121	0.000	0.000
GOLGA1	3.328	3.743	0.415	0.000	0.000
GOLGA2	4.354	4.909	0.555	0.000	0.000
GOLGA3	3.114	4.017	0.903	0.000	0.000
GOLGA4	4.803	5.059	0.256	0.000	0.000
GOLGA5	5.095	5.523	0.427	0.000	0.000
GOLGA6A	0.254	0.227	-0.027	0.141	0.151
GOLGA6B	0.291	0.251	-0.040	0.072	0.078
GOLGA7	5.058	5.448	0.390	0.000	0.000
GOLGA8A	2.187	2.643	0.456	0.000	0.000
GOLGA8B	3.415	4.255	0.839	0.000	0.000
GOLGB1	4.341	4.758	0.417	0.000	0.000
GOLIM4	4.838	5.062	0.224	0.000	0.000
GOLM1	4.163	5.357	1.194	0.000	0.000
GOLPH3	6.531	6.978	0.447	0.000	0.000
GOLPH3L	3.118	4.415	1.297	0.000	0.000
GOLT1A	7.120	7.165	0.046	0.399	0.415
GOLT1B	3.840	4.931	1.091	0.000	0.000
GON4L	2.562	3.338	0.776	0.000	0.000
GOPC	3.663	4.038	0.375	0.000	0.000

GORAB	2.647	3.573	0.926	0.000	0.000
GORASP1	4.005	4.428	0.423	0.000	0.000
GORASP2	4.517	5.466	0.949	0.000	0.000
GOSR1	3.339	3.864	0.525	0.000	0.000
GOSR2	3.192	4.122	0.930	0.000	0.000
GOT1	8.369	7.678	-0.691	0.000	0.000
GOT2	7.930	7.249	-0.680	0.000	0.000
GP1BA	0.972	0.853	-0.119	0.001	0.001
GP1BB	0.389	0.851	0.462	0.000	0.000
GP6	0.311	0.203	-0.109	0.000	0.000
GPAA1	5.687	7.254	1.566	0.000	0.000
GPAM	5.405	5.710	0.305	0.000	0.000
GPANK1	3.472	4.423	0.952	0.000	0.000
GPAT2	0.726	1.076	0.350	0.000	0.000
GPATCH1	2.368	3.119	0.751	0.000	0.000
GPATCH2	1.506	2.315	0.809	0.000	0.000
GPATCH3	3.271	4.091	0.820	0.000	0.000
GPATCH4	3.442	4.711	1.268	0.000	0.000
GPATCH8	2.742	3.334	0.591	0.000	0.000
GPBAR1	1.413	1.160	-0.254	0.000	0.000
GPBP1	4.351	5.148	0.797	0.000	0.000
GPBP1L1	4.941	5.390	0.449	0.000	0.000
GPC1	4.153	3.872	-0.281	0.000	0.000
GPC2	0.169	0.556	0.387	0.000	0.000
GPC3	3.154	7.685	4.531	0.000	0.000
GPC4	1.555	1.620	0.065	0.408	0.424
GPC5	0.540	0.956	0.416	0.000	0.000
GPC6	3.461	3.679	0.218	0.001	0.001
GPCPD1	2.989	3.357	0.368	0.000	0.000
GPD1	6.358	4.653	-1.705	0.000	0.000
GPD1L	1.778	2.500	0.722	0.000	0.000
GPD2	1.909	2.715	0.806	0.000	0.000
GPHA2	0.332	0.268	-0.064	0.030	0.034
GPHN	4.403	3.782	-0.621	0.000	0.000
GPI	5.929	6.772	0.843	0.000	0.000
GPKOW	4.259	5.108	0.849	0.000	0.000
GPLD1	3.989	3.714	-0.275	0.003	0.004
GPM6A	2.246	0.596	-1.649	0.000	0.000
GPM6B	0.500	0.411	-0.089	0.000	0.000
GPN1	3.578	4.394	0.816	0.000	0.000
GPN2	2.859	3.478	0.619	0.000	0.000
GPN3	3.921	4.627	0.706	0.000	0.000
GPNMB	3.720	4.668	0.948	0.000	0.000
GPR107	3.174	4.182	1.009	0.000	0.000
GPR132	1.045	1.003	-0.042	0.326	0.341
GPR135	0.547	0.634	0.087	0.000	0.000
GPR137	3.576	4.460	0.883	0.000	0.000
GPR137B	3.600	3.942	0.342	0.000	0.000
GPR137C	0.581	1.232	0.651	0.000	0.000
GPR141	0.190	0.337	0.148	0.000	0.000
GPR143	1.208	1.568	0.360	0.000	0.000
GPR15	0.359	0.265	-0.094	0.001	0.001

GPR150	0.171	0.375	0.204	0.000	0.000
GPR153	1.944	2.210	0.266	0.000	0.000
GPR155	2.463	2.685	0.222	0.000	0.000
GPR157	1.739	1.963	0.224	0.000	0.000
GPR158	0.166	1.193	1.027	0.000	0.000
GPR160	1.202	1.964	0.762	0.000	0.000
GPR161	0.303	0.620	0.316	0.000	0.000
GPR162	2.274	1.622	-0.652	0.000	0.000
GPR171	1.804	1.379	-0.426	0.000	0.000
GPR173	0.397	0.504	0.107	0.000	0.000
GPR174	1.042	0.880	-0.162	0.001	0.001
GPR176	1.170	1.731	0.561	0.000	0.000
GPR18	0.929	0.752	-0.177	0.000	0.000
GPR180	2.413	2.095	-0.318	0.000	0.000
GPR182	1.642	0.433	-1.209	0.000	0.000
GPR183	3.215	2.928	-0.287	0.000	0.000
GPR19	0.202	0.673	0.471	0.000	0.000
GPR20	0.223	0.381	0.158	0.000	0.000
GPR27	0.248	0.304	0.056	0.133	0.143
GPR3	0.503	0.650	0.147	0.000	0.000
GPR34	2.111	2.450	0.339	0.000	0.000
GPR35	0.623	1.341	0.718	0.000	0.000
GPR37	2.981	3.262	0.282	0.002	0.003
GPR37L1	0.138	0.241	0.102	0.000	0.000
GPR39	1.872	2.482	0.611	0.000	0.000
GPR4	1.925	2.468	0.543	0.000	0.000
GPR55	0.287	0.245	-0.042	0.016	0.018
GPR62	0.286	0.379	0.093	0.000	0.000
GPR65	1.869	1.389	-0.480	0.000	0.000
GPR68	1.080	1.101	0.021	0.627	0.641
GPR75	1.168	1.359	0.191	0.000	0.000
GPR75-ASB3	1.883	2.366	0.483	0.000	0.000
GPR82	0.521	0.386	-0.135	0.000	0.000
GPR83	0.314	0.177	-0.137	0.000	0.000
GPR84	0.387	0.581	0.193	0.000	0.000
GPR85	0.373	0.417	0.044	0.041	0.046
GPR88	2.179	2.832	0.653	0.000	0.000
GPRASP1	1.689	1.374	-0.315	0.000	0.000
GPRASP2	2.495	2.693	0.198	0.000	0.000
GPRC5A	0.484	0.416	-0.068	0.189	0.201
GPRC5B	2.457	2.757	0.300	0.000	0.000
GPRC5C	5.573	5.721	0.147	0.000	0.000
GPRIN1	0.571	1.587	1.016	0.000	0.000
GPRIN2	0.789	0.400	-0.389	0.000	0.000
GPRIN3	1.229	1.227	-0.002	0.965	0.967
GPS1	5.235	6.021	0.787	0.000	0.000
GPS2	3.109	3.625	0.516	0.000	0.000
GPSM1	1.574	2.835	1.261	0.000	0.000
GPSM2	0.854	1.928	1.074	0.000	0.000
GPSM3	4.113	3.903	-0.209	0.000	0.001
GPT	7.048	5.974	-1.074	0.000	0.000
GPT2	6.632	5.720	-0.912	0.000	0.000

GPX1	8.191	8.862	0.671	0.000	0.000
GPX2	6.843	8.000	1.157	0.000	0.000
GPX3	8.565	8.806	0.241	0.001	0.001
GPX4	8.591	8.994	0.403	0.000	0.000
GPX7	2.179	3.076	0.896	0.000	0.000
GPX8	1.305	1.955	0.651	0.000	0.000
GRAMD1A	3.154	4.506	1.352	0.000	0.000
GRAMD1B	1.010	1.244	0.234	0.000	0.000
GRAMD1C	3.486	2.413	-1.073	0.000	0.000
GRAMD4	4.621	4.677	0.055	0.394	0.410
GRAP2	0.929	0.953	0.023	0.572	0.586
GRAPL	0.325	0.208	-0.117	0.000	0.000
GRASP	3.143	2.793	-0.350	0.000	0.000
GRB10	3.896	4.451	0.555	0.000	0.000
GRB14	5.576	5.253	-0.323	0.000	0.000
GRB2	4.991	5.875	0.884	0.000	0.000
GRB7	2.932	3.403	0.471	0.000	0.000
GREB1	1.071	1.973	0.901	0.000	0.000
GREB1L	0.815	1.622	0.807	0.000	0.000
GREM1	0.692	0.960	0.268	0.000	0.000
GREM2	3.854	2.149	-1.705	0.000	0.000
GRHL1	2.276	2.216	-0.060	0.228	0.241
GRHL2	0.783	0.420	-0.363	0.000	0.000
GRHL3	0.320	0.427	0.107	0.000	0.000
GRHPR	7.708	6.880	-0.828	0.000	0.000
GRIA3	1.374	1.060	-0.314	0.000	0.000
GRID1	0.705	0.944	0.239	0.000	0.000
GRIK1	0.238	0.230	-0.008	0.656	0.669
GRIK5	0.212	0.342	0.129	0.000	0.000
GRIN2D	0.365	0.601	0.235	0.000	0.000
GRIN3A	0.262	0.368	0.105	0.000	0.000
GRIN3B	0.418	0.508	0.090	0.000	0.000
GRINA	7.585	8.517	0.932	0.000	0.000
GRIP1	0.221	0.263	0.042	0.059	0.064
GRIP2	0.304	0.348	0.043	0.089	0.097
GRIPAP1	3.914	4.405	0.491	0.000	0.000
GRK4	1.158	1.386	0.228	0.000	0.000
GRK5	2.718	2.765	0.047	0.249	0.263
GRK6	3.007	4.043	1.036	0.000	0.000
GRM2	0.152	0.299	0.147	0.000	0.000
GRM8	0.788	0.375	-0.413	0.000	0.000
GRN	6.627	7.794	1.167	0.000	0.000
GRPEL1	5.086	5.032	-0.054	0.046	0.051
GRPEL2	2.397	3.448	1.051	0.000	0.000
GRPR	0.223	0.834	0.612	0.000	0.000
GRSF1	4.377	4.715	0.338	0.000	0.000
GRTP1	4.843	4.810	-0.033	0.493	0.509
GRWD1	3.318	4.087	0.769	0.000	0.000
GSDMA	0.311	0.426	0.115	0.000	0.000
GSDMB	4.043	4.162	0.119	0.079	0.086
GSDMC	0.074	0.601	0.527	0.000	0.000
GSDMD	5.526	6.470	0.944	0.000	0.000

GSK3A	4.007	4.970	0.963	0.000	0.000
GSK3B	3.375	3.958	0.583	0.000	0.000
GSN	5.410	5.497	0.087	0.157	0.168
GSPT1	4.624	5.085	0.461	0.000	0.000
GSPT2	3.224	2.360	-0.864	0.000	0.000
GSR	5.207	5.802	0.595	0.000	0.000
GSS	5.512	6.102	0.590	0.000	0.000
GSTA1	10.692	9.679	-1.012	0.000	0.000
GSTA2	7.991	5.979	-2.012	0.000	0.000
GSTA4	3.777	5.157	1.381	0.000	0.000
GSTCD	1.081	1.708	0.627	0.000	0.000
GSTK1	7.409	7.716	0.306	0.000	0.000
GSTM1	3.621	2.902	-0.720	0.000	0.000
GSTM2	2.454	2.030	-0.424	0.000	0.000
GSTM3	2.978	3.363	0.386	0.000	0.000
GSTM4	3.790	4.158	0.368	0.000	0.000
GSTM5	0.983	0.380	-0.603	0.000	0.000
GSTO1	8.572	8.646	0.074	0.058	0.063
GSTO2	1.185	1.759	0.573	0.000	0.000
GSTP1	5.435	5.101	-0.334	0.000	0.000
GSTT2B	1.513	1.327	-0.186	0.005	0.005
GSTZ1	5.252	3.684	-1.568	0.000	0.000
GTDC1	1.432	1.877	0.444	0.000	0.000
GTF2A1	2.470	2.908	0.438	0.000	0.000
GTF2A2	4.480	5.148	0.668	0.000	0.000
GTF2B	4.576	4.928	0.352	0.000	0.000
GTF2E1	2.215	3.124	0.909	0.000	0.000
GTF2E2	4.077	4.344	0.267	0.000	0.000
GTF2F1	4.713	5.416	0.703	0.000	0.000
GTF2F2	3.164	3.848	0.685	0.000	0.000
GTF2H1	3.267	3.912	0.645	0.000	0.000
GTF2H2	0.718	1.169	0.451	0.000	0.000
GTF2H3	2.856	3.534	0.678	0.000	0.000
GTF2H4	2.986	3.500	0.514	0.000	0.000
GTF2H5	3.017	3.523	0.507	0.000	0.000
GTF2IRD1	1.720	2.930	1.210	0.000	0.000
GTF3A	6.316	6.710	0.395	0.000	0.000
GTF3C1	3.600	4.120	0.521	0.000	0.000
GTF3C2	3.484	4.341	0.857	0.000	0.000
GTF3C3	2.658	3.453	0.794	0.000	0.000
GTF3C4	1.828	2.301	0.473	0.000	0.000
GTF3C5	4.284	5.040	0.757	0.000	0.000
GTF3C6	5.432	6.238	0.806	0.000	0.000
GTPBP1	3.227	3.552	0.325	0.000	0.000
GTPBP10	2.816	3.146	0.331	0.000	0.000
GTPBP2	3.589	4.802	1.213	0.000	0.000
GTPBP3	2.722	3.578	0.856	0.000	0.000
GTPBP4	3.389	4.403	1.014	0.000	0.000
GTPBP8	2.118	2.390	0.272	0.000	0.000
GTSE1	0.439	2.143	1.704	0.000	0.000
GTSF1	0.433	1.547	1.114	0.000	0.000
GUCA1B	1.683	1.685	0.002	0.968	0.970

GUCA2A	0.402	0.828	0.426	0.000	0.000
GUCA2B	2.784	2.162	-0.623	0.000	0.000
GUCY1A2	0.504	0.638	0.134	0.000	0.000
GUCY2C	0.347	0.999	0.652	0.000	0.000
GUCY2D	0.188	0.353	0.165	0.000	0.000
GUF1	3.538	4.047	0.509	0.000	0.000
GUK1	6.382	7.192	0.810	0.000	0.000
GULP1	0.464	0.786	0.323	0.000	0.000
GUSB	6.420	6.709	0.289	0.000	0.000
GXYLT1	2.870	3.258	0.388	0.000	0.000
GXYLT2	0.261	0.674	0.413	0.000	0.000
GYG1	3.219	4.072	0.853	0.000	0.000
GYG2	3.814	3.723	-0.091	0.130	0.139
GYPC	3.747	3.750	0.003	0.955	0.957
GYPE	0.200	0.173	-0.027	0.055	0.061
GYS1	3.001	3.681	0.680	0.000	0.000
GYS2	6.577	4.168	-2.409	0.000	0.000
GZF1	2.522	3.020	0.498	0.000	0.000
GZMA	3.898	3.484	-0.414	0.000	0.000
GZMB	2.107	1.950	-0.157	0.019	0.021
GZMH	2.890	2.443	-0.447	0.000	0.000
GZMK	3.053	2.205	-0.848	0.000	0.000
H1F0	6.983	8.270	1.287	0.000	0.000
H1FX	6.424	6.935	0.511	0.000	0.000
H2AFJ	5.001	5.700	0.700	0.000	0.000
H2AFV	5.071	5.937	0.866	0.000	0.000
H2AFX	3.604	5.268	1.664	0.000	0.000
H2AFY	3.624	4.771	1.148	0.000	0.000
H2AFY2	2.958	3.508	0.550	0.000	0.000
H2AFZ	5.430	6.884	1.454	0.000	0.000
H3F3B	7.156	7.774	0.617	0.000	0.000
H3F3C	0.606	0.664	0.058	0.018	0.020
H6PD	5.625	5.686	0.062	0.216	0.229
HAAO	7.779	6.610	-1.169	0.000	0.000
HABP2	7.877	7.059	-0.818	0.000	0.000
HABP4	2.807	3.611	0.804	0.000	0.000
HACE1	1.034	1.623	0.589	0.000	0.000
HACL1	4.793	5.052	0.260	0.000	0.000
HADH	6.698	6.334	-0.364	0.000	0.000
HADHA	7.374	7.511	0.138	0.000	0.000
HADHB	7.257	7.143	-0.115	0.001	0.001
HAGH	6.627	6.272	-0.355	0.000	0.000
HAGHL	0.733	1.351	0.618	0.000	0.000
HAL	6.327	4.814	-1.513	0.000	0.000
HAMP	8.008	3.253	-4.755	0.000	0.000
HAND2	2.575	1.008	-1.567	0.000	0.000
HAO1	8.666	7.272	-1.394	0.000	0.000
HAO2	7.680	4.452	-3.228	0.000	0.000
HAP1	0.270	0.276	0.006	0.811	0.819
HAPLN2	0.102	0.173	0.071	0.000	0.000
HAPLN3	1.297	1.792	0.495	0.000	0.000
HAPLN4	1.588	1.019	-0.570	0.000	0.000

HARBI1	1.436	2.042	0.605	0.000	0.000
HARS	3.755	4.423	0.669	0.000	0.000
HARS2	3.846	4.721	0.875	0.000	0.000
HAS1	0.691	0.467	-0.223	0.000	0.000
HAS2	1.029	0.676	-0.354	0.000	0.000
HAS3	1.349	1.625	0.277	0.000	0.000
HAT1	3.124	3.996	0.872	0.000	0.000
HAUS1	2.833	3.807	0.974	0.000	0.000
HAUS2	2.587	3.403	0.816	0.000	0.000
HAUS3	1.889	2.661	0.773	0.000	0.000
HAUS4	3.726	4.676	0.950	0.000	0.000
HAUS5	2.314	3.374	1.060	0.000	0.000
HAUS6	2.003	2.676	0.672	0.000	0.000
HAUS7	1.959	2.477	0.518	0.000	0.000
HAUS8	1.703	2.181	0.478	0.000	0.000
HAVCR1	0.154	0.724	0.570	0.000	0.000
HAVCR2	2.532	2.544	0.013	0.841	0.848
HAX1	6.059	7.054	0.995	0.000	0.000
HBA1	3.347	2.075	-1.272	0.000	0.000
HBA2	5.407	3.705	-1.702	0.000	0.000
HBB	6.466	4.753	-1.714	0.000	0.000
HBEGF	2.775	2.763	-0.012	0.832	0.839
HBG2	0.291	0.213	-0.077	0.002	0.002
HBP1	4.274	4.735	0.462	0.000	0.000
HBS1L	3.332	3.811	0.479	0.000	0.000
HCAR2	1.066	0.937	-0.129	0.010	0.012
HCAR3	0.787	0.867	0.080	0.099	0.107
HCCS	4.118	4.716	0.598	0.000	0.000
HCFC1	3.305	4.295	0.990	0.000	0.000
HCFC1R1	4.623	5.375	0.753	0.000	0.000
HCFC2	2.577	2.931	0.355	0.000	0.000
HCG27	1.111	1.626	0.515	0.000	0.000
HCK	3.243	2.931	-0.312	0.000	0.000
HCLS1	4.472	3.957	-0.515	0.000	0.000
HCN2	0.285	0.821	0.536	0.000	0.000
HCN3	3.685	4.275	0.590	0.000	0.000
HCST	4.164	4.116	-0.048	0.468	0.484
HDAC1	4.959	5.835	0.876	0.000	0.000
HDAC10	2.925	3.552	0.627	0.000	0.000
HDAC11	1.902	3.533	1.631	0.000	0.000
HDAC2	2.873	3.688	0.815	0.000	0.000
HDAC3	4.359	4.949	0.590	0.000	0.000
HDAC4	1.183	1.791	0.608	0.000	0.000
HDAC5	3.505	4.457	0.952	0.000	0.000
HDAC6	5.437	5.029	-0.408	0.000	0.000
HDAC7	2.725	3.423	0.698	0.000	0.000
HDAC8	2.653	3.310	0.657	0.000	0.000
HDAC9	0.754	0.592	-0.162	0.000	0.000
HDC	1.006	0.721	-0.285	0.000	0.000
HDDC2	2.953	3.514	0.561	0.000	0.000
HDGF	7.279	8.075	0.796	0.000	0.000
HDHD2	3.891	4.182	0.292	0.000	0.000

HDHD3	5.978	6.393	0.415	0.000	0.000
HDLBP	6.752	7.198	0.446	0.000	0.000
HDX	0.681	0.817	0.137	0.000	0.000
HEATR1	2.692	3.713	1.021	0.000	0.000
HEATR3	3.027	3.542	0.515	0.000	0.000
HEATR4	0.660	0.736	0.076	0.002	0.003
HEATR5A	2.324	2.933	0.610	0.000	0.000
HEATR5B	2.907	3.161	0.254	0.000	0.000
HEATR6	1.871	2.806	0.934	0.000	0.000
HEBP1	5.887	5.643	-0.245	0.000	0.000
HEBP2	4.309	4.522	0.213	0.000	0.000
HECA	3.339	3.397	0.058	0.139	0.149
HECTD1	4.920	5.063	0.144	0.000	0.000
HECTD2	1.212	1.783	0.571	0.000	0.000
HECTD3	4.549	4.717	0.169	0.000	0.000
HECW2	1.309	1.490	0.182	0.000	0.000
HEG1	2.528	3.140	0.612	0.000	0.000
HELB	0.558	0.610	0.053	0.008	0.009
HELLS	0.652	1.842	1.190	0.000	0.000
HELQ	2.705	2.842	0.137	0.000	0.000
HELZ	2.838	3.145	0.307	0.000	0.000
HEMK1	2.511	2.832	0.321	0.000	0.000
HENMT1	1.619	1.761	0.141	0.028	0.031
HEPACAM	3.042	1.583	-1.459	0.000	0.000
HEPH	1.302	1.389	0.087	0.078	0.084
HEPHL1	0.161	0.202	0.041	0.001	0.001
HEPN1	2.609	1.295	-1.314	0.000	0.000
HERC1	3.006	3.520	0.514	0.000	0.000
HERC2	2.615	3.214	0.598	0.000	0.000
HERC3	2.655	2.697	0.042	0.386	0.402
HERC4	2.745	3.211	0.466	0.000	0.000
HERC5	2.991	2.505	-0.486	0.000	0.000
HERC6	2.246	2.184	-0.062	0.237	0.251
HERPUD1	6.272	6.203	-0.068	0.072	0.079
HERPUD2	4.079	4.580	0.500	0.000	0.000
HES1	5.778	5.616	-0.162	0.002	0.003
HES2	0.076	0.371	0.294	0.000	0.000
HES4	2.732	3.518	0.785	0.000	0.000
HES5	0.177	0.550	0.373	0.000	0.000
HES6	1.824	3.358	1.535	0.000	0.000
HESX1	1.391	1.608	0.217	0.000	0.000
HEXA	4.071	4.992	0.921	0.000	0.000
HEXB	5.915	6.865	0.950	0.000	0.000
HEXIM1	4.532	4.712	0.180	0.000	0.000
HEY1	1.452	2.628	1.176	0.000	0.000
HEY2	2.698	2.448	-0.251	0.000	0.000
HEYL	2.254	2.754	0.500	0.000	0.000
HFE	2.510	3.274	0.764	0.000	0.000
HGD	8.762	8.035	-0.727	0.000	0.000
HGF	2.814	1.372	-1.442	0.000	0.000
HGFAC	7.759	4.367	-3.392	0.000	0.000
HGS	3.824	5.039	1.216	0.000	0.000

HGSNAT	3.966	4.414	0.448	0.000	0.000
HHAT	1.460	2.350	0.890	0.000	0.000
HHATL	0.188	0.202	0.014	0.655	0.668
HHEX	5.460	5.866	0.406	0.000	0.000
HHIP	1.401	0.328	-1.074	0.000	0.000
HHIPL1	0.590	0.621	0.032	0.259	0.273
HHIPL2	0.231	1.063	0.832	0.000	0.000
HHLA3	3.970	4.109	0.139	0.001	0.001
HIBADH	6.812	6.678	-0.134	0.002	0.003
HIBCH	4.670	4.426	-0.244	0.000	0.000
HIC1	1.578	1.446	-0.132	0.001	0.001
HIC2	1.165	1.900	0.735	0.000	0.000
HIF1A	4.677	5.195	0.518	0.000	0.000
HIF3A	0.719	0.686	-0.033	0.454	0.470
HIGD1A	7.603	7.480	-0.123	0.003	0.003
HIGD1B	0.586	2.097	1.512	0.000	0.000
HIGD1C	0.436	0.433	-0.003	0.911	0.914
HINFP	1.918	2.571	0.653	0.000	0.000
HINT1	8.273	8.839	0.566	0.000	0.000
HINT2	6.275	6.148	-0.127	0.000	0.000
HINT3	4.185	4.624	0.439	0.000	0.000
HIP1	2.694	3.425	0.731	0.000	0.000
HIP1R	4.027	4.632	0.606	0.000	0.000
HIPK1	3.159	3.740	0.581	0.000	0.000
HIPK2	3.241	3.777	0.536	0.000	0.000
HIPK3	3.752	3.780	0.028	0.580	0.595
HIPK4	0.147	0.214	0.067	0.000	0.000
HIRA	3.292	3.895	0.603	0.000	0.000
HIRIP3	3.386	3.925	0.539	0.000	0.000
HIST1H1B	0.071	0.384	0.313	0.000	0.000
HIST1H1C	7.143	8.233	1.089	0.000	0.000
HIST1H1E	0.493	1.146	0.654	0.000	0.000
HIST1H2AA	0.226	0.366	0.140	0.000	0.000
HIST1H2AC	5.227	6.076	0.849	0.000	0.000
HIST1H2AD	0.622	1.545	0.923	0.000	0.000
HIST1H2AG	0.779	1.581	0.803	0.000	0.000
HIST1H2AI	0.183	0.952	0.769	0.000	0.000
HIST1H2BC	2.829	3.461	0.632	0.000	0.000
HIST1H2BD	5.339	5.927	0.588	0.000	0.000
HIST1H2BJ	0.966	2.081	1.114	0.000	0.000
HIST1H2BK	6.998	8.164	1.166	0.000	0.000
HIST1H2BL	0.088	0.517	0.429	0.000	0.000
HIST1H2BN	0.436	0.903	0.467	0.000	0.000
HIST1H3D	1.274	2.326	1.052	0.000	0.000
HIST1H4C	0.774	1.209	0.435	0.000	0.000
HIST1H4H	1.082	2.692	1.610	0.000	0.000
HIST1H4J	1.522	2.009	0.487	0.000	0.000
HIST2H2AB	0.166	0.329	0.163	0.000	0.000
HIST2H2AC	1.451	2.517	1.066	0.000	0.000
HIST2H2BE	2.987	4.076	1.089	0.000	0.000
HIST2H2BF	0.337	0.930	0.593	0.000	0.000
HIST2H3D	0.187	0.574	0.387	0.000	0.000

HIST3H2A	1.001	1.577	0.576	0.000	0.000
HIST4H4	0.351	0.520	0.169	0.000	0.000
HIVEP1	2.812	2.698	-0.114	0.011	0.012
HIVEP2	2.318	2.445	0.127	0.002	0.002
HIVEP3	0.826	0.857	0.030	0.331	0.347
HJURP	0.606	2.800	2.194	0.000	0.000
HK1	2.701	3.062	0.361	0.000	0.000
HK2	1.276	1.888	0.612	0.000	0.000
HK3	3.246	2.151	-1.095	0.000	0.000
HKDC1	2.494	4.087	1.593	0.000	0.000
HKR1	2.824	3.320	0.496	0.000	0.000
HLA-A	9.096	9.927	0.831	0.000	0.000
HLA-B	9.534	9.906	0.373	0.000	0.000
HLA-C	8.950	9.458	0.509	0.000	0.000
HLA-DMA	5.163	5.660	0.497	0.000	0.000
HLA-DMB	3.651	3.929	0.278	0.000	0.000
HLA-DOA	2.989	2.747	-0.242	0.001	0.001
HLA-DOB	1.654	1.966	0.312	0.000	0.000
HLA-DPA1	6.101	5.990	-0.111	0.175	0.187
HLA-DPB1	6.770	6.593	-0.177	0.021	0.024
HLA-DQA1	3.882	3.781	-0.101	0.259	0.273
HLA-DQA2	3.227	3.074	-0.153	0.149	0.159
HLA-DQB1	4.215	4.387	0.172	0.062	0.068
HLA-DQB2	1.971	2.036	0.065	0.408	0.424
HLA-DRA	8.915	8.926	0.011	0.893	0.897
HLA-DRB1	7.673	7.605	-0.068	0.429	0.445
HLA-DRB5	5.327	5.265	-0.062	0.600	0.614
HLA-E	8.626	8.875	0.248	0.000	0.000
HLA-F	5.481	5.976	0.495	0.000	0.000
HLCS	2.859	3.217	0.358	0.000	0.000
HLF	5.395	4.769	-0.625	0.000	0.000
HLTF	2.939	4.171	1.232	0.000	0.000
HLX	2.986	3.117	0.131	0.002	0.003
HM13	4.936	6.035	1.099	0.000	0.000
HMBOX1	2.199	2.208	0.009	0.791	0.800
HMBS	3.333	4.210	0.877	0.000	0.000
HMCN1	1.534	1.460	-0.074	0.170	0.182
HMG20A	3.348	3.751	0.403	0.000	0.000
HMG20B	4.867	5.435	0.568	0.000	0.000
HMGA1	4.166	6.003	1.837	0.000	0.000
HMGB1	6.043	6.476	0.434	0.000	0.000
HMGB2	4.165	5.732	1.567	0.000	0.000
HMGB3	4.562	5.177	0.615	0.000	0.000
HMGCL	6.815	6.046	-0.769	0.000	0.000
HMGCLL1	0.545	0.132	-0.412	0.000	0.000
HMGCR	4.603	5.092	0.489	0.000	0.000
HMGCS1	6.363	6.894	0.531	0.000	0.000
HMGCS2	10.603	9.489	-1.114	0.000	0.000
HMGN1	5.019	6.044	1.025	0.000	0.000
HMGN3	6.806	6.879	0.073	0.087	0.094
HMGN4	4.459	5.659	1.201	0.000	0.000
HMGN5	3.257	2.718	-0.538	0.000	0.000

HMGXB3	3.138	4.246	1.108	0.000	0.000
HMGXB4	2.469	3.232	0.764	0.000	0.000
HMMR	0.810	2.947	2.137	0.000	0.000
HMOX1	6.318	5.697	-0.621	0.000	0.000
HMOX2	5.370	5.409	0.039	0.284	0.298
HMSD	0.256	0.395	0.139	0.000	0.000
HNF1A	3.540	4.153	0.613	0.000	0.000
HNF4A	5.884	6.289	0.406	0.000	0.000
HNF4G	2.957	3.239	0.281	0.000	0.000
HNMT	6.207	6.132	-0.076	0.093	0.100
HNRNPA0	4.475	5.201	0.726	0.000	0.000
HNRNPA1	6.642	7.638	0.996	0.000	0.000
HNRNPA2B1	7.387	8.104	0.717	0.000	0.000
HNRNPA3	5.870	6.836	0.966	0.000	0.000
HNRNPAB	6.222	7.065	0.842	0.000	0.000
HNRNPD	5.484	5.886	0.403	0.000	0.000
HNRNPF	6.754	7.237	0.483	0.000	0.000
HNRNPH1	5.977	6.557	0.580	0.000	0.000
HNRNPH2	5.820	6.428	0.608	0.000	0.000
HNRNPH3	5.094	5.510	0.417	0.000	0.000
HNRNPK	7.143	7.723	0.580	0.000	0.000
HNRNPL	5.448	6.139	0.691	0.000	0.000
HNRNPM	5.714	6.304	0.590	0.000	0.000
HNRNPR	4.109	5.018	0.909	0.000	0.000
HNRNPU	5.777	6.731	0.954	0.000	0.000
HNRNPUL1	4.986	5.983	0.997	0.000	0.000
HNRNPUL2	4.246	5.008	0.762	0.000	0.000
HOGA1	4.775	3.196	-1.580	0.000	0.000
HOMER1	0.978	1.615	0.637	0.000	0.000
HOMER2	3.462	3.278	-0.184	0.003	0.003
HOMER3	2.303	3.200	0.897	0.000	0.000
HOMEZ	2.097	2.783	0.686	0.000	0.000
HOOK1	4.293	4.014	-0.279	0.000	0.000
HOOK2	3.557	3.761	0.204	0.000	0.000
HOPX	0.766	1.168	0.403	0.000	0.000
HORMAD1	0.178	0.282	0.105	0.000	0.000
HORMAD2	0.872	0.821	-0.051	0.294	0.309
HOXA1	0.308	0.346	0.039	0.091	0.099
HOXA10	0.098	1.246	1.148	0.000	0.000
HOXA13	0.240	1.623	1.383	0.000	0.000
HOXA2	0.346	0.422	0.076	0.002	0.003
HOXA3	0.470	1.283	0.813	0.000	0.000
HOXA4	0.885	1.064	0.179	0.000	0.000
HOXA5	0.854	1.311	0.457	0.000	0.000
HOXA9	0.068	0.272	0.204	0.000	0.000
HOXB2	1.881	1.960	0.079	0.113	0.121
HOXB3	1.295	1.374	0.080	0.099	0.107
HOXB4	0.982	0.873	-0.109	0.001	0.002
HOXB5	0.809	0.935	0.126	0.002	0.002
HOXB6	0.904	0.938	0.033	0.435	0.451
HOXB7	0.975	1.379	0.403	0.000	0.000
HOXD1	0.014	0.514	0.500	0.000	0.000

HOXD3	0.051	0.393	0.342	0.000	0.000
HOXD4	0.038	0.684	0.646	0.000	0.000
HOXD8	0.378	0.978	0.600	0.000	0.000
HOXD9	0.389	1.323	0.934	0.000	0.000
HP	13.576	11.158	-2.418	0.000	0.000
HP1BP3	4.865	5.446	0.581	0.000	0.000
HPCA	0.113	0.238	0.124	0.000	0.000
HPCAL1	3.952	4.536	0.584	0.000	0.000
HPD	10.810	8.592	-2.218	0.000	0.000
HPDL	0.210	0.653	0.443	0.000	0.000
HPGD	5.412	3.933	-1.480	0.000	0.000
HPGDS	0.909	1.030	0.121	0.004	0.005
HPN	8.600	8.178	-0.422	0.000	0.000
HPR	8.670	7.761	-0.909	0.000	0.000
HPRT1	5.327	5.629	0.303	0.000	0.000
HPS1	4.271	4.898	0.627	0.000	0.000
HPS3	4.906	4.904	-0.001	0.979	0.980
HPS4	2.509	3.358	0.849	0.000	0.000
HPS5	4.129	3.748	-0.381	0.000	0.000
HPS6	3.096	3.655	0.559	0.000	0.000
HPSE	1.255	1.335	0.079	0.087	0.094
HPX	11.609	9.865	-1.745	0.000	0.000
HR	0.350	0.445	0.095	0.004	0.004
HRAS	4.101	5.151	1.050	0.000	0.000
HRASLS	0.198	0.227	0.029	0.226	0.239
HRASLS2	1.716	1.677	-0.039	0.643	0.657
HRC	1.093	1.224	0.131	0.001	0.001
HRCT1	1.715	3.440	1.725	0.000	0.000
HRG	10.716	8.885	-1.830	0.000	0.000
HRH1	0.694	0.961	0.267	0.000	0.000
HRH2	0.299	0.358	0.059	0.035	0.038
HRH4	0.115	0.119	0.004	0.595	0.609
HS1BP3	3.051	3.974	0.923	0.000	0.000
HS2ST1	3.122	3.783	0.661	0.000	0.000
HS3ST1	0.617	0.733	0.116	0.004	0.004
HS3ST2	0.768	1.348	0.579	0.000	0.000
HS3ST3A1	0.696	0.474	-0.223	0.000	0.000
HS3ST3B1	3.403	2.743	-0.660	0.000	0.000
HS6ST1	4.163	5.099	0.937	0.000	0.000
HSBP1	4.201	4.611	0.410	0.000	0.000
HSBP1L1	4.590	4.556	-0.034	0.413	0.429
HSCB	4.625	4.794	0.168	0.000	0.000
HSD11B1	9.282	7.142	-2.139	0.000	0.000
HSD11B1L	1.888	2.082	0.195	0.000	0.000
HSD11B2	2.092	2.844	0.752	0.000	0.000
HSD17B1	1.123	1.676	0.553	0.000	0.000
HSD17B10	7.979	7.916	-0.063	0.056	0.061
HSD17B11	7.195	7.402	0.207	0.000	0.000
HSD17B12	4.781	4.934	0.153	0.000	0.000
HSD17B13	8.427	4.567	-3.860	0.000	0.000
HSD17B14	4.205	5.135	0.930	0.000	0.000
HSD17B2	5.856	4.787	-1.069	0.000	0.000

HSD17B3	2.549	2.662	0.113	0.102	0.110
HSD17B4	7.174	7.173	0.000	0.997	0.997
HSD17B6	9.534	8.148	-1.385	0.000	0.000
HSD17B7	3.633	4.305	0.672	0.000	0.000
HSD3B1	0.410	0.493	0.083	0.063	0.069
HSD3B2	0.646	0.387	-0.259	0.000	0.000
HSD3B7	5.571	5.994	0.423	0.000	0.000
HSDL1	2.412	3.297	0.885	0.000	0.000
HSDL2	6.551	6.532	-0.020	0.636	0.649
HSF1	4.540	5.552	1.012	0.000	0.000
HSF2	2.296	3.224	0.928	0.000	0.000
HSF2BP	0.212	0.942	0.730	0.000	0.000
HSF4	2.873	3.787	0.913	0.000	0.000
HSH2D	2.225	1.993	-0.232	0.001	0.001
HSP90AA1	8.260	9.010	0.750	0.000	0.000
HSP90AB1	8.287	9.736	1.450	0.000	0.000
HSP90B1	8.876	9.567	0.692	0.000	0.000
HSPA12A	1.049	1.316	0.267	0.000	0.000
HSPA12B	1.626	2.287	0.661	0.000	0.000
HSPA13	3.216	3.983	0.767	0.000	0.000
HSPA14	2.648	3.544	0.896	0.000	0.000
HSPA1A	6.953	7.814	0.860	0.000	0.000
HSPA1B	5.847	6.567	0.720	0.000	0.000
HSPA1L	1.198	1.560	0.362	0.000	0.000
HSPA2	1.741	2.307	0.567	0.000	0.000
HSPA4	5.030	6.086	1.056	0.000	0.000
HSPA4L	2.018	2.157	0.139	0.004	0.005
HSPA5	8.083	9.110	1.027	0.000	0.000
HSPA6	2.309	2.823	0.514	0.000	0.000
HSPA8	8.253	8.800	0.546	0.000	0.000
HSPA9	7.290	7.926	0.636	0.000	0.000
HSPB1	7.960	9.564	1.604	0.000	0.000
HSPB11	3.664	4.529	0.866	0.000	0.000
HSPB2	1.167	1.047	-0.120	0.004	0.005
HSPB2-C11orf	1.432	1.527	0.094	0.000	0.000
HSPB6	4.226	3.302	-0.924	0.000	0.000
HSPB7	0.745	0.712	-0.034	0.452	0.468
HSPB8	2.483	2.891	0.408	0.000	0.000
HSPBAP1	1.909	2.507	0.597	0.000	0.000
HSPBP1	4.535	5.381	0.846	0.000	0.000
HSPD1	7.882	8.570	0.688	0.000	0.000
HSPE1	7.171	7.788	0.617	0.000	0.000
HSPG2	3.357	4.213	0.855	0.000	0.000
HSPH1	4.049	4.933	0.884	0.000	0.000
HTATIP2	5.533	6.666	1.133	0.000	0.000
HTATSF1	4.676	5.615	0.940	0.000	0.000
HTR1D	0.175	0.935	0.760	0.000	0.000
HTR2B	1.760	1.626	-0.134	0.038	0.042
HTR7	0.408	0.354	-0.054	0.019	0.022
HTRA1	6.610	6.945	0.335	0.000	0.000
HTRA2	3.906	4.667	0.761	0.000	0.000
HTRA3	1.490	1.976	0.487	0.000	0.000

HTRA4	0.277	0.539	0.262	0.000	0.000
HTT	2.887	3.611	0.724	0.000	0.000
HUNK	0.675	0.907	0.232	0.000	0.000
HUS1	3.018	3.584	0.566	0.000	0.000
HUS1B	0.340	0.482	0.142	0.000	0.000
HUWE1	4.139	4.778	0.639	0.000	0.000
HVCN1	1.725	1.845	0.120	0.007	0.008
HYAL1	6.717	6.292	-0.425	0.000	0.000
HYAL2	4.281	4.896	0.615	0.000	0.000
HYAL3	2.278	2.905	0.627	0.000	0.000
HYI	4.871	5.066	0.196	0.000	0.000
HYLS1	2.058	2.657	0.599	0.000	0.000
HYOU1	4.979	6.112	1.133	0.000	0.000
IAH1	4.011	4.529	0.518	0.000	0.000
IAPP	0.815	0.534	-0.281	0.000	0.000
IARS	3.879	4.850	0.971	0.000	0.000
IARS2	5.516	6.483	0.967	0.000	0.000
IBA57	1.694	2.192	0.498	0.000	0.000
IBTK	4.595	4.898	0.304	0.000	0.000
ICA1	2.611	3.240	0.630	0.000	0.000
ICA1L	0.349	0.535	0.185	0.000	0.000
ICAM1	5.096	5.302	0.207	0.012	0.013
ICAM3	3.361	3.296	-0.065	0.078	0.085
ICAM5	0.216	0.448	0.232	0.000	0.000
ICK	2.665	3.737	1.072	0.000	0.000
ICMT	4.012	4.628	0.616	0.000	0.000
ICOS	0.935	1.003	0.068	0.179	0.190
ICOSLG	1.574	1.827	0.253	0.000	0.000
ID1	7.128	5.224	-1.904	0.000	0.000
ID2	8.329	7.526	-0.803	0.000	0.000
ID3	5.087	4.774	-0.313	0.000	0.000
ID4	2.763	2.012	-0.751	0.000	0.000
IDE	3.282	3.901	0.619	0.000	0.000
IDH1	7.436	7.658	0.222	0.000	0.000
IDH2	7.449	7.543	0.095	0.043	0.047
IDH3B	5.589	6.260	0.670	0.000	0.000
IDH3G	4.823	5.539	0.716	0.000	0.000
IDI1	5.958	6.747	0.789	0.000	0.000
IDO1	1.520	1.784	0.264	0.000	0.000
IDO2	2.970	0.990	-1.979	0.000	0.000
IDS	4.838	4.962	0.124	0.008	0.009
IDUA	3.006	3.994	0.988	0.000	0.000
IER2	6.361	5.664	-0.697	0.000	0.000
IER3	5.596	5.583	-0.013	0.883	0.887
IER3IP1	4.615	5.422	0.807	0.000	0.000
IER5	3.391	4.244	0.853	0.000	0.000
IER5L	3.378	3.519	0.141	0.047	0.051
IFFO1	3.119	3.836	0.717	0.000	0.000
IFFO2	2.388	2.745	0.358	0.000	0.000
IFI16	4.300	4.045	-0.254	0.000	0.000
IFI27	6.132	6.554	0.422	0.001	0.001
IFI27L1	2.753	3.805	1.052	0.000	0.000

IFI27L2	2.984	3.642	0.658	0.000	0.000
IFI30	3.774	4.627	0.853	0.000	0.000
IFI35	4.910	5.714	0.804	0.000	0.000
IFI44	4.764	4.635	-0.129	0.092	0.099
IFI44L	2.396	2.000	-0.396	0.000	0.000
IFI6	7.525	7.749	0.225	0.018	0.020
IFIH1	3.566	3.846	0.280	0.000	0.000
IFIT1	4.809	4.516	-0.294	0.000	0.000
IFIT2	3.962	4.034	0.072	0.279	0.293
IFIT3	4.528	4.704	0.176	0.005	0.006
IFIT5	3.471	3.790	0.319	0.000	0.000
IFITM1	6.923	5.977	-0.947	0.000	0.000
IFITM10	3.430	2.615	-0.815	0.000	0.000
IFITM2	8.279	8.039	-0.240	0.000	0.000
IFITM3	10.222	10.672	0.451	0.000	0.000
IFNAR1	5.091	5.099	0.008	0.805	0.813
IFNG	0.870	0.772	-0.098	0.057	0.063
IFNGR1	5.998	6.359	0.361	0.000	0.000
IFNGR2	4.761	5.448	0.687	0.000	0.000
IFRD1	3.450	4.036	0.586	0.000	0.000
IFRD2	5.030	5.696	0.667	0.000	0.000
IFT122	2.518	2.688	0.170	0.000	0.000
IFT140	1.754	2.065	0.311	0.000	0.000
IFT172	2.110	2.706	0.596	0.000	0.000
IFT20	4.168	4.741	0.573	0.000	0.000
IFT27	2.618	3.351	0.733	0.000	0.000
IFT46	3.063	2.808	-0.255	0.000	0.000
IFT52	2.863	3.918	1.055	0.000	0.000
IFT57	2.634	2.767	0.133	0.024	0.027
IFT74	2.869	3.230	0.361	0.000	0.000
IFT80	1.898	2.618	0.720	0.000	0.000
IFT81	1.452	2.458	1.006	0.000	0.000
IFT88	3.105	3.128	0.023	0.477	0.492
IGBP1	4.686	5.536	0.849	0.000	0.000
IGDCC4	0.109	0.560	0.451	0.000	0.000
IGF1	3.698	2.211	-1.487	0.000	0.000
IGF1R	0.983	1.045	0.062	0.215	0.228
IGF2	8.046	5.595	-2.451	0.000	0.000
IGF2BP1	0.085	1.745	1.660	0.000	0.000
IGF2BP2	1.578	2.959	1.381	0.000	0.000
IGF2BP3	0.141	1.454	1.313	0.000	0.000
IGF2R	4.210	4.901	0.692	0.000	0.000
IGFALS	6.231	2.926	-3.305	0.000	0.000
IGFBP1	10.147	8.736	-1.410	0.000	0.000
IGFBP2	7.684	7.315	-0.369	0.000	0.000
IGFBP3	8.297	6.344	-1.953	0.000	0.000
IGFBP4	9.977	9.406	-0.571	0.000	0.000
IGFBP5	4.202	4.103	-0.099	0.225	0.238
IGFBP6	3.127	2.484	-0.643	0.000	0.000
IGFBP7	8.150	8.178	0.027	0.688	0.701
IGFBPL1	0.339	0.696	0.356	0.000	0.000
IGFLR1	2.957	3.066	0.109	0.011	0.013

IGHMBP2	3.016	3.495	0.479	0.000	0.000
IGLL5	4.488	2.693	-1.795	0.000	0.000
IGLON5	1.196	0.730	-0.467	0.000	0.000
IGSF1	0.350	1.305	0.955	0.000	0.000
IGSF10	0.430	0.272	-0.158	0.000	0.000
IGSF21	0.757	1.236	0.479	0.000	0.000
IGSF22	0.341	0.400	0.059	0.002	0.002
IGSF23	3.532	3.789	0.257	0.006	0.006
IGSF3	0.833	2.339	1.506	0.000	0.000
IGSF6	2.768	2.813	0.045	0.461	0.477
IGSF8	5.679	6.484	0.805	0.000	0.000
IGSF9	2.880	2.178	-0.702	0.000	0.000
IHH	1.443	2.377	0.933	0.000	0.000
IK	5.048	5.973	0.925	0.000	0.000
IKBIP	2.471	3.324	0.854	0.000	0.000
IKBKB	3.029	3.455	0.426	0.000	0.000
IKZF1	1.741	1.477	-0.264	0.000	0.000
IKZF2	0.902	0.878	-0.025	0.438	0.454
IKZF3	0.750	0.650	-0.099	0.008	0.010
IKZF4	1.303	1.502	0.199	0.000	0.000
IKZF5	2.910	3.164	0.254	0.000	0.000
IL10	0.862	0.573	-0.289	0.000	0.000
IL10RA	3.378	2.919	-0.460	0.000	0.000
IL10RB	4.619	5.064	0.445	0.000	0.000
IL11	0.115	0.321	0.206	0.000	0.000
IL11RA	3.875	4.120	0.245	0.000	0.000
IL12A	0.307	0.614	0.307	0.000	0.000
IL12RB1	1.326	1.284	-0.042	0.348	0.363
IL12RB2	0.471	0.551	0.080	0.004	0.005
IL13RA1	6.227	6.387	0.160	0.000	0.000
IL13RA2	2.450	0.925	-1.525	0.000	0.000
IL15	1.284	1.238	-0.046	0.219	0.232
IL15RA	3.716	3.805	0.090	0.119	0.128
IL16	2.149	1.888	-0.261	0.000	0.000
IL17B	0.049	0.217	0.169	0.000	0.000
IL17D	0.327	1.263	0.936	0.000	0.000
IL17RA	2.627	2.936	0.309	0.000	0.000
IL17RB	5.071	5.647	0.576	0.000	0.000
IL17RC	5.222	4.993	-0.229	0.000	0.000
IL17RD	0.267	0.377	0.110	0.000	0.000
IL17RE	2.382	2.638	0.256	0.000	0.000
IL18	3.059	2.785	-0.274	0.000	0.000
IL18BP	2.495	3.035	0.540	0.000	0.000
IL18R1	2.696	1.905	-0.792	0.000	0.000
IL18RAP	1.593	1.126	-0.468	0.000	0.000
IL1B	1.877	1.173	-0.703	0.000	0.000
IL1R1	5.398	5.178	-0.220	0.000	0.000
IL1R2	2.433	3.038	0.605	0.000	0.000
IL1RAP	4.192	3.277	-0.915	0.000	0.000
IL1RAPL2	0.743	0.334	-0.409	0.000	0.000
IL1RL1	1.291	0.427	-0.864	0.000	0.000
IL1RL2	1.506	1.461	-0.046	0.414	0.430

IL1RN	5.580	5.023	-0.557	0.000	0.000
IL20RA	0.582	0.585	0.003	0.959	0.961
IL20RB	1.665	1.405	-0.260	0.000	0.000
IL21R	0.818	0.796	-0.022	0.620	0.634
IL22RA1	2.670	3.351	0.681	0.000	0.000
IL23A	1.187	1.251	0.064	0.050	0.055
IL27	3.604	2.957	-0.648	0.000	0.000
IL27RA	2.108	2.565	0.458	0.000	0.000
IL2RA	0.825	1.078	0.253	0.000	0.000
IL2RB	2.694	2.099	-0.595	0.000	0.000
IL2RG	3.656	3.787	0.131	0.124	0.134
IL32	7.940	8.461	0.522	0.000	0.000
IL33	3.981	2.884	-1.096	0.000	0.000
IL34	1.831	2.084	0.253	0.000	0.000
IL36G	0.217	0.234	0.017	0.515	0.530
IL3RA	0.835	1.167	0.332	0.000	0.000
IL4I1	2.444	2.586	0.142	0.043	0.048
IL4R	5.359	4.925	-0.434	0.000	0.000
IL6	1.455	0.837	-0.618	0.000	0.000
IL6R	5.172	5.667	0.495	0.000	0.000
IL6ST	6.226	6.025	-0.201	0.000	0.000
IL7	1.831	2.087	0.257	0.000	0.000
IL7R	2.360	1.756	-0.604	0.000	0.000
ILDR1	0.758	1.019	0.261	0.000	0.000
ILDR2	0.314	0.981	0.667	0.000	0.000
ILF2	5.778	7.256	1.479	0.000	0.000
ILF3	4.471	5.411	0.940	0.000	0.000
ILKAP	3.531	4.038	0.507	0.000	0.000
ILVBL	5.241	5.336	0.095	0.006	0.007
IMMP1L	2.837	3.526	0.689	0.000	0.000
IMMP2L	3.372	3.953	0.580	0.000	0.000
IMMT	5.190	5.880	0.690	0.000	0.000
IMP3	5.416	5.662	0.246	0.000	0.000
IMP4	4.843	5.476	0.632	0.000	0.000
IMPA1	4.410	4.990	0.580	0.000	0.000
IMPA2	4.087	4.740	0.653	0.000	0.000
IMPACT	3.700	3.990	0.290	0.000	0.000
IMPAD1	4.482	5.325	0.843	0.000	0.000
IMPDH1	2.471	3.094	0.623	0.000	0.000
IMPDH2	5.287	6.518	1.232	0.000	0.000
IMPG2	0.137	0.216	0.079	0.000	0.000
INCA1	1.820	1.915	0.095	0.011	0.012
INCENP	1.517	2.859	1.342	0.000	0.000
INF2	4.956	4.937	-0.019	0.664	0.676
ING1	2.839	3.202	0.363	0.000	0.000
ING2	3.418	3.690	0.273	0.000	0.000
ING3	2.167	2.453	0.287	0.000	0.000
ING4	4.128	4.758	0.629	0.000	0.000
ING5	2.563	3.147	0.584	0.000	0.000
INHA	0.319	0.771	0.451	0.000	0.000
INHBA	2.060	2.121	0.061	0.350	0.365
INHBB	4.522	4.609	0.087	0.228	0.241

INHBC	5.699	4.984	-0.715	0.000	0.000
INHBE	5.563	5.323	-0.240	0.022	0.024
INMT	4.401	2.348	-2.053	0.000	0.000
INO80	2.886	3.402	0.516	0.000	0.000
INO80B	3.774	4.431	0.656	0.000	0.000
INO80C	2.676	3.346	0.670	0.000	0.000
INO80D	2.004	2.223	0.219	0.000	0.000
INO80E	3.753	4.504	0.751	0.000	0.000
INPP1	4.343	4.123	-0.220	0.000	0.000
INPP4A	1.827	2.260	0.433	0.000	0.000
INPP4B	0.657	0.792	0.135	0.000	0.000
INPP5A	3.411	3.826	0.415	0.000	0.000
INPP5B	2.888	2.544	-0.345	0.000	0.000
INPP5D	2.610	2.573	-0.036	0.496	0.511
INPP5E	2.885	3.483	0.598	0.000	0.000
INPP5F	1.630	2.176	0.547	0.000	0.000
INPP5J	0.258	0.761	0.502	0.000	0.000
INPP5K	3.428	3.666	0.238	0.000	0.000
INPPL1	4.103	5.018	0.915	0.000	0.000
INSC	0.205	0.541	0.336	0.000	0.000
INSIG1	8.229	7.935	-0.294	0.000	0.000
INSIG2	4.925	4.946	0.021	0.657	0.670
INS-IGF2	5.890	2.596	-3.294	0.000	0.000
INSL3	0.359	0.407	0.048	0.109	0.117
INSR	4.728	5.167	0.439	0.000	0.000
INTS1	4.283	5.177	0.894	0.000	0.000
INTS10	4.529	4.224	-0.305	0.000	0.000
INTS12	3.204	3.622	0.418	0.000	0.000
INTS2	1.658	2.320	0.662	0.000	0.000
INTS3	3.743	4.608	0.864	0.000	0.000
INTS4	2.712	3.372	0.660	0.000	0.000
INTS5	3.460	4.038	0.579	0.000	0.000
INTS6	2.404	2.399	-0.005	0.882	0.887
INTS7	2.848	3.954	1.105	0.000	0.000
INTS8	2.509	3.820	1.311	0.000	0.000
INTS9	1.958	2.493	0.535	0.000	0.000
INTU	0.602	0.857	0.255	0.000	0.000
INVS	2.594	2.663	0.069	0.024	0.027
IP6K1	3.126	4.370	1.245	0.000	0.000
IP6K2	4.117	4.868	0.751	0.000	0.000
IP6K3	1.886	1.948	0.061	0.547	0.561
IPCEF1	0.914	0.665	-0.249	0.000	0.000
IPMK	2.067	2.474	0.406	0.000	0.000
IPO11	2.288	3.051	0.764	0.000	0.000
IPO13	3.392	4.216	0.824	0.000	0.000
IPO4	2.224	3.035	0.811	0.000	0.000
IPO5	4.330	4.946	0.616	0.000	0.000
IPO7	4.426	5.147	0.720	0.000	0.000
IPO8	3.652	4.080	0.427	0.000	0.000
IPO9	3.171	4.292	1.121	0.000	0.000
IPP	2.057	2.691	0.634	0.000	0.000
IQCA1	0.283	0.371	0.088	0.003	0.003

IQCB1	2.802	3.534	0.731	0.000	0.000
IQCC	1.083	1.882	0.799	0.000	0.000
IQCD	0.395	0.953	0.557	0.000	0.000
IQCE	1.607	2.537	0.930	0.000	0.000
IQCG	1.133	1.678	0.545	0.000	0.000
IQCH	0.414	0.820	0.406	0.000	0.000
IQCJ-SCHIP1	1.999	1.962	-0.037	0.353	0.369
IQCK	1.653	1.873	0.221	0.000	0.000
IQGAP1	3.494	3.773	0.279	0.000	0.000
IQGAP2	6.152	5.932	-0.220	0.000	0.000
IQGAP3	0.902	2.943	2.041	0.000	0.000
IQSEC1	4.313	5.148	0.835	0.000	0.000
IQSEC2	1.003	1.072	0.069	0.082	0.089
IQSEC3	0.460	0.337	-0.124	0.000	0.000
IRAK1	4.489	6.119	1.630	0.000	0.000
IRAK2	3.322	4.028	0.706	0.000	0.000
IRAK3	1.339	1.129	-0.211	0.000	0.000
IRAK4	2.876	3.167	0.291	0.000	0.000
IREB2	3.220	3.645	0.426	0.000	0.000
IRF1	4.380	4.517	0.137	0.010	0.012
IRF2	4.445	4.646	0.201	0.000	0.000
IRF2BP1	4.358	5.037	0.679	0.000	0.000
IRF2BP2	6.024	6.893	0.870	0.000	0.000
IRF2BPL	3.972	3.857	-0.115	0.008	0.009
IRF4	1.158	0.777	-0.380	0.000	0.000
IRF5	2.470	3.287	0.818	0.000	0.000
IRF6	4.476	5.126	0.650	0.000	0.000
IRF7	5.198	4.905	-0.293	0.000	0.000
IRF8	4.041	3.193	-0.848	0.000	0.000
IRF9	3.721	3.906	0.185	0.000	0.000
IRGQ	2.178	3.105	0.928	0.000	0.000
IRS1	3.525	4.117	0.592	0.000	0.000
IRS2	4.577	4.733	0.156	0.008	0.009
IRX3	0.683	1.853	1.170	0.000	0.000
IRX5	0.080	0.460	0.381	0.000	0.000
ISCA1	4.718	5.115	0.397	0.000	0.000
ISCA2	3.222	4.001	0.779	0.000	0.000
ISCU	5.556	5.900	0.344	0.000	0.000
ISG15	6.741	6.950	0.209	0.008	0.009
ISG20	2.796	3.126	0.330	0.000	0.000
ISG20L2	3.015	4.166	1.151	0.000	0.000
ISL2	0.105	0.549	0.445	0.000	0.000
ISLR	3.302	2.396	-0.906	0.000	0.000
ISLR2	0.181	0.336	0.155	0.000	0.000
ISM1	1.911	2.124	0.213	0.003	0.003
ISOC1	6.250	6.039	-0.211	0.000	0.000
ISOC2	6.134	6.191	0.056	0.103	0.111
ISPD	1.184	1.204	0.021	0.481	0.497
ISX	0.196	1.601	1.405	0.000	0.000
ISY1	2.969	3.705	0.736	0.000	0.000
ISY1-RAB43	2.001	2.465	0.464	0.000	0.000
ISYNA1	3.696	3.761	0.065	0.383	0.399

ITCH	4.609	4.747	0.137	0.000	0.000
ITFG1	4.417	4.495	0.078	0.039	0.043
ITFG2	2.819	3.232	0.413	0.000	0.000
ITGA1	3.914	4.066	0.153	0.000	0.000
ITGA10	0.868	1.004	0.136	0.000	0.000
ITGA11	0.518	1.028	0.510	0.000	0.000
ITGA2	0.995	1.867	0.872	0.000	0.000
ITGA2B	0.261	0.282	0.022	0.318	0.333
ITGA3	1.738	2.086	0.348	0.000	0.000
ITGA4	1.798	1.695	-0.103	0.047	0.052
ITGA5	4.291	5.277	0.985	0.000	0.000
ITGA6	3.032	4.808	1.777	0.000	0.000
ITGA7	2.711	3.430	0.719	0.000	0.000
ITGA8	0.526	0.629	0.102	0.001	0.001
ITGA9	2.553	1.701	-0.852	0.000	0.000
ITGAD	1.511	0.903	-0.608	0.000	0.000
ITGAE	2.042	2.632	0.590	0.000	0.000
ITGAL	3.512	3.755	0.243	0.000	0.000
ITGAM	1.936	2.360	0.424	0.000	0.000
ITGAV	3.559	4.545	0.986	0.000	0.000
ITGAX	2.525	2.915	0.390	0.000	0.000
ITGB1	6.490	7.326	0.836	0.000	0.000
ITGB1BP1	3.025	4.143	1.118	0.000	0.000
ITGB1BP2	0.834	1.173	0.339	0.000	0.000
ITGB2	4.092	4.134	0.042	0.552	0.567
ITGB3BP	1.769	2.911	1.142	0.000	0.000
ITGB4	1.697	2.531	0.834	0.000	0.000
ITGB5	5.233	6.056	0.823	0.000	0.000
ITGB6	0.240	0.447	0.206	0.000	0.000
ITGB7	1.969	2.111	0.143	0.004	0.005
ITGB8	0.805	0.526	-0.278	0.000	0.000
ITGBL1	1.902	1.783	-0.119	0.132	0.141
ITIH1	10.249	9.264	-0.985	0.000	0.000
ITIH2	10.188	10.111	-0.077	0.337	0.353
ITIH3	9.299	9.056	-0.243	0.006	0.007
ITIH4	8.079	6.824	-1.254	0.000	0.000
ITIH5	1.042	0.982	-0.060	0.425	0.441
ITK	1.475	1.129	-0.346	0.000	0.000
ITLN1	2.527	0.674	-1.853	0.000	0.000
ITLN2	0.465	0.987	0.522	0.000	0.000
ITM2A	2.920	3.267	0.348	0.000	0.000
ITM2B	7.058	7.358	0.300	0.000	0.000
ITM2C	4.907	5.368	0.461	0.000	0.000
ITPA	4.888	5.809	0.922	0.000	0.000
ITPK1	4.375	5.285	0.910	0.000	0.000
ITPKA	1.215	3.001	1.786	0.000	0.000
ITPKB	1.976	2.188	0.212	0.000	0.000
ITPKC	3.865	4.552	0.687	0.000	0.000
ITPR1	1.731	2.009	0.278	0.000	0.000
ITPR2	4.267	4.498	0.231	0.000	0.000
ITPR3	1.476	1.912	0.436	0.000	0.000
ITPRIP	2.757	2.713	-0.044	0.356	0.372

ITPRIPL1	0.527	0.590	0.063	0.022	0.025
ITPRIPL2	2.394	3.029	0.635	0.000	0.000
ITSN1	2.957	3.026	0.069	0.062	0.068
ITSN2	3.328	3.705	0.377	0.000	0.000
IVD	6.476	5.913	-0.563	0.000	0.000
IVNS1ABP	4.453	5.249	0.796	0.000	0.000
IWS1	4.413	4.983	0.570	0.000	0.000
IYD	3.309	2.383	-0.926	0.000	0.000
IZUMO1	0.101	0.259	0.157	0.000	0.000
IZUMO4	2.096	1.714	-0.383	0.000	0.000
JAG2	1.433	2.520	1.087	0.000	0.000
JAGN1	5.603	5.835	0.232	0.000	0.000
JAK1	5.345	5.665	0.320	0.000	0.000
JAK2	2.266	2.278	0.013	0.771	0.781
JAK3	1.995	1.987	-0.008	0.899	0.902
JAKMIP1	0.516	0.557	0.042	0.188	0.200
JAKMIP2	0.567	0.712	0.145	0.000	0.000
JAKMIP3	0.194	0.325	0.131	0.000	0.000
JAM2	1.522	1.581	0.059	0.183	0.194
JAM3	1.935	2.367	0.433	0.000	0.000
JARID2	2.180	2.869	0.689	0.000	0.000
JAZF1	3.182	3.861	0.679	0.000	0.000
JDP2	3.397	2.695	-0.702	0.000	0.000
JKAMP	3.752	4.441	0.690	0.000	0.000
JMJD1C	4.001	3.971	-0.030	0.497	0.512
JMJD6	3.237	3.928	0.691	0.000	0.000
JMJD7	1.719	1.822	0.103	0.000	0.000
JMJD7-PLA2G	1.618	1.826	0.209	0.000	0.000
JMY	2.392	2.901	0.509	0.000	0.000
JOSD1	4.297	4.857	0.560	0.000	0.000
JOSD2	4.072	4.991	0.919	0.000	0.000
JPH1	0.322	0.822	0.500	0.000	0.000
JPH2	0.229	0.389	0.160	0.000	0.000
JPH4	0.353	0.339	-0.014	0.406	0.422
JRK	1.040	2.006	0.966	0.000	0.000
JRKL	1.928	2.914	0.987	0.000	0.000
JTB	7.325	8.222	0.897	0.000	0.000
JUN	7.319	6.665	-0.654	0.000	0.000
JUNB	7.658	6.719	-0.939	0.000	0.000
JUND	7.831	7.655	-0.176	0.001	0.001
JUP	5.520	6.147	0.627	0.000	0.000
KAAG1	0.938	1.277	0.338	0.000	0.000
KALRN	2.285	2.176	-0.109	0.004	0.005
KANK1	4.728	4.796	0.068	0.162	0.173
KANK2	4.309	4.885	0.575	0.000	0.000
KANK3	2.341	2.404	0.062	0.135	0.144
KANK4	1.041	0.673	-0.367	0.000	0.000
KARS	5.428	6.004	0.576	0.000	0.000
KAT2A	4.943	5.847	0.905	0.000	0.000
KAT2B	4.201	4.137	-0.065	0.152	0.162
KATNA1	2.981	3.653	0.672	0.000	0.000
KATNAL1	1.710	1.929	0.218	0.000	0.000

KATNAL2	1.704	2.040	0.336	0.000	0.000
KATNB1	2.446	3.420	0.974	0.000	0.000
KAZALD1	0.811	1.419	0.608	0.000	0.000
KAZN	1.475	0.701	-0.774	0.000	0.000
KBTBD11	2.594	1.215	-1.379	0.000	0.000
KBTBD2	3.796	4.405	0.610	0.000	0.000
KBTBD3	1.535	1.837	0.302	0.000	0.000
KBTBD6	1.977	2.410	0.433	0.000	0.000
KBTBD7	2.092	2.276	0.184	0.000	0.000
KBTBD8	0.814	0.988	0.174	0.000	0.000
KCMF1	3.442	4.206	0.765	0.000	0.000
KCNA3	0.859	0.613	-0.246	0.000	0.000
KCNA5	0.349	0.423	0.074	0.005	0.006
KCNAB1	0.899	0.869	-0.031	0.195	0.207
KCNAB2	2.765	2.785	0.020	0.782	0.791
KCNAB3	0.690	0.553	-0.137	0.000	0.000
KCNB1	0.684	0.805	0.122	0.019	0.021
KCNC3	1.132	1.497	0.364	0.000	0.000
KCNC4	0.413	0.611	0.198	0.000	0.000
KCND1	0.695	0.818	0.123	0.000	0.000
KCND3	2.266	1.321	-0.945	0.000	0.000
KCNE1	0.552	0.266	-0.286	0.000	0.000
KCNE2	0.934	0.880	-0.054	0.056	0.061
KCNE3	1.647	2.747	1.100	0.000	0.000
KCNE4	1.487	1.611	0.124	0.052	0.057
KCNF1	0.251	0.658	0.407	0.000	0.000
KCNG1	0.184	0.298	0.114	0.000	0.000
KCNG2	0.131	0.289	0.159	0.000	0.000
KCNH2	0.161	0.477	0.316	0.000	0.000
KCNH3	0.460	0.470	0.010	0.798	0.807
KCNH4	0.178	0.408	0.230	0.000	0.000
KCNH8	0.352	0.386	0.034	0.324	0.340
KCNIP1	0.204	0.162	-0.042	0.026	0.029
KCNIP2	0.747	1.058	0.311	0.000	0.000
KCNIP3	0.524	0.909	0.385	0.000	0.000
KCNJ10	1.092	0.636	-0.456	0.000	0.000
KCNJ11	0.724	1.408	0.684	0.000	0.000
KCNJ12	0.253	0.257	0.004	0.799	0.808
KCNJ13	0.229	0.163	-0.066	0.000	0.000
KCNJ14	0.573	0.835	0.262	0.000	0.000
KCNJ15	1.086	0.618	-0.468	0.000	0.000
KCNJ16	1.185	0.470	-0.716	0.000	0.000
KCNJ2	1.006	1.283	0.278	0.000	0.000
KCNJ3	0.952	1.108	0.155	0.012	0.014
KCNJ4	0.929	1.649	0.719	0.000	0.000
KCNJ5	0.535	1.152	0.617	0.000	0.000
KCNJ8	5.413	5.002	-0.410	0.000	0.000
KCNK1	3.155	3.288	0.132	0.085	0.092
KCNK13	0.657	0.823	0.166	0.000	0.000
KCNK15	0.378	0.399	0.022	0.584	0.598
KCNK17	1.616	0.723	-0.893	0.000	0.000
KCNK3	0.341	0.264	-0.077	0.001	0.001

KCNK5	3.447	2.556	-0.891	0.000	0.000
KCNK6	1.637	1.386	-0.251	0.000	0.000
KCNK7	0.539	0.627	0.088	0.001	0.001
KCNMA1	1.752	1.310	-0.442	0.000	0.000
KCNMB1	0.465	0.609	0.145	0.000	0.000
KCNMB2	0.253	0.256	0.002	0.909	0.913
KCNMB3	0.623	1.089	0.466	0.000	0.000
KCNMB4	0.691	0.824	0.133	0.001	0.001
KCNN1	0.090	0.258	0.168	0.000	0.000
KCNN2	2.591	0.687	-1.904	0.000	0.000
KCNN3	0.407	0.851	0.444	0.000	0.000
KCNN4	0.957	1.227	0.270	0.000	0.000
KCNQ1	1.629	1.855	0.226	0.000	0.000
KCNQ4	0.375	0.599	0.224	0.000	0.000
KCNRG	0.653	0.746	0.092	0.000	0.000
KCNS3	2.157	2.019	-0.138	0.047	0.052
KCNT2	1.936	2.086	0.150	0.008	0.009
KCP	0.091	0.525	0.434	0.000	0.000
KCTD1	0.964	1.569	0.605	0.000	0.000
KCTD10	2.554	3.254	0.700	0.000	0.000
KCTD11	2.444	2.617	0.173	0.000	0.000
KCTD12	3.363	3.353	-0.011	0.872	0.877
KCTD13	2.378	2.916	0.538	0.000	0.000
KCTD14	2.100	2.398	0.298	0.000	0.000
KCTD15	2.178	2.370	0.193	0.001	0.001
KCTD17	2.002	3.070	1.068	0.000	0.000
KCTD18	2.597	3.134	0.536	0.000	0.000
KCTD2	2.915	3.795	0.881	0.000	0.000
KCTD20	4.161	4.987	0.827	0.000	0.000
KCTD21	3.426	3.883	0.457	0.000	0.000
KCTD3	5.440	6.115	0.675	0.000	0.000
KCTD5	3.484	4.078	0.594	0.000	0.000
KCTD6	2.952	3.858	0.906	0.000	0.000
KCTD7	1.275	2.183	0.908	0.000	0.000
KCTD9	2.748	3.180	0.432	0.000	0.000
KDELC1	2.564	3.509	0.945	0.000	0.000
KDELC2	3.177	3.286	0.109	0.034	0.038
KDELR1	6.565	7.441	0.876	0.000	0.000
KDELR2	7.121	7.738	0.617	0.000	0.000
KDELR3	3.446	4.562	1.116	0.000	0.000
KDM1A	3.778	4.532	0.753	0.000	0.000
KDM1B	2.494	3.154	0.660	0.000	0.000
KDM2A	4.287	4.856	0.569	0.000	0.000
KDM2B	2.364	2.751	0.387	0.000	0.000
KDM3A	2.701	3.351	0.650	0.000	0.000
KDM3B	3.443	4.198	0.755	0.000	0.000
KDM4A	3.444	4.184	0.740	0.000	0.000
KDM4B	2.629	2.968	0.339	0.000	0.000
KDM4C	2.761	3.286	0.525	0.000	0.000
KDM4D	0.383	0.561	0.178	0.000	0.000
KDM5A	3.088	3.509	0.421	0.000	0.000
KDM5B	2.156	3.177	1.021	0.000	0.000

KDM5C	3.775	4.478	0.704	0.000	0.000
KDM5D	2.348	2.473	0.125	0.222	0.235
KDM6A	2.883	3.318	0.434	0.000	0.000
KDM6B	3.705	3.287	-0.419	0.000	0.000
KDR	3.479	3.499	0.020	0.744	0.754
KDSR	4.274	4.304	0.030	0.341	0.356
KEAP1	5.041	5.666	0.625	0.000	0.000
KEL	0.520	0.732	0.211	0.000	0.000
KHDC1	0.121	0.256	0.135	0.000	0.000
KHDRBS1	5.348	6.100	0.752	0.000	0.000
KHDRBS3	2.052	2.767	0.715	0.000	0.000
KHK	7.930	7.214	-0.717	0.000	0.000
KHNYN	3.527	4.155	0.628	0.000	0.000
KIAA0040	3.577	3.316	-0.261	0.000	0.000
KIAA0100	3.524	4.651	1.127	0.000	0.000
KIAA0232	3.294	4.058	0.764	0.000	0.000
KIAA0319L	4.096	4.664	0.569	0.000	0.000
KIAA0355	2.695	3.182	0.487	0.000	0.000
KIAA0391	2.455	2.933	0.479	0.000	0.000
KIAA0408	0.304	0.315	0.011	0.533	0.548
KIAA0513	1.234	1.754	0.520	0.000	0.000
KIAA0556	1.771	2.406	0.636	0.000	0.000
KIAA0586	1.726	2.085	0.359	0.000	0.000
KIAA0753	1.812	2.095	0.283	0.000	0.000
KIAA0825	0.312	0.322	0.010	0.426	0.442
KIAA0895	1.703	2.199	0.496	0.000	0.000
KIAA0895L	2.452	2.754	0.302	0.000	0.000
KIAA0930	3.950	4.426	0.476	0.000	0.000
KIAA1107	1.105	1.426	0.322	0.000	0.000
KIAA1109	2.988	3.004	0.016	0.688	0.700
KIAA1143	2.907	3.588	0.681	0.000	0.000
KIAA1147	3.633	4.227	0.594	0.000	0.000
KIAA1191	5.504	5.867	0.363	0.000	0.000
KIAA1211	0.636	0.645	0.009	0.836	0.843
KIAA1217	3.336	3.289	-0.047	0.276	0.291
KIAA1324	0.277	0.628	0.351	0.000	0.000
KIAA1324L	0.878	1.057	0.179	0.000	0.000
KIAA1328	0.730	1.124	0.394	0.000	0.000
KIAA1522	3.169	4.646	1.476	0.000	0.000
KIAA1549	0.503	0.967	0.464	0.000	0.000
KIAA1586	2.111	2.852	0.741	0.000	0.000
KIAA1614	0.282	0.643	0.361	0.000	0.000
KIAA1671	3.590	3.637	0.047	0.256	0.270
KIAA1755	0.703	0.872	0.170	0.000	0.000
KIAA1841	1.275	1.988	0.713	0.000	0.000
KIAA1958	1.052	1.438	0.386	0.000	0.000
KIAA2013	5.506	5.831	0.325	0.000	0.000
KIAA2026	2.806	2.949	0.143	0.000	0.001
KIDINS220	3.178	3.711	0.533	0.000	0.000
KIF11	0.969	2.505	1.536	0.000	0.000
KIF12	4.331	4.173	-0.158	0.064	0.070
KIF13A	2.895	3.552	0.657	0.000	0.000

KIF13B	2.975	3.095	0.120	0.004	0.004
KIF14	0.403	1.667	1.264	0.000	0.000
KIF15	0.336	1.710	1.375	0.000	0.000
KIF16B	2.382	2.821	0.439	0.000	0.000
KIF17	1.248	1.174	-0.074	0.036	0.040
KIF18A	0.319	1.501	1.182	0.000	0.000
KIF18B	0.394	1.930	1.536	0.000	0.000
KIF19	0.903	0.307	-0.596	0.000	0.000
KIF1A	0.320	0.457	0.136	0.005	0.005
KIF1B	2.478	3.047	0.569	0.000	0.000
KIF1C	4.975	5.498	0.524	0.000	0.000
KIF20A	0.668	2.873	2.205	0.000	0.000
KIF20B	1.205	2.136	0.930	0.000	0.000
KIF21A	3.287	3.871	0.584	0.000	0.000
KIF21B	1.164	1.718	0.554	0.000	0.000
KIF22	4.277	4.892	0.615	0.000	0.000
KIF23	0.467	2.211	1.744	0.000	0.000
KIF24	0.605	1.221	0.616	0.000	0.000
KIF25	0.493	0.510	0.017	0.624	0.638
KIF26A	0.816	0.769	-0.047	0.148	0.158
KIF26B	0.297	0.571	0.274	0.000	0.000
KIF27	1.007	1.111	0.104	0.000	0.000
KIF2A	1.899	2.781	0.882	0.000	0.000
KIF2C	0.690	2.831	2.141	0.000	0.000
KIF3A	1.226	1.978	0.752	0.000	0.000
KIF3B	3.958	4.770	0.812	0.000	0.000
KIF3C	0.899	1.229	0.330	0.000	0.000
KIF4A	0.572	2.649	2.078	0.000	0.000
KIF5B	4.574	5.425	0.851	0.000	0.000
KIF5C	0.359	0.541	0.181	0.000	0.000
KIF6	0.189	0.334	0.144	0.000	0.000
KIF7	0.872	1.508	0.636	0.000	0.000
KIF9	1.440	1.869	0.429	0.000	0.000
KIFAP3	3.282	4.181	0.899	0.000	0.000
KIFC1	0.905	3.355	2.450	0.000	0.000
KIFC2	2.973	4.110	1.137	0.000	0.000
KIFC3	3.990	4.272	0.282	0.000	0.000
KIN	2.272	2.946	0.674	0.000	0.000
KIR2DL3	0.207	0.117	-0.089	0.000	0.000
KIR2DL4	0.303	0.314	0.011	0.644	0.657
KISS1	1.619	2.636	1.017	0.000	0.000
KIT	0.851	1.003	0.152	0.000	0.000
KITLG	1.402	2.263	0.861	0.000	0.000
KL	0.338	0.545	0.207	0.000	0.000
KLB	3.704	4.615	0.910	0.000	0.000
KLC1	2.494	2.827	0.333	0.000	0.000
KLC2	2.020	3.084	1.063	0.000	0.000
KLC3	0.389	0.913	0.525	0.000	0.000
KLC4	5.532	5.394	-0.139	0.001	0.001
KLF1	0.090	0.159	0.069	0.000	0.000
KLF10	5.236	5.068	-0.169	0.003	0.004
KLF11	4.420	4.271	-0.149	0.002	0.002

KLF12	2.859	2.887	0.029	0.521	0.536
KLF13	3.540	4.578	1.037	0.000	0.000
KLF15	5.587	5.963	0.376	0.000	0.000
KLF16	3.728	4.064	0.336	0.000	0.000
KLF2	4.198	3.779	-0.419	0.000	0.000
KLF3	4.483	4.825	0.342	0.000	0.000
KLF4	3.702	3.063	-0.639	0.000	0.000
KLF5	2.003	2.349	0.346	0.000	0.000
KLF6	6.430	5.922	-0.508	0.000	0.000
KLF7	1.495	1.461	-0.034	0.444	0.460
KLF8	0.702	0.564	-0.138	0.000	0.000
KLF9	5.690	5.340	-0.350	0.000	0.000
KLHDC1	1.882	1.745	-0.137	0.000	0.000
KLHDC10	3.859	4.568	0.709	0.000	0.000
KLHDC2	4.689	4.978	0.290	0.000	0.000
KLHDC3	5.961	6.951	0.989	0.000	0.000
KLHDC4	2.525	2.842	0.316	0.000	0.000
KLHDC7A	2.028	1.915	-0.113	0.043	0.048
KLHDC7B	0.742	1.201	0.459	0.000	0.000
KLHDC8A	0.116	0.377	0.260	0.000	0.000
KLHDC8B	3.736	4.563	0.827	0.000	0.000
KLHDC9	2.643	3.230	0.588	0.000	0.000
KLHL10	0.120	0.157	0.037	0.000	0.000
KLHL11	0.837	1.050	0.213	0.000	0.000
KLHL12	3.681	4.894	1.212	0.000	0.000
KLHL13	1.135	1.677	0.542	0.000	0.000
KLHL15	3.024	2.747	-0.277	0.000	0.000
KLHL17	2.050	2.724	0.674	0.000	0.000
KLHL18	2.193	2.656	0.463	0.000	0.000
KLHL2	4.324	3.927	-0.397	0.000	0.000
KLHL20	3.276	3.670	0.394	0.000	0.000
KLHL21	3.338	4.182	0.844	0.000	0.000
KLHL22	2.858	3.579	0.720	0.000	0.000
KLHL23	2.000	3.282	1.282	0.000	0.000
KLHL24	3.501	3.949	0.447	0.000	0.000
KLHL25	2.260	2.653	0.393	0.000	0.000
KLHL26	1.776	2.098	0.321	0.000	0.000
KLHL29	1.183	1.760	0.577	0.000	0.000
KLHL3	1.276	1.193	-0.083	0.086	0.094
KLHL30	0.187	0.394	0.207	0.000	0.000
KLHL31	0.524	0.937	0.413	0.000	0.000
KLHL32	0.491	0.542	0.051	0.017	0.019
KLHL36	2.943	2.718	-0.225	0.000	0.000
KLHL4	0.374	0.371	-0.004	0.884	0.888
KLHL5	4.115	5.077	0.963	0.000	0.000
KLHL6	1.180	1.197	0.017	0.709	0.720
KLHL7	2.301	3.216	0.915	0.000	0.000
KLHL8	2.521	2.644	0.123	0.000	0.001
KLHL9	3.200	3.591	0.391	0.000	0.000
KLK14	0.325	0.414	0.089	0.000	0.000
KLKB1	7.392	5.767	-1.624	0.000	0.000
KLRB1	3.201	2.486	-0.716	0.000	0.000

KLRC1	0.533	0.414	-0.119	0.000	0.000
KLRC2	0.376	0.302	-0.073	0.002	0.003
KLRC3	0.519	0.367	-0.152	0.000	0.000
KLRC4	0.687	0.524	-0.163	0.000	0.000
KLRC4-KLRK	1.417	1.116	-0.301	0.000	0.000
KLRD1	0.744	0.452	-0.291	0.000	0.000
KLRF1	1.708	0.929	-0.779	0.000	0.000
KLRG1	1.488	1.256	-0.232	0.000	0.000
KLRK1	2.024	1.337	-0.686	0.000	0.000
KMO	4.715	3.702	-1.014	0.000	0.000
KNDC1	0.986	1.160	0.174	0.019	0.021
KNG1	10.591	10.133	-0.458	0.000	0.000
KNTC1	1.484	2.852	1.369	0.000	0.000
KPNA1	3.921	4.310	0.389	0.000	0.000
KPNA2	3.532	5.620	2.088	0.000	0.000
KPNA3	3.962	4.491	0.530	0.000	0.000
KPNA4	3.527	4.227	0.699	0.000	0.000
KPNA5	1.284	1.524	0.240	0.000	0.000
KPNA6	3.659	4.105	0.446	0.000	0.000
KPNA7	0.519	1.323	0.804	0.000	0.000
KPNB1	5.051	5.997	0.946	0.000	0.000
KPTN	2.977	3.778	0.801	0.000	0.000
KRAS	3.150	3.460	0.310	0.000	0.000
KRBA1	1.091	1.708	0.616	0.000	0.000
KRBA2	0.409	0.637	0.229	0.000	0.000
KRBOX1	0.369	0.443	0.074	0.010	0.011
KRCC1	5.196	5.303	0.108	0.004	0.005
KREMEN1	1.050	1.287	0.237	0.000	0.000
KREMEN2	0.139	0.383	0.244	0.000	0.000
KRI1	3.587	4.311	0.724	0.000	0.000
KRIT1	3.305	3.831	0.525	0.000	0.000
KRR1	3.090	3.405	0.315	0.000	0.000
KRT10	3.804	4.443	0.639	0.000	0.000
KRT17	0.514	1.171	0.657	0.000	0.000
KRT18	8.504	8.664	0.160	0.001	0.002
KRT19	3.494	2.039	-1.455	0.000	0.000
KRT222	1.441	1.524	0.084	0.167	0.178
KRT23	2.124	3.109	0.985	0.000	0.000
KRT27	0.168	0.131	-0.037	0.001	0.002
KRT7	4.363	2.978	-1.386	0.000	0.000
KRT8	8.254	8.401	0.147	0.006	0.007
KRT80	1.165	1.022	-0.143	0.045	0.049
KRT81	0.291	0.421	0.130	0.001	0.001
KRT86	0.387	0.782	0.395	0.000	0.000
KRTAP5-10	0.201	0.318	0.117	0.000	0.000
KRTAP5-2	0.260	0.350	0.090	0.002	0.002
KRTAP5-6	0.966	1.587	0.621	0.000	0.000
KRTAP5-7	0.105	0.316	0.211	0.000	0.000
KRTAP5-8	0.139	0.180	0.041	0.001	0.001
KRTAP5-9	0.440	0.493	0.053	0.034	0.037
KRTCAP2	4.426	5.797	1.372	0.000	0.000
KRTCAP3	3.674	2.627	-1.047	0.000	0.000

KSR1	1.290	1.690	0.400	0.000	0.000
KTI12	2.810	3.573	0.763	0.000	0.000
KTN1	4.782	5.242	0.460	0.000	0.000
KYNU	3.348	2.983	-0.365	0.000	0.000
L1CAM	0.403	0.305	-0.098	0.009	0.010
L2HGDH	2.382	2.529	0.147	0.000	0.000
L3MBTL1	0.679	1.187	0.508	0.000	0.000
L3MBTL2	3.262	3.957	0.695	0.000	0.000
L3MBTL3	1.837	1.958	0.121	0.004	0.005
L3MBTL4	2.410	2.504	0.094	0.010	0.011
LACTB	4.457	4.804	0.348	0.000	0.000
LACTB2	5.199	5.763	0.564	0.000	0.000
LAD1	4.303	4.718	0.415	0.000	0.000
LAG3	2.703	2.021	-0.682	0.000	0.000
LAGE3	4.457	5.827	1.371	0.000	0.000
LAIR1	1.898	2.454	0.557	0.000	0.000
LAIR2	0.325	0.716	0.391	0.000	0.000
LAMA1	0.255	0.267	0.012	0.720	0.731
LAMA2	2.104	1.566	-0.538	0.000	0.000
LAMA3	1.064	2.470	1.407	0.000	0.000
LAMA4	1.743	2.972	1.229	0.000	0.000
LAMA5	3.739	4.235	0.497	0.000	0.000
LAMB1	4.100	4.817	0.717	0.000	0.000
LAMB2	6.229	6.387	0.158	0.001	0.001
LAMB3	2.362	3.204	0.842	0.000	0.000
LAMC1	3.708	5.448	1.739	0.000	0.000
LAMC2	1.149	0.956	-0.193	0.014	0.016
LAMC3	3.004	2.290	-0.714	0.000	0.000
LAMP1	6.919	7.388	0.469	0.000	0.000
LAMP2	6.659	7.200	0.542	0.000	0.000
LAMP3	1.175	1.555	0.380	0.000	0.000
LAMTOR1	5.131	6.092	0.961	0.000	0.000
LAMTOR2	6.387	7.334	0.947	0.000	0.000
LAMTOR3	3.985	4.356	0.371	0.000	0.000
LANCL1	3.596	4.683	1.087	0.000	0.000
LANCL2	3.110	3.678	0.568	0.000	0.000
LAP3	6.652	6.435	-0.217	0.000	0.000
LAPTM4A	7.881	8.335	0.454	0.000	0.000
LAPTM4B	4.239	6.107	1.868	0.000	0.000
LAPTM5	5.661	5.875	0.214	0.003	0.003
LARP1	4.609	5.753	1.143	0.000	0.000
LARP1B	3.844	3.341	-0.503	0.000	0.000
LARP4	4.706	4.902	0.196	0.000	0.000
LARP4B	2.795	3.656	0.861	0.000	0.000
LARP6	1.458	1.620	0.162	0.011	0.013
LARP7	4.779	4.781	0.002	0.943	0.946
LARS	3.734	4.865	1.131	0.000	0.000
LARS2	3.023	3.376	0.353	0.000	0.000
LAS1L	3.627	4.471	0.843	0.000	0.000
LASP1	5.475	6.588	1.113	0.000	0.000
LAT	1.705	1.733	0.028	0.481	0.497
LAT2	2.434	2.425	-0.008	0.870	0.875

LATS1	2.155	2.477	0.322	0.000	0.000
LATS2	3.057	3.068	0.011	0.797	0.806
LAX1	0.952	0.804	-0.148	0.001	0.001
LAYN	1.114	1.381	0.268	0.000	0.000
LBH	3.210	3.466	0.256	0.000	0.000
LBP	8.931	8.579	-0.353	0.000	0.000
LBR	4.615	5.645	1.030	0.000	0.000
LBX2	2.215	2.728	0.513	0.000	0.000
LCA5	0.757	0.739	-0.018	0.563	0.578
LCA5L	0.499	0.645	0.146	0.000	0.000
LCAT	7.242	4.889	-2.353	0.000	0.000
LCK	2.518	2.244	-0.273	0.000	0.000
LCLAT1	2.094	2.738	0.644	0.000	0.000
LCMT1	3.148	4.024	0.876	0.000	0.000
LCMT2	1.803	2.202	0.399	0.000	0.000
LCN10	0.389	0.281	-0.108	0.000	0.000
LCN12	3.144	3.107	-0.037	0.526	0.540
LCN2	3.022	5.552	2.530	0.000	0.000
LCN6	0.347	0.192	-0.154	0.000	0.000
LCNL1	0.276	0.317	0.041	0.246	0.260
LCOR	2.215	2.641	0.425	0.000	0.000
LCORL	1.328	1.926	0.598	0.000	0.000
LCP1	5.084	5.157	0.073	0.295	0.310
LCP2	3.240	2.818	-0.422	0.000	0.000
LCTL	0.245	0.595	0.350	0.000	0.000
LDB1	3.883	4.468	0.585	0.000	0.000
LDB2	3.179	2.885	-0.295	0.000	0.000
LDB3	0.248	0.349	0.101	0.000	0.000
LDHA	8.011	7.998	-0.014	0.753	0.764
LDHAL6B	0.287	0.236	-0.050	0.001	0.001
LDHB	4.883	4.841	-0.042	0.541	0.555
LDHC	0.555	0.763	0.208	0.000	0.000
LDHD	6.933	5.774	-1.160	0.000	0.000
LDLR	5.617	4.957	-0.660	0.000	0.000
LDLRAD1	0.247	1.062	0.815	0.000	0.000
LDLRAD2	1.978	2.260	0.281	0.000	0.000
LDLRAD3	1.145	1.375	0.231	0.000	0.000
LDLRAP1	3.757	4.024	0.267	0.000	0.000
LDOC1	2.710	2.349	-0.360	0.000	0.000
LEAP2	7.162	6.339	-0.823	0.000	0.000
LECT2	7.369	5.599	-1.770	0.000	0.000
LEF1	0.888	2.024	1.136	0.000	0.000
LEFTY1	0.872	0.973	0.101	0.096	0.103
LEKR1	0.312	0.529	0.217	0.000	0.000
LEMD2	3.521	4.484	0.963	0.000	0.000
LEMD3	2.963	3.334	0.372	0.000	0.000
LENG1	3.413	3.926	0.513	0.000	0.000
LEO1	4.021	4.886	0.865	0.000	0.000
LEPR	5.550	4.680	-0.871	0.000	0.000
LEPROT	4.989	5.581	0.592	0.000	0.000
LEPROTL1	3.901	4.176	0.275	0.000	0.000
LETM1	3.251	3.988	0.737	0.000	0.000

LETM2	0.303	0.538	0.235	0.000	0.000
LETMD1	4.374	4.878	0.504	0.000	0.000
LFNG	1.809	2.361	0.552	0.000	0.000
LGALS1	7.342	7.881	0.539	0.000	0.000
LGALS12	0.125	0.176	0.051	0.033	0.037
LGALS2	2.809	2.747	-0.062	0.461	0.477
LGALS3	4.470	5.482	1.012	0.000	0.000
LGALS3BP	7.394	7.327	-0.067	0.474	0.489
LGALS4	6.214	6.938	0.724	0.000	0.000
LGALS8	4.275	5.026	0.751	0.000	0.000
LGALS9	3.488	3.941	0.452	0.000	0.000
LGALS9C	0.269	0.359	0.090	0.005	0.005
LGI2	0.442	0.749	0.307	0.000	0.000
LGI4	2.123	1.859	-0.264	0.000	0.000
LGMN	6.106	6.266	0.161	0.001	0.001
LGR4	5.035	5.169	0.133	0.009	0.011
LGR5	0.875	1.710	0.835	0.000	0.000
LGR6	1.298	1.060	-0.238	0.000	0.000
LGSN	1.654	1.012	-0.642	0.000	0.000
LHB	0.183	0.402	0.219	0.000	0.000
LHFPL2	1.793	2.536	0.743	0.000	0.000
LHPP	4.775	4.529	-0.246	0.000	0.000
LHX2	1.962	0.902	-1.060	0.000	0.000
LHX4	0.272	0.650	0.378	0.000	0.000
LHX6	0.414	0.557	0.144	0.000	0.000
LHX9	0.234	0.478	0.244	0.000	0.000
LIAS	3.505	3.897	0.392	0.000	0.000
LIF	2.148	1.986	-0.162	0.061	0.067
LIFR	3.747	1.920	-1.826	0.000	0.000
LIG1	2.884	4.029	1.146	0.000	0.000
LIG3	1.822	2.732	0.910	0.000	0.000
LIG4	3.204	3.207	0.003	0.943	0.945
LILRA1	0.897	0.556	-0.341	0.000	0.000
LILRA2	1.245	0.703	-0.542	0.000	0.000
LILRA4	0.563	0.445	-0.118	0.000	0.000
LILRA5	2.153	1.258	-0.895	0.000	0.000
LILRA6	1.197	1.076	-0.121	0.001	0.002
LILRB1	2.174	1.625	-0.549	0.000	0.000
LILRB2	2.787	2.265	-0.522	0.000	0.000
LILRB3	1.655	1.573	-0.082	0.058	0.064
LILRB4	1.932	2.065	0.133	0.036	0.040
LILRB5	3.035	1.794	-1.241	0.000	0.000
LIMA1	3.500	3.958	0.458	0.000	0.000
LIMCH1	2.302	2.065	-0.237	0.000	0.000
LIMD1	2.822	3.528	0.705	0.000	0.000
LIMD2	3.336	3.362	0.025	0.698	0.710
LIME1	4.843	4.246	-0.597	0.000	0.000
LIMK1	1.796	2.924	1.129	0.000	0.000
LIMK2	2.902	3.935	1.033	0.000	0.000
LIMS1	3.197	3.956	0.758	0.000	0.000
LIMS2	3.903	3.656	-0.248	0.000	0.000
LIN37	2.055	2.940	0.885	0.000	0.000

LIN52	2.357	2.923	0.566	0.000	0.000
LIN54	2.213	2.533	0.320	0.000	0.000
LIN7A	2.966	3.177	0.211	0.000	0.000
LIN7B	2.176	2.728	0.552	0.000	0.000
LIN7C	4.316	4.328	0.012	0.681	0.694
LIN9	0.932	2.113	1.182	0.000	0.000
LINGO1	0.664	1.571	0.907	0.000	0.000
LINGO3	0.300	0.235	-0.065	0.000	0.000
LINGO4	0.862	0.954	0.092	0.053	0.058
LIPA	5.621	6.137	0.515	0.000	0.000
LIPC	6.248	5.351	-0.898	0.000	0.000
LIPE	0.842	1.106	0.264	0.000	0.000
LIPG	3.953	3.260	-0.693	0.000	0.000
LIPH	0.644	0.837	0.193	0.001	0.001
LIPT1	2.500	2.928	0.428	0.000	0.000
LIPT2	1.624	2.041	0.416	0.000	0.000
LITAF	5.834	6.259	0.425	0.000	0.000
LLGL1	1.493	2.375	0.883	0.000	0.000
LLGL2	4.435	5.146	0.710	0.000	0.000
LLPH	2.635	3.327	0.693	0.000	0.000
LMAN1	6.244	6.173	-0.071	0.079	0.085
LMAN2	7.248	8.294	1.046	0.000	0.000
LMAN2L	3.846	4.429	0.583	0.000	0.000
LMBR1	2.726	3.260	0.534	0.000	0.000
LMBR1L	3.382	3.722	0.340	0.000	0.000
LMBRD1	5.883	6.027	0.144	0.000	0.000
LMBRD2	2.491	2.758	0.267	0.000	0.000
LMCD1	1.841	2.529	0.688	0.000	0.000
LMF1	2.775	2.960	0.185	0.000	0.000
LMF2	5.596	6.366	0.769	0.000	0.000
LMLN	1.287	1.569	0.282	0.000	0.000
LMNA	5.779	7.096	1.317	0.000	0.000
LMNB1	2.603	4.121	1.518	0.000	0.000
LMNB2	2.404	3.658	1.254	0.000	0.000
LMO2	3.064	3.336	0.272	0.000	0.000
LMO3	0.190	0.218	0.028	0.068	0.074
LMO4	3.757	3.920	0.163	0.000	0.001
LMO7	4.034	4.123	0.089	0.043	0.047
LMOD1	1.857	2.260	0.402	0.000	0.000
LMTK2	2.627	3.346	0.719	0.000	0.000
LMTK3	0.500	0.742	0.242	0.000	0.000
LNP1	2.188	1.974	-0.214	0.000	0.000
LNPEP	1.536	1.913	0.377	0.000	0.000
LNX1	1.809	2.120	0.311	0.000	0.000
LNX2	3.653	3.639	-0.014	0.731	0.741
LONP1	5.484	6.037	0.553	0.000	0.000
LONP2	5.451	4.945	-0.506	0.000	0.000
LONRF1	2.991	2.657	-0.334	0.000	0.000
LONRF2	1.041	1.002	-0.039	0.427	0.443
LONRF3	2.570	2.551	-0.019	0.680	0.692
LOX	1.183	2.155	0.971	0.000	0.000
LOXL1	1.529	1.883	0.354	0.000	0.000

LOXL2	1.545	2.845	1.300	0.000	0.000
LOXL3	1.491	1.806	0.315	0.000	0.000
LOXL4	2.722	3.416	0.694	0.000	0.000
LPA	4.508	2.709	-1.798	0.000	0.000
LPAR1	1.182	0.900	-0.282	0.000	0.000
LPAR2	1.912	2.261	0.349	0.000	0.000
LPAR5	1.247	1.159	-0.088	0.044	0.048
LPAR6	3.549	3.609	0.060	0.254	0.268
LPCAT1	2.910	4.013	1.103	0.000	0.000
LPCAT2	1.571	2.101	0.530	0.000	0.000
LPCAT3	4.687	5.022	0.335	0.000	0.000
LPCAT4	1.894	2.455	0.561	0.000	0.000
LPGAT1	4.624	5.852	1.228	0.000	0.000
LPIN1	3.157	3.567	0.410	0.000	0.000
LPIN2	6.192	5.976	-0.216	0.000	0.000
LPIN3	3.812	4.104	0.291	0.000	0.000
LPL	0.603	1.744	1.140	0.000	0.000
LPP	2.837	3.381	0.544	0.000	0.000
LPXN	3.606	3.699	0.093	0.034	0.037
LRAT	2.050	0.842	-1.208	0.000	0.000
LRBA	2.892	3.108	0.216	0.000	0.000
LRCH1	2.719	2.917	0.198	0.000	0.000
LRCH2	0.395	0.540	0.144	0.000	0.000
LRCH3	1.897	2.356	0.458	0.000	0.000
LRCH4	3.350	3.761	0.411	0.000	0.000
LRFN1	0.811	1.237	0.426	0.000	0.000
LRFN3	2.195	2.963	0.768	0.000	0.000
LRFN4	1.328	1.671	0.343	0.000	0.000
LRG1	8.571	7.593	-0.978	0.000	0.000
LRGUK	0.480	0.693	0.213	0.000	0.000
LRIF1	2.894	3.456	0.562	0.000	0.000
LRIG1	3.784	4.141	0.357	0.000	0.000
LRIG2	1.315	1.721	0.406	0.000	0.000
LRIG3	2.525	3.121	0.596	0.000	0.000
LRIT3	0.327	0.295	-0.032	0.042	0.046
LRMP	1.637	1.620	-0.017	0.678	0.690
LRP1	6.109	6.208	0.099	0.020	0.022
LRP10	4.165	4.994	0.829	0.000	0.000
LRP11	3.256	4.440	1.185	0.000	0.000
LRP12	0.703	1.334	0.630	0.000	0.000
LRP2	0.178	0.404	0.225	0.000	0.000
LRP2BP	0.625	0.654	0.029	0.146	0.156
LRP3	4.628	4.893	0.265	0.000	0.000
LRP4	0.379	1.049	0.670	0.000	0.000
LRP5	5.860	6.034	0.174	0.000	0.000
LRP5L	2.397	2.675	0.278	0.000	0.000
LRP6	3.912	3.985	0.073	0.100	0.108
LRP8	0.325	0.648	0.324	0.000	0.000
LRPAP1	5.040	5.681	0.641	0.000	0.000
LRPPRC	4.609	5.427	0.819	0.000	0.000
LRR1	2.015	2.850	0.836	0.000	0.000
LRRC1	1.192	2.707	1.515	0.000	0.000

LRRC10B	0.224	0.626	0.402	0.000	0.000
LRRC14	2.718	4.131	1.413	0.000	0.000
LRRC15	0.213	0.382	0.169	0.000	0.000
LRRC17	1.401	1.610	0.209	0.000	0.000
LRRC19	0.635	0.403	-0.231	0.000	0.000
LRRC2	2.290	2.495	0.205	0.000	0.000
LRRC20	3.721	4.848	1.127	0.000	0.000
LRRC23	1.604	1.946	0.342	0.000	0.000
LRRC25	2.891	2.571	-0.320	0.000	0.000
LRRC26	0.082	0.501	0.418	0.000	0.000
LRRC27	1.203	1.572	0.369	0.000	0.000
LRRC28	3.397	3.573	0.176	0.000	0.000
LRRC29	1.603	1.590	-0.012	0.691	0.703
LRRC3	2.870	2.486	-0.384	0.000	0.000
LRRC31	1.634	2.106	0.471	0.000	0.000
LRRC32	3.793	3.896	0.103	0.101	0.109
LRRC34	0.388	0.484	0.096	0.000	0.000
LRRC36	0.204	0.221	0.018	0.371	0.387
LRRC37A3	0.863	1.562	0.699	0.000	0.000
LRRC37B	1.120	1.684	0.564	0.000	0.000
LRRC39	1.202	1.527	0.325	0.000	0.000
LRRC3B	0.077	0.181	0.104	0.000	0.000
LRRC3C	0.144	0.181	0.037	0.008	0.009
LRRC4	1.027	0.584	-0.443	0.000	0.000
LRRC40	3.737	3.929	0.192	0.000	0.000
LRRC41	3.734	4.332	0.598	0.000	0.000
LRRC42	3.976	5.055	1.079	0.000	0.000
LRRC43	0.325	0.309	-0.016	0.520	0.535
LRRC45	3.454	4.622	1.168	0.000	0.000
LRRC46	0.769	1.208	0.438	0.000	0.000
LRRC47	4.706	4.979	0.273	0.000	0.000
LRRC49	0.406	0.480	0.073	0.002	0.002
LRRC4B	0.900	1.078	0.178	0.000	0.000
LRRC4C	0.604	0.342	-0.262	0.000	0.000
LRRC55	0.666	0.234	-0.432	0.000	0.000
LRRC56	1.054	1.265	0.211	0.000	0.000
LRRC57	1.587	2.333	0.746	0.000	0.000
LRRC58	3.063	3.853	0.790	0.000	0.000
LRRC59	5.147	6.235	1.088	0.000	0.000
LRRC6	0.981	1.013	0.032	0.436	0.452
LRRC61	3.903	4.643	0.740	0.000	0.000
LRRC66	0.363	0.416	0.054	0.063	0.068
LRRC69	0.440	1.021	0.581	0.000	0.000
LRRC7	0.286	0.232	-0.054	0.007	0.008
LRRC70	1.336	1.228	-0.108	0.001	0.001
LRRC71	0.109	0.163	0.054	0.000	0.000
LRRC8A	4.414	4.984	0.571	0.000	0.000
LRRC8B	1.762	1.998	0.236	0.000	0.000
LRRC8C	1.354	1.479	0.124	0.001	0.001
LRRC8D	3.616	4.572	0.956	0.000	0.000
LRRC8E	1.044	1.472	0.428	0.000	0.000
LRRCC1	1.831	2.321	0.490	0.000	0.000

LRRFIP1	4.215	4.407	0.192	0.000	0.000
LRRFIP2	3.983	4.371	0.387	0.000	0.000
LRRIQ3	0.269	0.365	0.095	0.000	0.000
LRRIQ4	0.158	0.181	0.023	0.096	0.103
LRRK1	0.944	0.849	-0.095	0.009	0.010
LRRK2	1.919	1.503	-0.415	0.000	0.000
LRRN1	0.648	0.329	-0.319	0.000	0.000
LRRN2	0.773	1.116	0.343	0.000	0.000
LRRN3	0.948	0.327	-0.622	0.000	0.000
LRRN4	0.390	0.315	-0.076	0.020	0.022
LRRN4CL	0.134	0.232	0.099	0.000	0.000
LRRTM2	0.196	0.192	-0.004	0.765	0.775
LRRTM4	0.273	0.273	0.000	0.995	0.995
LRSAM1	3.356	3.817	0.460	0.000	0.000
LRTOMT	2.229	2.602	0.372	0.000	0.000
LRWD1	2.919	3.489	0.570	0.000	0.000
LSAMP	0.277	0.328	0.051	0.112	0.121
LSG1	4.076	4.621	0.546	0.000	0.000
LSM10	4.967	5.508	0.540	0.000	0.000
LSM11	0.945	1.479	0.534	0.000	0.000
LSM12	3.350	4.139	0.789	0.000	0.000
LSM14A	4.971	5.757	0.786	0.000	0.000
LSM14B	3.790	4.763	0.973	0.000	0.000
LSM2	4.651	5.945	1.294	0.000	0.000
LSM3	4.103	4.731	0.628	0.000	0.000
LSM4	5.217	6.541	1.324	0.000	0.000
LSM5	4.031	4.632	0.601	0.000	0.000
LSM6	3.615	3.924	0.309	0.000	0.000
LSM7	5.089	5.913	0.824	0.000	0.000
LSR	6.887	6.969	0.082	0.208	0.221
LSS	4.232	5.078	0.845	0.000	0.000
LST1	3.913	3.344	-0.569	0.000	0.000
LTA	0.593	0.729	0.136	0.000	0.000
LTA4H	4.024	4.715	0.691	0.000	0.000
LTB	3.447	3.627	0.180	0.037	0.041
LTB4R	2.788	3.065	0.277	0.000	0.000
LTBP1	3.335	3.010	-0.325	0.000	0.000
LTBP2	2.015	2.751	0.735	0.000	0.000
LTBP3	4.841	5.005	0.164	0.003	0.003
LTBP4	4.681	3.718	-0.963	0.000	0.000
LTBR	5.520	5.957	0.438	0.000	0.000
LTC4S	1.346	1.295	-0.051	0.102	0.110
LTF	0.929	1.007	0.078	0.171	0.182
LTK	2.068	1.014	-1.054	0.000	0.000
LTN1	2.728	3.052	0.324	0.000	0.000
LTV1	4.343	5.011	0.668	0.000	0.000
LUC7L	3.876	4.120	0.244	0.000	0.000
LUC7L2	3.058	3.126	0.068	0.012	0.013
LUC7L3	4.895	5.531	0.636	0.000	0.000
LUM	5.586	4.118	-1.468	0.000	0.000
LUZP1	2.021	2.506	0.485	0.000	0.000
LXN	2.653	2.725	0.072	0.324	0.339

LY6E	8.169	5.844	-2.325	0.000	0.000
LY6G5B	1.515	2.051	0.536	0.000	0.000
LY6G5C	0.988	1.277	0.289	0.000	0.000
LY6H	0.104	0.638	0.534	0.000	0.000
LY6K	0.117	0.480	0.363	0.000	0.000
LY75	0.992	1.074	0.082	0.051	0.056
LY86	3.050	2.824	-0.226	0.000	0.000
LY9	0.932	0.665	-0.267	0.000	0.000
LY96	4.001	4.484	0.483	0.000	0.000
LYAR	3.370	4.098	0.728	0.000	0.000
LYG1	1.651	1.996	0.345	0.000	0.000
LYG2	0.117	0.410	0.293	0.000	0.000
LYL1	1.589	1.874	0.285	0.000	0.000
LYN	3.400	3.951	0.551	0.000	0.000
LYNX1	3.703	3.940	0.237	0.002	0.002
LYPD1	1.119	2.595	1.476	0.000	0.000
LYPD2	1.559	0.449	-1.110	0.000	0.000
LYPD3	0.599	1.121	0.522	0.000	0.000
LYPD5	0.363	0.530	0.166	0.000	0.000
LYPD6	0.385	0.615	0.230	0.000	0.000
LYPD6B	0.731	0.599	-0.132	0.034	0.037
LYPLA1	5.019	5.669	0.650	0.000	0.000
LYPLA2	5.665	6.264	0.599	0.000	0.000
LYPLAL1	4.453	5.227	0.774	0.000	0.000
LYRM1	5.321	5.007	-0.313	0.000	0.000
LYRM2	2.907	3.573	0.665	0.000	0.000
LYRM4	2.214	3.166	0.951	0.000	0.000
LYRM7	2.733	3.072	0.339	0.000	0.000
LYSMD1	2.489	3.657	1.168	0.000	0.000
LYSMD2	4.063	4.230	0.167	0.000	0.000
LYSMD3	3.856	4.162	0.306	0.000	0.000
LYSMD4	1.693	2.574	0.881	0.000	0.000
LYST	2.006	2.434	0.428	0.000	0.000
LYVE1	4.066	1.832	-2.234	0.000	0.000
LYZ	5.918	7.785	1.867	0.000	0.000
LZIC	3.438	4.154	0.715	0.000	0.000
LZTFL1	2.500	2.383	-0.117	0.004	0.005
LZTR1	3.713	4.493	0.779	0.000	0.000
LZTS1	0.933	1.513	0.580	0.000	0.000
LZTS2	3.111	3.975	0.864	0.000	0.000
M6PR	4.795	5.331	0.536	0.000	0.000
MAB21L2	1.317	1.049	-0.268	0.000	0.000
MAB21L3	0.999	1.230	0.230	0.000	0.000
MACC1	0.566	0.467	-0.100	0.018	0.020
MACF1	3.642	3.751	0.109	0.006	0.006
MACROD1	5.250	4.702	-0.548	0.000	0.000
MACROD2	0.856	1.225	0.369	0.000	0.000
MAD1L1	3.445	3.755	0.309	0.000	0.000
MAD2L1	0.894	2.529	1.635	0.000	0.000
MAD2L1BP	4.097	4.901	0.804	0.000	0.000
MAD2L2	3.976	4.779	0.803	0.000	0.000
MADCAM1	0.562	0.640	0.078	0.004	0.005

MADD	3.456	3.805	0.349	0.000	0.000
MAEA	3.396	4.031	0.634	0.000	0.000
MAEL	0.180	0.794	0.614	0.000	0.000
MAF	4.532	4.305	-0.227	0.000	0.000
MAF1	5.451	6.563	1.112	0.000	0.000
MAFA	0.120	0.701	0.581	0.000	0.000
MAFB	4.590	4.497	-0.093	0.118	0.127
MAFF	3.828	3.549	-0.280	0.000	0.000
MAFG	2.742	3.977	1.234	0.000	0.000
MAFK	4.071	4.082	0.011	0.855	0.861
MAGEA1	0.205	2.251	2.046	0.000	0.000
MAGED1	5.216	6.004	0.788	0.000	0.000
MAGED2	5.511	6.742	1.231	0.000	0.000
MAGEE1	0.610	0.973	0.362	0.000	0.000
MAGEF1	4.629	5.167	0.538	0.000	0.000
MAGEH1	3.996	4.047	0.051	0.414	0.430
MAGI1	3.117	3.243	0.126	0.001	0.001
MAGI2	0.779	0.805	0.026	0.360	0.375
MAGI3	2.252	2.509	0.257	0.000	0.000
MAGOH	5.145	5.842	0.698	0.000	0.000
MAGOHB	2.773	3.458	0.685	0.000	0.000
MAGT1	5.480	6.083	0.603	0.000	0.000
MAK	0.345	0.406	0.061	0.000	0.000
MAK16	2.789	2.951	0.162	0.000	0.000
MAL	0.728	0.626	-0.102	0.010	0.011
MAL2	4.798	5.643	0.845	0.000	0.000
MALL	1.298	1.733	0.435	0.000	0.000
MALT1	2.879	3.004	0.125	0.002	0.002
MAMDC2	0.906	0.587	-0.320	0.000	0.000
MAMDC4	3.886	3.196	-0.690	0.000	0.000
MAML1	2.381	3.075	0.694	0.000	0.000
MAML2	1.349	1.427	0.077	0.094	0.102
MAML3	1.765	1.928	0.163	0.000	0.000
MAMLD1	1.575	1.978	0.403	0.000	0.000
MAMSTR	0.396	1.157	0.761	0.000	0.000
MAN1A1	6.226	6.006	-0.220	0.000	0.000
MAN1A2	3.269	3.609	0.339	0.000	0.000
MAN1B1	4.112	4.879	0.768	0.000	0.000
MAN1C1	3.707	2.468	-1.239	0.000	0.000
MAN2A1	3.768	4.377	0.609	0.000	0.000
MAN2A2	3.959	4.440	0.482	0.000	0.000
MAN2B1	4.677	5.408	0.731	0.000	0.000
MAN2B2	4.094	4.238	0.144	0.000	0.000
MAN2C1	4.636	5.035	0.399	0.000	0.000
MANBA	2.760	3.186	0.426	0.000	0.000
MANBAL	4.504	5.617	1.113	0.000	0.000
MANEA	3.464	3.617	0.153	0.002	0.002
MANEAL	2.728	3.872	1.143	0.000	0.000
MANF	5.694	6.958	1.264	0.000	0.000
MANSC1	2.274	2.950	0.676	0.000	0.000
MANSC4	0.097	0.164	0.067	0.000	0.000
MAOA	6.231	6.132	-0.099	0.069	0.075

MAOB	7.708	7.386	-0.322	0.000	0.000
MAP1A	0.427	0.726	0.299	0.000	0.000
MAP1B	1.417	2.067	0.651	0.000	0.000
MAP1LC3A	5.466	4.992	-0.474	0.000	0.000
MAP1LC3B	4.356	4.665	0.309	0.000	0.000
MAP1LC3B2	0.517	0.533	0.017	0.300	0.315
MAP1LC3C	0.339	0.343	0.004	0.842	0.849
MAP1S	3.134	3.881	0.747	0.000	0.000
MAP2	1.383	2.677	1.294	0.000	0.000
MAP2K1	5.278	4.952	-0.326	0.000	0.000
MAP2K2	4.934	5.849	0.915	0.000	0.000
MAP2K3	5.657	5.150	-0.508	0.000	0.000
MAP2K4	3.595	3.517	-0.078	0.012	0.013
MAP2K5	2.578	3.212	0.634	0.000	0.000
MAP2K6	1.611	2.060	0.449	0.000	0.000
MAP2K7	3.745	4.262	0.517	0.000	0.000
MAP3K1	2.750	3.279	0.530	0.000	0.000
MAP3K10	2.168	2.890	0.722	0.000	0.000
MAP3K11	4.604	5.341	0.737	0.000	0.000
MAP3K12	1.674	2.020	0.347	0.000	0.000
MAP3K13	2.805	3.514	0.709	0.000	0.000
MAP3K14	3.363	3.534	0.171	0.001	0.001
MAP3K15	0.865	1.026	0.161	0.000	0.000
MAP3K2	3.630	3.898	0.268	0.000	0.000
MAP3K3	3.103	3.572	0.468	0.000	0.000
MAP3K4	2.620	3.162	0.542	0.000	0.000
MAP3K5	2.953	2.935	-0.019	0.775	0.784
MAP3K6	2.717	2.700	-0.018	0.742	0.752
MAP3K7	2.904	3.575	0.671	0.000	0.000
MAP3K8	2.999	3.354	0.355	0.000	0.000
MAP3K9	0.792	1.235	0.443	0.000	0.000
MAP4	4.083	5.049	0.966	0.000	0.000
MAP4K1	2.352	2.154	-0.198	0.001	0.001
MAP4K2	1.910	2.738	0.827	0.000	0.000
MAP4K3	3.212	3.912	0.700	0.000	0.000
MAP4K4	3.802	4.849	1.048	0.000	0.000
MAP4K5	2.658	3.206	0.549	0.000	0.000
MAP6	0.575	0.826	0.251	0.000	0.000
MAP6D1	0.920	1.289	0.369	0.000	0.000
MAP7	3.718	3.601	-0.118	0.017	0.019
MAP7D1	3.508	4.350	0.842	0.000	0.000
MAP7D2	0.255	0.913	0.657	0.000	0.000
MAP7D3	2.065	1.968	-0.097	0.030	0.033
MAP9	0.741	0.614	-0.127	0.001	0.001
MAPK1	3.655	4.580	0.926	0.000	0.000
MAPK10	0.890	0.616	-0.274	0.000	0.000
MAPK11	2.348	3.150	0.802	0.000	0.000
MAPK12	0.978	1.988	1.010	0.000	0.000
MAPK13	1.511	2.337	0.826	0.000	0.000
MAPK14	4.309	4.909	0.600	0.000	0.000
MAPK15	0.616	0.518	-0.098	0.032	0.035
MAPK1IP1L	4.016	4.588	0.572	0.000	0.000

MAPK3	3.626	4.726	1.099	0.000	0.000
MAPK4	0.461	0.780	0.319	0.000	0.000
MAPK6	4.202	5.017	0.815	0.000	0.000
MAPK7	2.101	2.584	0.483	0.000	0.000
MAPK8	2.634	2.871	0.237	0.000	0.000
MAPK8IP1	2.349	2.487	0.139	0.005	0.006
MAPK8IP2	0.474	2.107	1.634	0.000	0.000
MAPK8IP3	3.275	3.811	0.536	0.000	0.000
MAPK9	2.937	3.718	0.781	0.000	0.000
MAPKAP1	4.069	4.929	0.860	0.000	0.000
MAPKAPK2	5.124	6.058	0.934	0.000	0.000
MAPKAPK3	3.807	4.511	0.704	0.000	0.000
MAPKAPK5	2.217	2.983	0.766	0.000	0.000
MAPKBP1	1.546	2.176	0.630	0.000	0.000
MAPRE1	4.573	5.557	0.984	0.000	0.000
MAPRE2	3.987	4.111	0.124	0.005	0.006
MAPRE3	3.832	3.852	0.020	0.635	0.648
MAPT	0.241	1.189	0.948	0.000	0.000
MARCKSL1	5.370	6.191	0.821	0.000	0.000
MARCO	6.000	1.733	-4.267	0.000	0.000
MARK1	0.465	0.747	0.282	0.000	0.000
MARK2	3.037	3.715	0.678	0.000	0.000
MARK3	3.696	4.133	0.437	0.000	0.000
MARK4	2.893	4.213	1.320	0.000	0.000
MARS	4.274	5.267	0.993	0.000	0.000
MARS2	2.514	3.181	0.666	0.000	0.000
MARVELD1	2.388	2.494	0.105	0.113	0.121
MARVELD2	3.024	3.728	0.704	0.000	0.000
MARVELD3	2.023	2.367	0.344	0.000	0.000
MASP1	5.166	4.235	-0.931	0.000	0.000
MASP2	6.963	6.108	-0.855	0.000	0.000
MAST1	0.349	0.872	0.523	0.000	0.000
MAST2	3.149	3.866	0.717	0.000	0.000
MAST3	3.114	3.719	0.606	0.000	0.000
MAST4	2.593	2.539	-0.054	0.287	0.302
MASTL	2.150	3.023	0.873	0.000	0.000
MAT1A	9.908	8.498	-1.409	0.000	0.000
MAT2A	5.755	6.331	0.576	0.000	0.000
MAT2B	4.758	5.222	0.464	0.000	0.000
MATK	1.624	1.437	-0.187	0.000	0.000
MATN1	0.105	0.180	0.075	0.000	0.000
MATN2	3.565	3.791	0.226	0.001	0.001
MATN3	0.189	1.000	0.811	0.000	0.000
MATN4	0.204	0.190	-0.015	0.509	0.524
MATR3	3.533	4.201	0.668	0.000	0.000
MAU2	2.926	3.576	0.650	0.000	0.000
MAVS	2.953	3.886	0.933	0.000	0.000
MAX	4.202	4.585	0.383	0.000	0.000
MB	0.678	0.960	0.282	0.000	0.000
MB21D2	1.118	1.138	0.020	0.632	0.646
MBD1	3.687	4.306	0.619	0.000	0.000
MBD2	4.182	4.614	0.432	0.000	0.000

MBD3	4.402	4.917	0.514	0.000	0.000
MBD4	4.559	4.815	0.256	0.000	0.000
MBD5	1.160	1.557	0.398	0.000	0.000
MBD6	4.300	4.869	0.569	0.000	0.000
MBIP	3.557	3.754	0.198	0.000	0.000
MBL2	6.642	5.031	-1.610	0.000	0.000
MBLAC1	1.478	2.226	0.748	0.000	0.000
MBLAC2	2.095	2.240	0.145	0.000	0.000
MBNL1	4.329	4.604	0.275	0.000	0.000
MBNL2	5.309	4.896	-0.413	0.000	0.000
MBNL3	4.129	5.049	0.920	0.000	0.000
MBOAT1	3.070	3.571	0.501	0.000	0.000
MBOAT2	0.754	0.745	-0.009	0.830	0.837
MBOAT4	0.730	0.853	0.122	0.023	0.025
MBOAT7	3.803	4.675	0.873	0.000	0.000
MBP	2.619	2.847	0.228	0.000	0.000
MBTD1	2.771	3.217	0.447	0.000	0.000
MBTPS1	4.196	4.511	0.316	0.000	0.000
MBTPS2	2.792	3.349	0.558	0.000	0.000
MC1R	0.899	1.317	0.419	0.000	0.000
MCAM	2.935	4.238	1.303	0.000	0.000
MCAT	3.818	4.184	0.366	0.000	0.000
MCC	2.949	2.090	-0.858	0.000	0.000
MCCC1	4.987	4.925	-0.062	0.106	0.115
MCCC2	5.696	5.799	0.104	0.003	0.003
MCEE	4.969	4.737	-0.232	0.000	0.000
MCF2	0.183	0.174	-0.009	0.483	0.498
MCF2L	1.474	1.959	0.485	0.000	0.000
MCFD2	5.606	6.163	0.557	0.000	0.000
MCHR1	1.075	1.666	0.591	0.000	0.000
MCL1	7.988	7.717	-0.271	0.000	0.000
MCM10	0.391	1.507	1.117	0.000	0.000
MCM2	1.789	3.840	2.051	0.000	0.000
MCM3	3.705	5.485	1.780	0.000	0.000
MCM3AP	3.761	4.375	0.613	0.000	0.000
MCM4	2.580	4.395	1.815	0.000	0.000
MCM5	2.837	4.354	1.517	0.000	0.000
MCM6	2.669	4.292	1.623	0.000	0.000
MCM7	4.326	5.795	1.469	0.000	0.000
MCM8	0.996	2.128	1.132	0.000	0.000
MCM9	1.378	1.750	0.372	0.000	0.000
MCMBP	3.847	4.386	0.539	0.000	0.000
MCOLN1	4.027	4.539	0.512	0.000	0.000
MCOLN2	1.162	1.035	-0.127	0.015	0.017
MCOLN3	0.321	0.738	0.417	0.000	0.000
MCPH1	1.722	1.915	0.193	0.000	0.000
MCRS1	4.204	5.247	1.043	0.000	0.000
MCTP1	0.831	1.440	0.610	0.000	0.000
MCTP2	0.800	0.670	-0.131	0.002	0.003
MCTS1	4.684	5.482	0.798	0.000	0.000
MCU	2.725	3.540	0.815	0.000	0.000
MDC1	3.184	3.902	0.718	0.000	0.000

MDFI	0.767	1.634	0.867	0.000	0.000
MDFIC	3.028	3.200	0.172	0.007	0.008
MDGA1	0.526	0.947	0.421	0.000	0.000
MDH1	6.292	6.879	0.587	0.000	0.000
MDH1B	0.316	0.372	0.057	0.009	0.010
MDH2	6.726	7.481	0.755	0.000	0.000
MDK	3.421	6.971	3.550	0.000	0.000
MDM1	1.575	2.050	0.475	0.000	0.000
MDM2	4.517	4.680	0.162	0.000	0.000
MDM4	2.785	3.494	0.710	0.000	0.000
MDN1	3.258	3.134	-0.124	0.006	0.007
MDP1	2.822	2.884	0.063	0.045	0.050
MDS2	0.120	0.126	0.005	0.608	0.622
ME1	2.912	3.852	0.940	0.000	0.000
ME2	2.561	3.362	0.801	0.000	0.000
ME3	2.255	2.683	0.428	0.000	0.000
MEA1	5.945	7.128	1.183	0.000	0.000
MEAF6	4.904	5.520	0.616	0.000	0.000
MECOM	0.911	1.725	0.814	0.000	0.000
MECP2	2.089	2.503	0.414	0.000	0.000
MECR	3.405	3.939	0.534	0.000	0.000
MED1	2.829	3.478	0.648	0.000	0.000
MED10	4.231	4.949	0.718	0.000	0.000
MED11	4.640	4.812	0.172	0.000	0.000
MED12	2.717	3.563	0.846	0.000	0.000
MED13	3.400	3.939	0.539	0.000	0.000
MED13L	2.491	2.892	0.401	0.000	0.000
MED14	3.013	3.515	0.501	0.000	0.000
MED15	3.455	4.148	0.693	0.000	0.000
MED16	4.463	4.892	0.429	0.000	0.000
MED17	2.129	2.683	0.554	0.000	0.000
MED18	3.898	4.251	0.353	0.000	0.000
MED19	3.247	3.985	0.738	0.000	0.000
MED20	2.767	3.876	1.109	0.000	0.000
MED21	3.061	3.614	0.553	0.000	0.000
MED22	2.032	2.936	0.904	0.000	0.000
MED23	2.743	3.286	0.543	0.000	0.000
MED24	3.819	4.822	1.004	0.000	0.000
MED25	2.915	3.860	0.945	0.000	0.000
MED26	1.832	2.420	0.589	0.000	0.000
MED27	2.585	3.487	0.903	0.000	0.000
MED28	2.542	3.056	0.515	0.000	0.000
MED29	4.544	5.217	0.673	0.000	0.000
MED30	3.642	4.343	0.700	0.000	0.000
MED31	2.905	3.221	0.316	0.000	0.000
MED4	4.822	5.191	0.369	0.000	0.000
MED6	2.934	3.529	0.595	0.000	0.000
MED7	2.759	3.484	0.726	0.000	0.000
MED8	4.648	5.387	0.739	0.000	0.000
MED9	2.785	3.275	0.490	0.000	0.000
MEF2A	3.870	4.054	0.184	0.000	0.000
MEF2B	1.125	1.460	0.335	0.000	0.000

MEF2C	2.195	2.415	0.220	0.000	0.000
MEF2D	3.645	4.587	0.942	0.000	0.000
MEFV	0.635	0.454	-0.181	0.000	0.000
MEGF6	2.550	2.493	-0.057	0.456	0.472
MEGF8	2.407	3.306	0.898	0.000	0.000
MEGF9	4.378	4.434	0.056	0.197	0.209
MEI1	1.068	0.924	-0.144	0.001	0.001
MEIG1	0.291	0.417	0.127	0.000	0.000
MEIS1	1.224	1.572	0.349	0.000	0.000
MEIS2	2.021	2.455	0.434	0.000	0.000
MEIS3	0.821	0.914	0.093	0.014	0.016
MELK	0.595	2.912	2.316	0.000	0.000
MEMO1	2.452	3.018	0.566	0.000	0.000
MEN1	3.900	4.709	0.808	0.000	0.000
MEOX1	0.401	0.611	0.210	0.000	0.000
MEOX2	0.594	1.169	0.575	0.000	0.000
MEP1A	0.107	1.668	1.561	0.000	0.000
MEP1B	0.981	0.433	-0.548	0.000	0.000
MEPCE	4.625	5.353	0.728	0.000	0.000
MERTK	3.566	3.765	0.199	0.001	0.001
MESP1	0.770	1.894	1.125	0.000	0.000
MESP2	0.242	1.304	1.063	0.000	0.000
MEST	4.233	4.478	0.245	0.000	0.000
MET	4.722	5.475	0.753	0.000	0.000
METAP1	3.907	4.411	0.505	0.000	0.000
METAP1D	1.958	2.363	0.405	0.000	0.000
METAP2	5.279	5.742	0.463	0.000	0.000
METRN	3.881	3.674	-0.207	0.003	0.003
METRNL	3.442	3.384	-0.058	0.435	0.451
METTL1	3.156	4.005	0.849	0.000	0.000
METTL14	2.937	3.061	0.124	0.000	0.000
METTL15	2.524	2.979	0.454	0.000	0.000
METTL16	2.278	2.697	0.419	0.000	0.000
METTL17	4.281	4.785	0.504	0.000	0.000
METTL18	2.716	3.804	1.088	0.000	0.000
METTL21A	3.154	3.619	0.465	0.000	0.000
METTL22	1.937	2.332	0.395	0.000	0.000
METTL23	4.440	5.341	0.901	0.000	0.000
METTL2A	2.465	3.359	0.895	0.000	0.000
METTL2B	2.341	3.143	0.801	0.000	0.000
METTL3	3.356	4.215	0.859	0.000	0.000
METTL4	1.695	2.318	0.623	0.000	0.000
METTL5	4.131	5.111	0.980	0.000	0.000
METTL6	1.710	2.603	0.893	0.000	0.000
METTL7A	8.599	8.103	-0.496	0.000	0.000
METTL7B	7.668	7.303	-0.365	0.000	0.000
METTL8	1.593	2.055	0.463	0.000	0.000
METTL9	3.150	3.769	0.619	0.000	0.000
MEX3B	0.738	1.135	0.397	0.000	0.000
MEX3C	2.554	3.230	0.676	0.000	0.000
MEX3D	2.889	3.689	0.800	0.000	0.000
MFAP1	3.884	4.727	0.843	0.000	0.000

MFAP2	0.914	1.517	0.603	0.000	0.000
MFAP3	2.718	3.325	0.607	0.000	0.000
MFAP3L	3.479	2.357	-1.121	0.000	0.000
MFAP4	5.270	2.975	-2.296	0.000	0.000
MFAP5	0.139	0.372	0.233	0.000	0.000
MFF	4.081	4.938	0.857	0.000	0.000
MFGE8	2.482	3.473	0.991	0.000	0.000
MFHAS1	2.492	2.963	0.471	0.000	0.000
MFN1	3.031	3.682	0.651	0.000	0.000
MFN2	5.524	5.808	0.284	0.000	0.000
MFNG	2.923	2.995	0.072	0.161	0.172
MFSD1	4.549	5.157	0.608	0.000	0.000
MFSD10	3.476	4.325	0.849	0.000	0.000
MFSD11	2.908	3.587	0.680	0.000	0.000
MFSD2A	6.423	3.234	-3.189	0.000	0.000
MFSD2B	0.156	0.324	0.168	0.000	0.000
MFSD3	4.817	5.726	0.909	0.000	0.000
MFSD5	3.513	4.570	1.057	0.000	0.000
MFSD6	1.638	2.631	0.993	0.000	0.000
MFSD6L	0.226	0.217	-0.009	0.731	0.741
MFSD8	3.060	3.221	0.161	0.000	0.000
MFSD9	2.590	2.962	0.371	0.000	0.000
MGA	1.811	2.259	0.448	0.000	0.000
MGAM	0.168	0.392	0.224	0.000	0.000
MGAT1	5.980	6.500	0.520	0.000	0.000
MGAT3	0.345	0.637	0.292	0.000	0.000
MGAT4A	2.246	3.167	0.921	0.000	0.000
MGAT4B	6.376	7.094	0.719	0.000	0.000
MGAT5	1.540	2.111	0.571	0.000	0.000
MGLL	5.369	4.834	-0.535	0.000	0.000
MGMT	6.044	5.589	-0.454	0.000	0.000
MGP	4.421	4.799	0.377	0.000	0.000
MGRN1	3.932	4.326	0.394	0.000	0.000
MGST1	8.471	8.011	-0.460	0.000	0.000
MGST2	6.741	6.628	-0.113	0.000	0.001
MGST3	4.932	5.472	0.540	0.000	0.000
MIA	0.379	0.611	0.232	0.000	0.000
MIA2	2.303	2.141	-0.161	0.001	0.001
MIA3	5.036	5.472	0.436	0.000	0.000
MIB1	2.844	3.562	0.718	0.000	0.000
MIB2	3.613	3.802	0.189	0.000	0.000
MICA	3.522	4.160	0.638	0.000	0.000
MICAL1	2.402	2.996	0.594	0.000	0.000
MICAL2	2.000	2.462	0.462	0.000	0.000
MICAL3	2.179	2.649	0.469	0.000	0.000
MICALCL	0.419	0.432	0.013	0.514	0.529
MICALL1	2.379	3.236	0.857	0.000	0.000
MICALL2	3.327	3.366	0.039	0.514	0.529
MICB	1.444	2.440	0.996	0.000	0.000
MICU1	5.532	5.468	-0.065	0.040	0.044
MID1	2.044	2.705	0.661	0.000	0.000
MID1IP1	4.170	5.560	1.389	0.000	0.000

MID2	2.348	3.022	0.674	0.000	0.000
MIDN	4.775	4.844	0.069	0.169	0.180
MIER1	3.404	3.456	0.052	0.144	0.154
MIER2	2.294	3.114	0.820	0.000	0.000
MIER3	2.950	3.419	0.470	0.000	0.000
MIF4GD	3.620	4.555	0.935	0.000	0.000
MIIP	3.730	4.552	0.822	0.000	0.000
MINK1	3.889	4.279	0.389	0.000	0.000
MINPP1	4.463	4.715	0.252	0.000	0.000
MIOS	2.802	3.066	0.264	0.000	0.000
MIOX	0.248	0.825	0.577	0.000	0.000
MIP	0.637	0.323	-0.314	0.000	0.000
MIPEP	3.441	3.440	-0.001	0.973	0.975
MIPOL1	0.469	0.914	0.445	0.000	0.000
MIS12	3.307	3.693	0.386	0.000	0.000
MIS18A	2.432	3.631	1.198	0.000	0.000
MIS18BP1	1.895	2.563	0.668	0.000	0.000
MITD1	2.920	3.763	0.843	0.000	0.000
MITF	1.297	1.435	0.138	0.003	0.003
MKI67	0.794	2.775	1.981	0.000	0.000
MKKS	3.935	4.793	0.859	0.000	0.000
MKLN1	3.315	3.592	0.277	0.000	0.000
MKNK1	3.235	3.580	0.345	0.000	0.000
MKNK2	5.165	5.652	0.487	0.000	0.000
MKRN1	4.397	5.251	0.854	0.000	0.000
MKRN2	3.747	4.462	0.715	0.000	0.000
MKRN3	0.007	0.709	0.701	0.000	0.000
MKS1	2.362	2.988	0.626	0.000	0.000
MKX	0.478	0.503	0.025	0.504	0.519
MLANA	0.340	0.394	0.054	0.029	0.033
MLC1	0.225	0.252	0.027	0.152	0.163
MLEC	5.284	6.518	1.234	0.000	0.000
MLF1	1.113	0.972	-0.142	0.007	0.008
MLF2	7.105	7.141	0.036	0.176	0.187
MLH1	3.744	4.405	0.662	0.000	0.000
MLH3	2.879	3.414	0.535	0.000	0.000
MLIP	3.045	2.583	-0.461	0.000	0.000
MLKL	2.363	2.507	0.144	0.001	0.002
MLLT10	3.241	3.793	0.552	0.000	0.000
MLLT11	1.210	1.915	0.705	0.000	0.000
MLLT3	0.940	1.182	0.242	0.000	0.000
MLPH	3.729	3.627	-0.102	0.168	0.179
MLST8	3.848	4.663	0.816	0.000	0.000
MLX	4.840	5.493	0.653	0.000	0.000
MLXIP	3.031	3.214	0.183	0.000	0.000
MLXIPL	7.517	7.560	0.043	0.573	0.587
MLYCD	3.259	2.628	-0.631	0.000	0.000
MMAA	3.595	3.034	-0.561	0.000	0.000
MMAB	5.189	5.131	-0.058	0.152	0.162
MMACHC	3.267	3.361	0.094	0.005	0.006
MMADHC	6.474	6.570	0.096	0.003	0.003
MMD	3.300	4.054	0.754	0.000	0.000

MME	3.349	2.148	-1.201	0.000	0.000
MMEL1	0.494	1.163	0.669	0.000	0.000
MMGT1	2.922	3.604	0.682	0.000	0.000
MMP1	0.466	1.164	0.698	0.000	0.000
MMP10	0.250	0.655	0.404	0.000	0.000
MMP11	0.899	3.033	2.134	0.000	0.000
MMP14	3.905	5.183	1.277	0.000	0.000
MMP15	5.240	5.502	0.262	0.000	0.000
MMP17	0.638	0.857	0.219	0.000	0.000
MMP19	2.857	2.721	-0.136	0.025	0.028
MMP2	3.407	3.451	0.044	0.628	0.641
MMP21	0.333	0.302	-0.031	0.047	0.052
MMP23B	0.885	0.715	-0.169	0.000	0.000
MMP24	1.340	1.831	0.491	0.000	0.000
MMP25	1.204	1.252	0.048	0.213	0.225
MMP7	2.955	2.378	-0.577	0.000	0.000
MMP9	1.675	3.253	1.577	0.000	0.000
MMRN1	2.225	1.293	-0.932	0.000	0.000
MMRN2	2.929	3.360	0.431	0.000	0.000
MMS19	3.612	4.452	0.841	0.000	0.000
MMS22L	0.319	0.954	0.634	0.000	0.000
MN1	0.638	0.808	0.170	0.000	0.000
MNAT1	2.753	3.449	0.696	0.000	0.000
MND1	0.781	2.541	1.759	0.000	0.000
MNDA	2.654	2.411	-0.243	0.000	0.000
MNS1	1.189	2.490	1.300	0.000	0.000
MNT	2.141	2.464	0.323	0.000	0.000
MOAP1	4.243	4.604	0.361	0.000	0.000
MOB2	3.335	3.995	0.660	0.000	0.000
MOCOS	3.432	3.569	0.137	0.003	0.003
MOCS1	4.291	4.540	0.249	0.000	0.000
MOCS2	4.813	5.172	0.359	0.000	0.000
MOCS3	1.915	2.541	0.626	0.000	0.000
MOGAT1	1.491	1.074	-0.417	0.000	0.000
MOGAT2	4.657	2.391	-2.266	0.000	0.000
MOGAT3	3.322	4.218	0.896	0.000	0.000
MOGS	5.200	5.939	0.739	0.000	0.000
MON1A	2.468	3.082	0.615	0.000	0.000
MON1B	3.173	3.490	0.318	0.000	0.000
MON2	3.199	3.336	0.137	0.000	0.000
MORC2	3.386	4.351	0.965	0.000	0.000
MORC3	3.534	3.631	0.097	0.017	0.019
MORC4	3.195	3.889	0.695	0.000	0.000
MORF4L1	5.598	6.223	0.625	0.000	0.000
MORF4L2	6.995	7.733	0.739	0.000	0.000
MORN1	1.019	1.083	0.064	0.010	0.011
MORN2	3.331	4.178	0.847	0.000	0.000
MORN3	0.244	0.239	-0.005	0.742	0.752
MORN4	2.076	2.253	0.177	0.000	0.000
MOSPD1	2.575	3.565	0.990	0.000	0.000
MOSPD2	2.277	2.990	0.713	0.000	0.000
MOSPD3	4.329	5.183	0.854	0.000	0.000

MOV10	4.776	5.503	0.726	0.000	0.000
MOV10L1	0.401	0.543	0.141	0.000	0.000
MOXD1	1.266	1.445	0.179	0.013	0.014
MPDU1	5.895	5.877	-0.018	0.523	0.538
MPDZ	4.595	3.995	-0.600	0.000	0.000
MPEG1	4.249	3.836	-0.413	0.000	0.000
MPG	5.259	5.560	0.301	0.000	0.000
MPHOSPH10	4.084	4.588	0.504	0.000	0.000
MPHOSPH6	3.320	3.942	0.623	0.000	0.000
MPHOSPH8	4.467	4.702	0.235	0.000	0.000
MPHOSPH9	1.997	2.579	0.582	0.000	0.000
MPI	3.295	3.856	0.560	0.000	0.000
MPL	0.341	0.468	0.127	0.000	0.000
MPND	5.046	5.337	0.291	0.000	0.000
MPO	0.224	0.179	-0.045	0.008	0.009
MPP1	4.129	4.330	0.201	0.000	0.000
MPP2	0.251	0.448	0.196	0.000	0.000
MPP3	0.811	1.529	0.718	0.000	0.000
MPP5	2.930	3.300	0.370	0.000	0.000
MPP6	1.923	2.673	0.750	0.000	0.000
MPP7	1.168	1.906	0.738	0.000	0.000
MPPE1	3.047	3.711	0.663	0.000	0.000
MPPED1	3.156	1.825	-1.331	0.000	0.000
MPPED2	0.309	0.634	0.325	0.000	0.000
MPRIP	4.044	4.273	0.230	0.000	0.000
MPST	6.903	7.033	0.129	0.001	0.001
MPV17	3.250	4.305	1.055	0.000	0.000
MPV17L	2.720	3.364	0.644	0.000	0.000
MPV17L2	3.287	4.322	1.034	0.000	0.000
MPZ	1.613	2.831	1.217	0.000	0.000
MPZL1	3.332	4.708	1.377	0.000	0.000
MPZL2	3.573	4.158	0.586	0.000	0.000
MPZL3	2.247	2.417	0.170	0.000	0.000
MR1	2.403	2.889	0.487	0.000	0.000
MRAP	0.996	1.298	0.302	0.000	0.000
MRAP2	0.474	1.851	1.377	0.000	0.000
MRAS	1.932	2.839	0.907	0.000	0.000
MRC2	2.854	2.415	-0.439	0.000	0.000
MREG	2.710	3.063	0.353	0.000	0.000
MRFAP1	7.472	7.973	0.502	0.000	0.000
MRFAP1L1	5.596	6.177	0.581	0.000	0.000
MRGPRF	2.869	1.943	-0.926	0.000	0.000
MRI1	3.785	4.164	0.380	0.000	0.000
MRO	2.015	1.026	-0.989	0.000	0.000
MRPL1	4.612	4.815	0.204	0.000	0.000
MRPL10	4.238	5.116	0.877	0.000	0.000
MRPL11	4.769	5.559	0.790	0.000	0.000
MRPL13	4.227	5.328	1.101	0.000	0.000
MRPL14	6.558	7.223	0.666	0.000	0.000
MRPL15	6.072	6.925	0.852	0.000	0.000
MRPL16	5.570	5.838	0.268	0.000	0.000
MRPL17	4.508	5.319	0.810	0.000	0.000

MRPL18	5.843	6.527	0.684	0.000	0.000
MRPL19	4.633	5.067	0.434	0.000	0.000
MRPL2	5.451	5.924	0.473	0.000	0.000
MRPL20	6.046	6.328	0.282	0.000	0.000
MRPL21	4.779	5.552	0.773	0.000	0.000
MRPL22	3.774	4.451	0.677	0.000	0.000
MRPL23	5.261	5.753	0.492	0.000	0.000
MRPL24	6.347	7.548	1.202	0.000	0.000
MRPL27	5.308	5.876	0.567	0.000	0.000
MRPL28	5.440	6.028	0.589	0.000	0.000
MRPL3	5.155	5.789	0.634	0.000	0.000
MRPL30	3.127	3.752	0.625	0.000	0.000
MRPL32	5.331	5.375	0.044	0.059	0.065
MRPL33	5.178	5.873	0.695	0.000	0.000
MRPL34	6.639	6.622	-0.017	0.592	0.606
MRPL35	4.095	4.693	0.598	0.000	0.000
MRPL36	5.137	5.762	0.625	0.000	0.000
MRPL37	6.302	6.558	0.256	0.000	0.000
MRPL38	3.659	4.263	0.604	0.000	0.000
MRPL39	5.249	5.273	0.024	0.404	0.420
MRPL4	4.690	5.251	0.561	0.000	0.000
MRPL40	5.554	5.883	0.329	0.000	0.000
MRPL41	6.909	7.103	0.194	0.000	0.000
MRPL42	2.740	3.437	0.697	0.000	0.000
MRPL43	5.063	5.637	0.574	0.000	0.000
MRPL44	5.077	5.512	0.434	0.000	0.000
MRPL46	3.879	3.834	-0.044	0.154	0.165
MRPL47	5.042	5.863	0.821	0.000	0.000
MRPL48	3.287	4.038	0.751	0.000	0.000
MRPL49	4.801	5.543	0.742	0.000	0.000
MRPL50	3.490	4.213	0.723	0.000	0.000
MRPL51	6.428	7.150	0.722	0.000	0.000
MRPL53	3.867	4.576	0.710	0.000	0.000
MRPL54	6.723	6.598	-0.125	0.000	0.000
MRPL55	5.441	6.321	0.881	0.000	0.000
MRPL9	4.882	6.106	1.225	0.000	0.000
MRPS10	4.912	5.752	0.840	0.000	0.000
MRPS11	3.710	4.308	0.598	0.000	0.000
MRPS12	4.698	5.716	1.018	0.000	0.000
MRPS14	4.236	5.129	0.893	0.000	0.000
MRPS15	5.764	6.376	0.612	0.000	0.000
MRPS16	5.573	6.319	0.747	0.000	0.000
MRPS17	3.061	4.046	0.985	0.000	0.000
MRPS18B	6.231	6.866	0.635	0.000	0.000
MRPS18C	3.347	3.535	0.187	0.000	0.000
MRPS2	5.322	5.572	0.250	0.000	0.000
MRPS22	4.256	4.649	0.394	0.000	0.000
MRPS23	3.250	4.548	1.298	0.000	0.000
MRPS24	4.450	5.016	0.566	0.000	0.000
MRPS25	4.877	5.267	0.390	0.000	0.000
MRPS27	3.937	4.676	0.740	0.000	0.000
MRPS28	5.119	5.641	0.522	0.000	0.000

MRPS30	3.557	4.119	0.562	0.000	0.000
MRPS31	4.541	4.676	0.135	0.000	0.000
MRPS33	4.339	5.006	0.667	0.000	0.000
MRPS34	6.445	7.087	0.642	0.000	0.000
MRPS35	5.914	6.489	0.575	0.000	0.000
MRPS36	5.035	5.256	0.222	0.000	0.000
MRPS5	4.560	5.124	0.564	0.000	0.000
MRPS6	4.421	4.413	-0.008	0.824	0.831
MRPS7	5.040	5.822	0.782	0.000	0.000
MRPS9	4.631	5.219	0.588	0.000	0.000
MRRF	2.742	3.184	0.442	0.000	0.000
MRS2	3.546	4.431	0.885	0.000	0.000
MRTO4	4.153	4.882	0.730	0.000	0.000
MRVI1	1.132	1.441	0.309	0.000	0.000
MS4A1	1.520	0.969	-0.551	0.000	0.000
MS4A14	1.001	1.116	0.115	0.010	0.011
MS4A2	0.213	0.226	0.013	0.544	0.559
MS4A4A	3.566	3.340	-0.226	0.001	0.001
MS4A6A	5.085	3.871	-1.214	0.000	0.000
MS4A7	4.474	3.633	-0.841	0.000	0.000
MSC	2.091	2.650	0.558	0.000	0.000
MSH2	2.178	3.662	1.484	0.000	0.000
MSH3	2.969	3.386	0.418	0.000	0.000
MSH4	0.094	0.260	0.166	0.000	0.000
MSH5	1.339	2.424	1.085	0.000	0.000
MSH6	2.893	3.843	0.949	0.000	0.000
MSI1	0.670	1.821	1.151	0.000	0.000
MSI2	1.638	2.198	0.560	0.000	0.000
MSL1	4.078	4.819	0.742	0.000	0.000
MSL2	3.313	3.802	0.490	0.000	0.000
MSL3	2.918	3.294	0.376	0.000	0.000
MSLN	0.273	0.316	0.043	0.360	0.375
MSN	5.505	6.148	0.643	0.000	0.000
MSR1	2.555	2.542	-0.013	0.841	0.847
MSRA	4.711	3.816	-0.895	0.000	0.000
MSRB2	5.512	5.743	0.231	0.000	0.000
MSRB3	1.822	2.233	0.411	0.000	0.000
MST1	7.974	7.048	-0.926	0.000	0.000
MST1R	0.777	1.005	0.227	0.000	0.000
MSTN	0.275	0.336	0.061	0.004	0.004
MSTO1	2.103	3.711	1.608	0.000	0.000
MSX1	0.713	1.597	0.884	0.000	0.000
MT1A	6.335	4.508	-1.827	0.000	0.000
MT1E	9.734	6.305	-3.429	0.000	0.000
MT1F	7.401	3.551	-3.849	0.000	0.000
MT1G	10.793	6.239	-4.554	0.000	0.000
MT1H	7.295	2.945	-4.350	0.000	0.000
MT1M	6.844	3.057	-3.787	0.000	0.000
MT1X	9.390	6.206	-3.184	0.000	0.000
MT2A	11.256	8.392	-2.863	0.000	0.000
MTA1	3.829	4.294	0.465	0.000	0.000
MTA2	4.554	5.171	0.617	0.000	0.000

MTA3	2.053	3.004	0.951	0.000	0.000
MTAP	2.444	2.709	0.265	0.000	0.000
MTBP	0.402	1.314	0.912	0.000	0.000
MTCH1	5.982	6.904	0.922	0.000	0.000
MTCH2	6.789	7.156	0.366	0.000	0.000
MTCP1	2.298	2.265	-0.033	0.451	0.467
MTDH	5.565	6.333	0.768	0.000	0.000
MTF1	1.783	2.222	0.439	0.000	0.000
MTF2	2.642	3.127	0.485	0.000	0.000
MTFMT	2.803	3.241	0.438	0.000	0.000
MTFP1	2.908	3.917	1.009	0.000	0.000
MTFR1	4.073	4.887	0.814	0.000	0.000
MTG1	3.669	4.154	0.485	0.000	0.000
MTHFD1	6.951	5.939	-1.012	0.000	0.000
MTHFD1L	1.647	3.129	1.482	0.000	0.000
MTHFD2	1.932	2.249	0.317	0.000	0.000
MTHFD2L	2.104	1.755	-0.350	0.000	0.000
MTHFR	2.546	2.913	0.367	0.000	0.000
MTHFS	6.180	5.914	-0.265	0.000	0.000
MTHFSD	2.065	2.597	0.532	0.000	0.000
MTIF2	4.460	5.289	0.829	0.000	0.000
MTIF3	5.569	6.060	0.491	0.000	0.000
MTM1	3.389	3.580	0.191	0.000	0.000
MTMR1	2.413	3.064	0.651	0.000	0.000
MTMR10	3.576	3.948	0.372	0.000	0.000
MTMR11	1.957	3.025	1.068	0.000	0.000
MTMR12	3.647	4.072	0.425	0.000	0.000
MTMR14	3.660	4.417	0.758	0.000	0.000
MTMR2	1.976	2.738	0.762	0.000	0.000
MTMR3	2.278	2.803	0.525	0.000	0.000
MTMR4	4.373	4.758	0.385	0.000	0.000
MTMR6	3.084	3.449	0.364	0.000	0.000
MTMR7	1.168	1.706	0.538	0.000	0.000
MTMR8	0.329	0.473	0.144	0.000	0.000
MTMR9	1.844	1.850	0.006	0.857	0.862
MTO1	3.055	3.174	0.119	0.000	0.000
MTOR	3.459	4.241	0.781	0.000	0.000
MTPAP	2.464	3.088	0.624	0.000	0.000
MTPN	4.704	5.590	0.885	0.000	0.000
MTR	2.646	3.521	0.876	0.000	0.000
MTRF1	2.639	2.983	0.344	0.000	0.000
MTRF1L	2.678	2.954	0.276	0.000	0.000
MTRNR2L8	0.607	0.581	-0.026	0.409	0.425
MTRNR2L9	0.499	0.475	-0.024	0.413	0.429
MTRR	3.593	4.032	0.439	0.000	0.000
MTSS1	5.311	5.814	0.503	0.000	0.000
MTTP	6.708	5.932	-0.775	0.000	0.000
MTUS1	4.458	4.548	0.090	0.061	0.066
MTX1	3.511	4.629	1.118	0.000	0.000
MTX2	4.683	5.459	0.776	0.000	0.000
MTX3	1.827	2.413	0.586	0.000	0.000
MUC1	0.948	1.331	0.383	0.000	0.000

MUC13	1.422	4.063	2.641	0.000	0.000
MUC20	2.598	2.817	0.218	0.004	0.005
MUC4	0.133	0.176	0.044	0.003	0.004
MUC5B	0.743	0.839	0.096	0.328	0.344
MUC6	2.591	0.738	-1.853	0.000	0.000
MUL1	4.456	4.800	0.344	0.000	0.000
MUM1	3.845	3.937	0.092	0.022	0.024
MUM1L1	1.348	0.863	-0.485	0.000	0.000
MUS81	3.393	4.144	0.751	0.000	0.000
MUSTN1	2.170	2.422	0.252	0.000	0.000
MUT	6.718	6.116	-0.602	0.000	0.000
MUTYH	2.717	3.394	0.677	0.000	0.000
MVD	4.178	5.051	0.874	0.000	0.000
MVK	4.316	4.307	-0.009	0.850	0.856
MVP	5.043	5.914	0.871	0.000	0.000
MX1	3.940	4.110	0.170	0.024	0.027
MX2	1.561	1.725	0.164	0.005	0.006
MXD1	2.507	2.871	0.364	0.000	0.000
MXD3	2.772	4.014	1.243	0.000	0.000
MXD4	3.771	4.580	0.809	0.000	0.000
MXI1	4.430	4.517	0.087	0.021	0.024
MXRA5	1.838	1.377	-0.461	0.000	0.000
MXRA7	2.758	3.333	0.574	0.000	0.000
MXRA8	3.309	3.099	-0.210	0.005	0.006
MYADM	5.000	5.026	0.027	0.647	0.660
MYB	0.219	0.562	0.343	0.000	0.000
MYBBP1A	3.652	4.118	0.466	0.000	0.000
MYBL1	1.162	1.709	0.547	0.000	0.000
MYBL2	0.944	3.698	2.754	0.000	0.000
MYBPC1	0.035	0.281	0.246	0.000	0.000
MYBPC3	0.188	0.199	0.012	0.484	0.500
MYBPH	1.055	0.708	-0.346	0.000	0.000
MYC	5.224	5.048	-0.176	0.035	0.039
MYCBP	3.039	3.743	0.704	0.000	0.000
MYCBP2	3.141	3.049	-0.092	0.033	0.036
MYCBPAP	0.101	0.238	0.137	0.000	0.000
MYCN	0.337	1.294	0.957	0.000	0.000
MYCT1	3.026	2.611	-0.414	0.000	0.000
MYD88	5.297	5.038	-0.259	0.000	0.000
MYEF2	0.440	0.949	0.509	0.000	0.000
MYEOV	1.055	0.776	-0.279	0.000	0.000
MYH10	3.917	3.786	-0.131	0.010	0.011
MYH11	2.025	1.955	-0.070	0.335	0.351
MYH14	4.403	5.094	0.691	0.000	0.000
MYH3	1.558	1.480	-0.079	0.146	0.157
MYH7B	0.931	1.166	0.236	0.000	0.000
MYH9	6.559	7.396	0.837	0.000	0.000
MYL12A	7.203	7.792	0.590	0.000	0.000
MYL12B	8.149	8.879	0.730	0.000	0.000
MYL3	1.170	0.579	-0.591	0.000	0.000
MYL4	0.380	0.543	0.163	0.000	0.000
MYL5	2.847	3.153	0.305	0.000	0.000

MYL6	7.965	8.619	0.654	0.000	0.000
MYL6B	3.397	4.344	0.947	0.000	0.000
MYL9	5.254	5.292	0.038	0.607	0.621
MYLIP	2.543	3.067	0.524	0.000	0.000
MYLK	3.583	3.468	-0.114	0.027	0.030
MYLK2	0.083	0.219	0.136	0.000	0.000
MYLK4	0.455	0.577	0.122	0.000	0.000
MYLPF	0.505	0.382	-0.123	0.000	0.000
MYNN	2.713	3.242	0.529	0.000	0.000
MYO10	2.484	1.685	-0.799	0.000	0.000
MYO15A	0.678	0.422	-0.255	0.000	0.000
MYO16	1.185	0.854	-0.331	0.000	0.000
MYO18A	4.843	4.974	0.131	0.005	0.005
MYO1A	0.310	1.236	0.926	0.000	0.000
MYO1B	6.642	6.286	-0.356	0.000	0.000
MYO1C	4.796	5.328	0.532	0.000	0.000
MYO1D	3.214	3.848	0.635	0.000	0.000
MYO1E	4.037	4.071	0.034	0.510	0.525
MYO1F	3.127	2.663	-0.464	0.000	0.000
MYO1G	1.540	1.443	-0.097	0.044	0.048
MYO3B	0.258	0.209	-0.049	0.016	0.018
MYO5A	1.404	2.030	0.626	0.000	0.000
MYO5B	2.931	3.594	0.663	0.000	0.000
MYO5C	1.608	2.581	0.974	0.000	0.000
MYO6	3.661	4.176	0.515	0.000	0.000
MYO7A	3.405	3.067	-0.338	0.000	0.000
MYO7B	0.971	1.269	0.298	0.000	0.000
MYO9A	1.208	1.130	-0.079	0.010	0.011
MYO9B	3.908	4.618	0.710	0.000	0.000
MYOCD	0.208	0.237	0.029	0.111	0.119
MYOF	1.866	2.337	0.472	0.000	0.000
MYOM1	3.885	4.113	0.229	0.008	0.009
MYOM2	2.020	0.783	-1.238	0.000	0.000
MYOM3	0.072	0.236	0.165	0.000	0.000
MYOT	0.361	0.164	-0.196	0.000	0.000
MYOZ1	0.461	0.451	-0.010	0.740	0.750
MYOZ3	0.160	0.211	0.051	0.000	0.000
MYPOP	2.438	3.446	1.008	0.000	0.000
MYRIP	3.346	3.023	-0.323	0.000	0.000
MYSM1	2.589	2.999	0.411	0.000	0.000
MZB1	3.061	1.895	-1.166	0.000	0.000
MZF1	2.639	3.064	0.425	0.000	0.000
MZT1	2.442	3.644	1.203	0.000	0.000
MZT2A	4.205	4.906	0.701	0.000	0.000
MZT2B	6.095	6.710	0.615	0.000	0.000
N4BP1	3.053	3.346	0.293	0.000	0.000
N4BP2	2.294	2.416	0.122	0.004	0.005
N4BP2L1	5.178	4.183	-0.996	0.000	0.000
N4BP2L2	3.466	3.647	0.182	0.000	0.000
N4BP3	0.373	0.813	0.440	0.000	0.000
N6AMT1	2.038	2.610	0.571	0.000	0.000
NAA10	3.955	5.017	1.061	0.000	0.000

NAA15	2.791	3.279	0.488	0.000	0.000
NAA16	2.381	2.760	0.379	0.000	0.000
NAA20	5.399	6.605	1.205	0.000	0.000
NAA25	2.244	2.787	0.543	0.000	0.000
NAA30	2.951	3.281	0.330	0.000	0.000
NAA35	2.897	3.370	0.473	0.000	0.000
NAA38	5.386	5.916	0.530	0.000	0.000
NAA40	2.208	3.176	0.968	0.000	0.000
NAA50	4.628	5.347	0.719	0.000	0.000
NAAA	4.522	3.538	-0.984	0.000	0.000
NAALAD2	0.641	0.691	0.050	0.193	0.205
NAALADL1	1.110	1.733	0.624	0.000	0.000
NAALADL2	0.854	0.645	-0.209	0.000	0.000
NAB1	4.223	4.437	0.214	0.000	0.000
NAB2	4.558	4.317	-0.241	0.000	0.000
NACAD	0.316	0.287	-0.029	0.151	0.161
NACC1	3.674	4.693	1.019	0.000	0.000
NACC2	2.750	3.465	0.716	0.000	0.000
NADK	5.344	5.501	0.157	0.000	0.000
NADSYN1	3.228	3.655	0.427	0.000	0.000
NAE1	3.499	4.337	0.838	0.000	0.000
NAF1	1.867	2.022	0.154	0.000	0.000
NAGA	4.653	5.165	0.512	0.000	0.000
NAGK	3.883	4.186	0.302	0.000	0.000
NAGLU	5.586	5.738	0.152	0.000	0.000
NAGPA	2.565	3.582	1.018	0.000	0.000
NAGS	4.528	4.444	-0.083	0.328	0.343
NAIF1	2.343	3.038	0.695	0.000	0.000
NALCN	0.716	0.529	-0.187	0.000	0.000
NAMPT	6.046	5.684	-0.361	0.000	0.000
NANOS3	0.183	0.289	0.106	0.000	0.000
NANP	1.919	2.592	0.674	0.000	0.000
NANS	4.035	4.774	0.739	0.000	0.000
NAP1L1	4.515	5.474	0.959	0.000	0.000
NAP1L2	1.028	1.361	0.333	0.000	0.000
NAP1L3	1.052	0.851	-0.201	0.000	0.000
NAP1L4	5.023	5.799	0.775	0.000	0.000
NAP1L5	2.440	2.131	-0.309	0.000	0.000
NAPA	5.680	6.066	0.386	0.000	0.000
NAPB	2.231	2.995	0.764	0.000	0.000
NAPEPLD	2.821	3.070	0.249	0.000	0.000
NAPG	3.279	3.817	0.538	0.000	0.000
NAPSA	0.562	0.806	0.243	0.000	0.000
NARF	3.193	4.340	1.148	0.000	0.000
NARS	5.465	6.417	0.952	0.000	0.000
NARS2	3.853	4.303	0.450	0.000	0.000
NASP	4.200	4.952	0.752	0.000	0.000
NAT1	3.206	2.543	-0.663	0.000	0.000
NAT10	3.666	4.493	0.827	0.000	0.000
NAT14	2.106	2.936	0.830	0.000	0.000
NAT2	5.960	2.836	-3.124	0.000	0.000
NAT8	6.705	5.982	-0.723	0.000	0.000

NAT9	3.602	4.694	1.092	0.000	0.000
NAV1	0.767	1.144	0.377	0.000	0.000
NAV2	3.392	3.121	-0.271	0.000	0.000
NAV3	0.552	1.163	0.611	0.000	0.000
NBAS	3.019	3.873	0.854	0.000	0.000
NBEA	0.598	0.582	-0.016	0.604	0.618
NBEAL1	1.458	1.600	0.143	0.000	0.000
NBEAL2	2.856	3.308	0.452	0.000	0.000
NBL1	1.804	2.321	0.517	0.000	0.000
NBN	3.610	4.404	0.794	0.000	0.000
NBPF1	1.916	2.223	0.307	0.000	0.000
NBPF3	0.683	0.944	0.261	0.000	0.000
NBR1	5.233	5.686	0.453	0.000	0.000
NCALD	2.870	2.625	-0.245	0.000	0.000
NCAM1	1.105	0.484	-0.621	0.000	0.000
NCAM2	0.696	0.400	-0.296	0.000	0.000
NCAPD2	2.280	3.642	1.361	0.000	0.000
NCAPD3	2.444	2.703	0.259	0.000	0.000
NCAPG	0.989	2.910	1.920	0.000	0.000
NCAPG2	1.392	2.768	1.375	0.000	0.000
NCAPH	0.716	2.621	1.906	0.000	0.000
NCAPH2	4.507	5.470	0.963	0.000	0.000
NCBP1	3.466	3.906	0.440	0.000	0.000
NCBP2	4.070	4.915	0.844	0.000	0.000
NCCRP1	0.213	0.318	0.105	0.001	0.001
NCDN	2.323	3.106	0.784	0.000	0.000
NCEH1	2.063	2.575	0.512	0.000	0.000
NCF1	1.581	1.376	-0.205	0.000	0.000
NCF2	2.765	3.302	0.537	0.000	0.000
NCF4	3.181	3.211	0.030	0.619	0.633
NCK1	3.622	4.181	0.559	0.000	0.000
NCK2	3.280	3.811	0.531	0.000	0.000
NCKAP1	3.114	3.993	0.878	0.000	0.000
NCKAP1L	2.258	2.103	-0.155	0.006	0.007
NCKAP5	0.950	0.704	-0.246	0.000	0.000
NCKAP5L	2.300	2.681	0.380	0.000	0.000
NCKIPSD	3.488	4.425	0.938	0.000	0.000
NCL	6.527	7.310	0.783	0.000	0.000
NCLN	4.907	5.693	0.786	0.000	0.000
NCOA1	3.180	3.536	0.356	0.000	0.000
NCOA2	3.209	4.051	0.842	0.000	0.000
NCOA3	3.039	3.755	0.717	0.000	0.000
NCOA5	3.674	4.421	0.747	0.000	0.000
NCOA6	2.749	3.602	0.853	0.000	0.000
NCOA7	4.067	4.013	-0.054	0.379	0.395
NCOR1	4.412	4.184	-0.227	0.000	0.000
NCOR2	3.370	4.189	0.819	0.000	0.000
NCR1	0.469	0.275	-0.195	0.000	0.000
NCR3	1.223	0.941	-0.282	0.000	0.000
NCS1	1.459	2.007	0.549	0.000	0.000
NCSTN	5.204	6.382	1.178	0.000	0.000
NDC80	0.796	2.910	2.114	0.000	0.000

NDE1	1.434	2.309	0.875	0.000	0.000
NDEL1	3.757	3.506	-0.251	0.000	0.000
NDFIP1	5.936	6.353	0.417	0.000	0.000
NDFIP2	4.317	4.197	-0.119	0.003	0.003
NDN	2.755	2.759	0.005	0.954	0.956
NDNF	0.283	0.198	-0.086	0.001	0.001
NDOR1	2.745	3.717	0.972	0.000	0.000
NDRG1	4.741	5.871	1.130	0.000	0.000
NDRG2	7.124	6.047	-1.076	0.000	0.000
NDRG3	2.973	4.258	1.285	0.000	0.000
NDRG4	0.574	0.700	0.126	0.002	0.002
NDST1	4.138	5.095	0.957	0.000	0.000
NDST2	1.910	2.378	0.468	0.000	0.000
NDUFA1	7.630	8.427	0.796	0.000	0.000
NDUFA10	5.090	5.383	0.293	0.000	0.000
NDUFA11	5.579	5.965	0.386	0.000	0.000
NDUFA12	5.649	6.328	0.680	0.000	0.000
NDUFA13	5.638	6.124	0.486	0.000	0.000
NDUFA3	5.805	6.408	0.603	0.000	0.000
NDUFA4	6.597	7.187	0.590	0.000	0.000
NDUFA4L2	2.056	4.150	2.095	0.000	0.000
NDUFA5	4.493	4.744	0.251	0.000	0.000
NDUFA6	6.583	7.219	0.637	0.000	0.000
NDUFA7	2.611	3.098	0.488	0.000	0.000
NDUFA8	6.264	7.009	0.745	0.000	0.000
NDUFA9	3.637	4.064	0.427	0.000	0.000
NDUFAB1	6.415	6.770	0.355	0.000	0.000
NDUFAF1	4.941	5.025	0.084	0.003	0.003
NDUFAF2	4.275	5.181	0.906	0.000	0.000
NDUFAF3	5.340	5.962	0.622	0.000	0.000
NDUFAF4	4.351	4.591	0.240	0.000	0.000
NDUFB1	6.557	6.783	0.226	0.000	0.000
NDUFB10	7.215	7.509	0.294	0.000	0.000
NDUFB11	6.880	7.432	0.552	0.000	0.000
NDUFB2	5.872	6.343	0.471	0.000	0.000
NDUFB3	6.018	6.709	0.691	0.000	0.000
NDUFB4	6.620	7.169	0.549	0.000	0.000
NDUFB5	5.175	5.628	0.453	0.000	0.000
NDUFB6	5.940	6.390	0.450	0.000	0.000
NDUFB7	8.324	8.840	0.516	0.000	0.000
NDUFB8	6.112	6.367	0.255	0.000	0.000
NDUFB9	6.951	7.943	0.992	0.000	0.000
NDUFC1	5.977	6.105	0.127	0.000	0.000
NDUFC2	6.401	6.907	0.506	0.000	0.000
NDUFS1	4.458	4.722	0.264	0.000	0.000
NDUFS2	6.440	7.159	0.718	0.000	0.000
NDUFS3	5.540	6.007	0.468	0.000	0.000
NDUFS4	6.256	6.851	0.594	0.000	0.000
NDUFS5	8.475	8.884	0.409	0.000	0.000
NDUFS6	6.139	7.010	0.871	0.000	0.000
NDUFS8	5.205	5.939	0.734	0.000	0.000
NDUFV1	6.663	7.082	0.420	0.000	0.000

NDUFV2	5.699	5.787	0.088	0.002	0.003
NDUFV3	3.750	4.106	0.355	0.000	0.000
NEB	0.575	1.694	1.120	0.000	0.000
NEBL	1.121	0.940	-0.180	0.002	0.003
NECAB1	0.366	0.583	0.217	0.000	0.000
NECAB2	3.532	3.323	-0.209	0.034	0.038
NECAB3	3.271	4.875	1.604	0.000	0.000
NECAP1	3.688	4.007	0.320	0.000	0.000
NECAP2	4.150	4.625	0.475	0.000	0.000
NEDD1	2.045	2.876	0.830	0.000	0.000
NEDD4	3.221	3.965	0.744	0.000	0.000
NEDD4L	2.585	3.790	1.205	0.000	0.000
NEDD8	5.101	5.955	0.853	0.000	0.000
NEDD8-MDP1	2.056	2.373	0.317	0.000	0.000
NEDD9	2.944	3.442	0.497	0.000	0.000
NEFH	0.390	0.467	0.077	0.021	0.023
NEIL1	3.174	3.152	-0.022	0.662	0.675
NEIL2	3.095	3.105	0.010	0.777	0.787
NEIL3	0.270	1.394	1.124	0.000	0.000
NEK1	1.894	2.014	0.120	0.000	0.000
NEK10	0.311	0.486	0.176	0.000	0.000
NEK11	0.724	0.967	0.243	0.000	0.000
NEK2	0.457	2.946	2.490	0.000	0.000
NEK3	2.670	3.373	0.704	0.000	0.000
NEK4	2.679	3.493	0.815	0.000	0.000
NEK5	0.117	0.230	0.113	0.000	0.000
NEK6	5.144	5.355	0.211	0.000	0.000
NEK7	3.910	4.804	0.894	0.000	0.000
NEK8	1.798	2.430	0.631	0.000	0.000
NEK9	4.104	4.481	0.377	0.000	0.000
NELL2	0.681	0.945	0.264	0.000	0.000
NEMF	3.131	3.470	0.339	0.000	0.000
NENF	5.974	7.005	1.031	0.000	0.000
NEO1	2.837	3.241	0.403	0.000	0.000
NES	2.532	3.634	1.102	0.000	0.000
NET1	4.723	5.199	0.476	0.000	0.000
NETO2	0.455	1.129	0.674	0.000	0.000
NEU1	4.421	6.009	1.588	0.000	0.000
NEU3	1.403	1.697	0.294	0.000	0.000
NEU4	3.607	2.975	-0.632	0.000	0.000
NEURL1B	2.609	3.232	0.622	0.000	0.000
NEURL2	1.978	2.572	0.595	0.000	0.000
NEURL3	1.650	2.338	0.688	0.000	0.000
NEURL4	2.726	3.066	0.340	0.000	0.000
NEXN	2.171	1.847	-0.325	0.000	0.000
NF1	2.249	2.782	0.533	0.000	0.000
NF2	2.316	3.272	0.955	0.000	0.000
NFAM1	1.864	1.487	-0.377	0.000	0.000
NFASC	1.487	1.423	-0.064	0.197	0.210
NFAT5	2.294	2.336	0.042	0.355	0.370
NFATC1	1.565	1.398	-0.167	0.000	0.000
NFATC2	1.683	1.745	0.062	0.201	0.213

NFATC2IP	3.422	4.040	0.618	0.000	0.000
NFATC3	3.204	3.305	0.101	0.004	0.004
NFATC4	1.418	2.016	0.598	0.000	0.000
NFE2	0.515	0.484	-0.031	0.398	0.414
NFE2L1	6.355	7.063	0.708	0.000	0.000
NFE2L2	5.745	5.664	-0.082	0.037	0.040
NFE2L3	2.345	2.825	0.480	0.000	0.000
NFIA	4.321	4.064	-0.257	0.000	0.000
NFIB	3.831	3.827	-0.004	0.927	0.930
NFIC	4.500	4.687	0.187	0.000	0.000
NFIL3	6.271	5.769	-0.502	0.000	0.000
NFIX	3.810	4.317	0.507	0.000	0.000
NFKB1	3.764	3.938	0.174	0.000	0.000
NFKB2	4.388	5.262	0.873	0.000	0.000
NFKBIA	7.224	7.142	-0.082	0.061	0.066
NFKBIB	4.515	4.968	0.453	0.000	0.000
NFKBID	1.797	1.761	-0.036	0.430	0.446
NFKBIE	3.494	4.289	0.795	0.000	0.000
NFKBIL1	4.042	5.107	1.065	0.000	0.000
NFKBIZ	4.386	3.871	-0.515	0.000	0.000
NFRKB	2.924	3.676	0.752	0.000	0.000
NFS1	3.969	4.512	0.543	0.000	0.000
NFU1	4.286	4.887	0.602	0.000	0.000
NFX1	3.943	4.224	0.281	0.000	0.000
NFXL1	2.368	3.046	0.678	0.000	0.000
NFYA	3.388	4.300	0.912	0.000	0.000
NFYB	4.004	4.558	0.555	0.000	0.000
NFYC	3.925	4.607	0.682	0.000	0.000
NGDN	3.619	4.327	0.708	0.000	0.000
NGEF	4.006	4.369	0.364	0.000	0.000
NGF	1.764	1.419	-0.345	0.000	0.000
NGFR	3.902	2.151	-1.751	0.000	0.000
NGLY1	3.325	3.672	0.347	0.000	0.000
NGRN	3.679	4.329	0.650	0.000	0.000
NHEJ1	1.666	2.275	0.608	0.000	0.000
NHLH1	0.196	0.220	0.025	0.073	0.079
NHLRC1	2.510	3.328	0.818	0.000	0.000
NHLRC2	2.260	2.271	0.011	0.765	0.775
NHLRC3	3.533	3.705	0.172	0.000	0.000
NHLRC4	0.423	0.451	0.028	0.179	0.190
NHP2	5.394	6.546	1.152	0.000	0.000
NHS	0.376	0.457	0.082	0.003	0.003
NHSL1	3.433	3.651	0.219	0.000	0.000
NHSL2	0.483	0.549	0.067	0.006	0.007
NICN1	3.480	3.885	0.405	0.000	0.000
NID1	5.593	5.967	0.374	0.000	0.000
NID2	1.832	2.133	0.301	0.000	0.000
NIF3L1	3.696	4.628	0.932	0.000	0.000
NIN	1.888	2.689	0.801	0.000	0.000
NINJ1	5.823	6.263	0.440	0.000	0.000
NINJ2	2.564	3.317	0.753	0.000	0.000
NINL	2.875	3.216	0.340	0.000	0.000

NIP7	3.520	3.989	0.469	0.000	0.000
NIPA1	2.264	2.882	0.618	0.000	0.000
NIPA2	3.688	4.597	0.909	0.000	0.000
NIPAL1	2.127	1.612	-0.516	0.000	0.000
NIPAL2	2.579	3.474	0.895	0.000	0.000
NIPAL3	1.455	2.121	0.666	0.000	0.000
NIPBL	3.187	3.770	0.584	0.000	0.000
NIPSNAP1	7.290	7.423	0.133	0.001	0.001
NIPSNAP3A	4.676	4.690	0.014	0.720	0.731
NIPSNAP3B	1.042	1.250	0.207	0.000	0.000
NISCH	3.982	4.550	0.568	0.000	0.000
NIT1	5.226	5.622	0.396	0.000	0.000
NIT2	5.197	5.020	-0.177	0.000	0.000
NKAIN2	0.256	0.641	0.385	0.000	0.000
NKAP	2.641	3.505	0.863	0.000	0.000
NKAPL	0.958	0.620	-0.338	0.000	0.000
NKD1	0.889	1.846	0.958	0.000	0.000
NKD2	1.753	1.427	-0.326	0.000	0.000
NKG7	4.452	3.806	-0.647	0.000	0.000
NKIRAS1	3.479	3.359	-0.121	0.000	0.000
NKIRAS2	3.448	4.450	1.003	0.000	0.000
NKRF	2.591	3.061	0.470	0.000	0.000
NKTR	3.436	3.776	0.340	0.000	0.000
NKX3-1	1.727	1.252	-0.475	0.000	0.000
NLE1	2.158	3.020	0.862	0.000	0.000
NLGN2	2.166	2.179	0.013	0.817	0.825
NLGN3	0.392	0.429	0.037	0.077	0.083
NLGN4X	0.762	0.941	0.179	0.004	0.004
NLGN4Y	0.632	0.689	0.058	0.161	0.172
NLK	3.013	3.730	0.718	0.000	0.000
NLN	3.288	3.542	0.254	0.000	0.000
NLRC3	1.373	1.251	-0.122	0.004	0.005
NLRC4	1.317	1.165	-0.153	0.000	0.000
NLRC5	2.745	3.023	0.278	0.000	0.000
NLRP1	2.175	2.417	0.242	0.000	0.000
NLRP11	0.597	0.899	0.301	0.000	0.000
NLRP14	0.281	0.218	-0.062	0.000	0.000
NLRP2	0.464	0.519	0.055	0.220	0.233
NLRP3	1.079	0.904	-0.174	0.000	0.000
NLRP6	1.367	1.268	-0.099	0.090	0.097
NLRX1	2.747	3.215	0.468	0.000	0.000
NMB	1.757	3.227	1.470	0.000	0.000
NMD3	4.901	5.156	0.255	0.000	0.000
NME1	4.414	5.896	1.481	0.000	0.000
NME1-NME2	5.331	6.547	1.216	0.000	0.000
NME2	3.306	4.195	0.889	0.000	0.000
NME3	5.267	5.856	0.590	0.000	0.000
NME4	5.611	6.029	0.418	0.000	0.000
NME5	0.863	0.487	-0.375	0.000	0.000
NME6	2.316	3.196	0.880	0.000	0.000
NME7	2.475	3.269	0.794	0.000	0.000
NMI	3.782	4.324	0.542	0.000	0.000

NMNAT1	2.393	2.694	0.302	0.000	0.000
NMNAT2	0.165	0.317	0.152	0.000	0.000
NMNAT3	1.710	2.282	0.572	0.000	0.000
NMRAL1	4.769	4.949	0.180	0.003	0.004
NMT1	5.217	5.879	0.662	0.000	0.000
NMT2	3.752	4.155	0.403	0.000	0.000
NMUR1	0.899	0.616	-0.283	0.000	0.000
NNMT	9.574	7.124	-2.450	0.000	0.000
NNT	5.600	5.565	-0.035	0.485	0.500
NOB1	4.540	5.040	0.500	0.000	0.000
NOC2L	4.606	5.123	0.517	0.000	0.000
NOC3L	2.830	3.331	0.501	0.000	0.000
NOC4L	3.926	4.573	0.648	0.000	0.000
NOD1	1.630	2.058	0.428	0.000	0.000
NOD2	1.030	1.232	0.202	0.000	0.000
NODAL	0.246	0.408	0.163	0.000	0.000
NOL10	3.533	4.363	0.830	0.000	0.000
NOL11	4.046	4.854	0.808	0.000	0.000
NOL3	3.781	4.346	0.565	0.000	0.000
NOL4	0.615	0.260	-0.354	0.000	0.000
NOL6	3.431	4.203	0.772	0.000	0.000
NOL8	2.983	3.629	0.647	0.000	0.000
NOL9	2.310	2.699	0.389	0.000	0.000
NOLC1	4.801	5.375	0.574	0.000	0.000
NOM1	2.493	3.093	0.600	0.000	0.000
NONO	6.041	7.060	1.020	0.000	0.000
NOP10	7.374	8.110	0.736	0.000	0.000
NOP14	4.422	4.759	0.337	0.000	0.000
NOP16	3.607	4.303	0.696	0.000	0.000
NOP2	3.722	4.572	0.850	0.000	0.000
NOP56	4.528	5.608	1.080	0.000	0.000
NOP58	5.159	5.896	0.737	0.000	0.000
NOS1AP	0.829	1.170	0.341	0.000	0.000
NOS2	0.441	0.817	0.376	0.000	0.000
NOS3	2.431	2.326	-0.105	0.037	0.041
NOSIP	4.347	5.075	0.728	0.000	0.000
NOSTRIN	2.579	2.198	-0.381	0.000	0.000
NOTCH1	2.715	3.049	0.335	0.000	0.000
NOTCH2	3.342	3.649	0.306	0.000	0.000
NOTCH3	1.807	3.300	1.493	0.000	0.000
NOTCH4	2.798	3.194	0.396	0.000	0.000
NOTUM	3.710	3.597	-0.113	0.431	0.448
NOV	0.913	1.645	0.732	0.000	0.000
NOVA1	0.378	0.607	0.229	0.000	0.000
NOVA2	0.571	0.853	0.282	0.000	0.000
NOX1	0.373	0.660	0.287	0.000	0.000
NOX4	0.153	0.963	0.810	0.000	0.000
NOXA1	3.824	4.194	0.370	0.000	0.000
NPAS1	0.869	0.818	-0.051	0.241	0.254
NPAS2	1.861	2.785	0.924	0.000	0.000
NPAT	2.046	2.435	0.389	0.000	0.000
NPB	0.314	1.051	0.737	0.000	0.000

NPBWR1	0.789	0.380	-0.410	0.000	0.000
NPC1	2.851	3.905	1.054	0.000	0.000
NPC1L1	4.284	2.945	-1.339	0.000	0.000
NPC2	5.972	6.856	0.884	0.000	0.000
NPDC1	4.162	4.047	-0.116	0.041	0.046
NPEPL1	2.525	3.244	0.719	0.000	0.000
NPEPPS	3.370	4.072	0.702	0.000	0.000
NPFF	1.360	1.501	0.141	0.000	0.000
NPHP1	0.472	0.687	0.215	0.000	0.000
NPHP3	2.153	2.457	0.305	0.000	0.000
NPHP4	1.067	1.077	0.010	0.804	0.812
NPL	3.516	3.927	0.411	0.000	0.000
NPLOC4	4.104	5.321	1.217	0.000	0.000
NPM1	6.855	8.038	1.182	0.000	0.000
NPM2	1.094	2.145	1.050	0.000	0.000
NPM3	3.843	4.848	1.005	0.000	0.000
NPNT	1.940	2.545	0.605	0.000	0.000
NPPA	0.588	0.773	0.185	0.000	0.000
NPR1	2.524	2.509	-0.015	0.741	0.751
NPR2	3.267	4.003	0.736	0.000	0.000
NPR3	1.574	1.346	-0.227	0.000	0.000
NPRL2	3.484	4.167	0.683	0.000	0.000
NPRL3	3.056	3.617	0.561	0.000	0.000
NPTN	4.734	5.648	0.914	0.000	0.000
NPTX2	1.355	1.219	-0.136	0.162	0.173
NPTXR	0.967	1.029	0.062	0.364	0.380
NPW	4.765	3.334	-1.431	0.000	0.000
NPY1R	2.848	1.222	-1.626	0.000	0.000
NPY5R	0.532	0.234	-0.298	0.000	0.000
NQO1	2.064	4.675	2.611	0.000	0.000
NQO2	4.365	4.592	0.228	0.000	0.000
NR0B2	7.088	6.145	-0.943	0.000	0.000
NR1D1	4.213	4.442	0.229	0.000	0.000
NR1D2	3.495	4.041	0.546	0.000	0.000
NR1H2	4.937	5.619	0.682	0.000	0.000
NR1H3	5.398	5.558	0.161	0.000	0.000
NR1H4	5.909	5.774	-0.135	0.004	0.004
NR1I2	5.265	3.797	-1.468	0.000	0.000
NR1I3	6.337	5.394	-0.943	0.000	0.000
NR2C1	3.284	3.682	0.399	0.000	0.000
NR2C2	2.226	2.732	0.506	0.000	0.000
NR2C2AP	3.241	4.800	1.559	0.000	0.000
NR2F1	3.512	3.206	-0.306	0.000	0.000
NR2F2	4.076	4.376	0.301	0.000	0.000
NR2F6	5.813	6.548	0.735	0.000	0.000
NR3C1	4.631	4.571	-0.061	0.148	0.159
NR3C2	2.355	1.787	-0.568	0.000	0.000
NR4A1	4.621	3.450	-1.171	0.000	0.000
NR4A2	3.186	2.517	-0.669	0.000	0.000
NR4A3	2.103	1.242	-0.862	0.000	0.000
NR5A2	3.928	4.247	0.319	0.000	0.000
NR6A1	0.917	1.428	0.511	0.000	0.000

NRAP	2.003	1.826	-0.177	0.000	0.000
NRARP	2.238	3.092	0.854	0.000	0.000
NRAS	4.036	5.099	1.063	0.000	0.000
NRBF2	5.092	4.978	-0.115	0.001	0.001
NRBP1	4.911	5.817	0.906	0.000	0.000
NRBP2	5.428	6.258	0.830	0.000	0.000
NRCAM	0.527	1.562	1.035	0.000	0.000
NRF1	2.435	3.151	0.716	0.000	0.000
NRG1	1.209	0.726	-0.482	0.000	0.000
NRG2	0.321	0.727	0.406	0.000	0.000
NRG3	0.403	0.298	-0.106	0.001	0.001
NRG4	1.001	1.197	0.196	0.000	0.000
NRGN	2.074	2.484	0.411	0.000	0.000
NRIP1	3.604	3.730	0.126	0.006	0.007
NRIP2	1.332	1.846	0.514	0.000	0.000
NRIP3	0.235	0.413	0.178	0.000	0.000
NRL	1.403	1.559	0.156	0.000	0.000
NRM	2.559	3.831	1.272	0.000	0.000
NRN1	2.160	2.052	-0.108	0.054	0.059
NRN1L	0.759	0.696	-0.062	0.008	0.009
NRP1	4.015	4.525	0.510	0.000	0.000
NRP2	1.809	1.942	0.132	0.008	0.010
NRSN2	2.323	3.068	0.745	0.000	0.000
NRTN	3.474	3.640	0.166	0.004	0.004
NRXN2	0.892	1.161	0.269	0.000	0.000
NRXN3	0.283	0.685	0.402	0.000	0.000
NSA2	4.875	5.507	0.631	0.000	0.000
NSD1	2.348	3.204	0.856	0.000	0.000
NSDHL	4.923	5.640	0.717	0.000	0.000
NSF	3.261	4.336	1.075	0.000	0.000
NSFL1C	4.522	5.406	0.884	0.000	0.000
NSL1	3.577	4.520	0.943	0.000	0.000
NSMAF	2.308	3.072	0.764	0.000	0.000
NSMCE1	4.677	5.272	0.595	0.000	0.000
NSMCE2	2.992	4.593	1.601	0.000	0.000
NSMCE4A	4.404	4.764	0.360	0.000	0.000
NSRP1	3.863	4.162	0.299	0.000	0.000
NSUN2	4.802	5.276	0.474	0.000	0.000
NSUN3	1.621	2.065	0.444	0.000	0.000
NSUN4	2.472	2.985	0.512	0.000	0.000
NSUN5	3.170	4.363	1.193	0.000	0.000
NSUN6	4.798	4.306	-0.492	0.000	0.000
NSUN7	0.620	0.625	0.005	0.923	0.926
NT5C	4.460	5.211	0.751	0.000	0.000
NT5DC1	4.008	3.755	-0.253	0.000	0.000
NT5DC2	2.233	3.712	1.479	0.000	0.000
NT5DC3	1.766	2.332	0.566	0.000	0.000
NT5E	4.794	4.764	-0.030	0.639	0.652
NT5M	1.210	2.181	0.972	0.000	0.000
NTAN1	4.123	4.691	0.569	0.000	0.000
NTF3	2.042	0.608	-1.435	0.000	0.000
NTHL1	5.064	4.955	-0.109	0.018	0.020

NTM	0.242	0.767	0.525	0.000	0.000
NTN1	2.195	2.440	0.244	0.000	0.000
NTN3	0.321	0.401	0.080	0.001	0.001
NTN4	3.848	3.297	-0.551	0.000	0.000
NTN5	0.354	0.481	0.127	0.000	0.000
NTNG2	0.314	0.369	0.055	0.037	0.041
NTPCR	2.636	3.581	0.945	0.000	0.000
NTRK1	0.178	0.193	0.015	0.408	0.424
NTRK2	0.541	0.316	-0.225	0.000	0.000
NTS	1.931	1.800	-0.130	0.345	0.361
NTSR1	0.317	0.258	-0.059	0.006	0.007
NUAK1	1.900	2.924	1.024	0.000	0.000
NUAK2	2.546	2.957	0.410	0.000	0.000
NUB1	4.389	5.260	0.871	0.000	0.000
NUBP2	4.515	5.087	0.572	0.000	0.000
NUBPL	2.238	2.500	0.262	0.000	0.000
NUCB1	7.715	8.155	0.439	0.000	0.000
NUCB2	4.081	4.897	0.816	0.000	0.000
NUCKS1	6.598	7.247	0.649	0.000	0.000
NUDC	6.173	6.666	0.493	0.000	0.000
NUDCD1	2.294	3.515	1.222	0.000	0.000
NUDCD2	2.743	3.394	0.651	0.000	0.000
NUDCD3	3.697	4.549	0.852	0.000	0.000
NUDT1	2.195	3.793	1.598	0.000	0.000
NUDT10	0.881	0.234	-0.647	0.000	0.000
NUDT11	0.168	0.262	0.094	0.005	0.006
NUDT12	3.782	3.861	0.078	0.121	0.130
NUDT13	2.597	2.746	0.148	0.001	0.001
NUDT14	3.673	4.361	0.689	0.000	0.000
NUDT15	3.688	4.079	0.391	0.000	0.000
NUDT16	4.257	4.540	0.283	0.000	0.000
NUDT16L1	4.764	5.292	0.527	0.000	0.000
NUDT17	1.262	2.219	0.956	0.000	0.000
NUDT19	2.962	3.625	0.662	0.000	0.000
NUDT2	4.528	5.411	0.883	0.000	0.000
NUDT21	4.125	4.551	0.426	0.000	0.000
NUDT22	4.019	4.416	0.396	0.000	0.000
NUDT4	3.458	3.909	0.450	0.000	0.000
NUDT5	4.931	5.712	0.781	0.000	0.000
NUDT6	2.636	2.245	-0.391	0.000	0.000
NUDT7	4.864	4.653	-0.211	0.000	0.000
NUDT8	4.241	4.344	0.103	0.045	0.050
NUDT9	4.757	4.908	0.151	0.000	0.000
NUF2	0.509	2.779	2.270	0.000	0.000
NUFIP1	2.021	2.483	0.462	0.000	0.000
NUFIP2	3.910	4.405	0.495	0.000	0.000
NUMA1	4.380	4.940	0.560	0.000	0.000
NUMB	4.077	4.224	0.147	0.000	0.000
NUMBL	2.031	2.445	0.414	0.000	0.000
NUP107	2.577	3.638	1.061	0.000	0.000
NUP133	3.163	4.236	1.074	0.000	0.000
NUP153	3.727	4.374	0.646	0.000	0.000

NUP155	1.940	3.014	1.074	0.000	0.000
NUP160	2.989	3.671	0.682	0.000	0.000
NUP188	2.813	3.507	0.693	0.000	0.000
NUP205	2.855	3.939	1.084	0.000	0.000
NUP210	3.534	4.327	0.794	0.000	0.000
NUP214	3.270	3.801	0.532	0.000	0.000
NUP35	2.797	3.310	0.513	0.000	0.000
NUP37	2.368	3.620	1.252	0.000	0.000
NUP43	3.084	3.966	0.881	0.000	0.000
NUP50	3.659	4.143	0.484	0.000	0.000
NUP54	3.753	4.057	0.304	0.000	0.000
NUP62	3.790	4.636	0.846	0.000	0.000
NUP62CL	0.840	1.179	0.339	0.000	0.000
NUP85	3.734	4.642	0.908	0.000	0.000
NUP88	4.343	4.519	0.176	0.000	0.000
NUP93	2.049	2.856	0.807	0.000	0.000
NUP98	3.969	4.465	0.496	0.000	0.000
NUPL2	3.210	4.129	0.919	0.000	0.000
NUPR1	6.113	7.214	1.100	0.000	0.000
NUS1	4.116	4.449	0.333	0.000	0.000
NUSAP1	2.171	4.527	2.356	0.000	0.000
NUTF2	4.508	5.357	0.848	0.000	0.000
NVL	2.514	3.636	1.122	0.000	0.000
NXF1	4.911	5.164	0.252	0.000	0.000
NXF3	1.499	0.395	-1.104	0.000	0.000
NXF5	0.170	0.165	-0.005	0.682	0.694
NXN	2.431	2.138	-0.293	0.000	0.000
NXPH3	0.221	0.476	0.255	0.000	0.000
NXPH4	0.380	1.605	1.225	0.000	0.000
NXT1	3.849	4.633	0.784	0.000	0.000
NXT2	2.657	3.648	0.991	0.000	0.000
NYNRIN	1.867	2.175	0.308	0.000	0.000
OAF	7.668	7.287	-0.381	0.000	0.000
OAS1	4.450	4.412	-0.037	0.534	0.548
OAS2	3.537	3.469	-0.069	0.347	0.363
OAS3	2.803	3.451	0.648	0.000	0.000
OASL	3.745	3.647	-0.098	0.227	0.240
OAT	5.743	4.576	-1.167	0.000	0.000
OAZ1	7.745	8.383	0.638	0.000	0.000
OAZ2	4.749	5.071	0.321	0.000	0.000
OAZ3	0.838	1.563	0.726	0.000	0.000
OBSCN	0.775	0.910	0.136	0.008	0.009
OBSL1	3.965	3.744	-0.221	0.006	0.007
OCA2	0.331	0.700	0.369	0.000	0.000
OCEL1	5.502	5.637	0.134	0.000	0.000
OCIAD1	6.159	6.453	0.294	0.000	0.000
OCIAD2	6.063	5.796	-0.267	0.000	0.000
OCLM	0.645	0.918	0.274	0.000	0.000
OCLN	3.004	3.838	0.833	0.000	0.000
OCM	0.245	0.306	0.061	0.001	0.001
OCRL	3.444	4.194	0.751	0.000	0.000
ODAM	0.202	1.288	1.086	0.000	0.000

ODC1	5.372	6.282	0.910	0.000	0.000
ODF2	3.136	3.634	0.498	0.000	0.000
ODF2L	1.801	1.682	-0.119	0.011	0.013
ODF3B	4.545	4.599	0.054	0.340	0.355
ODF3L1	1.108	0.888	-0.219	0.000	0.000
ODF3L2	0.380	0.373	-0.007	0.731	0.742
OFD1	3.403	4.012	0.609	0.000	0.000
OGDH	4.977	5.767	0.789	0.000	0.000
OGDHL	6.304	4.672	-1.632	0.000	0.000
OGFOD1	3.304	3.764	0.460	0.000	0.000
OGFOD2	2.169	2.678	0.510	0.000	0.000
OGFR	5.186	5.632	0.446	0.000	0.000
OGFRL1	1.714	1.972	0.259	0.000	0.000
OGG1	2.213	3.166	0.954	0.000	0.000
OGN	1.121	1.095	-0.026	0.713	0.725
OGT	4.407	5.219	0.812	0.000	0.000
OIP5	0.977	2.740	1.763	0.000	0.000
OIT3	5.553	3.161	-2.392	0.000	0.000
OLA1	3.686	4.878	1.192	0.000	0.000
OLFM1	1.561	0.929	-0.632	0.000	0.000
OLFM2	4.890	5.517	0.626	0.000	0.000
OLFML1	2.707	2.571	-0.136	0.039	0.043
OLFML2A	0.842	2.026	1.184	0.000	0.000
OLFML2B	1.014	2.673	1.660	0.000	0.000
OLFML3	4.347	2.819	-1.528	0.000	0.000
OLR1	0.813	1.190	0.377	0.000	0.000
OMA1	3.569	3.964	0.395	0.000	0.000
OMD	0.850	0.679	-0.171	0.000	0.000
OMG	0.459	0.343	-0.116	0.000	0.000
OMP	0.635	0.747	0.111	0.002	0.002
ONECUT1	2.452	2.991	0.539	0.000	0.000
ONECUT2	2.859	3.813	0.954	0.000	0.000
OOEP	0.254	0.326	0.072	0.033	0.037
OPA1	3.867	4.403	0.536	0.000	0.000
OPA3	2.318	3.060	0.742	0.000	0.000
OPHN1	0.800	1.248	0.448	0.000	0.000
OPLAH	4.714	5.359	0.645	0.000	0.000
OPRL1	0.548	0.854	0.306	0.000	0.000
OPTN	5.188	6.095	0.907	0.000	0.000
OR2A7	0.973	1.048	0.076	0.054	0.059
OR2B6	0.273	0.824	0.550	0.000	0.000
OR51E1	0.161	0.569	0.409	0.000	0.000
OR52N4	0.422	0.335	-0.087	0.000	0.000
ORAI2	1.344	1.747	0.403	0.000	0.000
ORAI3	4.704	4.807	0.104	0.006	0.007
ORC1	0.662	1.927	1.264	0.000	0.000
ORC2	2.863	3.341	0.478	0.000	0.000
ORC3	3.279	4.113	0.834	0.000	0.000
ORC4	2.972	3.414	0.442	0.000	0.000
ORC5	3.266	3.895	0.629	0.000	0.000
ORC6	0.540	1.893	1.352	0.000	0.000
ORM1	13.114	11.877	-1.237	0.000	0.000

ORM2	11.809	10.557	-1.252	0.000	0.000
ORMDL1	4.354	4.918	0.563	0.000	0.000
ORMDL3	7.279	7.366	0.087	0.019	0.021
OS9	7.249	7.705	0.456	0.000	0.000
OSBP	5.324	5.490	0.165	0.000	0.000
OSBP2	0.608	1.455	0.847	0.000	0.000
OSBPL10	0.826	1.007	0.181	0.000	0.000
OSBPL11	3.771	3.821	0.050	0.265	0.279
OSBPL1A	2.841	3.006	0.166	0.001	0.001
OSBPL2	2.916	3.498	0.582	0.000	0.000
OSBPL3	1.404	2.537	1.134	0.000	0.000
OSBPL5	1.546	1.531	-0.015	0.741	0.752
OSBPL6	1.372	1.257	-0.115	0.005	0.006
OSBPL7	1.242	1.632	0.389	0.000	0.000
OSBPL8	3.136	3.481	0.345	0.000	0.000
OSBPL9	4.861	5.010	0.149	0.000	0.001
OSCAR	1.617	1.570	-0.047	0.351	0.367
OSCP1	1.449	1.723	0.275	0.000	0.000
OSGEP	3.546	4.002	0.456	0.000	0.000
OSGEPL1	2.227	3.003	0.776	0.000	0.000
OSGIN1	6.049	6.704	0.656	0.000	0.000
OSGIN2	3.378	3.998	0.620	0.000	0.000
OSM	1.056	1.025	-0.031	0.555	0.569
OSMR	3.158	3.179	0.021	0.781	0.790
OSR1	0.283	0.659	0.377	0.000	0.000
OSR2	0.185	0.959	0.773	0.000	0.000
OST4	7.963	8.639	0.675	0.000	0.000
OSTC	6.824	7.221	0.396	0.000	0.000
OSTF1	5.632	5.665	0.032	0.309	0.325
OSTM1	3.276	3.965	0.689	0.000	0.000
OTC	7.548	6.394	-1.153	0.000	0.000
OTOA	0.222	0.282	0.060	0.000	0.000
OTUB1	4.390	5.152	0.762	0.000	0.000
OTUB2	0.638	1.500	0.862	0.000	0.000
OTUD1	3.281	3.391	0.111	0.007	0.008
OTUD3	1.409	1.868	0.459	0.000	0.000
OTUD4	3.448	3.475	0.027	0.492	0.508
OTUD5	4.900	5.354	0.454	0.000	0.000
OTUD6B	2.205	3.291	1.086	0.000	0.000
OTUD7A	0.159	0.276	0.117	0.000	0.000
OTX1	0.087	0.786	0.700	0.000	0.000
OVCA2	3.198	3.290	0.092	0.000	0.000
OVGP1	2.914	3.021	0.107	0.080	0.087
OVOL1	0.218	0.472	0.254	0.000	0.000
OVOL2	0.381	0.386	0.005	0.908	0.912
OXA1L	5.794	6.465	0.671	0.000	0.000
OXCT1	1.284	1.677	0.394	0.000	0.000
OXCT2	0.743	0.868	0.125	0.001	0.001
OXER1	5.012	4.452	-0.560	0.000	0.000
OXNAD1	3.166	3.135	-0.031	0.285	0.300
OXR1	3.361	3.908	0.548	0.000	0.000
OXSM	3.857	4.108	0.250	0.000	0.000

OXSR1	3.665	4.097	0.432	0.000	0.000
OXT	3.521	1.399	-2.122	0.000	0.000
OXTR	0.453	0.805	0.352	0.000	0.000
P2RX1	0.831	0.727	-0.104	0.006	0.007
P2RX3	0.439	0.397	-0.043	0.095	0.102
P2RX4	2.735	3.666	0.931	0.000	0.000
P2RX5	0.962	0.916	-0.046	0.329	0.345
P2RX6	0.496	0.403	-0.093	0.003	0.004
P2RX7	1.893	2.261	0.369	0.000	0.000
P2RY1	0.690	0.645	-0.044	0.151	0.162
P2RY10	1.154	0.945	-0.209	0.000	0.000
P2RY11	2.618	3.068	0.450	0.000	0.000
P2RY12	1.069	0.533	-0.536	0.000	0.000
P2RY13	2.255	1.483	-0.772	0.000	0.000
P2RY14	0.752	0.813	0.061	0.084	0.091
P2RY2	1.435	1.881	0.446	0.000	0.000
P2RY6	0.951	1.294	0.343	0.000	0.000
P4HA1	5.376	5.582	0.205	0.003	0.003
P4HA2	2.196	3.740	1.544	0.000	0.000
P4HA3	0.799	0.724	-0.074	0.023	0.026
P4HB	9.273	10.066	0.793	0.000	0.000
P4HTM	3.074	3.164	0.091	0.086	0.093
PA2G4	5.215	6.219	1.004	0.000	0.000
PAAF1	2.582	3.139	0.557	0.000	0.000
PABPC1	7.684	8.941	1.257	0.000	0.000
PABPC1L	3.647	4.452	0.804	0.000	0.000
PABPC3	0.721	0.835	0.114	0.000	0.000
PABPC4	5.278	5.831	0.552	0.000	0.000
PABPC4L	0.210	0.462	0.252	0.000	0.000
PABPC5	0.274	0.339	0.065	0.011	0.013
PABPN1	5.507	6.179	0.672	0.000	0.000
PACRG	1.367	1.188	-0.179	0.001	0.001
PACRGL	1.685	2.240	0.555	0.000	0.000
PACS1	2.995	3.677	0.682	0.000	0.000
PACS2	2.659	3.473	0.814	0.000	0.000
PACSIN1	0.416	0.884	0.468	0.000	0.000
PACSIN2	4.659	5.576	0.917	0.000	0.000
PACSIN3	4.708	4.661	-0.047	0.440	0.456
PADI2	0.589	0.776	0.187	0.000	0.000
PADI4	0.408	0.170	-0.238	0.000	0.000
PAF1	5.435	5.861	0.426	0.000	0.000
PAFAH1B1	4.298	4.563	0.264	0.000	0.000
PAFAH1B2	3.943	4.550	0.607	0.000	0.000
PAFAH1B3	2.687	4.448	1.761	0.000	0.000
PAFAH2	3.883	3.849	-0.035	0.310	0.325
PAG1	2.062	2.689	0.628	0.000	0.000
PAGE2B	0.232	1.142	0.910	0.000	0.000
PAGE4	0.293	1.657	1.363	0.000	0.000
PAGE5	0.662	1.630	0.969	0.000	0.000
PAH	9.001	8.228	-0.773	0.000	0.000
PAICS	5.085	5.633	0.548	0.000	0.000
PAIP1	4.733	5.471	0.739	0.000	0.000

PAIP2B	3.293	2.421	-0.872	0.000	0.000
PAK1	3.116	4.071	0.956	0.000	0.000
PAK1IP1	3.789	4.720	0.931	0.000	0.000
PAK2	4.465	5.089	0.624	0.000	0.000
PAK3	0.191	0.316	0.125	0.000	0.000
PAK4	3.263	4.114	0.851	0.000	0.000
PAK6	0.119	0.352	0.233	0.000	0.000
PALB2	2.251	2.987	0.736	0.000	0.000
PALLD	3.033	3.996	0.963	0.000	0.000
PALM	2.399	2.597	0.198	0.001	0.001
PALM2	2.439	1.697	-0.742	0.000	0.000
PALM2-AKAP	1.824	1.694	-0.130	0.000	0.000
PALM3	5.217	3.830	-1.388	0.000	0.000
PALMD	3.955	4.060	0.105	0.073	0.079
PAM	2.841	3.232	0.390	0.000	0.000
PAM16	3.835	4.296	0.460	0.000	0.000
PAMR1	2.834	1.424	-1.410	0.000	0.000
PAN2	4.422	4.608	0.186	0.000	0.000
PAN3	3.157	3.378	0.222	0.000	0.000
PANK1	5.189	4.143	-1.046	0.000	0.000
PANK2	2.983	3.487	0.504	0.000	0.000
PANK3	3.636	4.106	0.470	0.000	0.000
PANK4	3.089	3.366	0.277	0.000	0.000
PANX1	3.717	4.052	0.335	0.000	0.000
PANX2	2.770	3.379	0.609	0.000	0.000
PAOX	3.770	3.959	0.189	0.000	0.000
PAPLN	1.887	2.236	0.350	0.000	0.000
PAPOLA	4.678	5.251	0.573	0.000	0.000
PAPOLG	1.721	2.205	0.485	0.000	0.000
PAPPA	0.395	0.411	0.016	0.595	0.609
PAPPA2	0.546	0.985	0.439	0.000	0.000
PAPSS1	3.005	3.994	0.989	0.000	0.000
PAPSS2	5.572	5.251	-0.321	0.000	0.000
PAQR3	1.314	1.711	0.396	0.000	0.000
PAQR4	1.581	3.112	1.531	0.000	0.000
PAQR5	1.301	1.809	0.509	0.000	0.000
PAQR6	1.672	2.070	0.399	0.000	0.000
PAQR7	2.724	3.022	0.298	0.000	0.000
PAQR8	1.280	1.791	0.511	0.000	0.000
PAQR9	3.142	3.939	0.797	0.000	0.000
PARD3	3.673	4.352	0.678	0.000	0.000
PARD3B	1.690	2.340	0.650	0.000	0.000
PARD6A	2.415	2.653	0.237	0.000	0.000
PARD6B	2.147	2.661	0.515	0.000	0.000
PARD6G	1.596	2.402	0.806	0.000	0.000
PARG	2.292	3.060	0.767	0.000	0.000
PARK7	7.449	7.770	0.321	0.000	0.000
PARL	5.081	5.729	0.648	0.000	0.000
PARM1	1.392	1.787	0.395	0.000	0.000
PARN	3.700	4.346	0.646	0.000	0.000
PARP1	4.525	5.774	1.249	0.000	0.000
PARP10	5.044	5.762	0.718	0.000	0.000

PARP11	1.611	1.848	0.237	0.000	0.000
PARP12	3.687	4.692	1.005	0.000	0.000
PARP14	4.331	4.759	0.428	0.000	0.000
PARP15	1.183	1.180	-0.003	0.945	0.948
PARP16	3.157	3.557	0.400	0.000	0.000
PARP2	3.088	4.069	0.981	0.000	0.000
PARP3	4.315	4.374	0.059	0.204	0.217
PARP4	4.091	4.631	0.540	0.000	0.000
PARP6	3.480	3.387	-0.093	0.132	0.142
PARP8	2.409	2.444	0.035	0.488	0.503
PARP9	4.814	4.914	0.100	0.014	0.016
PARS2	1.922	2.740	0.817	0.000	0.000
PARVA	3.535	3.565	0.030	0.477	0.493
PARVB	2.522	3.296	0.774	0.000	0.000
PARVG	2.543	2.185	-0.358	0.000	0.000
PASK	1.353	1.833	0.480	0.000	0.000
PATL1	4.333	4.718	0.385	0.000	0.000
PATL2	1.300	1.323	0.023	0.613	0.627
PATZ1	3.417	4.083	0.666	0.000	0.000
PAWR	3.233	3.468	0.235	0.000	0.000
PAX5	0.239	0.212	-0.027	0.204	0.216
PAX6	0.254	0.473	0.218	0.000	0.000
PAX8	0.640	1.188	0.548	0.000	0.000
PAXIP1	1.772	2.550	0.777	0.000	0.000
PBK	0.687	2.983	2.296	0.000	0.000
PBLD	6.819	5.258	-1.561	0.000	0.000
PBRM1	3.182	3.575	0.393	0.000	0.000
PBX1	1.640	1.193	-0.447	0.000	0.000
PBX2	4.330	5.114	0.783	0.000	0.000
PBX3	3.386	3.800	0.414	0.000	0.000
PBX4	0.861	0.820	-0.041	0.321	0.336
PBXIP1	5.258	6.253	0.994	0.000	0.000
PC	6.914	6.478	-0.436	0.000	0.000
PCBD1	8.234	7.960	-0.274	0.000	0.000
PCBD2	2.084	2.543	0.459	0.000	0.000
PCBP2	6.639	7.130	0.491	0.000	0.000
PCBP3	0.426	0.361	-0.066	0.012	0.013
PCBP4	3.592	4.372	0.781	0.000	0.000
PCCA	5.086	4.741	-0.345	0.000	0.000
PCCB	5.568	5.491	-0.076	0.046	0.051
PCDH1	3.682	3.805	0.123	0.015	0.017
PCDH12	1.879	2.481	0.602	0.000	0.000
PCDH17	0.951	1.742	0.791	0.000	0.000
PCDH18	1.176	0.988	-0.188	0.000	0.000
PCDH20	0.346	0.541	0.195	0.000	0.000
PCDH7	0.434	0.394	-0.040	0.191	0.203
PCDH9	0.369	0.201	-0.168	0.000	0.000
PCDHA1	0.495	0.688	0.193	0.000	0.000
PCDHA11	0.469	0.533	0.064	0.059	0.064
PCDHA12	0.542	0.574	0.032	0.397	0.413
PCDHA13	0.539	0.598	0.059	0.124	0.133
PCDHA7	0.462	0.552	0.090	0.010	0.011

PCDHAC1	0.488	0.522	0.033	0.375	0.391
PCDHAC2	0.612	0.657	0.045	0.320	0.336
PCDHB10	0.299	0.666	0.367	0.000	0.000
PCDHB11	0.233	0.489	0.256	0.000	0.000
PCDHB12	0.395	0.547	0.152	0.000	0.000
PCDHB13	0.432	0.837	0.404	0.000	0.000
PCDHB14	0.759	1.305	0.547	0.000	0.000
PCDHB15	0.746	0.830	0.084	0.019	0.021
PCDHB2	0.187	0.663	0.476	0.000	0.000
PCDHB3	0.175	0.447	0.272	0.000	0.000
PCDHB4	0.470	0.641	0.171	0.000	0.000
PCDHB5	0.755	1.097	0.342	0.000	0.000
PCDHB6	0.220	0.454	0.235	0.000	0.000
PCDHB7	0.414	0.605	0.191	0.000	0.000
PCDHB8	0.176	0.472	0.296	0.000	0.000
PCDHB9	0.197	0.437	0.240	0.000	0.000
PCDHGA1	1.298	1.519	0.221	0.000	0.000
PCDHGA10	1.732	1.829	0.097	0.025	0.027
PCDHGA11	1.391	1.441	0.051	0.105	0.113
PCDHGA12	1.707	1.696	-0.011	0.768	0.778
PCDHGA2	1.511	1.787	0.276	0.000	0.000
PCDHGA3	1.566	1.704	0.138	0.001	0.001
PCDHGA4	1.518	1.832	0.314	0.000	0.000
PCDHGA5	1.382	1.551	0.169	0.000	0.000
PCDHGA6	1.321	1.483	0.161	0.000	0.000
PCDHGA7	1.447	1.618	0.171	0.000	0.000
PCDHGA8	1.137	1.238	0.101	0.001	0.001
PCDHGA9	1.541	1.614	0.073	0.032	0.036
PCDHGB1	1.439	1.648	0.209	0.000	0.000
PCDHGB2	1.654	1.918	0.264	0.000	0.000
PCDHGB3	1.451	1.554	0.103	0.002	0.002
PCDHGB4	1.513	1.739	0.226	0.000	0.000
PCDHGB6	1.562	1.683	0.122	0.002	0.002
PCDHGB7	1.802	1.722	-0.080	0.036	0.040
PCDHGC3	2.153	2.116	-0.036	0.466	0.481
PCDHGC5	1.393	1.418	0.025	0.367	0.382
PCF11	3.141	3.401	0.260	0.000	0.000
PCGF1	3.240	4.186	0.945	0.000	0.000
PCGF3	3.536	4.102	0.566	0.000	0.000
PCGF5	4.209	4.526	0.317	0.000	0.000
PCGF6	2.979	3.378	0.399	0.000	0.000
PCID2	3.380	4.031	0.650	0.000	0.000
PCIF1	3.841	4.592	0.751	0.000	0.000
PCK1	9.586	6.709	-2.877	0.000	0.000
PCK2	8.442	7.441	-1.001	0.000	0.000
PCLO	0.234	0.324	0.090	0.002	0.002
PCM1	4.096	4.468	0.372	0.000	0.000
PCMT1	5.197	5.450	0.254	0.000	0.000
PCMTD1	3.636	4.190	0.554	0.000	0.000
PCMTD2	4.171	4.845	0.674	0.000	0.000
PCNA	5.493	6.920	1.426	0.000	0.000
PCNP	5.451	6.047	0.596	0.000	0.000

PCNT	2.466	2.889	0.423	0.000	0.000
PCOLCE	5.544	4.818	-0.726	0.000	0.000
PCOLCE2	3.023	3.902	0.879	0.000	0.000
PCP2	1.634	1.496	-0.138	0.008	0.010
PCP4L1	2.031	2.046	0.015	0.848	0.855
PCSK1N	0.838	1.610	0.772	0.000	0.000
PCSK4	1.930	2.078	0.148	0.002	0.003
PCSK5	0.757	1.260	0.503	0.000	0.000
PCSK6	5.431	5.049	-0.382	0.000	0.000
PCSK7	2.171	2.626	0.454	0.000	0.000
PCSK9	3.856	4.634	0.778	0.000	0.000
PCTP	5.139	5.019	-0.120	0.002	0.002
PCYOX1	5.717	5.789	0.071	0.089	0.096
PCYOX1L	1.021	1.452	0.431	0.000	0.000
PCYT1A	3.224	3.750	0.526	0.000	0.000
PCYT2	5.656	6.262	0.606	0.000	0.000
PDAP1	5.476	6.445	0.968	0.000	0.000
PDCD1	1.315	1.420	0.104	0.129	0.139
PDCD10	3.661	4.539	0.878	0.000	0.000
PDCD11	3.280	4.054	0.774	0.000	0.000
PDCD1LG2	1.778	1.343	-0.434	0.000	0.000
PDCD2	3.315	3.898	0.583	0.000	0.000
PDCD2L	2.445	3.601	1.156	0.000	0.000
PDCD4	4.898	5.187	0.289	0.000	0.000
PDCD5	5.068	6.199	1.130	0.000	0.000
PDCD6IP	4.467	5.107	0.640	0.000	0.000
PDCD7	3.369	3.964	0.595	0.000	0.000
PDCL	2.499	3.356	0.857	0.000	0.000
PDCL3	3.249	4.251	1.003	0.000	0.000
PDE10A	0.475	0.555	0.080	0.002	0.003
PDE11A	1.434	1.005	-0.429	0.000	0.000
PDE12	2.692	3.198	0.506	0.000	0.000
PDE1A	1.262	1.040	-0.221	0.000	0.000
PDE1B	1.174	1.038	-0.136	0.000	0.000
PDE1C	0.089	0.194	0.104	0.000	0.000
PDE2A	3.075	2.114	-0.962	0.000	0.000
PDE3A	1.006	1.159	0.152	0.002	0.002
PDE3B	3.322	3.143	-0.179	0.000	0.000
PDE4A	1.692	1.869	0.177	0.001	0.001
PDE4B	2.243	1.998	-0.245	0.000	0.000
PDE4C	0.440	0.972	0.532	0.000	0.000
PDE4D	0.966	1.030	0.064	0.094	0.102
PDE4DIP	3.335	3.640	0.305	0.000	0.000
PDE5A	1.081	1.400	0.319	0.000	0.000
PDE6B	0.597	0.666	0.069	0.052	0.056
PDE6C	0.259	0.387	0.128	0.000	0.000
PDE6D	3.073	3.974	0.901	0.000	0.000
PDE6G	1.705	1.426	-0.279	0.000	0.000
PDE7A	2.181	2.786	0.605	0.000	0.000
PDE7B	2.043	1.150	-0.894	0.000	0.000
PDE8A	4.138	4.207	0.069	0.056	0.061
PDE8B	1.006	0.948	-0.058	0.066	0.072

PDE9A	1.876	2.105	0.229	0.001	0.001
PDGFA	2.299	3.860	1.561	0.000	0.000
PDGFB	2.138	3.280	1.143	0.000	0.000
PDGFC	3.214	2.878	-0.336	0.000	0.000
PDGFD	2.234	2.349	0.115	0.076	0.082
PDGFRA	3.181	1.830	-1.350	0.000	0.000
PDGFRB	3.526	4.480	0.954	0.000	0.000
PDGFRL	0.779	1.522	0.743	0.000	0.000
PDHA1	5.779	6.165	0.385	0.000	0.000
PDHB	5.261	5.655	0.394	0.000	0.000
PDHX	4.138	4.632	0.494	0.000	0.000
PDIA2	0.217	0.974	0.757	0.000	0.000
PDIA3	7.504	8.413	0.909	0.000	0.000
PDIA4	7.305	8.176	0.871	0.000	0.000
PDIA5	5.354	5.395	0.042	0.266	0.281
PDIA6	6.633	7.674	1.041	0.000	0.000
PDIK1L	2.581	3.081	0.500	0.000	0.000
PDK1	1.690	2.364	0.674	0.000	0.000
PDK2	4.582	4.569	-0.013	0.764	0.774
PDK3	2.115	2.387	0.273	0.000	0.000
PDK4	7.031	5.828	-1.202	0.000	0.000
PDLIM1	6.921	7.292	0.372	0.000	0.000
PDLIM2	2.958	2.606	-0.352	0.000	0.000
PDLIM3	1.973	2.335	0.362	0.000	0.000
PDLIM4	0.905	0.989	0.084	0.103	0.111
PDLIM5	4.994	4.544	-0.450	0.000	0.000
PDLIM7	2.869	3.903	1.034	0.000	0.000
PDP1	1.931	2.182	0.251	0.000	0.000
PDP2	1.956	2.263	0.306	0.000	0.000
PDPK1	2.638	3.345	0.707	0.000	0.000
PDPN	0.536	0.806	0.270	0.000	0.000
PDPR	2.092	2.481	0.389	0.000	0.000
PDS5A	3.812	4.486	0.673	0.000	0.000
PDS5B	2.319	2.735	0.416	0.000	0.000
PDSS1	2.517	3.578	1.062	0.000	0.000
PDSS2	3.902	3.992	0.090	0.006	0.007
PDX1	0.917	1.196	0.280	0.001	0.001
PDXDC1	5.303	5.421	0.118	0.001	0.001
PDXK	4.060	4.765	0.705	0.000	0.000
PDXP	3.458	3.830	0.372	0.000	0.000
PDZD11	4.641	5.859	1.218	0.000	0.000
PDZD2	0.422	0.699	0.277	0.000	0.000
PDZD3	0.256	0.297	0.041	0.198	0.210
PDZD4	1.639	1.140	-0.499	0.000	0.000
PDZD7	0.306	0.601	0.295	0.000	0.000
PDZD8	2.904	3.205	0.301	0.000	0.000
PDZD9	0.101	0.147	0.046	0.000	0.000
PDZK1	5.264	6.347	1.083	0.000	0.000
PDZK1IP1	2.999	4.567	1.568	0.000	0.000
PDZRN3	1.077	0.719	-0.358	0.000	0.000
PDZRN4	0.578	0.217	-0.361	0.000	0.000
PEA15	5.107	6.714	1.606	0.000	0.000

PEAK1	1.421	1.966	0.545	0.000	0.000
PEAR1	2.110	1.901	-0.209	0.000	0.000
PEBP1	10.817	10.462	-0.355	0.000	0.000
PEBP4	0.528	0.478	-0.051	0.338	0.354
PECR	6.665	6.216	-0.449	0.000	0.000
PEF1	5.585	6.099	0.515	0.000	0.000
PEG10	1.705	4.452	2.747	0.000	0.000
PEG3	1.778	1.529	-0.249	0.006	0.007
PELI1	3.445	3.766	0.321	0.000	0.000
PELI2	1.305	0.885	-0.420	0.000	0.000
PELI3	1.662	2.312	0.650	0.000	0.000
PELO	3.497	3.858	0.361	0.000	0.000
PELP1	3.702	4.304	0.602	0.000	0.000
PEMT	7.071	6.012	-1.059	0.000	0.000
PEPD	6.621	6.522	-0.100	0.018	0.021
PER1	5.178	4.585	-0.594	0.000	0.000
PER2	2.947	2.959	0.011	0.787	0.796
PER3	3.195	3.206	0.010	0.852	0.858
PERP	6.060	6.356	0.297	0.000	0.000
PES1	4.549	5.509	0.960	0.000	0.000
PET117	2.997	3.796	0.799	0.000	0.000
PEX1	2.944	3.446	0.502	0.000	0.000
PEX10	3.627	4.079	0.452	0.000	0.000
PEX11A	4.109	4.088	-0.020	0.637	0.651
PEX11B	4.612	5.595	0.983	0.000	0.000
PEX11G	4.464	3.474	-0.990	0.000	0.000
PEX12	2.883	3.160	0.277	0.000	0.000
PEX13	4.077	4.121	0.044	0.128	0.137
PEX14	4.372	4.351	-0.021	0.529	0.544
PEX16	4.516	5.040	0.524	0.000	0.000
PEX19	5.709	6.206	0.498	0.000	0.000
PEX2	3.800	4.784	0.984	0.000	0.000
PEX26	2.275	2.850	0.575	0.000	0.000
PEX3	4.182	4.098	-0.085	0.026	0.029
PEX5	4.290	4.854	0.564	0.000	0.000
PEX6	4.251	5.179	0.928	0.000	0.000
PEX7	3.135	3.609	0.474	0.000	0.000
PF4	0.643	0.397	-0.246	0.000	0.000
PF4V1	0.727	0.713	-0.014	0.820	0.827
PFAS	2.489	3.207	0.718	0.000	0.000
PFDN1	4.446	5.147	0.702	0.000	0.000
PFDN2	6.648	7.657	1.009	0.000	0.000
PFDN4	3.535	4.690	1.155	0.000	0.000
PFDN5	7.185	7.676	0.491	0.000	0.000
PFDN6	4.870	6.079	1.209	0.000	0.000
PFKFB1	5.065	4.132	-0.933	0.000	0.000
PFKFB2	1.335	2.388	1.053	0.000	0.000
PFKFB3	4.192	3.649	-0.542	0.000	0.000
PFKFB4	0.722	1.537	0.815	0.000	0.000
PFKL	5.364	5.795	0.432	0.000	0.000
PFKM	2.171	2.671	0.501	0.000	0.000
PFKP	2.144	2.593	0.450	0.000	0.000

PFN1	8.671	9.059	0.388	0.000	0.000
PFN2	3.139	3.075	-0.064	0.437	0.453
PFN4	0.612	1.283	0.671	0.000	0.000
PGAM5	4.077	4.749	0.672	0.000	0.000
PGAP1	1.011	1.511	0.501	0.000	0.000
PGAP2	3.914	4.715	0.801	0.000	0.000
PGAP3	4.712	5.019	0.307	0.000	0.000
PGBD1	1.746	2.620	0.874	0.000	0.000
PGBD2	1.969	2.425	0.456	0.000	0.000
PGBD3	0.952	1.075	0.123	0.000	0.000
PGBD4	0.711	1.031	0.320	0.000	0.000
PGBD5	1.482	1.985	0.503	0.000	0.000
PGC	0.290	1.954	1.664	0.000	0.000
PGD	5.462	6.353	0.891	0.000	0.000
PGF	1.444	2.100	0.656	0.000	0.000
PGGT1B	2.415	2.680	0.265	0.000	0.000
PGK1	6.352	7.252	0.900	0.000	0.000
PGLS	4.906	5.569	0.662	0.000	0.000
PGLYRP1	0.355	0.214	-0.141	0.000	0.000
PGLYRP2	7.666	5.389	-2.277	0.000	0.000
PGM1	7.043	6.426	-0.617	0.000	0.000
PGM2	3.646	3.977	0.330	0.000	0.000
PGM2L1	0.952	1.220	0.269	0.000	0.000
PGM3	3.843	4.206	0.363	0.000	0.000
PGM5	1.730	1.217	-0.513	0.000	0.000
PGP	3.277	4.463	1.187	0.000	0.000
PGPEP1	4.299	4.629	0.330	0.000	0.000
PGR	0.300	0.251	-0.049	0.029	0.032
PGRMC1	8.914	8.840	-0.075	0.126	0.135
PGRMC2	5.758	5.959	0.201	0.000	0.000
PGS1	2.530	3.422	0.892	0.000	0.000
PHACTR1	0.522	0.591	0.069	0.007	0.008
PHACTR2	2.360	2.400	0.040	0.375	0.390
PHACTR3	0.405	0.115	-0.289	0.000	0.000
PHACTR4	3.676	3.975	0.299	0.000	0.000
PHAX	3.757	4.588	0.831	0.000	0.000
PHB	5.801	6.700	0.900	0.000	0.000
PHB2	7.240	7.547	0.308	0.000	0.000
PHC1	2.281	2.519	0.238	0.000	0.000
PHC2	5.547	5.816	0.269	0.000	0.000
PHC3	2.159	2.551	0.392	0.000	0.000
PHEX	0.159	0.371	0.213	0.000	0.000
PHF1	4.731	5.260	0.529	0.000	0.000
PHF10	4.265	4.403	0.138	0.001	0.001
PHF11	3.632	3.984	0.352	0.000	0.000
PHF12	2.616	3.269	0.653	0.000	0.000
PHF13	3.316	3.714	0.398	0.000	0.000
PHF14	3.041	3.889	0.848	0.000	0.000
PHF19	1.550	2.533	0.983	0.000	0.000
PHF2	3.332	3.606	0.274	0.000	0.000
PHF20	2.665	3.262	0.597	0.000	0.000
PHF20L1	2.389	3.142	0.753	0.000	0.000

PHF21A	2.078	2.718	0.640	0.000	0.000
PHF23	4.341	4.761	0.420	0.000	0.000
PHF3	3.739	4.215	0.477	0.000	0.000
PHF5A	4.556	5.456	0.900	0.000	0.000
PHF6	1.998	2.902	0.904	0.000	0.000
PHF7	2.120	2.522	0.402	0.000	0.000
PHF8	3.638	3.905	0.267	0.000	0.000
PHGDH	5.972	4.616	-1.357	0.000	0.000
PHGR1	0.199	0.382	0.183	0.000	0.000
PHIP	2.550	3.180	0.630	0.000	0.000
PHKA1	1.324	1.385	0.061	0.149	0.159
PHKA2	4.683	4.937	0.255	0.000	0.000
PHKB	3.410	3.861	0.451	0.000	0.000
PHKG1	0.619	0.859	0.240	0.000	0.000
PHKG2	3.157	3.617	0.461	0.000	0.000
PHLDA1	5.470	4.145	-1.325	0.000	0.000
PHLDA2	2.757	3.568	0.810	0.000	0.000
PHLDA3	2.932	3.271	0.338	0.000	0.000
PHLDB1	1.827	2.109	0.282	0.000	0.000
PHLDB2	3.873	3.440	-0.433	0.000	0.000
PHLDB3	3.360	4.024	0.664	0.000	0.000
PHLPP1	3.269	3.328	0.059	0.152	0.163
PHLPP2	1.425	1.748	0.322	0.000	0.000
PHOSPHO1	0.583	0.385	-0.198	0.000	0.000
PHOSPHO2	1.622	2.358	0.736	0.000	0.000
PHPT1	5.823	6.903	1.079	0.000	0.000
PHRF1	4.125	4.533	0.408	0.000	0.000
PHTF1	2.000	2.379	0.379	0.000	0.000
PHTF2	1.939	2.663	0.724	0.000	0.000
PHYH	8.136	7.516	-0.620	0.000	0.000
PHYHD1	5.096	3.484	-1.612	0.000	0.000
PHYHIP	0.445	0.473	0.028	0.331	0.347
PHYHIPL	1.564	2.887	1.323	0.000	0.000
PI15	0.244	0.839	0.595	0.000	0.000
PI16	0.512	0.265	-0.247	0.000	0.000
PI3	0.738	2.048	1.310	0.000	0.000
PI4K2B	4.912	5.130	0.219	0.000	0.000
PI4KA	3.421	4.017	0.596	0.000	0.000
PI4KB	4.031	5.084	1.053	0.000	0.000
PIAS1	2.186	2.787	0.601	0.000	0.000
PIAS2	1.501	1.931	0.429	0.000	0.000
PIAS3	2.316	3.462	1.146	0.000	0.000
PIAS4	3.029	3.736	0.707	0.000	0.000
PIBF1	2.695	3.150	0.455	0.000	0.000
PICALM	5.639	6.015	0.376	0.000	0.000
PICK1	3.684	4.175	0.492	0.000	0.000
PID1	4.899	4.766	-0.133	0.020	0.022
PIF1	0.525	1.659	1.134	0.000	0.000
PIGA	2.655	3.061	0.406	0.000	0.000
PIGB	2.689	3.102	0.412	0.000	0.000
PIGC	2.955	4.410	1.454	0.000	0.000
PIGF	2.478	3.332	0.854	0.000	0.000

PIGG	2.812	3.443	0.631	0.000	0.000
PIGH	3.629	4.238	0.610	0.000	0.000
PIGK	3.439	3.925	0.486	0.000	0.000
PIGL	2.302	2.493	0.191	0.000	0.000
PIGM	2.798	3.738	0.939	0.000	0.000
PIGN	2.510	3.132	0.623	0.000	0.000
PIGO	3.269	3.896	0.627	0.000	0.000
PIGP	3.007	3.286	0.279	0.000	0.000
PIGQ	3.771	3.983	0.212	0.000	0.000
PIGR	4.946	4.563	-0.383	0.002	0.002
PIGS	2.688	3.477	0.789	0.000	0.000
PIGT	5.583	6.896	1.313	0.000	0.000
PIGU	3.307	4.693	1.386	0.000	0.000
PIGV	4.273	4.030	-0.243	0.000	0.000
PIGX	2.613	3.395	0.782	0.000	0.000
PIGZ	1.687	2.524	0.837	0.000	0.000
PIH1D2	0.668	1.007	0.339	0.000	0.000
PIK3AP1	5.330	5.389	0.059	0.264	0.279
PIK3C2A	3.247	3.693	0.446	0.000	0.000
PIK3C2B	1.941	2.942	1.001	0.000	0.000
PIK3C2G	2.881	2.243	-0.638	0.000	0.000
PIK3C3	2.375	2.774	0.399	0.000	0.000
PIK3CA	2.169	2.577	0.408	0.000	0.000
PIK3CB	2.547	3.327	0.780	0.000	0.000
PIK3CD	2.375	2.097	-0.277	0.000	0.000
PIK3CG	0.809	0.833	0.024	0.547	0.561
PIK3IP1	3.102	3.834	0.732	0.000	0.000
PIK3R1	4.787	4.620	-0.167	0.001	0.002
PIK3R2	1.508	2.706	1.199	0.000	0.000
PIK3R3	1.865	2.619	0.753	0.000	0.000
PIK3R4	4.098	4.217	0.118	0.001	0.001
PIK3R5	1.880	1.611	-0.269	0.000	0.000
PIKFYVE	2.657	3.045	0.388	0.000	0.000
PILRA	2.623	2.658	0.035	0.498	0.513
PILRB	3.360	3.680	0.320	0.000	0.000
PIM1	4.979	4.974	-0.005	0.929	0.932
PIM2	3.703	3.858	0.155	0.007	0.008
PIM3	5.834	5.702	-0.131	0.006	0.006
PIN1	4.032	4.724	0.692	0.000	0.000
PIN4	3.340	3.949	0.609	0.000	0.000
PIP4K2A	3.487	3.647	0.160	0.000	0.001
PIP4K2C	3.529	4.490	0.961	0.000	0.000
PIP5K1A	3.778	4.615	0.837	0.000	0.000
PIP5K1B	0.381	0.455	0.073	0.027	0.030
PIP5K1C	2.649	3.383	0.734	0.000	0.000
PIP5KL1	0.243	0.483	0.240	0.000	0.000
PIPOX	7.915	7.071	-0.843	0.000	0.000
PIR	3.606	4.956	1.351	0.000	0.000
PISD	3.627	3.982	0.354	0.000	0.000
PITHD1	5.204	5.476	0.271	0.000	0.000
PITPNA	4.637	4.715	0.078	0.023	0.026
PITPNB	4.711	4.991	0.279	0.000	0.000

PITPNC1	2.409	2.997	0.588	0.000	0.000
PITPNM1	3.029	3.322	0.293	0.000	0.000
PITPNM2	2.947	3.357	0.410	0.000	0.000
PITPNM3	1.112	0.421	-0.691	0.000	0.000
PITRM1	4.414	4.822	0.408	0.000	0.000
PITX1	0.044	1.543	1.499	0.000	0.000
PIWIL2	0.535	0.664	0.129	0.000	0.000
PIWIL4	1.049	1.097	0.048	0.350	0.366
PJA1	2.753	3.562	0.809	0.000	0.000
PJA2	5.425	5.654	0.228	0.000	0.000
PKD1	2.845	3.388	0.543	0.000	0.000
PKD1L1	0.162	0.333	0.172	0.000	0.000
PKD2	2.825	2.980	0.155	0.003	0.004
PKD2L1	0.633	0.954	0.322	0.000	0.000
PKDCC	2.990	3.817	0.828	0.000	0.000
PKDREJ	0.135	0.226	0.091	0.000	0.000
PKHD1	1.676	1.063	-0.613	0.000	0.000
PKHD1L1	0.476	0.301	-0.175	0.000	0.000
PKIA	0.298	0.645	0.347	0.000	0.000
PKIB	1.664	2.433	0.769	0.000	0.000
PKIG	4.843	5.054	0.211	0.000	0.000
PKLR	6.191	6.348	0.157	0.087	0.095
PKMYT1	0.863	2.411	1.548	0.000	0.000
PKN1	4.613	5.744	1.131	0.000	0.000
PKN2	3.449	3.862	0.413	0.000	0.000
PKN3	1.829	3.073	1.244	0.000	0.000
PKNOX1	2.134	2.778	0.644	0.000	0.000
PKNOX2	0.493	0.412	-0.082	0.003	0.004
PKP2	3.385	3.456	0.071	0.179	0.191
PKP3	1.036	0.676	-0.360	0.000	0.000
PKP4	3.651	4.453	0.803	0.000	0.000
PLA1A	4.932	4.727	-0.205	0.006	0.007
PLA2G10	0.119	0.261	0.142	0.000	0.000
PLA2G12A	4.386	4.230	-0.156	0.000	0.000
PLA2G12B	5.922	6.049	0.127	0.083	0.090
PLA2G15	3.645	3.602	-0.043	0.290	0.305
PLA2G16	6.029	5.155	-0.874	0.000	0.000
PLA2G1B	0.968	1.976	1.009	0.000	0.000
PLA2G2A	6.273	5.478	-0.795	0.000	0.000
PLA2G2D	0.981	1.096	0.115	0.116	0.124
PLA2G4A	1.235	1.003	-0.232	0.000	0.000
PLA2G4B	1.904	1.952	0.048	0.179	0.190
PLA2G4C	2.635	3.702	1.067	0.000	0.000
PLA2G5	2.476	1.850	-0.626	0.000	0.000
PLA2G6	2.638	3.598	0.961	0.000	0.000
PLA2G7	2.407	3.182	0.775	0.000	0.000
PLA2R1	0.918	0.607	-0.311	0.000	0.000
PLAA	3.633	4.245	0.612	0.000	0.000
PLAC8	3.185	1.293	-1.892	0.000	0.000
PLAC8L1	0.898	1.330	0.432	0.000	0.000
PLAC9	2.468	2.567	0.099	0.100	0.107
PLAG1	0.550	1.152	0.602	0.000	0.000

PLAGL1	1.294	1.328	0.034	0.511	0.526
PLAGL2	2.027	2.623	0.596	0.000	0.000
PLAT	2.136	2.302	0.166	0.010	0.011
PLAU	2.131	3.182	1.050	0.000	0.000
PLAUR	2.465	2.346	-0.120	0.098	0.105
PLB1	0.645	0.689	0.044	0.116	0.125
PLBD1	2.214	2.762	0.548	0.000	0.000
PLBD2	3.686	4.491	0.805	0.000	0.000
PLCB1	0.854	2.172	1.318	0.000	0.000
PLCB2	3.087	2.572	-0.516	0.000	0.000
PLCB3	2.576	3.374	0.799	0.000	0.000
PLCB4	0.411	0.712	0.301	0.000	0.000
PLCD1	2.739	3.039	0.300	0.000	0.000
PLCD3	1.109	1.739	0.630	0.000	0.000
PLCD4	0.637	1.109	0.472	0.000	0.000
PLCE1	0.647	1.337	0.690	0.000	0.000
PLCG1	2.955	4.021	1.066	0.000	0.000
PLCG2	3.154	2.947	-0.207	0.000	0.000
PLCH1	0.220	0.434	0.214	0.000	0.000
PLCH2	1.263	1.418	0.155	0.025	0.027
PLCL1	0.515	0.739	0.224	0.000	0.000
PLCL2	2.645	2.856	0.211	0.000	0.000
PLCXD1	1.375	1.485	0.110	0.005	0.006
PLCXD2	2.979	2.630	-0.349	0.000	0.000
PLCXD3	1.356	0.668	-0.689	0.000	0.000
PLCZ1	0.140	0.147	0.007	0.513	0.528
PLD1	3.145	2.953	-0.192	0.000	0.000
PLD2	2.939	3.334	0.395	0.000	0.000
PLD3	6.062	6.669	0.606	0.000	0.000
PLD4	0.929	0.756	-0.173	0.000	0.000
PLD6	2.161	2.564	0.403	0.000	0.000
PLEC	4.280	5.207	0.927	0.000	0.000
PLEK	3.621	3.360	-0.261	0.000	0.000
PLEK2	3.417	3.028	-0.389	0.000	0.000
PLEKHA1	2.747	3.067	0.321	0.000	0.000
PLEKHA2	2.877	2.698	-0.179	0.001	0.001
PLEKHA3	1.846	2.171	0.325	0.000	0.000
PLEKHA4	4.168	3.582	-0.586	0.000	0.000
PLEKHA5	2.546	3.132	0.585	0.000	0.000
PLEKHA6	4.014	4.336	0.322	0.000	0.000
PLEKHA7	2.646	2.759	0.113	0.019	0.021
PLEKHB1	1.415	1.356	-0.060	0.459	0.475
PLEKHB2	3.627	4.179	0.552	0.000	0.000
PLEKHF1	3.479	3.802	0.323	0.000	0.000
PLEKHF2	3.388	4.339	0.951	0.000	0.000
PLEKHG1	2.103	2.301	0.198	0.000	0.000
PLEKHG2	1.893	2.796	0.903	0.000	0.000
PLEKHG3	3.300	3.455	0.156	0.000	0.000
PLEKHG4	0.472	0.999	0.526	0.000	0.000
PLEKHG5	1.293	1.732	0.439	0.000	0.000
PLEKHG6	1.849	2.423	0.574	0.000	0.000
PLEKHG7	0.403	0.590	0.187	0.000	0.000

PLEKHH1	1.047	1.765	0.718	0.000	0.000
PLEKHH2	1.035	0.958	-0.077	0.126	0.135
PLEKHH3	3.074	3.729	0.655	0.000	0.000
PLEKHJ1	4.298	4.980	0.682	0.000	0.000
PLEKHM1	2.368	2.808	0.440	0.000	0.000
PLEKHM2	4.101	4.808	0.707	0.000	0.000
PLEKHM3	1.500	1.781	0.281	0.000	0.000
PLEKHN1	0.534	1.041	0.507	0.000	0.000
PLEKHO1	3.233	3.533	0.301	0.000	0.000
PLEKHO2	3.528	3.898	0.371	0.000	0.000
PLG	10.127	8.576	-1.551	0.000	0.000
PLGLB1	4.370	3.120	-1.250	0.000	0.000
PLGLB2	5.388	3.819	-1.569	0.000	0.000
PLIN1	3.931	2.668	-1.263	0.000	0.000
PLIN2	8.314	7.296	-1.018	0.000	0.000
PLIN3	4.252	4.993	0.741	0.000	0.000
PLIN4	4.749	4.058	-0.691	0.000	0.000
PLIN5	5.821	5.118	-0.702	0.000	0.000
PLK1	0.848	2.688	1.839	0.000	0.000
PLK2	4.572	4.476	-0.097	0.059	0.064
PLK3	3.396	2.855	-0.541	0.000	0.000
PLK4	0.641	1.713	1.072	0.000	0.000
PLLP	1.992	2.073	0.082	0.150	0.160
PLN	0.909	1.055	0.146	0.009	0.010
PLOD1	5.839	6.613	0.775	0.000	0.000
PLOD2	4.651	5.207	0.555	0.000	0.000
PLOD3	4.524	5.985	1.461	0.000	0.000
PLP2	4.193	5.606	1.413	0.000	0.000
PLRG1	4.373	4.759	0.387	0.000	0.000
PLS1	3.451	4.068	0.617	0.000	0.000
PLS3	6.436	6.760	0.324	0.000	0.000
PLSCR1	4.555	4.407	-0.148	0.013	0.014
PLSCR4	4.618	3.659	-0.959	0.000	0.000
PLTP	4.589	4.864	0.275	0.000	0.001
PLVAP	3.590	6.313	2.723	0.000	0.000
PLXDC1	0.882	1.958	1.077	0.000	0.000
PLXDC2	1.356	1.630	0.274	0.000	0.000
PLXNA1	1.774	2.689	0.915	0.000	0.000
PLXNA2	2.141	2.719	0.578	0.000	0.000
PLXNA3	1.667	2.464	0.797	0.000	0.000
PLXNA4	0.312	0.222	-0.090	0.001	0.001
PLXNB1	5.496	5.608	0.111	0.037	0.040
PLXNB2	6.375	6.708	0.333	0.000	0.000
PLXNB3	0.780	1.033	0.253	0.000	0.000
PLXNC1	1.209	2.561	1.352	0.000	0.000
PLXND1	3.930	4.847	0.917	0.000	0.000
PM20D1	0.464	0.319	-0.145	0.000	0.000
PMAIP1	1.082	1.209	0.128	0.032	0.036
PMCH	0.344	0.894	0.550	0.000	0.000
PMEL	2.232	2.132	-0.100	0.024	0.026
PMEPA1	2.261	2.553	0.293	0.001	0.001
PMF1	4.819	5.813	0.994	0.000	0.000

PMF1-BGLAP	2.834	3.688	0.854	0.000	0.000
PMFBP1	0.341	0.827	0.486	0.000	0.000
PML	2.773	3.319	0.546	0.000	0.000
PMM1	4.749	5.030	0.281	0.000	0.000
PMM2	3.034	3.421	0.388	0.000	0.000
PMP22	3.165	3.374	0.209	0.003	0.003
PMPCA	5.344	5.135	-0.209	0.000	0.000
PMPCB	5.211	5.765	0.554	0.000	0.000
PMS1	2.477	2.979	0.503	0.000	0.000
PMS2	1.687	2.458	0.771	0.000	0.000
PMVK	6.245	7.199	0.955	0.000	0.000
PNISR	4.298	4.517	0.219	0.000	0.000
PNKD	5.717	6.428	0.712	0.000	0.000
PNKP	3.714	4.433	0.719	0.000	0.000
PNLDC1	0.236	0.276	0.039	0.171	0.182
PNMA1	2.757	3.707	0.949	0.000	0.000
PNMA2	0.859	0.626	-0.233	0.000	0.000
PNMA3	0.726	1.579	0.854	0.000	0.000
PNMA6A	2.445	2.562	0.117	0.233	0.246
PNMT	0.265	0.493	0.229	0.000	0.000
PNN	5.666	6.040	0.374	0.000	0.000
PNO1	3.323	4.179	0.856	0.000	0.000
PNOC	0.435	0.290	-0.144	0.000	0.000
PNP	4.888	4.589	-0.299	0.000	0.000
PNPLA2	6.037	6.207	0.170	0.000	0.000
PNPLA3	4.135	3.904	-0.231	0.000	0.000
PNPLA4	3.948	3.979	0.031	0.397	0.414
PNPLA6	4.145	4.548	0.403	0.000	0.000
PNPLA7	3.193	2.184	-1.009	0.000	0.000
PNPLA8	3.870	4.138	0.268	0.000	0.000
PNPO	5.785	5.888	0.103	0.036	0.040
PNPT1	3.206	3.935	0.729	0.000	0.000
PNRC1	6.804	6.505	-0.299	0.000	0.000
POC1A	2.315	3.548	1.233	0.000	0.000
POC1B	1.940	2.414	0.474	0.000	0.000
POC1B-GALN	1.005	1.094	0.089	0.000	0.000
POC5	1.825	2.646	0.821	0.000	0.000
PODN	2.994	1.961	-1.033	0.000	0.000
PODNL1	0.238	0.514	0.276	0.000	0.000
PODXL	2.369	3.845	1.476	0.000	0.000
PODXL2	1.370	2.046	0.676	0.000	0.000
POF1B	0.933	1.192	0.259	0.001	0.001
POFUT1	4.442	5.195	0.752	0.000	0.000
POFUT2	3.320	3.675	0.355	0.000	0.000
POGK	2.999	4.094	1.095	0.000	0.000
POGLUT1	2.323	2.740	0.417	0.000	0.000
POGZ	3.760	4.644	0.884	0.000	0.000
POLA1	1.549	2.408	0.859	0.000	0.000
POLA2	1.829	3.000	1.171	0.000	0.000
POLB	3.667	4.161	0.494	0.000	0.000
POLD1	2.489	3.870	1.382	0.000	0.000
POLD2	5.729	6.291	0.562	0.000	0.000

POLD3	2.148	2.943	0.795	0.000	0.000
POLD4	5.657	6.000	0.342	0.000	0.000
POLDIP2	7.264	7.815	0.551	0.000	0.000
POLDIP3	4.359	5.219	0.859	0.000	0.000
POLE	3.075	3.399	0.324	0.000	0.000
POLE2	1.154	2.566	1.412	0.000	0.000
POLE3	4.580	5.428	0.848	0.000	0.000
POLE4	4.581	4.942	0.361	0.000	0.000
POLG	4.017	4.494	0.477	0.000	0.000
POLG2	2.209	3.246	1.036	0.000	0.000
POLH	2.550	3.323	0.773	0.000	0.000
POLI	1.992	2.404	0.412	0.000	0.000
POLK	2.362	2.755	0.393	0.000	0.000
POLL	3.181	3.755	0.574	0.000	0.000
POLM	3.162	3.843	0.681	0.000	0.000
POLN	0.511	0.858	0.348	0.000	0.000
POLQ	0.281	1.141	0.860	0.000	0.000
POLR1A	1.948	2.789	0.841	0.000	0.000
POLR1B	3.013	3.498	0.484	0.000	0.000
POLR1C	3.808	4.561	0.753	0.000	0.000
POLR1D	4.060	4.778	0.719	0.000	0.000
POLR1E	4.627	4.190	-0.437	0.000	0.000
POLR2A	4.351	4.574	0.223	0.000	0.000
POLR2B	4.323	4.899	0.576	0.000	0.000
POLR2C	4.943	5.300	0.357	0.000	0.000
POLR2D	3.076	3.895	0.819	0.000	0.000
POLR2E	6.099	6.482	0.383	0.000	0.000
POLR2F	3.227	3.589	0.362	0.000	0.000
POLR2G	5.026	5.981	0.955	0.000	0.000
POLR2H	4.761	5.516	0.755	0.000	0.000
POLR2J	5.339	6.096	0.757	0.000	0.000
POLR2J2	1.465	1.883	0.418	0.000	0.000
POLR2J3	1.393	1.812	0.420	0.000	0.000
POLR2K	5.345	6.676	1.331	0.000	0.000
POLR2L	6.870	7.569	0.698	0.000	0.000
POLR3A	2.277	3.000	0.723	0.000	0.000
POLR3B	2.832	3.176	0.344	0.000	0.000
POLR3C	3.362	4.422	1.060	0.000	0.000
POLR3D	2.133	2.535	0.401	0.000	0.000
POLR3E	3.004	3.519	0.515	0.000	0.000
POLR3F	2.484	3.376	0.893	0.000	0.000
POLR3G	1.225	1.786	0.561	0.000	0.000
POLR3GL	5.313	5.931	0.618	0.000	0.000
POLR3H	3.955	4.479	0.524	0.000	0.000
POLR3K	3.026	3.844	0.818	0.000	0.000
POLRMT	4.748	5.333	0.585	0.000	0.000
POMC	0.747	0.704	-0.043	0.191	0.203
POMGNT1	4.423	5.055	0.632	0.000	0.000
POMP	6.605	7.133	0.528	0.000	0.000
POMT1	3.629	4.122	0.492	0.000	0.000
POMT2	1.922	2.606	0.685	0.000	0.000
PON1	8.942	7.402	-1.539	0.000	0.000

PON2	6.354	7.305	0.952	0.000	0.000
PON3	7.688	6.496	-1.191	0.000	0.000
POP1	1.439	2.098	0.659	0.000	0.000
POP4	3.494	4.397	0.902	0.000	0.000
POP5	4.371	5.195	0.824	0.000	0.000
POP7	5.542	6.135	0.593	0.000	0.000
POPDC2	1.099	1.420	0.321	0.000	0.000
POR	8.151	7.969	-0.182	0.000	0.000
PORCN	1.411	2.337	0.925	0.000	0.000
POSTN	2.045	2.659	0.614	0.000	0.000
POT1	2.624	3.236	0.612	0.000	0.000
POU2AF1	1.185	0.926	-0.259	0.000	0.000
POU2F1	1.677	2.387	0.711	0.000	0.000
POU2F2	1.075	0.864	-0.211	0.000	0.000
POU2F3	0.155	0.360	0.205	0.000	0.000
POU3F1	0.134	0.183	0.049	0.004	0.005
POU5F1	1.049	1.829	0.781	0.000	0.000
POU5F1B	0.242	0.217	-0.025	0.033	0.037
POU6F1	1.878	1.789	-0.089	0.026	0.029
PPA1	5.429	6.171	0.741	0.000	0.000
PPA2	4.889	5.061	0.172	0.000	0.000
PPAN	3.127	3.763	0.636	0.000	0.000
PPARA	4.466	4.398	-0.068	0.167	0.178
PPARD	3.638	4.295	0.656	0.000	0.000
PPARG	2.972	3.921	0.949	0.000	0.000
PPARGC1A	4.157	3.448	-0.709	0.000	0.000
PPARGC1B	1.097	1.194	0.097	0.002	0.002
PPAT	1.980	2.774	0.794	0.000	0.000
PPBP	0.908	0.392	-0.516	0.000	0.000
PPCDC	2.415	3.221	0.806	0.000	0.000
PPCS	5.531	5.776	0.245	0.000	0.000
PPDPF	6.498	7.310	0.812	0.000	0.000
PPEF1	0.090	0.308	0.218	0.000	0.000
PPFIA1	3.184	3.940	0.756	0.000	0.000
PPFIA2	0.192	0.357	0.165	0.000	0.000
PPFIA3	1.568	2.430	0.861	0.000	0.000
PPFIA4	0.122	0.417	0.295	0.000	0.000
PPFIBP1	3.255	2.952	-0.302	0.000	0.000
PPFIBP2	3.909	3.748	-0.161	0.000	0.000
PPHLN1	2.566	3.358	0.792	0.000	0.000
PPIC	4.560	5.378	0.818	0.000	0.000
PPID	5.577	5.403	-0.173	0.000	0.000
PPIE	3.286	3.668	0.381	0.000	0.000
PPIF	6.834	6.691	-0.143	0.000	0.000
PPIG	4.628	4.928	0.300	0.000	0.000
PPIH	3.676	4.708	1.032	0.000	0.000
PPIL1	4.125	5.223	1.097	0.000	0.000
PPIL2	3.443	4.223	0.780	0.000	0.000
PPIL3	3.747	4.297	0.550	0.000	0.000
PPIL4	3.965	4.151	0.186	0.000	0.000
PPIL6	0.414	0.671	0.257	0.000	0.000
PPIP5K1	1.417	2.254	0.837	0.000	0.000

PPIP5K2	2.549	3.036	0.487	0.000	0.000
PPL	3.365	2.824	-0.541	0.000	0.000
PPM1A	3.990	4.214	0.224	0.000	0.000
PPM1B	3.821	4.261	0.440	0.000	0.000
PPM1D	2.358	2.913	0.554	0.000	0.000
PPM1E	0.414	0.688	0.273	0.000	0.000
PPM1F	2.179	3.277	1.098	0.000	0.000
PPM1G	4.908	5.965	1.057	0.000	0.000
PPM1H	1.898	1.989	0.090	0.178	0.189
PPM1J	0.665	0.779	0.114	0.000	0.000
PPM1K	1.826	1.422	-0.404	0.000	0.000
PPM1L	1.137	1.530	0.393	0.000	0.000
PPM1M	2.841	3.475	0.634	0.000	0.000
PPM1N	0.946	0.998	0.052	0.167	0.178
PPME1	2.750	3.619	0.869	0.000	0.000
PPOX	2.692	3.927	1.235	0.000	0.000
PPP1CA	6.189	7.034	0.845	0.000	0.000
PPP1CB	5.254	5.891	0.637	0.000	0.000
PPP1CC	4.510	5.551	1.041	0.000	0.000
PPP1R10	5.029	5.347	0.318	0.000	0.000
PPP1R11	5.134	6.098	0.964	0.000	0.000
PPP1R12A	3.079	3.476	0.397	0.000	0.000
PPP1R12B	2.015	2.196	0.181	0.000	0.000
PPP1R12C	4.401	4.963	0.562	0.000	0.000
PPP1R13B	2.928	3.138	0.211	0.000	0.000
PPP1R13L	2.463	3.288	0.825	0.000	0.000
PPP1R14A	3.053	3.161	0.108	0.072	0.078
PPP1R14B	5.368	6.403	1.035	0.000	0.000
PPP1R14D	0.214	0.875	0.661	0.000	0.000
PPP1R15A	5.401	5.090	-0.311	0.000	0.000
PPP1R15B	5.008	5.461	0.453	0.000	0.000
PPP1R16A	4.767	5.906	1.140	0.000	0.000
PPP1R16B	1.744	1.706	-0.038	0.435	0.451
PPP1R1A	5.741	4.389	-1.352	0.000	0.000
PPP1R1C	1.780	1.469	-0.311	0.000	0.000
PPP1R2	3.496	4.170	0.674	0.000	0.000
PPP1R3B	6.053	5.026	-1.028	0.000	0.000
PPP1R3C	5.495	5.322	-0.173	0.019	0.021
PPP1R3D	1.189	1.412	0.223	0.000	0.000
PPP1R3E	2.512	3.050	0.537	0.000	0.000
PPP1R3F	1.518	2.014	0.496	0.000	0.000
PPP1R3G	2.538	2.579	0.041	0.662	0.675
PPP1R7	4.728	5.385	0.658	0.000	0.000
PPP1R8	4.020	4.672	0.652	0.000	0.000
PPP1R9A	0.555	1.064	0.509	0.000	0.000
PPP1R9B	3.908	4.263	0.355	0.000	0.000
PPP2CA	5.103	5.773	0.670	0.000	0.000
PPP2CB	5.350	5.251	-0.099	0.011	0.012
PPP2R1A	5.175	6.174	0.999	0.000	0.000
PPP2R1B	5.240	5.317	0.077	0.178	0.190
PPP2R2A	3.580	3.442	-0.138	0.000	0.000
PPP2R2B	0.359	0.450	0.091	0.004	0.004

PPP2R2D	3.223	3.392	0.169	0.000	0.000
PPP2R3A	0.824	1.011	0.187	0.000	0.000
PPP2R3C	3.047	3.670	0.623	0.000	0.000
PPP2R5A	5.145	6.271	1.126	0.000	0.000
PPP2R5B	3.010	3.672	0.662	0.000	0.000
PPP2R5C	3.682	4.117	0.435	0.000	0.000
PPP2R5D	4.180	5.207	1.027	0.000	0.000
PPP2R5E	2.757	3.142	0.385	0.000	0.000
PPP3CA	3.463	3.586	0.123	0.000	0.000
PPP3CB	3.556	4.101	0.546	0.000	0.000
PPP3CC	3.216	3.394	0.178	0.000	0.000
PPP3R1	4.947	5.329	0.382	0.000	0.000
PPP4C	5.981	6.438	0.457	0.000	0.000
PPP4R1	3.264	4.214	0.950	0.000	0.000
PPP4R2	4.026	4.560	0.534	0.000	0.000
PPP4R4	1.591	1.834	0.243	0.000	0.000
PPP5C	3.891	4.478	0.588	0.000	0.000
PPP6C	4.310	4.834	0.524	0.000	0.000
PPP6R1	4.022	5.036	1.014	0.000	0.000
PPP6R2	5.635	6.245	0.610	0.000	0.000
PPP6R3	4.122	4.760	0.638	0.000	0.000
PPRC1	3.409	3.798	0.389	0.000	0.000
PPT1	4.644	5.596	0.952	0.000	0.000
PPT2	3.111	3.710	0.599	0.000	0.000
PPT2-EGFL8	1.441	1.796	0.356	0.000	0.000
PPTC7	3.268	3.597	0.329	0.000	0.000
PPWD1	3.421	4.019	0.599	0.000	0.000
PQBP1	5.659	6.390	0.731	0.000	0.000
PQLC1	6.039	5.992	-0.047	0.214	0.227
PQLC2	3.965	4.450	0.485	0.000	0.000
PQLC3	3.578	3.857	0.280	0.000	0.000
PRADC1	5.292	5.654	0.362	0.000	0.000
PRAF2	3.171	3.996	0.825	0.000	0.000
PRAM1	1.666	0.989	-0.676	0.000	0.000
PRAMEF10	0.578	0.898	0.320	0.000	0.000
PRAP1	8.068	8.193	0.125	0.180	0.191
PRC1	1.124	3.678	2.554	0.000	0.000
PRCC	4.385	5.674	1.289	0.000	0.000
PRCD	1.003	0.774	-0.229	0.000	0.000
PRCP	4.632	4.630	-0.002	0.961	0.963
PRDM1	1.705	1.776	0.071	0.168	0.180
PRDM10	1.778	2.292	0.514	0.000	0.000
PRDM11	0.573	0.657	0.084	0.000	0.000
PRDM12	0.104	0.253	0.149	0.000	0.000
PRDM15	1.068	1.710	0.642	0.000	0.000
PRDM16	0.364	0.524	0.161	0.000	0.000
PRDM2	2.483	2.494	0.011	0.770	0.779
PRDM4	2.656	3.395	0.738	0.000	0.000
PRDM5	0.216	0.297	0.081	0.000	0.000
PRDM6	0.226	0.302	0.077	0.000	0.000
PRDM8	0.449	0.384	-0.065	0.010	0.012
PRDX1	8.526	9.369	0.843	0.000	0.000

PRDX2	7.367	7.787	0.420	0.000	0.000
PRDX3	7.692	7.675	-0.017	0.625	0.639
PRDX4	7.292	7.418	0.127	0.002	0.002
PRDX5	8.471	9.106	0.635	0.000	0.000
PRDX6	8.961	9.009	0.047	0.164	0.175
PREB	5.460	6.393	0.933	0.000	0.000
PRELID1	6.027	6.926	0.898	0.000	0.000
PRELID2	0.704	1.226	0.521	0.000	0.000
PRELP	3.402	2.237	-1.164	0.000	0.000
PREP	3.801	4.199	0.398	0.000	0.000
PREPL	3.215	3.802	0.587	0.000	0.000
PREX1	2.892	3.376	0.484	0.000	0.000
PREX2	0.708	0.841	0.133	0.000	0.000
PRF1	2.939	2.462	-0.477	0.000	0.000
PRG2	1.432	1.201	-0.232	0.000	0.000
PRG4	6.708	5.426	-1.281	0.000	0.000
PRH1	0.387	0.424	0.037	0.026	0.029
PRH2	0.931	1.124	0.193	0.000	0.000
PRICKLE1	0.922	0.697	-0.225	0.000	0.000
PRICKLE2	1.519	1.380	-0.139	0.004	0.005
PRICKLE3	1.909	2.446	0.537	0.000	0.000
PRICKLE4	2.483	2.638	0.155	0.001	0.001
PRIM1	2.041	3.508	1.468	0.000	0.000
PRIM2	1.399	2.602	1.203	0.000	0.000
PRKAA1	4.680	4.861	0.181	0.000	0.000
PRKAA2	1.094	2.254	1.161	0.000	0.000
PRKAB1	3.297	4.039	0.742	0.000	0.000
PRKAB2	4.346	5.479	1.132	0.000	0.000
PRKACA	5.207	5.419	0.212	0.000	0.000
PRKACB	3.244	3.543	0.299	0.000	0.000
PRKAG1	4.186	5.027	0.841	0.000	0.000
PRKAG2	4.002	3.549	-0.453	0.000	0.000
PRKAR1A	5.873	6.519	0.647	0.000	0.000
PRKAR1B	3.389	3.329	-0.061	0.235	0.248
PRKAR2A	3.010	3.761	0.751	0.000	0.000
PRKAR2B	1.766	0.985	-0.781	0.000	0.000
PRKCA	2.619	3.471	0.852	0.000	0.000
PRKCB	1.303	0.992	-0.311	0.000	0.000
PRKCD	3.164	4.067	0.903	0.000	0.000
PRKCE	1.866	2.109	0.244	0.000	0.000
PRKCH	2.333	2.284	-0.049	0.266	0.280
PRKCI	2.521	3.360	0.839	0.000	0.000
PRKCQ	1.082	0.924	-0.158	0.000	0.000
PRKCSH	6.714	7.466	0.752	0.000	0.000
PRKCZ	2.614	2.767	0.153	0.001	0.001
PRKD1	1.115	1.493	0.378	0.000	0.000
PRKD2	3.622	4.440	0.818	0.000	0.000
PRKD3	2.408	2.858	0.450	0.000	0.000
PRKDC	3.327	4.669	1.343	0.000	0.000
PRKG1	0.867	0.758	-0.109	0.001	0.001
PRKRA	3.910	4.087	0.177	0.000	0.000
PRKRIP1	3.457	4.380	0.923	0.000	0.000

PRKX	1.817	1.939	0.122	0.029	0.032
PRLR	2.199	2.903	0.704	0.000	0.000
PRMT1	4.571	5.433	0.862	0.000	0.000
PRMT2	3.149	3.676	0.527	0.000	0.000
PRMT3	2.037	3.022	0.985	0.000	0.000
PRMT6	2.937	3.191	0.254	0.000	0.000
PRMT7	2.836	3.304	0.468	0.000	0.000
PRND	0.169	0.471	0.302	0.000	0.000
PRNP	5.692	5.180	-0.512	0.000	0.000
PROC	8.674	8.110	-0.564	0.000	0.000
PROCA1	0.649	1.201	0.552	0.000	0.000
PROCR	3.034	3.098	0.064	0.325	0.341
PRODH	3.035	2.447	-0.588	0.000	0.000
PRODH2	6.783	5.717	-1.066	0.000	0.000
PROK1	0.218	0.399	0.181	0.000	0.000
PROK2	0.497	0.231	-0.266	0.000	0.000
PROM1	1.459	0.738	-0.721	0.000	0.000
PROM2	0.726	0.460	-0.266	0.000	0.000
PROS1	7.298	6.678	-0.620	0.000	0.000
PROX1	4.664	4.614	-0.050	0.440	0.456
PROZ	6.284	4.334	-1.950	0.000	0.000
PRPF18	3.524	4.086	0.562	0.000	0.000
PRPF19	5.730	6.476	0.747	0.000	0.000
PRPF3	3.966	4.870	0.904	0.000	0.000
PRPF31	4.949	5.798	0.848	0.000	0.000
PRPF38A	3.392	4.089	0.697	0.000	0.000
PRPF38B	4.214	4.527	0.314	0.000	0.000
PRPF39	3.139	3.797	0.659	0.000	0.000
PRPF4	3.513	4.180	0.667	0.000	0.000
PRPF40A	3.896	4.586	0.690	0.000	0.000
PRPF40B	1.598	2.296	0.698	0.000	0.000
PRPF4B	3.956	4.643	0.687	0.000	0.000
PRPF6	5.535	6.491	0.956	0.000	0.000
PRPF8	5.139	5.445	0.306	0.000	0.000
PRPH2	0.289	0.385	0.096	0.000	0.000
PRPS1	4.957	5.474	0.516	0.000	0.000
PRPS2	4.171	4.525	0.354	0.000	0.000
PRPSAP1	4.413	5.342	0.929	0.000	0.000
PRPSAP2	3.440	3.837	0.397	0.000	0.000
PRR11	0.760	2.366	1.605	0.000	0.000
PRR12	2.221	3.044	0.823	0.000	0.000
PRR14	3.973	4.571	0.598	0.000	0.000
PRR14L	2.421	3.039	0.618	0.000	0.000
PRR15	0.553	1.131	0.578	0.000	0.000
PRR15L	1.842	2.424	0.582	0.000	0.000
PRR16	0.572	0.939	0.366	0.000	0.000
PRR18	2.034	1.512	-0.522	0.000	0.000
PRR19	0.507	1.326	0.819	0.000	0.000
PRR22	2.347	1.988	-0.358	0.000	0.000
PRR3	2.383	3.254	0.871	0.000	0.000
PRR4	1.164	1.469	0.305	0.000	0.000
PRR5	3.664	3.295	-0.369	0.000	0.000

PRR5-ARHGA	0.693	0.685	-0.007	0.778	0.787
PRR5L	1.265	1.711	0.446	0.000	0.000
PRR7	1.238	2.324	1.086	0.000	0.000
PRRC1	3.651	4.559	0.909	0.000	0.000
PRRC2A	4.806	5.698	0.891	0.000	0.000
PRRC2B	3.815	4.462	0.647	0.000	0.000
PRRC2C	4.281	5.138	0.857	0.000	0.000
PRRG1	2.274	2.339	0.065	0.065	0.071
PRRG2	2.680	2.344	-0.336	0.000	0.000
PRRG4	3.910	3.545	-0.365	0.000	0.000
PRRT1	1.262	1.091	-0.171	0.000	0.000
PRRT2	0.705	1.076	0.371	0.000	0.000
PRRT3	0.615	1.128	0.513	0.000	0.000
PRRT4	0.188	0.405	0.218	0.000	0.000
PRRX1	0.419	1.015	0.595	0.000	0.000
PRRX2	0.110	0.420	0.311	0.000	0.000
PRSS12	0.488	0.685	0.196	0.000	0.000
PRSS16	0.336	0.550	0.215	0.000	0.000
PRSS21	0.337	0.367	0.030	0.501	0.516
PRSS22	1.494	0.654	-0.839	0.000	0.000
PRSS23	2.669	3.044	0.375	0.000	0.000
PRSS27	0.234	0.492	0.258	0.000	0.000
PRSS3	2.178	2.420	0.242	0.040	0.044
PRSS35	0.589	0.795	0.206	0.000	0.000
PRSS36	0.978	1.212	0.234	0.000	0.000
PRSS42	0.218	0.266	0.048	0.001	0.001
PRSS45	0.514	0.471	-0.043	0.120	0.129
PRSS50	1.008	0.975	-0.034	0.468	0.484
PRSS53	1.526	1.241	-0.286	0.000	0.000
PRSS8	4.103	2.431	-1.673	0.000	0.000
PRTFDC1	1.395	2.330	0.935	0.000	0.000
PRTG	0.103	0.250	0.147	0.000	0.000
PRUNE2	0.784	0.620	-0.163	0.000	0.000
PRX	0.999	1.526	0.527	0.000	0.000
PSAP	9.167	9.770	0.602	0.000	0.000
PSAT1	6.973	6.187	-0.786	0.000	0.000
PSCA	0.120	0.417	0.297	0.000	0.000
PSD	1.054	1.173	0.119	0.000	0.000
PSD2	0.116	0.216	0.100	0.000	0.000
PSD3	3.055	2.809	-0.246	0.000	0.000
PSD4	4.312	4.106	-0.207	0.000	0.000
PSEN1	3.492	4.127	0.635	0.000	0.000
PSEN2	3.339	4.182	0.843	0.000	0.000
PSENEN	4.808	5.625	0.817	0.000	0.000
PSIP1	3.510	4.147	0.637	0.000	0.000
PSKH1	3.059	4.007	0.947	0.000	0.000
PSMA1	6.321	7.095	0.773	0.000	0.000
PSMA2	5.485	5.684	0.199	0.000	0.000
PSMA3	5.867	6.429	0.562	0.000	0.000
PSMA4	5.239	5.966	0.727	0.000	0.000
PSMA5	5.467	6.013	0.546	0.000	0.000
PSMA6	4.069	4.921	0.851	0.000	0.000

PSMA7	7.376	8.091	0.716	0.000	0.000
PSMB1	7.014	7.505	0.491	0.000	0.000
PSMB10	5.201	5.400	0.198	0.000	0.000
PSMB2	5.351	6.053	0.702	0.000	0.000
PSMB4	7.359	8.602	1.244	0.000	0.000
PSMB5	6.100	7.059	0.959	0.000	0.000
PSMB6	6.826	7.113	0.287	0.000	0.000
PSMB7	6.988	7.516	0.528	0.000	0.000
PSMB8	6.344	6.777	0.434	0.000	0.000
PSMB9	4.763	5.395	0.632	0.000	0.000
PSMC1	3.879	4.354	0.475	0.000	0.000
PSMC2	5.694	6.627	0.932	0.000	0.000
PSMC3	6.476	7.261	0.785	0.000	0.000
PSMC3IP	1.955	2.850	0.895	0.000	0.000
PSMC4	5.697	6.849	1.152	0.000	0.000
PSMC5	5.731	6.488	0.757	0.000	0.000
PSMC6	4.340	4.944	0.604	0.000	0.000
PSMD1	5.027	5.886	0.859	0.000	0.000
PSMD10	4.088	5.280	1.192	0.000	0.000
PSMD11	4.228	5.129	0.902	0.000	0.000
PSMD12	4.354	4.937	0.584	0.000	0.000
PSMD13	5.316	6.102	0.786	0.000	0.000
PSMD14	3.582	4.706	1.124	0.000	0.000
PSMD2	5.184	6.346	1.162	0.000	0.000
PSMD3	5.314	6.119	0.805	0.000	0.000
PSMD4	6.213	7.712	1.500	0.000	0.000
PSMD5	3.761	4.140	0.379	0.000	0.000
PSMD6	4.035	4.562	0.527	0.000	0.000
PSMD7	5.435	6.006	0.571	0.000	0.000
PSMD8	6.007	6.800	0.793	0.000	0.000
PSMD9	2.878	3.483	0.605	0.000	0.000
PSME1	7.552	8.161	0.609	0.000	0.000
PSME2	6.517	7.163	0.646	0.000	0.000
PSME3	4.207	5.412	1.204	0.000	0.000
PSME4	4.246	4.753	0.507	0.000	0.000
PSMF1	5.956	6.352	0.396	0.000	0.000
PSMG1	4.366	4.841	0.475	0.000	0.000
PSMG2	4.714	5.359	0.645	0.000	0.000
PSMG3	3.835	5.112	1.277	0.000	0.000
PSMG4	2.290	2.885	0.595	0.000	0.000
PSORS1C1	0.200	0.467	0.266	0.000	0.000
PSPC1	3.438	4.131	0.694	0.000	0.000
PSPH	2.666	4.328	1.662	0.000	0.000
PSPN	0.805	0.891	0.086	0.000	0.000
PSRC1	1.188	2.588	1.401	0.000	0.000
PSTK	1.374	1.920	0.546	0.000	0.000
PSTPIP1	1.924	1.794	-0.130	0.022	0.024
PSTPIP2	3.187	3.402	0.215	0.000	0.000
PTAFR	1.954	2.030	0.076	0.210	0.223
PTAR1	2.927	3.319	0.392	0.000	0.000
PTBP1	5.570	6.335	0.765	0.000	0.000
PTBP2	1.726	2.319	0.593	0.000	0.000

PTCD1	2.106	3.139	1.033	0.000	0.000
PTCD2	1.215	1.534	0.319	0.000	0.000
PTCD3	3.799	4.319	0.520	0.000	0.000
PTCH1	1.126	1.255	0.129	0.001	0.001
PTCH2	0.859	0.830	-0.030	0.347	0.362
PTCRA	0.514	0.403	-0.111	0.000	0.000
PTDSS1	4.455	5.251	0.796	0.000	0.000
PTDSS2	2.885	3.645	0.760	0.000	0.000
PTEN	4.101	4.296	0.195	0.000	0.000
PTER	3.132	3.354	0.222	0.000	0.000
PTGDR	0.847	0.543	-0.304	0.000	0.000
PTGDS	5.221	3.999	-1.222	0.000	0.000
PTGER1	0.647	0.614	-0.033	0.385	0.401
PTGER2	1.467	1.072	-0.394	0.000	0.000
PTGER3	0.209	0.334	0.125	0.000	0.000
PTGER4	1.997	1.914	-0.082	0.146	0.156
PTGES	0.833	1.488	0.654	0.000	0.000
PTGES2	4.406	5.377	0.971	0.000	0.000
PTGES3	6.738	7.757	1.020	0.000	0.000
PTGFR	0.970	1.684	0.714	0.000	0.000
PTGFRN	3.095	4.180	1.086	0.000	0.000
PTGIR	2.265	1.532	-0.733	0.000	0.000
PTGIS	2.371	1.427	-0.944	0.000	0.000
PTGR1	7.682	7.279	-0.403	0.000	0.000
PTGR2	2.815	3.273	0.458	0.000	0.000
PTGS1	1.803	1.607	-0.196	0.000	0.001
PTGS2	1.675	0.946	-0.729	0.000	0.000
PTH1R	4.748	1.855	-2.893	0.000	0.000
PTH2R	0.430	1.260	0.830	0.000	0.000
PTHLH	0.276	0.999	0.723	0.000	0.000
PTK2	3.232	4.426	1.194	0.000	0.000
PTK2B	3.231	3.044	-0.187	0.000	0.000
PTK6	1.895	1.879	-0.016	0.827	0.834
PTK7	1.080	1.979	0.899	0.000	0.000
PTMA	7.954	8.791	0.837	0.000	0.000
PTMS	9.886	9.393	-0.494	0.000	0.000
PTN	2.660	1.890	-0.769	0.000	0.000
PTP4A1	8.037	7.977	-0.060	0.192	0.204
PTP4A2	5.307	5.745	0.438	0.000	0.000
PTP4A3	2.193	3.972	1.779	0.000	0.000
PTPDC1	0.976	1.757	0.780	0.000	0.000
PTPN1	4.007	4.731	0.724	0.000	0.000
PTPN11	4.600	5.208	0.608	0.000	0.000
PTPN12	4.056	4.837	0.782	0.000	0.000
PTPN13	1.116	0.704	-0.412	0.000	0.000
PTPN14	0.782	1.459	0.678	0.000	0.000
PTPN18	4.307	4.823	0.516	0.000	0.000
PTPN2	3.179	3.607	0.428	0.000	0.000
PTPN21	2.615	2.680	0.065	0.097	0.105
PTPN22	1.530	1.298	-0.232	0.000	0.000
PTPN23	3.561	4.403	0.842	0.000	0.000
PTPN3	4.021	3.818	-0.203	0.000	0.000

PTPN4	1.797	2.046	0.249	0.000	0.000
PTPN6	4.381	4.626	0.246	0.000	0.000
PTPN7	1.650	1.753	0.104	0.070	0.076
PTPN9	2.817	3.249	0.432	0.000	0.000
PTPRA	3.986	4.849	0.862	0.000	0.000
PTPRB	2.627	2.219	-0.408	0.000	0.000
PTPRC	3.470	2.987	-0.483	0.000	0.000
PTPRCAP	2.698	2.484	-0.214	0.000	0.000
PTPRD	1.831	1.498	-0.332	0.000	0.000
PTPRE	1.632	1.755	0.123	0.014	0.016
PTPRF	5.351	6.249	0.898	0.000	0.000
PTPRG	1.961	2.805	0.844	0.000	0.000
PTPRH	3.764	4.080	0.316	0.000	0.000
PTPRJ	2.813	3.472	0.659	0.000	0.000
PTPRK	3.963	4.474	0.511	0.000	0.000
PTPRM	3.296	3.646	0.350	0.000	0.000
PTPRN2	1.569	1.302	-0.267	0.000	0.000
PTPRO	0.657	0.711	0.055	0.083	0.089
PTPRR	0.087	0.288	0.201	0.000	0.000
PTPRS	2.320	1.536	-0.785	0.000	0.000
PTPRU	2.573	2.933	0.360	0.000	0.000
PTRH2	3.596	4.382	0.786	0.000	0.000
PTRHD1	3.985	4.473	0.489	0.000	0.000
PTS	4.831	4.567	-0.265	0.000	0.000
PTTG1	1.482	4.408	2.926	0.000	0.000
PTTG1IP	6.554	7.050	0.496	0.000	0.000
PTX3	0.749	0.850	0.101	0.065	0.071
PUF60	5.466	6.625	1.159	0.000	0.000
PUM1	4.173	4.665	0.492	0.000	0.000
PUM2	4.790	5.396	0.606	0.000	0.000
PURA	2.714	2.969	0.256	0.000	0.000
PURB	3.031	3.719	0.688	0.000	0.000
PUS1	3.123	4.059	0.937	0.000	0.000
PUS10	2.655	2.495	-0.160	0.000	0.000
PUS3	4.513	4.676	0.163	0.000	0.000
PUS7	2.286	3.291	1.004	0.000	0.000
PUS7L	1.200	1.633	0.433	0.000	0.000
PUSL1	2.766	3.671	0.905	0.000	0.000
PVALB	1.943	0.361	-1.582	0.000	0.000
PVR	5.014	5.613	0.600	0.000	0.000
PWP1	4.446	5.246	0.799	0.000	0.000
PWP2	1.895	2.286	0.390	0.000	0.000
PWWP2A	2.104	2.713	0.609	0.000	0.000
PWWP2B	2.617	3.565	0.948	0.000	0.000
PXDN	2.686	2.600	-0.087	0.115	0.124
PXDNL	0.116	0.390	0.274	0.000	0.000
PXK	2.204	2.922	0.719	0.000	0.000
PXMP2	7.108	6.424	-0.684	0.000	0.000
PXMP4	2.533	3.613	1.080	0.000	0.000
PXN	4.052	4.634	0.583	0.000	0.000
PXT1	0.095	0.135	0.040	0.000	0.000
PYCARD	3.674	4.180	0.506	0.000	0.000

PYCR1	1.880	3.323	1.443	0.000	0.000
PYCR2	4.186	5.385	1.198	0.000	0.000
PYGB	3.588	5.134	1.546	0.000	0.000
PYGL	6.134	6.173	0.039	0.407	0.423
PYGM	0.502	0.512	0.010	0.688	0.700
PYGO2	3.722	5.215	1.493	0.000	0.000
PYHIN1	1.487	1.168	-0.319	0.000	0.000
PYROXD1	2.907	3.470	0.562	0.000	0.000
PYROXD2	3.413	2.328	-1.084	0.000	0.000
PYY	0.290	0.358	0.069	0.011	0.012
PZP	2.952	0.945	-2.007	0.000	0.000
QARS	5.658	6.681	1.022	0.000	0.000
QDPR	7.021	6.411	-0.610	0.000	0.000
QKI	3.430	3.758	0.328	0.000	0.000
QPCT	1.217	1.893	0.677	0.000	0.000
QPCTL	3.110	4.086	0.977	0.000	0.000
QPRT	6.667	6.869	0.202	0.000	0.000
QRICH1	4.117	4.751	0.634	0.000	0.000
QRICH2	1.316	2.074	0.758	0.000	0.000
QRSL1	2.751	3.351	0.600	0.000	0.000
QSER1	2.124	2.789	0.665	0.000	0.000
QSOX1	4.288	3.813	-0.475	0.000	0.000
QSOX2	2.753	3.592	0.839	0.000	0.000
QTRT1	4.710	5.254	0.544	0.000	0.000
R3HCC1	4.396	4.574	0.178	0.000	0.000
R3HDM1	2.239	3.175	0.936	0.000	0.000
R3HDM2	4.040	4.509	0.469	0.000	0.000
R3HDML	0.320	0.351	0.031	0.174	0.185
RAB10	5.300	6.335	1.035	0.000	0.000
RAB11A	5.057	5.832	0.775	0.000	0.000
RAB11B	6.213	6.599	0.386	0.000	0.000
RAB11FIP1	2.177	2.962	0.785	0.000	0.000
RAB11FIP2	2.254	2.804	0.551	0.000	0.000
RAB11FIP3	3.573	4.073	0.499	0.000	0.000
RAB11FIP4	1.554	2.963	1.409	0.000	0.000
RAB11FIP5	2.744	3.063	0.318	0.000	0.000
RAB12	4.764	5.009	0.245	0.000	0.000
RAB13	5.759	6.579	0.821	0.000	0.000
RAB14	5.748	5.923	0.175	0.000	0.000
RAB15	3.114	3.875	0.761	0.000	0.000
RAB17	5.992	5.384	-0.607	0.000	0.000
RAB18	4.635	5.054	0.419	0.000	0.000
RAB19	0.283	0.319	0.035	0.205	0.217
RAB1A	6.556	7.206	0.650	0.000	0.000
RAB20	5.454	5.092	-0.362	0.000	0.000
RAB21	3.032	3.464	0.432	0.000	0.000
RAB22A	2.770	3.500	0.729	0.000	0.000
RAB23	1.203	1.944	0.741	0.000	0.000
RAB24	3.685	4.589	0.904	0.000	0.000
RAB25	2.331	0.905	-1.426	0.000	0.000
RAB26	4.193	3.503	-0.690	0.000	0.000
RAB27A	3.900	3.855	-0.046	0.348	0.364

RAB27B	0.841	1.068	0.227	0.000	0.000
RAB28	3.032	3.625	0.593	0.000	0.000
RAB2A	5.614	6.366	0.752	0.000	0.000
RAB2B	3.321	3.636	0.315	0.000	0.000
RAB30	1.358	1.415	0.057	0.082	0.089
RAB31	2.741	2.936	0.195	0.002	0.002
RAB32	5.266	5.536	0.270	0.000	0.000
RAB33A	0.768	0.813	0.045	0.215	0.228
RAB33B	3.197	2.969	-0.229	0.000	0.000
RAB34	2.799	3.356	0.556	0.000	0.000
RAB35	3.741	4.418	0.678	0.000	0.000
RAB36	0.681	0.563	-0.118	0.004	0.004
RAB37	3.002	2.926	-0.076	0.129	0.138
RAB38	1.167	1.211	0.044	0.416	0.431
RAB39B	0.409	0.420	0.011	0.646	0.659
RAB3A	1.483	2.318	0.835	0.000	0.000
RAB3B	0.514	1.620	1.106	0.000	0.000
RAB3C	0.272	0.673	0.401	0.000	0.000
RAB3D	1.292	1.952	0.660	0.000	0.000
RAB3GAP1	3.460	4.040	0.580	0.000	0.000
RAB3GAP2	2.838	3.674	0.837	0.000	0.000
RAB3IL1	3.128	2.867	-0.261	0.000	0.000
RAB3IP	2.275	2.871	0.596	0.000	0.000
RAB40A	0.171	0.268	0.097	0.000	0.000
RAB40AL	0.095	0.165	0.069	0.000	0.000
RAB40B	2.745	3.405	0.660	0.000	0.000
RAB40C	3.345	3.834	0.489	0.000	0.000
RAB42	0.874	1.340	0.466	0.000	0.000
RAB43	3.057	3.547	0.491	0.000	0.000
RAB4A	4.910	5.744	0.834	0.000	0.000
RAB4B	3.044	3.759	0.716	0.000	0.000
RAB4B-EGLN	1.914	2.337	0.424	0.000	0.000
RAB5A	4.588	5.048	0.460	0.000	0.000
RAB5B	4.429	5.151	0.722	0.000	0.000
RAB5C	5.520	6.371	0.851	0.000	0.000
RAB6A	5.239	5.563	0.324	0.000	0.000
RAB6B	0.785	1.442	0.657	0.000	0.000
RAB7A	7.017	7.531	0.514	0.000	0.000
RAB8B	3.113	3.497	0.384	0.000	0.000
RAB9A	4.151	4.564	0.413	0.000	0.000
RAB9B	0.247	0.344	0.097	0.000	0.000
RABAC1	5.866	6.279	0.413	0.000	0.000
RABEP1	4.138	4.132	-0.005	0.867	0.872
RABEP2	2.702	3.301	0.600	0.000	0.000
RABEPK	4.183	5.057	0.874	0.000	0.000
RABGAP1	2.717	3.372	0.655	0.000	0.000
RABGAP1L	1.989	2.409	0.421	0.000	0.000
RABGEF1	2.399	2.903	0.504	0.000	0.000
RABGGTA	3.677	4.289	0.612	0.000	0.000
RABGGTB	4.441	5.233	0.793	0.000	0.000
RABIF	2.887	4.104	1.217	0.000	0.000
RABL2A	1.405	1.705	0.300	0.000	0.000

RABL2B	2.104	2.579	0.475	0.000	0.000
RABL3	3.186	3.702	0.516	0.000	0.000
RAC1	6.840	7.651	0.811	0.000	0.000
RAC2	3.921	3.694	-0.227	0.002	0.003
RAC3	3.424	4.602	1.178	0.000	0.000
RACGAP1	1.431	3.512	2.081	0.000	0.000
RAD1	2.406	3.300	0.893	0.000	0.000
RAD17	3.369	3.967	0.598	0.000	0.000
RAD18	1.594	2.555	0.961	0.000	0.000
RAD21	4.866	6.126	1.260	0.000	0.000
RAD23A	6.135	6.704	0.569	0.000	0.000
RAD23B	6.396	6.816	0.421	0.000	0.000
RAD50	3.477	4.111	0.635	0.000	0.000
RAD51	0.634	1.996	1.362	0.000	0.000
RAD51AP1	1.036	2.758	1.723	0.000	0.000
RAD51AP2	0.275	0.357	0.082	0.000	0.000
RAD51B	0.716	1.024	0.308	0.000	0.000
RAD51C	2.034	2.989	0.956	0.000	0.000
RAD51D	1.118	1.653	0.535	0.000	0.000
RAD52	2.157	2.640	0.483	0.000	0.000
RAD54B	0.922	1.771	0.849	0.000	0.000
RAD54L	0.560	1.943	1.384	0.000	0.000
RAD9A	3.024	3.883	0.858	0.000	0.000
RAD9B	0.346	0.567	0.221	0.000	0.000
RADIL	0.175	0.236	0.062	0.001	0.001
RAE1	2.805	3.659	0.854	0.000	0.000
RAET1E	0.077	0.162	0.085	0.000	0.000
RAET1G	0.311	0.536	0.226	0.000	0.000
RAF1	4.929	5.563	0.633	0.000	0.000
RAG1	0.539	0.492	-0.048	0.094	0.101
RAI1	2.060	2.581	0.521	0.000	0.000
RAI14	3.492	4.310	0.818	0.000	0.000
RAI2	2.217	1.910	-0.308	0.000	0.000
RALA	3.838	4.767	0.929	0.000	0.000
RALB	4.469	4.967	0.498	0.000	0.000
RALBP1	4.313	5.183	0.870	0.000	0.000
RALGAPA1	2.091	2.413	0.322	0.000	0.000
RALGAPA2	3.718	3.995	0.277	0.000	0.000
RALGAPB	2.632	3.424	0.792	0.000	0.000
RALGDS	3.371	3.633	0.262	0.000	0.000
RALGPS1	1.025	1.520	0.495	0.000	0.000
RALGPS2	3.675	4.131	0.455	0.000	0.000
RALY	4.493	5.550	1.057	0.000	0.000
RAMP1	5.771	6.545	0.774	0.000	0.000
RAMP2	3.861	4.532	0.671	0.000	0.000
RAMP3	4.943	4.013	-0.929	0.000	0.000
RAN	5.435	6.597	1.163	0.000	0.000
RANBP1	4.650	5.358	0.707	0.000	0.000
RANBP10	3.953	3.977	0.024	0.598	0.612
RANBP17	0.645	1.007	0.361	0.000	0.000
RANBP2	3.575	3.888	0.313	0.000	0.000
RANBP3	3.599	4.236	0.637	0.000	0.000

RANBP3L	2.392	1.006	-1.386	0.000	0.000
RANBP6	3.435	3.576	0.141	0.000	0.000
RANBP9	4.430	5.086	0.656	0.000	0.000
RANGAP1	5.214	6.004	0.790	0.000	0.000
RAP1A	4.788	5.130	0.342	0.000	0.000
RAP1GAP	3.548	4.000	0.452	0.000	0.000
RAP1GAP2	1.197	1.390	0.193	0.001	0.001
RAP1GDS1	3.399	3.743	0.344	0.000	0.000
RAP2A	2.780	4.178	1.398	0.000	0.000
RAP2B	3.064	3.694	0.631	0.000	0.000
RAP2C	5.016	5.262	0.246	0.000	0.000
RAPGEF1	3.788	4.448	0.660	0.000	0.000
RAPGEF2	3.187	3.359	0.172	0.000	0.000
RAPGEF3	1.234	1.651	0.417	0.000	0.000
RAPGEF4	3.259	3.234	-0.025	0.596	0.610
RAPGEF5	2.950	3.047	0.098	0.060	0.065
RAPGEF6	1.554	1.934	0.380	0.000	0.000
RAPGEFL1	1.746	2.391	0.645	0.000	0.000
RAPSN	0.380	0.658	0.278	0.000	0.000
RARA	4.130	4.857	0.727	0.000	0.000
RARB	1.917	1.567	-0.349	0.000	0.000
RARG	1.728	2.006	0.279	0.000	0.000
RARRES1	2.148	2.896	0.748	0.000	0.000
RARRES2	10.054	10.039	-0.015	0.806	0.814
RARRES3	6.458	6.194	-0.264	0.000	0.000
RARS	4.854	5.625	0.772	0.000	0.000
RARS2	4.288	4.856	0.568	0.000	0.000
RASA1	2.983	3.551	0.568	0.000	0.000
RASA2	1.955	2.186	0.231	0.000	0.000
RASA3	2.524	2.430	-0.094	0.109	0.117
RASA4	0.998	1.317	0.319	0.000	0.000
RASAL1	0.480	0.679	0.199	0.000	0.000
RASAL2	1.397	2.200	0.803	0.000	0.000
RASAL3	2.483	2.391	-0.092	0.101	0.109
RASD1	5.560	4.812	-0.748	0.000	0.000
RASD2	0.367	0.967	0.600	0.000	0.000
RASEF	1.048	1.629	0.581	0.000	0.000
RASGEF1A	0.452	0.723	0.270	0.000	0.000
RASGEF1B	3.721	2.619	-1.102	0.000	0.000
RASGRF1	0.145	0.465	0.321	0.000	0.000
RASGRF2	0.616	1.215	0.599	0.000	0.000
RASGRP1	1.082	1.049	-0.034	0.463	0.479
RASGRP2	2.481	2.092	-0.390	0.000	0.000
RASGRP3	1.708	2.334	0.626	0.000	0.000
RASGRP4	1.075	0.865	-0.210	0.000	0.000
RASIP1	2.595	2.829	0.233	0.000	0.000
RASL10A	0.693	0.674	-0.019	0.462	0.478
RASL11A	3.457	2.975	-0.482	0.000	0.000
RASL11B	0.769	0.580	-0.189	0.000	0.000
RASL12	0.857	1.925	1.068	0.000	0.000
RASSF1	3.513	4.192	0.679	0.000	0.000
RASSF2	2.327	2.314	-0.012	0.815	0.823

RASSF3	3.212	4.198	0.986	0.000	0.000
RASSF4	3.665	4.813	1.148	0.000	0.000
RASSF6	0.674	0.877	0.203	0.000	0.000
RASSF7	5.122	5.628	0.506	0.000	0.000
RASSF8	2.006	2.045	0.038	0.457	0.473
RASSF9	0.891	0.780	-0.111	0.029	0.032
RAVER1	2.277	2.816	0.539	0.000	0.000
RAVER2	1.214	1.917	0.703	0.000	0.000
RB1	3.153	3.529	0.375	0.000	0.000
RB1CC1	3.934	4.792	0.859	0.000	0.000
RBAK	2.467	2.807	0.340	0.000	0.000
RBBP5	3.002	3.935	0.932	0.000	0.000
RBBP6	3.821	3.951	0.129	0.000	0.001
RBBP7	4.376	5.379	1.003	0.000	0.000
RBBP8	2.869	3.595	0.726	0.000	0.000
RBBP9	3.978	4.347	0.369	0.000	0.000
RBCK1	5.429	6.404	0.975	0.000	0.000
RBFA	3.625	4.028	0.403	0.000	0.000
RBFOX2	3.500	4.230	0.730	0.000	0.000
RBKS	4.037	3.889	-0.148	0.000	0.000
RBL2	4.848	4.555	-0.293	0.000	0.000
RBM10	4.524	5.223	0.699	0.000	0.000
RBM11	0.227	0.241	0.014	0.604	0.618
RBM12	3.371	4.075	0.704	0.000	0.000
RBM12B	1.615	2.531	0.916	0.000	0.000
RBM14	4.113	4.691	0.578	0.000	0.000
RBM14-RBM4	2.170	2.553	0.384	0.000	0.000
RBM15	2.337	2.674	0.337	0.000	0.000
RBM17	4.317	5.034	0.717	0.000	0.000
RBM18	2.921	3.437	0.516	0.000	0.000
RBM19	3.067	3.896	0.829	0.000	0.000
RBM20	0.239	0.386	0.147	0.000	0.000
RBM22	4.253	4.939	0.686	0.000	0.000
RBM23	3.353	3.690	0.338	0.000	0.000
RBM24	0.748	2.251	1.503	0.000	0.000
RBM25	4.247	4.496	0.249	0.000	0.000
RBM26	2.912	3.357	0.445	0.000	0.000
RBM27	2.914	3.415	0.501	0.000	0.000
RBM28	2.105	2.790	0.685	0.000	0.000
RBM3	5.578	6.753	1.175	0.000	0.000
RBM33	3.172	3.773	0.601	0.000	0.000
RBM34	2.637	3.656	1.019	0.000	0.000
RBM38	3.768	4.092	0.324	0.000	0.000
RBM39	5.626	6.237	0.611	0.000	0.000
RBM4	3.182	3.878	0.696	0.000	0.000
RBM41	2.297	2.827	0.530	0.000	0.000
RBM42	5.427	6.436	1.009	0.000	0.000
RBM43	2.210	2.375	0.165	0.000	0.000
RBM44	0.285	0.382	0.097	0.000	0.000
RBM45	1.998	2.740	0.741	0.000	0.000
RBM47	4.276	4.588	0.312	0.000	0.000
RBM4B	2.615	3.302	0.687	0.000	0.000

RBM5	4.240	4.683	0.443	0.000	0.000
RBM6	4.672	5.045	0.373	0.000	0.000
RBM7	3.075	3.069	-0.006	0.861	0.867
RBMS1	3.193	3.208	0.015	0.760	0.770
RBMS2	1.814	2.039	0.225	0.000	0.000
RBMS3	1.738	1.098	-0.640	0.000	0.000
RBMX	4.627	5.480	0.853	0.000	0.000
RBMX2	3.448	4.291	0.844	0.000	0.000
RBMXL1	3.119	3.151	0.032	0.388	0.404
RBP1	5.461	4.107	-1.354	0.000	0.000
RBP2	0.224	0.782	0.557	0.000	0.000
RBP4	13.481	12.400	-1.081	0.000	0.000
RBP5	7.796	6.707	-1.089	0.000	0.000
RBP7	3.180	4.709	1.529	0.000	0.000
RBPJ	3.008	3.451	0.443	0.000	0.000
RBPMS	4.029	4.092	0.063	0.170	0.181
RBPMS2	3.213	3.727	0.514	0.000	0.000
RBX1	4.278	5.013	0.735	0.000	0.000
RC3H1	2.577	3.049	0.472	0.000	0.000
RC3H2	2.302	2.781	0.480	0.000	0.000
RCAN1	5.810	4.332	-1.478	0.000	0.000
RCAN2	2.182	2.250	0.068	0.296	0.311
RCAN3	1.041	1.578	0.537	0.000	0.000
RCBTB1	2.848	3.635	0.787	0.000	0.000
RCBTB2	3.087	2.782	-0.305	0.000	0.000
RCC1	3.314	4.341	1.027	0.000	0.000
RCC2	3.961	4.944	0.982	0.000	0.000
RCCD1	2.421	3.433	1.012	0.000	0.000
RCE1	2.731	3.628	0.898	0.000	0.000
RCHY1	2.871	2.959	0.087	0.006	0.007
RCL1	5.752	4.685	-1.068	0.000	0.000
RCN1	4.891	5.213	0.321	0.000	0.000
RCN2	2.433	3.637	1.203	0.000	0.000
RCN3	4.535	4.435	-0.100	0.079	0.086
RCOR1	3.036	3.541	0.505	0.000	0.000
RCOR2	0.246	0.640	0.394	0.000	0.000
RCOR3	2.911	3.762	0.850	0.000	0.000
RCSD1	2.310	2.151	-0.158	0.002	0.003
RDH10	4.664	5.300	0.635	0.000	0.000
RDH11	5.463	5.930	0.466	0.000	0.000
RDH12	1.950	1.955	0.005	0.934	0.937
RDH13	1.835	2.413	0.578	0.000	0.000
RDH14	4.346	4.874	0.529	0.000	0.000
RDH16	8.172	5.752	-2.420	0.000	0.000
RDH5	4.984	3.394	-1.590	0.000	0.000
RDX	5.305	5.459	0.155	0.001	0.001
REC8	2.590	2.412	-0.177	0.003	0.003
RECK	1.804	1.950	0.146	0.003	0.004
RECQL	3.085	3.930	0.845	0.000	0.000
RECQL4	1.816	3.838	2.022	0.000	0.000
RECQL5	3.012	3.847	0.835	0.000	0.000
REEP1	0.819	0.915	0.096	0.043	0.047

REEP2	0.222	0.724	0.502	0.000	0.000
REEP3	3.980	4.242	0.262	0.000	0.000
REEP4	3.118	4.031	0.913	0.000	0.000
REEP5	6.284	6.495	0.211	0.000	0.000
REEP6	8.214	7.426	-0.789	0.000	0.000
REG1A	0.361	1.718	1.357	0.000	0.000
REG3A	0.726	2.718	1.992	0.000	0.000
REG4	0.125	0.329	0.204	0.000	0.000
REL	1.103	1.036	-0.067	0.032	0.036
RELA	4.749	5.238	0.489	0.000	0.000
RELB	3.234	4.293	1.059	0.000	0.000
RELL1	2.034	2.345	0.311	0.000	0.000
RELL2	1.332	2.066	0.734	0.000	0.000
RELN	3.624	2.775	-0.848	0.000	0.000
RELT	1.313	1.638	0.325	0.000	0.000
REM1	0.795	0.905	0.110	0.003	0.004
REM2	0.347	0.361	0.014	0.470	0.485
REN	0.677	1.369	0.692	0.000	0.000
RENBP	3.107	3.418	0.311	0.000	0.000
REPIN1	5.239	6.421	1.182	0.000	0.000
REPS1	3.453	3.258	-0.195	0.000	0.000
REPS2	2.423	2.285	-0.138	0.000	0.000
RER1	5.831	6.280	0.449	0.000	0.000
RERE	3.564	3.898	0.335	0.000	0.000
RERG	1.485	1.511	0.026	0.625	0.638
RERGL	1.290	1.249	-0.041	0.470	0.485
REST	2.995	3.252	0.257	0.000	0.000
RET	0.775	0.390	-0.385	0.000	0.000
RETN	0.493	0.512	0.019	0.680	0.692
RETSAT	6.441	6.321	-0.120	0.008	0.009
REV1	3.169	3.435	0.267	0.000	0.000
REV3L	2.322	2.496	0.174	0.000	0.000
REXO1	3.636	3.874	0.238	0.000	0.000
REXO2	4.156	4.756	0.600	0.000	0.000
REXO4	3.761	4.721	0.960	0.000	0.000
RFC1	3.918	4.914	0.996	0.000	0.000
RFC2	4.052	4.939	0.887	0.000	0.000
RFC3	2.053	3.345	1.292	0.000	0.000
RFC4	2.403	4.146	1.743	0.000	0.000
RFC5	2.850	3.792	0.941	0.000	0.000
RFESD	0.708	1.075	0.368	0.000	0.000
RFFL	3.671	4.219	0.548	0.000	0.000
RFK	3.997	4.593	0.596	0.000	0.000
RFNG	5.205	5.624	0.419	0.000	0.000
RFPL2	0.255	0.253	-0.002	0.914	0.917
RFPL4A	1.120	0.780	-0.340	0.000	0.000
RFT1	2.690	3.581	0.891	0.000	0.000
RFTN1	4.028	4.190	0.162	0.010	0.011
RFTN2	0.781	1.173	0.393	0.000	0.000
RFWD3	2.188	2.982	0.794	0.000	0.000
RFX1	2.635	2.851	0.215	0.000	0.000
RFX2	1.248	1.205	-0.043	0.307	0.322

RFX3	0.634	0.885	0.251	0.000	0.000
RFX5	3.177	4.709	1.532	0.000	0.000
RFX7	1.666	2.165	0.499	0.000	0.000
RFX8	0.217	0.830	0.613	0.000	0.000
RFXANK	4.021	5.270	1.249	0.000	0.000
RFXAP	1.554	1.958	0.404	0.000	0.000
RGL1	3.286	3.080	-0.206	0.000	0.000
RGL2	4.137	4.819	0.682	0.000	0.000
RGL3	3.568	3.936	0.369	0.000	0.000
RGL4	1.206	1.350	0.144	0.000	0.000
RGMA	0.126	0.327	0.201	0.000	0.000
RGMB	2.086	2.271	0.186	0.000	0.000
RGN	7.894	6.912	-0.981	0.000	0.000
RGP1	2.104	2.769	0.665	0.000	0.000
RGPD2	0.458	0.422	-0.036	0.082	0.089
RGS1	4.151	3.784	-0.366	0.000	0.000
RGS10	3.256	3.888	0.632	0.000	0.000
RGS11	1.084	0.690	-0.394	0.000	0.000
RGS12	2.518	3.345	0.828	0.000	0.000
RGS14	4.653	5.420	0.767	0.000	0.000
RGS16	3.039	3.123	0.084	0.316	0.331
RGS18	1.517	1.189	-0.328	0.000	0.000
RGS19	2.853	3.474	0.621	0.000	0.000
RGS2	4.777	4.291	-0.486	0.000	0.000
RGS3	3.379	3.476	0.097	0.040	0.044
RGS4	1.462	1.192	-0.271	0.000	0.000
RGS5	3.536	5.157	1.621	0.000	0.000
RGS7BP	0.579	0.531	-0.047	0.210	0.223
RGS9	0.991	1.132	0.141	0.005	0.006
RHBDD1	2.951	3.447	0.495	0.000	0.000
RHBDD2	5.877	6.364	0.487	0.000	0.000
RHBDD3	3.404	4.543	1.139	0.000	0.000
RHBDF1	2.503	3.106	0.604	0.000	0.000
RHBDF2	2.422	3.407	0.985	0.000	0.000
RHBDL1	1.181	2.092	0.911	0.000	0.000
RHBDL2	0.346	0.643	0.297	0.000	0.000
RHBDL3	0.141	0.596	0.455	0.000	0.000
RHBG	2.600	3.212	0.612	0.000	0.000
RHCE	0.904	1.089	0.185	0.000	0.000
RHCG	0.484	0.933	0.449	0.000	0.000
RHEB	4.964	6.022	1.059	0.000	0.000
RHEBL1	0.870	1.492	0.623	0.000	0.000
RHOA	7.396	8.280	0.883	0.000	0.000
RHOB	9.403	8.543	-0.860	0.000	0.000
RHOBTB1	2.796	3.172	0.376	0.000	0.000
RHOBTB2	1.505	2.267	0.763	0.000	0.000
RHOBTB3	3.836	4.410	0.575	0.000	0.000
RHOC	5.753	6.827	1.074	0.000	0.000
RHOD	5.858	6.084	0.226	0.000	0.000
RHOG	4.869	5.529	0.659	0.000	0.000
RHOH	1.356	1.135	-0.221	0.000	0.000
RHOJ	1.893	2.170	0.278	0.000	0.000

RHOQ	3.531	4.292	0.761	0.000	0.000
RHOT1	3.086	3.711	0.625	0.000	0.000
RHOT2	4.849	5.449	0.600	0.000	0.000
RHOU	5.506	5.943	0.437	0.000	0.000
RHOV	0.504	0.716	0.211	0.001	0.002
RHOXF1	0.500	0.602	0.102	0.005	0.006
RHPN1	1.528	2.357	0.829	0.000	0.000
RHPN2	3.675	4.248	0.573	0.000	0.000
RIBC1	0.495	0.746	0.251	0.000	0.000
RIBC2	0.340	1.217	0.876	0.000	0.000
RIC3	1.361	0.522	-0.839	0.000	0.000
RIC8A	4.107	5.072	0.965	0.000	0.000
RIC8B	2.403	2.835	0.431	0.000	0.000
RICTOR	2.600	2.912	0.313	0.000	0.000
RIF1	2.089	2.642	0.553	0.000	0.000
RILP	4.879	4.664	-0.215	0.000	0.000
RILPL2	2.628	2.649	0.021	0.553	0.567
RIMKLA	0.154	0.310	0.156	0.000	0.000
RIMKLB	1.405	1.425	0.021	0.641	0.654
RIMS3	0.526	0.899	0.373	0.000	0.000
RIN1	1.055	1.352	0.297	0.000	0.000
RIN2	2.664	2.800	0.136	0.004	0.005
RIN3	2.861	3.109	0.248	0.000	0.000
RINL	2.420	3.084	0.664	0.000	0.000
RINT1	3.233	4.015	0.781	0.000	0.000
RIOK1	3.102	3.911	0.809	0.000	0.000
RIOK2	2.852	3.426	0.573	0.000	0.000
RIOK3	5.183	5.406	0.224	0.000	0.000
RIPK1	4.367	4.768	0.400	0.000	0.000
RIPK2	2.948	4.037	1.089	0.000	0.000
RIPK3	1.496	1.386	-0.109	0.021	0.023
RIPK4	4.127	3.785	-0.342	0.000	0.000
RIPPLY1	2.526	2.158	-0.369	0.000	0.000
RIT1	2.788	3.765	0.977	0.000	0.000
RLF	2.713	2.899	0.185	0.000	0.000
RLIM	2.969	3.373	0.404	0.000	0.000
RLN2	0.136	0.157	0.021	0.143	0.153
RMI1	2.128	3.120	0.991	0.000	0.000
RMI2	1.266	2.747	1.481	0.000	0.000
RMND1	4.444	4.769	0.325	0.000	0.000
RMND5A	4.605	5.058	0.453	0.000	0.000
RMND5B	3.080	3.871	0.791	0.000	0.000
RNASE1	5.338	6.152	0.815	0.000	0.000
RNASE13	0.447	0.459	0.012	0.655	0.668
RNASE2	0.648	0.994	0.345	0.000	0.000
RNASE4	6.215	5.471	-0.744	0.000	0.000
RNASE6	3.650	3.741	0.091	0.181	0.192
RNASE7	0.677	0.712	0.035	0.445	0.461
RNASEH1	2.664	3.588	0.923	0.000	0.000
RNASEH2A	2.666	4.593	1.927	0.000	0.000
RNASEH2B	2.265	2.942	0.677	0.000	0.000
RNASEH2C	4.362	4.915	0.552	0.000	0.000

RNASEK	4.933	5.381	0.447	0.000	0.000
RNASEL	1.664	1.863	0.199	0.000	0.000
RNASET2	3.853	4.217	0.364	0.000	0.000
RND1	5.498	5.039	-0.460	0.000	0.000
RND2	2.137	1.861	-0.276	0.002	0.003
RND3	6.245	4.593	-1.652	0.000	0.000
RNF10	5.458	6.041	0.583	0.000	0.000
RNF103	4.392	4.868	0.477	0.000	0.000
RNF11	5.491	5.685	0.194	0.000	0.000
RNF111	2.832	3.230	0.398	0.000	0.000
RNF112	0.823	0.723	-0.099	0.002	0.002
RNF113A	3.996	4.792	0.796	0.000	0.000
RNF114	4.984	5.454	0.470	0.000	0.000
RNF121	3.339	4.096	0.757	0.000	0.000
RNF122	2.973	2.703	-0.270	0.000	0.000
RNF123	4.228	4.368	0.140	0.000	0.001
RNF125	3.290	2.282	-1.008	0.000	0.000
RNF126	4.551	5.087	0.536	0.000	0.000
RNF128	6.183	6.191	0.008	0.860	0.865
RNF13	5.182	5.469	0.287	0.000	0.000
RNF130	5.178	5.092	-0.086	0.004	0.004
RNF133	0.160	0.208	0.047	0.000	0.001
RNF135	2.758	2.434	-0.323	0.000	0.000
RNF138	3.500	3.709	0.209	0.000	0.000
RNF139	4.546	5.148	0.602	0.000	0.000
RNF14	4.257	4.940	0.683	0.000	0.000
RNF141	3.177	3.580	0.403	0.000	0.000
RNF144A	1.050	1.838	0.789	0.000	0.000
RNF144B	3.391	3.216	-0.175	0.000	0.000
RNF145	3.447	4.040	0.594	0.000	0.000
RNF146	4.132	4.375	0.243	0.000	0.000
RNF148	0.225	0.344	0.119	0.000	0.000
RNF149	4.547	4.931	0.384	0.000	0.000
RNF150	0.439	0.309	-0.130	0.000	0.000
RNF152	3.022	2.279	-0.743	0.000	0.000
RNF157	1.140	2.479	1.339	0.000	0.000
RNF165	1.249	0.469	-0.780	0.000	0.000
RNF166	3.289	3.476	0.188	0.000	0.000
RNF167	6.182	6.438	0.256	0.000	0.000
RNF168	3.012	3.283	0.271	0.000	0.000
RNF169	2.031	2.420	0.389	0.000	0.000
RNF170	3.205	3.564	0.359	0.000	0.000
RNF175	0.374	0.303	-0.071	0.001	0.001
RNF180	1.611	1.213	-0.398	0.000	0.000
RNF181	6.748	7.526	0.777	0.000	0.000
RNF183	0.094	0.265	0.171	0.000	0.000
RNF185	5.012	5.283	0.271	0.000	0.000
RNF186	0.408	0.483	0.076	0.037	0.041
RNF187	5.785	7.099	1.314	0.000	0.000
RNF19A	4.619	5.075	0.456	0.000	0.000
RNF19B	3.396	3.939	0.543	0.000	0.000
RNF2	2.272	3.054	0.782	0.000	0.000

RNF20	3.524	4.487	0.963	0.000	0.000
RNF207	2.287	2.978	0.691	0.000	0.000
RNF208	3.631	3.985	0.354	0.000	0.000
RNF212	0.392	0.221	-0.172	0.000	0.000
RNF213	3.854	4.928	1.074	0.000	0.000
RNF214	2.742	3.275	0.534	0.000	0.000
RNF215	1.728	2.537	0.809	0.000	0.000
RNF216	2.938	3.876	0.938	0.000	0.000
RNF217	1.914	1.997	0.083	0.052	0.057
RNF219	2.011	2.686	0.675	0.000	0.000
RNF220	3.341	4.288	0.947	0.000	0.000
RNF224	0.079	0.249	0.170	0.000	0.000
RNF24	1.056	1.683	0.627	0.000	0.000
RNF25	3.773	4.476	0.703	0.000	0.000
RNF26	3.964	5.026	1.062	0.000	0.000
RNF31	3.405	4.044	0.639	0.000	0.000
RNF32	0.953	1.368	0.415	0.000	0.000
RNF34	2.960	3.823	0.863	0.000	0.000
RNF38	2.899	3.519	0.620	0.000	0.000
RNF39	0.761	1.068	0.307	0.000	0.000
RNF4	3.393	4.161	0.768	0.000	0.000
RNF40	4.089	4.735	0.646	0.000	0.000
RNF41	2.977	3.521	0.545	0.000	0.000
RNF43	2.142	3.332	1.190	0.000	0.000
RNF44	3.271	4.170	0.899	0.000	0.000
RNF5	6.785	7.288	0.503	0.000	0.000
RNF6	3.225	3.780	0.554	0.000	0.000
RNF7	4.974	5.589	0.615	0.000	0.000
RNF8	2.140	3.149	1.009	0.000	0.000
RNFT1	3.911	4.043	0.132	0.001	0.001
RNFT2	0.202	0.895	0.693	0.000	0.000
RNGTT	2.659	3.126	0.467	0.000	0.000
RNH1	5.168	5.707	0.538	0.000	0.000
RNLS	2.441	2.716	0.275	0.000	0.000
RNMT	3.155	3.885	0.730	0.000	0.000
RNPC3	2.275	2.690	0.415	0.000	0.000
RNPEP	5.008	5.872	0.865	0.000	0.000
RNPEPL1	4.303	5.220	0.917	0.000	0.000
RNPS1	3.832	4.642	0.809	0.000	0.000
ROBO1	1.563	3.943	2.380	0.000	0.000
ROBO2	0.158	0.377	0.219	0.000	0.000
ROBO3	1.428	1.776	0.349	0.000	0.000
ROBO4	2.514	2.721	0.207	0.000	0.000
ROCK1	3.286	3.633	0.347	0.000	0.000
ROCK2	3.045	3.689	0.644	0.000	0.000
ROGDI	3.995	4.270	0.274	0.000	0.000
ROM1	1.731	1.773	0.042	0.245	0.259
ROMO1	7.145	7.982	0.838	0.000	0.000
ROPN1B	0.549	0.510	-0.039	0.170	0.181
ROPN1L	0.315	0.258	-0.057	0.001	0.002
ROR1	0.383	0.632	0.249	0.000	0.000
ROR2	0.669	0.826	0.157	0.001	0.001

RORA	3.507	3.094	-0.413	0.000	0.000
RORC	5.532	5.587	0.055	0.465	0.480
RP2	3.095	3.322	0.227	0.000	0.000
RP9	2.715	3.534	0.818	0.000	0.000
RPA1	3.780	4.516	0.736	0.000	0.000
RPA2	4.331	5.044	0.713	0.000	0.000
RPA3	3.890	4.638	0.748	0.000	0.000
RPA4	0.300	0.370	0.070	0.000	0.000
RPAIN	3.381	3.446	0.065	0.034	0.038
RPAP1	2.591	3.566	0.975	0.000	0.000
RPAP2	1.327	1.823	0.496	0.000	0.000
RPAP3	2.945	3.623	0.678	0.000	0.000
RPE	3.640	4.393	0.753	0.000	0.000
RPF1	4.743	5.262	0.519	0.000	0.000
RPF2	3.818	4.480	0.662	0.000	0.000
RPGR	1.738	2.072	0.334	0.000	0.000
RPGRIP1	0.723	0.688	-0.035	0.172	0.183
RPGRIP1L	0.452	0.876	0.423	0.000	0.000
RPH3AL	2.898	2.604	-0.295	0.000	0.000
RPIA	3.069	4.071	1.001	0.000	0.000
RPL10A	8.651	9.409	0.758	0.000	0.000
RPL11	9.394	9.714	0.320	0.000	0.000
RPL12	8.176	8.712	0.537	0.000	0.000
RPL13	8.334	8.714	0.380	0.000	0.000
RPL13A	9.386	10.138	0.751	0.000	0.000
RPL14	6.631	7.380	0.749	0.000	0.000
RPL15	7.669	8.355	0.686	0.000	0.000
RPL17	6.573	7.413	0.840	0.000	0.000
RPL18	8.016	8.747	0.731	0.000	0.000
RPL18A	7.135	7.840	0.704	0.000	0.000
RPL19	9.153	9.909	0.756	0.000	0.000
RPL21	7.557	7.919	0.362	0.000	0.000
RPL22	6.901	7.282	0.382	0.000	0.000
RPL22L1	4.517	5.630	1.113	0.000	0.000
RPL23	7.577	8.397	0.819	0.000	0.000
RPL23A	7.488	8.336	0.848	0.000	0.000
RPL24	8.144	8.658	0.514	0.000	0.000
RPL26	8.136	8.327	0.191	0.000	0.000
RPL27	8.698	9.591	0.893	0.000	0.000
RPL27A	7.320	8.020	0.700	0.000	0.000
RPL28	7.189	8.040	0.852	0.000	0.000
RPL29	8.778	9.457	0.679	0.000	0.000
RPL3	9.357	9.758	0.401	0.000	0.000
RPL30	8.393	9.462	1.069	0.000	0.000
RPL31	7.759	8.408	0.649	0.000	0.000
RPL32	8.238	8.887	0.649	0.000	0.000
RPL34	8.050	8.272	0.221	0.000	0.000
RPL35	8.880	9.566	0.687	0.000	0.000
RPL35A	7.745	8.495	0.750	0.000	0.000
RPL36	7.880	8.456	0.576	0.000	0.000
RPL36A	5.689	6.777	1.088	0.000	0.000
RPL36A-HNRI	3.198	3.680	0.482	0.000	0.000

RPL36AL	8.515	8.692	0.177	0.000	0.000
RPL37	7.348	8.165	0.817	0.000	0.000
RPL37A	7.879	8.573	0.695	0.000	0.000
RPL38	7.439	8.363	0.925	0.000	0.000
RPL39	7.603	8.401	0.798	0.000	0.000
RPL39L	1.973	3.277	1.304	0.000	0.000
RPL4	8.309	8.980	0.671	0.000	0.000
RPL5	8.370	9.035	0.665	0.000	0.000
RPL7	7.922	8.931	1.009	0.000	0.000
RPL7A	8.723	9.495	0.772	0.000	0.000
RPL7L1	3.930	4.616	0.685	0.000	0.000
RPL8	9.427	10.599	1.172	0.000	0.000
RPL9	7.117	7.705	0.588	0.000	0.000
RPLP1	8.539	9.537	0.999	0.000	0.000
RPLP2	8.389	9.250	0.861	0.000	0.000
RPN1	7.177	7.843	0.666	0.000	0.000
RPN2	7.105	8.266	1.162	0.000	0.000
RPP14	4.222	4.513	0.291	0.000	0.000
RPP21	3.518	4.539	1.021	0.000	0.000
RPP25	2.322	2.521	0.199	0.002	0.002
RPP30	3.040	3.761	0.721	0.000	0.000
RPP38	3.554	4.280	0.726	0.000	0.000
RPP40	2.583	3.642	1.059	0.000	0.000
RPRD1A	3.181	4.054	0.873	0.000	0.000
RPRD1B	4.226	4.595	0.370	0.000	0.000
RPRD2	2.923	3.886	0.963	0.000	0.000
RPRML	0.156	0.388	0.232	0.000	0.000
RPS10	7.240	8.272	1.032	0.000	0.000
RPS10-NUDT3	3.416	4.020	0.603	0.000	0.000
RPS11	9.821	10.561	0.740	0.000	0.000
RPS12	9.600	10.394	0.794	0.000	0.000
RPS13	8.050	8.586	0.535	0.000	0.000
RPS14	8.119	8.834	0.715	0.000	0.000
RPS15	7.689	8.294	0.605	0.000	0.000
RPS15A	6.963	7.389	0.426	0.000	0.000
RPS16	8.745	9.530	0.785	0.000	0.000
RPS17	5.034	5.333	0.299	0.000	0.000
RPS18	9.137	10.090	0.953	0.000	0.000
RPS19	8.318	9.176	0.857	0.000	0.000
RPS19BP1	5.027	5.783	0.756	0.000	0.000
RPS20	8.879	9.734	0.855	0.000	0.000
RPS21	8.630	9.615	0.985	0.000	0.000
RPS23	7.979	8.705	0.726	0.000	0.000
RPS24	8.325	8.980	0.655	0.000	0.000
RPS25	8.820	9.315	0.494	0.000	0.000
RPS26	7.016	7.523	0.507	0.000	0.000
RPS27	9.251	10.172	0.921	0.000	0.000
RPS27A	7.749	8.517	0.768	0.000	0.000
RPS27L	5.222	5.257	0.034	0.375	0.391
RPS28	7.548	8.108	0.560	0.000	0.000
RPS29	6.445	6.870	0.425	0.000	0.000
RPS3	7.686	8.451	0.765	0.000	0.000

RPS3A	8.092	8.619	0.527	0.000	0.000
RPS4X	8.419	9.176	0.757	0.000	0.000
RPS4Y1	5.146	5.429	0.284	0.139	0.149
RPS5	7.572	8.685	1.113	0.000	0.000
RPS6	9.343	9.905	0.562	0.000	0.000
RPS6KA1	3.276	3.521	0.245	0.000	0.000
RPS6KA2	2.210	2.297	0.087	0.063	0.068
RPS6KA3	4.273	5.095	0.823	0.000	0.000
RPS6KA4	3.301	4.121	0.820	0.000	0.000
RPS6KA5	0.465	0.507	0.042	0.024	0.026
RPS6KA6	0.407	0.230	-0.177	0.000	0.000
RPS6KB1	3.058	3.617	0.559	0.000	0.000
RPS6KB2	4.087	4.829	0.742	0.000	0.000
RPS6KC1	2.404	3.442	1.038	0.000	0.000
RPS6KL1	1.008	1.923	0.915	0.000	0.000
RPS7	7.129	8.008	0.879	0.000	0.000
RPS8	8.575	9.287	0.712	0.000	0.000
RPS9	8.358	8.807	0.449	0.000	0.000
RPSA	6.641	7.751	1.110	0.000	0.000
RPTOR	2.325	3.359	1.034	0.000	0.000
RPUSD1	3.035	3.988	0.953	0.000	0.000
RPUSD2	2.800	3.516	0.717	0.000	0.000
RPUSD3	3.316	4.084	0.768	0.000	0.000
RPUSD4	3.561	3.972	0.411	0.000	0.000
RRAD	2.216	1.932	-0.284	0.000	0.000
RRAGA	6.120	6.500	0.380	0.000	0.000
RRAGB	2.848	3.394	0.545	0.000	0.000
RRAGC	2.568	3.378	0.810	0.000	0.000
RRAGD	2.572	4.080	1.508	0.000	0.000
RRAS	5.002	5.648	0.646	0.000	0.000
RRAS2	4.201	4.578	0.377	0.000	0.000
RRBP1	7.286	7.484	0.198	0.000	0.000
RREB1	3.130	3.640	0.510	0.000	0.000
RRH	0.280	0.282	0.002	0.877	0.882
RRM1	3.517	4.724	1.207	0.000	0.000
RRM2	1.394	3.955	2.562	0.000	0.000
RRM2B	3.539	4.179	0.640	0.000	0.000
RRN3	4.472	4.574	0.101	0.004	0.004
RRNAD1	4.270	5.024	0.754	0.000	0.000
RRP1	3.444	4.297	0.853	0.000	0.000
RRP12	2.605	3.493	0.888	0.000	0.000
RRP15	2.320	3.270	0.950	0.000	0.000
RRP1B	3.140	3.942	0.803	0.000	0.000
RRP36	4.397	5.422	1.025	0.000	0.000
RRP7A	3.887	4.763	0.876	0.000	0.000
RRP8	3.049	3.400	0.351	0.000	0.000
RRP9	3.838	4.961	1.123	0.000	0.000
RRS1	3.683	5.161	1.478	0.000	0.000
RSAD1	4.689	5.288	0.600	0.000	0.000
RSAD2	2.087	1.992	-0.095	0.125	0.134
RSBN1	2.347	2.504	0.157	0.000	0.000
RSBN1L	2.787	3.177	0.390	0.000	0.000

RSF1	2.651	2.881	0.230	0.000	0.000
RSL1D1	5.188	5.725	0.537	0.000	0.000
RSL24D1	5.203	5.754	0.551	0.000	0.000
RSPH1	0.565	0.701	0.136	0.001	0.001
RSPH4A	0.552	0.521	-0.031	0.135	0.145
RSPO2	0.335	0.421	0.087	0.053	0.058
RSPO3	2.369	0.675	-1.695	0.000	0.000
RSPO4	0.316	0.295	-0.021	0.409	0.425
RSPRY1	3.020	3.317	0.297	0.000	0.000
RSRC1	2.548	3.359	0.811	0.000	0.000
RSRC2	3.940	4.417	0.477	0.000	0.000
RSU1	4.173	4.974	0.801	0.000	0.000
RTEL1	1.536	2.118	0.582	0.000	0.000
RTEL1-TNFRS	1.284	1.769	0.484	0.000	0.000
RTF1	4.081	4.632	0.551	0.000	0.000
RTKN	5.114	5.491	0.377	0.000	0.000
RTKN2	0.176	0.804	0.628	0.000	0.000
RTN1	0.788	1.192	0.403	0.000	0.000
RTN2	1.730	2.592	0.862	0.000	0.000
RTN3	5.103	6.042	0.939	0.000	0.000
RTN4	6.907	6.906	-0.001	0.984	0.985
RTN4IP1	2.912	3.490	0.577	0.000	0.000
RTN4R	2.272	2.630	0.358	0.000	0.000
RTN4RL1	1.579	2.025	0.446	0.000	0.000
RTN4RL2	3.320	4.117	0.797	0.000	0.000
RTP3	5.455	4.778	-0.676	0.000	0.000
RTP4	3.136	3.839	0.703	0.000	0.000
RTTN	1.987	2.125	0.138	0.000	0.000
RUFY1	3.619	4.552	0.933	0.000	0.000
RUFY2	1.662	2.248	0.586	0.000	0.000
RUFY3	3.379	3.439	0.060	0.144	0.154
RUFY4	0.420	0.530	0.110	0.001	0.001
RUNDC1	2.268	2.986	0.718	0.000	0.000
RUNDC3A	0.103	0.380	0.277	0.000	0.000
RUNDC3B	2.819	2.267	-0.552	0.000	0.000
RUNX1	1.839	2.308	0.469	0.000	0.000
RUNX1T1	0.540	0.526	-0.014	0.589	0.604
RUNX2	0.655	0.722	0.067	0.067	0.073
RUNX3	2.325	1.933	-0.392	0.000	0.000
RUSC1	2.406	3.821	1.415	0.000	0.000
RUSC2	3.288	3.394	0.106	0.017	0.019
RUVBL1	2.795	3.898	1.103	0.000	0.000
RUVBL2	4.781	5.776	0.996	0.000	0.000
RWDD1	4.141	4.478	0.337	0.000	0.000
RWDD2A	2.241	2.647	0.406	0.000	0.000
RWDD2B	3.874	3.901	0.028	0.368	0.384
RWDD3	3.632	4.046	0.414	0.000	0.000
RWDD4	3.117	3.310	0.193	0.000	0.000
RXFP1	0.555	0.267	-0.287	0.000	0.000
RXRA	5.442	5.626	0.184	0.000	0.000
RXRB	4.472	5.223	0.751	0.000	0.000
RXRG	0.577	0.832	0.255	0.000	0.000

RYBP	3.474	3.683	0.209	0.000	0.000
RYK	3.282	3.747	0.465	0.000	0.000
RYR1	0.332	0.337	0.005	0.825	0.832
RYR2	0.173	0.387	0.214	0.000	0.000
RYR3	0.188	0.232	0.043	0.004	0.004
S100A1	1.523	2.228	0.705	0.000	0.000
S100A11	6.581	7.309	0.728	0.000	0.000
S100A12	1.567	0.698	-0.869	0.000	0.000
S100A13	4.783	5.108	0.325	0.000	0.000
S100A14	4.224	4.103	-0.122	0.315	0.331
S100A16	6.709	7.151	0.441	0.000	0.000
S100A2	0.405	0.870	0.465	0.000	0.000
S100A3	0.285	0.932	0.647	0.000	0.000
S100A4	4.955	5.298	0.343	0.000	0.000
S100A6	6.120	6.658	0.538	0.000	0.000
S100A8	3.809	2.759	-1.050	0.000	0.000
S100A9	5.248	5.103	-0.145	0.204	0.217
S100B	1.107	1.097	-0.010	0.852	0.858
S100P	1.083	3.768	2.685	0.000	0.000
S100PBP	2.153	2.733	0.580	0.000	0.000
S100Z	0.188	0.202	0.014	0.308	0.323
S1PR1	4.212	4.092	-0.120	0.019	0.021
S1PR2	2.018	2.574	0.555	0.000	0.000
S1PR3	2.058	2.066	0.008	0.888	0.892
S1PR4	1.647	1.474	-0.173	0.001	0.001
S1PR5	1.079	0.914	-0.165	0.000	0.000
SAA1	9.685	8.269	-1.416	0.000	0.000
SAA2	6.747	5.385	-1.362	0.000	0.000
SAA2-SAA4	5.965	4.607	-1.358	0.000	0.000
SAA4	9.451	7.292	-2.159	0.000	0.000
SAAL1	2.470	3.451	0.980	0.000	0.000
SAC3D1	2.876	4.390	1.514	0.000	0.000
SACM1L	3.687	4.031	0.345	0.000	0.000
SACS	0.953	1.420	0.467	0.000	0.000
SAE1	4.353	5.799	1.446	0.000	0.000
SAFB	4.581	5.054	0.473	0.000	0.000
SAFB2	4.287	4.752	0.465	0.000	0.000
SALL1	4.552	4.243	-0.309	0.000	0.000
SALL2	0.766	1.438	0.671	0.000	0.000
SALL4	0.726	1.187	0.461	0.000	0.000
SAMD1	3.720	4.821	1.101	0.000	0.000
SAMD10	2.089	3.119	1.030	0.000	0.000
SAMD11	2.804	1.952	-0.852	0.000	0.000
SAMD12	0.970	0.793	-0.177	0.000	0.000
SAMD13	0.301	0.613	0.312	0.000	0.000
SAMD14	0.509	0.631	0.122	0.000	0.000
SAMD15	0.235	0.324	0.089	0.000	0.000
SAMD3	0.894	0.643	-0.251	0.000	0.000
SAMD4A	2.918	2.642	-0.276	0.000	0.000
SAMD4B	3.798	4.660	0.862	0.000	0.000
SAMD5	2.654	1.583	-1.072	0.000	0.000
SAMD8	2.583	2.749	0.166	0.000	0.000

SAMD9	2.003	2.026	0.023	0.661	0.674
SAMD9L	2.322	2.389	0.067	0.276	0.291
SAMHD1	4.206	4.762	0.556	0.000	0.000
SAMM50	3.853	4.758	0.906	0.000	0.000
SAMSN1	2.670	2.256	-0.414	0.000	0.000
SAP130	2.824	3.761	0.937	0.000	0.000
SAP18	6.124	6.507	0.383	0.000	0.000
SAP25	1.680	1.639	-0.041	0.389	0.405
SAP30	2.642	3.517	0.875	0.000	0.000
SAP30BP	3.849	4.616	0.767	0.000	0.000
SAR1A	4.888	5.342	0.454	0.000	0.000
SAR1B	5.382	5.541	0.159	0.000	0.000
SARDH	6.241	5.223	-1.018	0.000	0.000
SARNP	2.692	3.354	0.662	0.000	0.000
SARS	5.847	6.392	0.544	0.000	0.000
SARS2	2.773	3.284	0.511	0.000	0.000
SART1	4.683	5.462	0.778	0.000	0.000
SART3	3.321	4.017	0.697	0.000	0.000
SASH1	2.393	2.565	0.172	0.000	0.000
SASH3	3.027	2.872	-0.155	0.017	0.019
SASS6	1.208	2.148	0.940	0.000	0.000
SAT1	9.149	8.629	-0.519	0.000	0.000
SAT2	8.033	7.565	-0.469	0.000	0.000
SATB1	2.512	1.890	-0.622	0.000	0.000
SATB2	1.333	2.125	0.792	0.000	0.000
SAV1	3.204	3.291	0.087	0.016	0.018
SBDS	6.483	6.816	0.333	0.000	0.000
SBF1	3.462	4.307	0.845	0.000	0.000
SBF2	2.311	2.830	0.519	0.000	0.000
SBK1	0.447	0.776	0.330	0.000	0.000
SBNO1	2.719	3.276	0.556	0.000	0.000
SBNO2	3.882	4.256	0.374	0.000	0.000
SCAF1	4.266	5.179	0.913	0.000	0.000
SCAF11	3.894	4.264	0.370	0.000	0.000
SCAF4	3.558	3.892	0.333	0.000	0.000
SCAF8	3.311	3.694	0.383	0.000	0.000
SCAI	1.091	1.385	0.294	0.000	0.000
SCAMP1	3.318	3.975	0.657	0.000	0.000
SCAMP2	4.376	5.150	0.774	0.000	0.000
SCAMP3	4.958	6.661	1.703	0.000	0.000
SCAMP4	3.330	4.015	0.685	0.000	0.000
SCAMP5	1.948	3.064	1.116	0.000	0.000
SCAND1	5.795	6.232	0.436	0.000	0.000
SCAP	5.236	5.676	0.440	0.000	0.000
SCAPER	2.159	2.377	0.218	0.000	0.000
SCARA3	1.857	3.150	1.293	0.000	0.000
SCARA5	1.227	0.540	-0.688	0.000	0.000
SCARB1	6.284	6.925	0.641	0.000	0.000
SCARB2	6.438	6.694	0.256	0.000	0.000
SCARF1	3.041	2.885	-0.156	0.000	0.000
SCARF2	1.786	1.936	0.150	0.003	0.003
SCCPDH	6.820	7.541	0.721	0.000	0.000

SCD	7.427	8.563	1.135	0.000	0.000
SCD5	1.771	1.218	-0.553	0.000	0.000
SCFD1	3.980	4.563	0.583	0.000	0.000
SCFD2	2.564	3.147	0.583	0.000	0.000
SCG2	0.132	0.449	0.317	0.000	0.000
SCG5	3.185	2.867	-0.319	0.000	0.000
SCGB2A1	0.250	0.710	0.460	0.000	0.000
SCGB3A1	1.699	0.905	-0.794	0.000	0.000
SCGN	2.297	3.396	1.099	0.000	0.000
SCHIP1	0.871	1.078	0.208	0.000	0.000
SCIN	0.081	0.358	0.277	0.000	0.000
SCLT1	1.270	1.561	0.290	0.000	0.000
SCLY	2.086	2.682	0.596	0.000	0.000
SCMH1	3.120	3.713	0.593	0.000	0.000
SCML1	4.072	4.468	0.396	0.000	0.000
SCML2	0.876	1.967	1.091	0.000	0.000
SCML4	1.212	0.968	-0.245	0.000	0.000
SCN11A	0.285	0.204	-0.081	0.000	0.000
SCN1B	2.427	2.527	0.100	0.041	0.045
SCN2A	0.175	0.186	0.011	0.521	0.536
SCN4A	0.273	0.692	0.419	0.000	0.000
SCN4B	0.676	1.118	0.442	0.000	0.000
SCN7A	0.757	0.383	-0.374	0.000	0.000
SCN8A	1.093	1.487	0.394	0.000	0.000
SCN9A	1.785	1.599	-0.186	0.001	0.001
SCNM1	3.389	4.848	1.458	0.000	0.000
SCNN1A	2.038	1.884	-0.154	0.019	0.022
SCNN1B	0.398	0.414	0.016	0.600	0.614
SCNN1D	2.530	2.545	0.015	0.801	0.809
SCO1	3.072	3.279	0.207	0.000	0.000
SCO2	5.288	5.666	0.378	0.000	0.000
SCOC	4.341	4.662	0.321	0.000	0.000
SCP2	7.824	7.144	-0.680	0.000	0.000
SCPEP1	4.256	5.157	0.902	0.000	0.000
SCRIB	4.178	5.592	1.414	0.000	0.000
SCRN1	1.798	2.121	0.323	0.000	0.000
SCRN2	5.091	5.475	0.384	0.000	0.000
SCRN3	3.208	3.663	0.456	0.000	0.000
SCT	0.865	0.832	-0.032	0.485	0.500
SCTR	1.393	1.141	-0.252	0.001	0.002
SCUBE1	0.751	1.491	0.740	0.000	0.000
SCUBE2	0.601	0.871	0.270	0.000	0.000
SCYL1	5.672	5.928	0.257	0.000	0.000
SCYL2	3.563	3.953	0.390	0.000	0.000
SCYL3	2.641	3.111	0.471	0.000	0.000
SDAD1	3.673	4.222	0.549	0.000	0.000
SDC1	8.448	8.665	0.216	0.000	0.000
SDC2	7.158	7.935	0.777	0.000	0.000
SDC3	4.249	3.961	-0.288	0.000	0.000
SDC4	8.258	8.122	-0.136	0.005	0.006
SDCBP	5.831	6.657	0.826	0.000	0.000
SDCBP2	3.482	3.015	-0.467	0.000	0.000

SDCCAG8	3.068	3.440	0.371	0.000	0.000
SDF2	4.423	5.181	0.758	0.000	0.000
SDF2L1	5.624	6.861	1.237	0.000	0.000
SDF4	6.280	6.650	0.370	0.000	0.000
SDHA	6.484	6.265	-0.220	0.000	0.000
SDHAF1	4.463	5.071	0.608	0.000	0.000
SDHAF2	4.037	4.589	0.552	0.000	0.000
SDHB	7.213	6.885	-0.328	0.000	0.000
SDHC	4.729	5.492	0.763	0.000	0.000
SDK1	0.319	0.573	0.254	0.000	0.000
SDK2	0.285	0.348	0.062	0.061	0.067
SDR42E1	1.153	1.004	-0.149	0.000	0.000
SDR9C7	0.145	0.184	0.038	0.005	0.005
SDS	8.374	6.169	-2.205	0.000	0.000
SDSL	5.696	5.484	-0.213	0.001	0.002
SEC11A	5.110	5.847	0.738	0.000	0.000
SEC11C	5.511	6.436	0.925	0.000	0.000
SEC13	5.097	5.887	0.791	0.000	0.000
SEC14L1	3.137	3.496	0.359	0.000	0.000
SEC14L2	6.196	5.316	-0.880	0.000	0.000
SEC14L3	1.066	0.681	-0.384	0.000	0.000
SEC14L4	3.726	3.082	-0.644	0.000	0.000
SEC14L5	0.143	0.272	0.129	0.000	0.000
SEC14L6	0.492	0.523	0.031	0.495	0.510
SEC16A	5.010	5.360	0.350	0.000	0.000
SEC16B	3.273	3.497	0.223	0.000	0.000
SEC22A	2.696	3.377	0.681	0.000	0.000
SEC22C	2.596	3.231	0.635	0.000	0.000
SEC23A	4.590	4.722	0.133	0.002	0.002
SEC23B	4.261	5.203	0.942	0.000	0.000
SEC23IP	3.337	3.855	0.517	0.000	0.000
SEC24A	4.118	4.456	0.337	0.000	0.000
SEC24B	4.671	4.293	-0.378	0.000	0.000
SEC24C	4.393	5.155	0.762	0.000	0.000
SEC24D	4.051	3.986	-0.065	0.146	0.157
SEC31A	5.381	5.808	0.427	0.000	0.000
SEC31B	2.269	2.090	-0.180	0.000	0.000
SEC61A1	6.705	7.619	0.914	0.000	0.000
SEC61A2	2.483	2.988	0.505	0.000	0.000
SEC61B	7.617	8.196	0.579	0.000	0.000
SEC62	6.459	6.140	-0.319	0.000	0.000
SEC63	4.993	5.330	0.336	0.000	0.000
SECISBP2	3.733	4.056	0.323	0.000	0.000
SECISBP2L	3.321	3.589	0.268	0.000	0.000
SECTM1	2.889	3.597	0.708	0.000	0.000
SEH1L	2.921	3.414	0.493	0.000	0.000
SEL1L	5.143	5.413	0.270	0.000	0.000
SEL1L3	2.999	3.237	0.238	0.005	0.006
SELE	1.950	1.319	-0.631	0.000	0.000
SELENBP1	7.503	7.199	-0.304	0.000	0.000
SELL	2.738	2.399	-0.339	0.000	0.000
SELP	2.073	1.190	-0.883	0.000	0.000

SELPLG	3.239	3.156	-0.083	0.199	0.211
SEMA3A	0.267	0.478	0.211	0.000	0.000
SEMA3B	2.202	2.656	0.454	0.000	0.000
SEMA3C	0.851	0.954	0.103	0.104	0.112
SEMA3D	0.599	0.582	-0.016	0.653	0.666
SEMA3E	0.470	0.456	-0.014	0.785	0.794
SEMA3F	2.132	3.464	1.332	0.000	0.000
SEMA3G	1.529	2.372	0.844	0.000	0.000
SEMA4A	1.400	1.479	0.079	0.123	0.132
SEMA4B	3.784	3.967	0.183	0.001	0.001
SEMA4C	3.471	3.792	0.321	0.000	0.000
SEMA4D	1.707	1.585	-0.122	0.019	0.021
SEMA4F	0.821	1.744	0.923	0.000	0.000
SEMA4G	4.649	5.131	0.482	0.000	0.000
SEMA5A	1.913	1.850	-0.063	0.233	0.247
SEMA5B	0.667	1.684	1.017	0.000	0.000
SEMA6A	2.187	2.052	-0.136	0.041	0.045
SEMA6B	1.979	2.611	0.632	0.000	0.000
SEMA6C	2.852	3.500	0.648	0.000	0.000
SEMA6D	1.423	1.005	-0.418	0.000	0.000
SEMA7A	1.623	2.529	0.906	0.000	0.000
SENP1	1.804	2.571	0.767	0.000	0.000
SENP2	3.192	3.719	0.527	0.000	0.000
SENP3	3.136	3.844	0.708	0.000	0.000
SENP5	3.021	3.646	0.625	0.000	0.000
SENP6	3.615	3.934	0.320	0.000	0.000
SENP7	2.473	2.959	0.487	0.000	0.000
SENP8	0.950	1.004	0.054	0.010	0.011
SEPHS1	4.608	5.391	0.783	0.000	0.000
SEPHS2	8.378	8.772	0.394	0.000	0.000
SEPSECS	4.269	4.113	-0.156	0.000	0.000
SERAC1	1.291	1.826	0.534	0.000	0.000
SERBP1	5.908	6.445	0.538	0.000	0.000
SERF1B	0.906	1.227	0.321	0.000	0.000
SERGEF	3.194	3.778	0.584	0.000	0.000
SERHL2	1.082	1.221	0.138	0.001	0.001
SERINC1	7.143	7.084	-0.059	0.125	0.135
SERINC2	6.282	6.894	0.611	0.000	0.000
SERINC3	5.518	6.019	0.501	0.000	0.000
SERINC5	3.777	4.377	0.600	0.000	0.000
SERP1	6.316	6.698	0.382	0.000	0.000
SERP2	1.789	1.730	-0.059	0.223	0.236
SERPINA1	13.645	13.042	-0.603	0.000	0.000
SERPINA10	7.158	6.508	-0.651	0.000	0.000
SERPINA11	8.852	6.702	-2.150	0.000	0.000
SERPINA12	1.085	1.323	0.238	0.002	0.002
SERPINA3	8.017	7.300	-0.718	0.000	0.000
SERPINA4	8.481	7.181	-1.299	0.000	0.000
SERPINA5	8.322	7.709	-0.613	0.000	0.000
SERPINA6	9.164	8.638	-0.526	0.000	0.000
SERPINA7	7.582	6.773	-0.809	0.000	0.000
SERPINA9	0.227	0.253	0.026	0.200	0.212

SERPINB1	4.975	5.697	0.722	0.000	0.000
SERPINB8	3.438	2.667	-0.771	0.000	0.000
SERPINB9	3.807	3.125	-0.682	0.000	0.000
SERPINC1	11.754	10.927	-0.828	0.000	0.000
SERPIND1	9.291	9.109	-0.182	0.044	0.048
SERPINE1	6.823	5.882	-0.941	0.000	0.000
SERPINE2	2.137	2.839	0.702	0.000	0.000
SERPINF1	9.264	9.097	-0.167	0.005	0.005
SERPINF2	10.440	9.514	-0.927	0.000	0.000
SERPING1	11.056	10.197	-0.859	0.000	0.000
SERPINH1	4.507	5.685	1.178	0.000	0.000
SERPINI1	1.567	3.053	1.485	0.000	0.000
SERTAD1	4.472	3.867	-0.605	0.000	0.000
SERTAD2	3.353	3.754	0.401	0.000	0.000
SERTAD3	3.740	4.242	0.502	0.000	0.000
SERTAD4	0.200	0.331	0.131	0.000	0.000
SESN1	3.849	3.874	0.025	0.572	0.586
SESN2	3.858	4.150	0.292	0.000	0.000
SESN3	1.765	1.861	0.097	0.102	0.110
SESTD1	2.133	3.272	1.138	0.000	0.000
SET	5.914	6.740	0.827	0.000	0.000
SETBP1	1.961	1.894	-0.068	0.102	0.110
SETD1A	2.867	3.458	0.590	0.000	0.000
SETD1B	2.846	3.306	0.459	0.000	0.000
SETD2	3.602	4.119	0.517	0.000	0.000
SETD3	4.643	4.849	0.206	0.000	0.000
SETD4	1.948	2.562	0.614	0.000	0.000
SETD5	3.829	4.348	0.519	0.000	0.000
SETD6	2.919	3.379	0.460	0.000	0.000
SETD7	4.017	4.135	0.118	0.003	0.004
SETDB1	3.206	4.334	1.128	0.000	0.000
SETDB2	2.355	2.697	0.342	0.000	0.000
SETMAR	2.577	3.167	0.591	0.000	0.000
SETX	3.776	3.968	0.192	0.000	0.000
SEZ6	0.084	0.669	0.585	0.000	0.000
SEZ6L2	1.322	2.918	1.596	0.000	0.000
SF1	5.919	6.202	0.283	0.000	0.000
SF3A1	4.376	5.108	0.732	0.000	0.000
SF3A2	4.210	5.293	1.083	0.000	0.000
SF3A3	4.242	5.129	0.887	0.000	0.000
SF3B1	6.001	6.453	0.452	0.000	0.000
SF3B2	5.115	5.821	0.706	0.000	0.000
SF3B3	4.082	4.699	0.618	0.000	0.000
SF3B4	4.689	6.166	1.476	0.000	0.000
SF3B5	7.231	7.899	0.668	0.000	0.000
SFI1	1.819	2.664	0.845	0.000	0.000
SFMBT1	2.474	2.995	0.521	0.000	0.000
SFMBT2	1.191	1.240	0.049	0.260	0.274
SFN	1.298	4.002	2.703	0.000	0.000
SFPQ	5.600	6.085	0.486	0.000	0.000
SFRP1	1.825	0.643	-1.181	0.000	0.000
SFRP4	0.660	1.777	1.117	0.000	0.000

SFRP5	4.252	1.197	-3.055	0.000	0.000
SFSWAP	2.939	3.635	0.696	0.000	0.000
SFT2D1	3.591	3.906	0.315	0.000	0.000
SFT2D2	2.414	3.002	0.588	0.000	0.000
SFTPD	1.186	0.798	-0.388	0.000	0.000
SFXN1	5.128	4.937	-0.190	0.000	0.000
SFXN2	3.215	3.115	-0.100	0.021	0.023
SFXN3	2.667	3.164	0.496	0.000	0.000
SFXN4	4.889	5.528	0.639	0.000	0.000
SFXN5	4.297	4.169	-0.128	0.001	0.001
SGCA	1.929	1.082	-0.847	0.000	0.000
SGCB	2.904	2.795	-0.108	0.098	0.106
SGCD	0.779	0.586	-0.193	0.000	0.000
SGCE	3.364	4.361	0.997	0.000	0.000
SGIP1	0.522	0.794	0.272	0.000	0.000
SGK1	4.783	4.511	-0.272	0.000	0.000
SGK2	4.550	4.671	0.120	0.023	0.026
SGK3	2.797	3.327	0.530	0.000	0.000
SGK494	1.110	1.473	0.362	0.000	0.000
SGMS1	3.368	3.840	0.472	0.000	0.000
SGMS2	3.035	2.634	-0.401	0.000	0.000
SGPL1	3.915	4.547	0.632	0.000	0.000
SGPP1	4.310	4.205	-0.105	0.007	0.008
SGPP2	0.662	0.838	0.175	0.005	0.006
SGSH	3.161	3.814	0.653	0.000	0.000
SGSM1	0.281	0.631	0.350	0.000	0.000
SGSM2	3.311	3.583	0.272	0.000	0.000
SGSM3	4.359	5.098	0.739	0.000	0.000
SGTA	4.924	5.637	0.714	0.000	0.000
SGTB	1.483	1.959	0.476	0.000	0.000
SH2B1	3.623	4.262	0.639	0.000	0.000
SH2B2	1.468	2.028	0.560	0.000	0.000
SH2B3	3.814	4.107	0.293	0.000	0.000
SH2D1A	1.857	1.394	-0.463	0.000	0.000
SH2D1B	0.745	0.503	-0.242	0.000	0.000
SH2D2A	1.880	1.863	-0.017	0.774	0.784
SH2D3A	1.304	1.439	0.135	0.020	0.022
SH2D3C	2.343	2.853	0.509	0.000	0.000
SH2D4A	4.079	4.256	0.177	0.000	0.000
SH2D5	0.099	0.248	0.149	0.000	0.000
SH2D6	0.340	0.440	0.100	0.000	0.000
SH3BGR	2.748	2.890	0.142	0.000	0.000
SH3BGRL	5.096	5.098	0.002	0.972	0.973
SH3BGRL2	5.120	5.065	-0.055	0.283	0.298
SH3BGRL3	6.130	6.702	0.572	0.000	0.000
SH3BP1	1.809	2.232	0.423	0.000	0.000
SH3BP2	3.576	3.600	0.024	0.582	0.596
SH3BP4	3.974	4.665	0.691	0.000	0.000
SH3BP5L	2.995	4.113	1.118	0.000	0.000
SH3D19	4.652	4.604	-0.047	0.252	0.266
SH3D21	0.803	1.390	0.587	0.000	0.000
SH3GL1	4.719	5.310	0.591	0.000	0.000

SH3GL2	0.281	0.189	-0.092	0.000	0.000
SH3GLB1	4.296	4.740	0.444	0.000	0.000
SH3GLB2	3.844	4.473	0.629	0.000	0.000
SH3KBP1	3.191	3.868	0.677	0.000	0.000
SH3PXD2A	2.859	3.388	0.529	0.000	0.000
SH3PXD2B	1.519	2.555	1.037	0.000	0.000
SH3RF1	2.683	2.650	-0.033	0.490	0.506
SH3RF2	2.007	2.549	0.542	0.000	0.000
SH3RF3	1.262	1.618	0.356	0.000	0.000
SH3TC1	3.318	3.188	-0.131	0.003	0.003
SH3TC2	0.172	0.247	0.075	0.000	0.000
SH3YL1	2.805	2.217	-0.588	0.000	0.000
SHANK2	1.980	2.574	0.594	0.000	0.000
SHARPIN	4.898	6.151	1.253	0.000	0.000
SHB	4.761	4.632	-0.129	0.004	0.005
SHBG	6.110	3.746	-2.363	0.000	0.000
SHC1	5.338	6.637	1.299	0.000	0.000
SHC2	5.430	5.285	-0.145	0.013	0.014
SHC3	0.166	0.430	0.264	0.000	0.000
SHC4	0.166	0.314	0.148	0.000	0.000
SHCBP1	0.483	1.731	1.248	0.000	0.000
SHD	2.670	2.491	-0.179	0.021	0.023
SHE	2.168	2.273	0.104	0.031	0.034
SHF	3.474	3.342	-0.131	0.017	0.020
SHH	2.675	2.521	-0.154	0.004	0.004
SHISA2	0.202	0.410	0.208	0.000	0.000
SHISA3	0.980	1.163	0.183	0.005	0.006
SHISA4	2.737	3.981	1.243	0.000	0.000
SHISA5	5.926	6.549	0.623	0.000	0.000
SHKBP1	3.841	4.861	1.020	0.000	0.000
SHMT1	7.187	6.229	-0.958	0.000	0.000
SHMT2	6.670	6.750	0.080	0.024	0.027
SHOC2	4.000	4.367	0.367	0.000	0.000
SHOX2	0.029	0.346	0.317	0.000	0.000
SHPK	3.355	2.933	-0.422	0.000	0.000
SHPRH	1.137	1.331	0.194	0.000	0.000
SHQ1	2.183	2.862	0.679	0.000	0.000
SHROOM1	5.108	5.701	0.593	0.000	0.000
SHROOM2	1.946	1.977	0.031	0.525	0.540
SHROOM3	2.741	2.762	0.021	0.676	0.689
SHROOM4	0.836	1.168	0.332	0.000	0.000
SIAE	4.174	4.125	-0.049	0.307	0.322
SIAH1	3.414	3.517	0.103	0.003	0.003
SIAH2	5.481	6.134	0.653	0.000	0.000
SIDT1	0.736	0.648	-0.088	0.006	0.007
SIDT2	3.723	3.986	0.263	0.000	0.000
SIGIRR	5.771	5.105	-0.665	0.000	0.000
SIGLEC1	3.270	2.445	-0.825	0.000	0.000
SIGLEC10	1.822	1.513	-0.309	0.000	0.000
SIGLEC11	1.610	0.724	-0.887	0.000	0.000
SIGLEC12	0.788	0.929	0.142	0.002	0.002
SIGLEC14	1.210	0.923	-0.287	0.000	0.000

SIGLEC15	0.538	1.063	0.524	0.000	0.000
SIGLEC5	0.701	0.544	-0.158	0.000	0.000
SIGLEC7	2.460	1.631	-0.829	0.000	0.000
SIGLEC8	0.744	0.694	-0.049	0.225	0.238
SIGLEC9	2.002	1.545	-0.457	0.000	0.000
SIGMAR1	7.010	7.646	0.636	0.000	0.000
SIK1	4.449	3.971	-0.478	0.000	0.000
SIK2	4.097	4.453	0.355	0.000	0.000
SIK3	3.341	3.442	0.101	0.004	0.004
SIKE1	3.583	3.993	0.410	0.000	0.000
SIL1	5.770	6.286	0.516	0.000	0.000
SIM2	0.346	0.555	0.209	0.000	0.000
SIN3A	2.874	3.368	0.494	0.000	0.000
SIN3B	3.084	3.661	0.577	0.000	0.000
SIPA1	4.461	4.902	0.440	0.000	0.000
SIPA1L1	3.164	3.557	0.393	0.000	0.000
SIPA1L2	2.199	3.286	1.087	0.000	0.000
SIPA1L3	1.838	2.964	1.126	0.000	0.000
SIRPA	4.559	4.861	0.302	0.000	0.000
SIRPB1	0.694	0.573	-0.120	0.000	0.000
SIRPB2	0.714	0.695	-0.019	0.585	0.599
SIRPG	1.144	1.250	0.106	0.061	0.067
SIRT1	3.411	3.559	0.148	0.000	0.000
SIRT2	4.347	4.780	0.433	0.000	0.000
SIRT3	4.053	4.148	0.096	0.001	0.001
SIRT4	2.038	2.672	0.634	0.000	0.000
SIRT5	4.183	4.108	-0.076	0.041	0.045
SIRT6	3.191	4.022	0.832	0.000	0.000
SIRT7	2.854	3.920	1.066	0.000	0.000
SIT1	1.608	1.656	0.048	0.441	0.457
SIVA1	4.901	5.217	0.316	0.000	0.000
SIX1	0.075	0.584	0.509	0.000	0.000
SIX2	0.067	0.622	0.555	0.000	0.000
SIX4	0.168	0.748	0.580	0.000	0.000
SKA1	0.446	2.274	1.828	0.000	0.000
SKA2	3.473	4.400	0.927	0.000	0.000
SKA3	0.476	2.174	1.698	0.000	0.000
SKAP1	4.338	2.946	-1.392	0.000	0.000
SKAP2	2.747	3.463	0.716	0.000	0.000
SKI	3.901	4.329	0.428	0.000	0.000
SKIL	3.000	3.360	0.360	0.000	0.000
SKIV2L	4.877	5.489	0.612	0.000	0.000
SKOR1	0.191	0.275	0.084	0.000	0.000
SKP1	5.174	6.014	0.840	0.000	0.000
SKP2	2.704	3.597	0.893	0.000	0.000
SLA	2.503	2.195	-0.308	0.000	0.000
SLA2	1.412	1.303	-0.109	0.021	0.023
SLAIN1	1.888	1.361	-0.527	0.000	0.000
SLAIN2	3.709	4.139	0.430	0.000	0.000
SLAMF1	1.016	0.970	-0.046	0.266	0.280
SLAMF6	2.026	1.613	-0.413	0.000	0.000
SLAMF7	2.606	2.198	-0.408	0.000	0.000

SLAMF8	1.950	2.727	0.777	0.000	0.000
SLAMF9	0.885	1.173	0.288	0.000	0.000
SLBP	4.578	5.641	1.063	0.000	0.000
SLC10A1	8.136	5.814	-2.322	0.000	0.000
SLC10A3	3.112	3.759	0.647	0.000	0.000
SLC10A4	0.135	0.265	0.130	0.000	0.000
SLC10A5	1.698	2.105	0.407	0.000	0.000
SLC10A6	0.295	0.393	0.097	0.000	0.000
SLC10A7	1.906	2.184	0.278	0.000	0.000
SLC11A1	1.104	1.143	0.039	0.444	0.460
SLC11A2	3.420	4.017	0.597	0.000	0.000
SLC12A2	2.277	2.544	0.266	0.000	0.000
SLC12A4	3.711	3.852	0.141	0.000	0.000
SLC12A5	0.294	0.552	0.259	0.000	0.000
SLC12A6	1.859	2.189	0.331	0.000	0.000
SLC12A7	5.071	5.885	0.814	0.000	0.000
SLC12A8	2.114	2.885	0.771	0.000	0.000
SLC12A9	2.687	3.728	1.041	0.000	0.000
SLC13A2	0.900	1.185	0.285	0.000	0.000
SLC13A3	2.796	3.114	0.318	0.006	0.007
SLC13A5	6.729	5.393	-1.336	0.000	0.000
SLC14A1	1.042	0.696	-0.346	0.000	0.000
SLC15A1	3.676	3.646	-0.030	0.708	0.719
SLC15A2	0.703	0.827	0.124	0.000	0.000
SLC15A3	3.332	3.481	0.149	0.005	0.006
SLC15A4	3.458	3.906	0.449	0.000	0.000
SLC16A1	5.191	5.347	0.156	0.010	0.011
SLC16A10	1.520	1.469	-0.051	0.272	0.286
SLC16A11	3.089	3.581	0.492	0.000	0.000
SLC16A12	0.910	1.347	0.437	0.000	0.000
SLC16A13	4.897	4.572	-0.324	0.000	0.000
SLC16A14	1.309	1.491	0.182	0.003	0.003
SLC16A2	5.481	4.830	-0.650	0.000	0.000
SLC16A3	1.593	2.495	0.902	0.000	0.000
SLC16A4	2.619	1.688	-0.930	0.000	0.000
SLC16A5	1.720	1.398	-0.322	0.000	0.000
SLC16A6	0.770	1.074	0.304	0.000	0.000
SLC16A7	1.078	1.202	0.124	0.008	0.009
SLC16A9	0.953	1.256	0.302	0.000	0.000
SLC17A1	4.637	3.860	-0.777	0.000	0.000
SLC17A2	5.478	4.615	-0.862	0.000	0.000
SLC17A3	3.954	3.261	-0.692	0.000	0.000
SLC17A4	4.505	4.893	0.388	0.000	0.000
SLC17A5	3.788	4.885	1.097	0.000	0.000
SLC17A7	0.201	0.221	0.020	0.199	0.211
SLC17A9	5.539	5.480	-0.059	0.419	0.436
SLC19A1	2.404	3.000	0.596	0.000	0.000
SLC19A2	4.484	4.748	0.263	0.000	0.000
SLC19A3	3.983	2.947	-1.036	0.000	0.000
SLC1A1	4.853	3.844	-1.009	0.000	0.000
SLC1A2	3.870	2.998	-0.872	0.000	0.000
SLC1A3	1.254	2.319	1.065	0.000	0.000

SLC1A4	2.468	3.552	1.083	0.000	0.000
SLC1A5	2.951	3.653	0.703	0.000	0.000
SLC1A7	0.946	1.318	0.373	0.000	0.000
SLC20A1	4.667	4.775	0.108	0.028	0.032
SLC20A2	4.260	4.441	0.181	0.000	0.000
SLC22A1	9.358	5.746	-3.611	0.000	0.000
SLC22A10	5.835	3.635	-2.199	0.000	0.000
SLC22A11	0.643	1.838	1.195	0.000	0.000
SLC22A12	0.422	1.149	0.727	0.000	0.000
SLC22A15	0.969	1.557	0.588	0.000	0.000
SLC22A17	1.881	1.585	-0.297	0.000	0.000
SLC22A18	5.031	5.445	0.413	0.000	0.000
SLC22A23	2.987	4.005	1.018	0.000	0.000
SLC22A24	0.227	0.218	-0.010	0.516	0.531
SLC22A25	3.220	2.930	-0.290	0.000	0.000
SLC22A3	4.878	4.679	-0.199	0.005	0.005
SLC22A4	0.899	1.425	0.525	0.000	0.000
SLC22A5	1.718	2.353	0.636	0.000	0.000
SLC22A7	7.808	6.565	-1.244	0.000	0.000
SLC22A9	4.693	4.965	0.272	0.001	0.001
SLC23A1	3.888	4.155	0.267	0.000	0.000
SLC23A2	5.218	4.730	-0.487	0.000	0.000
SLC23A3	1.376	1.395	0.019	0.730	0.740
SLC24A1	1.530	1.846	0.315	0.000	0.000
SLC24A3	0.724	0.660	-0.064	0.153	0.164
SLC25A1	6.979	7.535	0.556	0.000	0.000
SLC25A10	5.622	5.994	0.372	0.000	0.000
SLC25A11	5.464	5.724	0.259	0.000	0.000
SLC25A12	1.418	1.968	0.551	0.000	0.000
SLC25A13	6.256	6.356	0.100	0.018	0.021
SLC25A14	2.039	2.736	0.698	0.000	0.000
SLC25A15	6.052	5.649	-0.403	0.000	0.000
SLC25A16	3.290	3.621	0.332	0.000	0.000
SLC25A17	3.675	4.119	0.444	0.000	0.000
SLC25A18	5.717	4.686	-1.031	0.000	0.000
SLC25A19	2.044	3.068	1.024	0.000	0.000
SLC25A20	6.728	6.282	-0.446	0.000	0.000
SLC25A21	0.191	0.305	0.114	0.000	0.000
SLC25A22	3.991	4.237	0.246	0.000	0.000
SLC25A23	4.268	4.770	0.501	0.000	0.000
SLC25A24	1.607	1.963	0.356	0.000	0.000
SLC25A25	5.750	5.237	-0.513	0.000	0.000
SLC25A26	3.379	3.577	0.198	0.000	0.000
SLC25A27	3.501	3.022	-0.479	0.000	0.000
SLC25A28	5.549	5.725	0.176	0.000	0.000
SLC25A29	3.167	3.797	0.630	0.000	0.000
SLC25A3	6.471	7.252	0.782	0.000	0.000
SLC25A30	3.955	4.397	0.442	0.000	0.000
SLC25A32	3.775	4.217	0.442	0.000	0.000
SLC25A33	3.168	4.128	0.960	0.000	0.000
SLC25A34	1.835	1.617	-0.218	0.000	0.000
SLC25A35	1.685	2.366	0.681	0.000	0.000

SLC25A36	1.750	1.785	0.036	0.523	0.538
SLC25A37	3.009	2.726	-0.284	0.000	0.000
SLC25A38	5.230	5.510	0.280	0.000	0.000
SLC25A39	5.741	7.064	1.324	0.000	0.000
SLC25A4	4.727	4.749	0.023	0.521	0.536
SLC25A40	2.314	3.236	0.922	0.000	0.000
SLC25A41	0.354	0.348	-0.006	0.800	0.808
SLC25A42	5.387	5.151	-0.236	0.000	0.000
SLC25A43	2.985	4.025	1.040	0.000	0.000
SLC25A44	3.900	4.816	0.916	0.000	0.000
SLC25A45	2.642	2.829	0.187	0.000	0.000
SLC25A46	3.386	3.798	0.412	0.000	0.000
SLC25A47	8.787	5.016	-3.771	0.000	0.000
SLC25A48	0.279	0.275	-0.004	0.875	0.880
SLC25A5	8.175	8.894	0.719	0.000	0.000
SLC25A6	3.560	3.730	0.170	0.000	0.000
SLC26A1	3.519	3.666	0.147	0.005	0.006
SLC26A10	0.765	0.713	-0.052	0.101	0.109
SLC26A11	2.747	3.600	0.853	0.000	0.000
SLC26A2	1.175	2.127	0.953	0.000	0.000
SLC26A3	0.331	0.855	0.524	0.000	0.000
SLC26A4	0.149	0.185	0.036	0.000	0.000
SLC26A5	0.274	0.202	-0.072	0.000	0.000
SLC26A6	1.925	3.837	1.912	0.000	0.000
SLC26A7	0.160	0.349	0.189	0.000	0.000
SLC26A8	0.134	0.175	0.041	0.000	0.000
SLC27A1	2.345	2.569	0.224	0.000	0.000
SLC27A2	7.609	6.470	-1.139	0.000	0.000
SLC27A3	4.784	5.137	0.353	0.000	0.000
SLC27A4	4.623	4.999	0.376	0.000	0.000
SLC27A5	8.245	6.373	-1.872	0.000	0.000
SLC28A1	5.067	3.506	-1.561	0.000	0.000
SLC28A2	0.265	0.402	0.137	0.000	0.000
SLC28A3	0.627	0.412	-0.215	0.000	0.000
SLC29A1	5.146	6.282	1.136	0.000	0.000
SLC29A2	2.524	3.295	0.771	0.000	0.000
SLC29A3	2.986	3.438	0.453	0.000	0.000
SLC29A4	1.575	2.415	0.840	0.000	0.000
SLC2A1	1.523	2.329	0.806	0.000	0.000
SLC2A10	4.201	4.226	0.025	0.701	0.713
SLC2A11	1.316	1.785	0.469	0.000	0.000
SLC2A12	1.943	2.089	0.146	0.023	0.026
SLC2A13	1.910	2.186	0.276	0.000	0.000
SLC2A14	0.249	0.484	0.235	0.000	0.000
SLC2A2	8.434	7.733	-0.700	0.000	0.000
SLC2A3	3.083	2.932	-0.151	0.037	0.041
SLC2A4	1.214	1.761	0.547	0.000	0.000
SLC2A4RG	6.771	7.248	0.477	0.000	0.000
SLC2A5	0.364	1.449	1.085	0.000	0.000
SLC2A6	1.882	2.676	0.794	0.000	0.000
SLC2A8	3.974	4.406	0.432	0.000	0.000
SLC2A9	3.301	2.669	-0.631	0.000	0.000

SLC30A1	5.093	5.027	-0.067	0.228	0.241
SLC30A10	4.048	4.726	0.678	0.000	0.000
SLC30A2	0.300	0.695	0.395	0.000	0.000
SLC30A3	0.050	0.491	0.440	0.000	0.000
SLC30A4	1.040	1.098	0.057	0.140	0.150
SLC30A5	3.956	4.605	0.649	0.000	0.000
SLC30A6	2.942	3.737	0.795	0.000	0.000
SLC30A7	3.143	3.627	0.485	0.000	0.000
SLC30A9	3.683	4.170	0.487	0.000	0.000
SLC31A1	5.746	5.611	-0.135	0.001	0.001
SLC33A1	3.285	4.058	0.773	0.000	0.000
SLC34A1	0.676	0.425	-0.251	0.000	0.000
SLC34A2	1.247	0.798	-0.450	0.000	0.000
SLC34A3	0.076	0.218	0.142	0.000	0.000
SLC35A1	4.144	4.345	0.201	0.000	0.000
SLC35A2	3.774	4.700	0.926	0.000	0.000
SLC35A3	3.606	3.817	0.211	0.000	0.000
SLC35A4	4.609	5.458	0.849	0.000	0.000
SLC35A5	3.486	3.829	0.343	0.000	0.000
SLC35B1	4.577	5.409	0.833	0.000	0.000
SLC35B2	4.836	5.973	1.137	0.000	0.000
SLC35B3	4.386	5.080	0.694	0.000	0.000
SLC35B4	3.040	3.623	0.583	0.000	0.000
SLC35C1	5.023	5.411	0.387	0.000	0.000
SLC35C2	3.924	4.683	0.760	0.000	0.000
SLC35D1	5.229	4.752	-0.476	0.000	0.000
SLC35D2	5.528	6.133	0.605	0.000	0.000
SLC35E1	3.498	4.205	0.706	0.000	0.000
SLC35E2B	3.083	3.588	0.506	0.000	0.000
SLC35E3	1.331	2.000	0.668	0.000	0.000
SLC35E4	0.437	0.974	0.538	0.000	0.000
SLC35F1	0.146	0.183	0.037	0.029	0.032
SLC35F2	0.903	1.099	0.196	0.000	0.001
SLC35F3	0.178	0.289	0.112	0.001	0.001
SLC35F5	3.495	4.034	0.539	0.000	0.000
SLC36A1	1.481	2.380	0.899	0.000	0.000
SLC36A4	1.572	1.828	0.256	0.000	0.000
SLC37A1	1.829	2.444	0.615	0.000	0.000
SLC37A2	1.738	1.784	0.047	0.332	0.347
SLC37A3	2.319	2.869	0.551	0.000	0.000
SLC37A4	6.267	5.947	-0.320	0.000	0.000
SLC38A1	2.438	3.214	0.776	0.000	0.000
SLC38A10	5.819	6.319	0.500	0.000	0.000
SLC38A11	1.119	1.321	0.203	0.000	0.000
SLC38A2	7.371	6.468	-0.903	0.000	0.000
SLC38A3	7.914	7.808	-0.106	0.236	0.249
SLC38A4	8.092	6.948	-1.144	0.000	0.000
SLC38A5	0.821	1.047	0.226	0.000	0.000
SLC38A6	1.735	2.990	1.255	0.000	0.000
SLC38A7	2.606	3.423	0.817	0.000	0.000
SLC38A9	2.378	3.207	0.829	0.000	0.000
SLC39A1	5.442	6.827	1.385	0.000	0.000

SLC39A10	1.580	2.501	0.921	0.000	0.000
SLC39A11	4.308	4.643	0.335	0.000	0.000
SLC39A13	3.348	4.384	1.036	0.000	0.000
SLC39A14	7.491	6.785	-0.706	0.000	0.000
SLC39A3	3.120	4.047	0.927	0.000	0.000
SLC39A4	2.249	2.835	0.586	0.000	0.000
SLC39A5	7.119	5.642	-1.477	0.000	0.000
SLC39A6	3.463	4.392	0.929	0.000	0.000
SLC39A7	6.157	7.297	1.139	0.000	0.000
SLC39A8	3.972	3.771	-0.201	0.000	0.000
SLC39A9	4.617	5.153	0.536	0.000	0.000
SLC3A1	2.376	1.869	-0.507	0.000	0.000
SLC3A2	5.218	5.781	0.564	0.000	0.000
SLC40A1	7.033	7.136	0.104	0.047	0.052
SLC41A1	1.909	2.801	0.892	0.000	0.000
SLC41A2	4.997	4.391	-0.605	0.000	0.000
SLC41A3	2.792	4.015	1.223	0.000	0.000
SLC43A1	6.411	6.663	0.252	0.000	0.000
SLC43A2	1.743	2.495	0.752	0.000	0.000
SLC43A3	5.537	5.709	0.172	0.003	0.004
SLC44A1	3.731	4.099	0.367	0.000	0.000
SLC44A2	3.935	4.180	0.245	0.000	0.000
SLC44A3	2.615	3.648	1.033	0.000	0.000
SLC44A4	0.581	0.769	0.188	0.001	0.001
SLC44A5	0.346	1.376	1.030	0.000	0.000
SLC45A1	0.466	0.747	0.281	0.000	0.000
SLC45A2	0.679	0.898	0.219	0.000	0.000
SLC45A3	3.636	3.140	-0.496	0.000	0.000
SLC45A4	0.783	1.577	0.794	0.000	0.000
SLC46A1	3.669	4.350	0.681	0.000	0.000
SLC46A2	0.243	0.416	0.172	0.000	0.000
SLC46A3	5.490	4.371	-1.119	0.000	0.000
SLC47A1	5.489	5.116	-0.372	0.000	0.000
SLC47A2	0.108	0.276	0.167	0.000	0.000
SLC48A1	2.959	3.821	0.861	0.000	0.000
SLC4A10	0.283	0.174	-0.109	0.000	0.000
SLC4A11	0.299	0.936	0.638	0.000	0.000
SLC4A1AP	3.173	3.968	0.795	0.000	0.000
SLC4A2	4.931	5.976	1.046	0.000	0.000
SLC4A3	0.473	0.775	0.302	0.000	0.000
SLC4A4	3.944	2.826	-1.118	0.000	0.000
SLC4A5	0.401	0.561	0.160	0.000	0.000
SLC4A7	1.844	2.146	0.302	0.000	0.000
SLC50A1	4.950	6.465	1.515	0.000	0.000
SLC5A1	1.741	0.510	-1.231	0.000	0.000
SLC5A10	0.708	0.896	0.188	0.000	0.000
SLC5A11	0.459	1.223	0.763	0.000	0.000
SLC5A12	0.554	0.428	-0.126	0.006	0.007
SLC5A2	1.339	1.635	0.296	0.000	0.000
SLC5A3	1.802	1.671	-0.130	0.001	0.001
SLC5A4	0.476	0.628	0.153	0.000	0.000
SLC5A6	4.587	5.388	0.801	0.000	0.000

SLC5A9	3.188	3.649	0.461	0.000	0.000
SLC6A1	5.757	5.775	0.018	0.838	0.845
SLC6A11	0.922	1.204	0.282	0.001	0.001
SLC6A12	5.031	4.068	-0.963	0.000	0.000
SLC6A13	3.619	2.269	-1.351	0.000	0.000
SLC6A16	2.545	2.130	-0.416	0.000	0.000
SLC6A19	1.151	0.530	-0.621	0.000	0.000
SLC6A2	0.411	0.783	0.372	0.000	0.000
SLC6A6	1.623	2.041	0.418	0.000	0.000
SLC6A8	1.486	2.793	1.307	0.000	0.000
SLC6A9	0.734	1.574	0.840	0.000	0.000
SLC7A1	1.317	1.821	0.505	0.000	0.000
SLC7A10	0.261	0.739	0.478	0.000	0.000
SLC7A11	0.340	1.313	0.972	0.000	0.000
SLC7A2	6.609	5.307	-1.302	0.000	0.000
SLC7A4	0.404	0.220	-0.183	0.000	0.000
SLC7A5	3.060	3.159	0.099	0.170	0.181
SLC7A6	1.592	2.463	0.872	0.000	0.000
SLC7A7	2.757	2.656	-0.100	0.113	0.121
SLC7A8	2.456	1.794	-0.662	0.000	0.000
SLC7A9	3.604	4.031	0.428	0.000	0.000
SLC8A1	0.841	0.665	-0.177	0.000	0.000
SLC9A1	2.040	2.392	0.351	0.000	0.000
SLC9A3	0.713	1.239	0.527	0.000	0.000
SLC9A3R1	7.050	7.934	0.885	0.000	0.000
SLC9A3R2	7.503	7.017	-0.486	0.000	0.000
SLC9A5	0.314	0.423	0.109	0.000	0.000
SLC9A6	2.315	3.181	0.866	0.000	0.000
SLC9A7	1.293	1.473	0.180	0.000	0.000
SLC9A8	3.091	3.496	0.405	0.000	0.000
SLC9A9	2.349	1.880	-0.469	0.000	0.000
SLCO1A2	1.137	1.192	0.055	0.263	0.277
SLCO1B1	7.718	6.611	-1.106	0.000	0.000
SLCO1B3	5.894	2.832	-3.062	0.000	0.000
SLCO1C1	0.044	0.297	0.253	0.000	0.000
SLCO2A1	1.646	2.379	0.733	0.000	0.000
SLCO2B1	6.127	5.612	-0.515	0.000	0.000
SLCO3A1	1.874	2.235	0.361	0.000	0.000
SLCO4A1	1.289	0.925	-0.364	0.000	0.000
SLCO4C1	1.635	1.046	-0.589	0.000	0.000
SLFN11	2.361	2.318	-0.043	0.446	0.462
SLFN12	1.250	1.403	0.153	0.001	0.001
SLFN12L	0.506	0.408	-0.098	0.000	0.000
SLFN13	1.601	1.582	-0.019	0.801	0.809
SLFN5	1.966	2.035	0.069	0.162	0.173
SLFNL1	0.676	0.626	-0.051	0.056	0.062
SLIRP	5.204	5.754	0.551	0.000	0.000
SLIT1	0.134	0.148	0.015	0.253	0.267
SLIT2	0.801	0.817	0.016	0.719	0.730
SLIT3	0.927	1.162	0.235	0.000	0.000
SLITRK3	0.826	0.433	-0.393	0.000	0.000
SLITRK6	0.554	0.119	-0.435	0.000	0.000

SLK	3.561	3.949	0.388	0.000	0.000
SLMAP	2.513	3.117	0.604	0.000	0.000
SLPI	7.674	7.299	-0.375	0.012	0.013
SLTM	4.545	4.920	0.375	0.000	0.000
SLU7	4.083	4.737	0.654	0.000	0.000
SLX1A	1.389	1.502	0.113	0.000	0.000
SLX1A-SULT1	0.489	0.605	0.117	0.000	0.000
SLX1B	1.018	1.094	0.076	0.005	0.006
SLX4	1.107	1.707	0.600	0.000	0.000
SMAD1	2.710	2.795	0.085	0.062	0.067
SMAD2	2.018	2.828	0.810	0.000	0.000
SMAD3	3.452	4.130	0.678	0.000	0.000
SMAD4	3.528	3.904	0.376	0.000	0.000
SMAD5	3.460	4.313	0.853	0.000	0.000
SMAD6	2.210	1.669	-0.541	0.000	0.000
SMAD7	3.243	3.189	-0.054	0.293	0.308
SMAD9	1.183	1.028	-0.155	0.000	0.000
SMAGP	2.255	3.112	0.857	0.000	0.000
SMAP2	5.520	5.851	0.332	0.000	0.000
SMARCA1	5.280	5.705	0.425	0.000	0.000
SMARCA2	4.819	4.559	-0.260	0.000	0.000
SMARCA4	3.403	4.518	1.116	0.000	0.000
SMARCA5	3.749	4.110	0.361	0.000	0.000
SMARCAD1	2.819	3.093	0.275	0.000	0.000
SMARCAL1	2.504	3.342	0.838	0.000	0.000
SMARCB1	4.726	5.703	0.977	0.000	0.000
SMARCC1	3.471	4.503	1.032	0.000	0.000
SMARCC2	3.514	4.232	0.718	0.000	0.000
SMARCD1	3.419	4.422	1.002	0.000	0.000
SMARCD2	4.989	5.723	0.734	0.000	0.000
SMARCD3	1.762	2.363	0.601	0.000	0.000
SMARCE1	3.587	4.543	0.957	0.000	0.000
SMC1A	3.421	4.219	0.798	0.000	0.000
SMC1B	0.145	0.701	0.556	0.000	0.000
SMC2	2.007	3.038	1.031	0.000	0.000
SMC3	3.880	4.786	0.906	0.000	0.000
SMC4	2.903	4.157	1.254	0.000	0.000
SMC5	3.519	3.679	0.160	0.000	0.000
SMC6	3.237	3.818	0.581	0.000	0.000
SMCHD1	2.715	3.227	0.512	0.000	0.000
SMCR8	2.433	2.686	0.253	0.000	0.000
SMG1	3.062	3.120	0.058	0.166	0.177
SMG5	4.699	6.118	1.419	0.000	0.000
SMG6	2.396	2.603	0.207	0.000	0.000
SMG7	3.837	4.804	0.967	0.000	0.000
SMG8	2.469	3.366	0.896	0.000	0.000
SMN2	2.143	2.694	0.551	0.000	0.000
SMNDC1	3.155	3.597	0.442	0.000	0.000
SMO	3.957	5.101	1.144	0.000	0.000
SMOC1	5.817	5.340	-0.477	0.000	0.000
SMOC2	1.996	2.401	0.405	0.000	0.000
SMOX	2.043	3.098	1.056	0.000	0.000

SMPD1	5.917	5.849	-0.069	0.040	0.044
SMPD2	2.587	3.500	0.913	0.000	0.000
SMPD3	1.504	0.872	-0.632	0.000	0.000
SMPD4	3.575	4.353	0.778	0.000	0.000
SMPDL3A	5.476	5.502	0.025	0.593	0.607
SMPDL3B	0.679	1.124	0.445	0.000	0.000
SMPX	0.505	1.897	1.392	0.000	0.000
SMS	4.814	5.657	0.843	0.000	0.000
SMTN	2.953	3.391	0.438	0.000	0.000
SMTNL2	0.136	0.268	0.132	0.000	0.000
SMU1	3.826	4.344	0.518	0.000	0.000
SMUG1	3.685	4.348	0.663	0.000	0.000
SMURF1	3.330	4.149	0.819	0.000	0.000
SMURF2	2.031	2.643	0.612	0.000	0.000
SMYD2	4.104	5.308	1.204	0.000	0.000
SMYD3	1.279	2.676	1.398	0.000	0.000
SMYD4	1.871	2.199	0.328	0.000	0.000
SMYD5	3.313	4.282	0.969	0.000	0.000
SNAI1	2.445	2.158	-0.287	0.000	0.000
SNAI2	2.950	3.447	0.496	0.000	0.000
SNAI3	1.172	1.052	-0.120	0.001	0.001
SNAP25	1.338	1.910	0.572	0.000	0.000
SNAP29	4.405	4.955	0.550	0.000	0.000
SNAP47	3.280	4.207	0.927	0.000	0.000
SNAPC1	2.237	2.553	0.316	0.000	0.000
SNAPC2	2.950	3.794	0.843	0.000	0.000
SNAPC3	2.572	3.043	0.471	0.000	0.000
SNAPC4	2.999	3.284	0.284	0.000	0.000
SNAPC5	4.024	4.230	0.206	0.000	0.000
SNAPIN	4.729	5.840	1.111	0.000	0.000
SNCA	0.648	0.575	-0.073	0.030	0.033
SNCAIP	0.622	1.101	0.479	0.000	0.000
SNCG	2.567	3.758	1.190	0.000	0.000
SND1	5.688	6.634	0.946	0.000	0.000
SNED1	2.208	1.924	-0.283	0.000	0.000
SNF8	4.461	5.474	1.013	0.000	0.000
SNIP1	2.726	2.988	0.262	0.000	0.000
SNN	3.213	3.807	0.593	0.000	0.000
SNPH	0.411	0.729	0.318	0.000	0.000
SNRK	3.873	3.905	0.032	0.383	0.399
SNRNP200	4.846	5.663	0.816	0.000	0.000
SNRNP25	4.090	4.695	0.605	0.000	0.000
SNRNP27	4.086	4.670	0.585	0.000	0.000
SNRNP35	3.349	3.859	0.510	0.000	0.000
SNRNP40	3.369	3.991	0.622	0.000	0.000
SNRNP48	2.668	3.216	0.548	0.000	0.000
SNRNP70	6.410	6.884	0.474	0.000	0.000
SNRPA	4.528	5.663	1.135	0.000	0.000
SNRPA1	3.621	4.504	0.883	0.000	0.000
SNRPB	6.236	7.788	1.552	0.000	0.000
SNRPB2	4.604	5.371	0.767	0.000	0.000
SNRPC	5.949	7.269	1.320	0.000	0.000

SNRPD1	3.184	4.313	1.129	0.000	0.000
SNRPD2	6.492	7.694	1.203	0.000	0.000
SNRPD3	5.813	6.621	0.807	0.000	0.000
SNRPE	5.150	6.622	1.472	0.000	0.000
SNRPF	4.171	4.972	0.802	0.000	0.000
SNRPN	3.937	4.418	0.481	0.000	0.000
SNTA1	3.779	4.756	0.977	0.000	0.000
SNTB1	5.371	5.968	0.597	0.000	0.000
SNTB2	2.086	2.456	0.369	0.000	0.000
SNTG1	0.192	0.471	0.279	0.000	0.000
SNUPN	3.131	3.827	0.696	0.000	0.000
SNURF	2.327	2.463	0.137	0.000	0.000
SNW1	4.953	5.650	0.697	0.000	0.000
SNX1	4.654	5.121	0.467	0.000	0.000
SNX10	4.754	4.859	0.105	0.118	0.127
SNX11	3.242	4.058	0.817	0.000	0.000
SNX12	3.460	4.334	0.874	0.000	0.000
SNX13	2.117	2.703	0.585	0.000	0.000
SNX14	4.561	4.895	0.334	0.000	0.000
SNX15	1.372	2.172	0.799	0.000	0.000
SNX16	1.693	2.432	0.739	0.000	0.000
SNX17	5.503	6.228	0.725	0.000	0.000
SNX18	3.586	3.899	0.313	0.000	0.000
SNX19	3.741	4.094	0.354	0.000	0.000
SNX2	4.727	5.291	0.564	0.000	0.000
SNX20	1.030	1.061	0.031	0.465	0.481
SNX21	2.816	3.409	0.593	0.000	0.000
SNX22	3.094	4.106	1.013	0.000	0.000
SNX24	2.849	3.497	0.648	0.000	0.000
SNX25	2.710	2.874	0.164	0.000	0.000
SNX27	2.818	4.062	1.244	0.000	0.000
SNX29	1.445	1.940	0.495	0.000	0.000
SNX3	6.877	7.516	0.639	0.000	0.000
SNX30	1.871	2.460	0.588	0.000	0.000
SNX31	0.132	0.199	0.066	0.000	0.000
SNX32	0.261	0.351	0.090	0.000	0.000
SNX33	3.556	3.764	0.208	0.000	0.000
SNX4	5.194	5.528	0.334	0.000	0.000
SNX5	4.498	5.054	0.556	0.000	0.000
SNX6	4.300	4.861	0.561	0.000	0.000
SNX7	3.285	3.987	0.702	0.000	0.000
SNX8	3.064	4.107	1.043	0.000	0.000
SNX9	5.006	5.030	0.024	0.491	0.506
SOAT1	3.110	3.810	0.700	0.000	0.000
SOAT2	1.471	2.182	0.711	0.000	0.000
SOBP	1.169	1.662	0.493	0.000	0.000
SOCS1	2.789	3.019	0.231	0.002	0.002
SOCS2	4.209	2.843	-1.366	0.000	0.000
SOCS3	5.363	4.321	-1.042	0.000	0.000
SOCS4	2.243	2.754	0.511	0.000	0.000
SOCS5	2.526	3.349	0.823	0.000	0.000
SOCS6	4.062	3.974	-0.088	0.022	0.024

SOD1	9.824	9.535	-0.289	0.000	0.000
SOD2	6.115	6.441	0.326	0.000	0.000
SOD3	3.501	3.392	-0.109	0.255	0.269
SON	5.414	5.602	0.188	0.000	0.000
SORBS1	3.989	3.748	-0.241	0.000	0.000
SORBS2	4.556	4.235	-0.321	0.000	0.000
SORBS3	5.452	5.226	-0.226	0.000	0.000
SORCS2	1.637	1.405	-0.232	0.000	0.000
SORD	7.160	6.608	-0.552	0.000	0.000
SORL1	4.844	3.814	-1.031	0.000	0.000
SORT1	2.165	3.753	1.588	0.000	0.000
SOS1	2.964	3.625	0.661	0.000	0.000
SOS2	2.918	3.285	0.367	0.000	0.000
SOSTDC1	0.120	0.644	0.524	0.000	0.000
SOX12	2.513	3.770	1.257	0.000	0.000
SOX13	3.193	4.324	1.131	0.000	0.000
SOX17	1.583	1.858	0.275	0.000	0.000
SOX18	2.869	3.686	0.817	0.000	0.000
SOX2	0.094	0.591	0.498	0.000	0.000
SOX4	2.673	3.762	1.088	0.000	0.000
SOX5	1.858	1.711	-0.147	0.000	0.000
SOX6	1.567	1.163	-0.404	0.000	0.000
SOX8	0.269	0.293	0.024	0.339	0.355
SOX9	2.945	3.924	0.978	0.000	0.000
SP1	3.807	4.502	0.695	0.000	0.000
SP100	3.895	4.191	0.296	0.000	0.000
SP110	3.438	3.668	0.230	0.000	0.000
SP140	1.082	1.182	0.100	0.050	0.054
SP140L	2.953	3.328	0.376	0.000	0.000
SP2	3.058	3.778	0.720	0.000	0.000
SP3	3.512	4.192	0.681	0.000	0.000
SP4	1.382	1.867	0.485	0.000	0.000
SP5	1.514	2.867	1.353	0.000	0.000
SP6	0.212	0.740	0.529	0.000	0.000
SPA17	0.839	1.667	0.827	0.000	0.000
SPAG1	2.037	2.447	0.411	0.000	0.000
SPAG16	2.174	2.441	0.267	0.000	0.000
SPAG4	1.818	2.772	0.954	0.000	0.000
SPAG5	2.767	4.162	1.394	0.000	0.000
SPAG7	5.329	5.204	-0.125	0.000	0.000
SPAG8	0.792	1.193	0.401	0.000	0.000
SPAG9	3.761	4.103	0.342	0.000	0.000
SPARC	6.469	7.763	1.294	0.000	0.000
SPARCL1	4.212	5.567	1.355	0.000	0.000
SPAST	2.618	3.342	0.724	0.000	0.000
SPATA13	3.645	3.833	0.189	0.000	0.000
SPATA17	0.090	0.314	0.224	0.000	0.000
SPATA18	2.223	1.309	-0.914	0.000	0.000
SPATA2	2.564	3.290	0.725	0.000	0.000
SPATA20	4.590	5.001	0.411	0.000	0.000
SPATA21	0.742	1.244	0.501	0.000	0.000
SPATA24	1.943	2.728	0.785	0.000	0.000

SPATA2L	3.890	3.946	0.056	0.140	0.150
SPATA5L1	2.838	3.502	0.664	0.000	0.000
SPATA6	1.239	1.301	0.062	0.150	0.161
SPATA7	1.683	1.911	0.228	0.000	0.000
SPATA9	0.397	0.440	0.043	0.020	0.023
SPATC1	0.510	0.741	0.231	0.000	0.000
SPATS2	1.966	3.385	1.419	0.000	0.000
SPATS2L	4.971	5.579	0.608	0.000	0.000
SPC24	0.941	2.534	1.594	0.000	0.000
SPC25	0.694	2.537	1.843	0.000	0.000
SPCS1	5.625	6.436	0.811	0.000	0.000
SPCS3	4.852	5.117	0.265	0.000	0.000
SPDEF	0.247	0.516	0.269	0.000	0.000
SPDYA	0.802	1.288	0.486	0.000	0.000
SPDYC	2.070	1.869	-0.200	0.001	0.002
SPECC1	0.854	1.314	0.460	0.000	0.000
SPECC1L	3.231	3.888	0.656	0.000	0.000
SPEF1	0.197	0.363	0.166	0.000	0.000
SPEF2	0.665	1.124	0.458	0.000	0.000
SPEG	0.407	0.417	0.010	0.774	0.784
SPEN	3.520	3.792	0.271	0.000	0.000
SPESP1	0.717	1.041	0.324	0.000	0.000
SPG11	3.746	4.172	0.426	0.000	0.000
SPG21	5.648	6.210	0.562	0.000	0.000
SPG7	4.260	4.364	0.105	0.005	0.006
SPHK1	1.930	2.714	0.783	0.000	0.000
SPHK2	3.324	3.448	0.124	0.009	0.010
SPI1	4.206	3.949	-0.257	0.000	0.000
SPIB	0.725	0.686	-0.039	0.497	0.512
SPIC	1.143	0.582	-0.562	0.000	0.000
SPICE1	1.620	2.074	0.454	0.000	0.000
SPIN1	4.025	4.542	0.517	0.000	0.000
SPIN2A	0.115	0.134	0.019	0.035	0.039
SPIN2B	1.795	2.332	0.537	0.000	0.000
SPIN3	2.027	2.652	0.625	0.000	0.000
SPIN4	1.058	1.664	0.606	0.000	0.000
SPINK1	2.535	6.834	4.299	0.000	0.000
SPINK5	0.185	1.114	0.929	0.000	0.000
SPINT1	2.558	2.669	0.111	0.343	0.359
SPINT2	3.512	2.896	-0.617	0.000	0.000
SPIRE1	1.988	1.841	-0.148	0.016	0.018
SPIRE2	2.063	3.016	0.953	0.000	0.000
SPN	1.572	1.399	-0.172	0.000	0.001
SPNS1	2.407	3.444	1.037	0.000	0.000
SPNS2	3.142	2.849	-0.294	0.000	0.000
SPNS3	0.948	0.863	-0.085	0.044	0.049
SPOCD1	0.078	0.427	0.348	0.000	0.000
SPOCK1	0.229	0.983	0.754	0.000	0.000
SPOCK2	2.491	2.563	0.072	0.246	0.260
SPON2	4.935	6.371	1.436	0.000	0.000
SPOP	4.212	4.721	0.509	0.000	0.000
SPOPL	2.749	3.433	0.683	0.000	0.000

SPP1	4.757	7.141	2.385	0.000	0.000
SPP2	8.362	6.018	-2.344	0.000	0.000
SPPL2A	4.404	5.035	0.631	0.000	0.000
SPPL2B	3.709	4.261	0.552	0.000	0.000
SPPL3	3.477	4.081	0.605	0.000	0.000
SPR	6.358	6.845	0.487	0.000	0.000
SPRED1	2.324	2.490	0.166	0.001	0.002
SPRED2	3.828	3.975	0.147	0.001	0.001
SPRED3	0.132	0.210	0.078	0.000	0.000
SPRY1	3.590	3.913	0.322	0.000	0.000
SPRY2	4.245	3.589	-0.657	0.000	0.000
SPRY4	3.177	3.220	0.044	0.380	0.396
SPRYD3	3.839	4.810	0.971	0.000	0.000
SPRYD4	4.522	3.847	-0.674	0.000	0.000
SPRYD7	3.387	3.760	0.373	0.000	0.000
SPSB1	4.288	4.781	0.493	0.000	0.000
SPSB2	2.245	3.558	1.313	0.000	0.000
SPSB4	0.564	0.139	-0.425	0.000	0.000
SPTAN1	4.399	5.467	1.069	0.000	0.000
SPTB	0.567	0.700	0.133	0.000	0.000
SPTBN1	5.830	6.215	0.385	0.000	0.000
SPTBN2	4.291	3.182	-1.109	0.000	0.000
SPTBN4	0.398	0.545	0.147	0.000	0.000
SPTBN5	0.730	1.101	0.371	0.000	0.000
SPTLC1	3.724	4.359	0.634	0.000	0.000
SPTLC2	2.567	3.110	0.543	0.000	0.000
SPTLC3	2.762	2.896	0.134	0.014	0.016
SPTY2D1	2.957	3.421	0.464	0.000	0.000
SQLE	3.760	5.814	2.054	0.000	0.000
SQSTM1	7.085	8.427	1.342	0.000	0.000
SRA1	4.657	5.343	0.686	0.000	0.000
SRBD1	2.600	2.983	0.384	0.000	0.000
SRC	2.814	3.620	0.807	0.000	0.000
SRCAP	3.194	3.763	0.570	0.000	0.000
SRD5A1	4.537	3.776	-0.761	0.000	0.000
SRD5A3	2.780	3.715	0.935	0.000	0.000
SREBF1	5.636	5.756	0.121	0.042	0.046
SREBF2	4.478	5.553	1.076	0.000	0.000
SREK1	3.348	3.794	0.446	0.000	0.000
SREK1IP1	2.783	3.313	0.530	0.000	0.000
SRF	4.112	4.550	0.438	0.000	0.000
SRFBP1	2.231	2.924	0.693	0.000	0.000
SRGAP1	0.628	0.979	0.352	0.000	0.000
SRGAP3	0.546	0.864	0.318	0.000	0.000
SRGN	6.535	5.985	-0.550	0.000	0.000
SRI	3.577	4.494	0.917	0.000	0.000
SRL	0.298	0.297	0.000	0.977	0.978
SRM	5.077	6.158	1.080	0.000	0.000
SRMS	0.278	0.374	0.095	0.000	0.000
SRP14	7.242	8.075	0.833	0.000	0.000
SRP19	4.052	4.797	0.745	0.000	0.000
SRP54	4.939	5.361	0.422	0.000	0.000

SRP68	4.350	5.127	0.776	0.000	0.000
SRP72	4.887	5.557	0.670	0.000	0.000
SRP9	7.395	8.299	0.904	0.000	0.000
SRPK1	3.408	4.433	1.025	0.000	0.000
SRPK2	3.002	3.840	0.838	0.000	0.000
SRPK3	0.262	0.348	0.086	0.000	0.000
SRPRB	5.029	5.914	0.885	0.000	0.000
SRPX	3.818	1.781	-2.037	0.000	0.000
SRPX2	2.146	2.186	0.040	0.541	0.555
SRR	2.605	2.891	0.286	0.000	0.000
SRRM1	4.237	4.438	0.201	0.000	0.000
SRRM2	6.770	6.742	-0.029	0.602	0.616
SRRM3	0.229	0.710	0.482	0.000	0.000
SRRM5	1.133	1.557	0.424	0.000	0.000
SRRT	5.177	5.779	0.602	0.000	0.000
SRSF1	6.043	6.753	0.710	0.000	0.000
SRSF10	3.524	3.849	0.325	0.000	0.000
SRSF11	5.397	5.676	0.279	0.000	0.000
SRSF12	0.462	0.822	0.360	0.000	0.000
SRSF2	6.280	6.882	0.603	0.000	0.000
SRSF3	5.520	6.083	0.563	0.000	0.000
SRSF4	5.703	5.862	0.158	0.000	0.000
SRSF5	6.791	6.776	-0.015	0.673	0.685
SRSF6	6.296	6.508	0.212	0.000	0.000
SRSF7	5.634	5.984	0.350	0.000	0.000
SRSF9	5.294	5.982	0.688	0.000	0.000
SRXN1	2.440	4.128	1.687	0.000	0.000
SS18	4.463	4.737	0.274	0.000	0.000
SS18L1	4.294	4.473	0.180	0.000	0.000
SS18L2	4.665	5.189	0.524	0.000	0.000
SSB	4.710	5.530	0.820	0.000	0.000
SSBP1	4.754	5.660	0.905	0.000	0.000
SSBP2	1.119	1.375	0.256	0.000	0.000
SSBP3	4.427	4.584	0.158	0.000	0.000
SSBP4	3.917	4.697	0.780	0.000	0.000
SSC5D	1.316	0.860	-0.456	0.000	0.000
SSH1	2.346	2.907	0.561	0.000	0.000
SSH2	2.074	2.536	0.462	0.000	0.000
SSH3	3.512	4.169	0.658	0.000	0.000
SSNA1	5.587	6.491	0.904	0.000	0.000
SSPN	0.867	0.898	0.031	0.520	0.535
SSPO	0.582	0.772	0.190	0.000	0.000
SSR1	4.604	5.717	1.113	0.000	0.000
SSR2	5.589	7.081	1.492	0.000	0.000
SSR3	5.746	6.719	0.972	0.000	0.000
SSR4	6.476	7.072	0.597	0.000	0.000
SSRP1	4.411	5.422	1.011	0.000	0.000
SSSCA1	4.085	4.860	0.775	0.000	0.000
SSTR1	2.424	2.176	-0.248	0.001	0.002
SSTR2	1.080	1.056	-0.025	0.651	0.664
SSU72	5.043	5.526	0.483	0.000	0.000
SSX1	0.225	2.082	1.856	0.000	0.000

SSX2IP	2.213	3.088	0.875	0.000	0.000
ST13	6.916	7.284	0.368	0.000	0.000
ST14	3.424	4.095	0.671	0.000	0.000
ST20	2.714	3.303	0.589	0.000	0.000
ST20-MTHFS	3.026	3.127	0.101	0.003	0.003
ST3GAL1	5.029	5.197	0.168	0.007	0.007
ST3GAL2	2.376	3.085	0.709	0.000	0.000
ST3GAL3	2.713	3.161	0.447	0.000	0.000
ST3GAL4	3.380	3.771	0.391	0.000	0.000
ST3GAL5	2.966	3.111	0.145	0.000	0.001
ST3GAL6	4.541	3.641	-0.899	0.000	0.000
ST5	2.889	3.514	0.625	0.000	0.000
ST6GAL1	7.542	7.405	-0.137	0.009	0.010
ST6GAL2	1.366	0.481	-0.885	0.000	0.000
ST6GALNAC1	0.477	0.294	-0.183	0.000	0.000
ST6GALNAC2	0.737	1.348	0.611	0.000	0.000
ST6GALNAC3	0.897	0.780	-0.118	0.000	0.000
ST6GALNAC4	2.137	2.907	0.771	0.000	0.000
ST6GALNAC6	4.800	4.650	-0.150	0.000	0.000
ST7	3.731	4.012	0.281	0.000	0.000
ST7L	1.741	2.247	0.506	0.000	0.000
ST8SIA1	0.271	0.295	0.024	0.243	0.257
ST8SIA4	1.411	1.504	0.093	0.040	0.045
ST8SIA6	0.439	0.198	-0.242	0.000	0.000
STAB1	5.130	4.237	-0.893	0.000	0.000
STAB2	3.215	0.637	-2.578	0.000	0.000
STAC	0.530	0.560	0.030	0.489	0.505
STAC3	2.186	2.381	0.196	0.000	0.000
STAG1	2.489	3.102	0.612	0.000	0.000
STAG2	3.831	4.515	0.684	0.000	0.000
STAG3	1.613	1.795	0.182	0.000	0.000
STAM	2.850	3.652	0.802	0.000	0.000
STAM2	2.992	3.512	0.520	0.000	0.000
STAMBP	3.143	3.914	0.771	0.000	0.000
STAMBPL1	1.195	1.990	0.795	0.000	0.000
STAP1	0.820	0.599	-0.221	0.000	0.000
STAP2	5.617	6.030	0.413	0.000	0.000
STAR	0.170	0.281	0.111	0.000	0.001
STARD10	7.671	7.698	0.027	0.591	0.605
STARD13	2.441	2.538	0.097	0.016	0.017
STARD3	4.155	4.965	0.811	0.000	0.000
STARD3NL	2.879	3.589	0.710	0.000	0.000
STARD4	3.332	3.332	0.000	0.994	0.994
STARD5	3.290	2.400	-0.890	0.000	0.000
STARD7	5.518	6.246	0.729	0.000	0.000
STARD8	2.496	2.850	0.355	0.000	0.000
STARD9	0.720	1.026	0.306	0.000	0.000
STAT1	5.908	6.490	0.582	0.000	0.000
STAT2	5.653	5.836	0.183	0.000	0.000
STAT3	5.730	5.712	-0.018	0.681	0.693
STAT4	2.602	2.170	-0.432	0.000	0.000
STAT5A	2.809	3.205	0.396	0.000	0.000

STAT5B	4.194	4.653	0.459	0.000	0.000
STAT6	5.636	5.872	0.237	0.000	0.000
STAU1	6.409	6.828	0.419	0.000	0.000
STAU2	2.986	3.750	0.764	0.000	0.000
STC1	1.885	3.031	1.146	0.000	0.000
STC2	0.761	2.041	1.281	0.000	0.000
STEAP1	3.452	4.326	0.874	0.000	0.000
STEAP1B	0.076	0.364	0.288	0.000	0.000
STEAP2	1.069	1.890	0.822	0.000	0.000
STEAP3	6.661	5.289	-1.372	0.000	0.000
STEAP4	3.230	1.762	-1.468	0.000	0.000
STIL	0.586	1.690	1.104	0.000	0.000
STIM1	4.296	5.046	0.750	0.000	0.000
STIM2	2.807	3.288	0.481	0.000	0.000
STIP1	4.730	6.129	1.398	0.000	0.000
STK10	2.870	3.060	0.189	0.000	0.000
STK11	3.838	4.422	0.584	0.000	0.000
STK11IP	2.290	2.960	0.670	0.000	0.000
STK16	4.119	4.524	0.405	0.000	0.000
STK17A	3.038	3.673	0.635	0.000	0.000
STK17B	2.401	2.450	0.050	0.398	0.414
STK19	3.036	3.669	0.633	0.000	0.000
STK24	3.217	4.017	0.800	0.000	0.000
STK25	4.374	5.243	0.869	0.000	0.000
STK3	2.367	3.052	0.685	0.000	0.000
STK31	0.289	0.379	0.091	0.000	0.001
STK32B	0.241	0.389	0.149	0.000	0.000
STK32C	1.639	1.934	0.295	0.000	0.000
STK33	0.342	0.365	0.024	0.455	0.471
STK35	2.583	3.397	0.814	0.000	0.000
STK36	2.551	3.355	0.804	0.000	0.000
STK38	4.243	4.896	0.653	0.000	0.000
STK38L	2.899	3.025	0.126	0.004	0.004
STK39	1.529	2.906	1.376	0.000	0.000
STK4	2.810	3.338	0.528	0.000	0.000
STK40	4.580	4.486	-0.094	0.010	0.011
STMN1	3.221	5.466	2.245	0.000	0.000
STMN2	1.254	1.224	-0.030	0.734	0.745
STMN3	1.955	2.327	0.373	0.000	0.000
STOM	7.908	7.489	-0.419	0.000	0.000
STOML1	1.876	2.739	0.863	0.000	0.000
STOML2	6.206	6.963	0.757	0.000	0.000
STON1	1.019	1.064	0.045	0.267	0.282
STON2	1.106	1.219	0.112	0.002	0.002
STOX1	0.385	0.894	0.509	0.000	0.000
STOX2	0.863	1.030	0.167	0.000	0.000
STRA8	0.187	0.456	0.269	0.000	0.000
STRADA	2.155	2.817	0.661	0.000	0.000
STRADB	5.403	5.391	-0.011	0.740	0.750
STRAP	5.987	6.565	0.578	0.000	0.000
STRBP	2.275	3.193	0.919	0.000	0.000
STRC	0.233	0.337	0.104	0.000	0.000

STRN	2.554	2.966	0.413	0.000	0.000
STRN3	3.824	3.965	0.140	0.000	0.000
STRN4	3.565	4.555	0.989	0.000	0.000
STS	2.698	2.723	0.026	0.630	0.643
STT3A	4.905	6.001	1.097	0.000	0.000
STT3B	5.077	5.958	0.881	0.000	0.000
STX10	4.794	5.783	0.989	0.000	0.000
STX11	2.669	2.210	-0.458	0.000	0.000
STX12	4.249	4.514	0.265	0.000	0.000
STX16	4.512	5.188	0.676	0.000	0.000
STX16-NPEPL	2.043	2.442	0.399	0.000	0.000
STX17	3.459	3.596	0.137	0.000	0.000
STX18	3.496	4.027	0.532	0.000	0.000
STX19	0.325	0.287	-0.038	0.096	0.103
STX1A	0.901	1.795	0.894	0.000	0.000
STX1B	1.674	1.630	-0.044	0.314	0.330
STX2	3.166	3.541	0.375	0.000	0.000
STX3	2.968	3.638	0.669	0.000	0.000
STX4	4.461	5.185	0.724	0.000	0.000
STX5	5.076	5.622	0.546	0.000	0.000
STX6	2.304	3.392	1.089	0.000	0.000
STX7	2.501	3.070	0.569	0.000	0.000
STX8	3.647	3.874	0.227	0.000	0.000
STXBP1	1.619	2.173	0.555	0.000	0.000
STXBP2	3.909	4.275	0.367	0.000	0.000
STXBP3	3.723	4.178	0.455	0.000	0.000
STXBP4	0.547	1.149	0.602	0.000	0.000
STXBP5	1.364	1.920	0.556	0.000	0.000
STXBP6	1.063	2.366	1.303	0.000	0.000
STYK1	0.258	0.252	-0.007	0.810	0.818
STYX	3.913	4.181	0.269	0.000	0.000
STYXL1	4.294	5.061	0.767	0.000	0.000
SUB1	5.098	6.181	1.083	0.000	0.000
SUCLA2	3.853	4.107	0.254	0.000	0.000
SUCLG1	6.102	6.380	0.278	0.000	0.000
SUCLG2	7.198	6.615	-0.583	0.000	0.000
SUCNR1	1.631	1.292	-0.339	0.000	0.000
SUDS3	3.551	4.090	0.539	0.000	0.000
SUFU	2.161	2.670	0.509	0.000	0.000
SUGP1	3.380	4.137	0.756	0.000	0.000
SUGP2	3.513	4.189	0.675	0.000	0.000
SUGT1	2.895	3.310	0.415	0.000	0.000
SULF1	1.277	2.214	0.937	0.000	0.000
SULF2	4.931	4.005	-0.927	0.000	0.000
SULT1A1	5.842	4.769	-1.073	0.000	0.000
SULT1A2	4.677	3.744	-0.933	0.000	0.000
SULT1B1	2.363	1.778	-0.585	0.000	0.000
SULT1C2	0.587	2.741	2.155	0.000	0.000
SULT1C4	1.339	1.350	0.011	0.874	0.879
SULT1E1	4.109	2.718	-1.391	0.000	0.000
SULT2A1	9.858	8.438	-1.419	0.000	0.000
SULT2B1	0.301	0.531	0.230	0.000	0.000

SULT4A1	0.288	0.887	0.599	0.000	0.000
SUMF1	3.449	4.093	0.645	0.000	0.000
SUMF2	5.690	6.604	0.914	0.000	0.000
SUMO1	6.527	6.959	0.432	0.000	0.000
SUMO2	5.591	6.586	0.995	0.000	0.000
SUMO3	5.200	6.010	0.810	0.000	0.000
SUMO4	0.813	0.669	-0.144	0.000	0.000
SUN1	3.977	4.719	0.742	0.000	0.000
SUN2	6.085	5.860	-0.225	0.000	0.000
SUOX	4.894	4.749	-0.144	0.000	0.000
SUPT16H	3.980	4.940	0.960	0.000	0.000
SUPT3H	1.034	1.697	0.663	0.000	0.000
SUPT4H1	5.628	6.545	0.917	0.000	0.000
SUPT5H	4.938	5.625	0.687	0.000	0.000
SUPT6H	4.401	4.911	0.510	0.000	0.000
SUPT7L	3.951	4.556	0.605	0.000	0.000
SUPV3L1	4.114	4.674	0.560	0.000	0.000
SURF1	6.149	6.362	0.212	0.000	0.000
SURF2	4.539	5.009	0.470	0.000	0.000
SURF4	7.190	7.758	0.568	0.000	0.000
SURF6	3.661	4.347	0.686	0.000	0.000
SUSD1	1.676	2.194	0.519	0.000	0.000
SUSD2	1.727	1.989	0.261	0.000	0.000
SUSD3	4.015	3.880	-0.135	0.108	0.117
SUSD4	1.387	2.410	1.024	0.000	0.000
SUSD5	0.329	0.391	0.062	0.050	0.055
SUV39H1	2.430	3.599	1.169	0.000	0.000
SUV39H2	1.754	2.798	1.044	0.000	0.000
SUZ12	3.141	4.070	0.929	0.000	0.000
SV2A	0.653	0.870	0.217	0.000	0.000
SV2B	0.301	0.473	0.172	0.000	0.000
SVEP1	1.769	1.007	-0.762	0.000	0.000
SVIL	2.704	3.433	0.729	0.000	0.000
SVIP	2.813	3.441	0.628	0.000	0.000
SWAP70	2.660	3.602	0.942	0.000	0.000
SWT1	1.750	2.468	0.718	0.000	0.000
SYAP1	4.611	4.783	0.172	0.000	0.000
SYBU	4.103	4.116	0.013	0.849	0.855
SYCE1	0.367	0.399	0.031	0.535	0.549
SYCE1L	1.216	1.280	0.064	0.149	0.159
SYCE2	1.107	1.485	0.378	0.000	0.000
SYCP2	0.581	0.647	0.067	0.045	0.049
SYCP2L	0.208	0.318	0.109	0.000	0.000
SYCP3	1.086	1.418	0.332	0.000	0.000
SYDE1	2.175	2.375	0.199	0.000	0.000
SYDE2	1.329	1.097	-0.232	0.000	0.000
SYF2	4.719	5.301	0.582	0.000	0.000
SYK	2.243	2.255	0.012	0.849	0.855
SYMPK	4.267	4.953	0.686	0.000	0.000
SYN1	0.946	0.873	-0.073	0.116	0.125
SYN3	0.072	0.510	0.438	0.000	0.000
SYNCRIP	4.269	4.905	0.636	0.000	0.000

SYNE1	2.602	1.954	-0.649	0.000	0.000
SYNE2	2.738	2.819	0.081	0.068	0.074
SYNGAP1	1.136	1.512	0.376	0.000	0.000
SYNGR1	1.614	2.259	0.645	0.000	0.000
SYNGR2	5.308	6.342	1.034	0.000	0.000
SYNGR3	0.281	0.515	0.234	0.000	0.000
SYNGR4	0.174	0.429	0.255	0.000	0.000
SYNJ1	2.065	2.310	0.245	0.000	0.000
SYNJ2	2.092	3.007	0.916	0.000	0.000
SYNJ2BP	3.548	3.776	0.228	0.000	0.000
SYNJ2BP-COX	2.334	2.605	0.272	0.000	0.000
SYNM	1.814	2.478	0.664	0.000	0.000
SYNPO	4.428	4.676	0.248	0.000	0.000
SYNPO2	1.670	1.365	-0.305	0.000	0.000
SYP	0.471	1.050	0.580	0.000	0.000
SYPL1	5.459	6.493	1.034	0.000	0.000
SYPL2	1.812	2.153	0.341	0.000	0.000
SYS1	3.825	4.372	0.547	0.000	0.000
SYS1-DBNDD	1.521	1.862	0.341	0.000	0.000
SYT1	0.891	1.213	0.322	0.000	0.000
SYT11	1.395	1.755	0.360	0.000	0.000
SYT12	2.345	2.542	0.197	0.005	0.006
SYT13	0.855	0.782	-0.074	0.325	0.341
SYT15	0.643	0.883	0.240	0.000	0.000
SYT17	2.337	2.439	0.102	0.072	0.078
SYT2	0.225	0.293	0.068	0.001	0.001
SYT3	0.155	0.538	0.383	0.000	0.000
SYT7	4.556	3.980	-0.577	0.000	0.000
SYT8	0.610	0.850	0.240	0.001	0.001
SYT9	1.429	0.349	-1.080	0.000	0.000
SYTL1	1.589	1.501	-0.088	0.095	0.103
SYTL2	1.751	1.626	-0.125	0.006	0.007
SYTL3	1.739	1.303	-0.435	0.000	0.000
SYTL4	3.094	3.198	0.104	0.057	0.063
SYTL5	1.619	1.005	-0.614	0.000	0.000
SYVN1	5.723	6.116	0.393	0.000	0.000
SZT2	2.772	3.143	0.372	0.000	0.000
TAB1	3.360	4.174	0.814	0.000	0.000
TAB2	4.723	4.751	0.028	0.497	0.512
TAB3	3.288	3.742	0.454	0.000	0.000
TAC3	0.150	0.427	0.277	0.000	0.000
TACC1	3.584	4.139	0.555	0.000	0.000
TACC2	1.955	2.881	0.926	0.000	0.000
TACC3	1.924	3.570	1.646	0.000	0.000
TACO1	5.385	5.807	0.422	0.000	0.000
TACR2	0.233	0.369	0.136	0.000	0.000
TACSTD2	3.039	1.395	-1.644	0.000	0.000
TADA1	3.977	4.687	0.709	0.000	0.000
TADA2B	3.351	3.748	0.397	0.000	0.000
TADA3	4.796	5.374	0.578	0.000	0.000
TAF1	2.455	3.016	0.561	0.000	0.000
TAF10	4.276	5.220	0.944	0.000	0.000

TAF11	4.787	5.685	0.898	0.000	0.000
TAF12	3.728	4.398	0.669	0.000	0.000
TAF13	4.447	4.902	0.455	0.000	0.000
TAF1A	1.272	2.228	0.956	0.000	0.000
TAF1B	1.876	2.625	0.749	0.000	0.000
TAF1C	3.713	4.021	0.308	0.000	0.000
TAF1D	3.442	4.138	0.696	0.000	0.000
TAF2	2.676	3.609	0.933	0.000	0.000
TAF3	2.179	2.566	0.387	0.000	0.000
TAF4	2.097	2.865	0.768	0.000	0.000
TAF4B	0.538	0.580	0.042	0.221	0.234
TAF5	1.790	2.450	0.659	0.000	0.000
TAF5L	2.865	3.625	0.760	0.000	0.000
TAF6	2.970	4.376	1.406	0.000	0.000
TAF6L	3.855	4.220	0.366	0.000	0.000
TAF7	5.601	6.195	0.595	0.000	0.000
TAF7L	0.561	0.534	-0.028	0.349	0.365
TAF8	2.404	2.972	0.568	0.000	0.000
TAF9	5.003	6.083	1.080	0.000	0.000
TAF9B	3.297	4.070	0.773	0.000	0.000
TAGAP	1.930	1.520	-0.410	0.000	0.000
TAGLN	5.632	5.609	-0.022	0.768	0.778
TAGLN2	6.991	8.347	1.356	0.000	0.000
TAL1	1.216	1.072	-0.145	0.000	0.000
TAL2	0.282	0.707	0.425	0.000	0.000
TALDO1	6.440	7.411	0.971	0.000	0.000
TANC1	2.161	2.293	0.131	0.003	0.003
TANC2	0.548	0.796	0.247	0.000	0.000
TANK	4.364	4.436	0.072	0.039	0.043
TAOK1	2.458	3.143	0.685	0.000	0.000
TAOK2	3.148	4.057	0.909	0.000	0.000
TAOK3	2.969	3.654	0.685	0.000	0.000
TAP1	4.591	5.497	0.906	0.000	0.000
TAP2	3.205	4.060	0.855	0.000	0.000
TAPBP	6.181	6.967	0.785	0.000	0.000
TAPBPL	4.485	4.694	0.210	0.000	0.000
TAPT1	4.113	3.826	-0.287	0.000	0.000
TARBP1	2.715	4.313	1.598	0.000	0.000
TARBP2	3.450	4.110	0.660	0.000	0.000
TARDBP	6.212	6.802	0.590	0.000	0.000
TARS	5.128	5.633	0.505	0.000	0.000
TARS2	4.226	5.016	0.790	0.000	0.000
TARSL2	2.341	2.564	0.224	0.000	0.000
TAS1R3	0.583	0.536	-0.047	0.172	0.184
TAS2R10	0.247	0.342	0.095	0.000	0.000
TAS2R14	0.784	0.976	0.192	0.000	0.000
TAS2R19	0.281	0.355	0.074	0.000	0.000
TAS2R20	0.878	1.163	0.284	0.000	0.000
TAS2R31	0.210	0.258	0.048	0.003	0.004
TAS2R4	0.234	0.396	0.162	0.000	0.000
TAS2R5	0.569	0.865	0.296	0.000	0.000
TASP1	2.104	2.719	0.614	0.000	0.000

TAT	9.031	6.635	-2.396	0.000	0.000
TATDN1	2.789	3.920	1.131	0.000	0.000
TATDN2	2.987	3.880	0.893	0.000	0.000
TATDN3	2.863	3.774	0.911	0.000	0.000
TAX1BP1	4.610	5.599	0.990	0.000	0.000
TAX1BP3	3.678	4.638	0.959	0.000	0.000
TAZ	3.443	4.424	0.982	0.000	0.000
TBC1D1	3.631	4.100	0.470	0.000	0.000
TBC1D10A	2.655	2.819	0.164	0.000	0.000
TBC1D10B	3.031	3.706	0.675	0.000	0.000
TBC1D10C	2.305	2.225	-0.080	0.197	0.209
TBC1D12	2.113	2.621	0.507	0.000	0.000
TBC1D13	3.339	4.343	1.004	0.000	0.000
TBC1D15	3.823	3.953	0.130	0.000	0.000
TBC1D16	1.837	2.997	1.160	0.000	0.000
TBC1D17	4.498	5.118	0.621	0.000	0.000
TBC1D19	1.205	1.716	0.511	0.000	0.000
TBC1D2	3.147	3.507	0.360	0.000	0.000
TBC1D20	3.530	4.316	0.786	0.000	0.000
TBC1D22A	3.266	4.094	0.827	0.000	0.000
TBC1D22B	2.397	3.143	0.746	0.000	0.000
TBC1D23	3.947	4.213	0.266	0.000	0.000
TBC1D24	2.648	3.091	0.443	0.000	0.000
TBC1D25	2.783	3.316	0.533	0.000	0.000
TBC1D2B	3.876	3.914	0.038	0.430	0.446
TBC1D30	0.824	1.287	0.462	0.000	0.000
TBC1D4	3.138	3.284	0.146	0.009	0.010
TBC1D5	2.641	3.349	0.708	0.000	0.000
TBC1D7	2.118	3.183	1.065	0.000	0.000
TBC1D8	4.028	4.362	0.333	0.000	0.000
TBC1D8B	1.964	2.268	0.304	0.000	0.000
TBC1D9	3.555	3.610	0.055	0.268	0.283
TBC1D9B	4.415	5.067	0.652	0.000	0.000
TBCA	6.298	6.907	0.609	0.000	0.000
TBCB	4.321	5.338	1.017	0.000	0.000
TBCC	3.806	4.827	1.020	0.000	0.000
TBCCD1	2.735	3.337	0.602	0.000	0.000
TBCD	3.756	4.489	0.733	0.000	0.000
TBCE	3.809	5.285	1.476	0.000	0.000
TBCEL	2.862	3.160	0.297	0.000	0.000
TBCK	2.141	2.470	0.329	0.000	0.000
TBK1	3.596	4.107	0.511	0.000	0.000
TBKBP1	1.797	2.482	0.685	0.000	0.000
TBL1X	3.437	3.873	0.435	0.000	0.000
TBL1XR1	3.656	4.413	0.756	0.000	0.000
TBL1Y	0.332	0.363	0.031	0.338	0.353
TBL2	3.479	4.419	0.941	0.000	0.000
TBL3	3.612	4.174	0.563	0.000	0.000
TBP	3.235	3.835	0.600	0.000	0.000
TBPL1	2.719	3.252	0.534	0.000	0.000
TBRG4	4.180	5.024	0.844	0.000	0.000
TBX1	0.440	0.407	-0.033	0.314	0.329

TBX10	0.645	0.998	0.353	0.000	0.000
TBX15	4.024	2.777	-1.247	0.000	0.000
TBX19	1.119	1.712	0.593	0.000	0.000
TBX2	2.159	2.729	0.570	0.000	0.000
TBX21	1.348	1.108	-0.240	0.000	0.000
TBX3	3.925	4.275	0.350	0.000	0.000
TBX6	0.611	1.005	0.394	0.000	0.000
TBXA2R	2.603	1.568	-1.035	0.000	0.000
TBXAS1	2.371	2.142	-0.229	0.000	0.000
TC2N	2.155	2.231	0.077	0.340	0.356
TCAP	0.888	1.318	0.430	0.000	0.000
TCEA1	4.857	5.809	0.952	0.000	0.000
TCEA2	4.362	4.518	0.156	0.005	0.005
TCEA3	6.422	6.520	0.098	0.031	0.035
TCEAL1	3.956	4.633	0.677	0.000	0.000
TCEAL3	2.646	2.737	0.090	0.109	0.117
TCEAL4	5.796	6.220	0.424	0.000	0.000
TCEAL7	1.340	1.061	-0.278	0.000	0.000
TCEAL8	5.081	5.572	0.492	0.000	0.000
TCEANC	1.152	1.375	0.223	0.000	0.000
TCEANC2	0.881	1.405	0.525	0.000	0.000
TCERG1	3.185	4.019	0.834	0.000	0.000
TCF12	3.077	3.677	0.601	0.000	0.000
TCF15	0.415	0.939	0.523	0.000	0.000
TCF19	1.536	3.662	2.125	0.000	0.000
TCF20	2.856	3.779	0.923	0.000	0.000
TCF21	1.397	0.628	-0.769	0.000	0.000
TCF25	5.111	5.224	0.113	0.000	0.000
TCF3	3.125	4.185	1.061	0.000	0.000
TCF4	1.829	2.081	0.252	0.000	0.000
TCF7	2.037	2.507	0.470	0.000	0.000
TCF7L1	3.035	3.437	0.402	0.000	0.000
TCF7L2	3.819	3.942	0.123	0.001	0.001
TCFL5	2.248	3.138	0.890	0.000	0.000
TCHH	0.345	0.213	-0.132	0.000	0.000
TCHP	1.906	2.818	0.913	0.000	0.000
TCIRG1	5.180	5.918	0.738	0.000	0.000
TCL1A	0.768	0.438	-0.330	0.000	0.000
TCN1	0.506	0.402	-0.104	0.087	0.095
TCN2	3.623	3.926	0.303	0.000	0.000
TCOF1	2.421	3.547	1.126	0.000	0.000
TCP1	5.687	6.403	0.716	0.000	0.000
TCP10L	2.503	2.053	-0.450	0.000	0.000
TCP11	0.101	0.163	0.062	0.000	0.000
TCP11L1	1.567	2.110	0.543	0.000	0.000
TCP11L2	1.177	1.394	0.217	0.000	0.000
TCTA	4.746	5.510	0.764	0.000	0.000
TCTE1	0.122	0.137	0.016	0.248	0.262
TCTE3	0.509	0.652	0.143	0.000	0.000
TCTEX1D1	1.998	0.919	-1.079	0.000	0.000
TCTEX1D2	1.363	2.202	0.839	0.000	0.000
TCTN1	2.408	2.764	0.355	0.000	0.000

TCTN2	1.113	1.606	0.493	0.000	0.000
TCTN3	4.121	4.712	0.591	0.000	0.000
TDG	2.986	3.694	0.708	0.000	0.000
TDGF1	0.708	1.921	1.214	0.000	0.000
TDO2	8.198	5.960	-2.238	0.000	0.000
TDP1	1.797	2.569	0.772	0.000	0.000
TDP2	4.829	5.643	0.814	0.000	0.000
TDRD10	2.076	2.268	0.192	0.004	0.005
TDRD3	2.380	2.841	0.461	0.000	0.000
TDRD6	0.966	0.906	-0.060	0.078	0.085
TDRD7	2.883	3.566	0.683	0.000	0.000
TDRD9	0.362	0.397	0.035	0.365	0.380
TDRKH	1.441	2.866	1.425	0.000	0.000
TEAD1	3.233	3.726	0.493	0.000	0.000
TEAD2	2.732	3.890	1.158	0.000	0.000
TEAD3	3.529	4.358	0.829	0.000	0.000
TEAD4	1.786	2.409	0.624	0.000	0.000
TEC	1.798	1.620	-0.179	0.000	0.000
TECPR1	2.333	3.128	0.795	0.000	0.000
TECPR2	1.861	2.329	0.468	0.000	0.000
TECR	5.312	5.864	0.553	0.000	0.000
TECTA	0.627	0.464	-0.163	0.000	0.000
TECTB	0.402	0.470	0.067	0.053	0.058
TEF	3.431	4.106	0.674	0.000	0.000
TEK	2.640	1.966	-0.674	0.000	0.000
TEKT2	0.368	0.254	-0.114	0.000	0.000
TEKT3	0.198	0.198	0.000	0.976	0.977
TEKT5	0.465	0.745	0.280	0.000	0.000
TELO2	4.073	4.594	0.521	0.000	0.000
TEN1	2.916	3.181	0.265	0.000	0.000
TEP1	2.522	2.898	0.376	0.000	0.000
TERF1	2.509	3.349	0.839	0.000	0.000
TERF2	3.085	3.606	0.522	0.000	0.000
TERF2IP	5.146	5.189	0.043	0.157	0.168
TERT	0.035	1.238	1.203	0.000	0.000
TES	2.982	3.872	0.890	0.000	0.000
TESC	2.623	3.476	0.853	0.000	0.000
TESK1	3.812	4.493	0.681	0.000	0.000
TESK2	2.623	2.555	-0.068	0.069	0.075
TET1	0.305	0.711	0.406	0.000	0.000
TET2	1.506	1.561	0.055	0.090	0.097
TEX10	2.202	2.773	0.571	0.000	0.000
TEX11	0.158	0.634	0.476	0.000	0.000
TEX12	0.261	0.226	-0.035	0.006	0.007
TEX14	0.382	0.449	0.067	0.001	0.001
TEX19	0.085	0.346	0.261	0.000	0.000
TEX2	3.853	4.249	0.396	0.000	0.000
TEX22	0.209	0.534	0.325	0.000	0.000
TEX261	4.620	5.389	0.769	0.000	0.000
TEX264	4.737	5.260	0.524	0.000	0.000
TEX9	0.355	0.517	0.162	0.000	0.000
TF	11.865	10.882	-0.983	0.000	0.000

TFAM	3.509	3.883	0.374	0.000	0.000
TFAP2A	0.166	0.645	0.480	0.000	0.000
TFAP2E	0.421	0.725	0.304	0.000	0.000
TFAP4	1.449	2.211	0.762	0.000	0.000
TFB1M	3.298	3.542	0.244	0.000	0.000
TFB2M	4.714	5.421	0.707	0.000	0.000
TFCP2	3.207	3.901	0.694	0.000	0.000
TFCP2L1	0.333	0.475	0.142	0.000	0.000
TFDP1	4.269	4.795	0.526	0.000	0.000
TFDP2	2.559	3.112	0.554	0.000	0.000
TFE3	4.167	4.895	0.727	0.000	0.000
TFEB	2.616	2.896	0.279	0.000	0.000
TFEC	1.221	1.247	0.026	0.578	0.592
TFF2	1.262	0.964	-0.299	0.004	0.005
TFF3	2.533	2.736	0.203	0.026	0.028
TFG	5.501	6.426	0.925	0.000	0.000
TFIP11	3.784	4.532	0.748	0.000	0.000
TFPI	5.738	5.791	0.053	0.413	0.430
TFPI2	2.716	1.379	-1.337	0.000	0.000
TFPT	3.883	4.671	0.787	0.000	0.000
TFR2	8.621	7.946	-0.675	0.000	0.000
TFRC	3.826	5.068	1.243	0.000	0.000
TG	0.246	0.395	0.148	0.000	0.000
TGDS	4.197	4.035	-0.162	0.000	0.000
TGFA	1.933	1.520	-0.413	0.000	0.000
TGFB1	3.968	4.325	0.357	0.000	0.000
TGFB1I1	2.129	2.322	0.193	0.000	0.000
TGFB2	1.069	1.613	0.544	0.000	0.000
TGFB3	2.710	2.563	-0.147	0.021	0.023
TGFBI	6.210	6.696	0.486	0.000	0.000
TGFBR1	3.108	3.741	0.633	0.000	0.000
TGFBR2	5.182	5.509	0.327	0.000	0.000
TGFBR3	3.350	2.895	-0.455	0.000	0.000
TGFBRAP1	1.983	2.469	0.486	0.000	0.000
TGIF1	3.677	4.268	0.591	0.000	0.000
TGIF2	3.099	3.816	0.718	0.000	0.000
TGM1	0.570	0.703	0.134	0.000	0.000
TGM2	6.355	6.269	-0.086	0.207	0.220
TGM3	0.492	2.797	2.305	0.000	0.000
TGOLN2	6.112	6.604	0.492	0.000	0.000
TGS1	2.751	3.561	0.810	0.000	0.000
THADA	2.393	3.045	0.652	0.000	0.000
THAP1	2.801	3.204	0.403	0.000	0.000
THAP10	1.215	1.642	0.427	0.000	0.000
THAP11	3.893	4.480	0.587	0.000	0.000
THAP2	1.231	1.698	0.467	0.000	0.000
THAP3	3.238	3.751	0.513	0.000	0.000
THAP4	4.678	4.901	0.223	0.000	0.000
THAP5	3.529	3.929	0.400	0.000	0.000
THAP6	2.224	2.433	0.209	0.000	0.000
THAP7	4.240	4.724	0.483	0.000	0.000
THAP8	2.431	3.483	1.052	0.000	0.000

THAP9	1.751	1.952	0.202	0.000	0.000
THBD	3.265	2.593	-0.673	0.000	0.000
THBS1	5.600	4.442	-1.158	0.000	0.000
THBS2	2.939	3.283	0.345	0.001	0.001
THBS3	3.249	3.959	0.710	0.000	0.000
THBS4	0.544	2.494	1.950	0.000	0.000
THEM4	3.021	4.027	1.006	0.000	0.000
THEMIS	0.917	0.734	-0.184	0.000	0.000
THG1L	2.729	3.047	0.318	0.000	0.000
THNSL1	3.682	3.556	-0.127	0.001	0.001
THNSL2	4.082	4.204	0.122	0.052	0.057
THOC1	3.064	3.674	0.610	0.000	0.000
THOC2	3.412	4.185	0.773	0.000	0.000
THOC5	2.758	3.619	0.862	0.000	0.000
THOC6	4.140	4.867	0.727	0.000	0.000
THOC7	5.198	5.937	0.739	0.000	0.000
THOP1	4.082	4.453	0.371	0.000	0.000
THPO	4.041	4.095	0.054	0.317	0.333
THRA	2.423	2.977	0.553	0.000	0.000
THRAP3	5.263	5.744	0.481	0.000	0.000
THRB	3.189	3.411	0.222	0.000	0.000
THRSP	7.268	4.021	-3.247	0.000	0.000
THSD1	1.572	2.002	0.430	0.000	0.000
THSD4	1.786	1.660	-0.126	0.013	0.015
THSD7A	0.948	1.346	0.398	0.000	0.000
THSD7B	0.202	0.252	0.050	0.013	0.015
THTPA	2.347	2.979	0.632	0.000	0.000
THUMPD1	3.795	4.212	0.417	0.000	0.000
THUMPD2	2.205	2.808	0.603	0.000	0.000
THUMPD3	3.623	4.404	0.781	0.000	0.000
THY1	2.031	4.346	2.316	0.000	0.000
THYN1	5.108	5.109	0.000	0.991	0.992
TIA1	4.134	4.747	0.613	0.000	0.000
TIAF1	3.107	3.245	0.138	0.001	0.001
TIAL1	4.221	4.825	0.604	0.000	0.000
TIAM1	2.083	1.482	-0.601	0.000	0.000
TIAM2	0.739	1.187	0.448	0.000	0.000
TICAM1	3.929	4.513	0.583	0.000	0.000
TICAM2	1.073	1.385	0.312	0.000	0.000
TIE1	2.851	3.187	0.336	0.000	0.000
TIFA	2.660	2.954	0.294	0.000	0.000
TIFAB	0.162	0.251	0.089	0.000	0.000
TIGD1	1.369	2.464	1.095	0.000	0.000
TIGD2	3.784	3.580	-0.204	0.000	0.000
TIGD3	0.413	1.065	0.652	0.000	0.000
TIGD4	0.191	0.275	0.084	0.000	0.000
TIGD5	1.287	2.586	1.298	0.000	0.000
TIGD6	1.550	2.324	0.774	0.000	0.000
TIGIT	1.267	1.167	-0.100	0.068	0.074
TIMD4	2.680	1.000	-1.680	0.000	0.000
TIMELESS	3.038	4.234	1.196	0.000	0.000
TIMM10	6.088	6.707	0.619	0.000	0.000

TIMM13	5.702	6.431	0.730	0.000	0.000
TIMM17A	5.295	6.072	0.777	0.000	0.000
TIMM17B	4.583	5.520	0.937	0.000	0.000
TIMM22	2.702	3.143	0.441	0.000	0.000
TIMM44	4.069	4.684	0.615	0.000	0.000
TIMM50	3.844	4.848	1.003	0.000	0.000
TIMM8A	3.263	3.804	0.541	0.000	0.000
TIMM8B	5.871	6.478	0.607	0.000	0.000
TIMM9	4.525	5.329	0.805	0.000	0.000
TIMMDC1	4.944	5.685	0.741	0.000	0.000
TIMP1	8.147	8.035	-0.112	0.291	0.306
TIMP2	5.047	5.028	-0.019	0.808	0.815
TIMP3	4.242	4.502	0.260	0.000	0.000
TIMP4	0.578	0.702	0.124	0.002	0.003
TINAGL1	3.789	3.721	-0.069	0.247	0.261
TINF2	4.464	5.126	0.662	0.000	0.000
TIPARP	3.807	3.904	0.097	0.030	0.033
TIPIN	1.504	2.465	0.960	0.000	0.000
TIPRL	3.973	5.009	1.037	0.000	0.000
TJAP1	3.050	3.961	0.911	0.000	0.000
TJP1	3.644	4.294	0.650	0.000	0.000
TJP2	5.027	4.697	-0.329	0.000	0.000
TJP3	3.325	3.118	-0.207	0.006	0.007
TK1	2.915	5.034	2.120	0.000	0.000
TK2	3.864	3.944	0.080	0.011	0.013
TKT	4.448	6.244	1.796	0.000	0.000
TKTL1	0.150	0.436	0.286	0.000	0.000
TLCD1	2.778	4.379	1.601	0.000	0.000
TLCD2	1.479	1.880	0.400	0.000	0.000
TLE1	4.845	5.745	0.900	0.000	0.000
TLE2	3.210	3.146	-0.064	0.234	0.247
TLE3	2.427	2.849	0.422	0.000	0.000
TLE4	2.047	1.773	-0.274	0.000	0.000
TLE6	0.922	1.697	0.775	0.000	0.000
TLK1	3.386	4.044	0.658	0.000	0.000
TLK2	2.510	3.240	0.731	0.000	0.000
TLL1	0.226	0.196	-0.030	0.107	0.116
TLN1	5.402	6.009	0.606	0.000	0.000
TLN2	1.480	2.039	0.559	0.000	0.000
TLR1	1.842	1.865	0.023	0.648	0.661
TLR10	0.695	0.642	-0.053	0.232	0.246
TLR2	2.743	2.339	-0.404	0.000	0.000
TLR3	2.083	1.781	-0.302	0.000	0.000
TLR4	3.032	2.520	-0.512	0.000	0.000
TLR5	1.246	1.782	0.536	0.000	0.000
TLR6	1.080	1.148	0.068	0.073	0.080
TLR7	0.646	0.819	0.173	0.000	0.000
TLR8	0.984	0.943	-0.041	0.387	0.403
TLX1	0.525	1.823	1.298	0.000	0.000
TM2D1	3.013	3.629	0.616	0.000	0.000
TM2D2	4.108	4.350	0.242	0.000	0.000
TM2D3	3.497	3.857	0.360	0.000	0.000

TM4SF1	5.457	6.084	0.627	0.000	0.000
TM4SF18	2.121	2.637	0.516	0.000	0.000
TM4SF19	0.065	0.820	0.755	0.000	0.000
TM4SF20	0.191	1.094	0.903	0.000	0.000
TM4SF4	8.247	8.825	0.577	0.000	0.000
TM4SF5	7.407	8.045	0.638	0.000	0.000
TM6SF1	1.359	1.511	0.152	0.001	0.001
TM6SF2	4.767	3.649	-1.118	0.000	0.000
TM7SF2	6.025	6.593	0.568	0.000	0.000
TM7SF3	5.525	5.738	0.213	0.000	0.000
TM9SF2	6.527	6.984	0.457	0.000	0.000
TM9SF3	5.430	5.915	0.485	0.000	0.000
TM9SF4	4.585	5.367	0.782	0.000	0.000
TMBIM1	5.704	6.639	0.935	0.000	0.000
TMBIM4	5.234	5.693	0.458	0.000	0.000
TMBIM6	9.224	9.202	-0.022	0.463	0.479
TMC3	0.193	0.215	0.022	0.125	0.134
TMC4	2.428	2.357	-0.071	0.473	0.489
TMC5	0.356	1.317	0.961	0.000	0.000
TMC6	2.749	2.866	0.117	0.077	0.084
TMC7	0.427	1.179	0.753	0.000	0.000
TMC8	3.030	2.456	-0.573	0.000	0.000
TMCC1	3.395	4.058	0.664	0.000	0.000
TMCC2	0.417	0.541	0.124	0.000	0.000
TMCC3	1.703	2.240	0.537	0.000	0.000
TMCO1	5.547	6.707	1.160	0.000	0.000
TMCO3	2.226	3.664	1.438	0.000	0.000
TMCO4	2.991	3.406	0.415	0.000	0.000
TMCO6	3.887	4.459	0.573	0.000	0.000
TMED10	6.713	7.164	0.451	0.000	0.000
TMED2	6.971	7.641	0.669	0.000	0.000
TMED3	1.807	2.652	0.845	0.000	0.000
TMED4	4.696	5.418	0.722	0.000	0.000
TMED5	5.169	5.498	0.329	0.000	0.000
TMED6	1.961	2.161	0.200	0.002	0.002
TMED7	5.698	6.256	0.559	0.000	0.000
TMED7-TICAM	1.346	1.696	0.350	0.000	0.000
TMED8	1.338	1.477	0.140	0.000	0.000
TMED9	6.856	7.924	1.068	0.000	0.000
TMEFF1	0.282	0.664	0.383	0.000	0.000
TMEM100	2.280	1.508	-0.771	0.000	0.000
TMEM101	3.607	4.903	1.296	0.000	0.000
TMEM102	2.148	2.341	0.193	0.000	0.000
TMEM104	2.844	4.006	1.162	0.000	0.000
TMEM105	0.972	1.220	0.248	0.000	0.000
TMEM106A	1.985	1.984	-0.001	0.985	0.985
TMEM106B	3.464	4.071	0.607	0.000	0.000
TMEM106C	3.932	5.832	1.900	0.000	0.000
TMEM107	1.586	2.195	0.609	0.000	0.000
TMEM108	0.084	0.271	0.188	0.000	0.000
TMEM109	5.335	6.185	0.849	0.000	0.000
TMEM11	3.451	3.946	0.495	0.000	0.000

TMEM110-MU	1.885	2.054	0.169	0.000	0.000
TMEM115	4.880	5.781	0.901	0.000	0.000
TMEM116	2.108	2.428	0.320	0.000	0.000
TMEM117	1.727	2.358	0.631	0.000	0.000
TMEM119	1.033	1.578	0.545	0.000	0.000
TMEM120A	6.217	6.629	0.412	0.000	0.000
TMEM120B	1.954	2.753	0.799	0.000	0.000
TMEM121	1.227	0.823	-0.404	0.000	0.000
TMEM123	7.445	7.642	0.198	0.000	0.000
TMEM125	1.286	0.791	-0.495	0.000	0.000
TMEM126A	5.149	5.811	0.662	0.000	0.000
TMEM126B	4.487	5.097	0.610	0.000	0.000
TMEM127	4.404	5.134	0.730	0.000	0.000
TMEM128	3.885	4.366	0.482	0.000	0.000
TMEM129	4.814	5.211	0.397	0.000	0.000
TMEM130	0.249	0.323	0.075	0.036	0.040
TMEM131	2.973	3.613	0.640	0.000	0.000
TMEM132A	0.933	2.148	1.215	0.000	0.000
TMEM132E	0.870	0.442	-0.428	0.000	0.000
TMEM134	4.071	4.269	0.198	0.000	0.000
TMEM135	3.721	4.217	0.496	0.000	0.000
TMEM136	0.900	1.694	0.794	0.000	0.000
TMEM138	3.171	3.877	0.706	0.000	0.000
TMEM139	3.301	3.196	-0.105	0.171	0.182
TMEM140	5.685	5.885	0.200	0.000	0.000
TMEM141	6.164	6.548	0.384	0.000	0.000
TMEM143	3.188	3.361	0.173	0.000	0.000
TMEM144	0.815	1.244	0.429	0.000	0.000
TMEM145	0.114	0.882	0.768	0.000	0.000
TMEM147	5.244	6.380	1.136	0.000	0.000
TMEM14A	5.893	6.971	1.078	0.000	0.000
TMEM14B	4.628	5.497	0.870	0.000	0.000
TMEM14C	6.661	7.545	0.883	0.000	0.000
TMEM150A	4.214	4.932	0.717	0.000	0.000
TMEM150B	1.664	3.362	1.697	0.000	0.000
TMEM150C	3.358	3.297	-0.061	0.322	0.337
TMEM151A	0.418	0.867	0.449	0.000	0.000
TMEM154	1.116	0.638	-0.478	0.000	0.000
TMEM156	1.907	1.895	-0.013	0.874	0.879
TMEM158	1.054	1.002	-0.052	0.322	0.338
TMEM159	1.775	1.728	-0.047	0.441	0.457
TMEM160	4.350	5.209	0.859	0.000	0.000
TMEM161A	4.518	5.291	0.773	0.000	0.000
TMEM161B	2.161	2.714	0.553	0.000	0.000
TMEM163	0.233	0.516	0.283	0.000	0.000
TMEM164	1.678	2.849	1.170	0.000	0.000
TMEM165	3.016	3.999	0.983	0.000	0.000
TMEM167A	4.839	5.636	0.797	0.000	0.000
TMEM167B	3.976	4.566	0.590	0.000	0.000
TMEM168	2.414	3.168	0.754	0.000	0.000
TMEM169	1.014	1.648	0.633	0.000	0.000
TMEM17	1.137	1.588	0.450	0.000	0.000

TMEM170A	2.489	2.544	0.055	0.077	0.084
TMEM170B	2.432	2.893	0.460	0.000	0.000
TMEM171	1.726	2.317	0.591	0.000	0.000
TMEM173	3.455	3.332	-0.123	0.035	0.039
TMEM175	4.349	4.643	0.294	0.000	0.000
TMEM176A	8.931	8.972	0.041	0.458	0.474
TMEM176B	10.184	10.137	-0.047	0.373	0.388
TMEM177	3.711	4.019	0.308	0.000	0.000
TMEM18	3.427	3.772	0.345	0.000	0.000
TMEM181	3.526	4.246	0.720	0.000	0.000
TMEM182	0.732	1.465	0.733	0.000	0.000
TMEM183A	4.016	5.030	1.014	0.000	0.000
TMEM184A	3.656	3.619	-0.037	0.551	0.565
TMEM184B	3.058	4.227	1.169	0.000	0.000
TMEM184C	3.317	3.606	0.289	0.000	0.000
TMEM185B	3.098	3.980	0.882	0.000	0.000
TMEM186	3.318	3.893	0.575	0.000	0.000
TMEM187	3.152	3.747	0.596	0.000	0.000
TMEM189	2.824	3.777	0.953	0.000	0.000
TMEM19	2.902	3.741	0.839	0.000	0.000
TMEM192	3.727	3.480	-0.246	0.000	0.000
TMEM198	1.687	2.708	1.021	0.000	0.000
TMEM200A	0.476	0.722	0.246	0.000	0.000
TMEM200B	2.940	3.473	0.533	0.000	0.000
TMEM200C	0.409	0.290	-0.119	0.000	0.000
TMEM201	1.259	2.257	0.999	0.000	0.000
TMEM203	5.371	5.942	0.571	0.000	0.000
TMEM204	3.596	3.570	-0.026	0.633	0.646
TMEM205	7.659	7.800	0.142	0.000	0.000
TMEM206	1.387	2.541	1.154	0.000	0.000
TMEM208	6.022	6.477	0.455	0.000	0.000
TMEM209	2.685	3.729	1.044	0.000	0.000
TMEM214	5.123	5.758	0.635	0.000	0.000
TMEM216	2.060	2.650	0.590	0.000	0.000
TMEM217	0.901	0.937	0.036	0.288	0.303
TMEM218	2.588	3.193	0.605	0.000	0.000
TMEM219	6.352	6.782	0.430	0.000	0.000
TMEM220	5.735	4.698	-1.037	0.000	0.000
TMEM221	0.364	0.398	0.034	0.125	0.134
TMEM222	4.187	4.626	0.439	0.000	0.000
TMEM223	3.883	4.527	0.643	0.000	0.000
TMEM229B	0.964	1.205	0.241	0.000	0.000
TMEM231	1.084	1.132	0.048	0.194	0.206
TMEM232	0.284	0.271	-0.013	0.348	0.364
TMEM233	0.345	0.577	0.231	0.000	0.000
TMEM234	2.197	2.788	0.591	0.000	0.000
TMEM237	1.330	2.355	1.025	0.000	0.000
TMEM25	3.067	2.165	-0.902	0.000	0.000
TMEM26	1.193	0.564	-0.629	0.000	0.000
TMEM30A	5.718	5.960	0.242	0.000	0.000
TMEM30B	3.569	3.272	-0.297	0.000	0.000
TMEM31	0.208	0.252	0.044	0.025	0.027

TMEM33	4.205	4.854	0.648	0.000	0.000
TMEM37	6.025	6.059	0.034	0.530	0.544
TMEM38A	2.313	2.916	0.603	0.000	0.000
TMEM38B	2.807	4.025	1.218	0.000	0.000
TMEM39A	3.121	3.870	0.749	0.000	0.000
TMEM39B	2.896	3.409	0.514	0.000	0.000
TMEM40	0.093	0.402	0.309	0.000	0.000
TMEM41A	2.990	3.382	0.392	0.000	0.000
TMEM41B	4.139	4.802	0.663	0.000	0.000
TMEM42	3.291	3.918	0.627	0.000	0.000
TMEM43	4.377	4.837	0.460	0.000	0.000
TMEM44	1.992	2.480	0.488	0.000	0.000
TMEM45A	4.714	3.390	-1.324	0.000	0.000
TMEM45B	4.081	5.059	0.979	0.000	0.000
TMEM47	3.901	3.306	-0.595	0.000	0.000
TMEM50A	5.067	5.984	0.916	0.000	0.000
TMEM50B	4.028	4.433	0.405	0.000	0.000
TMEM51	2.573	2.878	0.304	0.000	0.000
TMEM52	2.548	2.494	-0.053	0.355	0.370
TMEM53	4.560	4.228	-0.332	0.000	0.000
TMEM54	3.083	3.992	0.910	0.000	0.000
TMEM56	5.915	5.190	-0.725	0.000	0.000
TMEM59	6.554	6.761	0.208	0.000	0.000
TMEM59L	0.172	0.288	0.116	0.000	0.000
TMEM60	4.601	5.201	0.600	0.000	0.000
TMEM61	0.240	0.714	0.474	0.000	0.000
TMEM62	3.121	3.778	0.657	0.000	0.000
TMEM63A	4.012	4.324	0.311	0.000	0.000
TMEM63B	4.899	5.331	0.432	0.000	0.000
TMEM63C	0.203	0.294	0.091	0.001	0.001
TMEM64	3.224	4.197	0.973	0.000	0.000
TMEM65	1.571	2.644	1.074	0.000	0.000
TMEM67	0.807	1.282	0.476	0.000	0.000
TMEM68	2.055	2.972	0.917	0.000	0.000
TMEM69	3.548	4.542	0.994	0.000	0.000
TMEM70	4.511	5.453	0.942	0.000	0.000
TMEM71	1.329	0.908	-0.420	0.000	0.000
TMEM74	0.367	1.402	1.035	0.000	0.000
TMEM79	1.877	2.910	1.033	0.000	0.000
TMEM80	2.702	3.515	0.813	0.000	0.000
TMEM81	1.991	3.034	1.043	0.000	0.000
TMEM82	5.589	3.410	-2.179	0.000	0.000
TMEM86A	1.964	2.138	0.174	0.000	0.000
TMEM86B	4.735	4.492	-0.243	0.000	0.000
TMEM87A	3.628	4.222	0.594	0.000	0.000
TMEM87B	2.898	3.344	0.445	0.000	0.000
TMEM88	2.665	2.926	0.261	0.000	0.000
TMEM8A	4.842	5.396	0.554	0.000	0.000
TMEM8B	1.919	2.588	0.669	0.000	0.000
TMEM9	4.902	6.193	1.290	0.000	0.000
TMEM91	2.015	2.551	0.536	0.000	0.000
TMEM92	2.025	2.161	0.136	0.141	0.151

TMEM97	5.267	6.200	0.933	0.000	0.000
TMEM98	3.274	4.577	1.303	0.000	0.000
TMEM99	3.258	3.595	0.337	0.000	0.000
TMEM9B	4.449	5.076	0.626	0.000	0.000
TMF1	3.205	3.610	0.405	0.000	0.000
TMIE	0.705	1.414	0.709	0.000	0.000
TMIGD2	0.846	0.628	-0.218	0.000	0.000
TMLHE	2.652	2.973	0.321	0.000	0.000
TMOD1	3.589	3.468	-0.121	0.051	0.056
TMOD2	1.009	1.437	0.428	0.000	0.000
TMOD3	3.191	3.946	0.755	0.000	0.000
TMPO	4.028	4.984	0.956	0.000	0.000
TMPPE	0.598	0.800	0.202	0.000	0.000
TMPRSS13	0.428	0.326	-0.102	0.001	0.001
TMPRSS2	4.657	3.864	-0.793	0.000	0.000
TMPRSS3	1.401	1.560	0.159	0.054	0.059
TMPRSS5	0.594	0.642	0.048	0.077	0.084
TMPRSS6	6.603	6.220	-0.383	0.000	0.000
TMPRSS9	1.329	2.050	0.721	0.000	0.000
TMSB10	9.725	10.757	1.032	0.000	0.000
TMSB15A	0.287	0.609	0.322	0.000	0.000
TMSB15B	0.164	0.202	0.038	0.004	0.005
TMSB4X	9.456	9.701	0.245	0.000	0.000
TMSB4Y	0.851	0.888	0.038	0.403	0.419
TMTC1	0.975	1.250	0.275	0.000	0.000
TMTC2	0.635	0.822	0.186	0.000	0.000
TMTC3	2.545	2.797	0.251	0.000	0.000
TMTC4	2.641	3.256	0.615	0.000	0.000
TMUB1	5.587	6.287	0.701	0.000	0.000
TMUB2	4.126	4.920	0.794	0.000	0.000
TMX1	3.948	4.522	0.574	0.000	0.000
TMX2	5.719	6.486	0.767	0.000	0.000
TMX3	3.595	3.679	0.084	0.056	0.061
TMX4	4.042	4.636	0.594	0.000	0.000
TNC	1.778	2.160	0.382	0.000	0.000
TNF	1.103	0.894	-0.209	0.000	0.000
TNFAIP1	5.101	5.589	0.487	0.000	0.000
TNFAIP2	5.429	5.408	-0.020	0.773	0.783
TNFAIP3	3.957	4.634	0.678	0.000	0.000
TNFAIP6	0.535	0.861	0.326	0.000	0.000
TNFAIP8	1.956	2.146	0.190	0.000	0.000
TNFAIP8L1	4.558	4.655	0.097	0.016	0.018
TNFAIP8L2	2.892	2.695	-0.197	0.000	0.001
TNFAIP8L3	1.516	2.002	0.485	0.000	0.000
TNFRSF10A	2.107	2.373	0.266	0.000	0.000
TNFRSF10B	5.132	5.023	-0.109	0.015	0.016
TNFRSF10C	1.295	1.477	0.182	0.000	0.000
TNFRSF10D	3.646	3.213	-0.434	0.000	0.000
TNFRSF11A	0.539	0.930	0.390	0.000	0.000
TNFRSF11B	3.412	2.545	-0.867	0.000	0.000
TNFRSF12A	5.299	5.958	0.658	0.000	0.000
TNFRSF13B	0.488	0.273	-0.214	0.000	0.000

TNFRSF13C	0.388	0.379	-0.009	0.755	0.765
TNFRSF14	4.882	5.149	0.267	0.000	0.000
TNFRSF17	1.086	0.672	-0.414	0.000	0.000
TNFRSF18	1.171	1.838	0.668	0.000	0.000
TNFRSF19	1.065	1.826	0.762	0.000	0.000
TNFRSF1A	6.523	6.440	-0.082	0.009	0.010
TNFRSF1B	4.955	4.720	-0.235	0.000	0.000
TNFRSF21	3.419	4.045	0.626	0.000	0.000
TNFRSF25	1.819	2.589	0.770	0.000	0.000
TNFRSF4	1.272	2.567	1.295	0.000	0.000
TNFRSF6B	1.351	1.525	0.174	0.000	0.000
TNFRSF8	0.323	0.410	0.087	0.000	0.000
TNFRSF9	0.428	0.675	0.247	0.000	0.000
TNFSF10	6.151	6.108	-0.044	0.466	0.481
TNFSF11	1.500	1.106	-0.394	0.000	0.000
TNFSF12	4.245	4.164	-0.081	0.114	0.123
TNFSF13	2.938	2.768	-0.169	0.001	0.001
TNFSF13B	2.584	2.507	-0.077	0.193	0.205
TNFSF14	3.997	3.727	-0.271	0.005	0.005
TNFSF15	0.406	0.879	0.474	0.000	0.000
TNFSF18	0.298	0.429	0.131	0.000	0.000
TNFSF4	1.019	1.905	0.885	0.000	0.000
TNFSF8	1.096	1.009	-0.086	0.048	0.053
TNFSF9	0.736	0.998	0.262	0.000	0.000
TNIK	1.672	1.600	-0.072	0.213	0.226
TNIP1	5.546	6.238	0.692	0.000	0.000
TNIP2	4.169	4.896	0.726	0.000	0.000
TNIP3	0.294	0.252	-0.042	0.053	0.058
TNK1	2.152	2.221	0.069	0.059	0.065
TNK2	3.219	3.556	0.337	0.000	0.000
TNKS	2.331	2.759	0.428	0.000	0.000
TNKS1BP1	5.357	5.476	0.119	0.002	0.002
TNKS2	3.899	4.217	0.319	0.000	0.000
TNN	0.974	0.747	-0.227	0.000	0.000
TNNC1	0.776	1.754	0.978	0.000	0.000
TNNC2	0.605	0.909	0.304	0.000	0.000
TNNI1	0.945	1.126	0.181	0.000	0.000
TNNI2	1.063	1.665	0.602	0.000	0.000
TNNT1	0.171	0.706	0.535	0.000	0.000
TNNT2	0.247	0.500	0.253	0.000	0.000
TNNT3	0.223	0.249	0.025	0.283	0.298
TNPO1	3.451	4.167	0.716	0.000	0.000
TNPO2	3.475	4.163	0.688	0.000	0.000
TNPO3	3.904	4.697	0.793	0.000	0.000
TNRC18	3.441	4.490	1.049	0.000	0.000
TNRC6A	3.746	3.875	0.129	0.003	0.003
TNRC6B	2.229	2.448	0.219	0.000	0.000
TNRC6C	1.936	2.045	0.108	0.003	0.003
TNS1	3.906	3.948	0.042	0.413	0.429
TNS3	4.514	5.007	0.493	0.000	0.000
TNXB	2.368	1.477	-0.891	0.000	0.000
TOB1	6.587	6.898	0.311	0.000	0.000

TOB2	4.066	4.735	0.669	0.000	0.000
TOE1	2.823	3.247	0.424	0.000	0.000
TOLLIP	5.701	5.972	0.271	0.000	0.000
TOM1	4.945	5.520	0.575	0.000	0.000
TOM1L1	4.330	4.800	0.471	0.000	0.000
TOM1L2	2.815	3.283	0.468	0.000	0.000
TOMM20	5.968	7.133	1.165	0.000	0.000
TOMM20L	0.649	0.855	0.206	0.000	0.000
TOMM22	5.322	6.100	0.778	0.000	0.000
TOMM34	3.983	5.002	1.019	0.000	0.000
TOMM40	4.342	5.578	1.236	0.000	0.000
TOMM40L	4.146	5.151	1.004	0.000	0.000
TOMM5	3.912	4.708	0.797	0.000	0.000
TOMM6	4.066	4.877	0.811	0.000	0.000
TOMM7	6.738	7.297	0.558	0.000	0.000
TONSL	1.273	2.739	1.467	0.000	0.000
TOP1	5.165	5.635	0.470	0.000	0.000
TOP1MT	3.626	4.394	0.768	0.000	0.000
TOP2A	1.288	4.540	3.252	0.000	0.000
TOP2B	4.814	5.404	0.589	0.000	0.000
TOP3A	2.789	3.269	0.479	0.000	0.000
TOP3B	1.769	2.241	0.472	0.000	0.000
TOPBP1	2.676	3.746	1.070	0.000	0.000
TOPORS	3.549	3.792	0.243	0.000	0.000
TOR1A	4.608	4.806	0.198	0.000	0.000
TOR1AIP1	3.605	4.154	0.549	0.000	0.000
TOR1AIP2	3.820	4.509	0.689	0.000	0.000
TOR1B	4.196	4.785	0.589	0.000	0.000
TOR2A	2.790	3.497	0.707	0.000	0.000
TOR3A	3.983	5.161	1.179	0.000	0.000
TOX	0.989	1.110	0.121	0.041	0.045
TOX2	2.026	2.390	0.364	0.000	0.000
TOX3	2.102	2.767	0.665	0.000	0.000
TOX4	3.924	4.599	0.675	0.000	0.000
TP53	3.622	4.203	0.582	0.000	0.000
TP53BP1	1.771	2.464	0.693	0.000	0.000
TP53BP2	3.580	4.895	1.314	0.000	0.000
TP53I11	2.656	3.496	0.840	0.000	0.000
TP53I13	4.638	4.629	-0.009	0.878	0.883
TP53I3	2.999	4.944	1.944	0.000	0.000
TP53INP1	5.763	6.281	0.518	0.000	0.000
TP53INP2	4.801	5.043	0.242	0.000	0.000
TP53RK	2.763	3.464	0.700	0.000	0.000
TP63	0.206	0.415	0.209	0.000	0.000
TP73	0.381	1.162	0.781	0.000	0.000
TPBG	1.008	0.917	-0.091	0.109	0.117
TPCN1	3.783	4.379	0.596	0.000	0.000
TPCN2	3.121	3.548	0.427	0.000	0.000
TPD52	3.728	4.720	0.991	0.000	0.000
TPD52L1	3.978	3.474	-0.504	0.000	0.000
TPD52L2	4.494	5.636	1.141	0.000	0.000
TPH1	0.168	0.255	0.087	0.000	0.000

TPI1	7.787	8.471	0.684	0.000	0.000
TPK1	1.392	1.282	-0.110	0.000	0.000
TPM1	4.427	5.389	0.962	0.000	0.000
TPM2	3.522	4.863	1.342	0.000	0.000
TPM3	5.467	6.686	1.219	0.000	0.000
TPM4	4.884	5.730	0.846	0.000	0.000
TPMT	5.688	5.550	-0.138	0.000	0.000
TPP1	6.071	6.984	0.913	0.000	0.000
TPP2	4.112	4.332	0.220	0.000	0.000
TPPP	1.869	2.093	0.224	0.000	0.000
TPPP2	2.554	1.075	-1.480	0.000	0.000
TPPP3	2.498	2.487	-0.010	0.873	0.878
TPR	4.250	5.026	0.776	0.000	0.000
TPRA1	4.207	4.934	0.727	0.000	0.000
TPRG1	1.681	1.274	-0.407	0.000	0.000
TPRG1L	6.091	6.094	0.003	0.932	0.935
TPRKB	3.874	4.832	0.958	0.000	0.000
TPRN	2.964	3.863	0.899	0.000	0.000
TPSAB1	1.624	1.379	-0.245	0.001	0.001
TPSB2	1.578	1.358	-0.220	0.002	0.003
TPSD1	0.277	0.267	-0.009	0.719	0.730
TPSG1	0.444	0.603	0.158	0.001	0.001
TPST1	4.035	4.128	0.092	0.111	0.119
TPST2	4.751	4.998	0.248	0.000	0.000
TPX2	2.172	4.194	2.022	0.000	0.000
TRA2A	4.750	5.247	0.498	0.000	0.000
TRA2B	4.426	4.778	0.352	0.000	0.000
TRABD	4.448	5.193	0.745	0.000	0.000
TRADD	3.730	4.286	0.556	0.000	0.000
TRAF1	2.282	2.753	0.471	0.000	0.000
TRAF2	3.045	4.350	1.305	0.000	0.000
TRAF3	2.191	2.823	0.632	0.000	0.000
TRAF3IP1	2.637	3.258	0.621	0.000	0.000
TRAF3IP2	2.335	2.562	0.227	0.000	0.000
TRAF3IP3	1.771	1.659	-0.112	0.018	0.020
TRAF4	4.864	5.267	0.402	0.000	0.000
TRAF5	1.423	2.304	0.881	0.000	0.000
TRAF6	2.541	2.746	0.205	0.000	0.000
TRAF7	4.430	5.378	0.948	0.000	0.000
TRAFD1	3.466	4.158	0.692	0.000	0.000
TRAIP	0.653	2.129	1.476	0.000	0.000
TRAK1	2.813	3.364	0.552	0.000	0.000
TRAK2	3.175	3.713	0.538	0.000	0.000
TRAM1	6.477	7.482	1.005	0.000	0.000
TRAM1L1	0.279	1.002	0.723	0.000	0.000
TRAM2	3.726	3.497	-0.229	0.000	0.000
TRANK1	3.095	2.790	-0.306	0.000	0.000
TRAP1	5.572	5.850	0.278	0.000	0.000
TRAPPC1	5.967	6.550	0.583	0.000	0.000
TRAPPC10	2.654	2.965	0.311	0.000	0.000
TRAPPC2	2.273	2.843	0.570	0.000	0.000
TRAPPC2L	3.955	4.417	0.463	0.000	0.000

TRAPPC3	4.690	5.345	0.655	0.000	0.000
TRAPPC4	3.685	4.437	0.752	0.000	0.000
TRAPPC5	2.968	3.173	0.205	0.000	0.000
TRAPPC6A	5.591	6.200	0.609	0.000	0.000
TRAPPC6B	3.842	4.005	0.163	0.000	0.000
TRAPPC8	3.771	3.919	0.148	0.000	0.000
TRAPPC9	2.792	3.777	0.985	0.000	0.000
TRAT1	1.152	0.884	-0.268	0.000	0.000
TRDMT1	0.586	0.749	0.164	0.000	0.000
TREH	2.864	1.484	-1.380	0.000	0.000
TREM1	0.749	0.787	0.038	0.478	0.493
TREM2	1.614	3.335	1.721	0.000	0.000
TREML1	0.259	0.464	0.205	0.000	0.000
TREML2	0.232	0.205	-0.026	0.179	0.191
TRERF1	1.061	1.070	0.009	0.820	0.827
TREX1	2.188	2.293	0.105	0.004	0.005
TREX2	0.370	0.507	0.137	0.000	0.000
TRHDE	0.268	0.456	0.188	0.000	0.000
TRIAP1	5.604	5.901	0.297	0.000	0.000
TRIB1	6.629	5.740	-0.888	0.000	0.000
TRIB2	3.318	3.925	0.607	0.000	0.000
TRIB3	4.694	5.507	0.814	0.000	0.000
TRIL	0.516	0.828	0.312	0.000	0.000
TRIM10	1.930	1.867	-0.063	0.191	0.203
TRIM11	2.050	3.227	1.177	0.000	0.000
TRIM13	2.560	2.731	0.171	0.000	0.000
TRIM14	3.984	4.262	0.278	0.000	0.000
TRIM15	4.238	3.622	-0.617	0.000	0.000
TRIM16	1.192	2.895	1.703	0.000	0.000
TRIM16L	1.742	3.332	1.591	0.000	0.000
TRIM17	0.123	0.521	0.397	0.000	0.000
TRIM2	2.767	2.751	-0.016	0.744	0.755
TRIM21	4.142	4.559	0.417	0.000	0.000
TRIM22	4.523	3.749	-0.774	0.000	0.000
TRIM23	2.616	3.060	0.444	0.000	0.000
TRIM24	3.292	4.405	1.113	0.000	0.000
TRIM25	3.860	4.385	0.525	0.000	0.000
TRIM26	5.239	5.813	0.574	0.000	0.000
TRIM27	4.218	5.055	0.837	0.000	0.000
TRIM28	5.890	6.971	1.081	0.000	0.000
TRIM29	0.515	0.584	0.069	0.090	0.097
TRIM3	2.115	2.670	0.555	0.000	0.000
TRIM31	1.245	2.792	1.547	0.000	0.000
TRIM32	2.016	2.779	0.763	0.000	0.000
TRIM33	3.181	3.698	0.516	0.000	0.000
TRIM34	1.142	1.380	0.239	0.000	0.000
TRIM35	3.623	3.512	-0.111	0.024	0.027
TRIM36	0.328	0.579	0.251	0.000	0.000
TRIM37	2.489	3.518	1.029	0.000	0.000
TRIM38	2.898	3.171	0.273	0.000	0.000
TRIM39	2.732	3.464	0.732	0.000	0.000
TRIM4	3.539	4.203	0.664	0.000	0.000

TRIM40	0.323	0.394	0.071	0.082	0.089
TRIM41	2.805	3.381	0.576	0.000	0.000
TRIM44	3.173	3.487	0.314	0.000	0.000
TRIM45	0.940	2.108	1.168	0.000	0.000
TRIM46	0.208	0.476	0.268	0.000	0.000
TRIM47	3.479	4.437	0.959	0.000	0.000
TRIM5	3.614	3.819	0.205	0.000	0.000
TRIM50	0.303	1.411	1.107	0.000	0.000
TRIM52	1.892	2.643	0.751	0.000	0.000
TRIM54	0.116	0.757	0.641	0.000	0.000
TRIM55	3.116	4.147	1.031	0.000	0.000
TRIM56	2.447	3.002	0.556	0.000	0.000
TRIM58	0.214	0.154	-0.060	0.000	0.000
TRIM59	0.398	1.132	0.734	0.000	0.000
TRIM6	0.674	1.384	0.710	0.000	0.000
TRIM61	0.329	0.289	-0.040	0.077	0.083
TRIM62	1.358	1.846	0.488	0.000	0.000
TRIM65	2.437	3.530	1.093	0.000	0.000
TRIM66	2.025	2.246	0.221	0.000	0.000
TRIM68	2.114	2.807	0.693	0.000	0.000
TRIM69	2.135	2.309	0.174	0.000	0.000
TRIM7	0.271	0.701	0.430	0.000	0.000
TRIM73	0.525	0.552	0.027	0.336	0.352
TRIM74	0.787	0.784	-0.004	0.924	0.928
TRIM8	5.510	6.160	0.651	0.000	0.000
TRIM9	0.343	0.590	0.247	0.000	0.000
TRIO	2.509	3.257	0.748	0.000	0.000
TRIOBP	2.427	3.317	0.890	0.000	0.000
TRIP10	3.363	4.044	0.682	0.000	0.000
TRIP11	2.840	3.156	0.316	0.000	0.000
TRIP12	3.920	4.544	0.623	0.000	0.000
TRIP13	0.540	2.318	1.778	0.000	0.000
TRIP4	3.360	4.236	0.876	0.000	0.000
TRIP6	5.301	5.875	0.574	0.000	0.000
TRIT1	3.274	3.831	0.558	0.000	0.000
TRMT1	4.047	4.957	0.910	0.000	0.000
TRMT11	4.023	4.007	-0.016	0.705	0.717
TRMT112	6.481	7.437	0.956	0.000	0.000
TRMT12	2.688	3.583	0.895	0.000	0.000
TRMT1L	3.347	3.967	0.620	0.000	0.000
TRMT2A	4.420	5.191	0.771	0.000	0.000
TRMT2B	2.398	3.091	0.694	0.000	0.000
TRMT5	2.394	2.949	0.555	0.000	0.000
TRMT6	2.646	3.710	1.064	0.000	0.000
TRMT61A	3.156	4.091	0.935	0.000	0.000
TRMT61B	3.229	3.930	0.700	0.000	0.000
TRMU	2.801	3.518	0.718	0.000	0.000
TRNAU1AP	2.526	3.389	0.863	0.000	0.000
TRNP1	2.630	3.792	1.162	0.000	0.000
TRNT1	2.587	3.218	0.631	0.000	0.000
TRO	2.792	2.296	-0.496	0.000	0.000
TROAP	0.580	2.739	2.159	0.000	0.000

TROVE2	2.376	3.096	0.721	0.000	0.000
TRPA1	0.141	0.276	0.135	0.000	0.000
TRPC1	0.840	1.309	0.469	0.000	0.000
TRPC4	0.332	0.479	0.147	0.000	0.000
TRPC5	0.318	0.294	-0.024	0.283	0.298
TRPC6	0.211	0.642	0.431	0.000	0.000
TRPM2	0.920	1.458	0.538	0.000	0.000
TRPM4	2.357	3.245	0.888	0.000	0.000
TRPM7	3.302	3.691	0.389	0.000	0.000
TRPM8	3.735	2.813	-0.923	0.000	0.000
TRPS1	0.906	1.374	0.468	0.000	0.000
TRPT1	4.528	5.014	0.486	0.000	0.000
TRPV1	2.070	1.847	-0.224	0.000	0.000
TRPV2	2.561	2.909	0.348	0.000	0.000
TRPV3	0.777	0.364	-0.413	0.000	0.000
TRPV4	3.291	2.016	-1.274	0.000	0.000
TRPV6	1.044	0.356	-0.688	0.000	0.000
TRRAP	2.596	3.320	0.724	0.000	0.000
TRUB1	3.140	3.565	0.424	0.000	0.000
TRUB2	4.186	4.493	0.307	0.000	0.000
TSC1	2.566	3.280	0.714	0.000	0.000
TSC2	3.879	4.448	0.569	0.000	0.000
TSC22D1	5.495	5.658	0.164	0.003	0.004
TSC22D2	3.110	3.106	-0.003	0.938	0.941
TSC22D3	5.651	5.368	-0.282	0.000	0.000
TSC22D4	3.877	4.677	0.800	0.000	0.000
TSEN15	3.650	4.768	1.118	0.000	0.000
TSEN2	1.896	2.513	0.617	0.000	0.000
TSEN34	4.287	5.062	0.775	0.000	0.000
TSEN54	3.628	4.597	0.969	0.000	0.000
TSFM	4.503	4.972	0.469	0.000	0.000
TSG101	4.831	5.623	0.791	0.000	0.000
TSGA10	0.762	1.104	0.343	0.000	0.000
TSGA10IP	0.081	0.192	0.111	0.000	0.000
TSHR	0.318	0.228	-0.090	0.000	0.000
TSHZ1	2.731	3.047	0.315	0.000	0.000
TSHZ2	2.429	2.627	0.198	0.000	0.000
TSHZ3	1.102	1.072	-0.029	0.448	0.464
TSKS	0.319	0.269	-0.050	0.024	0.027
TSKU	6.650	6.297	-0.354	0.000	0.000
TSLP	2.417	1.132	-1.286	0.000	0.000
TSN	4.477	5.370	0.893	0.000	0.000
TSNARE1	2.039	2.770	0.730	0.000	0.000
TSNAX	3.456	4.416	0.960	0.000	0.000
TSNAXIP1	1.046	0.887	-0.160	0.000	0.000
TSPAN1	2.381	2.321	-0.060	0.309	0.325
TSPAN10	0.791	1.406	0.615	0.000	0.000
TSPAN11	0.538	0.418	-0.120	0.000	0.000
TSPAN12	4.203	3.800	-0.404	0.000	0.000
TSPAN13	4.572	4.676	0.104	0.275	0.290
TSPAN14	2.920	3.417	0.497	0.000	0.000
TSPAN15	2.612	3.579	0.967	0.000	0.000

TSPAN17	3.232	4.373	1.141	0.000	0.000
TSPAN18	1.918	2.392	0.474	0.000	0.000
TSPAN2	0.719	0.718	-0.001	0.972	0.974
TSPAN3	4.327	5.028	0.701	0.000	0.000
TSPAN31	3.878	4.408	0.530	0.000	0.000
TSPAN32	0.951	0.835	-0.116	0.001	0.001
TSPAN33	4.734	5.590	0.856	0.000	0.000
TSPAN4	4.266	4.575	0.309	0.000	0.000
TSPAN5	0.828	1.498	0.670	0.000	0.000
TSPAN6	5.725	6.085	0.360	0.000	0.000
TSPAN7	3.864	3.543	-0.320	0.000	0.000
TSPAN8	4.770	5.806	1.036	0.000	0.000
TSPAN9	5.098	4.911	-0.187	0.000	0.000
TSPEAR	0.236	0.556	0.320	0.000	0.000
TSPO	5.447	5.917	0.470	0.000	0.000
TSPO2	0.525	1.254	0.729	0.000	0.000
TSPYL1	5.481	5.407	-0.074	0.038	0.042
TSPYL2	3.799	3.887	0.088	0.130	0.140
TSPYL4	2.751	2.934	0.183	0.000	0.000
TSPYL5	1.481	0.966	-0.515	0.000	0.000
TSR1	3.432	3.854	0.422	0.000	0.000
TSR2	5.287	5.897	0.610	0.000	0.000
TSSC4	4.361	4.989	0.628	0.000	0.000
TSSK3	1.183	1.375	0.192	0.000	0.000
TSSK6	1.067	1.917	0.851	0.000	0.000
TST	8.736	8.499	-0.237	0.000	0.000
TSTA3	5.097	6.078	0.981	0.000	0.000
TSTD1	5.686	6.065	0.379	0.000	0.000
TTBK1	1.035	1.150	0.115	0.012	0.014
TTBK2	1.178	1.366	0.188	0.000	0.000
TTC1	5.131	5.988	0.857	0.000	0.000
TTC12	2.265	2.456	0.191	0.000	0.000
TTC13	2.736	3.959	1.223	0.000	0.000
TTC14	3.150	3.404	0.254	0.000	0.000
TTC16	0.229	0.219	-0.009	0.471	0.487
TTC17	4.138	4.497	0.359	0.000	0.000
TTC19	3.972	3.892	-0.080	0.019	0.022
TTC21A	1.028	1.114	0.086	0.006	0.007
TTC21B	2.152	2.613	0.460	0.000	0.000
TTC22	1.379	1.749	0.370	0.000	0.000
TTC23	2.499	3.174	0.675	0.000	0.000
TTC23L	0.156	0.307	0.151	0.000	0.000
TTC24	0.317	0.262	-0.054	0.020	0.022
TTC25	0.542	0.683	0.141	0.000	0.000
TTC26	0.748	1.233	0.485	0.000	0.000
TTC27	2.897	3.790	0.893	0.000	0.000
TTC28	1.949	2.369	0.420	0.000	0.000
TTC3	4.027	4.469	0.443	0.000	0.000
TTC30A	1.373	1.785	0.412	0.000	0.000
TTC30B	1.206	1.689	0.484	0.000	0.000
TTC31	4.202	4.656	0.454	0.000	0.000
TTC32	2.857	3.420	0.563	0.000	0.000

TTC33	3.267	3.845	0.579	0.000	0.000
TTC36	7.585	3.420	-4.165	0.000	0.000
TTC37	3.694	4.545	0.851	0.000	0.000
TTC38	6.968	6.706	-0.261	0.000	0.000
TTC39A	0.411	1.863	1.452	0.000	0.000
TTC39B	1.790	1.434	-0.356	0.000	0.000
TTC39C	5.300	5.589	0.289	0.000	0.000
TTC4	2.147	2.774	0.627	0.000	0.000
TTC5	1.214	1.735	0.521	0.000	0.000
TTC7A	2.946	3.638	0.692	0.000	0.000
TTC7B	1.790	1.726	-0.064	0.053	0.058
TTC8	2.618	2.845	0.227	0.000	0.000
TTC9	1.832	2.934	1.102	0.000	0.000
TTC9C	3.182	4.040	0.857	0.000	0.000
TTF1	2.332	2.920	0.589	0.000	0.000
TTF2	1.007	1.871	0.864	0.000	0.000
TTI1	3.172	3.910	0.738	0.000	0.000
TTK	0.369	2.152	1.783	0.000	0.000
TTL	1.941	2.963	1.022	0.000	0.000
TTLL1	1.586	2.603	1.017	0.000	0.000
TTLL11	1.607	1.794	0.187	0.000	0.000
TTLL12	3.877	4.756	0.879	0.000	0.000
TTLL2	0.991	0.783	-0.208	0.000	0.000
TTLL3	2.114	2.456	0.342	0.000	0.000
TTLL4	2.344	3.520	1.176	0.000	0.000
TTLL5	1.531	2.072	0.541	0.000	0.000
TTLL6	0.110	0.331	0.222	0.000	0.000
TTLL7	0.749	1.057	0.308	0.000	0.000
TTN	0.315	0.324	0.009	0.590	0.605
TTPA	6.023	5.522	-0.501	0.000	0.000
TTPAL	3.788	3.567	-0.221	0.000	0.000
TTR	12.325	10.402	-1.923	0.000	0.000
TTYH1	0.690	0.618	-0.072	0.200	0.212
TTYH2	1.296	1.843	0.547	0.000	0.000
TTYH3	3.736	4.798	1.062	0.000	0.000
TUB	0.850	1.011	0.161	0.002	0.002
TUBA1A	3.564	4.196	0.631	0.000	0.000
TUBA1C	3.948	5.310	1.362	0.000	0.000
TUBA3D	0.339	0.582	0.243	0.000	0.000
TUBA4A	4.178	4.914	0.737	0.000	0.000
TUBA8	0.909	1.179	0.269	0.000	0.000
TUBB	7.275	8.510	1.235	0.000	0.000
TUBB1	0.436	0.471	0.036	0.048	0.053
TUBB2A	4.723	5.788	1.065	0.000	0.000
TUBB2B	1.156	1.911	0.756	0.000	0.000
TUBB3	0.336	0.634	0.298	0.000	0.000
TUBB6	3.515	3.603	0.088	0.159	0.170
TUBD1	2.090	2.762	0.672	0.000	0.000
TUBE1	3.995	2.895	-1.100	0.000	0.000
TUBG1	3.553	5.107	1.554	0.000	0.000
TUBG2	2.641	3.703	1.062	0.000	0.000
TUBGCP2	4.164	4.659	0.495	0.000	0.000

TUBGCP3	2.893	3.293	0.401	0.000	0.000
TUBGCP4	1.649	2.103	0.454	0.000	0.000
TUBGCP6	3.584	4.169	0.585	0.000	0.000
TUFM	7.622	7.983	0.360	0.000	0.000
TUFT1	2.257	3.467	1.209	0.000	0.000
TULP3	2.673	3.653	0.980	0.000	0.000
TULP4	1.536	2.217	0.681	0.000	0.000
TUSC1	3.565	3.043	-0.522	0.000	0.000
TUSC2	4.432	5.309	0.877	0.000	0.000
TUSC3	2.382	2.161	-0.221	0.007	0.008
TUT1	3.871	4.264	0.393	0.000	0.000
TWF1	4.545	5.345	0.799	0.000	0.000
TWF2	4.675	5.689	1.015	0.000	0.000
TWIST1	0.406	0.560	0.155	0.000	0.000
TWIST2	0.349	0.416	0.067	0.122	0.131
TWISTNB	3.008	3.570	0.562	0.000	0.000
TWSG1	2.688	3.007	0.320	0.000	0.000
TXK	1.091	0.836	-0.255	0.000	0.000
TXLNA	4.575	5.175	0.600	0.000	0.000
TXLNB	0.468	0.694	0.226	0.000	0.000
TXLNG	3.081	3.697	0.616	0.000	0.000
TXN	8.065	9.202	1.137	0.000	0.000
TXN2	6.644	6.911	0.267	0.000	0.000
TXNDC11	5.602	5.707	0.104	0.000	0.000
TXNDC12	4.921	5.676	0.755	0.000	0.000
TXNDC15	4.243	4.775	0.532	0.000	0.000
TXNDC16	2.268	2.909	0.640	0.000	0.000
TXNDC17	5.073	5.453	0.380	0.000	0.000
TXNDC5	3.993	4.687	0.693	0.000	0.000
TXNDC9	3.637	4.497	0.860	0.000	0.000
TXNL1	4.070	4.681	0.611	0.000	0.000
TXNL4A	4.281	5.195	0.914	0.000	0.000
TXNL4B	3.421	3.802	0.381	0.000	0.000
TXNRD1	4.230	6.019	1.789	0.000	0.000
TXNRD2	4.422	4.672	0.250	0.000	0.000
TXNRD3	2.732	3.118	0.387	0.000	0.000
TYK2	4.785	5.361	0.575	0.000	0.000
TYMP	5.870	6.385	0.515	0.000	0.000
TYMS	3.108	4.727	1.619	0.000	0.000
TYRO3	0.797	1.570	0.774	0.000	0.000
TYROBP	6.641	6.447	-0.194	0.006	0.006
TYSND1	2.954	3.837	0.883	0.000	0.000
TYW3	3.179	3.641	0.462	0.000	0.000
TYW5	1.600	1.975	0.375	0.000	0.000
U2AF1	2.995	3.319	0.324	0.000	0.000
U2AF1L4	2.988	3.308	0.320	0.000	0.000
U2AF2	5.456	6.294	0.837	0.000	0.000
U2SURP	3.738	4.544	0.806	0.000	0.000
UACA	3.311	3.891	0.581	0.000	0.000
UAP1	6.350	6.302	-0.048	0.227	0.240
UAP1L1	1.820	2.170	0.350	0.000	0.000
UBA1	5.819	6.583	0.764	0.000	0.000

UBA2	4.354	5.349	0.995	0.000	0.000
UBA3	3.929	4.579	0.650	0.000	0.000
UBA5	3.737	4.378	0.641	0.000	0.000
UBA52	7.674	8.415	0.741	0.000	0.000
UBA6	2.747	3.069	0.322	0.000	0.000
UBA7	4.779	5.100	0.322	0.000	0.000
UBAC1	4.233	4.856	0.623	0.000	0.000
UBAC2	4.680	5.487	0.807	0.000	0.000
UBAP1	5.205	5.555	0.350	0.000	0.000
UBAP2	2.182	3.037	0.855	0.000	0.000
UBAP2L	4.211	5.567	1.355	0.000	0.000
UBASH3A	1.078	0.945	-0.133	0.003	0.003
UBASH3B	1.036	1.103	0.068	0.147	0.158
UBC	8.738	9.125	0.387	0.000	0.000
UBD	4.988	7.863	2.875	0.000	0.000
UBE2A	3.817	4.880	1.064	0.000	0.000
UBE2B	5.511	6.064	0.553	0.000	0.000
UBE2C	1.277	4.595	3.317	0.000	0.000
UBE2D1	3.022	3.724	0.702	0.000	0.000
UBE2D2	4.946	5.706	0.759	0.000	0.000
UBE2D3	5.940	5.983	0.043	0.107	0.115
UBE2D4	3.430	3.854	0.424	0.000	0.000
UBE2E1	4.564	5.355	0.792	0.000	0.000
UBE2E2	3.555	3.486	-0.068	0.188	0.200
UBE2E3	4.471	5.208	0.738	0.000	0.000
UBE2F	3.924	4.493	0.569	0.000	0.000
UBE2F-SCLY	1.673	1.830	0.157	0.000	0.000
UBE2G1	4.258	4.525	0.267	0.000	0.000
UBE2G2	4.458	4.811	0.353	0.000	0.000
UBE2H	4.741	5.395	0.654	0.000	0.000
UBE2I	3.532	4.290	0.758	0.000	0.000
UBE2J1	4.523	5.032	0.509	0.000	0.000
UBE2J2	4.106	4.677	0.572	0.000	0.000
UBE2K	4.938	5.618	0.680	0.000	0.000
UBE2L3	5.053	5.763	0.710	0.000	0.000
UBE2L6	6.390	6.368	-0.022	0.679	0.692
UBE2M	4.835	5.928	1.094	0.000	0.000
UBE2N	4.041	4.805	0.764	0.000	0.000
UBE2O	2.705	3.718	1.012	0.000	0.000
UBE2Q1	4.735	6.023	1.288	0.000	0.000
UBE2Q2	2.697	3.761	1.064	0.000	0.000
UBE2QL1	1.215	1.239	0.024	0.664	0.676
UBE2R2	5.053	5.576	0.523	0.000	0.000
UBE2T	1.624	4.428	2.804	0.000	0.000
UBE2V1	3.447	4.173	0.726	0.000	0.000
UBE2V2	4.051	4.840	0.789	0.000	0.000
UBE2W	2.758	3.313	0.554	0.000	0.000
UBE2Z	4.294	5.250	0.955	0.000	0.000
UBE3A	3.844	4.177	0.333	0.000	0.000
UBE3B	2.725	3.475	0.750	0.000	0.000
UBE3C	3.652	4.457	0.805	0.000	0.000
UBE4B	3.039	3.768	0.729	0.000	0.000

UBFD1	3.340	4.071	0.731	0.000	0.000
UBIAD1	3.003	3.739	0.737	0.000	0.000
UBL3	4.766	4.783	0.016	0.683	0.695
UBL4A	4.270	5.383	1.114	0.000	0.000
UBL5	7.088	7.764	0.677	0.000	0.000
UBL7	4.444	5.471	1.027	0.000	0.000
UBLCP1	3.723	4.361	0.637	0.000	0.000
UBN1	3.159	3.846	0.687	0.000	0.000
UBN2	1.872	2.328	0.456	0.000	0.000
UBOX5	1.997	2.619	0.622	0.000	0.000
UBP1	3.628	4.289	0.660	0.000	0.000
UBQLN1	4.915	5.530	0.615	0.000	0.000
UBQLN2	4.057	4.571	0.514	0.000	0.000
UBQLN4	3.762	5.126	1.364	0.000	0.000
UBQLNL	0.764	0.808	0.045	0.182	0.193
UBR1	2.315	2.624	0.308	0.000	0.000
UBR2	3.645	4.001	0.356	0.000	0.000
UBR3	3.733	3.875	0.143	0.000	0.000
UBR4	4.432	4.563	0.131	0.001	0.001
UBR5	3.359	4.421	1.062	0.000	0.000
UBR7	3.504	4.190	0.686	0.000	0.000
UBTD1	3.430	4.085	0.655	0.000	0.000
UBTD2	3.400	4.093	0.693	0.000	0.000
UBTF	4.554	5.266	0.712	0.000	0.000
UBXN1	5.635	6.383	0.748	0.000	0.000
UBXN10	2.429	2.103	-0.326	0.000	0.000
UBXN11	3.106	3.549	0.442	0.000	0.000
UBXN2A	2.052	2.733	0.681	0.000	0.000
UBXN2B	3.359	4.375	1.017	0.000	0.000
UBXN4	5.906	6.316	0.410	0.000	0.000
UBXN6	5.678	5.927	0.249	0.000	0.000
UBXN7	1.830	2.274	0.444	0.000	0.000
UBXN8	4.152	3.891	-0.261	0.000	0.000
UCHL1	0.732	1.935	1.203	0.000	0.000
UCHL3	3.904	4.071	0.168	0.000	0.000
UCHL5	3.177	4.130	0.953	0.000	0.000
UCK1	4.904	5.226	0.322	0.000	0.000
UCK2	2.323	4.071	1.747	0.000	0.000
UCKL1	4.408	5.155	0.747	0.000	0.000
UCN	1.822	2.383	0.561	0.000	0.000
UCN2	0.076	0.349	0.274	0.000	0.000
UCP2	4.210	4.324	0.114	0.092	0.099
UCP3	0.597	0.830	0.233	0.000	0.000
UEVLD	2.240	2.890	0.650	0.000	0.000
UFC1	5.633	6.832	1.199	0.000	0.000
UFM1	5.000	5.281	0.281	0.000	0.000
UFSP1	1.697	2.407	0.710	0.000	0.000
UFSP2	4.033	4.131	0.099	0.002	0.002
UGCG	3.697	4.083	0.386	0.000	0.000
UGDH	5.705	6.587	0.882	0.000	0.000
UGGT1	3.458	4.581	1.123	0.000	0.000
UGGT2	2.407	3.008	0.601	0.000	0.000

UGP2	7.779	7.049	-0.729	0.000	0.000
UGT1A1	6.707	5.917	-0.790	0.000	0.000
UGT1A10	3.555	3.524	-0.031	0.697	0.709
UGT1A3	4.211	3.842	-0.368	0.000	0.000
UGT1A4	7.576	6.351	-1.225	0.000	0.000
UGT1A5	3.369	3.359	-0.010	0.854	0.860
UGT1A6	5.508	5.764	0.256	0.008	0.009
UGT1A7	3.301	3.249	-0.052	0.435	0.451
UGT1A8	5.076	4.531	-0.545	0.000	0.000
UGT1A9	5.215	4.970	-0.245	0.012	0.013
UGT2A1	0.492	0.979	0.488	0.000	0.000
UGT2A3	4.049	3.994	-0.055	0.532	0.547
UGT2B10	8.915	6.860	-2.055	0.000	0.000
UGT2B11	1.900	3.793	1.893	0.000	0.000
UGT2B15	8.185	7.163	-1.022	0.000	0.000
UGT2B17	1.802	1.582	-0.220	0.066	0.072
UGT2B4	9.213	9.202	-0.012	0.909	0.912
UGT2B7	9.532	7.120	-2.412	0.000	0.000
UGT3A1	3.967	3.148	-0.819	0.000	0.000
UGT3A2	0.539	0.824	0.285	0.000	0.000
UGT8	0.242	0.448	0.206	0.000	0.000
UHMK1	4.385	5.299	0.914	0.000	0.000
UHRF1BP1	2.013	2.784	0.771	0.000	0.000
UHRF1BP1L	3.345	3.774	0.429	0.000	0.000
UHRF2	2.716	3.161	0.444	0.000	0.000
UIMC1	2.880	3.579	0.699	0.000	0.000
ULBP1	0.189	0.366	0.177	0.000	0.000
ULBP2	0.616	1.134	0.518	0.000	0.000
ULBP3	0.340	0.369	0.029	0.287	0.302
ULK1	4.044	4.644	0.601	0.000	0.000
ULK2	1.408	1.443	0.035	0.442	0.458
ULK3	4.281	5.089	0.808	0.000	0.000
ULK4	1.072	1.874	0.802	0.000	0.000
UMPS	3.571	4.221	0.649	0.000	0.000
UNC119	2.803	3.724	0.921	0.000	0.000
UNC119B	2.266	3.244	0.978	0.000	0.000
UNC13B	3.490	4.410	0.920	0.000	0.000
UNC13D	2.560	2.096	-0.464	0.000	0.000
UNC45A	3.633	4.579	0.946	0.000	0.000
UNC50	4.518	5.186	0.668	0.000	0.000
UNC5A	0.217	0.358	0.141	0.000	0.000
UNC5B	1.589	2.173	0.583	0.000	0.000
UNC5C	0.642	0.524	-0.117	0.000	0.000
UNC93A	3.102	2.646	-0.455	0.000	0.000
UNC93B1	3.788	4.456	0.668	0.000	0.000
UNG	4.451	5.273	0.822	0.000	0.000
UNK	2.918	3.821	0.904	0.000	0.000
UNKL	1.998	2.844	0.847	0.000	0.000
UPB1	6.700	5.815	-0.885	0.000	0.000
UPF1	4.509	5.155	0.646	0.000	0.000
UPF2	3.858	4.313	0.455	0.000	0.000
UPF3A	3.571	4.176	0.604	0.000	0.000

UPF3B	3.265	4.116	0.851	0.000	0.000
UPK1A	0.206	0.591	0.385	0.000	0.000
UPK2	0.108	0.192	0.083	0.000	0.000
UPK3A	0.331	1.961	1.629	0.000	0.000
UPK3B	0.804	1.004	0.199	0.000	0.000
UPP1	3.383	3.645	0.261	0.000	0.000
UPP2	2.470	2.009	-0.461	0.000	0.000
UPRT	2.761	3.237	0.475	0.000	0.000
UQCR10	7.230	7.724	0.494	0.000	0.000
UQCR11	7.259	7.512	0.253	0.000	0.000
UQCRB	5.729	6.623	0.894	0.000	0.000
UQCRC1	7.439	7.744	0.304	0.000	0.000
UQCRC2	6.241	6.317	0.076	0.028	0.031
UQCRFS1	5.841	6.185	0.344	0.000	0.000
UQCRH	6.553	7.508	0.955	0.000	0.000
UQCRQ	8.606	8.974	0.368	0.000	0.000
URB1	2.173	2.754	0.581	0.000	0.000
URB2	1.612	2.401	0.789	0.000	0.000
URGCP	3.750	3.960	0.211	0.000	0.000
URGCP-MRPS	2.388	2.653	0.265	0.000	0.000
URM1	4.196	5.139	0.942	0.000	0.000
UROC1	6.317	3.454	-2.863	0.000	0.000
UROD	6.110	6.423	0.313	0.000	0.000
UROS	4.027	4.389	0.362	0.000	0.000
USE1	4.523	5.053	0.530	0.000	0.000
USF1	5.148	6.162	1.014	0.000	0.000
USH1C	1.191	1.625	0.434	0.000	0.000
USH2A	1.261	0.911	-0.349	0.000	0.000
USHBP1	1.211	1.498	0.287	0.000	0.000
USO1	5.637	5.647	0.010	0.801	0.809
USP1	3.226	4.181	0.956	0.000	0.000
USP10	4.404	4.708	0.304	0.000	0.000
USP11	3.631	4.333	0.702	0.000	0.000
USP12	4.083	4.090	0.007	0.852	0.858
USP13	2.235	2.705	0.470	0.000	0.000
USP14	3.898	4.912	1.014	0.000	0.000
USP15	3.425	3.323	-0.102	0.000	0.000
USP16	3.992	4.439	0.447	0.000	0.000
USP18	3.318	3.399	0.082	0.119	0.128
USP19	3.997	4.727	0.731	0.000	0.000
USP2	2.714	2.401	-0.312	0.000	0.000
USP20	3.508	3.973	0.465	0.000	0.000
USP21	2.811	3.971	1.160	0.000	0.000
USP22	4.450	5.326	0.876	0.000	0.000
USP24	3.301	3.845	0.544	0.000	0.000
USP25	3.127	3.488	0.361	0.000	0.000
USP28	2.653	3.176	0.523	0.000	0.000
USP30	3.352	3.899	0.547	0.000	0.000
USP31	1.926	2.615	0.690	0.000	0.000
USP32	3.330	3.723	0.393	0.000	0.000
USP33	3.703	4.238	0.536	0.000	0.000
USP34	3.211	3.672	0.461	0.000	0.000

USP35	2.684	3.085	0.401	0.000	0.000
USP36	3.232	3.873	0.642	0.000	0.000
USP37	1.509	1.925	0.416	0.000	0.000
USP38	3.352	3.193	-0.159	0.000	0.000
USP39	3.816	4.824	1.008	0.000	0.000
USP4	3.819	4.112	0.293	0.000	0.000
USP40	3.648	4.125	0.478	0.000	0.000
USP42	2.132	2.659	0.527	0.000	0.000
USP43	1.946	1.485	-0.461	0.000	0.000
USP45	1.613	2.001	0.388	0.000	0.000
USP46	1.410	2.080	0.670	0.000	0.000
USP47	4.088	4.434	0.346	0.000	0.000
USP48	3.324	3.752	0.429	0.000	0.000
USP49	0.591	1.004	0.412	0.000	0.000
USP5	4.749	5.635	0.886	0.000	0.000
USP50	0.674	0.778	0.105	0.000	0.000
USP51	0.866	0.889	0.023	0.505	0.520
USP53	2.331	2.132	-0.199	0.000	0.000
USP54	1.263	1.887	0.623	0.000	0.000
USP6NL	2.647	2.739	0.092	0.011	0.013
USP7	4.449	4.992	0.543	0.000	0.000
USP8	2.864	3.231	0.367	0.000	0.000
USP9X	4.388	4.706	0.318	0.000	0.000
USP9Y	1.647	1.462	-0.186	0.011	0.012
USPL1	3.123	3.247	0.124	0.001	0.001
UST	1.076	1.138	0.062	0.312	0.327
UTP14A	2.756	3.754	0.999	0.000	0.000
UTP14C	3.212	3.463	0.252	0.000	0.000
UTP15	2.122	2.884	0.762	0.000	0.000
UTP18	3.899	4.962	1.063	0.000	0.000
UTP20	1.852	2.506	0.653	0.000	0.000
UTP23	2.470	3.364	0.894	0.000	0.000
UTP3	4.715	4.811	0.096	0.002	0.002
UTP6	3.627	4.510	0.883	0.000	0.000
UTRN	2.903	3.519	0.616	0.000	0.000
UTS2	0.528	0.732	0.204	0.000	0.000
UTY	1.449	1.496	0.047	0.481	0.496
UVRAG	2.791	3.244	0.453	0.000	0.000
UXS1	2.722	4.057	1.335	0.000	0.000
VAC14	3.293	4.071	0.778	0.000	0.000
VAMP1	2.259	2.829	0.571	0.000	0.000
VAMP2	4.735	4.779	0.044	0.247	0.260
VAMP3	5.108	5.634	0.526	0.000	0.000
VAMP4	2.682	3.423	0.741	0.000	0.000
VAMP5	6.407	7.225	0.819	0.000	0.000
VAMP7	2.146	2.344	0.197	0.000	0.000
VAMP8	7.542	7.780	0.237	0.000	0.000
VANGL1	1.495	2.134	0.639	0.000	0.000
VANGL2	0.661	0.807	0.146	0.013	0.014
VAPA	4.657	5.317	0.659	0.000	0.000
VAPB	3.486	4.138	0.653	0.000	0.000
VARS	4.304	5.437	1.133	0.000	0.000

VARS2	3.653	4.138	0.485	0.000	0.000
VASH1	2.376	2.889	0.514	0.000	0.000
VASH2	0.280	0.939	0.659	0.000	0.000
VASN	4.666	5.060	0.394	0.000	0.000
VASP	4.855	5.468	0.614	0.000	0.000
VAT1	5.346	6.514	1.168	0.000	0.000
VAT1L	0.755	0.903	0.149	0.018	0.020
VAV1	2.273	2.047	-0.226	0.000	0.000
VAV2	4.671	5.018	0.347	0.000	0.000
VAV3	1.215	1.476	0.261	0.000	0.000
VAX2	0.228	0.633	0.405	0.000	0.000
VBP1	4.357	5.518	1.160	0.000	0.000
VCAM1	3.595	3.848	0.254	0.006	0.007
VCAN	1.352	2.220	0.868	0.000	0.000
VCL	3.497	3.786	0.289	0.000	0.000
VCP	6.175	7.038	0.863	0.000	0.000
VCPIP1	2.642	3.159	0.517	0.000	0.000
VDAC1	6.809	7.621	0.812	0.000	0.000
VDAC3	5.573	6.186	0.614	0.000	0.000
VDR	1.026	1.558	0.533	0.000	0.000
VEGFA	5.293	5.643	0.350	0.000	0.000
VEGFB	5.137	5.525	0.388	0.000	0.000
VEGFC	2.249	2.513	0.264	0.000	0.000
VENTX	0.564	0.667	0.103	0.001	0.001
VEPH1	0.734	0.656	-0.078	0.154	0.165
VEZF1	3.456	4.134	0.677	0.000	0.000
VEZT	3.420	3.961	0.542	0.000	0.000
VGLL3	0.490	0.586	0.096	0.008	0.009
VGLL4	2.603	3.489	0.886	0.000	0.000
VHL	3.737	4.465	0.728	0.000	0.000
VIL1	2.664	3.698	1.034	0.000	0.000
VILL	1.016	1.098	0.081	0.053	0.058
VIM	6.724	7.229	0.504	0.000	0.000
VIP	0.945	0.930	-0.015	0.704	0.716
VIPR1	3.673	1.203	-2.470	0.000	0.000
VIPR2	0.289	0.172	-0.117	0.000	0.000
VKORC1	6.274	6.221	-0.054	0.181	0.192
VKORC1L1	3.278	3.683	0.405	0.000	0.000
VLDLR	0.725	1.261	0.537	0.000	0.000
VMA21	3.184	4.012	0.829	0.000	0.000
VMAC	1.897	2.527	0.630	0.000	0.000
VMO1	4.069	3.085	-0.984	0.000	0.000
VMP1	5.422	6.147	0.725	0.000	0.000
VN1R1	0.456	0.883	0.427	0.000	0.000
VNN1	7.210	5.538	-1.672	0.000	0.000
VNN2	2.246	3.302	1.056	0.000	0.000
VNN3	3.984	3.261	-0.723	0.000	0.000
VOPP1	3.635	4.473	0.838	0.000	0.000
VPREB3	1.568	1.555	-0.013	0.803	0.811
VPS11	3.492	4.370	0.878	0.000	0.000
VPS13A	2.236	2.760	0.524	0.000	0.000
VPS13B	1.944	2.778	0.833	0.000	0.000

VPS13C	2.813	3.180	0.367	0.000	0.000
VPS13D	2.879	3.070	0.191	0.000	0.000
VPS16	4.057	4.900	0.842	0.000	0.000
VPS18	3.468	4.066	0.598	0.000	0.000
VPS25	5.018	6.013	0.995	0.000	0.000
VPS26A	4.303	5.080	0.778	0.000	0.000
VPS26B	4.198	4.756	0.558	0.000	0.000
VPS28	5.843	6.932	1.089	0.000	0.000
VPS29	5.352	6.135	0.783	0.000	0.000
VPS33A	2.136	3.223	1.088	0.000	0.000
VPS33B	2.611	3.336	0.725	0.000	0.000
VPS35	3.479	4.327	0.848	0.000	0.000
VPS36	4.014	4.314	0.300	0.000	0.000
VPS37A	3.347	3.789	0.442	0.000	0.000
VPS37B	2.954	2.860	-0.094	0.048	0.052
VPS37C	2.607	3.491	0.883	0.000	0.000
VPS37D	2.548	3.096	0.547	0.000	0.000
VPS39	3.543	4.223	0.680	0.000	0.000
VPS41	3.342	3.935	0.593	0.000	0.000
VPS45	3.102	4.320	1.218	0.000	0.000
VPS4A	4.184	4.569	0.385	0.000	0.000
VPS4B	4.084	4.458	0.374	0.000	0.000
VPS52	4.438	5.265	0.827	0.000	0.000
VPS53	1.579	1.961	0.382	0.000	0.000
VPS54	3.552	4.414	0.863	0.000	0.000
VPS72	3.402	4.893	1.491	0.000	0.000
VPS8	2.322	3.005	0.683	0.000	0.000
VRK1	2.111	3.186	1.075	0.000	0.000
VRK2	2.913	3.538	0.625	0.000	0.000
VRK3	3.341	3.702	0.361	0.000	0.000
VSIG1	0.236	0.752	0.516	0.000	0.000
VSIG10	2.392	3.560	1.168	0.000	0.000
VSIG10L	1.779	2.481	0.702	0.000	0.000
VSIG2	2.982	1.883	-1.099	0.000	0.000
VSIG4	4.366	3.208	-1.158	0.000	0.000
VSNL1	3.352	3.562	0.210	0.004	0.005
VSTM2L	1.827	1.617	-0.210	0.010	0.011
VSTM4	1.805	2.254	0.448	0.000	0.000
VSX1	0.102	0.265	0.163	0.000	0.000
VTA1	3.493	3.907	0.414	0.000	0.000
VTCN1	1.525	0.806	-0.718	0.000	0.000
VTI1A	1.947	2.492	0.545	0.000	0.000
VTI1B	4.705	4.953	0.248	0.000	0.000
VTN	12.216	11.882	-0.334	0.000	0.000
VWA1	4.908	5.693	0.785	0.000	0.000
VWA5A	2.011	2.072	0.061	0.247	0.260
VWA5B2	0.117	0.250	0.134	0.000	0.000
VWCE	3.169	2.938	-0.231	0.007	0.008
VWF	2.620	3.671	1.051	0.000	0.000
WAC	3.952	4.663	0.712	0.000	0.000
WARS	4.502	4.940	0.437	0.000	0.000
WARS2	2.578	3.286	0.708	0.000	0.000

WAS	3.294	3.186	-0.108	0.043	0.047
WASF1	2.327	3.261	0.934	0.000	0.000
WASF2	3.835	4.760	0.925	0.000	0.000
WASF3	2.172	1.893	-0.278	0.000	0.000
WASL	4.858	5.479	0.621	0.000	0.000
WBP1	4.244	4.977	0.733	0.000	0.000
WBP11	4.210	4.841	0.631	0.000	0.000
WBP2	6.009	6.382	0.374	0.000	0.000
WBP2NL	0.272	0.484	0.212	0.000	0.000
WBP4	3.752	3.924	0.172	0.000	0.000
WDFY1	3.656	4.170	0.513	0.000	0.000
WDFY2	1.656	1.718	0.062	0.034	0.038
WDFY3	2.054	2.215	0.161	0.000	0.000
WDFY4	1.188	1.088	-0.100	0.021	0.023
WDHD1	0.690	1.832	1.143	0.000	0.000
WDPCP	0.747	1.046	0.299	0.000	0.000
WDR1	5.403	5.998	0.595	0.000	0.000
WDR11	3.533	3.862	0.329	0.000	0.000
WDR12	2.638	3.614	0.977	0.000	0.000
WDR13	4.796	5.393	0.597	0.000	0.000
WDR17	0.290	0.165	-0.125	0.000	0.000
WDR18	4.907	5.317	0.410	0.000	0.000
WDR19	2.729	3.288	0.559	0.000	0.000
WDR20	2.402	2.775	0.373	0.000	0.000
WDR24	2.976	3.586	0.610	0.000	0.000
WDR25	2.250	2.645	0.396	0.000	0.000
WDR26	4.286	5.055	0.769	0.000	0.000
WDR27	1.800	2.092	0.292	0.000	0.000
WDR3	2.569	2.987	0.419	0.000	0.000
WDR31	0.964	1.215	0.251	0.000	0.000
WDR33	2.977	3.596	0.619	0.000	0.000
WDR34	4.812	5.132	0.321	0.000	0.000
WDR35	1.451	1.947	0.496	0.000	0.000
WDR36	3.298	3.567	0.269	0.000	0.000
WDR37	2.644	3.075	0.431	0.000	0.000
WDR4	2.178	3.069	0.890	0.000	0.000
WDR41	2.699	3.379	0.680	0.000	0.000
WDR43	3.826	4.491	0.665	0.000	0.000
WDR44	3.138	3.368	0.231	0.000	0.000
WDR45	4.606	5.148	0.542	0.000	0.000
WDR46	4.439	5.414	0.975	0.000	0.000
WDR47	1.781	2.189	0.408	0.000	0.000
WDR48	3.208	3.683	0.475	0.000	0.000
WDR5	4.091	5.047	0.956	0.000	0.000
WDR53	2.824	3.669	0.845	0.000	0.000
WDR54	1.644	2.177	0.533	0.000	0.000
WDR55	2.977	3.733	0.756	0.000	0.000
WDR59	3.974	4.172	0.199	0.000	0.000
WDR5B	2.311	2.963	0.652	0.000	0.000
WDR6	5.140	5.695	0.555	0.000	0.000
WDR60	2.412	2.679	0.267	0.000	0.000
WDR61	3.601	4.242	0.641	0.000	0.000

WDR62	0.468	1.626	1.157	0.000	0.000
WDR66	0.827	0.410	-0.416	0.000	0.000
WDR7	1.809	1.950	0.141	0.000	0.000
WDR70	2.794	3.532	0.738	0.000	0.000
WDR72	3.985	2.669	-1.316	0.000	0.000
WDR73	1.858	2.223	0.365	0.000	0.000
WDR75	3.531	4.269	0.738	0.000	0.000
WDR76	0.953	2.390	1.437	0.000	0.000
WDR77	3.833	4.527	0.695	0.000	0.000
WDR78	0.633	0.691	0.059	0.023	0.026
WDR81	3.865	4.056	0.191	0.000	0.000
WDR82	4.210	4.810	0.600	0.000	0.000
WDR83	3.196	3.900	0.704	0.000	0.000
WDR86	0.925	0.620	-0.305	0.000	0.000
WDR88	0.420	0.730	0.310	0.000	0.000
WDR89	3.009	3.374	0.365	0.000	0.000
WDR90	2.422	2.756	0.333	0.000	0.000
WDR91	2.894	3.622	0.728	0.000	0.000
WDR92	1.425	2.050	0.625	0.000	0.000
WDR93	0.136	0.173	0.037	0.001	0.001
WDSUB1	2.542	3.377	0.836	0.000	0.000
WDTC1	3.849	4.215	0.366	0.000	0.000
WDYHV1	2.538	3.860	1.322	0.000	0.000
WEE1	3.710	3.726	0.016	0.757	0.767
WEE2	0.134	0.159	0.025	0.002	0.002
WFDC1	1.157	0.752	-0.406	0.000	0.000
WFDC2	2.133	1.765	-0.368	0.001	0.001
WFDC3	0.341	0.452	0.111	0.000	0.000
WFIKKN1	0.576	0.659	0.083	0.008	0.009
WFS1	3.185	4.327	1.143	0.000	0.000
WHAMM	2.834	3.041	0.207	0.000	0.000
WIPF1	3.042	3.000	-0.042	0.486	0.502
WIPF2	2.819	3.535	0.715	0.000	0.000
WIPF3	0.873	1.250	0.377	0.000	0.000
WIPI1	3.462	4.188	0.726	0.000	0.000
WIPI2	4.043	4.835	0.792	0.000	0.000
WISP1	1.235	1.279	0.044	0.441	0.457
WISP2	0.989	0.706	-0.283	0.000	0.000
WISP3	0.387	0.258	-0.129	0.000	0.000
WIZ	3.023	3.896	0.873	0.000	0.000
WLS	3.448	3.264	-0.184	0.021	0.023
WNK1	3.914	4.702	0.788	0.000	0.000
WNK2	1.140	1.199	0.059	0.452	0.468
WNK3	1.673	2.070	0.397	0.000	0.000
WNK4	0.350	1.640	1.290	0.000	0.000
WNT10A	0.541	0.504	-0.037	0.363	0.379
WNT10B	0.574	0.509	-0.065	0.051	0.056
WNT11	2.941	1.855	-1.086	0.000	0.000
WNT16	0.165	0.195	0.030	0.034	0.038
WNT2	0.903	0.441	-0.462	0.000	0.000
WNT2B	0.295	0.400	0.105	0.000	0.000
WNT3	1.972	2.212	0.240	0.000	0.000

WNT4	1.343	1.395	0.052	0.477	0.493
WNT5A	1.303	1.845	0.543	0.000	0.000
WNT5B	1.905	1.837	-0.067	0.283	0.298
WNT6	0.230	0.484	0.254	0.000	0.000
WNT7B	0.396	0.329	-0.068	0.158	0.168
WNT9A	0.363	0.259	-0.104	0.000	0.000
WRAP53	1.834	2.361	0.527	0.000	0.000
WRAP73	2.122	2.845	0.723	0.000	0.000
WRB	3.335	4.012	0.677	0.000	0.000
WRN	1.972	2.249	0.277	0.000	0.000
WRNIP1	4.241	5.183	0.942	0.000	0.000
WSB1	4.442	4.570	0.128	0.016	0.018
WSB2	4.308	5.302	0.994	0.000	0.000
WSCD1	0.519	1.080	0.560	0.000	0.000
WTAP	4.134	4.665	0.530	0.000	0.000
WTIP	0.733	0.849	0.116	0.001	0.001
WWC1	3.142	3.612	0.470	0.000	0.000
WWC2	3.139	3.086	-0.053	0.220	0.233
WWC3	2.307	2.962	0.656	0.000	0.000
WWOX	2.628	2.862	0.233	0.000	0.000
WWP1	5.251	5.910	0.659	0.000	0.000
WWP2	3.001	3.685	0.684	0.000	0.000
WWTR1	3.349	3.666	0.318	0.000	0.000
XAB2	4.400	4.961	0.561	0.000	0.000
XAF1	4.213	3.922	-0.291	0.000	0.000
XAGE3	0.710	0.451	-0.259	0.000	0.000
XBP1	5.731	5.864	0.133	0.000	0.000
XCL1	1.174	1.308	0.134	0.020	0.023
XCL2	1.976	1.569	-0.407	0.000	0.000
XCR1	0.298	0.230	-0.068	0.001	0.001
XDH	5.379	4.062	-1.317	0.000	0.000
XG	0.646	0.527	-0.118	0.001	0.001
XIAP	3.610	4.107	0.496	0.000	0.000
XK	0.632	1.417	0.785	0.000	0.000
XKR6	0.195	0.201	0.006	0.698	0.710
XKR8	3.333	3.732	0.399	0.000	0.000
XKR9	0.811	1.124	0.313	0.000	0.000
XKRX	0.126	0.229	0.103	0.000	0.000
XPA	4.069	4.362	0.293	0.000	0.000
XPC	4.318	4.603	0.285	0.000	0.000
XPNPEP1	3.458	4.092	0.633	0.000	0.000
XPNPEP2	3.595	3.272	-0.324	0.030	0.033
XPNPEP3	2.659	3.015	0.356	0.000	0.000
XPO1	4.257	5.221	0.964	0.000	0.000
XPO4	2.069	2.555	0.486	0.000	0.000
XPO5	2.723	3.891	1.168	0.000	0.000
XPO6	4.126	4.778	0.652	0.000	0.000
XPO7	3.476	3.866	0.390	0.000	0.000
XPOT	3.655	4.780	1.125	0.000	0.000
XPR1	2.140	3.241	1.101	0.000	0.000
XRCC1	3.193	4.349	1.155	0.000	0.000
XRCC2	0.358	1.256	0.898	0.000	0.000

XRCC3	1.517	2.487	0.970	0.000	0.000
XRCC4	1.994	2.842	0.848	0.000	0.000
XRCC5	6.189	6.760	0.571	0.000	0.000
XRCC6	6.342	7.311	0.969	0.000	0.000
XRN1	3.182	3.292	0.110	0.005	0.006
XRN2	4.831	5.556	0.725	0.000	0.000
XRRA1	2.011	2.533	0.521	0.000	0.000
XYLB	3.894	4.092	0.198	0.000	0.000
XYLT1	0.509	0.597	0.087	0.004	0.004
XYLT2	2.919	3.903	0.984	0.000	0.000
YAF2	2.828	2.738	-0.090	0.001	0.001
YAP1	4.285	4.848	0.564	0.000	0.000
YARS	4.215	4.819	0.604	0.000	0.000
YARS2	3.314	3.905	0.590	0.000	0.000
YBEY	3.852	4.361	0.508	0.000	0.000
YBX1	7.788	8.474	0.686	0.000	0.000
YBX2	1.247	1.527	0.280	0.000	0.000
YDJC	3.497	4.419	0.922	0.000	0.000
YEATS2	1.826	2.825	0.999	0.000	0.000
YEATS4	3.397	4.300	0.903	0.000	0.000
YES1	4.638	4.896	0.258	0.000	0.000
YIF1A	6.272	6.825	0.553	0.000	0.000
YIF1B	4.265	5.454	1.189	0.000	0.000
YIPF1	4.796	5.592	0.797	0.000	0.000
YIPF2	4.638	5.354	0.716	0.000	0.000
YIPF3	5.865	6.909	1.044	0.000	0.000
YIPF4	3.466	3.807	0.341	0.000	0.000
YIPF5	4.188	4.721	0.533	0.000	0.000
YIPF6	3.683	4.300	0.616	0.000	0.000
YJEFN3	1.419	1.875	0.456	0.000	0.000
YKT6	3.987	5.189	1.203	0.000	0.000
YLPM1	2.651	3.075	0.424	0.000	0.000
YME1L1	4.862	5.490	0.627	0.000	0.000
YOD1	2.282	2.907	0.625	0.000	0.000
YPEL1	1.849	2.276	0.427	0.000	0.000
YPEL2	3.565	3.371	-0.194	0.000	0.000
YPEL3	4.738	5.260	0.523	0.000	0.000
YPEL4	0.414	0.507	0.094	0.000	0.000
YPEL5	5.456	5.970	0.513	0.000	0.000
YRDC	3.845	4.368	0.523	0.000	0.000
YTHDC1	4.310	4.484	0.174	0.000	0.000
YTHDC2	2.991	3.266	0.275	0.000	0.000
YTHDF1	4.719	5.393	0.674	0.000	0.000
YTHDF2	4.756	5.203	0.447	0.000	0.000
YTHDF3	4.458	5.153	0.695	0.000	0.000
YWHAB	6.019	6.822	0.803	0.000	0.000
YWHAE	7.167	7.454	0.287	0.000	0.000
YWHAG	5.634	6.577	0.943	0.000	0.000
YWHAH	5.482	6.575	1.093	0.000	0.000
YWHAQ	6.121	6.987	0.865	0.000	0.000
YWHAZ	5.707	6.945	1.238	0.000	0.000
YY1	4.245	4.764	0.519	0.000	0.000

YY1AP1	4.068	5.039	0.972	0.000	0.000
YY2	0.918	1.194	0.275	0.000	0.000
ZACN	1.474	2.087	0.612	0.000	0.000
ZADH2	3.524	3.355	-0.169	0.000	0.000
ZAP70	2.278	1.899	-0.380	0.000	0.000
ZBED1	2.186	2.194	0.007	0.718	0.730
ZBED2	0.242	0.507	0.266	0.000	0.000
ZBED3	2.276	2.790	0.514	0.000	0.000
ZBED4	2.080	2.853	0.772	0.000	0.000
ZBED5	2.387	3.136	0.749	0.000	0.000
ZBED6	1.763	1.930	0.167	0.010	0.012
ZBP1	0.967	0.802	-0.165	0.000	0.000
ZBTB1	3.401	3.710	0.309	0.000	0.000
ZBTB10	2.791	2.980	0.189	0.000	0.000
ZBTB11	2.692	3.174	0.482	0.000	0.000
ZBTB12	1.097	2.250	1.153	0.000	0.000
ZBTB16	2.492	2.144	-0.347	0.000	0.000
ZBTB17	3.348	3.953	0.605	0.000	0.000
ZBTB2	3.078	3.418	0.340	0.000	0.000
ZBTB20	1.116	1.093	-0.023	0.427	0.443
ZBTB22	3.159	4.152	0.993	0.000	0.000
ZBTB24	1.698	2.226	0.528	0.000	0.000
ZBTB25	1.334	1.605	0.271	0.000	0.000
ZBTB26	1.203	1.781	0.578	0.000	0.000
ZBTB3	1.468	1.992	0.524	0.000	0.000
ZBTB32	0.315	0.468	0.154	0.000	0.000
ZBTB33	3.559	4.385	0.826	0.000	0.000
ZBTB34	1.668	2.296	0.627	0.000	0.000
ZBTB37	0.967	1.250	0.283	0.000	0.000
ZBTB38	3.059	3.588	0.529	0.000	0.000
ZBTB39	1.621	2.106	0.485	0.000	0.000
ZBTB4	3.293	3.562	0.269	0.000	0.000
ZBTB40	2.232	2.880	0.647	0.000	0.000
ZBTB41	2.054	3.111	1.057	0.000	0.000
ZBTB42	2.167	2.834	0.667	0.000	0.000
ZBTB43	2.559	2.648	0.089	0.028	0.031
ZBTB44	3.107	3.537	0.430	0.000	0.000
ZBTB45	2.011	2.949	0.938	0.000	0.000
ZBTB46	2.192	2.410	0.218	0.000	0.000
ZBTB47	2.357	2.705	0.348	0.000	0.000
ZBTB48	4.090	4.155	0.064	0.077	0.084
ZBTB49	1.877	2.185	0.307	0.000	0.000
ZBTB5	3.050	3.437	0.387	0.000	0.000
ZBTB6	2.028	2.611	0.583	0.000	0.000
ZBTB7A	3.751	4.043	0.291	0.000	0.000
ZBTB7B	4.767	5.599	0.832	0.000	0.000
ZBTB7C	0.602	0.670	0.068	0.061	0.066
ZBTB8A	1.141	1.521	0.380	0.000	0.000
ZBTB8OS	3.470	4.113	0.643	0.000	0.000
ZBTB9	1.162	1.978	0.817	0.000	0.000
ZC3H10	1.327	1.963	0.636	0.000	0.000
ZC3H11A	4.726	5.291	0.565	0.000	0.000

ZC3H12A	4.032	3.795	-0.237	0.000	0.000
ZC3H12B	0.562	0.710	0.148	0.000	0.000
ZC3H12C	1.713	1.790	0.077	0.029	0.032
ZC3H12D	0.748	0.852	0.104	0.002	0.002
ZC3H13	4.444	4.045	-0.399	0.000	0.000
ZC3H14	3.060	3.188	0.127	0.000	0.000
ZC3H15	5.367	5.877	0.510	0.000	0.000
ZC3H18	3.137	3.571	0.434	0.000	0.000
ZC3H3	3.443	4.612	1.169	0.000	0.000
ZC3H4	3.008	3.535	0.527	0.000	0.000
ZC3H6	1.793	1.906	0.113	0.002	0.002
ZC3H7A	3.774	4.136	0.362	0.000	0.000
ZC3H7B	4.245	4.618	0.373	0.000	0.000
ZC3H8	1.703	2.351	0.648	0.000	0.000
ZC3HAV1	3.042	3.677	0.635	0.000	0.000
ZC3HAV1L	0.683	0.890	0.208	0.000	0.000
ZC3HC1	3.426	4.174	0.748	0.000	0.000
ZC4H2	1.947	2.262	0.314	0.000	0.000
ZCCHC10	3.479	4.107	0.628	0.000	0.000
ZCCHC14	3.855	4.019	0.164	0.000	0.000
ZCCHC17	3.850	4.703	0.853	0.000	0.000
ZCCHC18	0.243	0.321	0.078	0.000	0.000
ZCCHC2	3.512	3.484	-0.029	0.449	0.465
ZCCHC24	4.526	4.168	-0.358	0.000	0.000
ZCCHC3	2.815	3.559	0.744	0.000	0.000
ZCCHC4	1.898	2.488	0.590	0.000	0.000
ZCCHC7	2.892	3.608	0.716	0.000	0.000
ZCCHC8	3.024	3.443	0.419	0.000	0.000
ZCCHC9	3.733	4.599	0.867	0.000	0.000
ZCRB1	4.866	5.541	0.675	0.000	0.000
ZCWPW1	2.703	2.539	-0.164	0.000	0.000
ZCWPW2	0.638	0.707	0.069	0.000	0.000
ZDBF2	1.183	0.902	-0.282	0.000	0.000
ZDHHC1	2.083	1.983	-0.100	0.060	0.066
ZDHHC11	1.721	1.800	0.079	0.157	0.168
ZDHHC12	4.262	5.150	0.888	0.000	0.000
ZDHHC13	1.090	1.597	0.507	0.000	0.000
ZDHHC14	2.459	2.896	0.437	0.000	0.000
ZDHHC15	0.183	0.193	0.011	0.534	0.548
ZDHHC16	4.096	4.674	0.578	0.000	0.000
ZDHHC17	2.673	3.067	0.394	0.000	0.000
ZDHHC18	3.522	4.042	0.520	0.000	0.000
ZDHHC19	1.611	1.258	-0.353	0.000	0.000
ZDHHC2	1.828	2.068	0.241	0.000	0.000
ZDHHC20	2.867	3.392	0.524	0.000	0.000
ZDHHC21	1.823	1.988	0.165	0.000	0.000
ZDHHC23	1.895	2.423	0.528	0.000	0.000
ZDHHC24	1.968	2.807	0.839	0.000	0.000
ZDHHC3	3.649	4.318	0.669	0.000	0.000
ZDHHC4	4.586	4.844	0.258	0.000	0.000
ZDHHC5	4.723	5.331	0.609	0.000	0.000
ZDHHC6	4.161	4.799	0.638	0.000	0.000

ZDHHC7	3.802	4.193	0.391	0.000	0.000
ZDHHC8	3.387	3.522	0.134	0.019	0.022
ZDHHC9	4.438	5.461	1.023	0.000	0.000
ZEB1	2.627	3.073	0.446	0.000	0.000
ZEB2	2.297	1.853	-0.444	0.000	0.000
ZER1	4.070	4.219	0.150	0.000	0.000
ZFAND1	3.604	4.284	0.680	0.000	0.000
ZFAND2A	5.149	5.799	0.650	0.000	0.000
ZFAND2B	4.671	5.298	0.627	0.000	0.000
ZFAND3	4.902	5.743	0.841	0.000	0.000
ZFAND5	6.500	6.503	0.004	0.951	0.953
ZFAND6	5.159	5.493	0.334	0.000	0.000
ZFAT	1.156	1.964	0.808	0.000	0.000
ZFC3H1	2.606	3.309	0.703	0.000	0.000
ZFHX2	0.269	0.433	0.164	0.000	0.000
ZFHX3	1.849	2.261	0.411	0.000	0.000
ZFHX4	1.656	1.793	0.137	0.001	0.001
ZFP1	3.452	2.618	-0.834	0.000	0.000
ZFP14	1.310	1.930	0.621	0.000	0.000
ZFP2	0.814	0.980	0.167	0.000	0.000
ZFP28	0.738	0.832	0.094	0.006	0.007
ZFP3	1.267	1.009	-0.257	0.000	0.000
ZFP30	1.116	1.448	0.332	0.000	0.000
ZFP36	8.313	7.045	-1.268	0.000	0.000
ZFP36L1	7.186	6.978	-0.208	0.000	0.000
ZFP36L2	6.100	6.407	0.307	0.000	0.000
ZFP37	0.447	0.740	0.293	0.000	0.000
ZFP41	1.293	2.579	1.286	0.000	0.000
ZFP57	0.336	0.477	0.141	0.001	0.002
ZFP62	2.681	3.732	1.051	0.000	0.000
ZFP64	1.737	2.686	0.949	0.000	0.000
ZFP82	0.582	0.866	0.284	0.000	0.000
ZFP90	2.050	2.548	0.498	0.000	0.000
ZFP91	4.195	4.802	0.607	0.000	0.000
ZFP92	0.260	0.306	0.046	0.047	0.052
ZFPM1	2.518	2.726	0.209	0.000	0.000
ZFPM2	1.140	0.792	-0.348	0.000	0.000
ZFR	4.117	4.916	0.798	0.000	0.000
ZFX	2.767	3.308	0.541	0.000	0.000
ZFY	1.688	1.653	-0.036	0.625	0.639
ZFYVE1	3.032	3.431	0.399	0.000	0.000
ZFYVE16	2.878	3.328	0.450	0.000	0.000
ZFYVE19	3.929	4.492	0.563	0.000	0.000
ZFYVE21	3.350	3.469	0.118	0.000	0.000
ZFYVE26	1.770	2.580	0.810	0.000	0.000
ZFYVE27	4.050	4.465	0.415	0.000	0.000
ZFYVE28	1.790	1.619	-0.171	0.000	0.000
ZFYVE9	2.591	3.161	0.570	0.000	0.000
ZG16	4.171	2.392	-1.779	0.000	0.000
ZG16B	0.667	0.882	0.216	0.002	0.002
ZGLP1	1.528	1.713	0.185	0.000	0.000
ZGPAT	5.202	4.369	-0.833	0.000	0.000

ZHX1	3.985	4.816	0.831	0.000	0.000
ZHX2	3.738	4.466	0.728	0.000	0.000
ZHX3	2.913	3.208	0.296	0.000	0.000
ZIC1	0.954	1.693	0.739	0.000	0.000
ZIC2	0.165	2.032	1.867	0.000	0.000
ZIC4	0.120	0.677	0.557	0.000	0.000
ZIC5	0.093	1.004	0.911	0.000	0.000
ZIK1	0.525	0.731	0.206	0.000	0.000
ZKSCAN1	4.568	4.868	0.301	0.000	0.000
ZKSCAN2	1.231	1.423	0.192	0.000	0.000
ZKSCAN3	1.427	2.629	1.203	0.000	0.000
ZKSCAN4	2.008	2.741	0.733	0.000	0.000
ZKSCAN5	1.941	2.867	0.927	0.000	0.000
ZMAT1	2.607	2.553	-0.054	0.355	0.371
ZMAT2	5.834	6.648	0.814	0.000	0.000
ZMAT3	2.947	3.058	0.111	0.006	0.006
ZMAT5	3.365	4.239	0.874	0.000	0.000
ZMIZ1	2.934	3.630	0.696	0.000	0.000
ZMIZ2	3.954	4.797	0.842	0.000	0.000
ZMPSTE24	4.650	5.590	0.940	0.000	0.000
ZMYM1	1.807	2.527	0.720	0.000	0.000
ZMYM2	3.117	3.478	0.361	0.000	0.000
ZMYM3	3.047	3.857	0.810	0.000	0.000
ZMYM4	3.097	3.674	0.577	0.000	0.000
ZMYM5	2.654	3.012	0.358	0.000	0.000
ZMYM6	2.043	2.415	0.372	0.000	0.000
ZMYND10	0.590	0.985	0.395	0.000	0.000
ZMYND11	4.307	4.849	0.542	0.000	0.000
ZMYND12	2.341	1.930	-0.411	0.000	0.000
ZMYND15	2.150	2.406	0.256	0.000	0.000
ZMYND19	3.314	4.334	1.019	0.000	0.000
ZMYND8	2.865	3.454	0.589	0.000	0.000
ZNF10	2.244	2.418	0.175	0.000	0.000
ZNF100	1.044	1.386	0.342	0.000	0.000
ZNF101	1.450	2.097	0.647	0.000	0.000
ZNF107	1.062	1.677	0.615	0.000	0.000
ZNF114	0.120	0.194	0.075	0.000	0.000
ZNF117	1.457	1.921	0.465	0.000	0.000
ZNF12	3.028	3.668	0.640	0.000	0.000
ZNF121	2.364	2.607	0.243	0.000	0.000
ZNF124	1.029	1.545	0.516	0.000	0.000
ZNF131	2.551	3.091	0.540	0.000	0.000
ZNF132	1.059	1.196	0.136	0.000	0.000
ZNF133	2.388	3.038	0.649	0.000	0.000
ZNF134	1.941	2.384	0.442	0.000	0.000
ZNF135	0.635	0.789	0.154	0.000	0.000
ZNF136	1.691	1.964	0.272	0.000	0.000
ZNF138	2.002	2.802	0.800	0.000	0.000
ZNF14	1.188	1.566	0.378	0.000	0.000
ZNF140	2.671	3.020	0.349	0.000	0.000
ZNF141	1.399	1.411	0.012	0.794	0.803
ZNF142	1.796	2.635	0.838	0.000	0.000

ZNF143	2.574	3.398	0.824	0.000	0.000
ZNF146	4.448	5.262	0.814	0.000	0.000
ZNF148	2.790	3.317	0.527	0.000	0.000
ZNF154	0.768	0.791	0.023	0.451	0.467
ZNF155	1.812	2.108	0.296	0.000	0.000
ZNF16	1.751	2.654	0.904	0.000	0.000
ZNF160	2.485	2.300	-0.185	0.001	0.001
ZNF165	1.643	2.456	0.813	0.000	0.000
ZNF169	0.657	1.029	0.372	0.000	0.000
ZNF17	1.459	1.909	0.450	0.000	0.000
ZNF174	2.040	2.981	0.940	0.000	0.000
ZNF175	1.296	1.406	0.110	0.001	0.002
ZNF177	0.866	1.020	0.155	0.000	0.000
ZNF18	2.911	3.016	0.105	0.002	0.002
ZNF180	1.860	2.327	0.468	0.000	0.000
ZNF181	2.586	2.985	0.399	0.000	0.000
ZNF182	2.170	2.716	0.546	0.000	0.000
ZNF184	1.771	2.490	0.719	0.000	0.000
ZNF185	1.305	1.605	0.300	0.000	0.000
ZNF189	3.592	4.459	0.867	0.000	0.000
ZNF19	0.747	0.987	0.240	0.000	0.000
ZNF195	2.529	3.210	0.681	0.000	0.000
ZNF197	2.290	2.568	0.277	0.000	0.000
ZNF20	0.668	0.980	0.312	0.000	0.000
ZNF200	1.504	2.291	0.788	0.000	0.000
ZNF202	2.061	2.627	0.566	0.000	0.000
ZNF205	3.086	3.861	0.776	0.000	0.000
ZNF207	3.625	4.549	0.924	0.000	0.000
ZNF208	0.383	0.724	0.341	0.000	0.000
ZNF211	2.401	2.761	0.360	0.000	0.000
ZNF212	2.912	3.634	0.722	0.000	0.000
ZNF213	1.256	1.953	0.697	0.000	0.000
ZNF214	0.973	1.139	0.166	0.000	0.000
ZNF215	0.533	0.629	0.096	0.015	0.017
ZNF217	3.417	4.198	0.781	0.000	0.000
ZNF219	2.464	3.770	1.306	0.000	0.000
ZNF22	4.554	4.986	0.432	0.000	0.000
ZNF221	0.533	0.850	0.317	0.000	0.000
ZNF222	1.609	2.132	0.523	0.000	0.000
ZNF223	1.050	1.420	0.369	0.000	0.000
ZNF225	0.988	1.375	0.387	0.000	0.000
ZNF226	2.114	2.630	0.515	0.000	0.000
ZNF227	2.111	2.663	0.552	0.000	0.000
ZNF23	1.374	1.702	0.328	0.000	0.000
ZNF230	1.081	1.432	0.351	0.000	0.000
ZNF232	2.212	2.967	0.755	0.000	0.000
ZNF233	0.592	0.960	0.368	0.000	0.000
ZNF234	1.596	2.157	0.561	0.000	0.000
ZNF235	1.145	1.406	0.262	0.000	0.000
ZNF236	1.629	2.056	0.427	0.000	0.000
ZNF239	0.979	1.485	0.506	0.000	0.000
ZNF24	4.181	4.499	0.319	0.000	0.000

ZNF248	1.754	2.377	0.624	0.000	0.000
ZNF25	2.038	2.321	0.284	0.000	0.000
ZNF250	1.409	2.096	0.687	0.000	0.000
ZNF251	2.409	3.520	1.111	0.000	0.000
ZNF253	1.773	2.448	0.675	0.000	0.000
ZNF254	2.050	2.330	0.281	0.000	0.000
ZNF256	1.521	1.946	0.425	0.000	0.000
ZNF257	0.309	0.666	0.357	0.000	0.000
ZNF26	1.097	1.582	0.485	0.000	0.000
ZNF260	2.191	2.974	0.783	0.000	0.000
ZNF263	3.049	3.746	0.697	0.000	0.000
ZNF264	1.927	2.071	0.144	0.000	0.000
ZNF266	3.195	3.586	0.390	0.000	0.000
ZNF267	1.786	2.272	0.485	0.000	0.000
ZNF268	1.985	2.236	0.251	0.000	0.000
ZNF273	0.479	0.951	0.472	0.000	0.000
ZNF274	3.177	3.339	0.162	0.000	0.000
ZNF275	3.189	3.345	0.156	0.000	0.000
ZNF276	3.110	3.312	0.202	0.000	0.000
ZNF277	3.725	4.368	0.643	0.000	0.000
ZNF28	1.557	2.325	0.768	0.000	0.000
ZNF280C	1.039	1.580	0.541	0.000	0.000
ZNF280D	2.531	2.994	0.463	0.000	0.000
ZNF281	3.729	4.240	0.511	0.000	0.000
ZNF282	3.244	4.303	1.059	0.000	0.000
ZNF283	0.644	1.031	0.387	0.000	0.000
ZNF284	0.670	1.028	0.358	0.000	0.000
ZNF285	0.449	0.795	0.346	0.000	0.000
ZNF286A	0.791	1.306	0.515	0.000	0.000
ZNF286B	0.201	0.397	0.196	0.000	0.000
ZNF287	0.582	0.862	0.280	0.000	0.000
ZNF292	2.242	2.699	0.457	0.000	0.000
ZNF296	0.682	1.458	0.775	0.000	0.000
ZNF3	3.362	4.205	0.842	0.000	0.000
ZNF30	1.713	2.486	0.773	0.000	0.000
ZNF300	0.848	1.708	0.860	0.000	0.000
ZNF302	3.518	4.190	0.672	0.000	0.000
ZNF304	1.927	2.375	0.448	0.000	0.000
ZNF311	0.846	0.909	0.063	0.147	0.157
ZNF317	3.293	3.783	0.491	0.000	0.000
ZNF318	2.498	3.418	0.920	0.000	0.000
ZNF319	1.922	2.531	0.609	0.000	0.000
ZNF32	4.758	5.325	0.567	0.000	0.000
ZNF320	1.241	1.971	0.730	0.000	0.000
ZNF324	1.791	2.157	0.366	0.000	0.000
ZNF324B	1.167	1.662	0.495	0.000	0.000
ZNF326	2.679	2.884	0.205	0.000	0.000
ZNF329	2.464	2.986	0.522	0.000	0.000
ZNF330	5.108	4.758	-0.350	0.000	0.000
ZNF331	2.612	2.995	0.383	0.000	0.000
ZNF333	1.204	1.652	0.448	0.000	0.000
ZNF334	1.190	0.896	-0.294	0.000	0.000

ZNF335	2.586	3.341	0.755	0.000	0.000
ZNF33A	3.381	3.913	0.532	0.000	0.000
ZNF33B	2.637	2.965	0.328	0.000	0.000
ZNF34	2.297	3.043	0.747	0.000	0.000
ZNF341	1.199	1.970	0.771	0.000	0.000
ZNF343	2.200	2.856	0.656	0.000	0.000
ZNF345	1.218	1.633	0.415	0.000	0.000
ZNF346	1.879	2.638	0.759	0.000	0.000
ZNF347	0.806	0.883	0.077	0.030	0.033
ZNF35	1.546	2.053	0.507	0.000	0.000
ZNF350	1.862	2.288	0.426	0.000	0.000
ZNF354A	2.003	2.704	0.701	0.000	0.000
ZNF354B	1.940	2.421	0.481	0.000	0.000
ZNF354C	0.724	0.782	0.058	0.126	0.136
ZNF362	2.983	3.733	0.749	0.000	0.000
ZNF366	0.566	0.695	0.129	0.000	0.000
ZNF367	2.597	2.976	0.379	0.000	0.000
ZNF37A	2.085	2.586	0.500	0.000	0.000
ZNF382	0.337	0.620	0.283	0.000	0.000
ZNF383	1.165	1.524	0.359	0.000	0.000
ZNF384	3.012	3.729	0.717	0.000	0.000
ZNF385A	2.937	3.321	0.384	0.000	0.000
ZNF385B	3.095	2.351	-0.744	0.000	0.000
ZNF385C	0.326	0.869	0.542	0.000	0.000
ZNF385D	0.212	0.512	0.300	0.000	0.000
ZNF391	0.275	0.605	0.331	0.000	0.000
ZNF394	3.369	3.927	0.559	0.000	0.000
ZNF395	4.110	3.944	-0.166	0.000	0.000
ZNF396	0.644	0.960	0.316	0.000	0.000
ZNF397	1.992	2.456	0.464	0.000	0.000
ZNF398	1.955	2.676	0.721	0.000	0.000
ZNF404	1.107	1.656	0.549	0.000	0.000
ZNF407	1.336	1.832	0.497	0.000	0.000
ZNF408	3.222	3.793	0.572	0.000	0.000
ZNF41	1.945	2.226	0.282	0.000	0.000
ZNF410	2.183	2.559	0.376	0.000	0.000
ZNF414	2.895	3.563	0.668	0.000	0.000
ZNF415	0.925	0.974	0.049	0.280	0.295
ZNF416	1.398	2.014	0.616	0.000	0.000
ZNF417	1.774	1.972	0.198	0.000	0.000
ZNF418	1.203	1.390	0.187	0.000	0.000
ZNF419	1.397	1.914	0.517	0.000	0.000
ZNF420	1.763	2.293	0.530	0.000	0.000
ZNF423	0.452	0.599	0.146	0.000	0.000
ZNF425	0.851	1.208	0.357	0.000	0.000
ZNF426	0.946	1.216	0.270	0.000	0.000
ZNF428	4.631	5.291	0.660	0.000	0.000
ZNF429	1.890	2.017	0.127	0.000	0.000
ZNF43	0.811	1.316	0.505	0.000	0.000
ZNF430	0.869	1.127	0.258	0.000	0.000
ZNF431	0.660	1.118	0.458	0.000	0.000
ZNF432	1.358	2.065	0.707	0.000	0.000

ZNF433	1.713	2.053	0.340	0.000	0.000
ZNF436	2.215	2.802	0.586	0.000	0.000
ZNF438	2.209	2.503	0.294	0.000	0.000
ZNF439	0.901	0.939	0.038	0.318	0.333
ZNF44	2.608	2.993	0.386	0.000	0.000
ZNF440	1.486	1.924	0.438	0.000	0.000
ZNF441	1.903	2.147	0.244	0.000	0.000
ZNF442	0.719	0.995	0.277	0.000	0.000
ZNF443	1.790	2.111	0.321	0.000	0.000
ZNF444	4.245	4.718	0.472	0.000	0.000
ZNF445	2.045	2.561	0.515	0.000	0.000
ZNF446	1.987	2.574	0.588	0.000	0.000
ZNF449	1.428	2.044	0.616	0.000	0.000
ZNF45	2.053	2.588	0.536	0.000	0.000
ZNF451	2.503	2.952	0.449	0.000	0.000
ZNF454	0.242	0.361	0.120	0.000	0.000
ZNF460	0.993	1.007	0.014	0.659	0.672
ZNF461	1.227	1.857	0.630	0.000	0.000
ZNF462	0.704	0.598	-0.106	0.001	0.001
ZNF467	2.789	3.321	0.532	0.000	0.000
ZNF468	1.543	2.352	0.809	0.000	0.000
ZNF469	0.355	0.413	0.058	0.051	0.056
ZNF470	1.380	1.275	-0.105	0.004	0.004
ZNF471	1.104	1.024	-0.079	0.084	0.091
ZNF473	1.552	2.312	0.760	0.000	0.000
ZNF474	0.128	0.221	0.093	0.000	0.000
ZNF48	2.034	2.824	0.790	0.000	0.000
ZNF480	2.412	3.111	0.699	0.000	0.000
ZNF483	0.390	0.364	-0.027	0.313	0.328
ZNF484	1.242	1.408	0.165	0.000	0.000
ZNF485	1.303	1.911	0.607	0.000	0.000
ZNF486	0.514	0.793	0.280	0.000	0.000
ZNF490	0.641	1.103	0.462	0.000	0.000
ZNF491	0.839	1.256	0.417	0.000	0.000
ZNF493	1.107	1.441	0.334	0.000	0.000
ZNF496	1.657	2.160	0.503	0.000	0.000
ZNF497	1.248	1.705	0.457	0.000	0.000
ZNF500	1.620	2.223	0.603	0.000	0.000
ZNF501	0.662	1.044	0.383	0.000	0.000
ZNF502	1.518	1.510	-0.008	0.877	0.882
ZNF503	2.929	3.545	0.616	0.000	0.000
ZNF506	1.536	1.955	0.420	0.000	0.000
ZNF507	2.141	2.664	0.523	0.000	0.000
ZNF510	1.796	2.181	0.385	0.000	0.000
ZNF512	2.800	3.808	1.008	0.000	0.000
ZNF512B	3.579	4.321	0.743	0.000	0.000
ZNF513	4.368	4.821	0.453	0.000	0.000
ZNF514	1.930	2.489	0.560	0.000	0.000
ZNF516	2.499	3.087	0.588	0.000	0.000
ZNF517	1.609	2.921	1.312	0.000	0.000
ZNF518A	2.353	2.909	0.556	0.000	0.000
ZNF518B	1.009	1.089	0.080	0.054	0.059

ZNF519	0.192	0.514	0.322	0.000	0.000
ZNF521	0.763	0.956	0.194	0.000	0.000
ZNF524	4.233	4.901	0.668	0.000	0.000
ZNF525	0.999	1.496	0.497	0.000	0.000
ZNF526	1.793	2.672	0.880	0.000	0.000
ZNF527	1.207	1.684	0.476	0.000	0.000
ZNF528	1.192	1.513	0.321	0.000	0.000
ZNF529	1.950	2.429	0.479	0.000	0.000
ZNF530	0.689	1.380	0.690	0.000	0.000
ZNF532	1.446	2.181	0.736	0.000	0.000
ZNF540	1.264	1.234	-0.030	0.345	0.360
ZNF541	0.319	0.778	0.459	0.000	0.000
ZNF543	1.057	1.585	0.528	0.000	0.000
ZNF544	2.196	3.224	1.028	0.000	0.000
ZNF546	1.071	1.398	0.327	0.000	0.000
ZNF547	0.995	1.457	0.462	0.000	0.000
ZNF548	2.357	2.766	0.408	0.000	0.000
ZNF549	0.838	0.947	0.108	0.002	0.002
ZNF550	1.193	1.729	0.536	0.000	0.000
ZNF551	1.049	1.461	0.413	0.000	0.000
ZNF552	1.915	2.816	0.902	0.000	0.000
ZNF554	1.602	2.131	0.529	0.000	0.000
ZNF555	0.866	1.461	0.595	0.000	0.000
ZNF557	1.408	1.845	0.437	0.000	0.000
ZNF558	2.170	2.682	0.512	0.000	0.000
ZNF559	2.426	2.632	0.207	0.000	0.000
ZNF561	3.000	3.356	0.356	0.000	0.000
ZNF562	1.821	2.280	0.459	0.000	0.000
ZNF563	2.343	3.340	0.997	0.000	0.000
ZNF564	1.477	1.932	0.455	0.000	0.000
ZNF565	1.314	1.793	0.479	0.000	0.000
ZNF566	1.812	2.191	0.379	0.000	0.000
ZNF567	1.543	2.027	0.484	0.000	0.000
ZNF568	0.972	1.319	0.347	0.000	0.000
ZNF569	1.495	1.918	0.422	0.000	0.000
ZNF57	1.612	2.436	0.823	0.000	0.000
ZNF570	0.871	1.155	0.284	0.000	0.000
ZNF571	0.933	1.205	0.272	0.000	0.000
ZNF572	0.830	1.615	0.786	0.000	0.000
ZNF573	0.649	0.891	0.242	0.000	0.000
ZNF574	2.340	3.042	0.702	0.000	0.000
ZNF575	1.056	1.390	0.335	0.000	0.000
ZNF576	2.811	3.433	0.622	0.000	0.000
ZNF577	1.523	1.800	0.277	0.000	0.000
ZNF578	0.133	0.313	0.180	0.000	0.000
ZNF579	2.385	3.255	0.869	0.000	0.000
ZNF580	2.639	3.560	0.921	0.000	0.000
ZNF581	3.078	4.228	1.149	0.000	0.000
ZNF582	1.314	1.375	0.062	0.122	0.131
ZNF583	0.891	1.236	0.345	0.000	0.000
ZNF584	2.348	2.692	0.344	0.000	0.000
ZNF585A	1.128	1.665	0.537	0.000	0.000

ZNF585B	1.497	1.931	0.434	0.000	0.000
ZNF586	1.832	2.041	0.209	0.000	0.000
ZNF587	2.082	2.739	0.657	0.000	0.000
ZNF589	1.565	2.169	0.604	0.000	0.000
ZNF592	3.307	3.749	0.442	0.000	0.000
ZNF594	1.004	1.177	0.173	0.000	0.000
ZNF596	1.256	1.337	0.081	0.015	0.017
ZNF597	0.963	1.056	0.093	0.001	0.001
ZNF598	3.931	4.473	0.542	0.000	0.000
ZNF599	0.993	1.414	0.420	0.000	0.000
ZNF600	1.711	2.298	0.587	0.000	0.000
ZNF605	1.488	2.278	0.789	0.000	0.000
ZNF606	1.608	2.125	0.517	0.000	0.000
ZNF607	0.829	1.394	0.565	0.000	0.000
ZNF608	1.672	2.139	0.467	0.000	0.000
ZNF609	2.454	2.915	0.461	0.000	0.000
ZNF610	0.390	0.716	0.326	0.000	0.000
ZNF611	1.106	1.440	0.333	0.000	0.000
ZNF613	1.353	1.791	0.437	0.000	0.000
ZNF614	1.289	1.676	0.387	0.000	0.000
ZNF615	1.763	2.063	0.300	0.000	0.000
ZNF616	1.667	2.228	0.561	0.000	0.000
ZNF618	1.864	2.558	0.694	0.000	0.000
ZNF619	1.204	1.596	0.392	0.000	0.000
ZNF620	1.000	1.471	0.471	0.000	0.000
ZNF621	2.130	2.650	0.520	0.000	0.000
ZNF622	4.780	5.553	0.772	0.000	0.000
ZNF623	1.909	3.002	1.092	0.000	0.000
ZNF624	0.744	1.115	0.372	0.000	0.000
ZNF625	0.475	0.602	0.127	0.000	0.000
ZNF626	0.886	1.065	0.179	0.000	0.000
ZNF627	2.366	2.958	0.592	0.000	0.000
ZNF628	1.607	2.393	0.786	0.000	0.000
ZNF629	2.124	2.616	0.492	0.000	0.000
ZNF630	0.762	0.816	0.054	0.143	0.153
ZNF638	4.600	4.910	0.310	0.000	0.000
ZNF639	2.884	3.524	0.640	0.000	0.000
ZNF641	2.281	2.603	0.323	0.000	0.000
ZNF644	3.192	3.615	0.423	0.000	0.000
ZNF646	1.587	2.341	0.754	0.000	0.000
ZNF648	0.542	0.960	0.418	0.000	0.000
ZNF649	1.527	1.687	0.160	0.000	0.000
ZNF652	3.403	4.128	0.725	0.000	0.000
ZNF653	2.156	2.466	0.309	0.000	0.000
ZNF654	2.606	2.755	0.149	0.000	0.000
ZNF655	3.401	4.022	0.621	0.000	0.000
ZNF660	0.276	0.362	0.085	0.000	0.000
ZNF662	1.764	1.557	-0.207	0.000	0.000
ZNF664	4.527	5.398	0.871	0.000	0.000
ZNF665	0.222	0.386	0.164	0.000	0.000
ZNF667	0.637	0.595	-0.042	0.228	0.241
ZNF669	1.383	2.268	0.885	0.000	0.000

ZNF670	1.638	2.266	0.628	0.000	0.000
ZNF671	1.711	1.966	0.255	0.000	0.000
ZNF672	3.954	4.814	0.861	0.000	0.000
ZNF674	1.111	1.463	0.352	0.000	0.000
ZNF675	0.784	1.436	0.652	0.000	0.000
ZNF676	0.305	0.687	0.382	0.000	0.000
ZNF677	0.570	0.619	0.050	0.104	0.112
ZNF678	1.105	1.712	0.607	0.000	0.000
ZNF680	2.729	2.937	0.208	0.000	0.000
ZNF681	0.370	0.907	0.538	0.000	0.000
ZNF682	1.486	1.931	0.445	0.000	0.000
ZNF683	0.713	0.752	0.039	0.359	0.375
ZNF684	3.247	3.204	-0.042	0.350	0.366
ZNF687	2.815	4.025	1.211	0.000	0.000
ZNF688	3.284	3.393	0.109	0.002	0.003
ZNF689	2.087	2.533	0.446	0.000	0.000
ZNF69	2.490	2.500	0.010	0.843	0.850
ZNF691	3.234	3.895	0.661	0.000	0.000
ZNF692	3.397	4.652	1.255	0.000	0.000
ZNF696	1.749	2.945	1.196	0.000	0.000
ZNF697	2.060	2.600	0.540	0.000	0.000
ZNF699	0.585	0.705	0.120	0.000	0.000
ZNF7	2.176	3.122	0.946	0.000	0.000
ZNF70	0.530	0.797	0.266	0.000	0.000
ZNF700	3.224	3.487	0.262	0.000	0.000
ZNF701	0.867	1.169	0.302	0.000	0.000
ZNF703	1.846	2.582	0.736	0.000	0.000
ZNF704	1.761	2.457	0.696	0.000	0.000
ZNF706	3.981	5.088	1.108	0.000	0.000
ZNF707	1.597	2.549	0.952	0.000	0.000
ZNF708	1.325	1.603	0.278	0.000	0.000
ZNF709	0.300	0.362	0.062	0.000	0.000
ZNF71	0.819	1.406	0.586	0.000	0.000
ZNF710	2.485	3.236	0.751	0.000	0.000
ZNF711	0.693	1.089	0.396	0.000	0.000
ZNF713	0.402	0.722	0.320	0.000	0.000
ZNF714	0.436	0.921	0.486	0.000	0.000
ZNF717	1.190	1.334	0.144	0.000	0.000
ZNF718	1.076	1.692	0.616	0.000	0.000
ZNF720	1.416	1.854	0.438	0.000	0.000
ZNF721	2.637	3.143	0.506	0.000	0.000
ZNF726	0.518	0.968	0.450	0.000	0.000
ZNF732	0.140	0.236	0.097	0.000	0.000
ZNF736	0.729	1.151	0.423	0.000	0.000
ZNF737	1.056	1.448	0.391	0.000	0.000
ZNF738	0.763	1.428	0.665	0.000	0.000
ZNF74	1.423	2.334	0.910	0.000	0.000
ZNF740	2.342	3.267	0.925	0.000	0.000
ZNF746	2.240	3.067	0.827	0.000	0.000
ZNF747	3.859	4.216	0.356	0.000	0.000
ZNF749	1.599	2.121	0.522	0.000	0.000
ZNF75A	2.304	2.853	0.549	0.000	0.000

ZNF75D	2.345	2.691	0.346	0.000	0.000
ZNF76	3.361	4.075	0.714	0.000	0.000
ZNF761	2.111	2.913	0.802	0.000	0.000
ZNF764	2.230	3.005	0.775	0.000	0.000
ZNF765	0.817	1.349	0.531	0.000	0.000
ZNF766	1.865	2.601	0.736	0.000	0.000
ZNF768	4.661	5.416	0.755	0.000	0.000
ZNF77	1.477	2.110	0.632	0.000	0.000
ZNF770	3.415	3.793	0.379	0.000	0.000
ZNF771	1.805	2.136	0.331	0.000	0.000
ZNF772	1.066	1.433	0.367	0.000	0.000
ZNF773	0.794	1.089	0.295	0.000	0.000
ZNF774	0.362	0.558	0.195	0.000	0.000
ZNF775	1.743	2.647	0.904	0.000	0.000
ZNF776	2.307	2.671	0.364	0.000	0.000
ZNF777	2.994	3.726	0.732	0.000	0.000
ZNF778	0.810	1.106	0.297	0.000	0.000
ZNF780A	2.327	2.731	0.405	0.000	0.000
ZNF780B	1.711	2.177	0.466	0.000	0.000
ZNF781	0.550	0.993	0.444	0.000	0.000
ZNF782	1.186	1.662	0.476	0.000	0.000
ZNF783	1.503	1.986	0.483	0.000	0.000
ZNF784	3.028	3.679	0.651	0.000	0.000
ZNF785	1.390	2.069	0.679	0.000	0.000
ZNF786	1.920	2.568	0.649	0.000	0.000
ZNF787	4.371	5.210	0.840	0.000	0.000
ZNF789	1.691	2.411	0.719	0.000	0.000
ZNF79	2.028	2.542	0.514	0.000	0.000
ZNF790	2.213	2.304	0.090	0.002	0.002
ZNF791	2.213	2.601	0.388	0.000	0.000
ZNF792	1.051	1.832	0.781	0.000	0.000
ZNF793	0.414	0.734	0.320	0.000	0.000
ZNF799	1.631	1.900	0.269	0.000	0.000
ZNF80	0.401	0.325	-0.076	0.003	0.003
ZNF800	2.910	3.068	0.158	0.000	0.000
ZNF804A	0.154	0.313	0.159	0.000	0.000
ZNF805	1.945	2.228	0.283	0.000	0.000
ZNF808	1.370	1.402	0.032	0.440	0.456
ZNF81	0.887	1.189	0.302	0.000	0.000
ZNF813	0.727	1.138	0.411	0.000	0.000
ZNF814	1.913	2.222	0.308	0.000	0.000
ZNF816	1.319	1.726	0.407	0.000	0.000
ZNF821	1.479	2.032	0.552	0.000	0.000
ZNF823	2.391	2.795	0.404	0.000	0.000
ZNF827	0.630	0.639	0.009	0.790	0.799
ZNF829	0.615	0.860	0.246	0.000	0.000
ZNF83	2.509	2.983	0.474	0.000	0.000
ZNF830	3.559	3.950	0.391	0.000	0.000
ZNF831	0.545	0.415	-0.131	0.000	0.000
ZNF835	0.317	0.446	0.129	0.000	0.000
ZNF836	1.172	1.802	0.630	0.000	0.000
ZNF837	1.964	2.173	0.209	0.000	0.000

ZNF839	2.454	2.772	0.319	0.000	0.000
ZNF84	2.011	2.688	0.678	0.000	0.000
ZNF841	2.158	2.783	0.625	0.000	0.000
ZNF843	0.247	0.300	0.053	0.000	0.000
ZNF844	1.666	1.779	0.113	0.034	0.038
ZNF845	1.290	1.750	0.460	0.000	0.000
ZNF846	1.844	2.284	0.440	0.000	0.000
ZNF85	0.547	1.093	0.546	0.000	0.000
ZNF850	0.629	0.873	0.244	0.000	0.000
ZNF853	0.813	0.904	0.091	0.030	0.033
ZNF860	0.192	0.259	0.067	0.004	0.004
ZNF862	2.091	2.606	0.515	0.000	0.000
ZNF865	2.453	3.354	0.902	0.000	0.000
ZNF878	0.350	0.391	0.040	0.111	0.120
ZNF879	0.817	0.944	0.126	0.000	0.000
ZNF880	1.165	1.377	0.211	0.000	0.000
ZNF90	0.290	0.556	0.266	0.000	0.000
ZNF91	1.924	2.528	0.604	0.000	0.000
ZNF92	1.706	2.531	0.825	0.000	0.000
ZNF93	0.582	1.152	0.570	0.000	0.000
ZNFX1	3.204	3.720	0.516	0.000	0.000
ZNHIT1	6.288	6.415	0.127	0.000	0.000
ZNHIT2	3.737	4.392	0.654	0.000	0.000
ZNHIT6	2.245	2.696	0.451	0.000	0.000
ZNRD1	3.195	4.103	0.909	0.000	0.000
ZNRF1	2.786	3.272	0.486	0.000	0.000
ZNRF2	4.181	4.611	0.430	0.000	0.000
ZNRF3	1.702	2.159	0.457	0.000	0.000
ZP3	0.531	1.513	0.981	0.000	0.000
ZPLD1	0.294	0.441	0.148	0.005	0.005
ZRANB1	3.685	3.908	0.223	0.000	0.000
ZRANB2	4.552	5.135	0.583	0.000	0.000
ZRANB3	1.048	1.376	0.328	0.000	0.000
ZRSR2	3.184	3.555	0.371	0.000	0.000
ZSCAN12	1.118	1.631	0.513	0.000	0.000
ZSCAN16	2.237	3.270	1.033	0.000	0.000
ZSCAN18	2.846	2.743	-0.103	0.119	0.128
ZSCAN2	1.827	2.864	1.037	0.000	0.000
ZSCAN20	0.448	0.762	0.314	0.000	0.000
ZSCAN21	3.014	3.867	0.854	0.000	0.000
ZSCAN22	1.493	1.999	0.506	0.000	0.000
ZSCAN29	2.347	3.111	0.765	0.000	0.000
ZSCAN30	1.778	2.362	0.584	0.000	0.000
ZSCAN5A	0.907	1.283	0.377	0.000	0.000
ZSWIM1	2.312	3.160	0.847	0.000	0.000
ZSWIM3	1.583	2.125	0.542	0.000	0.000
ZSWIM4	1.628	1.973	0.345	0.000	0.000
ZSWIM5	0.872	1.881	1.009	0.000	0.000
ZSWIM6	2.823	2.552	-0.271	0.000	0.000
ZSWIM7	4.179	4.105	-0.074	0.024	0.027
ZW10	3.233	3.902	0.669	0.000	0.000
ZWILCH	1.639	2.771	1.132	0.000	0.000

ZWINT	1.909	4.227	2.318	0.000	0.000
ZXDA	1.208	1.472	0.264	0.000	0.000
ZXDB	2.779	3.305	0.525	0.000	0.000
ZXDC	3.168	3.573	0.405	0.000	0.000
ZYG11A	0.895	1.145	0.250	0.000	0.000
ZYG11B	3.551	3.666	0.115	0.003	0.003
ZYX	5.688	6.586	0.898	0.000	0.000
ZZEF1	2.550	2.698	0.148	0.000	0.000
ZZZ3	2.987	3.507	0.521	0.000	0.000

Supplemental Table 5. Results of cox regression analysis

Target gene	Hazard ratios	P.Value	95%CI
ACP1	1.963	0.000	1.579-2.441
ADA	1.445	0.000	1.263-1.653
ALDH18A1	1.427	0.000	1.238-1.645
ALDOA	1.436	0.000	1.306-1.579
APAF1	1.268	0.003	1.085-1.481
APEX1	1.904	0.000	1.555-2.332
APLP2	1.327	0.001	1.12-1.572
ARF6	1.217	0.047	1.003-1.477
ARPC2	1.668	0.000	1.402-1.985
ARPC3	1.648	0.000	1.356-2.001
ARPC4	1.787	0.000	1.478-2.16
ASNA1	1.503	0.000	1.236-1.828
AURKB	1.461	0.000	1.331-1.604
BRCC3	1.352	0.001	1.134-1.612
BRD9	1.644	0.000	1.384-1.952
CA9	1.165	0.000	1.117-1.214
CAD	1.523	0.000	1.325-1.752
CALM2	1.653	0.000	1.378-1.982
CAPN1	1.265	0.004	1.079-1.483
CAPNS1	1.377	0.001	1.148-1.652
CASK	1.295	0.001	1.114-1.505
CCNA2	1.466	0.000	1.334-1.611
CCNB1	1.579	0.000	1.426-1.749
CCT3	1.887	0.000	1.6-2.225
CDC25A	1.576	0.000	1.409-1.763
CDC25B	1.458	0.000	1.294-1.643
CDC42	1.617	0.000	1.328-1.969
CDC7	1.396	0.000	1.248-1.561
CDK1	1.500	0.000	1.361-1.653
CDK16	1.562	0.000	1.362-1.792
CDK5R1	1.575	0.000	1.334-1.86
CDK7	1.524	0.000	1.273-1.825
CENPE	1.574	0.000	1.388-1.785
CHEK1	1.627	0.000	1.431-1.85
CHEK2	1.211	0.004	1.065-1.378
CIT	1.643	0.000	1.426-1.893
CLIC1	1.510	0.000	1.355-1.682
CPNE1	1.425	0.000	1.229-1.652
CREB1	1.482	0.000	1.221-1.797
CSNK1E	1.401	0.000	1.209-1.624
CSNK1G1	1.508	0.000	1.242-1.832
CSNK2A1	1.565	0.000	1.327-1.845
DAGLB	1.553	0.000	1.241-1.944
DARS	1.610	0.000	1.321-1.961
DARS2	1.448	0.000	1.252-1.675
DCK	1.379	0.000	1.194-1.594
DDX39B	1.141	0.040	1.006-1.295
DNM2	1.348	0.001	1.131-1.608

DNMT1	1.458	0.000	1.288-1.652
DNMT3A	1.466	0.000	1.278-1.682
EED	1.616	0.000	1.309-1.995
EGLN3	1.272	0.000	1.187-1.364
EHMT2	1.549	0.000	1.34-1.791
EIF2S1	1.631	0.000	1.331-1.998
EIF2S3	1.541	0.000	1.308-1.815
EIF3F	1.918	0.000	1.551-2.372
ENO1	1.606	0.000	1.431-1.804
EWSR1	1.599	0.000	1.287-1.987
EZH2	1.594	0.000	1.411-1.802
FARSA	1.718	0.000	1.388-2.127
FARSB	2.515	0.000	2.049-3.088
FEN1	1.636	0.000	1.45-1.846
FGFR2	1.028	0.307	0.975-1.084
FGFR3	1.106	0.021	1.015-1.205
FKBP1A	1.865	0.000	1.559-2.229
FOXM1	1.488	0.000	1.355-1.635
FUBP1	1.449	0.000	1.248-1.683
G6PD	1.369	0.000	1.271-1.475
GARS	1.798	0.000	1.53-2.112
GLB1	1.389	0.000	1.156-1.669
GLTP	1.576	0.000	1.314-1.891
GNAS	1.238	0.005	1.067-1.436
GPI	1.266	0.002	1.087-1.475
GSK3A	1.726	0.000	1.423-2.093
GYG1	1.509	0.000	1.29-1.764
HDAC11	1.402	0.000	1.233-1.593
HDAC2	1.856	0.000	1.572-2.192
HM13	2.307	0.000	1.857-2.866
HMGB1	1.376	0.003	1.115-1.697
HMMR	1.463	0.000	1.315-1.627
HNRNPA2B1	1.843	0.000	1.465-2.319
HNRNPD	1.611	0.000	1.321-1.964
HNRNPH1	1.216	0.013	1.043-1.417
HNRNPH3	1.445	0.000	1.197-1.743
HNRNPK	1.624	0.000	1.284-2.055
HNRNPL	1.908	0.000	1.529-2.381
HP1BP3	1.293	0.001	1.104-1.514
HSP90AA1	1.490	0.000	1.276-1.739
HSP90AB1	1.493	0.000	1.29-1.728
HSPA13	1.378	0.000	1.197-1.586
HSPB1	1.263	0.000	1.126-1.418
HSPD1	1.836	0.000	1.539-2.19
HSPH1	1.367	0.000	1.2-1.556
IGF2R	1.243	0.003	1.079-1.432
IL12A	1.579	0.000	1.317-1.894
IMPDH1	1.324	0.000	1.209-1.451
IMPDH2	1.515	0.000	1.317-1.743
INCENP	1.451	0.000	1.28-1.645
IRAK1	1.406	0.000	1.235-1.601
ITPA	1.664	0.000	1.402-1.974

KDM1A	1.912	0.000	1.599-2.286
KDM3A	1.502	0.000	1.27-1.776
KDM5C	1.370	0.000	1.168-1.606
KDM6A	1.251	0.004	1.074-1.457
KIF11	1.488	0.000	1.334-1.66
KIF18A	1.611	0.000	1.426-1.819
KIF2C	1.526	0.000	1.391-1.674
LARS	1.651	0.000	1.353-2.015
LCMT1	1.507	0.000	1.265-1.794
LIMK1	1.307	0.000	1.169-1.461
M6PR	1.348	0.000	1.171-1.551
MAP2K2	1.681	0.000	1.397-2.022
MAP3K11	1.277	0.025	1.032-1.58
MAP3K4	1.391	0.001	1.153-1.679
MAP3K9	1.607	0.000	1.328-1.945
MAP4	1.337	0.001	1.121-1.596
MAP4K5	1.169	0.057	0.996-1.372
MAPK1	1.410	0.000	1.201-1.657
MAPK13	1.161	0.000	1.081-1.247
MAPK3	1.519	0.000	1.301-1.773
MAPKAPK5	2.265	0.000	1.803-2.845
MARK2	1.354	0.000	1.145-1.601
MAT2A	1.347	0.000	1.178-1.54
MMP14	1.191	0.000	1.095-1.297
MTAP	1.358	0.003	1.112-1.658
MYL6B	1.549	0.000	1.366-1.757
NEK2	1.457	0.000	1.325-1.603
NPM1	1.744	0.000	1.501-2.028
NUDT1	1.592	0.000	1.409-1.799
ODC1	1.329	0.000	1.19-1.483
P2RX4	1.481	0.000	1.283-1.709
PAFAH1B3	1.363	0.000	1.249-1.487
PAK2	1.521	0.000	1.247-1.855
PARP2	1.532	0.000	1.27-1.849
PDE9A	1.174	0.000	1.078-1.279
PHB2	1.533	0.000	1.229-1.912
PI4KA	1.302	0.001	1.118-1.516
PIK3R2	1.265	0.003	1.081-1.48
PKN1	1.413	0.000	1.243-1.606
PLCB3	1.434	0.000	1.24-1.658
PLK1	1.552	0.000	1.408-1.711
PLK4	1.546	0.000	1.353-1.766
POLA1	1.631	0.000	1.404-1.896
POLD1	1.733	0.000	1.507-1.993
PPARG	1.369	0.000	1.216-1.54
PPAT	1.697	0.000	1.436-2.006
PPIH	1.785	0.000	1.503-2.119
PPP2R1A	1.618	0.000	1.367-1.914
PPP5C	1.478	0.000	1.237-1.765
PRC1	1.498	0.000	1.354-1.658
PRKAG1	1.858	0.000	1.439-2.4
PRKCD	1.384	0.000	1.217-1.575

PRMT1	1.565	0.000	1.349-1.815
PSEN1	1.297	0.021	1.041-1.617
PSMA4	1.834	0.000	1.445-2.327
PSMB5	2.117	0.000	1.722-2.601
PTGES3	2.056	0.000	1.658-2.549
RAC1	1.934	0.000	1.607-2.328
RAD51	1.534	0.000	1.37-1.717
RALA	1.855	0.000	1.546-2.226
RAN	1.870	0.000	1.595-2.191
RANBP1	2.018	0.000	1.703-2.391
RHEB	1.806	0.000	1.47-2.218
RHOA	1.712	0.000	1.372-2.136
RIOK1	1.714	0.000	1.452-2.023
ROCK1	1.192	0.031	1.016-1.4
RPIA	1.759	0.000	1.482-2.087
RPL15	1.592	0.000	1.313-1.931
RPL23	1.551	0.000	1.332-1.805
RPL4	1.571	0.000	1.334-1.849
RPS19	1.355	0.000	1.19-1.544
RPSA	1.542	0.000	1.35-1.761
RRM1	1.581	0.000	1.366-1.83
SF3A3	1.959	0.000	1.607-2.388
SFPQ	1.840	0.000	1.529-2.214
SLC25A6	1.400	0.000	1.18-1.661
SLC2A1	1.321	0.000	1.23-1.418
SMARCA4	1.593	0.000	1.355-1.871
SNAP25	1.089	0.022	1.012-1.17
SPHK1	1.169	0.000	1.101-1.242
SRC	1.225	0.000	1.117-1.344
STIP1	1.928	0.000	1.629-2.282
STK24	1.436	0.000	1.252-1.648
STK39	1.164	0.000	1.074-1.262
TACC3	1.582	0.000	1.414-1.771
TFRC	1.413	0.000	1.254-1.592
TOP1	1.524	0.000	1.26-1.844
TOP2A	1.360	0.000	1.254-1.474
TOP2B	1.570	0.000	1.304-1.89
TPM3	1.669	0.000	1.417-1.965
TRAPPC4	1.673	0.000	1.406-1.99
TTK	1.546	0.000	1.392-1.717
TUBA1C	1.467	0.000	1.3-1.656
TUBB	1.435	0.000	1.253-1.644
TYMS	1.393	0.000	1.266-1.533
TYRO3	1.371	0.000	1.256-1.496
UBA1	1.617	0.000	1.326-1.971
UBE2D2	1.966	0.000	1.557-2.484
UBE2N	1.865	0.000	1.492-2.331
USP1	1.517	0.000	1.314-1.75
USP13	1.502	0.000	1.275-1.77
USP14	1.560	0.000	1.292-1.884
VARS	1.648	0.000	1.392-1.952
VDAC1	1.595	0.000	1.3-1.957

VEGFB	1.154	0.000	1.065-1.25
WEE1	1.324	0.000	1.163-1.508
YWHAB	1.972	0.000	1.612-2.412
YWHAH	1.647	0.000	1.392-1.949
YWHAQ	1.695	0.000	1.45-1.981

Supplemental Table 6. Gene dependency of target genes

CRISPR-based dependency				RNAi-based dependency		
Target gene	CERES score	Rank		Target gene	DEMETER score	Rank
RAN	-2.272	1		RAN	-1.507	1
UBA1	-1.999	2		PSMA4	-1.420	2
RPL23	-1.979	3		SFPQ	-1.305	3
WEE1	-1.977	4		VARS	-1.260	4
FARSB	-1.948	5		KIF11	-1.156	5
EIF2S1	-1.947	6		PSMB5	-1.151	6
RPL4	-1.865	7		PLK1	-1.144	7
CCT3	-1.854	8		UBA1	-1.134	8
KIF11	-1.803	9		POLA1	-1.117	9
HNRNPK	-1.780	10		RRM1	-1.105	10
PSMB5	-1.762	11		TUBB	-1.061	11
EIF2S3	-1.752	12		CCT3	-1.037	12
CDK1	-1.750	13		DARS	-1.009	13
PLK1	-1.740	14		PHB2	-0.962	14
RRM1	-1.720	15		CDK1	-0.941	15
CDK7	-1.715	16		RAD51	-0.911	16
SFPQ	-1.683	17		DDX39B	-0.845	17
TOP2A	-1.654	18		HSPD1	-0.822	18
TUBB	-1.623	19		KIF18A	-0.631	19
CHEK1	-1.577	20		PPP2R1A	-0.629	20
CDC7	-1.483	21		PRMT1	-0.609	21
PSMA4	-1.420	22		ALDOA	-0.587	22
PRC1	-1.409	23		TOP2A	-0.551	23
RPS19	-1.395	24		TACC3	-0.532	24
CCNA2	-1.391	25		CDC7	-0.510	25
AURKB	-1.386	26		POLD1	-0.505	26
ALDOA	-1.375	27		INCENP	-0.496	27
KIF18A	-1.345	28		RHEB	-0.492	28
PRMT1	-1.317	29		CCNA2	-0.485	29
DNM2	-1.287	30		TFRC	-0.476	30
VARS	-1.274	31		DNMT1	-0.474	31
TRAPPC4	-1.268	32		FUBP1	-0.470	32
HNRNPH1	-1.249	33		WEE1	-0.468	33
INCENP	-1.249	34		CENPE	-0.439	34
SF3A3	-1.245	35		EWSR1	-0.427	35
PPP2R1A	-1.227	36		TTK	-0.405	36
RPL15	-1.197	37		PRKAG1	-0.397	37
GARS	-1.171	38		DNM2	-0.390	38
PHB2	-1.165	39		AURKB	-0.375	39
DARS	-1.154	40		CAD	-0.365	40
HSPD1	-1.137	41		TOP1	-0.362	41
CDC42	-1.124	42		CALM2	-0.352	42
POLD1	-1.101	43		RALA	-0.333	43
UBE2N	-1.084	44		CHEK1	-0.328	44
PLK4	-1.074	45		ARPC4	-0.321	45
RIOK1	-1.048	46		PI4KA	-0.297	46

EIF3F	-1.027	47		KDM1A	-0.296	47
PI4KA	-1.002	48		RIOK1	-0.291	48
ARPC4	-0.973	49		NPM1	-0.287	49
EWSR1	-0.963	50		EGLN3	-0.280	50
TUBA1C	-0.957	51		FKBP1A	-0.272	51
RAD51	-0.952	52		CCNB1	-0.263	52
RPSA	-0.952	53		MAT2A	-0.257	53
ASNA1	-0.948	54		HNRNPA2B1	-0.253	54
FARSA	-0.900	55		MAPKAPK5	-0.241	55
MAT2A	-0.895	56		CDC25B	-0.238	56
LARS	-0.895	57		PRKCD	-0.235	57
POLA1	-0.880	58		PLK4	-0.211	58
FEN1	-0.867	59		FOXM1	-0.207	59
TTK	-0.834	60		CDC42	-0.205	60
CCNB1	-0.833	61		HSP90AB1	-0.205	61
HNRNPL	-0.808	62		SRC	-0.186	62
TFRC	-0.805	63		SMARCA4	-0.186	63
ARPC3	-0.782	64		IMPDH2	-0.185	64
ENO1	-0.756	65		PPP5C	-0.183	65
TOP1	-0.722	66		RAC1	-0.174	66
CENPE	-0.716	67		NEK2	-0.171	67
RAC1	-0.708	68		PAK2	-0.165	68
NPM1	-0.700	69		TYMS	-0.163	69
HMGB1	-0.641	70		HSP90AA1	-0.160	70
PPIH	-0.586	71		HM13	-0.160	71
SLC2A1	-0.574	72		EHMT2	-0.158	72
TACC3	-0.572	73		MARK2	-0.155	73
DDX39B	-0.558	74		USP1	-0.154	74
RHEB	-0.555	75		CLIC1	-0.142	75
ARPC2	-0.547	76		IMPDH1	-0.136	76
GPI	-0.539	77		APLP2	-0.133	77
LCMT1	-0.526	78		CDC25A	-0.129	78
KIF2C	-0.521	79		CDK7	-0.121	79
IMPDH2	-0.496	80		CASK	-0.120	80
CIT	-0.494	81		UBE2N	-0.119	81
TYMS	-0.488	82		HMMR	-0.118	82
DARS2	-0.471	83		CAPNS1	-0.113	83
DNMT1	-0.451	84		ENO1	-0.111	84
PPAT	-0.450	85		KDM5C	-0.110	85
VDAC1	-0.410	86		HMGB1	-0.104	86
FOXM1	-0.404	87		KDM3A	-0.103	87
ARF6	-0.391	88		LIMK1	-0.096	88
SMARCA4	-0.385	89		USP14	-0.094	89
HSP90AB1	-0.371	90		HDAC11	-0.090	90
MAP3K11	-0.368	91		STK24	-0.080	91
RPIA	-0.367	92		YWHAH	-0.076	92
EED	-0.357	93		TOP2B	-0.074	93
RHOA	-0.350	94		PPARG	-0.074	94
MARK2	-0.342	95		APEX1	-0.072	95
PAK2	-0.341	96		NUDT1	-0.071	96
CDC25B	-0.337	97		VEGFB	-0.069	97
ALDH18A1	-0.326	98		MTAP	-0.069	98

PIK3R2	-0.301	99		HNRNPD	-0.065	99
CDC25A	-0.295	100		TYRO3	-0.064	100
FUBP1	-0.282	101		PPAT	-0.054	101
NEK2	-0.278	102		KDM6A	-0.054	102
RANBP1	-0.275	103		MAPK1	-0.053	103
HM13	-0.272	104		SLC2A1	-0.043	104
KDM1A	-0.265	105		ALDH18A1	-0.042	105
MYL6B	-0.261	106		CSNK1G1	-0.039	106
HNRNPA2B	-0.260	107		TPM3	-0.039	107
IMPDH1	-0.255	108		GNAS	-0.038	108
CAPNS1	-0.254	109		DCK	-0.036	109
CAPN1	-0.254	110		PSEN1	-0.032	110
HSPA13	-0.249	111		PKN1	-0.030	111
FKBP1A	-0.242	112		GPI	-0.025	112
CAD	-0.237	113		SPHK1	-0.020	113
MAPK1	-0.234	114		PLCB3	-0.020	114
USP1	-0.231	115		FGFR3	-0.019	115
STK39	-0.226	116		CREB1	-0.018	116
MAP2K2	-0.220	117		BRD9	-0.017	117
MAPK3	-0.208	118		CSNK2A1	-0.015	118
HNRNPD	-0.208	119		CAPN1	-0.014	119
ITPA	-0.202	120		KIF2C	-0.014	120
PTGES3	-0.201	121		MAP3K4	-0.013	121
TPM3	-0.200	122		GSK3A	-0.012	122
P2RX4	-0.191	123		STK39	-0.011	123
STIP1	-0.185	124		FGFR2	-0.005	124
KDM5C	-0.175	125		G6PD	-0.003	125
HNRNPH3	-0.170	126		PIK3R2	-0.002	126
PRKAG1	-0.165	127		SNAP25	-0.002	127
CSNK2A1	-0.165	128		MAP3K11	-0.002	128
DAGLB	-0.164	129		MMP14	0.000	129
GLTP	-0.159	130		MAP4K5	0.006	130
YWHAH	-0.151	131		ITPA	0.007	131
MAP4	-0.146	132		PDE9A	0.014	132
EHMT2	-0.145	133		MAPK3	0.015	133
GLB1	-0.142	134		BRCC3	0.017	134
RALA	-0.138	135		MAP3K9	0.029	135
MAPKAPK5	-0.135	136		EZH2	0.031	136
HSP90AA1	-0.133	137		YWHAB	0.033	137
SRC	-0.130	138		EED	0.046	138
SPHK1	-0.129	139		TRAPPC4	0.051	139
G6PD	-0.129	140		APAF1	0.051	140
PRKCD	-0.127	141		IRAK1	0.057	141
TYRO3	-0.123	142		UBE2D2	0.063	142
VEGFB	-0.117	143		DNMT3A	0.064	143
CSNK1G1	-0.114	144		CHEK2	0.067	144
PSEN1	-0.101	145		ARF6	0.070	145
ROCK1	-0.101	146		RPIA	0.084	146
PAFAH1B3	-0.101	147		HSPB1	0.084	147
GYG1	-0.100	148		USP13	0.085	148
TOP2B	-0.098	149		CSNK1E	0.094	149
CLIC1	-0.083	150		RHOA	0.097	150

PLCB3	-0.078	151		HDAC2	0.113	151
CPNE1	-0.069	152		MAPK13	0.124	152
CA9	-0.069	153		FEN1	0.158	153
APEX1	-0.060	154		ROCK1	0.162	154
NUDT1	-0.060	155				
IL12A	-0.058	156				
CSNK1E	-0.058	157				
CREB1	-0.055	158				
CALM2	-0.040	159				
HP1BP3	-0.036	160				
HSPH1	-0.036	161				
APAF1	-0.029	162				
USP14	-0.027	163				
BRCC3	-0.022	164				
PKN1	-0.022	165				
USP13	-0.020	166				
FGFR2	-0.020	167				
GNAS	-0.019	168				
MMP14	-0.018	169				
MAPK13	-0.017	170				
PPP5C	-0.011	171				
LIMK1	-0.007	172				
M6PR	0.002	173				
MTAP	0.005	174				
CDK16	0.006	175				
FGFR3	0.007	176				
HDAC11	0.011	177				
MAP3K4	0.017	178				
HMMR	0.025	179				
ADA	0.042	180				
BRD9	0.044	181				
HDAC2	0.058	182				
SLC25A6	0.060	183				
YWHAB	0.062	184				
MAP3K9	0.065	185				
KDM3A	0.065	186				
PDE9A	0.068	187				
EZH2	0.068	188				
EGLN3	0.076	189				
IGF2R	0.079	190				
PARP2	0.081	191				
GSK3A	0.088	192				
ODC1	0.091	193				
PPARG	0.092	194				
SNAP25	0.103	195				
DCK	0.126	196				
UBE2D2	0.131	197				
APLP2	0.143	198				
KDM6A	0.149	199				
ACP1	0.156	200				
DNMT3A	0.157	201				
CASK	0.164	202				

CDK5R1	0.169	203				
IRAK1	0.172	204				
STK24	0.178	205				
HSPB1	0.187	206				
YWHAQ	0.245	207				
CHEK2	0.247	208				
